# A comparative analysis of cell surface targeting aptamers

**DOI:** 10.1038/s41467-021-26463-w

**Published:** 2021-11-01

**Authors:** Linsley Kelly, Keith E. Maier, Amy Yan, Matthew Levy

**Affiliations:** 1 Department of Biochemistry, Albert Einstein College of Medicine, 1301 Morris Park Ave, Bronx, NY 10461 USA; 2 Present Address: Department of Surgery, Duke University School of Medicine, Durham, NC 27710 USA; 3 Present Address: EpiCypher Inc, Durham, NC 27709 USA; 4 Present Address: Creyon Bio, Inc., San Diego, CA 92121 USA

**Keywords:** Nucleic-acid therapeutics, Chemical tools

## Abstract

Aptamers represent a potentially important class of ligands for the development of diagnostics and therapeutics. However, it is often difficult to compare the function and specificity of many of these molecules as assay formats and conditions vary greatly. Here, with an interest in developing aptamer targeted therapeutics that could effectively deliver cargoes to cells, we chemically synthesize 15 aptamers that have been reported to target cell surface receptors or cells. Using standardized assay conditions, we assess each aptamer’s binding properties on a panel of 11 different cancer cell lines, correlate aptamer binding to antibody controls and use siRNA transfection to validate each aptamer’s binding to reported target receptors. Using a subset of these molecules known to be expressed on prostate cancers, we use near-infrared in vivo imaging to assess the tumor localization following intravenous injection. Our data demonstrate some surprising differences in the reported specificity and function for many of these molecules and raise concerns regarding their cell targeting capabilities. They also identify an anti-human transferrin aptamer, Waz, as a robust candidate for targeting prostate cancers and for future development of aptamer-based therapeutics.

## Abstract (web summary)

Aptamers could potentially be widely used in therapy and diagnostics. Here the authors use standardised assay conditions to compare aptamer properties in tumour targeting.

## Introduction

The advancement of many biomedical reagents and therapeutics relies on the development of macromolecular ligands with high affinity and specificity. Over the past 40 years, this role has been largely dominated by the production of monoclonal antibodies. With high affinity (nM–pM) and high specificity, antibodies have revolutionized biomedical research and can be potent modulators of target protein activity, which has led to a revolution in drug development. During pre-clinical development, antibodies undergo thorough studies including target validation to ensure target specificity. However, as highlighted by multiple commentaries, this level of rigorous characterization has not been consistently applied to commercially produced antibody research tools^1,2^. Problems with commercial antibody reagents has led to a growing consensus that researchers worldwide should forego antibodies produced from traditional sources (e.g., animal sera, hybridomas) and instead rely on recombinant sources where sequences can be validated, and production can be more finely controlled^1^. Alternately, it has been suggested that researchers turn to the use of other recombinant protein scaffolds (e.g., affibodies) or nucleic acid based agents (e.g., aptamers)^1^.

Nucleic acid aptamers offer many potential advantages to proteins when developing ligands for use as both research tools and as therapeutics. Aptamers can achieve similar binding affinities to monoclonal antibodies, but unlike antibodies, they can be easily stored at room temperature and reversibly thermally denatured in solution. As nucleic acids, they can be chemically synthesized in large quantities by solid phase synthesis, are easily purified, and they are readily amenable to site-specific chemical conjugation^3–5^. For in vivo applications, aptamers are readily cleared from circulation, *t*_1/2_ = ~10 min, after intravenous administration, a consequence of their small size, ~10–20 kDa^4,6^. Clearance rates, however, can be controlled via conjugation to agents such as cholesterol or high molecular weight PEG, which can significantly increase circulation times to ~10–24 h^4^. Additionally, the mean residence time of these molecules can be prolonged with subcutaneous dosing^7^. Thus, the properties of these molecules can be readily tuned to fit various applications in ways not possible with antibodies.

There is now an increasing number of aptamers reported in the literature against targets ranging from small molecules to proteins^8,9^. More recently, there has been increasing interest in the development of aptamers that target cell surface receptors for use in the targeted delivery of therapeutics such as toxins, cytotoxic drugs, or therapeutic oligonucleotides. In particular, there are a number of aptamers reported in the literature that putatively bind therapeutically relevant cancer markers including: the prostate specific membrane antigen (PSMA)^10–12^, human transferrin receptor (hTfR)^13,14^, the epidermal growth factor receptor (EGFR)^15–17^, and others^18–23^. Other aptamers have been selected using whole-cell SELEX against cancer cell lines, and though their targets are currently unknown, they are reported to show increased uptake by target cells^24,25^.

Although many receptor or cell targeting aptamers have been reported as tools for various cell staining and cell capture experiments (reviewed in Abraham and Maliekal^26^) or for the delivery of therapeutic cargoes (reviewed in Zhou and Rossi^27^), a survey of the literature reveals that the characterization and validation of many of these molecules is largely confounded by a lack of uniform assessment. Assay formats for reported cell surface targeting aptamers vary greatly and range from using radioactive or fluorescent ‘bulk-labeling’ of cells, to quantitative PCR, fluorescence microscopy, or flow cytometry. Additionally, even within a given assay format, conditions between publications vary. For example, some studies fluorescently label aptamers via alkylation while others use site-specific conjugation, making it difficult to assess the relative function and activity of different aptamers. Moreover, while many potentially therapeutic aptamers have been characterized “functionally” for their ability to affect downstream cellular functions, growth, or even for the ability to deliver therapeutic cargoes and payloads, many studies have not thoroughly examined the first critical step in aptamer development: assessing whether the aptamer effectively and specifically binds the target. Finally, it is worth noting that some studies lack critical or make use of inappropriate negative controls; in particular, they fail to use a non-targeting control aptamer.

With an interest in developing aptamer targeted therapeutics that could effectively deliver cargoes to cells, we set out to establish a standardized set of assays and conditions that can be used to evaluate cell surface receptor binding aptamers in a consistent and comparable manner. We chemically synthesized 15 aptamers taken from the literature or developed in our own lab that are reported to target cancer relevant cell surface markers and surveyed them for their ability to specifically bind their molecular targets both in vitro cell culture and in vivo. The targets included: PSMA, EGFR, hTfR, HER2, AXL, EpCAM, and protein tyrosine kinase 7 (PTK7) (Table 1).

**Table 1:** Aptamers used in this study.

Chemistry	Aptamer	Target	SELEX	Ref.
fYrR	A9.min	PSMA	Protein	^11,28,29^
	A10-3	PSMA	Protein	^11^
	A10-3.2	PSMA	Protein	^10^
	Waz	hTfR	Protein/cells	^14^
	C2	hTfR	Protein/cells	^13^
	E07.min	EGFR	Protein	^17^
	CL4	EGFR	Cells	^16^
	GL21.T	AXL	Cells	^18^
	EpDT3	EpCAM	Cells	^21^
	SE15-8mini	HER2	Protein	^20^
	C1	Cells	Cells	^24^
	C36	Control	Designed	^31^
rGmH	XEO2-mini	Cells	Cells	^25^
DNA	AS1411	Nucleolin	Designed	^22^
	Sgc8c	PTK7	Cells	^23^
	2-2(t)	HER2	Peptide/cells	^19^

All aptamers were synthesized in house with a terminal 5′ thiol for dye conjugation.

*fYrR* = 2′ Fluoro C and U, 2′OH A and G; *rGmH* = 2′OH G and 2′OMe A, C and U.

In vitro, we performed analyses under standardized conditions using flow cytometry on 11 different cancer cell lines (Table 2) using site-specific fluorescently labeled aptamers. Specific binding for each cell line was correlated with antibody binding to the same target. Target specificity was further validated using siRNA transfection to knock down target protein expression. Then, using a subset of the molecules known to be expressed on prostate cancers, we performed an in vivo analysis using near infrared (NIR) imaging. Of the 15 aptamers we tested, only five demonstrated receptor specific activity on cells in vitro. These included the 2′-fluoro pyrimidine (2′F) modified anti-EGFR aptamer, E07^17^, an anti-PSMA aptamer, A9.min^28,29^, two anti-hTfR aptamers, C2.min^13^ and Waz^14^, and one DNA aptamer against PTK7^30^. Finally, using a subset of molecules with the potential for targeting prostate cancers (those targeting hTfR and PSMA), we screened molecules for the ability to localize to prostate tumors following systemic administration. Only the anti-hTfR aptamer, Waz, proved capable of localizing to tumors.

**Table 2:** Cell lines used in this study.

Cell Line	Cancer
HeLa	Cervical
HeLa PSMA	Cervical
PC3	Prostate
PC3 PSMA	Prostate
LNCaP	Prostate
22RV1	Prostate
A549	Lung
HT29	Colon
JurKat	Leukemia
MCF7	Breast
SKBr3	Breast

Our data demonstrate some striking inconsistencies in the reported efficacy and functionality of many of the aptamers we tested. They also demonstrate a disconnect between function in vitro and in vivo; target binding in and of itself is insufficient to assure in vivo activity. Altogether, our work provides standardized methods and guidance for assessing and validating aptamer function both in vitro and in vivo, which will help ensure reproducibility and effectiveness of aptamer function into the future. More immediately, our results should aid researchers interested in the pursuit of aptamers for the development of targeted therapeutics, identifying candidate molecules that have potential for further development and, perhaps more importantly, those that do not warrant attention. In this way we hope this work serves in part as a catalyst to move towards the production of more robust cell-targeting aptamers.

## Results

### Optimization of cell staining parameters for validating aptamer function by flow cytometry

As polyanions, aptamers (and oligonucleotides in general) have the potential to interact with proteins or lipids on the cell surface through charge–charge interactions. This can lead to non-specific uptake into cells, particularly in culture following extended incubation. Indeed, in previous work, we have observed differential non-specific uptake of aptamers between different cell lines^13,14,24,31^. To elucidate this in a more systematic fashion, we performed an initial set of experiments on HeLa cells, a PSMA negative cell line, using three aptamers that should show no binding to this cell type: two anti-PSMA aptamers, the minimized variants of A9 (A9.min)^28,29^ and A10 (A10.3)^11^, and a non-specific control sequence C36^31^. As a positive control for binding, we used the anti-TfR aptamer, C2.min, which we have previously reported to bind to and be internalized by HeLa cells^13^. Additionally, experiments were performed using AS1411, a DNA aptamer reported to bind nucleolin^32^, but whose ability to be taken up by cells was later demonstrated to be nucleolin independent^33^.

**Fig. 1:**
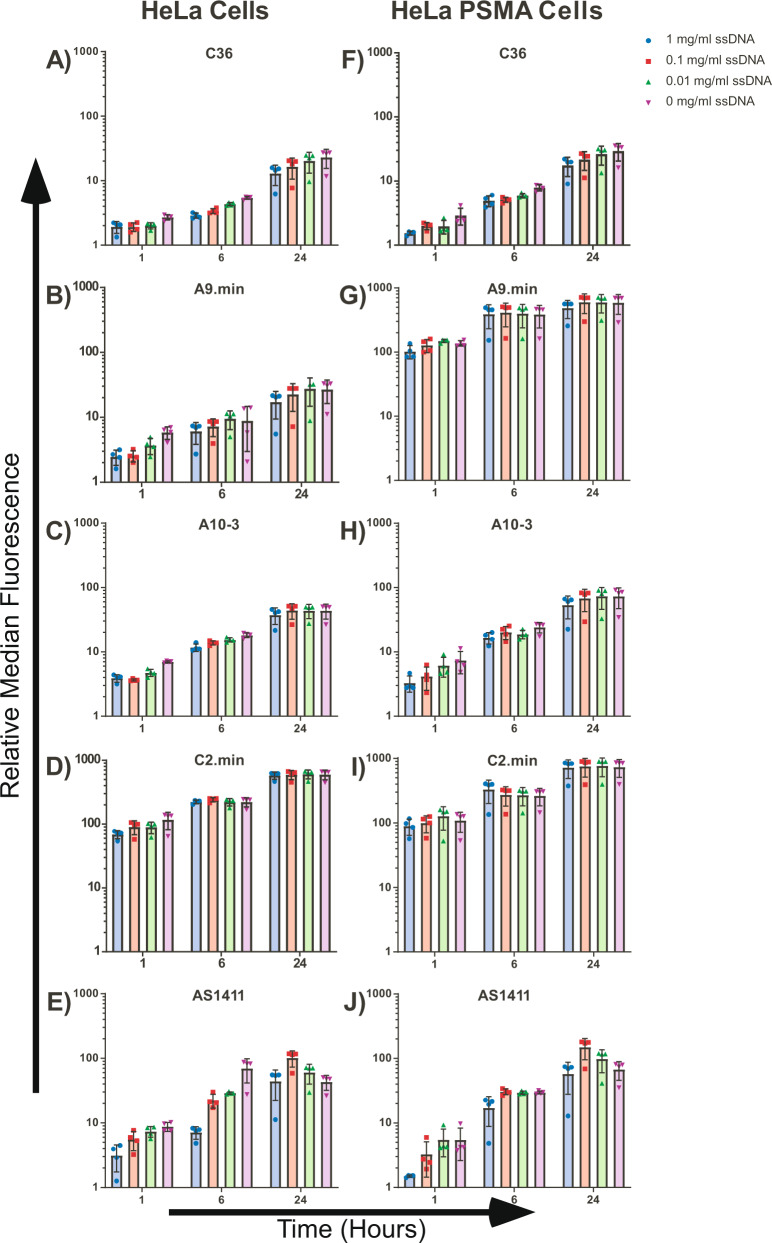
Effects of time and blocking on aptamer uptake. Aptamers labeled with DY650 were incubated at 37 °C with HeLa and HeLa-PSMA cell lines at 500 nM in full growth media with increasing concentrations of ssDNA as a non-specific inhibitor. Cells were allowed to bind/internalize the aptamers for 1, 6, or 24 h. The median fluorescence signal of each aptamer is shown relative to unstained cells. HeLa cells with **A** C36, **B** A9.min, **C** A10-3, **D** C2.min, **E** AS1411 and HeLa PSMA cells with **F** C36, **G** A9.min, **H** A10-3, **I** C2.min, **J** AS1411. Bar color corresponds to the concentration of added ssDNA. Purple = no competitor, green = 0.01 mg/mL, red = 0.1 mg/mL, and blue = 1.0 mg/mL. Flow cytometry histograms for the corresponding data can be found in Supplementary Figs. 2–6. Data represents the mean of four experimental replicates ±SD. Source data are provided as a Source Data file.

In short, fluorescently labeled aptamers were incubated with cells in culture media containing 10% FBS in the presence of increasing concentrations of a non-specific competitor (ssDNA) for increasing periods of time at 37 °C followed by analysis using flow cytometry (Fig. 1 and Supplementary Figs. 2–6). For all aptamers tested, cell staining increased with incubation time, even those lacking target receptors on HeLa cells (C36, A9.min, and A10.3), with significant levels of staining (>5-fold over unstained cells) observed with prolonged exposure (>6 h; Fig. 1A–C). Overall, the binding observed from the different non-specific molecules varied slightly, with the anti-PSMA aptamer A10.3 showing the greatest amount of non-specific binding, followed by A9.min and C36 (Fig. 1).

As might be predicted, non-specific interactions with cells could be inhibited to some extent by the presence of a non-specific competitor, ssDNA, with incubations performed in the presence of 1 mg/mL ssDNA providing the greatest amount of inhibition. However, even at these high concentrations, significant non-specific uptake (>5-fold) was observed after prolonged (>6 h) incubation with cells. Interestingly, when we examined the effects of both time and ssDNA concentration on uptake by a specific interaction using C2.min, while increased time led to increased signals, the presence of ssDNA had essentially no effect on cell staining (Fig. 1D). The presence of the non-specific competitor was also found to reduce uptake of AS1411 (Fig. 1E) during the shortest incubation time. However, a unique change was seen following longer incubation times (6 and 24 h), where the presence of the non-specific competitor appeared to modestly increase AS1411 uptake. This may be related to increased uptake due to macropinocytosis, as described by Reyes-Reyes et al.^33^.

To further assess the nature of non-specific uptake, we extended our analysis to HeLa-PSMA cells, HeLa cells engineered to stably express human PSMA. On these cells, the non-targeting control aptamer, C36, displayed slightly higher, albeit similar binding, and uptake characteristics to the parental line: cell staining increased with time, and could be blocked to some degree by the inclusion of a non-specific competitor (Fig. 1F). In contrast, the anti-PSMA aptamer, A9.min, displayed dramatically different binding characteristics, demonstrating a substantial increase in cell labeling consistent with the expression of the target receptor and displaying competitor-independent staining – uptake characteristics which paralleled those of the specific cell target interaction of C2.min (Fig. 1G).

In contrast, the anti-PSMA aptamer A10.3 did not demonstrate any significant increase in cell labeling when we performed experiments on HeLa-PSMA cells (Fig. 1H). Both the magnitude of binding and the observed binding characteristics in the presence of non-specific competitor closely mimicked those observed on the non-PSMA-expressing parental HeLa cells (compare Fig. 1C, H, also see Supplementary Figs. 2–6).

Finally, we assessed the stability of each of these molecules in media to ensure that degradation during the course of the assay was not confounding these results, especially at longer timepoints. With the exception of AS1411, the aptamers tested were stable showing little to no degradation at 24 h (Supplementary Fig 7). In the case of AS1411, ~80% full length remained following a 24 h incubation. Interestingly, while the other 2′F modified aptamers we tested also remained largely intact (>90% at 24 h), the other DNA aptamers we tested (2-2(t) and SGC8c) showed even greater levels of degradation (40% and 20% remaining, respectively) at 24 h (Supplementary Fig. 7).

Taken as a whole, these results suggest that differentiating between specific and non-specific cell staining was best achieved using short incubation times (~1 h) in the presence of a non-specific competitor (~1 mg/mL) to minimize non-specific uptake and, in the case of DNA aptamers, degradation.

Drawing from these results, we extended our analysis of non-specific interactions to a panel of 11 different cancer cell lines (Table 2). For simplicity, in these experiments, we performed assays using only the non-specific control aptamer, C36. Cell labeling was performed in full media containing 10% FBS with incubation times at 37 °C limited to 1 h. Additionally, we only performed assays under two sets of blocking conditions: no blocking or 1 mg/mL ssDNA. However, to assess how non-specific interactions varied with the concentration of the labeled aptamer, we performed assays with 50, 100, 500, or 1000 nM labeled aptamer.

**Fig. 2:**
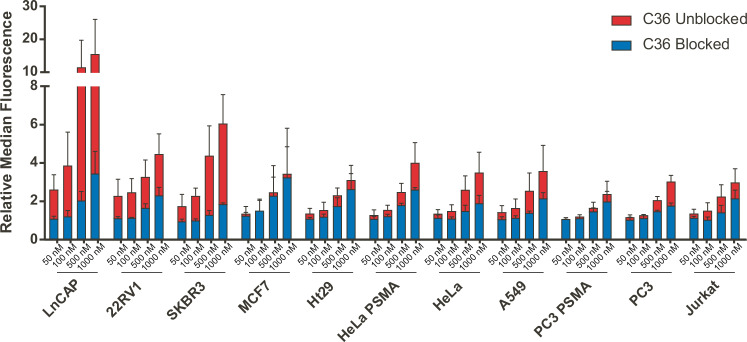
Variation of non-specific binding/uptake across cell types. The indicated cell line was incubated at 37 °C with 50 nM, 100 nM, 500 nM, and 1000 nM of a DY650 labeled non-binding control aptamer, C36, with or without 1 mg/mL ssDNA in full growth media for 1 h. The fold change in median fluorescence relative to unstained cells are shown. Samples with ssDNA blocking added (blue) are superimposed on data derived from the same experiment performed in the absence of ssDNA (red). Data represent three independent experimental replicates. Source data are provided as a Source Data file.

As shown in Fig. 2 and Supplementary Figs. 8 and 9, in the absence of any blocking agents, different cell lines displayed appreciably different levels of non-specific binding. The most dramatic effects were observed for incubations performed in the presence of higher concentrations of aptamer (500 and 1000 nM) and on the ‘stickiest’ cell line, LNCaP prostate cancer cells, which displayed ~10-fold more non-specific binding than PC3 prostate cancer cells or Jurkat T lymphocyte cells. In contrast, when cells were blocked by the addition of 1 mg/mL ssDNA, both the total level of background binding and the relative difference in binding between cell types were reduced. At 50 and 100 nM C36, there was virtually no difference in binding between cell-types. At 500 nM C36, the biggest difference was <2-fold, and at 1 μM, the maximum difference was slightly higher than 2-fold. Thus, in the presence of 1 mg/mL ssDNA blocking agent, only slight differences in non-specific binding was observed between cell lines.

### Comparative analysis of cell targeting aptamers in vitro

Having determined conditions to minimize background binding and uptake by cells, we extended our analysis further by testing all of the aptamers listed in Table 1 for the ability to bind each of the cell lines in Table 2. For these experiments, we utilized three sets of standard conditions. In the first, termed ‘internalization and binding with blocking’, we assayed aptamers as described above, by adding labeled molecules directly to cell culture media appropriate for a given cell type containing 10% FBS and 1 mg/mL ssDNA for 1 h at 37 °C. However, because many reports do not include blocking agents in their experiments, we also performed assays under a second set of identical conditions, but in the absence of blocking agents, termed ‘internalization and binding without blocking’. We note that both of these assay formats do not distinguish between surface bound aptamer and aptamers that have been internalized; the observed signal is a combination of the two. Finally, to parallel staining protocols commonly used for antibodies, we performed experiments under a third set of conditions, termed ‘binding’, in which the labeled aptamers were added to cells suspended in FACS buffer (HBSS, 1% BSA) supplemented with 1 mg/mL ssDNA, and incubated for 30 min at room temperature. In all cases, following incubation and washing, the treated cells were analyzed by flow cytometry. For internalization and binding experiments (both with and without blocking), aptamer function was assessed over a range of concentrations (50 nM, 100 nM, 500 nM, and 1 μM) that would be expected to provide an observable binding signal based on the published affinities of the aptamers tested (Supplementary Table 5). For experiments using the binding protocol, assays were only performed at 500 nM aptamer, again a concentration that would be expected to provide an observable binding signal based on the published affinities of the aptamers tested (Supplementary Table 5). In parallel with these experiments, we used antibodies to validate the expression level of each aptamer’s reported target on a given cell type. In this way, we could correlate target receptor expression with the observed staining from each aptamer.

**Fig. 3:**
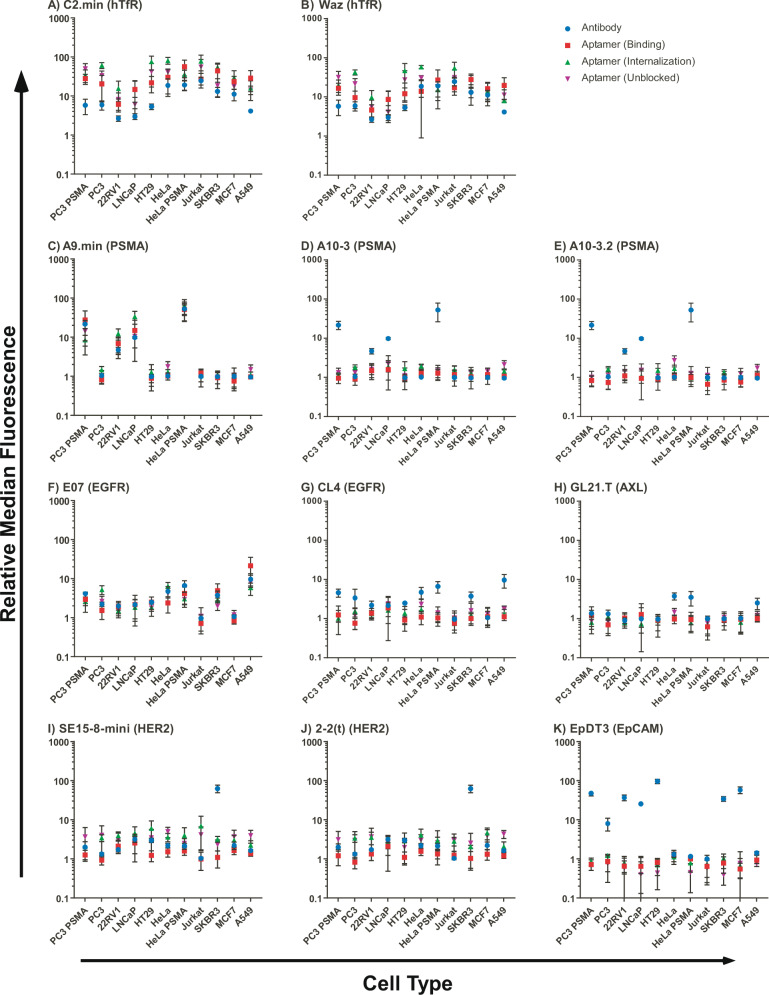
Correlation of aptamer and antibody binding. The median fluorescence staining of all aptamers at 500 nM are shown relative to the median fluorescence of C36 for each cell type. Each aptamer is compared to a commercially available antibody against the aptamer’s reported target using either the binding (red) incubated at room temperature or internalization and binding with blocking (green) protocol incubated at 37 °C or internalization and binding without blocking (purple) incubated at 37 °C. Antibody signals are shown relative to an isotype control on each cell line (blue). Aptamers **A** C2.min and **B** Waz compared to hTfR antibody. Aptamers **C** A9.min, **D** A10-3, and **E** A10-3.2 compared to PSMA antibody. Aptamers **F** E07 and **G** CL4 compared to EGFR antibody. Aptamer **H** GL21.T compared to AXL antibody. Aptamers **I** SE15-8-mini and **J** 2-2(t) compared to HER2 antibody. Aptamer **K** EpDT3 compared to EpCAM antibody. Data represent the mean of three independent experimental replicates for antibodies and four independent experimental replicates for aptamers ±SD. Source data are provided as a Source Data file.

For aptamers with reported targets, we generated a comparative binding plot on each cell type to directly compare the observed level of antibody binding for a given target with the level of staining observed for the corresponding aptamer (Fig. 3). For aptamers with no reported target (XEO2-mini and C1), similar binding plots, without companion antibody staining, can be found in Supplementary Fig. 10. Using this comparative approach, we used a Spearman’s rank-order correlation to identify aptamers that displayed significant correlation with target expression (*ρ* ≥ 0; *p* ≤ 0.05). As shown in Fig. 3 and summarized in Table 3, of the 11 cell specific aptamers tested, only four aptamers, the anti-hTfR aptamers, C2.min and Waz, the anti-PSMA aptamer A9.min, and the anti-EGFR aptamer, E07.min, yielded a positive correlation supporting specific binding. The other seven aptamers, which were reported to bind specific targets, failed to demonstrate significant positive correlation with target receptor expression as determined by antibody staining. In the case of XEO2-mini and C1, the target is unknown. C1, which is reported to be a generalist^24^, demonstrated varied levels of staining across the panel of cells. XEO2-mini, on the other hand, which is reported to bind the prostate cancer cell line PC3^25^, failed to demonstrate any significant signals above background for any of the cell types tested. The results for each aptamer or antibody under each set of staining conditions (internalization and binding with blocking, internalization and binding without blocking, and binding) and against each cell type can be found in the Supplementary Figs. 11–182, which display the cytometry histograms of cells stained at increasing concentrations of aptamer as well as a plot of the relative median fluorescence intensity versus concentration for each aptamer tested on each cell line. Cytometry histograms for experiments performed using antibodies can be found in Supplementary Figs. 183–185.

**Table 3:** Correlative analysis of aptamer and antibody binding.

		Binding		Internalization with blocking		Internalization without blocking	
Target	Aptamer	*ρ*	*P* value	*ρ*	*P* value	*ρ*	*P* value
**hTfR**	**C2**	**0.67**	**0.0277**	**0.72**	**0.0162**	0.60	0.0562
**hTfR**	**Waz**	**0.62**	**0.0478**	**0.65**	**0.0368**	0.58	0.0656
**PSMA**	**A9**	**0.68**	**0.0251**	**0.83**	**0.0027**	**0.79**	**0.0055**
**PSMA**	**A10-3**	0.35	0.2993	−0.02	0.9673	0.30	0.3713
**PSMA**	**A10-3.2**	0.01	0.9895	−0.35	0.2862	0.03	0.9462
**EGFR**	**E07.min**	**0.88**	**0.0007**	**0.85**	**0.0018**	**0.95**	**>0.0001**
**EGFR**	**CL4**	−0.03	0.9462	0.04	0.9241	0.28	0.4023
**HER2**	**SE15-8-mini**	0.35	0.2862	0.02	0.9673	−0.61	0.0519
**HER2**	**2-2**	0.16	0.6337	−0.08	0.8179	−0.47	0.1457
**EpCAM**	**EpDT3**	−0.37	0.2608	−0.33	0.3269	−0.10	0.7759
**AXL**	**GL21.T**	0.05	0.9033	0.40	0.2250	0.39	0.2366

Median fluorescence of each aptamer relative to non-specific control, C36, was compared to the median fluorescence of a commercially available antibody against the same receptor relative to an isotype control using a Spearman Correlation with a two tailed *P* value. This was performed for all aptamers using our three different assay conditions: binding, Internalization, and binding with blocking (blocked), and internalization and binding without blocking (unblocked). Significant correlation was defined for *P* < 0.05 and are represented in bold.

*ρ* Spearman’s correlation coefficient.

### In vitro target validation via siRNA knockdown

**Fig. 4:**
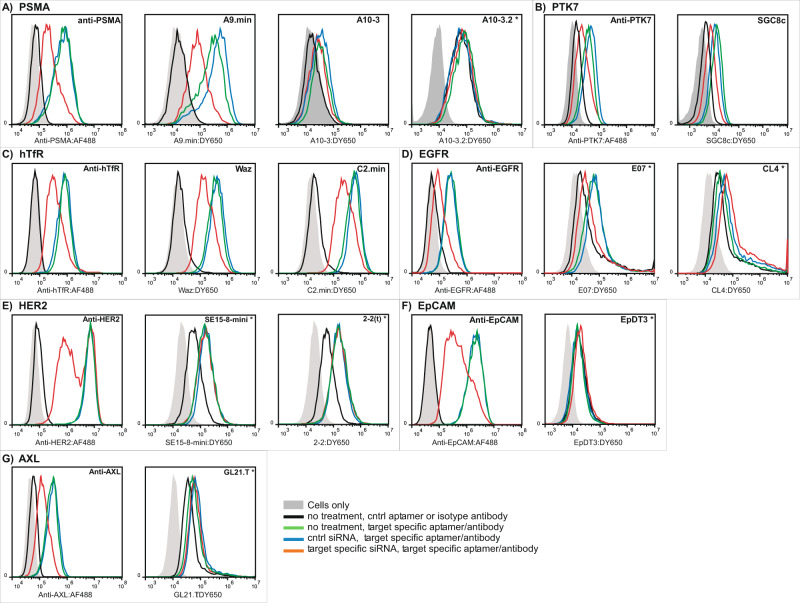
Validation of specificity using target-specific siRNA. Cell lines were transfected with siRNA specific for each aptamer’s reported target (red) or a non-specific siRNA control (blue) and subsequently stained with a target-specific antibody or aptamer. Aptamer or antibody binding to untreated cells are shown in green and non-targeted controls (isotype antibody or the non-targeting aptamer C36) are shown in black. Untreated cells are indicated in gray. All assays were performed at 37 °C using our internalization and binding with blocking protocol except for those denoted with an asterisk. The asterisk denotes experiments performed in the absence of blocking agents (see text for details). The cell type and aptamer concentration used for each experiment were: **A** LNCaP cells: A9.min (100 nM), A10-3 (100 nM), and A10-3.2 (500 nM); **B** A431 cells: SGC8c (100 nM); **C** HeLa cells: Waz (100 nM) and C2.min (100 nM); **D** HeLa cells: E07 (500 nM) and CL4 (1000 nM); **E** SKBR3 cells: SE15-8-mini (1000 nM) and 2-2(t) (1000 nM); **F** MCF7 cells: EpDt3 (1000 nM); and **G** HeLa cells: GL21.T (1000 nM). Data are representative of two independent experimental replicates.

To further assess target specificity, we transfected select cell lines with receptor specific siRNA and validated target knockdown with anti-receptor antibodies and aptamers by flow cytometry. For example, we transfected LNCaP cells with an anti-PSMA siRNA and 72 h later, assessed PSMA expression using an anti-PSMA antibody or the anti-PSMA aptamers A9.min, A10.3 or A10.3-2. As shown in Fig. 4A, target receptor knockdown was clearly observed by staining with the anti-PSMA antibody and the aptamer A9.min. In contrast, no change in signal was observed when staining was performed using the aptamers A10.3 or A10.3-2 (Fig. 4A). The data shown for A9.min and A10.3 were generated using 100 nM aptamer and our internalization and binding protocol with blocking. Similar results were observed when we performed the siRNA knockdown experiments measuring aptamer signal with our binding protocol (Supplementary Fig. 186). In the case of A10.3-2, cell binding in the presence of blocking agents proved exceptionally low (Supplementary Figs. 61 and 178). Therefore, for this molecule, data is shown for assays performed using our internalization without blocking protocol (denoted in Fig. 4A with an asterisk), conditions similar to those used in the literature for aptamer mediated delivery of siRNA^10^, in order to maximize the observed cell staining. Similar experiments were performed for the remaining aptamers reported to bind specific receptors and are shown in Fig. 4B–F and Supplementary Fig. 186. As with A10.3-2, aptamers CL4, SE15-8-mini, 2-2(t), EpDT3 and GL21.T all displayed very low cell staining signals in the presence of blocking agents. Therefore, cell staining with these molecules was performed using our internalization and binding without blocking protocol (Fig. 4D–F). We note that the data shown for E07, an aptamer that displays target cell-specific signal (Fig. 3F and Table 3), is also without blocking agents in order to directly compare to the other anti-EGFR aptamer, CL4. As summarized in Table 4, the results from these knockdown assays confirmed the findings from the comparative analysis experiments (Table 3) with correlated reductions in binding only observed for the anti-hTfR aptamers C2.min and Waz, the anti-PSMA aptamer A9.min, and the anti-EGFR aptamer E07.min. We also used siRNA knockdown experiments to validate the function of the anti-PTK7 aptamer, SGC8c (Fig. 4B and Supplementary Fig. 186) which was not included in our comparative analysis.

**Table 4:** Summary of target validation using siRNA knockdown.

Aptamer	Target	Cell line	Specific knockdown?
C2	TfR	HeLa	Yes
Waz	TfR	HeLa	Yes
A9	PSMA	LnCap	Yes
A10-3	PSMA	LnCap	No
A10-3.2	PSMA	LnCap	No
E07.min	EGFR	HeLa	Yes
CL4	EGFR	HeLa	No
SE15-8-mini	Her2	SkBr3	No
2-2(t)	Her2	SkBr3	No
EpDT3	EpCam	MCF7	No
GL21.T	AXL	HeLa	No
SGC8c	PTK7	A431	Yes

The specificity of aptamers with reported targets was assessed by knocking down the target receptor using target-specific siRNA.

### Analysis of cell targeting aptamers in vivo

Having established a validated list of aptamers that functioned in vitro, we next sought to assess the ability of these molecules to hit their molecular targets in vivo. For this, we utilized aptamers labeled with Alexa Fluor 750 (AF750), near infrared (NIR) imaging, and PC3-PSMA or 22Rv1 flank tumor models. The PC3-PSMA cell line was stability transfected to express PSMA and displays high expression of PSMA and hTfR in culture when measured by flow cytometry (Fig. 3A–E and Supplementary Fig. 182). The parental cell line is well documented in xenograft models^34^. The 22Rv1 cells naturally express PSMA as well as high levels of hTfR (Fig. 3A–E and Supplementary Fig. 182) and readily form tumors^35^. We also performed an immunohistochemical analysis of PSMA and hTfR expression in tumors generated from both cell lines in athymic nude mice to confirm maintained expression of these target receptors (Supplementary Fig. 186).

The use of these tumors, therefore, provided us with a means to test the in vitro validated anti-hTfR aptamers and the anti-PSMA aptamer A9.min. However, since both PC3 cells and 22Rv1 cells are EGFR^low^ and PTK7^low^, we did not perform analysis using the anti-EGFR or SGC8c aptamers in these models. We also chose not to test our aptamer C1 as this molecule has previously been shown to bind and be endocytosed by both mouse and human cells with little preference for cell type^24^.

**Fig. 5:**
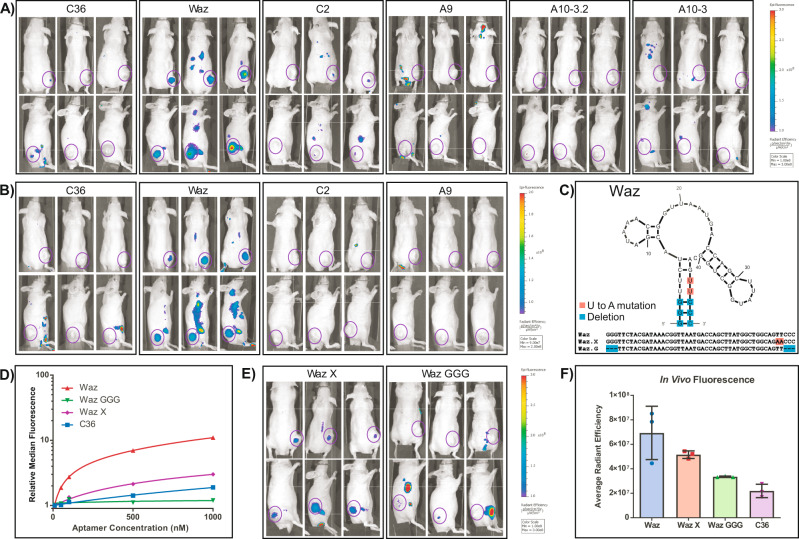
In vivo imaging and validation of aptamer function. Nude mice bearing subcutaneous xenograft tumors on their right flank were injected with 2 nmol of AF750 labeled aptamer (**A**). Dorsal and lateral images of mice bearing 22Rv1 tumors 12 h post injection. The location of the tumor is circled in purple. Injected aptamers are as indicated. Three animals from each cohort (*n* = 6) are shown. Additional animals in the cohorts can be found in Supplementary Fig. 188. A time course for a subset of animals (*n* = 3) used in this experiment can be found in Supplementary Fig. 189. **B** Dorsal and lateral images of mice bearing PC3-PSMA tumors 12 h post injection. The location of the tumor is circled in purple. Injected aptamers are as indicated (*n* = 3). A time course for a subset of animals used in this experiment can be found in Supplementary Fig. 190. **C** Predicted secondary structure of Waz and location of base mutations used to generate mutant variants Waz X and Waz GGG. **D** Relative binding affinity of Waz, Waz.X and Waz.GGG on 22Rv1 cells. **E** Dorsal and lateral images of mice bearing 22Rv1 tumors 12 h post injection. The location of the tumor is circled in purple. **F** Quantitation of fluorescence signal within tumors following injection with Waz, Waz.X, Waz.GGG, or C36 (*t* = 12 h, *n* = 3). Data represents the mean ± SD. Source data are provided as a Source Data file.

We initially assessed tumor localization in 22Rv1 tumors using the in vitro validated Waz, C2.min and A9.min. Additionally, we extended our assay to include both A10.3 and A10.3-2 in the 22Rv1 model. Despite a lack of significant correlation to antibody staining in the in vitro comparative analysis and knockdown assays, these two molecules have previously been reported in the literature to target and deliver a variety of cargos (siRNA^10,12^ and nanoparticles^36^) to this cell type. As shown in Fig. 5A and Supplementary Fig. 188, when viewed 12 h post injection, both dorsally and laterally, only the anti-TfR aptamer Waz demonstrated significant tumor staining. Although some tumor staining was observed for other aptamers (Supplementary Fig. 189; *t* = 3, 6, and 24 h), typically only a small subset 1–2 of animals in a given cohort (*n* = 5–6) displayed signal within the tumors suggesting that the effect was due to variation within the tumor vasculature (i.e., blood pooling, necrosis) as opposed to specific uptake via target binding.

As a follow up, we assessed the in vitro validated Waz, C2.min and A9.min using our PC3-PSMA tumor model. Here again, when viewed at 12 h post injection (Fig. 5B) or other time points (Supplementary Fig. 190) both dorsally and laterally, only the anti-TfR aptamer Waz demonstrated significant tumor staining.

Since aptamer stability in serum may affect its ability to localize to tumors, we measured the stability of each aptamer tested in vivo in fresh mouse serum. All aptamers displayed half-lives close to or greater than 5 h, with Waz displaying the longest half-life at ~11 h and the anti-PSMA aptamer, A9.min, displaying the shortest half-life at ~5 h (Supplementary Table 3 and Supplementary Fig. 191).

Of potentially greater concern is the rapid clearance of aptamers from the plasma following systemic injection. We therefore examined plasma clearance of fluorescently labeled aptamers in C57BL6 mice. Analysis of plasma collected from mice following systemic injection revealed a biphasic clearance profile for all tested aptamers with overall strikingly similar kinetics. Approximately 90% of the labeled aptamer was cleared from plasma within 10 min (*t*_1/2_^α^ = ~2 min) of injection followed by a slower elimination phase (*t*_1/2_^β^ = ~1–2 h; Supplementary Table 3 and Supplementary Fig. 191).

### Validation of in vivo aptamer function

The ability of Waz to function in two different tumor models prompted us to perform additional experiments to validate this molecule’s in vivo function. To this end, we generated two mutants of this aptamer: Waz.X, which includes two U to A mutations to close a small bulge within the aptamer’s stem that has previously been shown important for function, and Waz.GGG, which has a three nucleotide deletion from both the 5′ and 3′ ends and thus eliminates the aptamer terminal closing stem, another feature critical for optimal function (Fig. 5C). We confirmed the loss of activity for these mutants in vitro on 22rV1 cells (Fig. 5D) where Waz.X displayed an apparent Kd of ~1.5 µM, ~8-fold worse than the apparent Kd of Waz on these cells. Waz.GGG behaved similarly to C36 and displayed essentially no activity. We also assessed the serum stability and plasma clearance rates of these molecules (Supplementary Table 3). More importantly, when we assayed these molecules in vivo using the 22Rv1 model, the activity tracked roughly with the observed level of in vitro function, with Waz.X displaying diminished activity and Waz.GGG displaying background levels of staining (Fig. 5E, F and Supplementary Fig. 192).

## Discussion

Aptamers have great potential as therapeutic or diagnostic ligands, and numerous aptamers have been developed to bind to a variety of clinically relevant cell targets. However, assessment of aptamer function has varied quite dramatically between different groups, with methods often not directly assessing the first step in any target-specific downstream events, target binding. Indeed, a previous evaluation revealed potential challenges in using aptamers for cell typing^37^. Here we sought to establish and apply standardized protocols to identify aptamers with the ability to engage their targets on cells both in vitro and in vivo as a means to evaluate their future potential for the targeted delivery of therapeutics.

Due to their innate negative charge, we have observed that aptamers can demonstrate non-specific interactions with proteins or other cellular components, likely those predisposed to bind other polyanions (e.g., heparin sulfate) or scavenger receptors, which are known to bind nucleic acids and are expressed on many cell types^38,39^. Additionally, it is well known that dead or sickly cells can purge or non-specifically uptake macromolecules as their membranes lose integrity^40^. Indeed, when incubated for prolonged times (>6 h) with live cells, we observed significant levels (>5-fold) of non-specific cell staining. This coupled with the fact that different cell types display vastly different levels of non-specific background binding means care needs to be taken to account for these variations when confirming target specificity. For example, the oft-used PSMA (+) LNCaP prostate cancer cells display very high levels of non-specific binding (>10-fold) when compared to the PSMA (−) cell line PC3 cells.

In vitro, we have found that the inclusion of a non-specific competitor, ssDNA, can help to minimize non-specific cell staining and that this effect varies with cell line. Other anionic competitors (e.g., tRNA, dextran sulfate) likely function similarly. However, the presence of competitors is unable to completely block non-specific uptake or compensate for the variable levels of non-specific staining observed across different cell lines. As sites with positive charge potential have previously been reported to be sites of aptamer binding^41–43^, one might expect the presence of poly-anionic competitor to also adversely affect specific binding. However, we have previously observed that the overall electrostatic contributions to the formation of the binding complex can actually be quite small (~18%^44^). Thus, charge–charge interactions may not be as important as one might expect. For the aptamers assessed here that displayed specific binding for their targets (C2.min, Waz, A9.min, SGC8c, and E07), the presence of a non-specific competitor, even at 1 mg/mL, had only minor effects on the aptamer’s cell staining ability (see Fig. 3 and Supplementary Figs. 11–21, 22–32, 33–43, 99–109, and 121–131). In contrast, the presence of the non-specific competitor had a more significant effect on the level of background, non-specific cell labeling, and suppressed cell staining (over 10-fold) on some cell lines (Figs. 1 and 2).

Perhaps more importantly, when we omitted non-specific competitor from our binding correlation studies across multiple cell types (Fig. 3 and Table 3), two of the four aptamers we confirmed as specific for their targets, C2.min and Waz, were no longer statistically significant (*P* > 0.05), a consequence of increased non-specific binding by the non-targeting control sequence, C36. As we know that both C2.min and Waz are, specific for the human transferrin receptor, this serves to further highlight the need for including blocking agents in such analyses, as their absence may not only lead to the identification of false positive, but also false negatives.

Of the compounds we tested, 13 of the 15 molecules have reported Kd values or apparent binding affinities of <200 nM (see Supplementary Table 5). Thus, with the exception of XE0-mini, 2-2(t), and Waz, all experiments were performed under conditions in which the aptamer was in at least a 5 to 10-fold and sometimes >100-fold excess of the reported affinity constant for each aptamer. For the molecules outside this range, XE02-mini has a reported affinity of 1.5 μM by flow cytometry using conditions similar to those we employed here and would be expected to show some signal under the conditions we utilized. 2-2(t) was reported to demonstrate cell binding activity by flow cytometry at 1 μM^19^. The discrepancy observed between our work and both of these reports may rest on the choice of control aptamer used (XE02-mini binding was compared to the naïve aptamer library) and/or the lack of blocking agents (neither study employed a non-specific competitor).

It is possible that the binding affinity for all the compounds that failed to function in our assays are significantly worse on cellular targets than the values reported in their parent publications. However, the fact that we do not see any target-specific binding or cell uptake even at concentrations as high as 1 µM limits the utility of these molecules for the targeted delivery of therapeutics. For comparison, clinically approved antibody drug conjugates (e.g., Trastuzumab Emtansine, Brentuximab Vedotin, Enfortumab Vedotin) and those in clinical trials (e.g., Mirvetuximab soravtansine, Trastuzumab duocarmazine) or their parent antibody, which are being utilized for targeted delivery, typically demonstrate apparent binding affinities on cells under assay conditions similar to those used here in the low to sub nanomolar range (see for example refs. ^45–48^).

In our comparison of aptamer and antibody binding, for a given aptamer/antibody pair, there was some variability across cell types. For example, the signal from Waz on HeLa-PSMA cells using the ‘internalizing and binding with blocking’ protocol was lower than that from the ‘binding’ protocol (Fig. 3B). Similarly, the signal for E07 assayed with ‘internalizing and binding with blocking’ was lower than that observed for the ‘binding’ protocol (Fig. 3F). In some cases, these discrepancies may have arisen from differences in the background uptake levels, as our non-targeting control sequence, C36, was used to normalize the data. However, in other cases, differences in binding may have been due to target/epitope variations (e.g., variation in glycosylation, the presence of splice variants, etc.) on different cell types. Thus, as an additional check to validate aptamer specificity, we used siRNA to knock down the target receptor. Importantly, the results from our analysis using siRNA identified the same ‘hits’ as our correlative study across cell types.

There were a number of discrepancies between our in vitro work and those previously reported in the aptamer literature. In an attempt to reconcile some of these differences between our work and the literature, we also performed a number of additional experiments, which can be found in Supplementary Figs. 193–201. For example, in our studies the often used anti-PSMA aptamer A10.3 and its minimized variant A10.3.2, failed to demonstrate any significant correlation to anti-PSMA antibody binding to PSMA positive cell lines. Both aptamers showed a slight increases in binding on the PSMA positive LNCaP cells, with A10.3 performing better than A10.3.2 (see also Supplementary Figs. 50 and 61); however, using siRNA to knock down PSMA expression, we were unable to correlate aptamer binding with protein knockdown (Fig. 4 and Table 4). These aptamers also both failed to show any appreciable binding to 22Rv1 cells, another prostate cell line that naturally expresses PSMA, or either of the two engineered PSMA-expressing cell lines we tested (HeLa-PSMA and PC3-PSMA, Fig. 3D, E; also see Supplementary Figs. 44, 46, 52, 55, 57, and 63). As these molecules are two of the most widely used aptamers and reported to bind the human protein when expressed natively on human cells (22Rv1 and LnCAP^10–12^), ectopically on mouse cell lines (B16-PSMA, CT26-PSMA^49,50^), and even bind to canine cells expressing the canine PSMA (canine hemangiosarcoma cell lines^51^), we performed experiments on recombinant PSMA protein to reconcile these results. Interestingly, while A10.3 and A10.3.2 both fail to bind PSMA produced from mammalian cells, the molecules showed modest binding for protein produced from insect cells (Supplementary Fig. 193). As protein produced in insect cells was the target for the original selection^11^, the lack of activity on human protein may be due to differences in glycosylation. Similar selectivity in binding based on glycosylation has been shown in a selection against the EGFR receptor^52^. For this reason, it is important to characterize aptamer-target binding in a cellular context to determine specificity.

The aptamers GL21.T^18^, CL4^15^, and EpDT3^21^ demonstrated the lowest levels of correlation with target receptor expression (Table 3). They also failed to yield any target-specific cell labeling in our siRNA knockdown experiments, even when performed under conditions that favor staining (no blocking agents). In additional supplementary experiments using flow cytometry, the anti-AXL aptamer, GL21.T, also failed to stain HEK293T cells overexpressing AXL using all three of our assay formats (Supplementary Fig. 194). Similarly, in contrast to the anti-EGFR aptamer E07.min, the reported anti-EGFR aptamer CL4 failed to stain A431 cells, an EGFR^high^ cell line often employed for studies involving this receptor (Supplementary Fig. 195). In the case of the reported anti-EpCAM aptamer EpDT3, we performed additional experiments to assess prolonged, 24 h, incubation time on cell staining using HT29 cells, as a recent report suggested EpCAM endocytosis and subsequent cell staining by this molecule to be slow^53^. However, here again, the aptamer failed to display any signal above that observed for a non-specific control (Supplementary Fig. 196).

Similarly, both of the reported anti-HER2 aptamers we tested, SE-15-8-mini^20^ and 2-2(t)^19^, failed to show any significant binding to SkBR3 cells, the only HER2^high^ cell line we tested. Interestingly, both of these aptamers showed increased staining when assessed for internalization and binding on Jurkat cells, the only outright HER2^Negative^ cell line we tested. Together, these results suggest that neither HER2 aptamer is specific for the reported target, a conclusion supported by our siRNA data (Fig. 4E and Supplementary Fig. 186).

Again, it is important to note the standardized in vitro binding conditions we have utilized to test some of these molecules are somewhat different than conditions originally reported for each aptamer selection. However, molecules best suited for use as detection agents or for the development of therapeutics, arguably, need to be robust and maintain function in physiologically relevant biological buffers (e.g., DPBS, media, HBSS, etc.). Since folding conditions and buffers do vary between studies, there is the potential for aptamers to require specific conditions (e.g., temperature, time, divalent cations) to adopt a functional form. Therefore, to further examine these effects, we tested the aptamers that did not demonstrate specific signal in our assays for target binding when folded under previously reported conditions. However, when compared to the binding of aptamers folded under literature conditions with those folded under our standardized conditions, no changes in fluorescence signal were observed (Supplementary Fig. 199).

For our in vivo studies, we used IVIS imaging to look for aptamer localization to tumors following intravenous (tail vein) injection. Tumors were grown to ~0.5–1.0 cm in diameter and size matched for comparison to controls to minimize the potential for artifacts that may result from variation in vascularization^54,55^. Our choices of tumor models for these experiments, PSMA-expressing 22Rv1 cells or PC3-PSMA cells, express high levels of hTfR and PSMA (>10-fold staining as assessed by aptamer staining using flow cytometry), but low or no levels of EGFR and PTK7. As such, we limited our in vivo analyses to the in vitro validated anti-hTfR aptamers C2.min and Waz and the anti-PSMA aptamer A9. We also extended this analysis to the other two anti-PSMA aptamers, A10-3 and A10-3.2, given their prevalence in the aptamer literature. We note that while we didn’t assess the in vivo function of E07 or SGC8c, their ability to localize and stain tumors in vivo has previously been reported^56,57^.

In vivo systems are significantly more complex than assays in culture. It is thus, perhaps, not surprising that a strong specific signal in cell culture does not guarantee successful translation to in vivo activity. It is also important to note that while composed of 2′F RNA, none of the molecules we tested are fully stabilized molecules and degrade in serum; all of the aptamers displayed half-life in serum of between ~5 to 11+ hours (Supplementary Table 3). Based on their small size (~15 kDa), aptamer clearance is fast. When fit using a two-compartment model, the half-life for the first phase of clearance for all aptamers tested was ~2 min, followed by a longer, ~2 h, phase. Thus, for all of these molecules tested, plasma clearance, not stability, appears to be the limiting factor in vivo. This fact allowed us to directly compare in vivo activities of the aptamers based on monitoring the fluorescence signal, eschewing the more traditional dual-probe hybridization approach often used to confirm the presence of full length functional aptamers^4^.

In vivo, Waz displayed a specific signal above a non-specific control (C36) in both tumor models, a fact we confirmed by performing additional analyses with point mutants of this aptamer, which demonstrated diminished (Waz.X) or abolished function (Waz.GGG). The in vitro activity of these molecules correlated well with their in vivo function. Surprisingly, C2.min and A9.min did not demonstrate any activity over that observed for our non-targeting control, C36. Similarly, but consistent with our in vitro results, neither A10.3 nor A10.3-2 demonstrated significant tumor staining. Thus, unlike the other molecules tested, Waz is capable of engaging its target in vivo. However, while tumor localization is an encouraging proof of concept, since Waz does not cross react with the murine transferrin receptor, additional studies are required to better understand its targeting capability in more realistic systems.

However, it is important to note that even for Waz, the signal in the tumor above background in our images at 12 h was only ~2–3-fold as assessed by NIR imaging. These data, in fact, are similar in magnitude to the signals we observed using another aptamer on which we have recently reported, E3^58^. Together, these data demonstrate that 2′F modified RNA aptamers can target tumors even without further stabilization or PEGylation. Alterations to slow clearance rates (e.g., PEGylation) are likely to improve on these results but will require additional aptamer stabilization. Such alterations might also allow molecules such as C2.min and A9.min, which are specific for their targets in vitro, a greater opportunity to bind when utilized in vivo.

Interestingly, despite the fact that both C2.min and Waz bind the same target with similar affinities (C2 actually binds ~4-fold more tightly) and that the molecules share similar serum stabilities and clearance rates (Supplementary Table 3), only Waz functioned in vivo. There is likely more at play when moving from in vitro to in vivo. Indeed, while we have previously reported that C2.min showed no activity on mouse cell lines, in more recent analyses using DY650 labeled aptamers, which provides for increased signal over the AF488 labeled molecules used in our earlier studies, we have observed some cross-reactivity with murine transferrin receptor (Supplementary Fig. 197). Additionally, C2.min competes with both human transferrin^13^ and, to a lesser extent, murine transferrin (Supplementary Fig. 198), which could serve to suppress tumor targeting in vivo.

In the case of the anti-PSMA aptamer, A9.min, Dassie et al. reported on the ability of an anti-PSMA aptamer, A9g, to localize to PC3 PSMA + cells following systemic injection^59^. A9g is, in fact, the same aptamer sequence as A9.min, but linked to the fluorescent dye CW800 via an amide linkage. In light of this difference, we performed additional studies using an amide linked AF750 in an attempt to reconcile the difference in our results. These studies too, failed to demonstrate tumor staining (Supplementary Fig. 200). Thus, the observed discrepancy between that work and our own may reflect differences in the cell lines used, with the line employed by Dassie et al. potentially displaying more favorable target expression levels, or that the resulting tumors displayed more favorable targeting characteristics (e.g., large tumor size and/or increased leaky vasculature). Alternately, the observed differences could be a result of the choice of fluorescent dye (AF750 versus CW800).

To the extent that these in vivo studies may be affected by our choice of fluorescent dye, it is also important to note that in all of our studies, we could not rule out that the identity of the dye molecule (DY650, AF750) or its placement at the 5′ end of the aptamer via a 5′ thioester linkage did not adversely affect aptamer function. Therefore, for many of the molecules that failed to function, experiments were also performed with an alternate dye, AF488, to rule out this possibility. For example, anti-PSMA aptamers that failed to function bearing DY650 also failed to function with AF488 (Supplementary Fig. 201).

In summary, we have described standardized conditions to assess aptamer suitability for cell surface targeting, since an aptamer’s reported affinity for a target or its reported activity does not necessarily indicate how robustly it can function on cells or in vivo. We applied our assay conditions to the analysis and characterization of 15 cell surface targeting aptamers reported from the literature and identified the anti-transferrin receptor aptamer, Waz, as a robust candidate for future in vivo studies. The majority of other aptamers we tested failed to function effectively as cell targeting agents.

Through our investigation, we have found that a number of different factors can skew apparent aptamer function, such as variable levels of non-specific uptake between different cells. However, the use of appropriate non-targeting controls, inclusion of blocking agents, and validation of target engagement through knock down or knock in experiments would eliminate those non-specific and somewhat misleading interactions. Going forward, researchers can apply these assay conditions to determine an aptamer’s effectiveness before wasting time on more costly functional experiments. We do caution, however, that aptamer function in vitro may not necessarily translate to aptamer function in vivo.

Taken as a whole, our work should provide a framework for future studies in the development and validation of aptamers to cell surface targets. Aptamers that demonstrate high target specificity and efficient cell staining are likely to provide greater success in delivering a diagnostic or therapeutic payload while avoiding off target effects. As such, we expect that more careful and detailed aptamer characterization and target validation will lead to the identification of more robust aptamers in the future. This, in turn, should translate to the development of more effective aptamer-based diagnostics and therapeutics.

## Methods

### Cells and cell culture

All parental cell lines were obtained through ATCC. Cells were maintained according to ATCC guidelines. Jurkat (cat# TIB-152), LNCaP (cat# CRL-1740), 22RV1 (cat# CRL-2505), and SKBR3 (cat# HTB-30) cells were grown and assayed in RPMI supplemented with 10% FBS. HeLa (cat# CCL-2), HeLa-PSMA (HeLa cells stably transfected to express PSMA^24^), MCF7 (cat# HTB-22), A431 (cat# CRL-1555), and HT29 (cat# HTB-38) cells were maintained in DMEM supplemented with 10%FBS. PC3 (cat# RL-1435), PC3-PSMA (PC3 cells stably transfected to express PSMA^60^), and A549 (cat# CCL-185) cells were grown and assayed in F12K media supplemented with 10% FBS. Media for cell growth was purchased through Corning Life Sciences (Manassas, VA). Unless otherwise stated, FBS and all other cell culture buffers and solutions were obtained from Thermo Fisher Scientific (Grand Island, NY). All cell lines were routinely checked for mycoplasma contamination using the MycoAlert mycoplasma detection kit (Lonza, Basel, Switzerland). No contamination was observed during any point of our studies. All cells were grown at 37 °C with 5% CO_2_ and 95% relative humidity.

### Mice and husbandry

Male Nu/Nu nude mice were purchased from Charles River (Kingston, NY) at 4 weeks of age for tumor studies. Tumors were allowed to grow to a maximum size of 2 cm in diameter and was not exceeded. C57BL/6 mice were originally purchased from Taconic (Hudson, NY) and bred in house. Males and females between 8 and 14 weeks were used in serum stability studies and plasma clearance studies. Mice were housed, five animals per cage, in a temperature and humidity-controlled SPF facility with a 12-h light: dark cycle. All experiments were performed in accordance with Institutional Animal Care and Use Committee regulations and protocols were approved by the Einstein Institutional Animal Care and Use Committee.

### Aptamer synthesis and preparation

Fifteen aptamers (Table 1) were synthesized in house by solid phase synthesis with a 5′ disulfide modification, which was used for subsequent conjugation to fluorophores. The 5′ DMT was left on following synthesis to facilitate purification by reversed phase HPLC. The identity of each aptamer was confirmed by mass spectrometry (after reduction with TCEP) with the determined masses closely matching the predicted values (Supplementary Table 1). For in vitro experiments, each aptamer was labeled with DyLight 650 maleimide (DY650). For in vivo experiments, aptamers were conjugated to Alexa Fluor 750 maleimide (AF750). Dye conjugation reactions typically proceeded to ~100%, as determined by HPLC. Free label was removed by desalting (Bio-Spin 6; BioRad, Freemont CA) and confirmed by HPLC. Labeled aptamers were further characterized by denaturing gel electrophoresis (Supplementary Fig. 1) where they ran as a single band. Detailed synthesis and aptamer labeling methods can be found online (see Supplementary Methods)

### Aptamer internalization and binding assays

Adherent cells (~2 × 10^4^) were plated in 96-well cell culture plates 18–24 h prior to each assay to achieve ~90% confluency at the time of the assay. LNCaP cells were plated at 2 × 10^4^ cells per well 48 h in advance. One hour before the assay, media was replaced with fresh media with or without 1 mg/mL salmon sperm DNA (ssDNA, resuspended in dH2O at 10 mg/mL; Millipore, Burlington, MA) and allowed to incubate at 37 °C. For assays using Jurkat cells, cells were plated in 96-well round bottom plates at 5 × 10^4^ cells per well in fresh growth media with or without 1 mg/mL ssDNA and incubated for one hour at 37 °C. Aptamers were diluted in DPBS (without Mg and Ca) to a volume of 10 μL, denatured for 3 min at 70 °C, and allowed to refold at room temperature for at least 15 min prior to addition to cells. Refolded aptamers were added to the plated cells in full growth media with or without ssDNA (final volume of 100 μL) and incubated at 37 °C for 1 h unless otherwise noted. Adherent cells were washed twice with 50 μL DPBS and then lifted from the plate with a 5–10-min incubation in 50 μL 0.05% trypsin with 0.53 mM EDTA at 37 °C. The trypsin was inactivated with FACS buffer (HBSS containing 1.3 mM Ca^2+^ and 0.9 mM Mg^2+^, supplemented with 1% BSA, and 0.1% sodium azide), and cells were transferred to a 96-well round bottom plate. Plates were spun at 300 × *g* for 5 min to pellet cells. Cells were then resuspended in FACS buffer with 1 ng/mL bisbenzimide (FACS-bisbenz) and analyzed on an iCyt Eclipse flow cytometer (Sony Biotechnology, San Jose, CA). Jurkat cells were washed three times with FACS buffer and resuspended after the final wash in FACS-bisbenz.

### Antibody and aptamer binding assays

Antibody and aptamer binding assays were performed in 96 well round bottom plates on 5 × 10^4^ cells lifted with trypsin, as described above, and resuspended in 90 μL FACS buffer (HBSS containing 1.3 mM Ca^2+^, 0.9 mM Mg^2+^, 1% BSA, and 0.1% sodium azide) supplemented with 1 mg/mL ssDNA. Jurkat cells were counted and resuspended in FACS buffer without trypsin treatment. Aptamers were refolded in DPBS (without Mg and Ca) as described above and subsequently added to wells containing cells in 90 μL FACS buffer for a final assay volume of 100 μL. Cells and aptamers were incubated at room temperature for 30 min and then washed three times with FACS buffer. Cells were resuspended in FACS-bisbenz and analyzed on an iCyt Eclipse flow cytometer. All antibodies were titrated on cell lines previously reported to express their respective targets. All antibodies demonstrated activity at the vendor recommended dilutions. Subsequent studies were therefore performed at the vendor recommended concentrations. A full list of antibodies used is provided in the Supplementary Methods.

### siRNA Knockdown of targets

Knockdown of targets by siRNA was performed using HiPerFect transfection reagent (Qiagen, Valencia, CA) and a cell line with high expression of the chosen target. Cells expressing each aptamer’s reported target were plated at 2 × 10^4^ per well in a 96-well plate in 150 μL of the cells’ corresponding growth media. Master mixes of HiPerFect and siRNA were prepared in Opti-MEM serum free media (Thermo Fisher), allowed to sit at room temperature for 10 min, and 50 μL was then added to each well. Each siRNA reaction was first optimized for siRNA concentration and incubation time using the antibody control. Once detectable knockdown was observed by flow cytometry, each aptamer was tested in both internalization and antibody-like binding conditions. Each assay included cells treated with a target-specific siRNA, a control siRNA against EGFP, and untreated cells. Specific siRNA concentration and incubation times are listed in Supplementary Table 2.

### In vivo NIR imaging

Mice were injected subcutaneously with 2 × 10^6^ to 5 × 10^6^ cells 3–6 weeks prior to imaging. Tumor growth was monitored, and animals were used for imaging when the tumor was at least 0.5 cm but <1.5 cm in diameter. Tumor bearing mice were injected with 2 nmoles of AF750 labeled aptamer in PBS via tail vein injection. The mice were imaged on an IVIS Spectrum Imager 3, 6, 12, and 24 h post injection. Tumors were sized matched prior to the experiment for comparison to control sequences. Animal numbers used for each experiment are indicated in each figure legend. Images shown are representative of all animals treated. Images of all treated animals in each cohort can be found in the Supplementary Materials. For these studies, animals were not blinded or randomized.

### Statistical analysis

All graphs were plotted, and statistical analyses were performed using GraphPad Prism. Graphs represent the mean ± standard deviation. For correlative studies, the median fluorescence of each aptamer is shown relative to the median fluorescence of control aptamer C36. Antibodies are shown relative to a corresponding isotype control. Aptamers were compared to antibody controls using Spearman’s rank-order correlation, *ρ* = Spearman’s correlation coefficient. Significant correlation was defined for *P* < 0.05.

### Reporting summary

Further information on research design is available in the Nature Research Reporting Summary linked to this article.

## Supplementary information

Supplementary Information

Reporting Summary

## Data Availability

The source data for all graphs are provided in the attached Source Data.xlsx file. Raw data files are available from the corresponding author on reasonable request. Source data are provided with this paper.

## Source data

Source Data
