# Supplementary material for: A comparative analysis of cell surface targeting aptamers
Source: Nat Commun. 2021 Nov 1;12:6275. doi: 10.1038/s41467-021-26463-w (PMC8560833; doi:10.1038/s41467-021-26463-w)
Supplement: Supplementary file 1 — Supplementary Information [file 41467_2021_26463_MOESM1_ESM.pdf]

Supplementary Methods

Aptamer synthesis and conjugation. All chemical synthesis was performed using standard solid phase nucleic acid synthesis chemistry on an Expedite 8909 DNA synthesizer (Biolytic, Fremont, CA). Phosphoramidite monomers were purchased from ChemGenes (Wilmington, MA). Inverted 3'-3' dT columns (dT-5'-SynBase CPG) were purchased from Link Technologies (Bellshill, United Kingdom). All other synthesis reagents were purchased form Glen Research (Sterling, VA). Aptamers were generated with a 3' inverted dT residue for added serum stability and a 5' Thiol-Modifier C6 S-S for later chemical conjugations. All aptamers were synthesized with the final dimethoxytrityl group left on to facilitate purification. Following deprotection, using standard procedures, aptamers were purified by reversed-phase HLPC on a 10 × 50 mm Xbridge C18 column (Waters, Milford, MA) using a linear gradient of acetonitrile in 0.1 M triethylammonium acetate at pH 7.5 at 65°C.

Aptamers were analyzed by mass spectrometry (MS) using a Waters Acquity Ultra Performance Liquid Chromatography (UPLC) system coupled to a Waters Synapt G2 quadrupole-time of flight hybrid mass spectrometer (MS). For analysis, reduced aptamers were resuspended at 200 µM in TEAA. Samples, 5 uL, were desalted using a linear gradient of MeOH in 400 mM HFIP/8 mM TEA using a Waters Xbridge C18 2.1X50 mm column.

Dye conjugation was performed in a manner similar to that previously described (1). In short, individual aptamers were suspended in 0.1 M TEAA with 10 mM TCEP and heated to 70°C for 3 minutes. Reduction was confirmed by analytical HPLC. Reduced aptamers were desalted into PBS containing 2 mM EDTA using a Bio-Spin 6 desalting column (Bio-Rad). Maleimide activated dyes, DyLight 650 (Pierce, Rockford Il) for *in vitro* experiments or Alexa Fluor 750 (Invitrogen, Carlsbad CA) for *in vivo* experiments, were dissolved in DMSO and added to the reduced aptamers at a fivefold molar excess of dye. Dye reactions were monitored by analytical HPLC and were desalted into PBS with Bio-Spin 6 columns to remove excess dye. Conjugation reactions routinely proceeded to completion. The removal of free dye was confirmed by analytical HPLC.

Fluorescently labeled antibodies and controls. The Alexa Fluor 488 labeled anti-EGFR antibody (Cat #2364535), Anti-EpCAM antibody (Cat #2221045), Anti-PSMA antibody (Cat #2312525), Anti-HER2 antibody (Cat #2222050), Isotype control (Cat #2600660 and #2601645) were purchased from Sony Biotechnology (San Jose, CA). The Alexa Fluor 488 labeled anti-AXL antibody (Cat #FAB154G) was purchased from R&D systems (Minneapolis, MN). Unlabeled anti-hTfR antibody was purchased from BD systems (Cat # 555534; San Jose CA) and anti-PTK7 from Miltenyi Biotec (Cat #130-091-578; Bergisch Gladbach, Germany). Unlabeled antibodies were labeled using the amine reactive Alexa Fluor 488-SDP ester (Cat # A20000; Life Technologies, Carlsbad, CA) and subsequently desalted following standard protocols.

Serum stability assays and pharmacokinetics. For serum stability assays, whole blood from C57BL6 mice was collected via terminal cardiac stick and allowed to clot for 20 minutes at room temperature. Whole blood was centrifuged with a fixed angle rotor at 2000 x g for 15 minutes at 4°C to separate and collect the serum. Labeled AF750-labeled aptamers were prepared as 30 µM stocks in 10 µL PBS, heated to 70°C and allowed to incubate on the benchtop for 15 minutes prior to use. Samples were then added to 90 µL of serum for a final concentration of 3 µM in 100 µL of 90% serum. At each time point, 10 µL of reaction was removed to a 0.5 mL tube, and the RNA was recovered by a methanol chloroform extraction as previously described (2). In short, samples diluted with 50 µL of PBS, 100 µL methanol, and 50 µL chloroform and mixed after which an additional 50 µL of water and 50 µL of chloroform were added, and the tube was vortexed again. The samples were then spun at 500 x g for 20 minutes at 4°C in a fixed angle rotor. The aqueous layer (containing the RNA) was collected and stored at -20°C until completion of all time points. Samples were diluted 1:1 in RNA loading buffer (95% formamide, 10 mM EDTA, 0.05% bromophenol blue), and 4 µL was loaded onto an 8%, 19:1 (acrylamide:bis-acrylamide), 7 M urea gel. Gels were scanned on a LI-COR Odyssey scanner to visualize full length labeled aptamer. Full length aptamer was quantified in the LI-COR Imaging Suite Lite and normalized to the hour zero recovered RNA. Rates of decay and serum half-lives were determined using GraphPad Prism.

To determine the clearance of fluorescently labeled aptamers *in vivo*, 10-12 week old male C57BL6 mice were injected with 2 nmoles of AF750-labeled aptamer. Whole blood was collected in the presence of EDTA either by tail nick at 5, 15 or 20 minutes and terminal cardiac puncture at 1, 4 or 8 hours. Plasma was recovered by centrifuging for 15 minutes at 2000 x g at 4°C. Fluorescence was measured using a Synergy H4 Hybrid Multi-Mode Microplate Reader (BioTek, Winooski, VT). Samples were excited at 750 nm and emission was measured at 775 nm. Primary and secondary pharmacokinetic parameters were determined from analysis of the concentration vs. time data in the context of a biphasic, two compartment model.

Immunohistochemistry. A subset of tumors was submitted to the Einstein Histopathology Core facility to assess receptor expression. Briefly, tumors were excised and immersed in 10% neutral buffered formalin at 4°C. Tumors were then transferred to 70% ethanol and embedded in paraffin for sectioning. Tumors were treated with either anti-PSMA (LS-Bio; Seatle, WA) or anti-CD71 primary antibodies (eBioscience; San Diego. CA). After treatment with an HRP conjugated secondary antibody, the sections were stained with 3,3'-diaminobenzidine (DAB).

PSMA binding bead assay Recombinant extracellular domain of PSMA labeled with a N terminal 6x His tag (pAcGP67A; Addgene) was produced in insect cells (Sf9) in a manner similar to that previously described (1). Recombinant PSMA produced in mammalian CHO cells was purchased from R&D systems (Minneapolis MN). Both forms of the protein were immobilized on Dynal, 1 µm His-Tag Isolation magnetic beads at ~1 µg protein per 2.5 µL of beads. The beads were washed with PBS plus Mg^{2+} and Ca^{2+} with 0.2% Tween-20 to remove excess protein. Protein loaded beads were stained with increasing concentrations of the reported PSMA specific aptamers, A9.min, A10-3, A10-3.2 or a non-targeted control, C36 labeled with DyLight 650 for 30 minutes at room temperature. Beads were washed three times with PBS plus Mg^{2+} and Ca^{2+} and read by flow cytometry on a a Sony SA3800 flow cytometer (Sony Biotechnology, San Jose, CA).

Transferrin competition assays. Mouse apo-transferrin (mTf; Sigma; St. Louis, MO) at 50 mg/ml in PBS was mixed 1:1 with 1 mg/ml ferric ammonium citrate in 10 mM $NaHCO_3$ / 20 mM HEPES pH 7.7 and incubated at 37°C for 10 minutes and then desalted into PBS with a Biospin 6 desalting column (Biorad; Hercules, CA) to produce iron loaded holo-mTf. Jurkat cells prepared with 1 mg/ml ssDNA were pre-blocked with titrations of holo-mTf, followed by addition of thermally refolded AF488-labeled C2.min to 100 nM. Cells were incubated for 1 hour at 37°C, washed, and analyzed by flow cytometry.

Binding buffer and refolding comparison assays. For each test, we utilized a cell line with high expression of the aptamer's reported target, as described in the main text. Aptamers were resuspended at 5 µM in buffers reported in each selection publication and refolded according to the published protocol. The buffers and refolding conditions are summarized in **Table S4**. In parallel, each aptamer was also refolded using our standard conditions, in DPBS (without Mg^{2+} or Ca^{2+}) by heating to 70°C for 3 minutes and then allowing the sample to sit at room temperature for 15 minutes prior to use. Cell binding and uptake was determined using our "Aptamer internalization and binding assay." In short, 10 µL of refolded aptamer was added to plated cell lines in 90 µl of the cell line's growth media for a final volume of 100 µl and concentration of 500 nM. Cells were incubated at 37°C for 1 hour and then washed twice with 50 µL DPBS and then lifted from the plate by brief trypsinization. The trypsin was inactivated with FACS buffer (HBSS containing 1.3 mM Ca2+ and 0.9 mM Mg^{2+}, supplemented with 1% BSA and 0.1% sodium azide), and cells were transferred to a 96 well round bottom plate. Plates were spun at 300 x g for 5 minutes to pellet cells. Cells were then resuspended in FACS buffer with 1 ng/mL bisbenzimide (FACS-bisbenz) and analyzed on a Sony SA3800 flow cytometer (Sony Biotechnology, San Jose, CA).

Media Stability Assay. Aptamers labeled with DyLight 650 were diluted in DMEM with 10% FBS to a concentration of 1 µM. Diluted aptamers were then incubated at 37°C for 1 or 24 hours. Post incubation samples were diluted 10-fold in a 7M urea loading dye containing 1% SDS and heat denatured by heating at 65°C for 5 minutes. Analysis was performed by denaturing (7M urea) gel electrophoresis on a 12% polyacrylamide gel which had been pre-run with heated TBE containing 0.1% SDS. Gels were analyzed on a Bio-Rad ChemiDocXRS+ imaging system. Band signal intensity corresponding to the full length aptamer was

quantified using Image Lab 6.0 and normalized to samples in media diluted and heat inactivated in 7M urea dye without 37°C incubation.

Supplementary Table 1. Aptamer Sequences, Predicted Mass and Determined Mass

	Aptamer	Target	Sequence	Predicted MW	MS Determined MW
2'F-Py, 2'OH-Pu	A9min	PSMA	5'-GGGACCGAAAAAGACCUGACUUCUAUACUAAGUCUACGUUCCC-3'	14247.4	14248.0
	A10-3	PSMA	5'-GGGAGGACGAUGCGGAUCAGCCAUGUUUACGUCACUCCUUGUCAAUCCUCAUCGGC-3'	18466.8	18466.9
	A10-3.2	PSMA	5'-GGGAGGACGAUGCGGAUCAGCCAUGUUUACGUCACUCCU-3'	13059.7	13058.9
	WAZ	hTfR	5'-GGGUUCUACGAUAAACGGUUAAUGAUCAGCUUAUGGCUGGCAGUUCCC-3'	15933.3	15931.8
	C2	hTfR	5'-GGGGGAUCAAUCCAAGGGACCCGGAAACGCUCCCUUACACCCC-3'	14312.5	14311.2
	E07.min	EGFR	5'-GGACGGAUUUAAUCGCCGUAGAAAGCAUGUCAAAGCCGGAACCGUCC-3'	15686.3	15685.1
	CL4	EGFR	5'-GCCUUAGUAACGUGCUUUGAUGUCGAUUCGACAGGAGGC-3'	13061.7	13061.2
	GL21.T	AXL	5'-AUGAUCAAUCGCCUCAAUUCGACAGGAGGCUCAC-3'	11375.8	11373.3
	EpDT3	EpCAM	5'-GCGACUGGUUACCCGGUCG-3'	6588.9	6588.9
	SE15-8-mini	HER2	5'-AGCCGCGAGGGGAGGGAUAGGGUAGGGCGCGGCU-3'	11740.1	11739.5
	C1	Cells	5'-UGCGAAUCCUCUAUCCGUUCUAAACGCUUUAUGAUUUCGCA-3'	13473.8	13472.6
	C36	Control	5'-GGCGUAGUGAUUAUGAAUCGUGUGCUAAUACACGCC-3'	12085.1	12086.1
2'OMe H, 2'OH G	XEO2-mini	Cells	5'-CACGACGCUGAUGGAUCGUUACGACUAGCAUCGC-3'	11724.6	11724.5
DNA	AS1411	Nucleolin	5'-TTGGTGGTGGTGGTTGTGGTGGTGGTGG-3'	9076.9	9076.6
	Sgc8c	PTK7	5′-ATCTAACTGCTGCGCCGCCGGGAAAATACTGTACGGTTAGA-3′	12830.4	12829.5
	2-2(t)	HER2	5'-GCAGCGGTGTGGGGGCAGCGGTGTGGGGGCAGCGGTGTGGGG-3'	13522.8	13521.9

Supplementary Table 2. Target specific conditions of siRNA knockdown assays

Target	Cells	siRNA Concentration (nM in well)	Hiperfect Reagent (μL/well)	Incubation Time (hours)
hTfR	HeLas	1	0.75	72
EGFR	HeLas	1	0.75	72
AXL	HeLas	1	0.75	48
HER2	SKBR3s	7.5	0.75	72
EpCam	MCF7s	1	0.75	72
PSMA	LnCaps	5	1.5	72
PTK7	A431s	5	0.75	72

Supplementary Table 3. Serum Stability and *In Vivo* Plasma Clearance

Aptamer	Rate of Decay	Half-Life in Serum (Hrs)	$t_{1/2}^{\alpha}$ Plasma (mins)	$t_{1/2}^{\beta}$ Plasma (mins)
C2	-7.8E-02	8.9	1.9	92
WAZ	-6.0E-02	11.5	2.5	94
WAZ X	-3.7E-02	18.5	*	*
WAZ GGG	-1.6E-01	4.4	1.6	97
E07	-8.8E-02	7.8	*	*
A10-3.2	-1.5E-01	4.7	2.3	98
A9	-1.4E-01	5.1	1.4	91
C36	-1.5E-01	4.7	2.0	93
A10-3	-1.2E-01	5.9	2.1	88

* denotes aptamers not tested.

Supplementary Table 4. Literature refolding conditions for aptamers tested in Figure S198

Aptamer	Folding Buffer	Refolding	Cell Line	Previous Assay Conditions	Reference
A9	$dPBS^{+/+}$	65°C for 5 minutes then 37°C for 10 minutes	HeLa PSMA	Internal control to assess the effects of divalents on this molecule's function	
A10-3	$dPBS^{+/+}$	65°C for 5 minutes then 37°C for 10 minutes	HeLa PSMA	Binding assays performed in HEPES buffer on fixed cells. Gene silencing assays preformed in growth media.	McNamara, J., Andrechek, E., Wang, Y. et al. Cell type–specific delivery of siRNAs with aptamer-siRNA chimeras. Nat Biotechnol 24, 1005–1015 (2006).
A10-3.2	$dPBS^{+/+}$	65°C for 5 minutes then 37°C for 10 minutes	HeLa PSMA	^{32}P Binding assays performed in $dPBS^{+/+}$ with 100μg/ml tRNA and polyI:C. Silencing assays performed in growth media.	Dassie, J., Liu, X., Thomas, G. et al. Systemic administration of optimized aptamer-siRNA chimeras promotes regression of PSMA-expressing tumors. Nat Biotechnol 27, 839–846 (2009).
EpDT3	dPBS+5mM $MgCl_2$	85 °C for 5 minutes, cooled to room temperature for 10 min	Ht29	Flow cytometry staining performed in assay buffer, DPBS supplemented with 5mM MgCl2, 0.1mg/mL tRNA, 0.1mg/mL salmon sperm DNA, 0.2% sodium azide, and 5% FCS.	Shigdar, Sarah, et al. "RNA Aptamer Against a Cancer Stem Cell Marker Epithelial Cell Adhesion Molecule." Cancer Science, vol. 102, no. 5, 2011, pp. 991-998.
GL21.T	DMEM serum free	85°C for 5 min, snap-cooled on ice for 2 min then warmed to 37°C	HeLa	Binding analysis performed in DMEM serum free media (1). Proliferation assay preformed on A549 cells and U87MG cells in "complete growth medium". Molecule reported to work in vivo. (2)	(1) Cerchia L, Esposito CL, Jacobs AH, Tavitian B, de Franciscis V (2009) Differential SELEX in Human Glioma Cell Lines. PLoS ONE 4(11): e7971.<br><br>(2) Cerchia L, Esposito CL, Camorani S, Rienzo A, Stasio L, Insabato L, Affuso A, de Franciscis V (2012) Targeting Axl With an High-Affinity Inhibitory Aptamer. Mol. Ther. 23(12):2291-303.
CL4	DMEM serum free	85°C for 5 min, snap-cooled on ice for 2 min then warmed to 37°C	HeLa	Binding analysis performed in DMEM serum free media (1). Functional analysis (growth inhibition) performed in "complete growth medium". Molecule reported to work in vivo. (2)	(1) Cerchia L, Esposito CL, Jacobs AH, Tavitian B, de Franciscis V (2009) Differential SELEX in Human Glioma Cell Lines. PLoS ONE 4(11): e7971.<br><br>(2) Espositi CL, Passaro D, Longobardo I, Condorelli G, Marotta P, Affuso A, de Franciscis V, Cerchia L (2011) A Neutralizing RNA Aptamer against EGFR Causes Selective Apoptotic Cell Death PLoS ONE 6(9):e24071
2-2(t)	Saline	95 °C for 5 min	SKBr3	FACS analysis was performed in saline containing 0.1% albumin. Cell proliferation assays were performed in media containing 1% serum.	Mahlknecht, Georg, et al. "Aptamer to ErbB-2/HER2 Enhances Degradation of the Target and Inhibits Tumorigenic Growth." Proceedings of the National Academy of Sciences of the United States of America, vol. 110, no. 20, 2013, pp. 8170-8175.
SE15-8-mini	20 mM HEPES, 150 mM NaCl, 1 mM MgCl2	N/A	SKBr3	Binding assays performed in RNA binding buffer, 20mM HEPES (pH 7.0), 150mM NaCl, 1mM MgCl2, 2mM dithiothreitol, and 40U RNase inhibitor	Kim, Mee Y., and Sunjoo Jeong. "In Vitro Selection of RNA Aptamer and Specific Targeting of ErbB2 in Breast Cancer Cells." Nucleic Acid Therapeutics, vol. 21, no. 3, 2011, pp. 173-8.

Supplementary Table 5. Literature Reported Binding Affinities

Aptamer	Method	Ki/kd/IC50	Reference
A9	Enzymatic assay	Ki=2.1nM	Lupold, S. E., Hicke, B. J., Lin, Y. & Coffey, D. S. Identification and characterization of nuclease-stabilized RNA molecules that bind human prostate cancer cells via the prostate-specific membrane antigen. Cancer Res 62, 4029-4033 (2002).
A10-3	Enzymatic assay	Ki=20.5nM	Lupold, S. E., Hicke, B. J., Lin, Y. & Coffey, D. S. Identification and characterization of nuclease-stabilized RNA molecules that bind human prostate cancer cells via the prostate-specific membrane antigen. Cancer Res 62, 4029-4033 (2002).
A10-3.2	Cell Binding Filter assay	Kd=2.9nM	Dassie, J. P. et al. Systemic administration of optimized aptamer-siRNA chimeras promotes regression of PSMA-expressing tumors. Nature biotechnology 27, 839-849, doi:nbt.1560 [pii]
WAZ	Flow Cytometry	apparent Kd=390±40nM	Maier, K. E. et al. A New Transferrin Receptor Aptamer Inhibits New World Hemorrhagic Fever Mammarenavirus Entry. Mol Ther Nucleic Acids 5, e321, doi:10.1038/mtna.2016.32 (2016).
C2	Flow Cytometry	apparent Kd=104±6nM	Wilner, S. E. et al. An RNA Alternative to Human Transferrin: A New Tool for Targeting Human Cells. Mol Ther Nucleic Acids 1, e21, doi:10.1038/mtna.2012.14 (2012).
E07	Protein Filter binding	Kd=2.4nM	Li, N., Nguyen, H. H., Byrom, M. & Ellington, A. D. Inhibition of Cell Proliferation by an Anti-EGFR Aptamer. PLoS ONE 6, e20299, doi:10.1371/journal.pone.0020299 PONE-D-11-02209 [pii] (2011).
CL4	Protein Filter binding	Kd=10nM	Esposito, C. L. et al. A neutralizing RNA aptamer against EGFR causes selective apoptotic cell death. PLoS One 6, e24071, doi:10.1371/journal.pone.0024071 (2011).
GL21.T	Protein Filter binding	Kd=13nM	Cerchia, L. et al. Targeting Axl with an high-affinity inhibitory aptamer. Mol Ther 20, 2291-2303, doi:10.1038/mt.2012.163 (2012).
EpDT3	Flow Cytometry	approximate Kd=60nM	Shigdar, S. et al. RNA aptamer against a cancer stem cell marker epithelial cell adhesion molecule. Cancer Sci 102, 991-998, doi:10.1111/j.1349-7006.2011.01897.x (2011).
SE15-8-mini	Surface plasmon resonance	Kd=3.49nM	Kim, M. Y. & Jeong, S. In vitro selection of RNA aptamer and specific targeting of ErbB2 in breast cancer cells. Nucleic Acid Ther 21, 173-178, doi:10.1089/nat.2011.0283 (2011).
C1	Flow Cytometry	Used at 100nM	Magalhaes, M. L. et al. A general RNA motif for cellular transfection. Mol Ther 20, 616-624, doi:10.1038/mt.2011.277
XEO2-mini	Flow Cytometry	apparent Kd= 1500nM	Xiao, Z. et al. Engineering of targeted nanoparticles for cancer therapy using internalizing aptamers isolated by cell-uptake selection. ACS Nano 6, 696-704, doi:10.1021/nn204165v (2012).
AS1411	Flow Cytometry	Kd=54.8±7.3nM	Trinh, T. L. et al. A Synthetic Aptamer-Drug Adduct for Targeted Liver Cancer Therapy. PLoS One 10, e0136673, doi:10.1371/journal.pone.0136673 (2015).
SGC8c	Flow Cytometry	Kd=0.8nM	Shangguan, D., Tang, Z., Mallikaratchy, P., Xiao, Z. & Tan, W. Optimization and modifications of aptamers selected from live cancer cell lines. Chembiochem 8, 603-606, doi:10.1002/cbic.200600532 (2007).
2-2(t)	Flow Cytometry	Used at 1μM	Mahlknecht, G. et al. Aptamer to ErbB-2/HER2 enhances degradation of the target and inhibits tumorigenic growth. Proc Natl Acad Sci U S A 110, 8170-8175, doi:10.1073/pnas.1302594110 (2013).

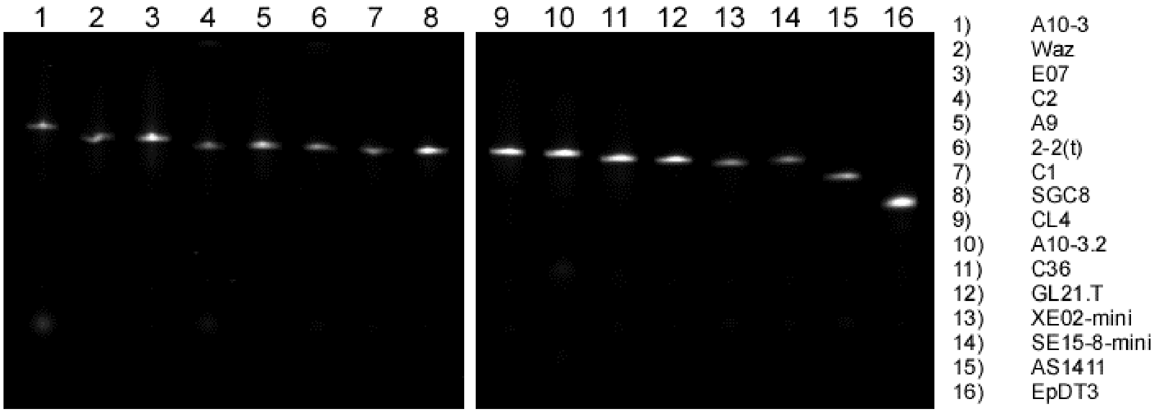

Supplementary Fig. 1. DyLight 650 labeled aptamers on a 12% polyacrylamide, 7M urea denaturing gel.

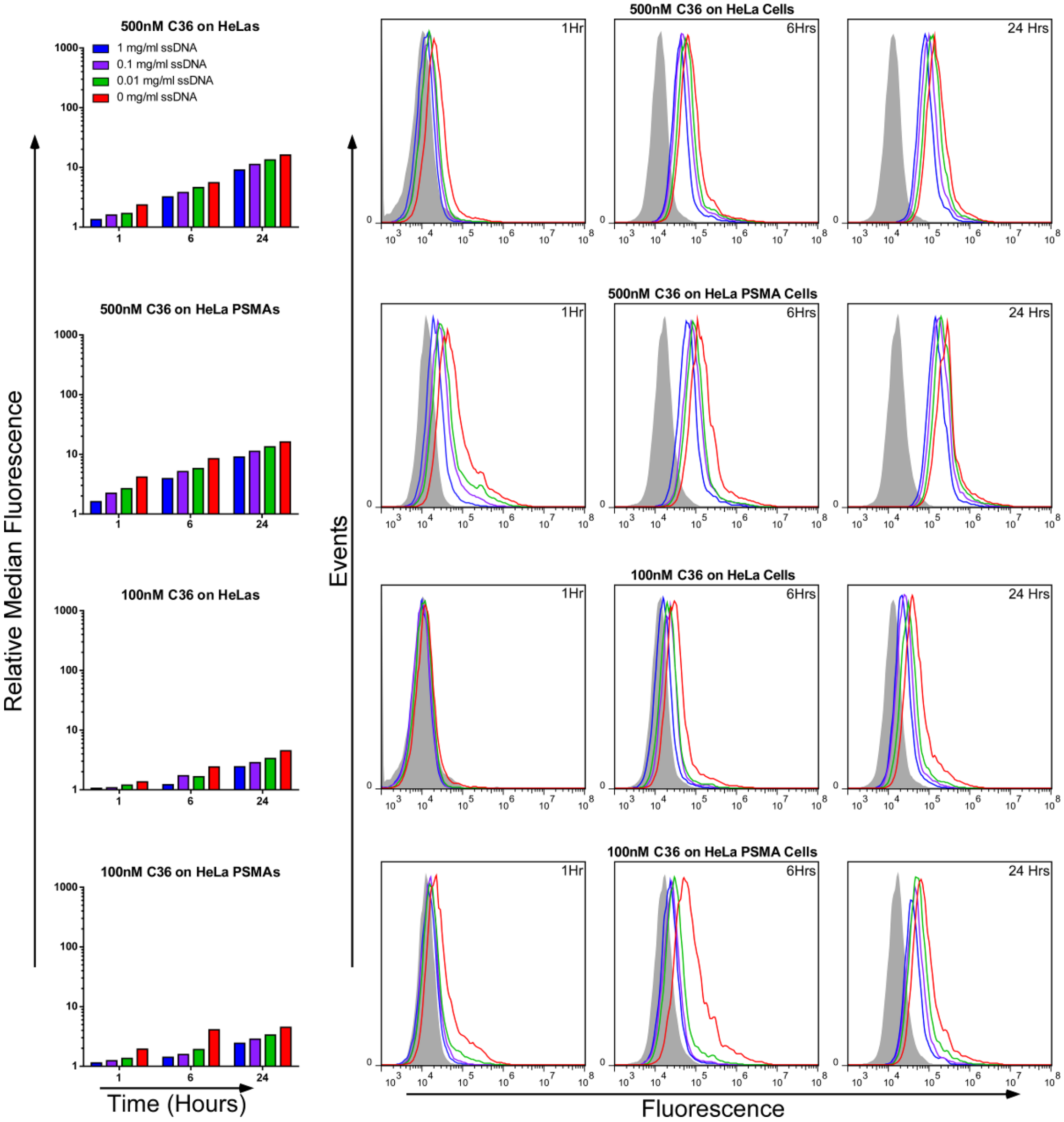

Supplementary Fig. 2. Non-specific control aptamer C36 incubated on HeLa cells at 500 nM (A) and 100 nM (B) and on HeLa PSMA cells at 500 nM (C) and 100 nM (D) with decreasing concentrations of ssDNA. Bar graphs represent median fluorescence of corresponding histograms relative to unstained cells (grey). The 500 nM data are included in **Figure 1.**

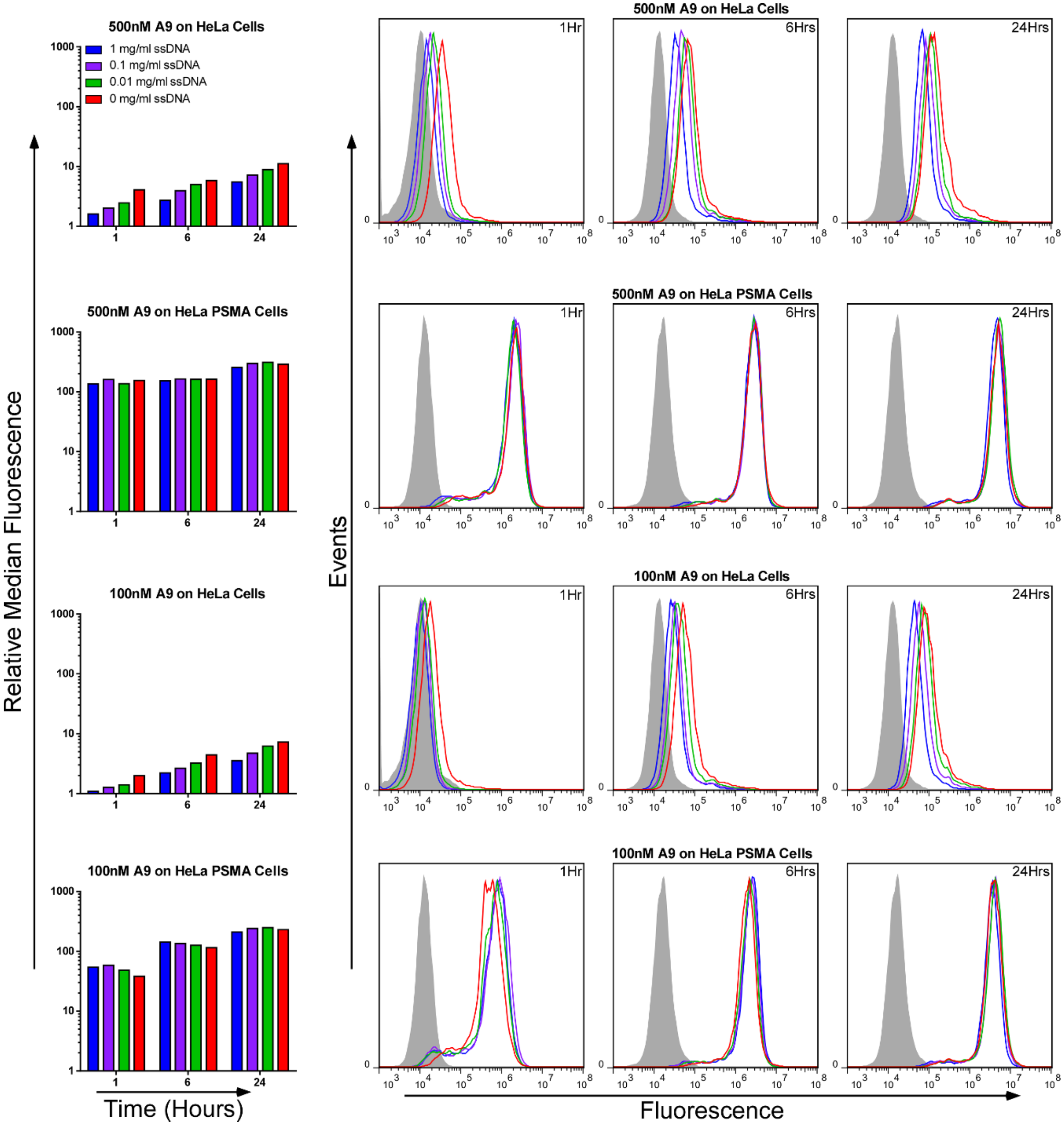

Supplementary Fig. 3. PSMA binding aptamer A9.min incubated on HeLa cells at 500 nM (A) and 100 nM (B) and on HeLa PSMA cells at 500 nM (C) and 100 nM (D) with decreasing concentrations of ssDNA. Bar graphs represent median fluorescence of corresponding histograms relative to unstained cells (grey). The 500 nM data are included in **Figure 1.**

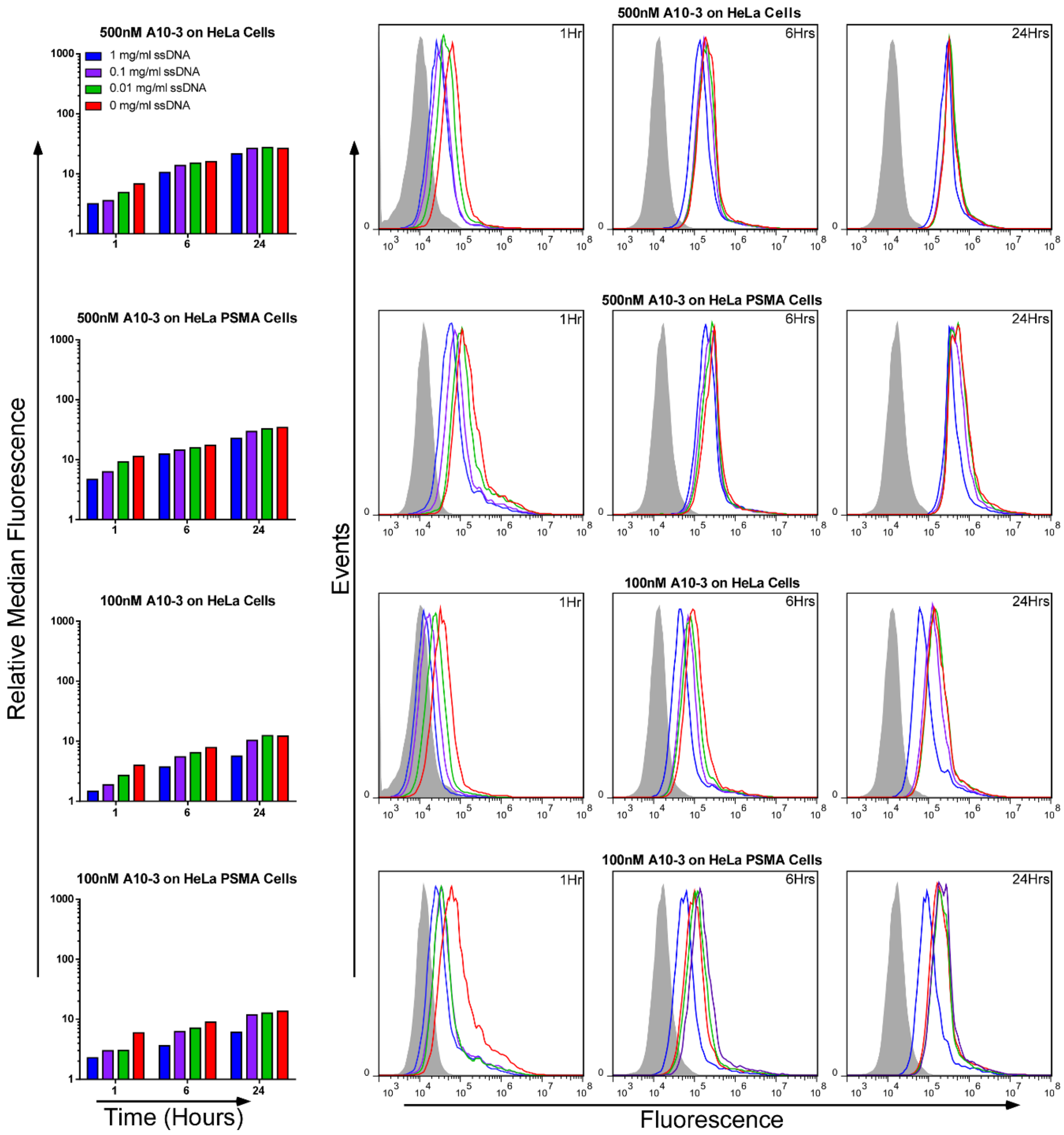

Supplementary Fig. 4. PSMA targeted aptamer A10-3 incubated on HeLa cells at 500 nM (A) and 100 nM (B) and on HeLa PSMA cells at 500 nM (C) and 100 nM (D) with decreasing concentrations of ssDNA. Bar graphs represent median fluorescence of corresponding histograms relative to unstained cells (grey). The 500 nM data are included in **Figure 1.**

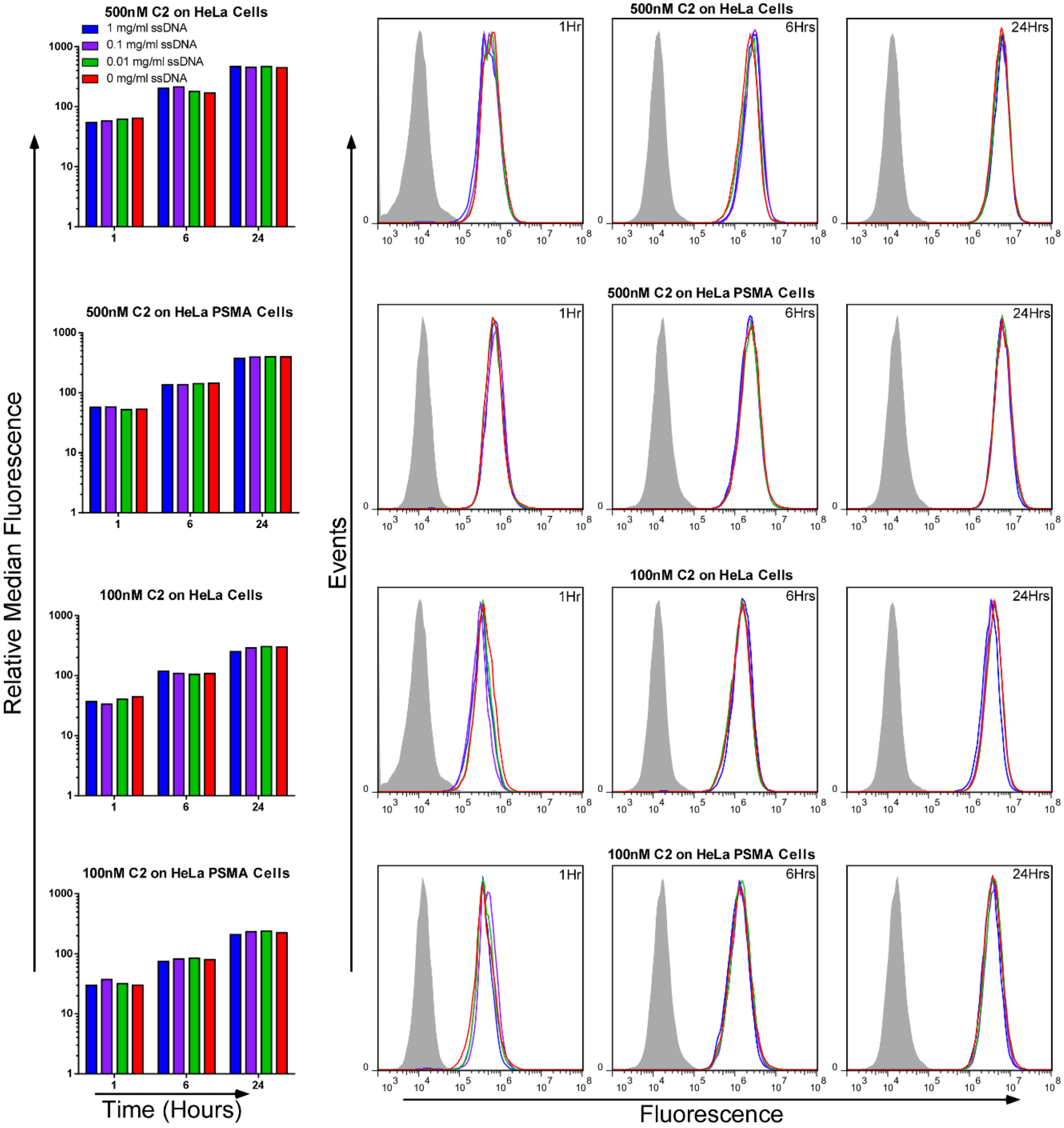

Supplementary Fig. 5. hTfR binding aptamer C2 incubated on HeLa cells at 500 nM (A) and 100 nM (B) and on HeLa PSMA cells at 500 nM (C) and 100 nM (D) with decreasing concentrations of ssDNA. Bar graphs represent median fluorescence of corresponding histograms relative to unstained cells (grey). The 500 nM data are included in **Figure 1.**

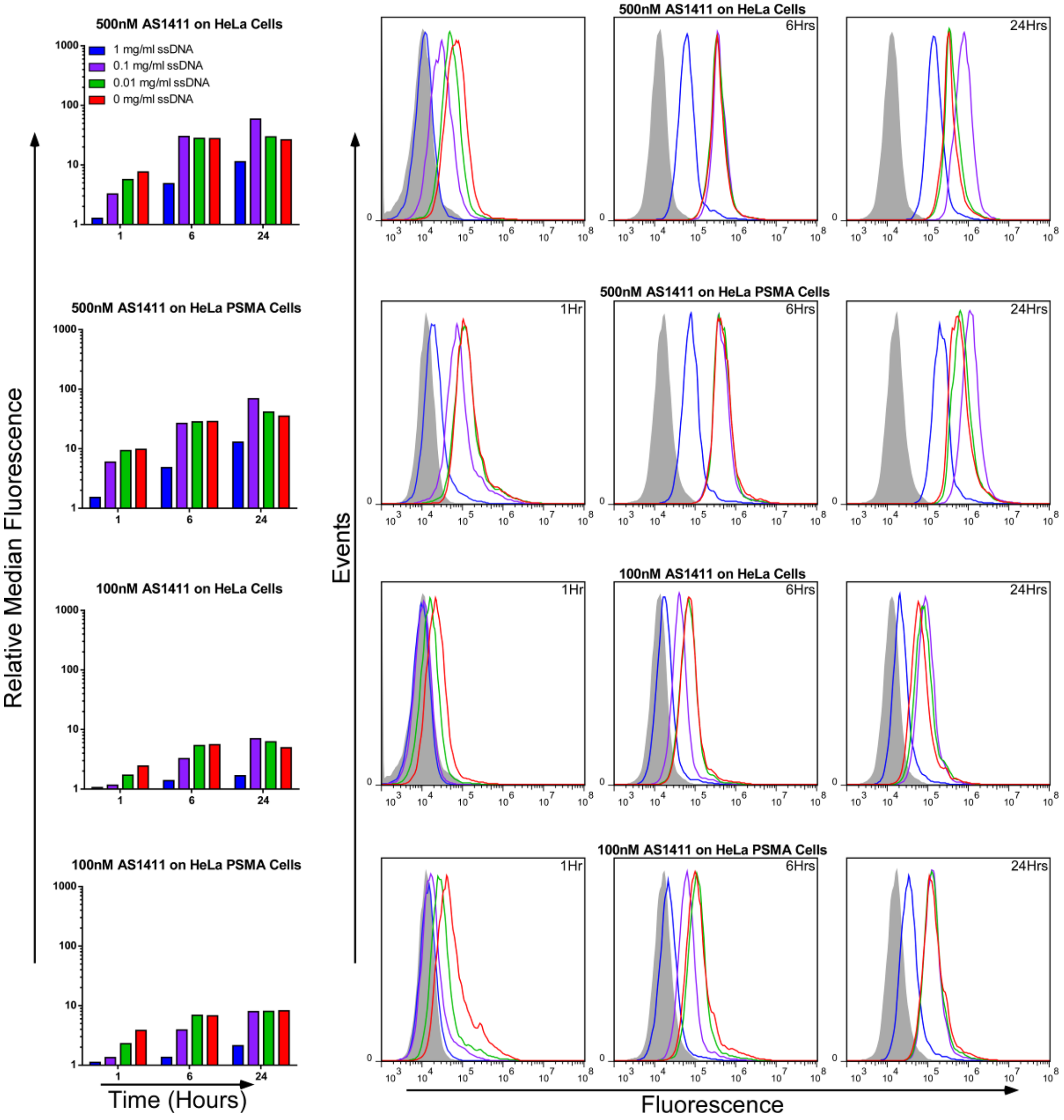

Supplementary Fig. 6. Nucleolin targeted aptamer AS1411 incubated on HeLa cells at 500 nM (A) and 100 nM (B) and on HeLa PSMA cells at 500 nM (C) and 100 nM (D) with decreasing concentrations of ssDNA. Bar graphs represent median fluorescence of corresponding histograms relative to unstained cells (grey). The 500 nM data are included in **Figure 1.**

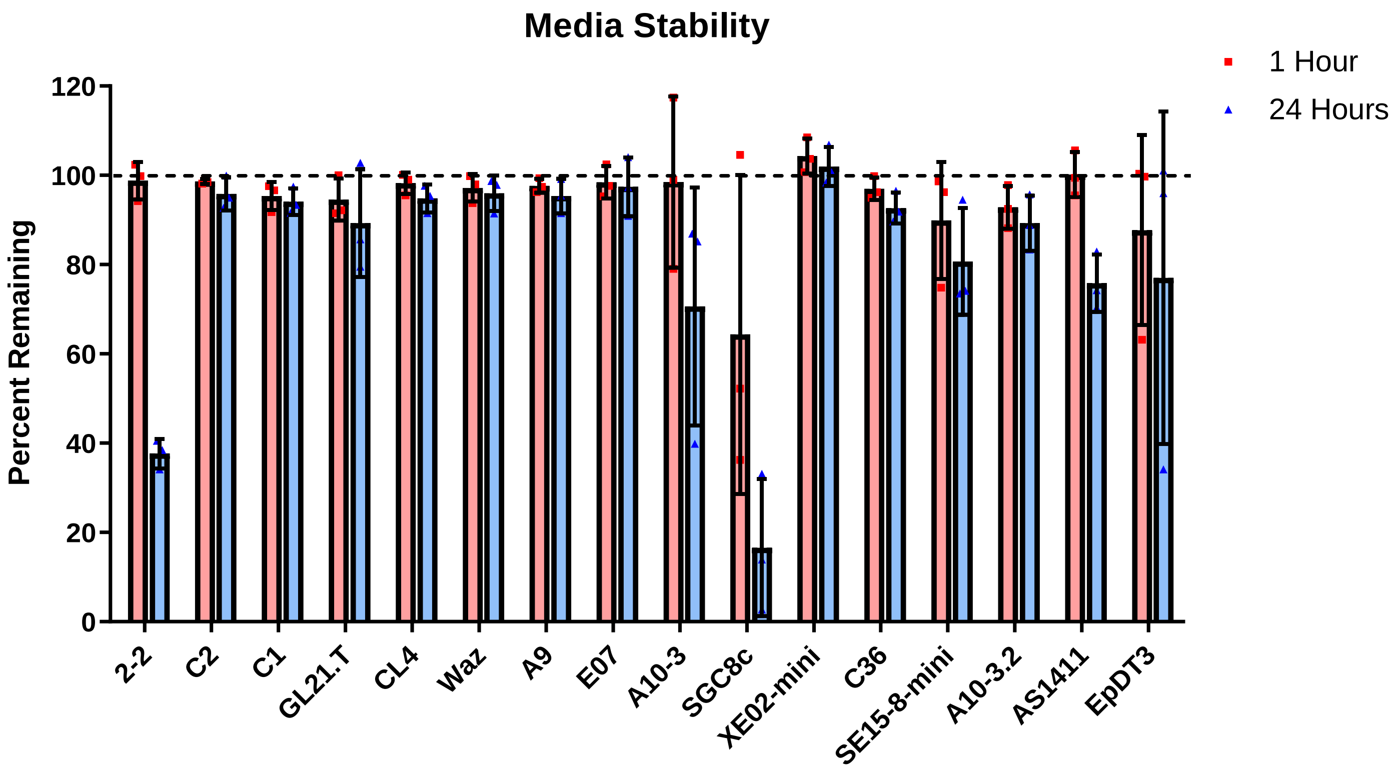

Supplementary Fig. 7. Aptamer stability in media. Aptamers were incubated in DMEM containing 10% FBS for 0, 1 or 24 hours. The amount of full-length aptamer remaining was determined by gel electrophoresis. 100% corresponds to the amount present at time = 0 hr. Data are the mean +/- SD of 3 independent replicates.

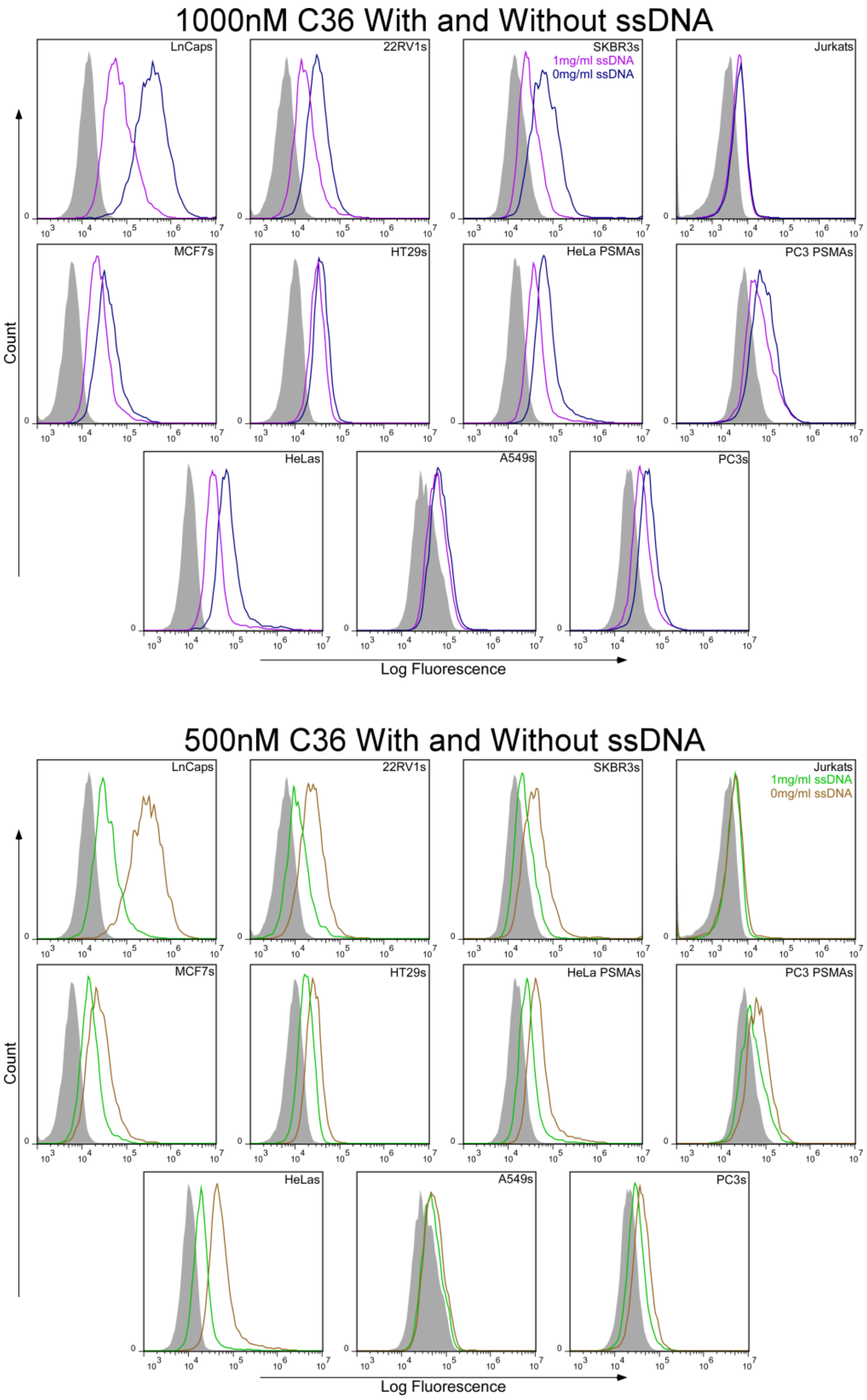

Supplementary Fig. 8. Histograms used to generate data shown in Figure 2**.** The control sequence, C36, was incubated at 1000 nM and 500 nM with the cell line indicated in full growth media or full growth media supplemented with 1 mg/ml ssDNA. Cells were incubated for one hour at 37°. Unstained cells are shown in grey. The presence of ssDNA is indicated in the key shown in the upper left histogram of each data set. Data are representative of two independent trials.

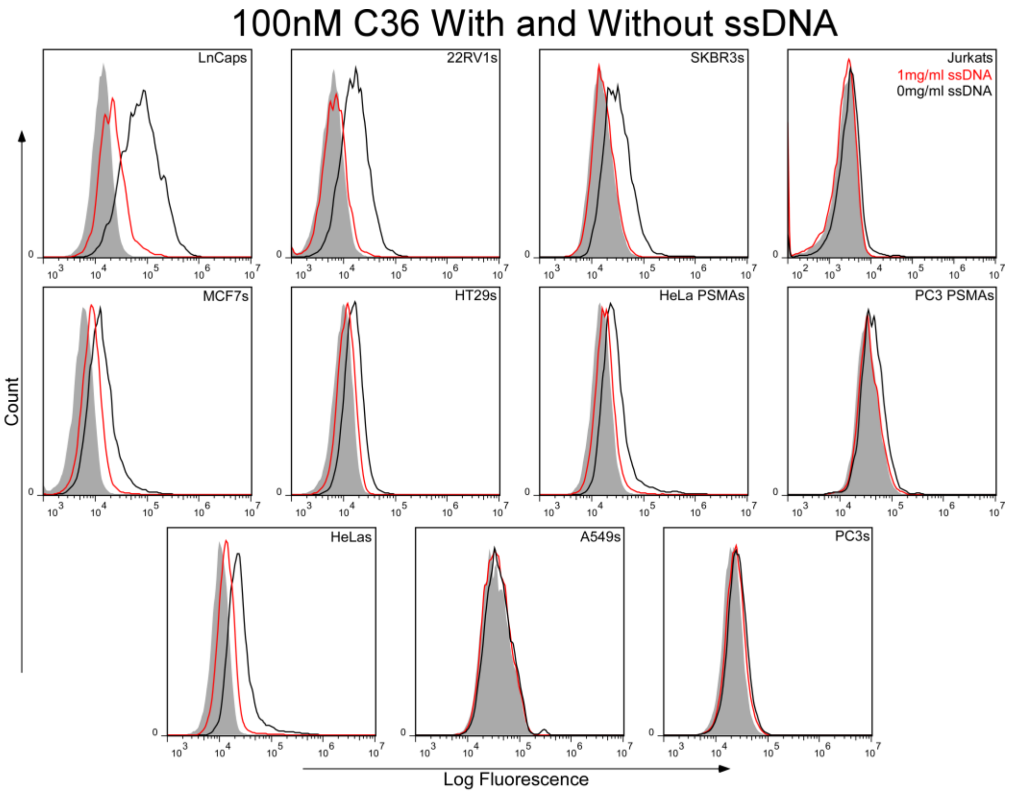

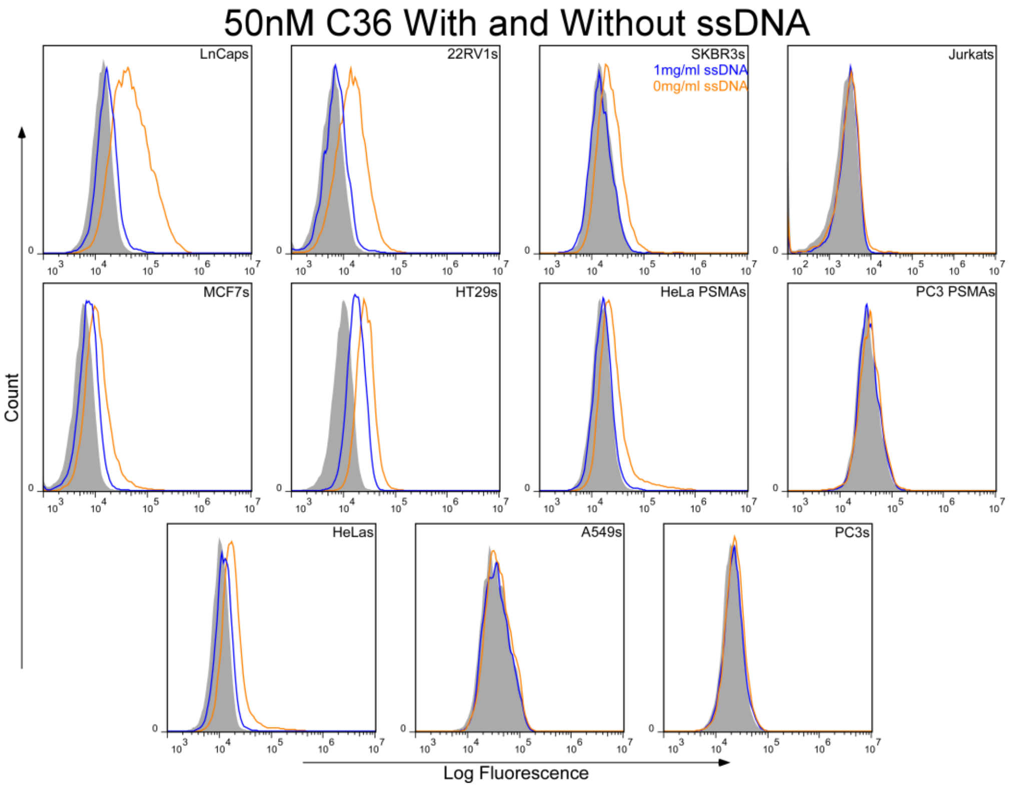

Supplementary Fig. 9. Histograms used to generate data shown in Figure 2**.** The control sequence, C36, was incubated at 100 nM and 50 nM with the cell line indicated in full growth media, or full growth media supplemented with 1 mg/ml ssDNA. Cells were incubated for one hour at 37°. Unstained cells are shown in gray. The presence of ssDNA is indicated in the key shown in the upper left histogram of each data set. Data are representative of three independent trials.

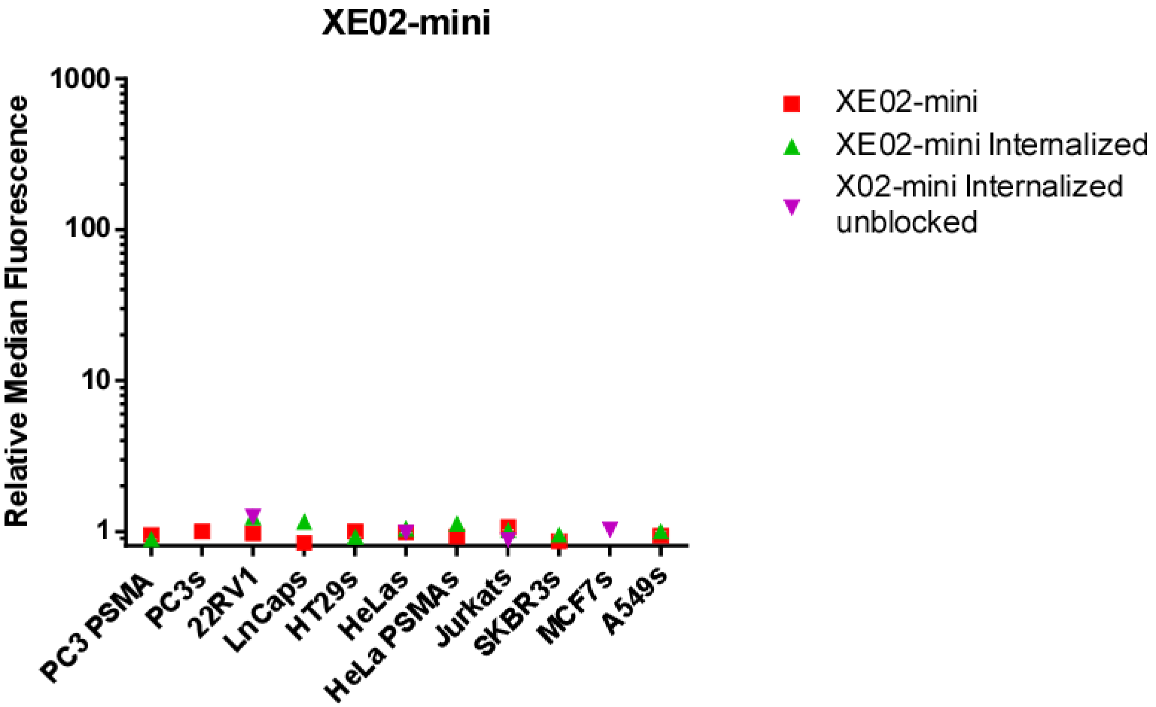

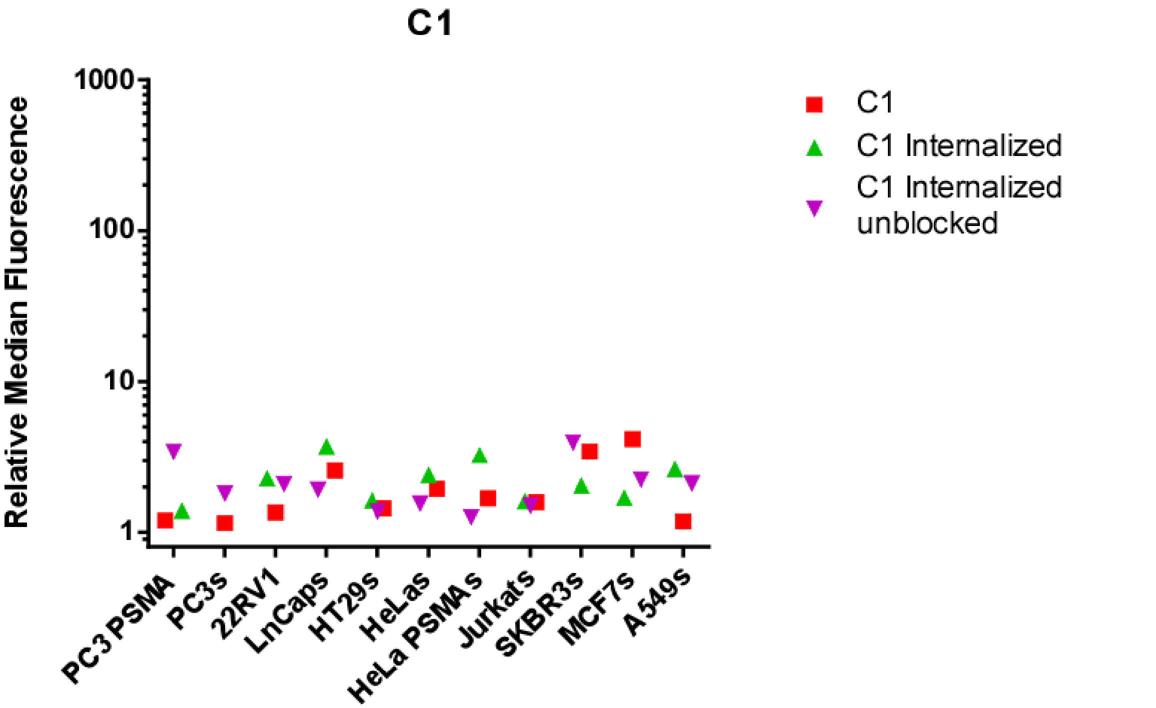

Supplementary Fig. 10. Binding plots for XE02-mini (top) and C1 (bottom). Aptamers were incubated at 500 nM and shown relative to the fluorescence of C36 control.

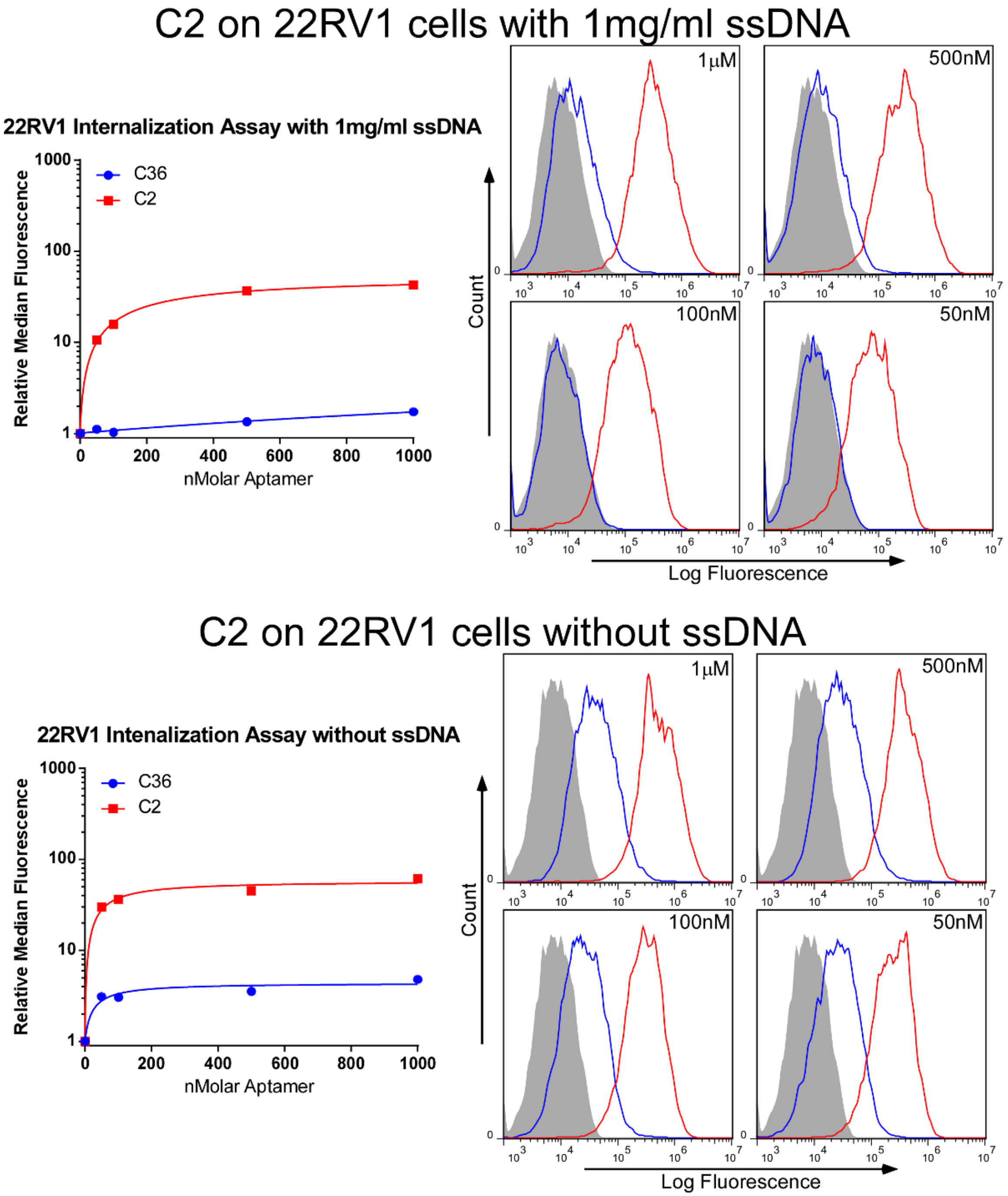

Supplementary Fig. 11. hTfR binding aptamer C2 internalization and binding assays on 22RV1 cells. Graphs represent the median fluorescence of the aptamer (Red) and C36 (Blue) relative to unstained cells (Gray).

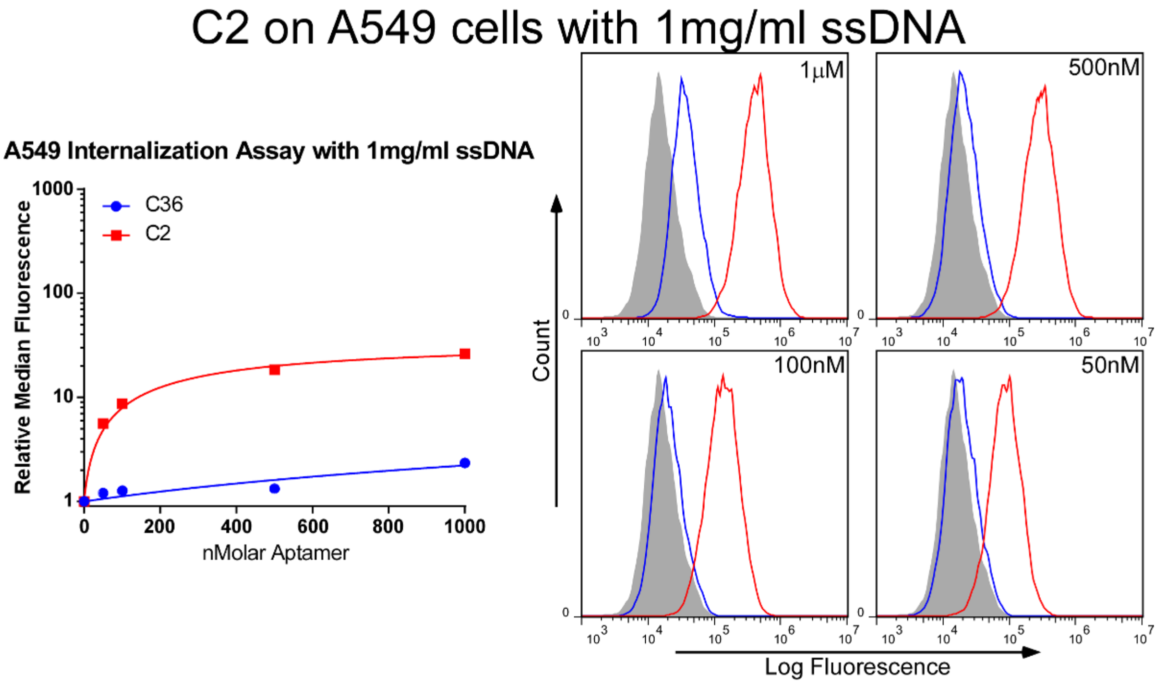

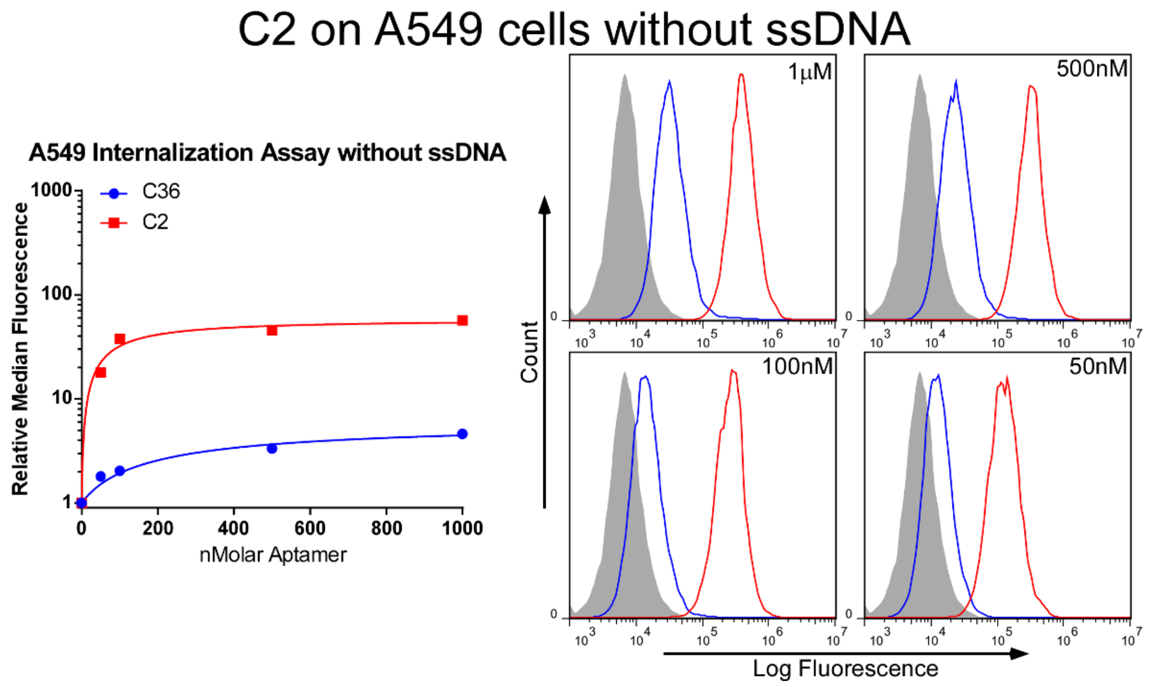

Supplementary Fig. 12. hTfR binding aptamer C2 internalization and binding assays on A549 cells. Graphs represent the median fluorescence of the aptamer (Red) and C36 (Blue) relative to unstained cells (Gray).

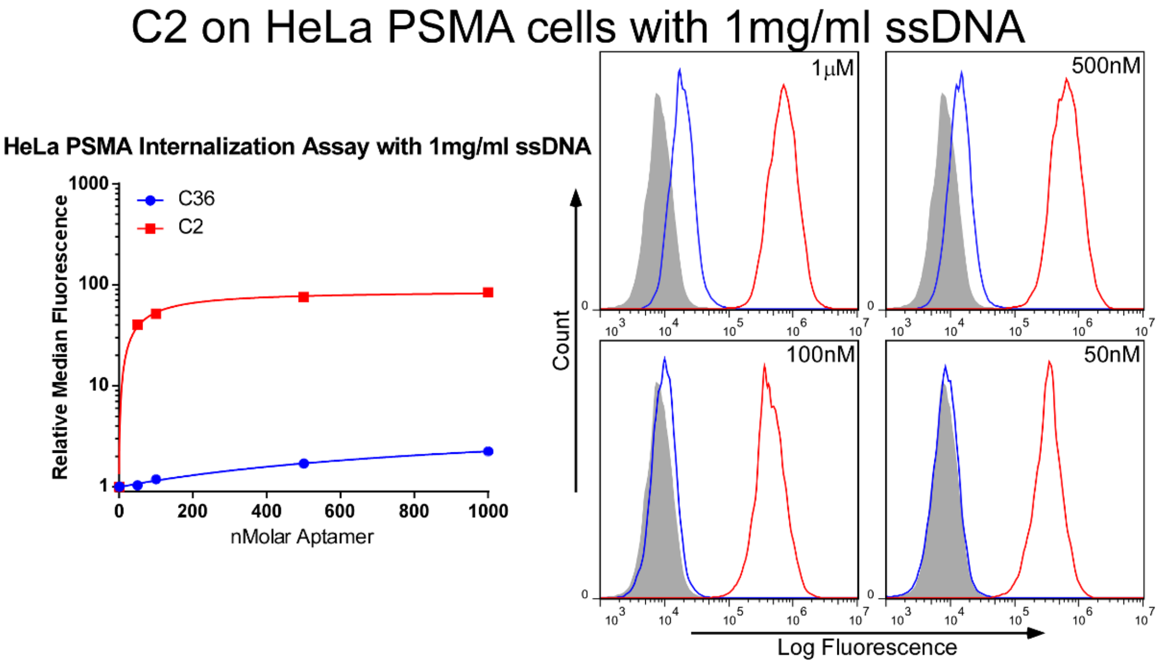

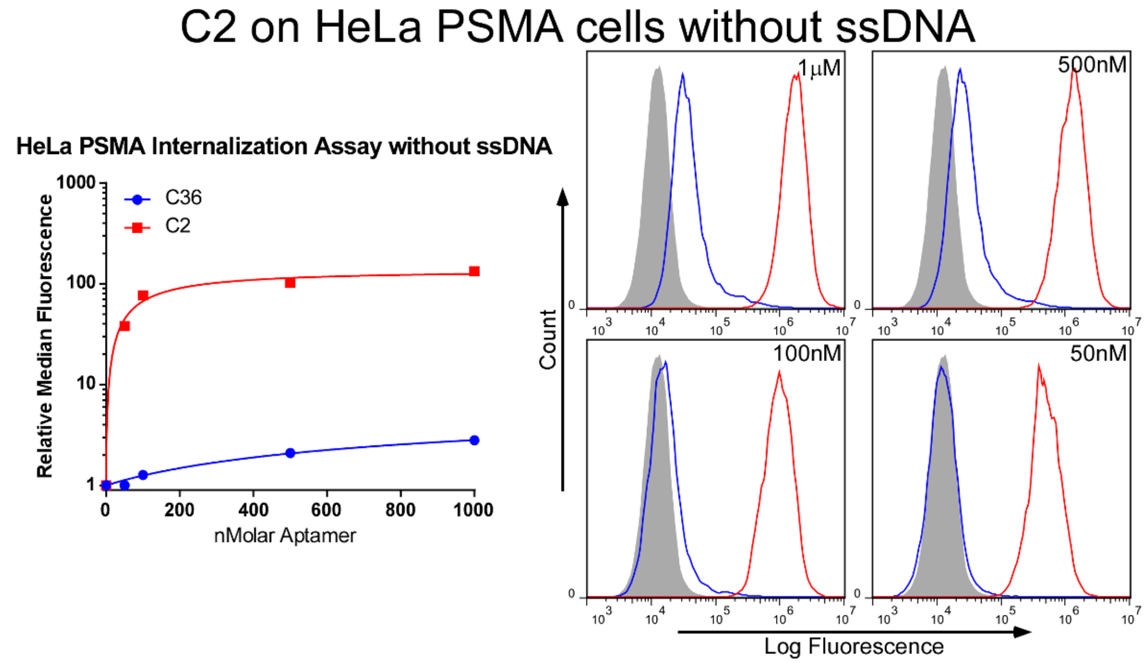

Supplementary Fig. 13. hTfR binding aptamer C2 internalization and binding assays on HeLa PSMA cells. Graphs represent the median fluorescence of the aptamer (Red) and C36 (Blue) relative to unstained cells (Gray).

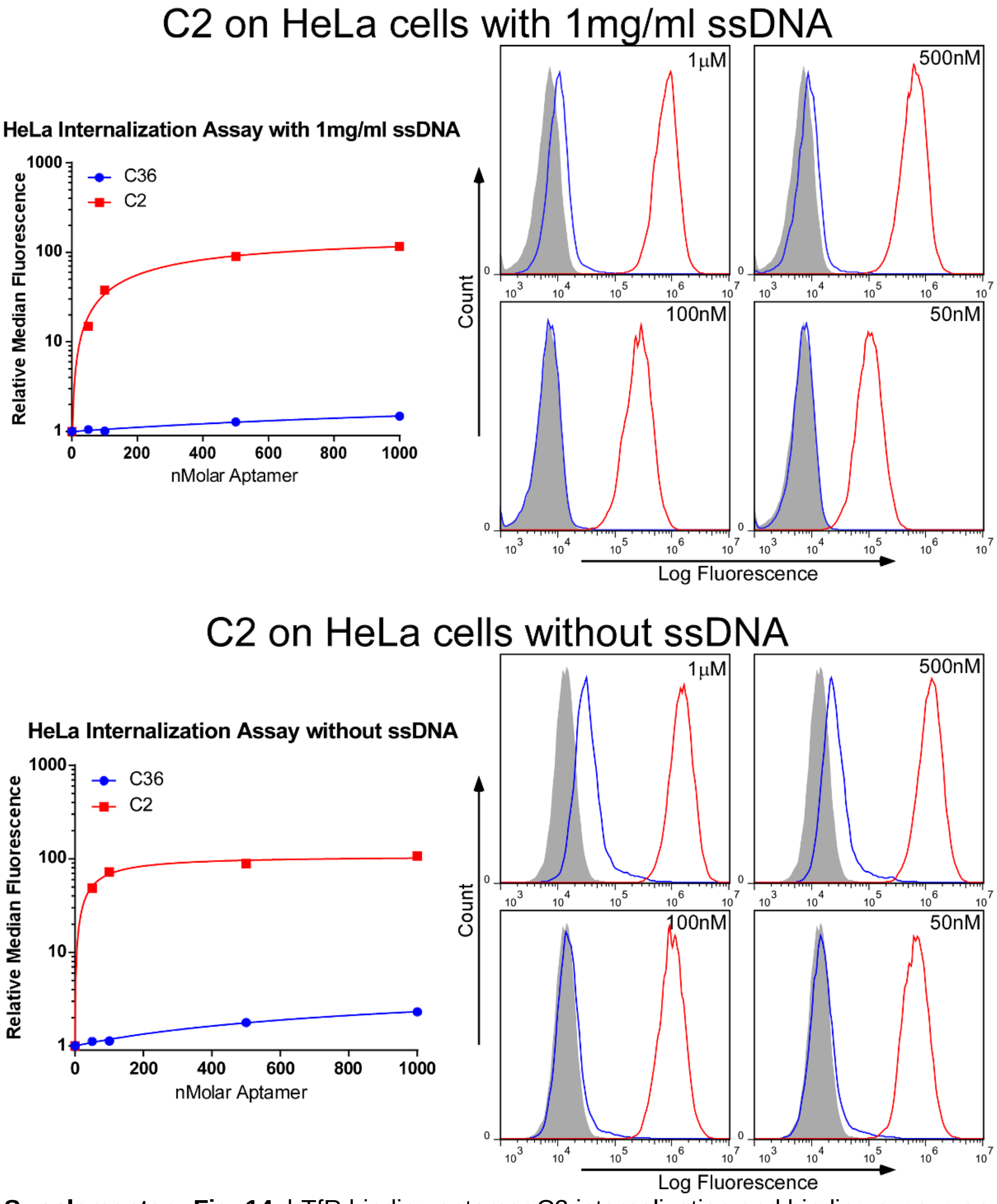

Supplementary Fig. 14. hTfR binding aptamer C2 internalization and binding assays on HeLa cells. Graphs represent the median fluorescence of the aptamer (Red) and C36 (Blue) relative to unstained cells (Gray).

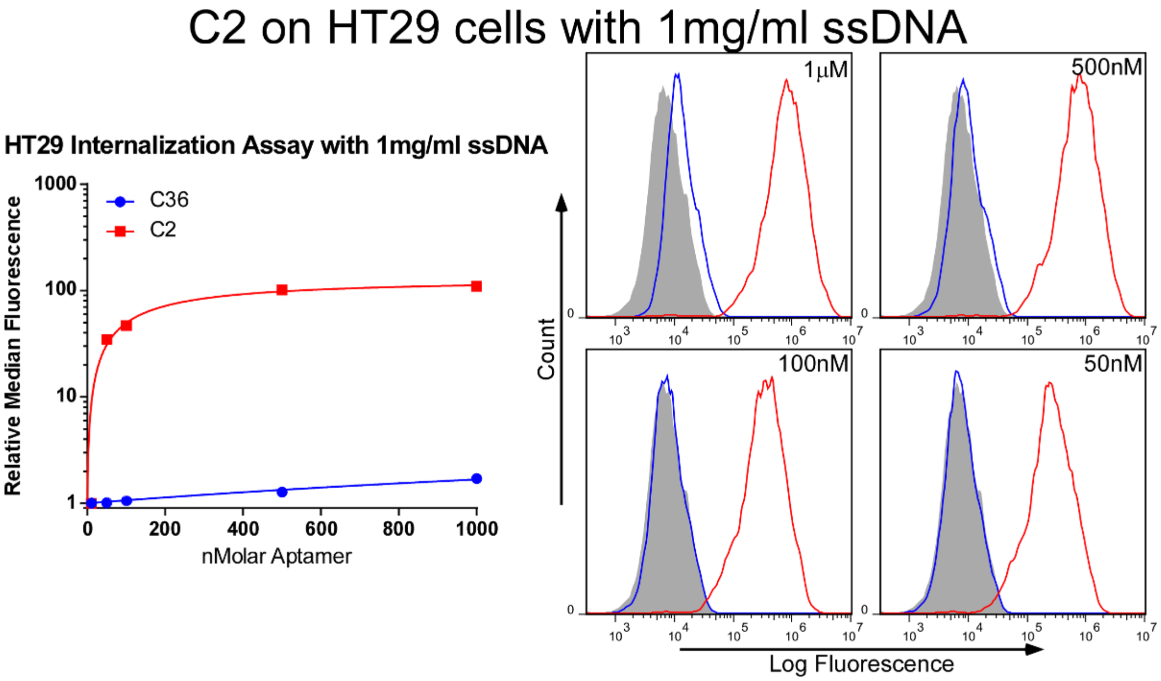

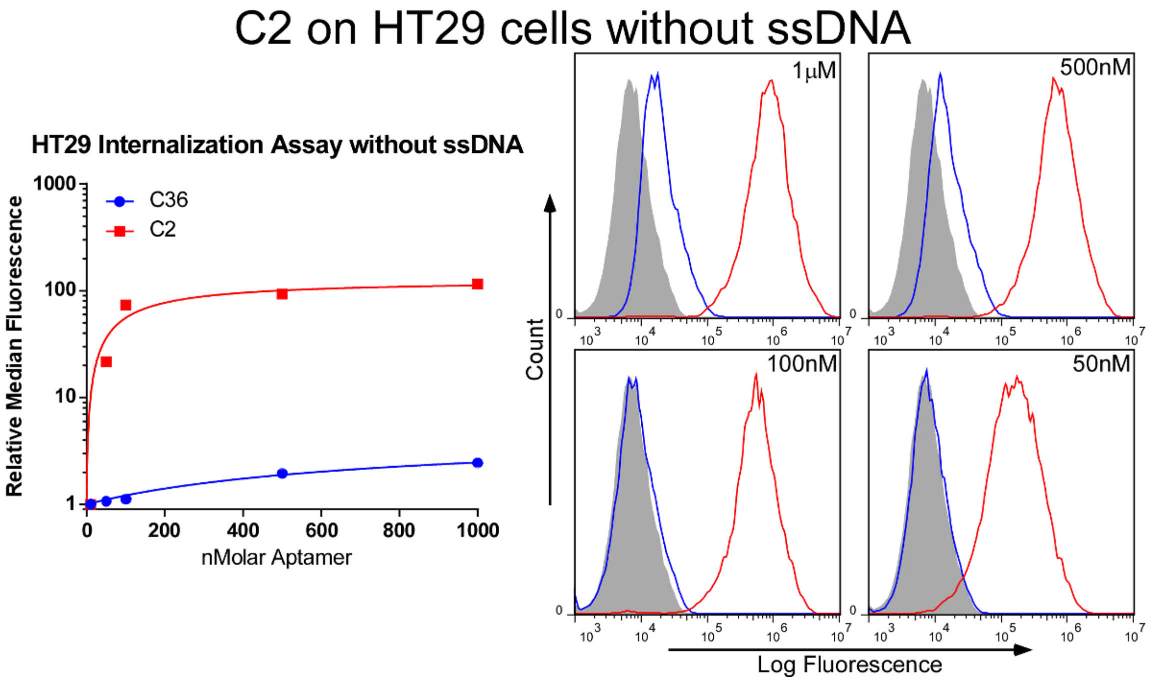

Supplementary Fig. 15. hTfR binding aptamer C2 internalization and binding assays on HT29 cells. Graphs represent the median fluorescence of the aptamer (Red) and C36 (Blue) relative to unstained cells (Gray).

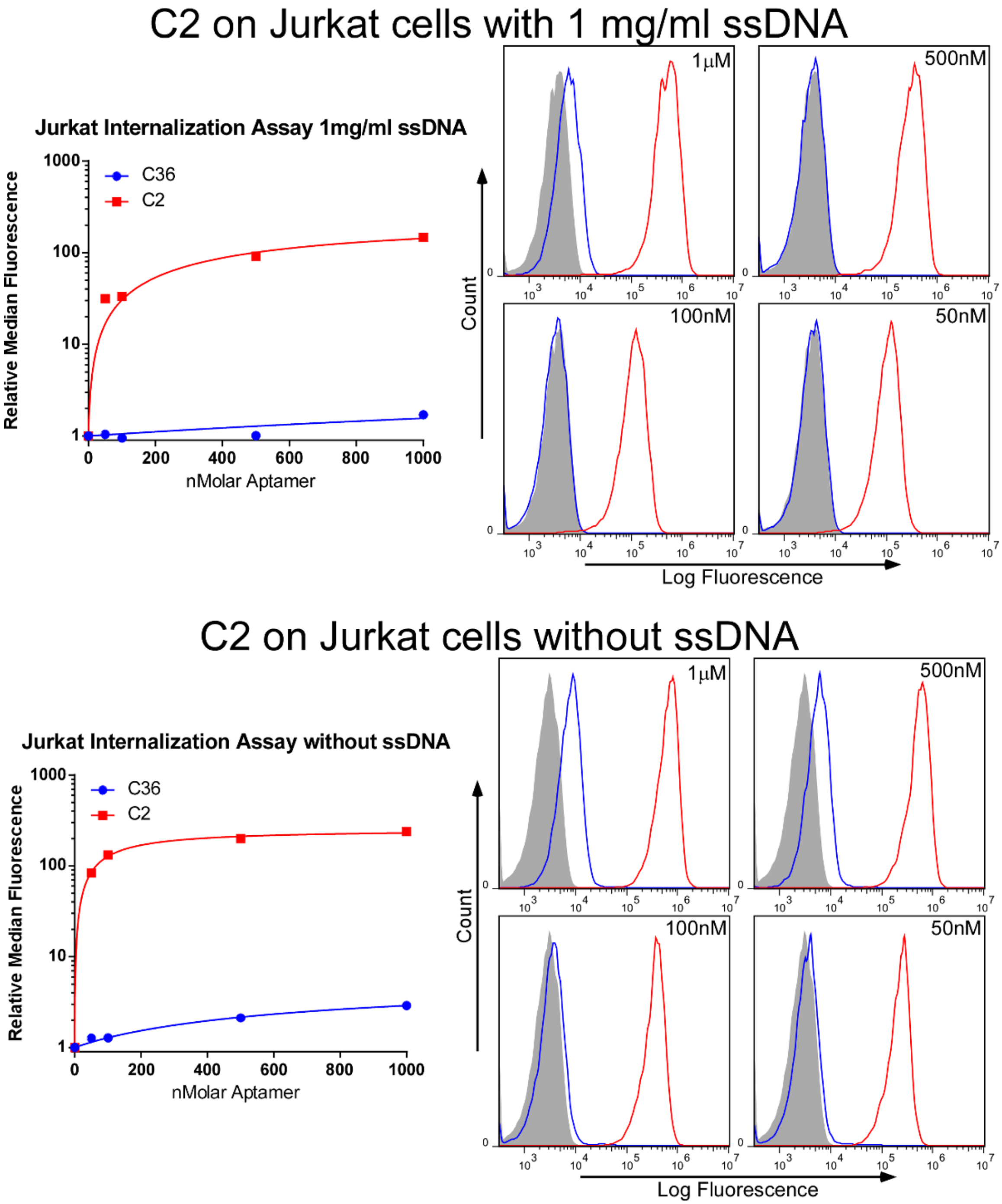

Supplementary Fig. 16. hTfR binding aptamer C2 internalization and binding assays on Jurkat cells. Graphs represent the median fluorescence of the aptamer (Red) and C36 (Blue) relative to unstained cells (Gray).

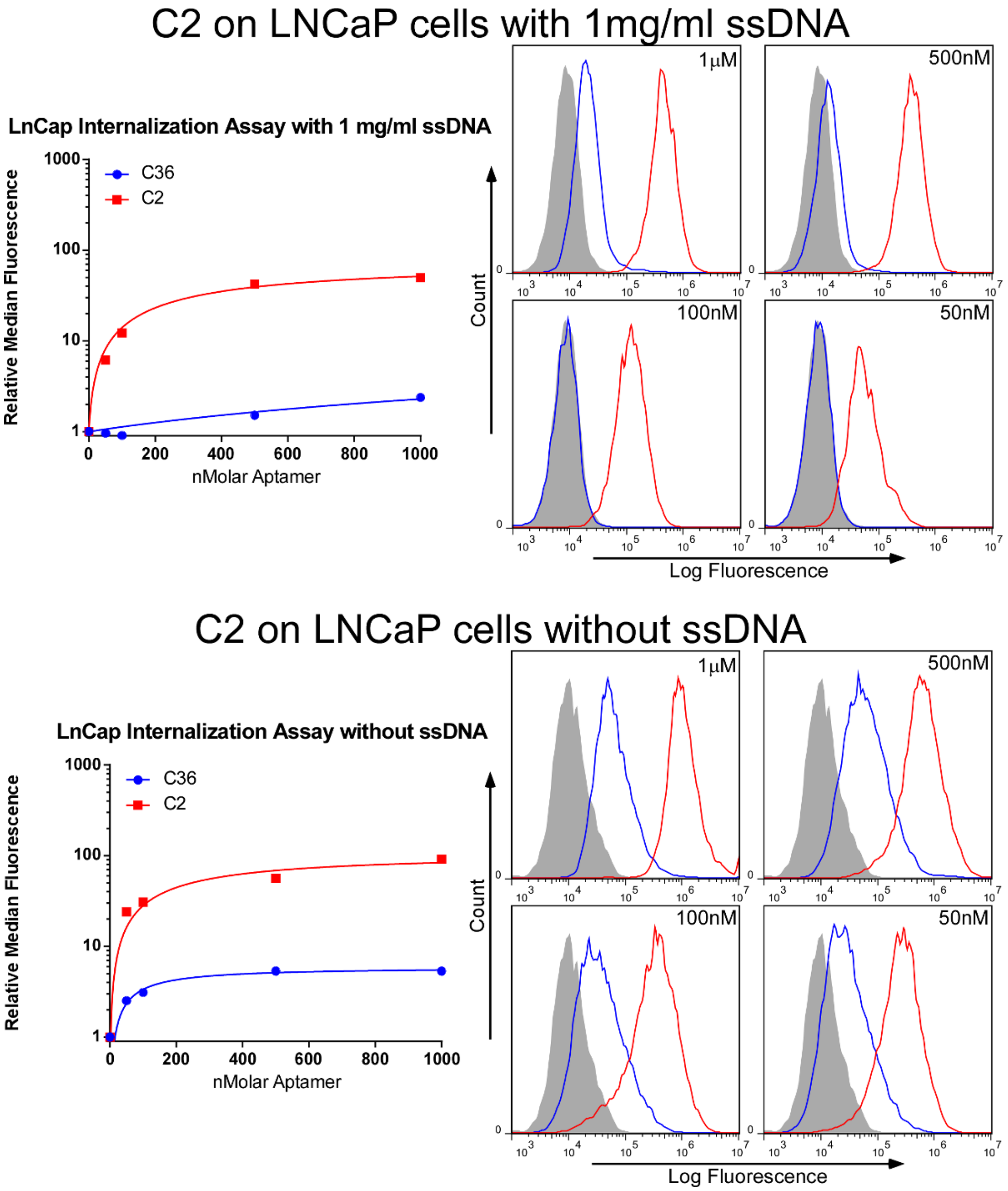

Supplementary Fig. 17. hTfR binding aptamer C2 internalization and binding assays on LNCaP cells. Graphs represent the median fluorescence of the aptamer (Red) and C36 (Blue) relative to unstained cells (Gray).

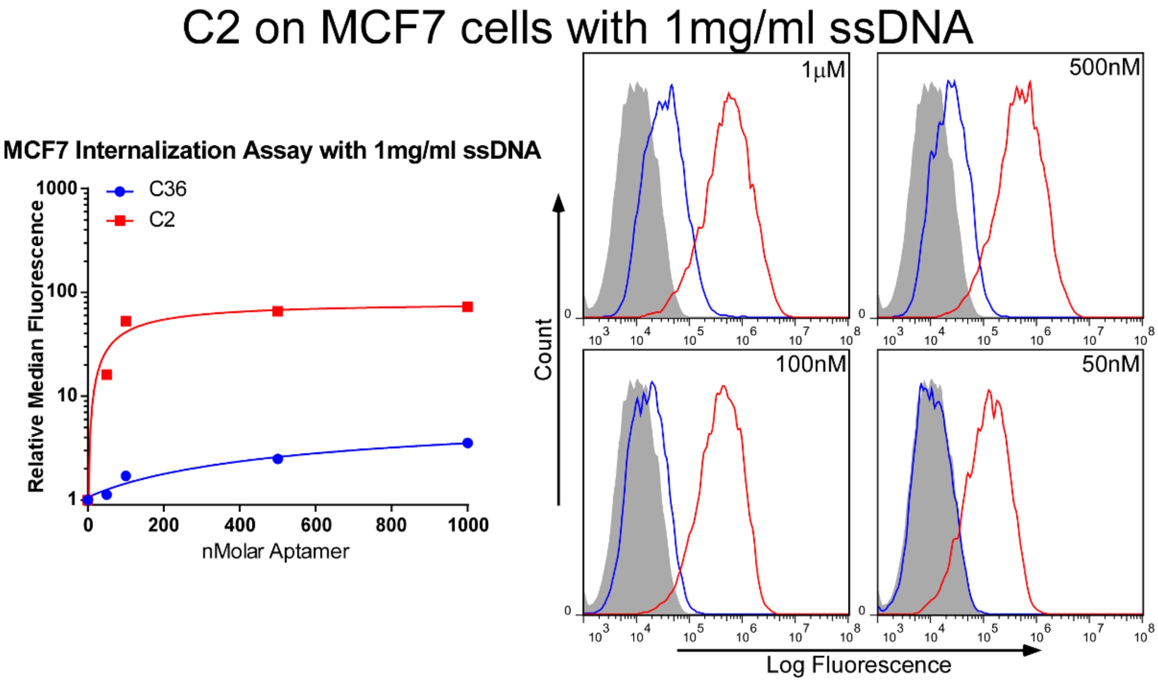

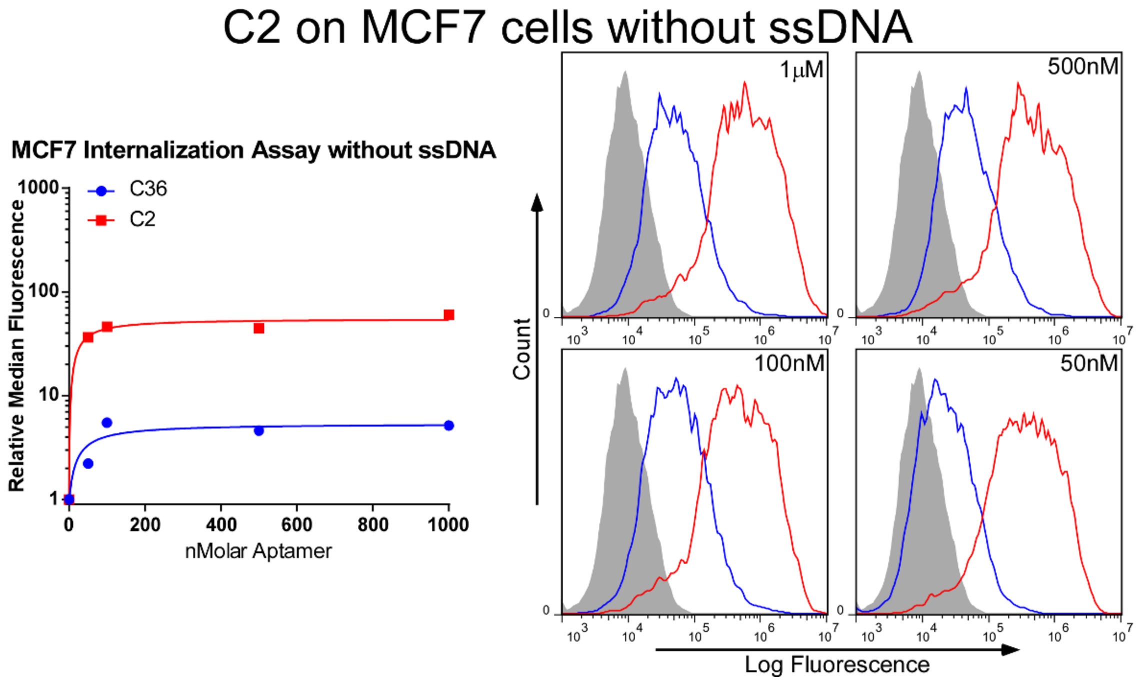

Supplementary Fig. 18. hTfR binding aptamer C2 internalization and binding assays on MCF7 cells. Graphs represent the median fluorescence of the aptamer (Red) and C36 (Blue) relative to unstained cells (Gray).

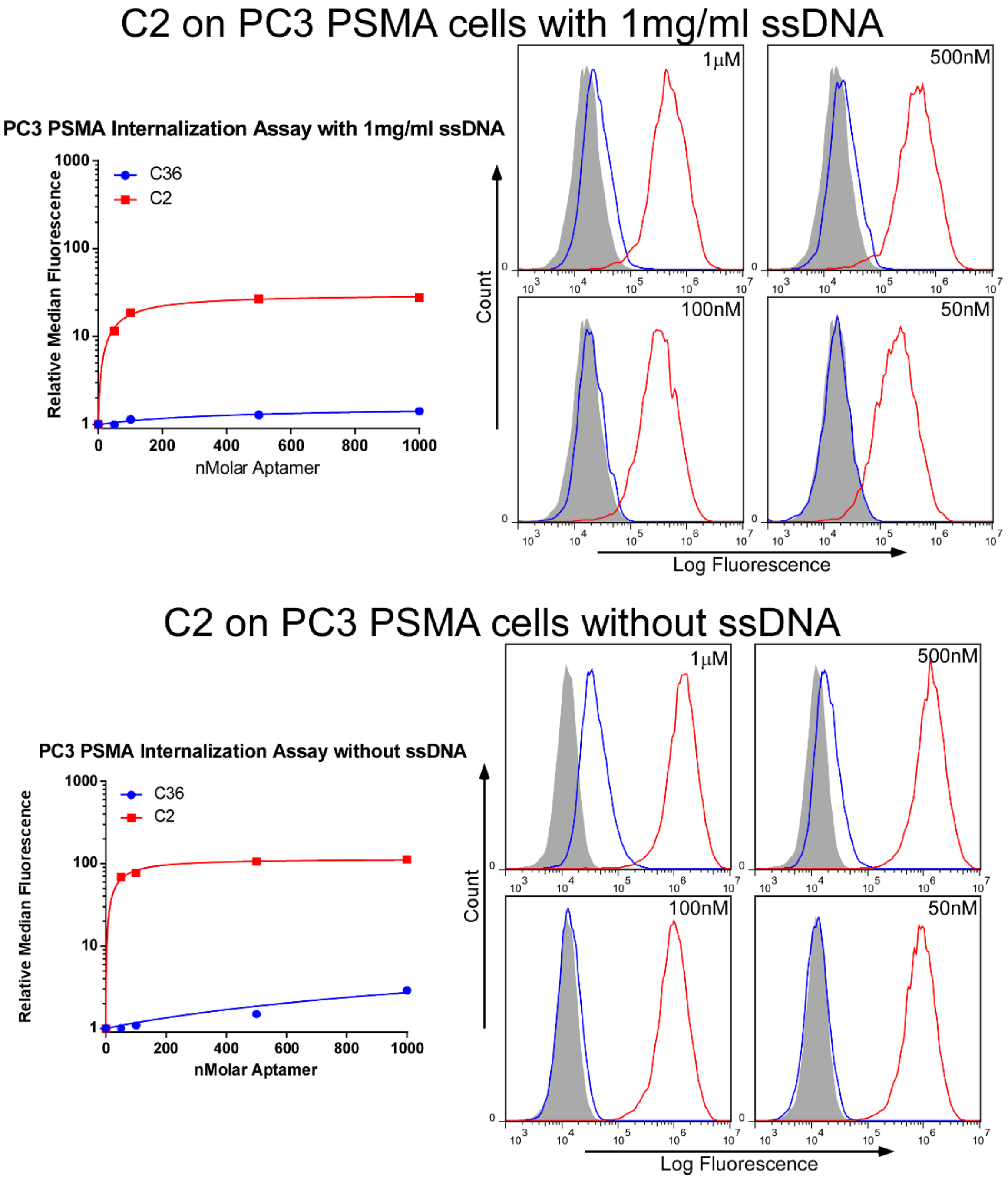

Supplementary Fig. 19. hTfR binding aptamer C2 internalization and binding assays on PC3 PSMA cells. Graphs represent the median fluorescence of the aptamer (Red) and C36 (Blue) relative to unstained cells (Gray).

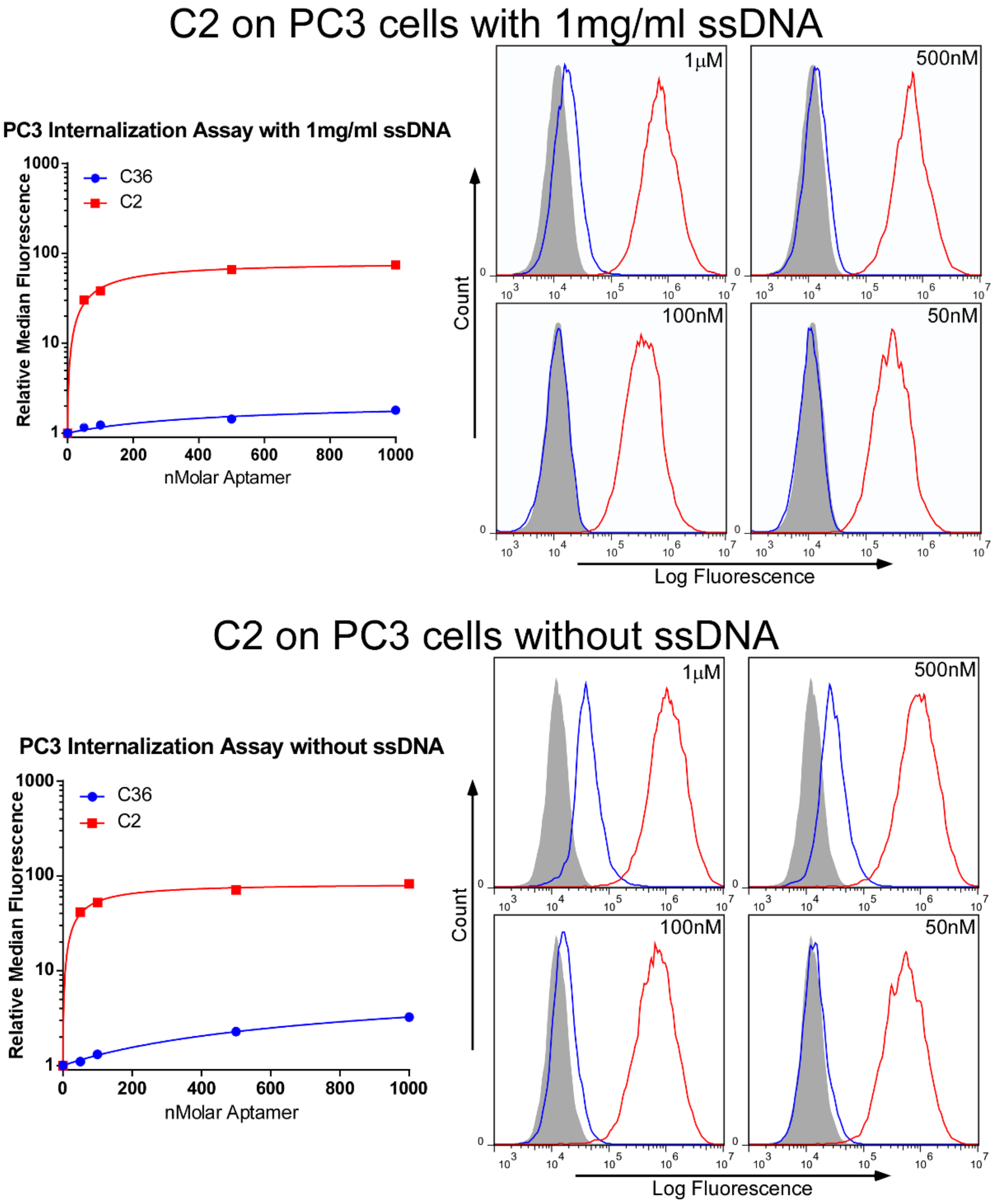

Supplementary Fig. 20. hTfR binding aptamer C2 internalization and binding assays on PC3 cells. Graphs represent the median fluorescence of the aptamer (Red) and C36 (Blue) relative to unstained cells (Gray).

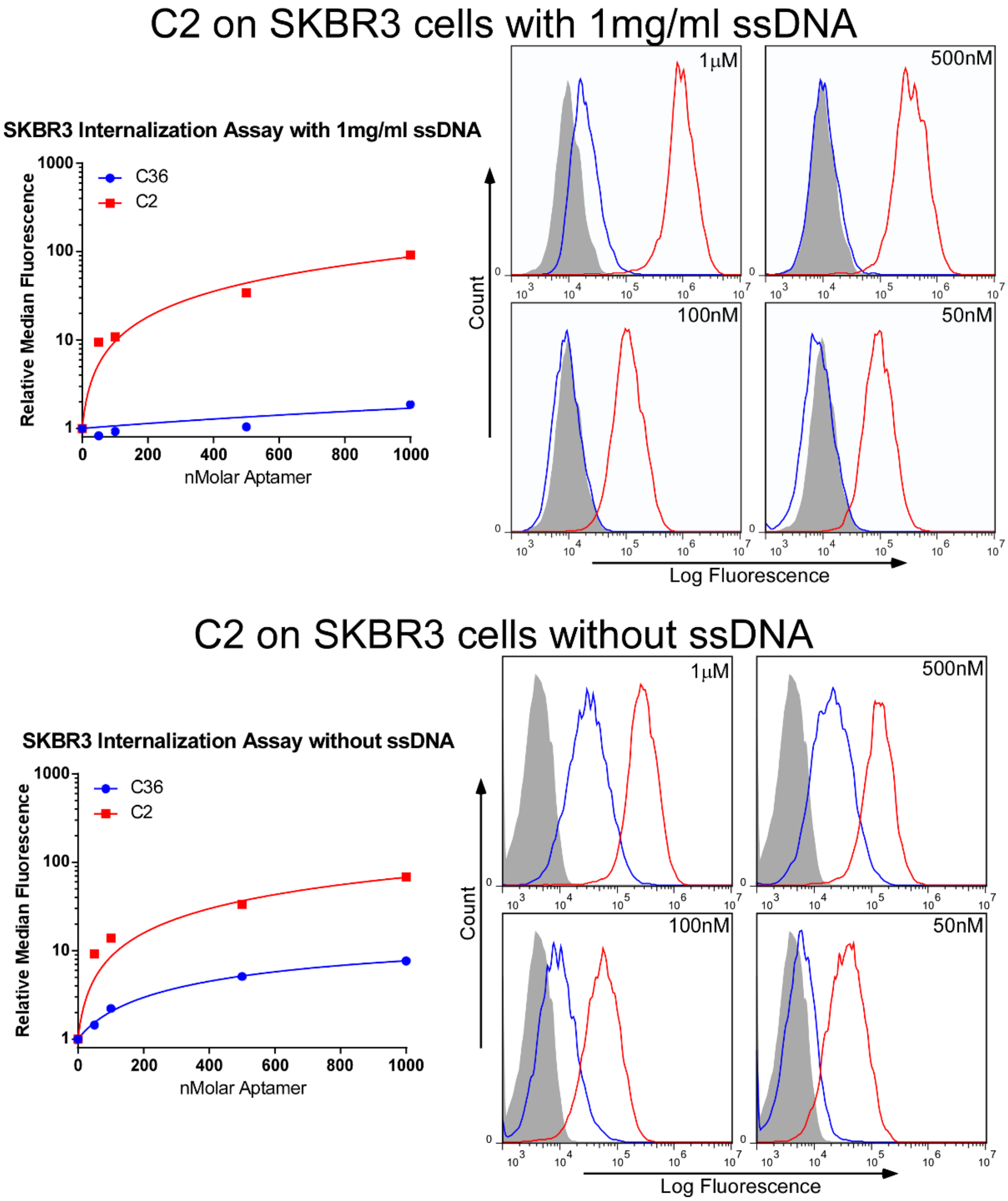

Supplementary Fig. 21. hTfR binding aptamer C2 internalization and binding assays on SKBR3 cells. Graphs represent the median fluorescence of the aptamer (Red) and C36 (Blue) relative to unstained cells (Gray).

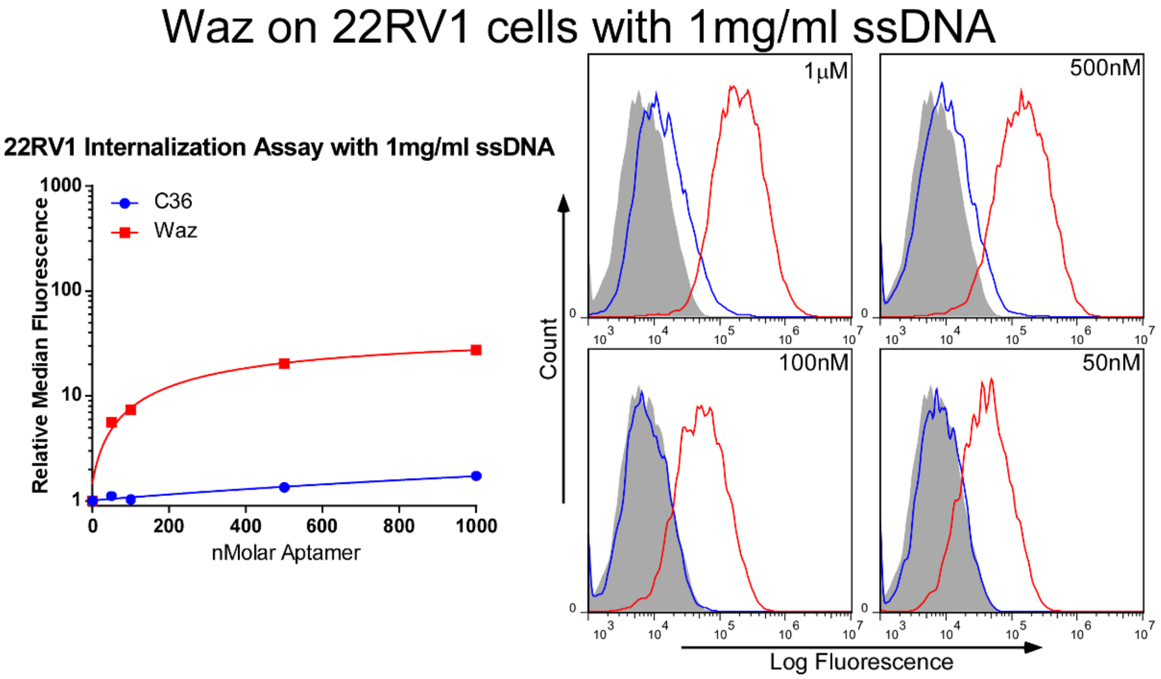

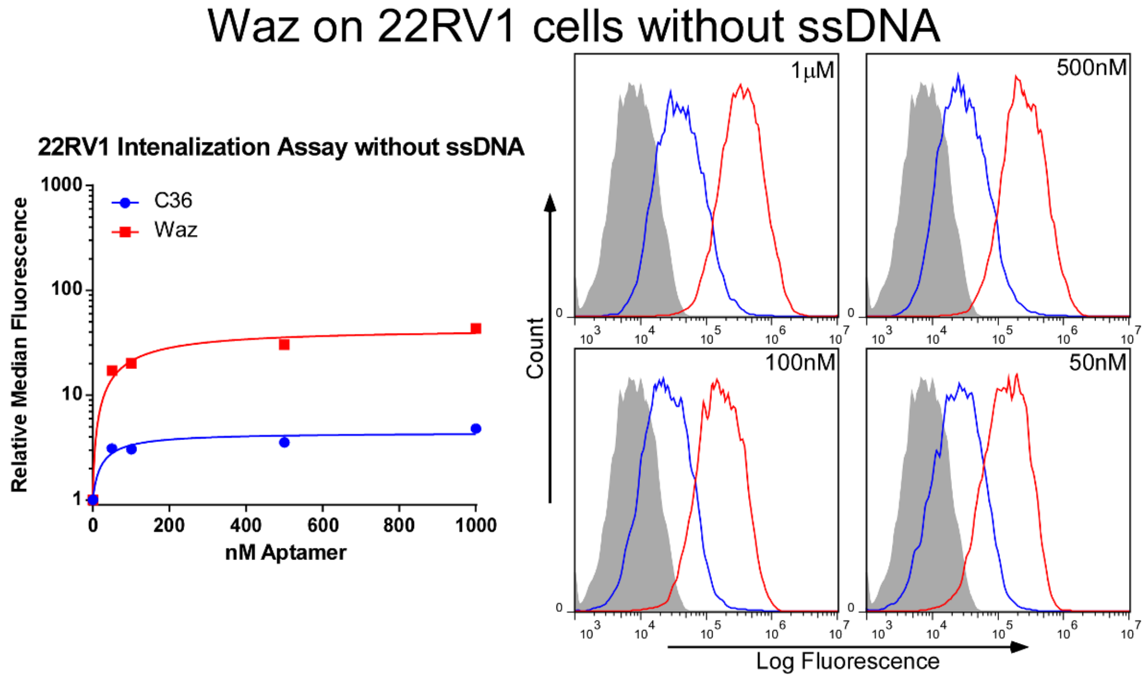

Supplementary Fig. 22. hTfR binding aptamer Waz internalization and binding assays on 22RV1 cells. Graphs represent the median fluorescence of the aptamer (Red) and C36 (Blue) relative to unstained cells (Gray).

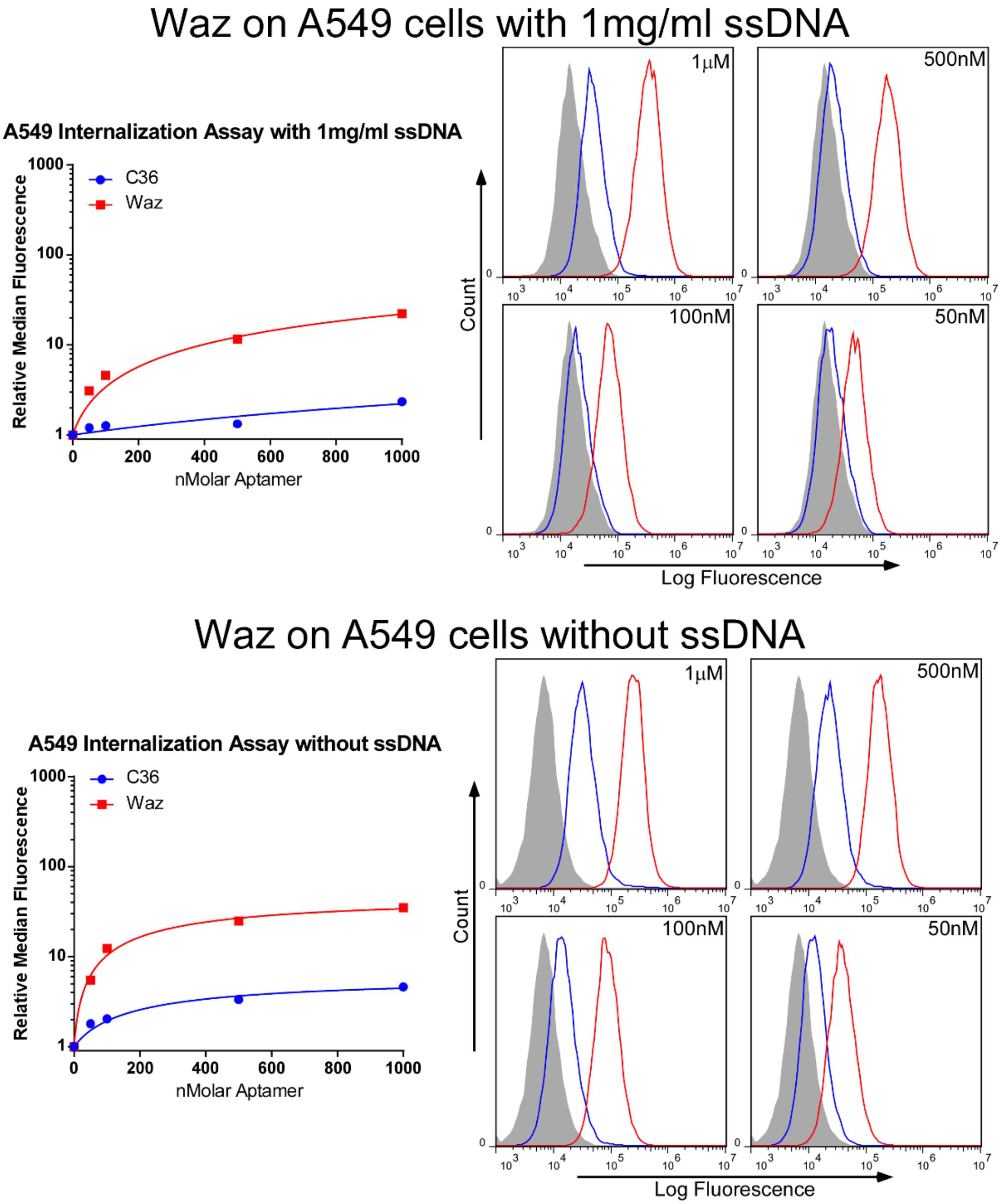

Supplementary Fig. 23. hTfR binding aptamer Waz internalization and binding assays on A549 cells. Graphs represent the median fluorescence of the aptamer (Red) and C36 (Blue) relative to unstained cells (Gray).

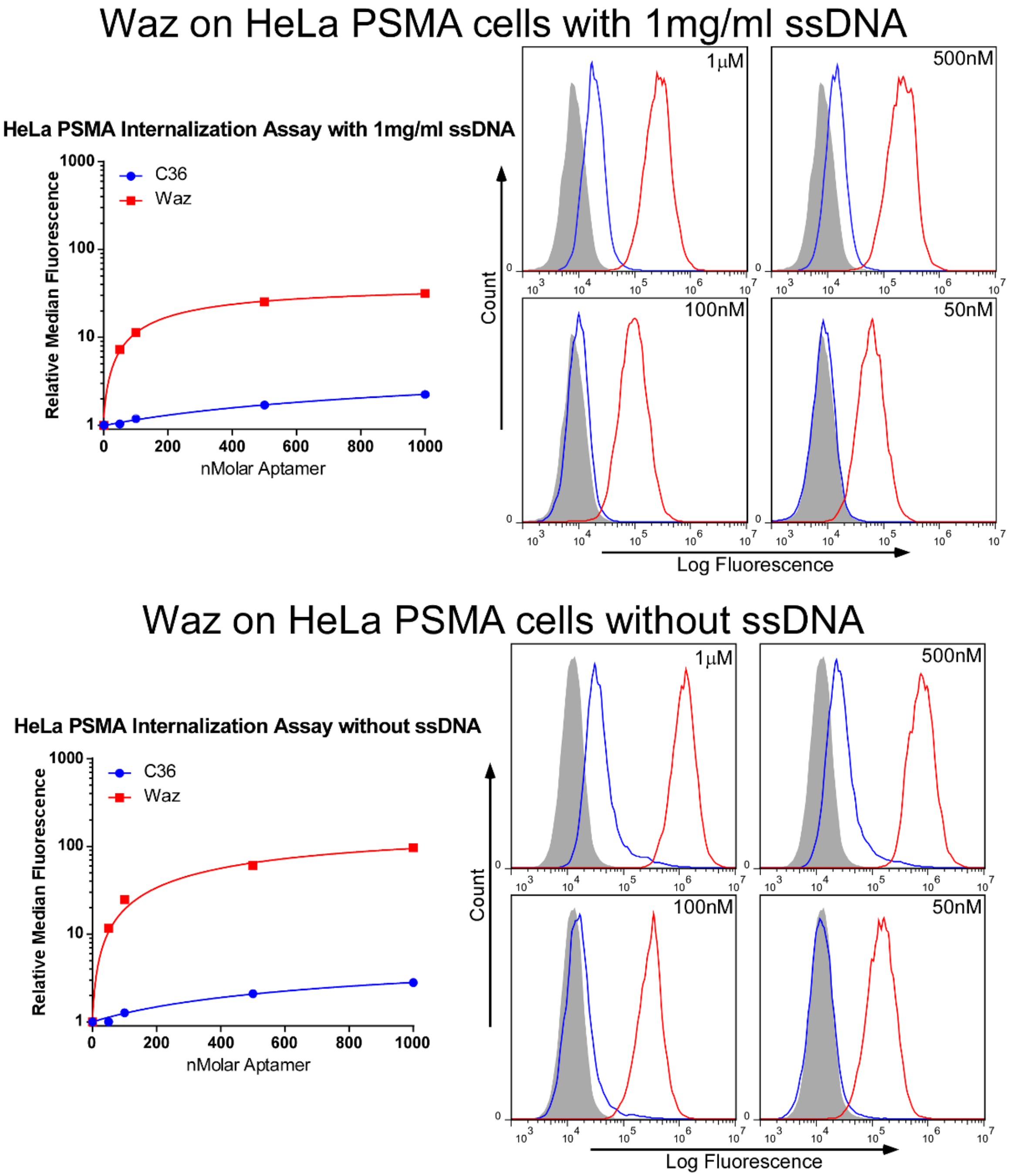

Supplementary Fig. 24. hTfR binding aptamer Waz internalization and binding assays on HeLa PSMA cells. Graphs represent the median fluorescence of the aptamer (Red) and C36 (Blue) relative to unstained cells (Gray).

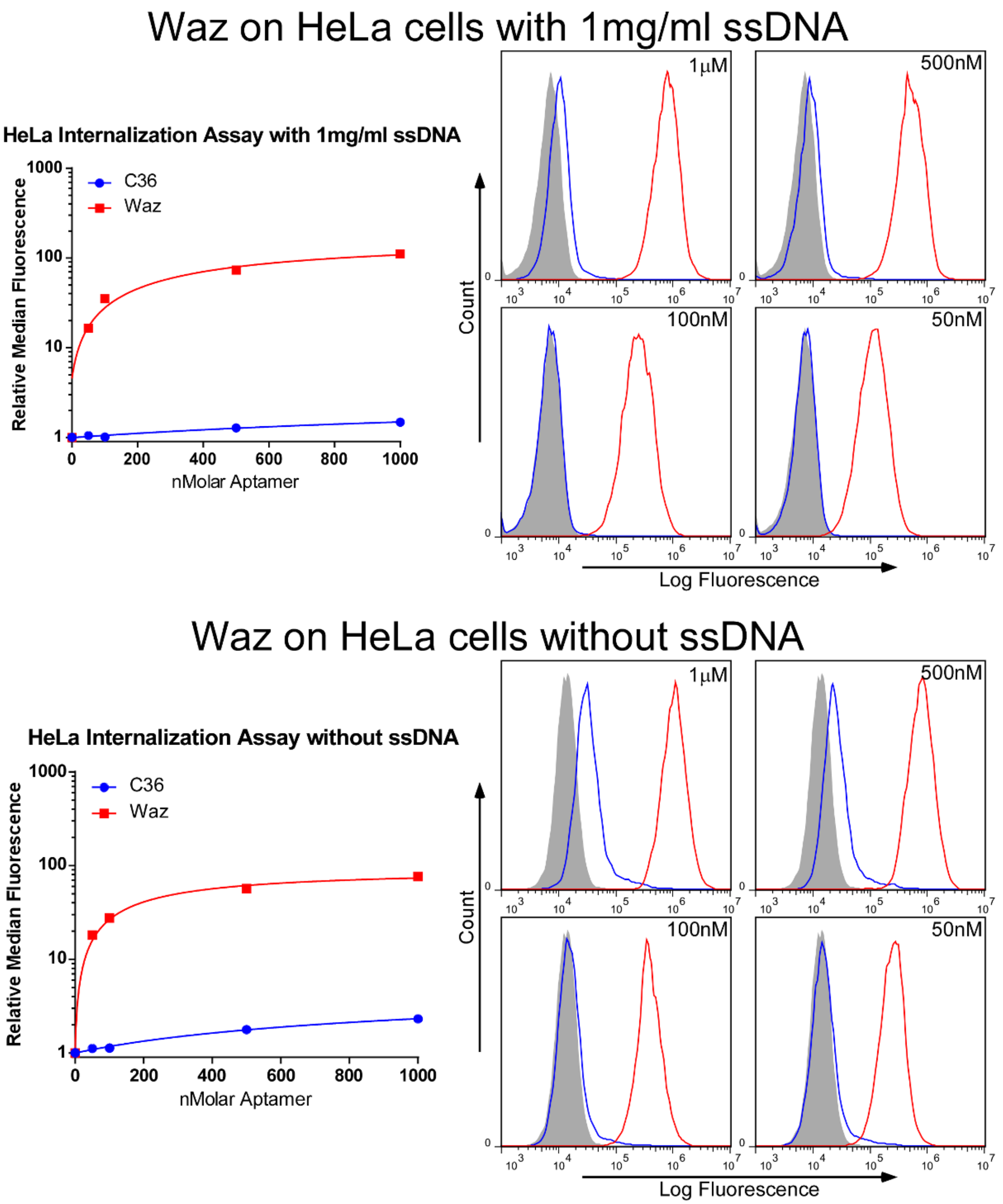

Supplementary Fig. 25. hTfR binding aptamer Waz internalization and binding assays on HeLa cells. Graphs represent the median fluorescence of the aptamer (Red) and C36 (Blue) relative to unstained cells (Gray).

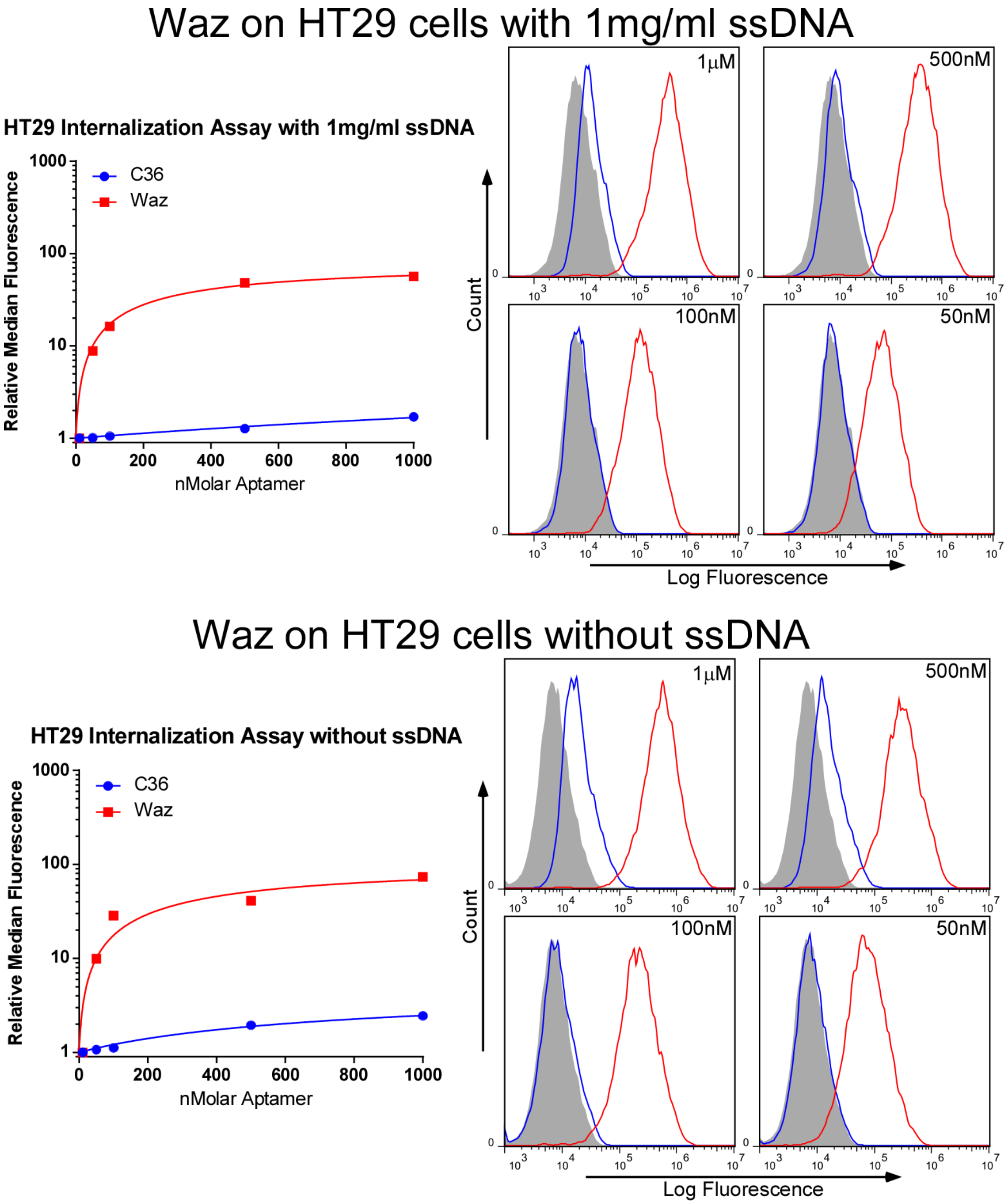

Supplementary Fig. 26. hTfR binding aptamer Waz internalization and binding assays on HT29 cells. Graphs represent the median fluorescence of the aptamer (Red) and C36 (Blue) relative to unstained cells (Gray).

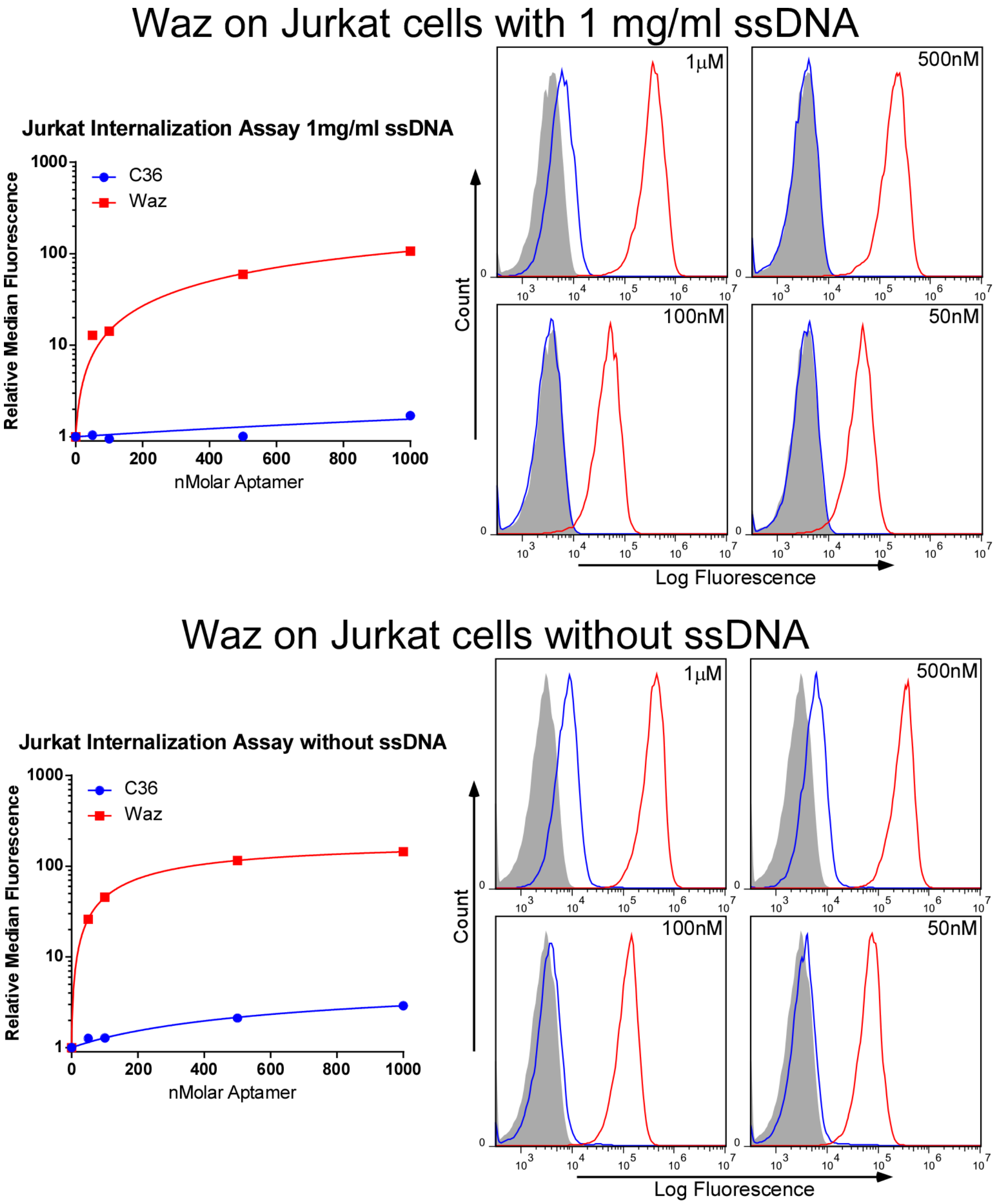

Supplementary Fig. 27. hTfR binding aptamer Waz internalization and binding assays on Jurkat cells. Graphs represent the median fluorescence of the aptamer (Red) and C36 (Blue) relative to unstained cells (Gray).

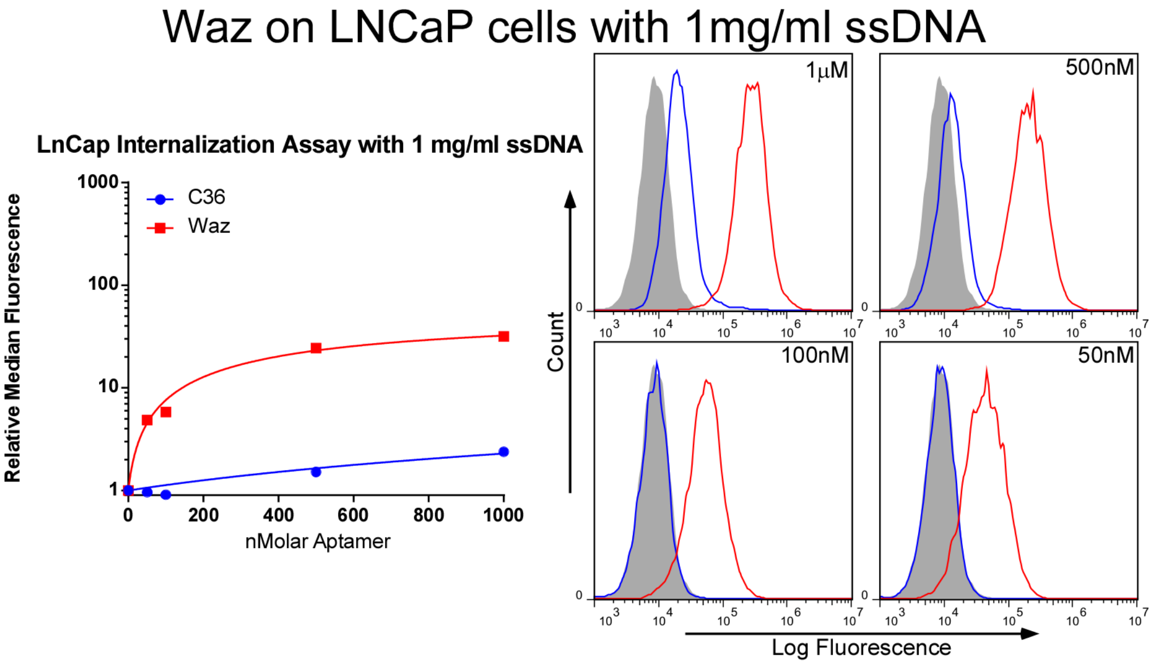

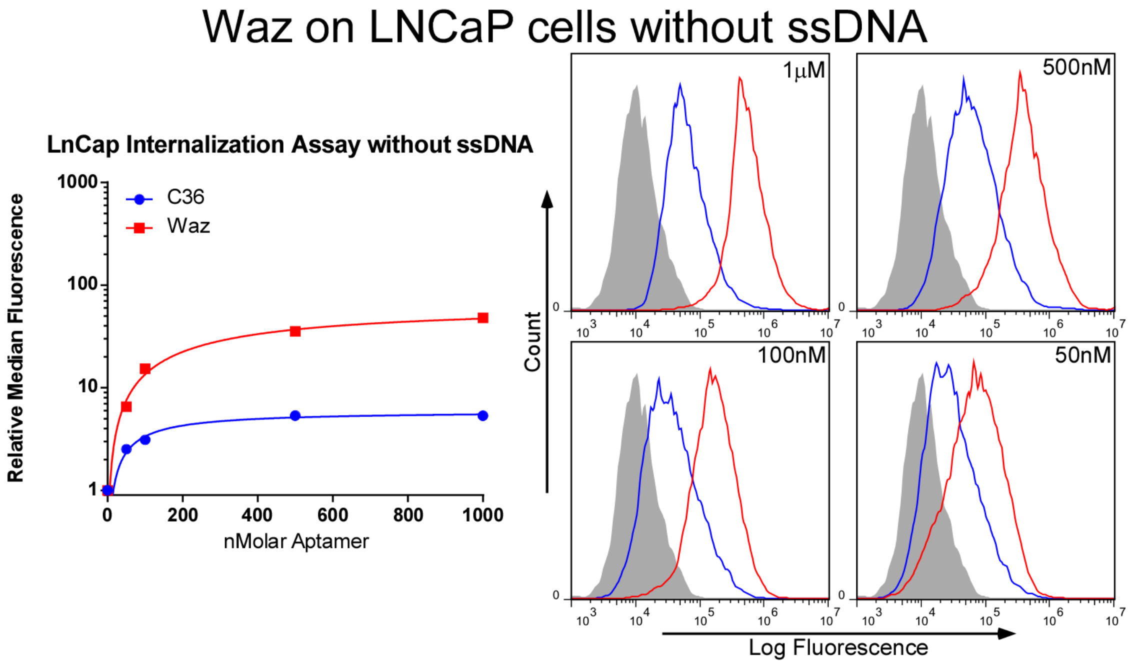

Supplementary Fig. 28. hTfR binding aptamer Waz internalization and binding assays on LNCaP cells. Graphs represent the median fluorescence of the aptamer (Red) and C36 (Blue) relative to unstained cells (Gray).

Waz on MCF7 cells with 1mg/ml ssDNA

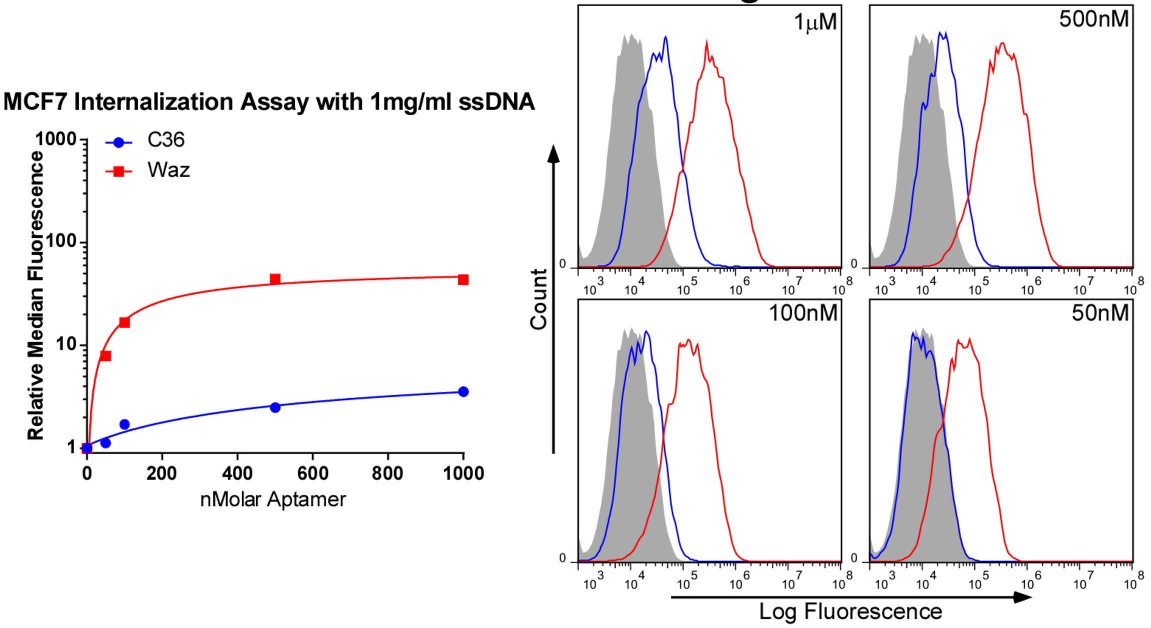

Waz on MCF7 cells without ssDNA

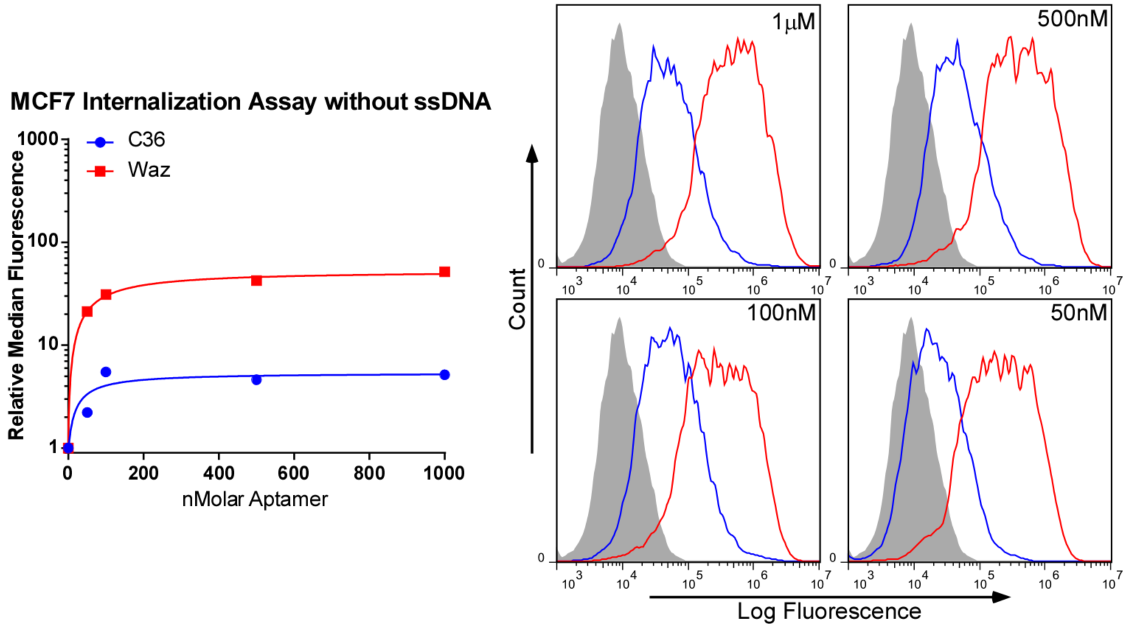

Supplementary Fig. 29. hTfR binding aptamer Waz internalization and binding assays on MCF7 cells. Graphs represent the median fluorescence of the aptamer (Red) and C36 (Blue) relative to unstained cells (Gray).

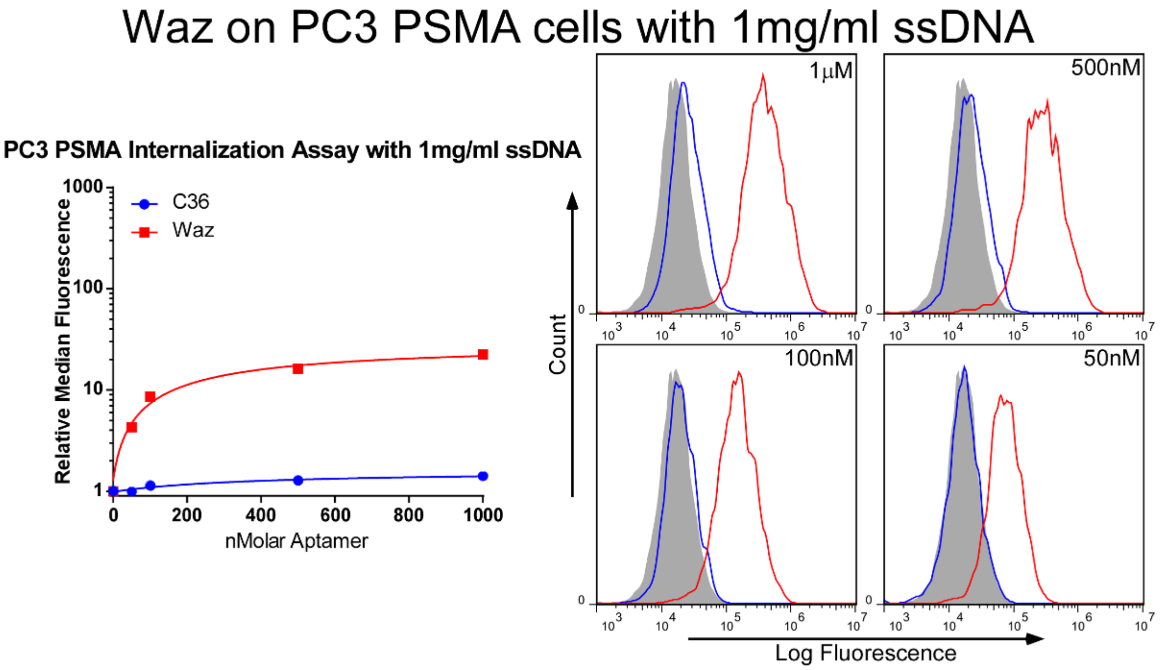

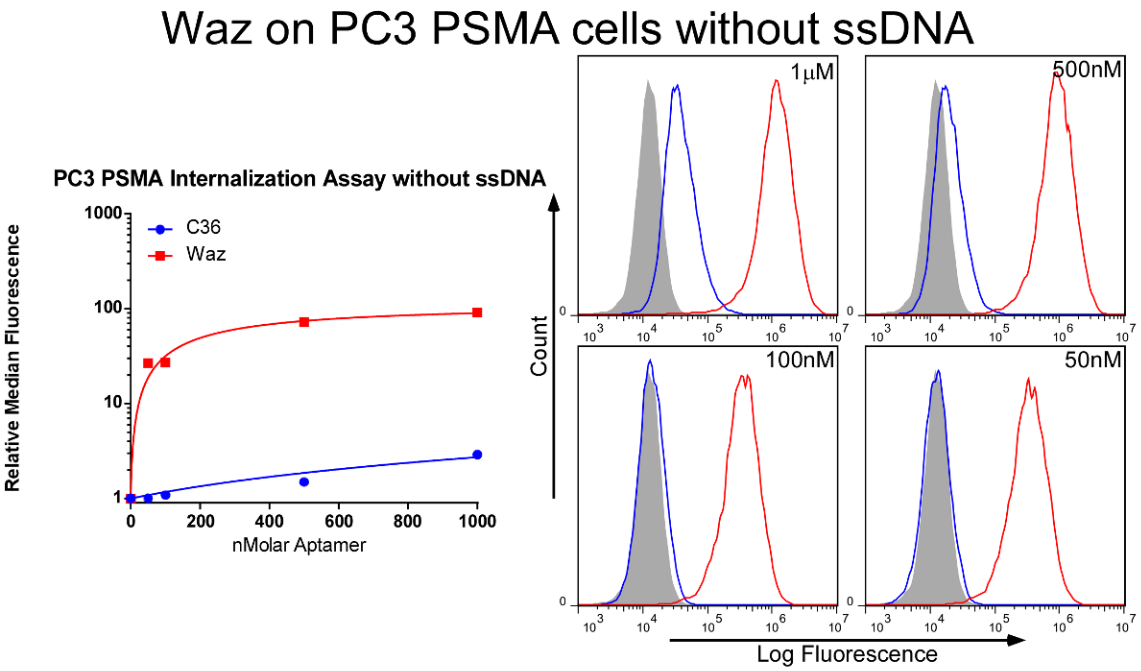

Supplementary Fig. 30. hTfR binding aptamer Waz internalization and binding assays on PC3 PSMA cells. Graphs represent the median fluorescence of the aptamer (Red) and C36 (Blue) relative to unstained cells (Gray).

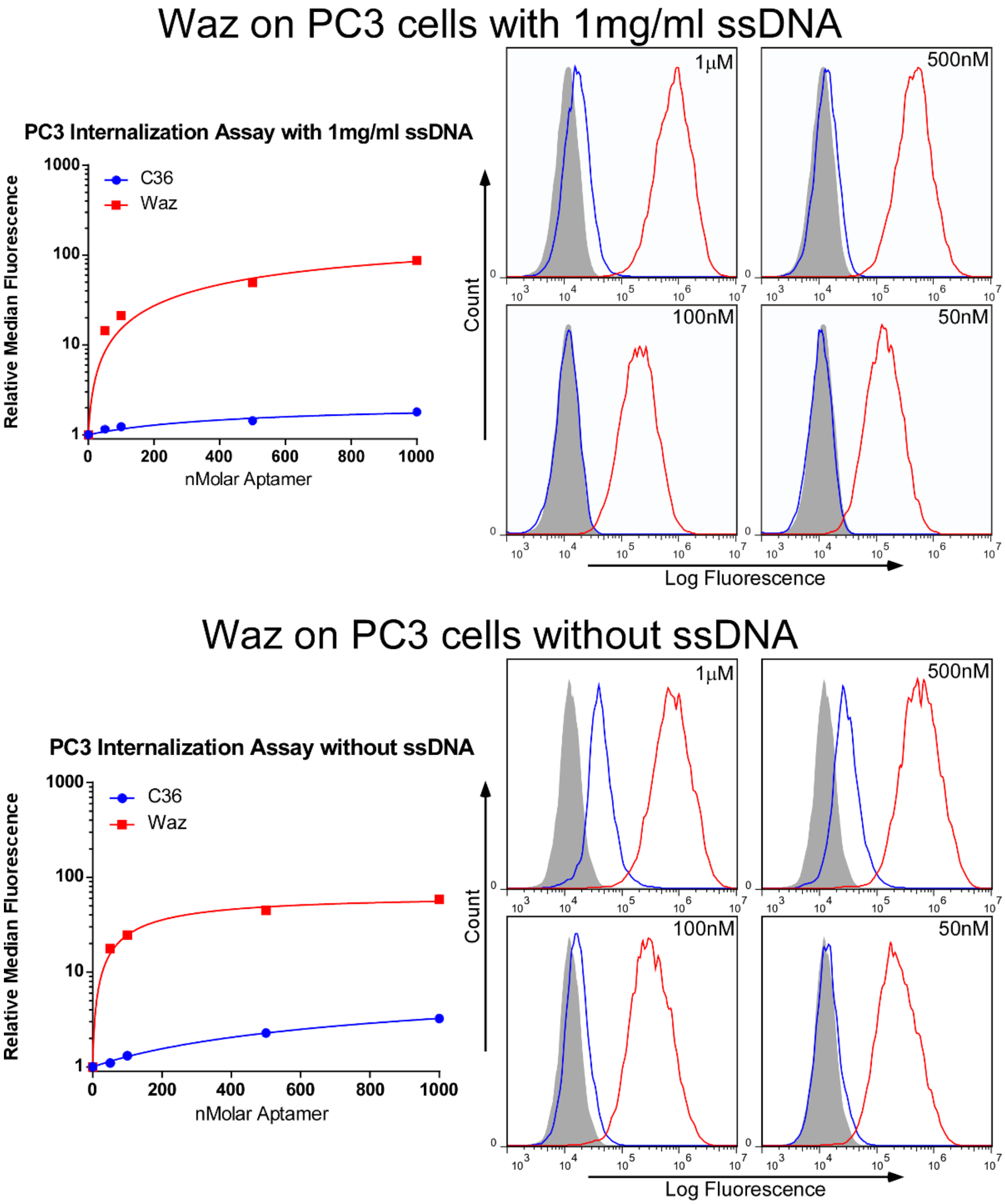

Supplementary Fig. 31. hTfR binding aptamer Waz internalization and binding assays on PC3 cells. Graphs represent the median fluorescence of the aptamer (Red) and C36 (Blue) relative to unstained cells (Gray).

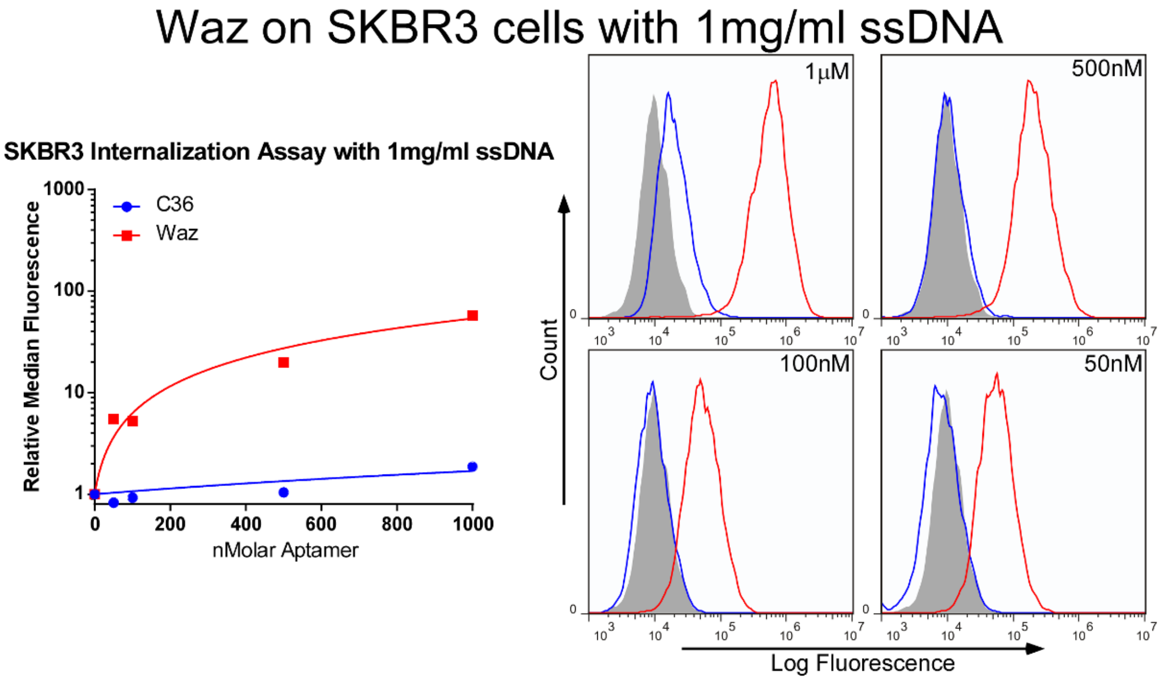

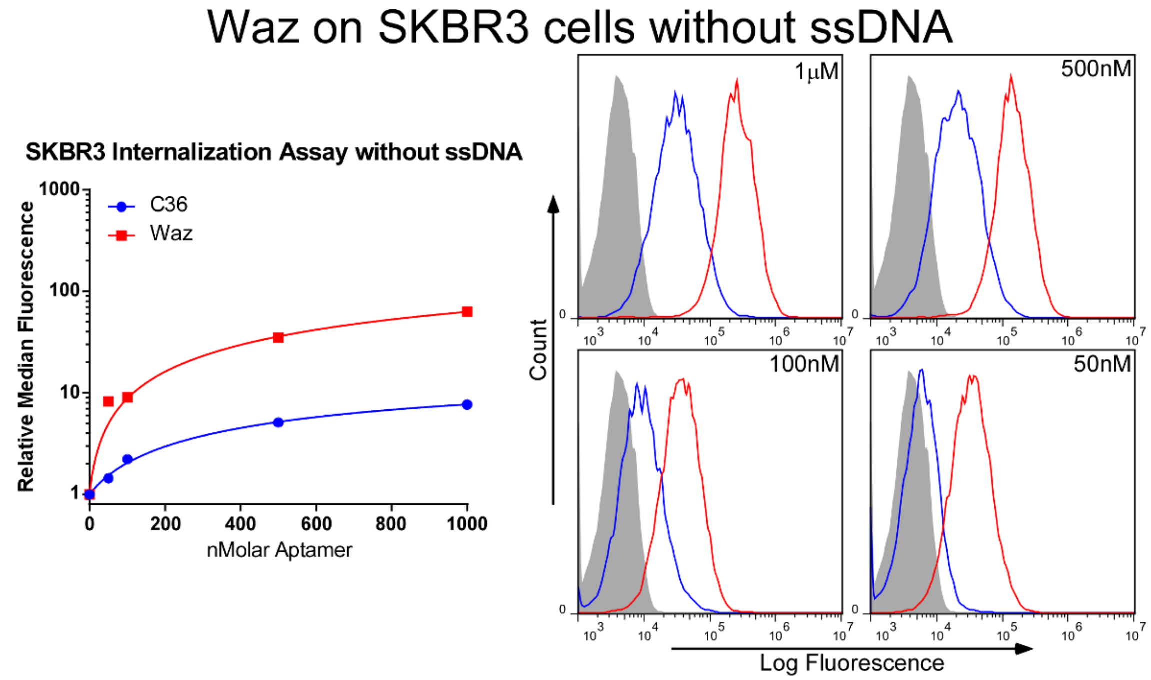

Supplementary Fig. 32. hTfR binding aptamer Waz internalization and binding assays on SKBR3 cells. Graphs represent the median fluorescence of the aptamer (Red) and C36 (Blue) relative to unstained cells (Gray).

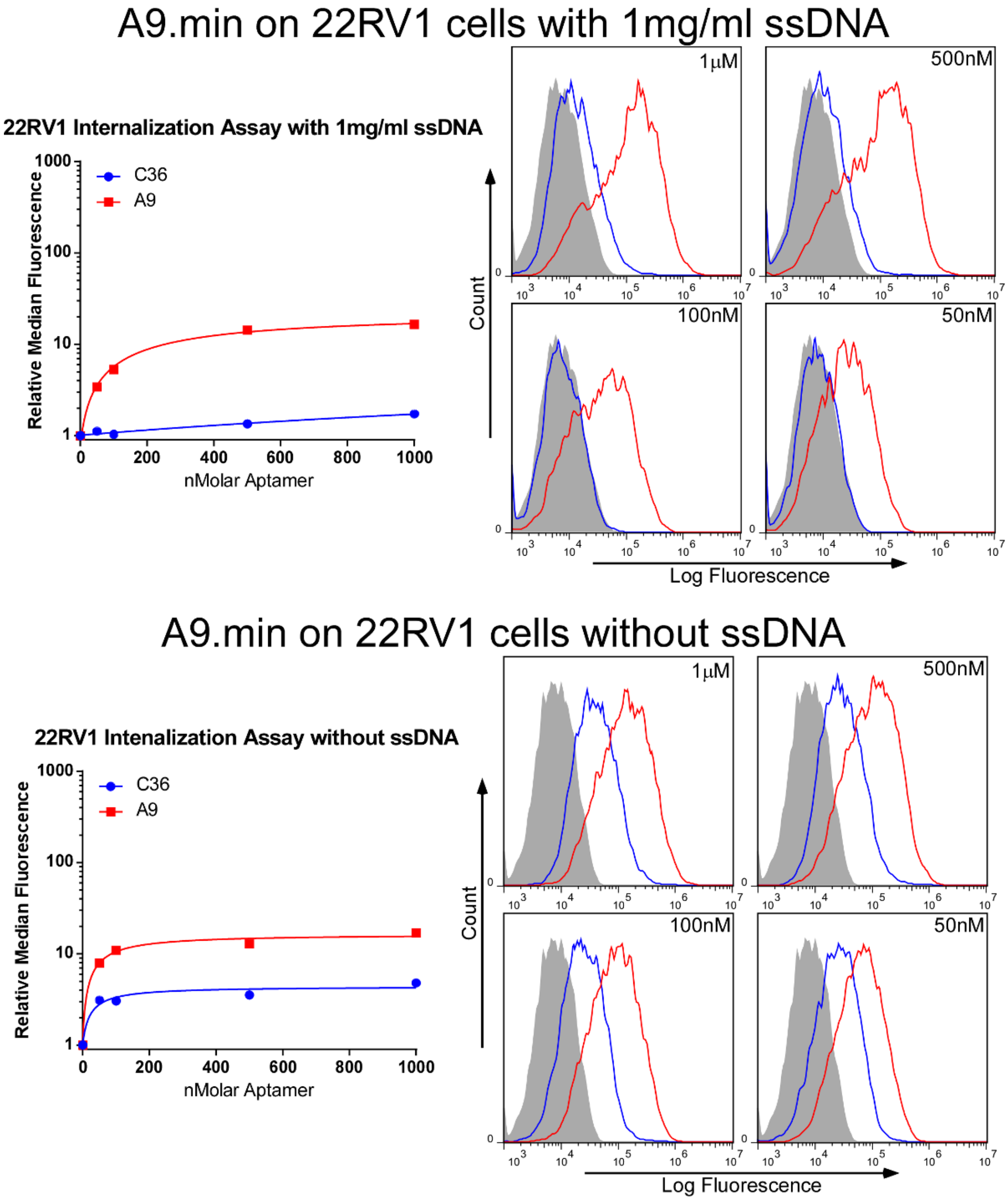

Supplementary Fig. 33. PSMA binding aptamer A9.min internalization and binding assays on 22RV1 cells. Graphs represent the median fluorescence of the aptamer (Red) and C36 (Blue) relative to unstained cells (Gray).

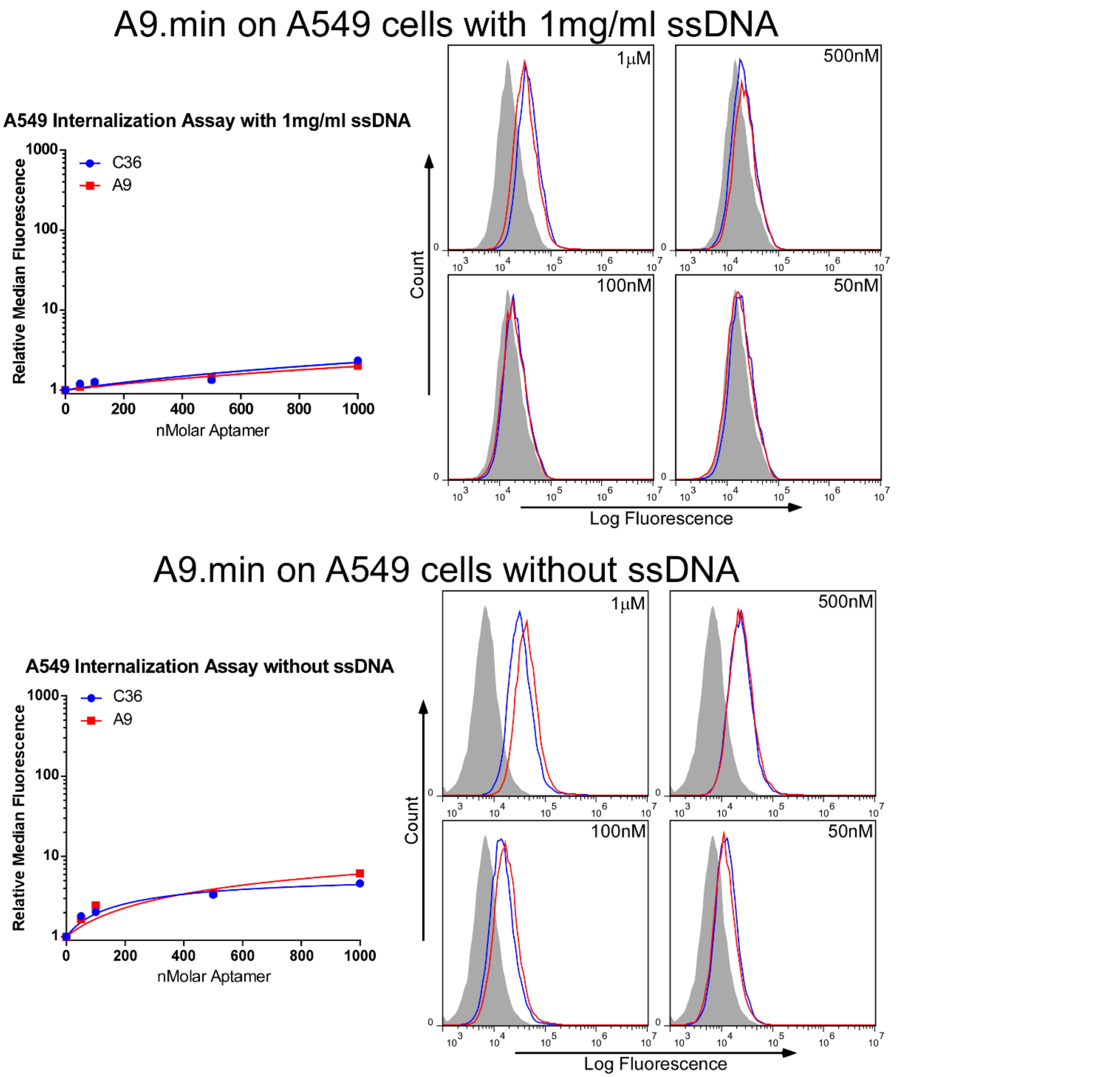

Supplementary Fig. 34. PSMA binding aptamer A9.min internalization and binding assays on A549 cells. Graphs represent the median fluorescence of the aptamer (Red) and C36 (Blue) relative to unstained cells (Gray).

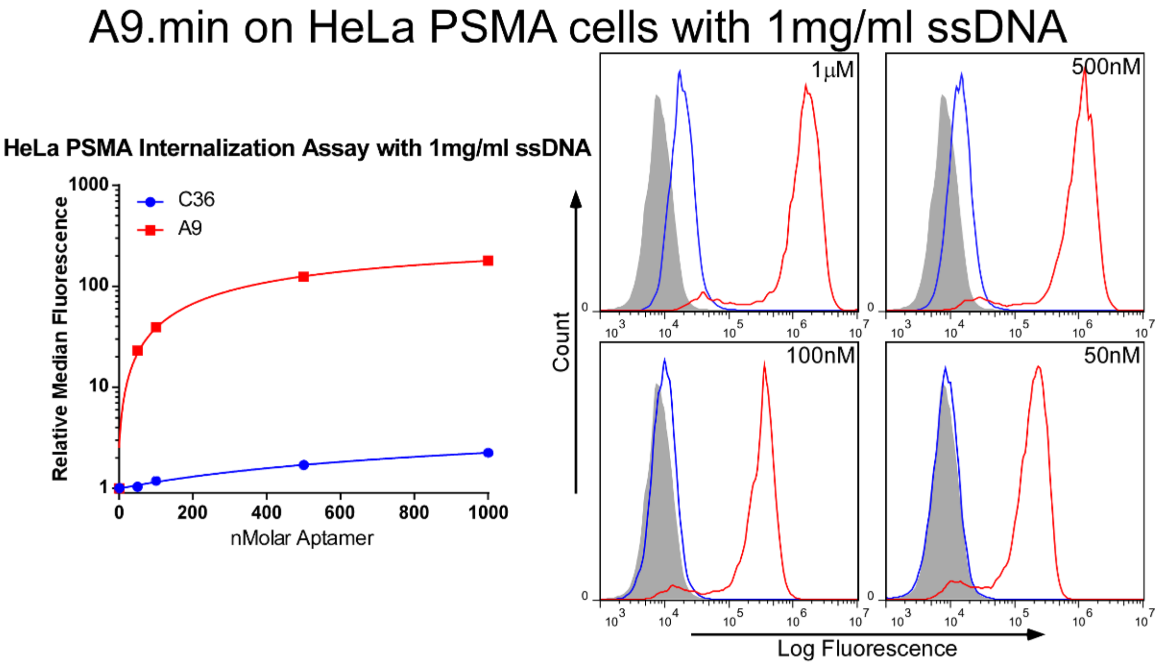

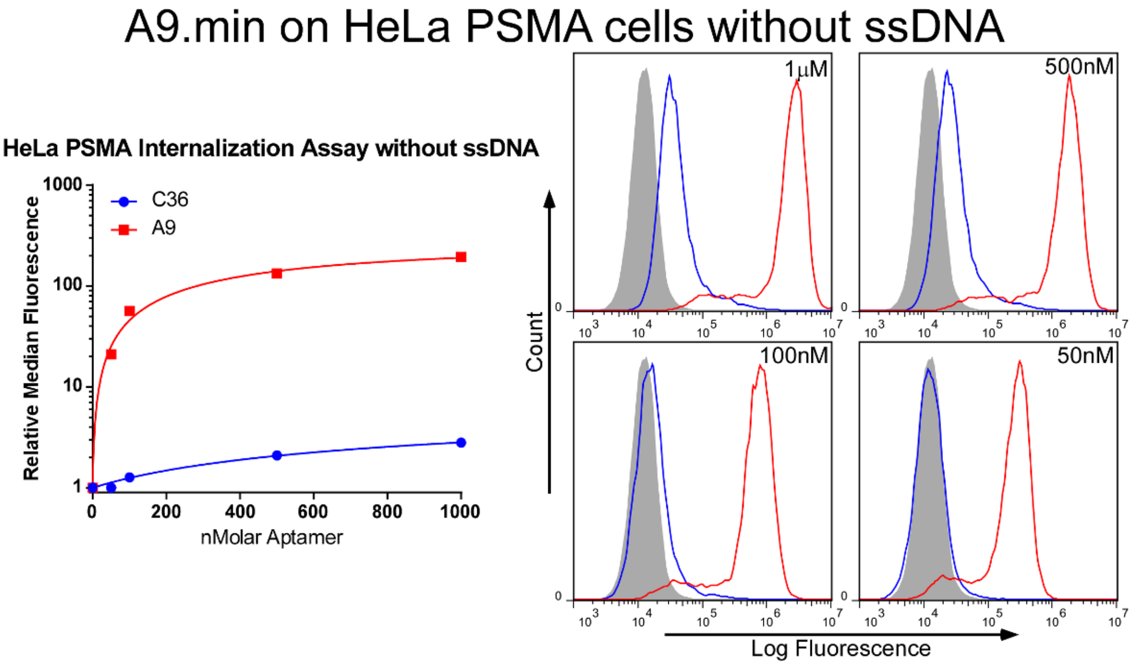

Supplementary Fig. 35. PSMA binding aptamer A9.min internalization and binding assays on HeLa PSMA cells. Graphs represent the median fluorescence of the aptamer (Red) and C36 (Blue) relative to unstained cells (Gray).

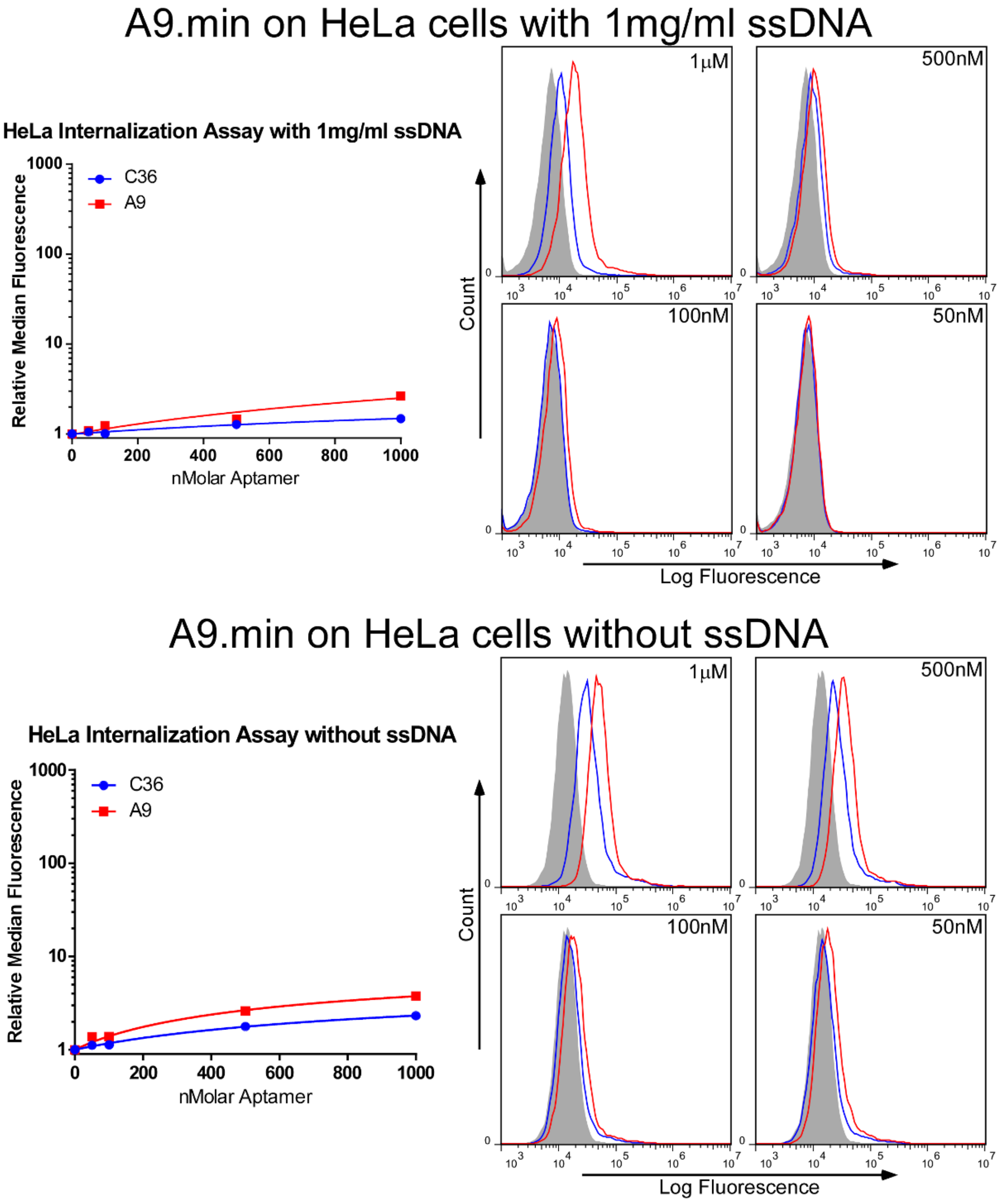

Supplementary Fig. 36. PSMA binding aptamer A9.min internalization and binding assays on HeLa cells. Graphs represent the median fluorescence of the aptamer (Red) and C36 (Blue) relative to unstained cells (Gray).

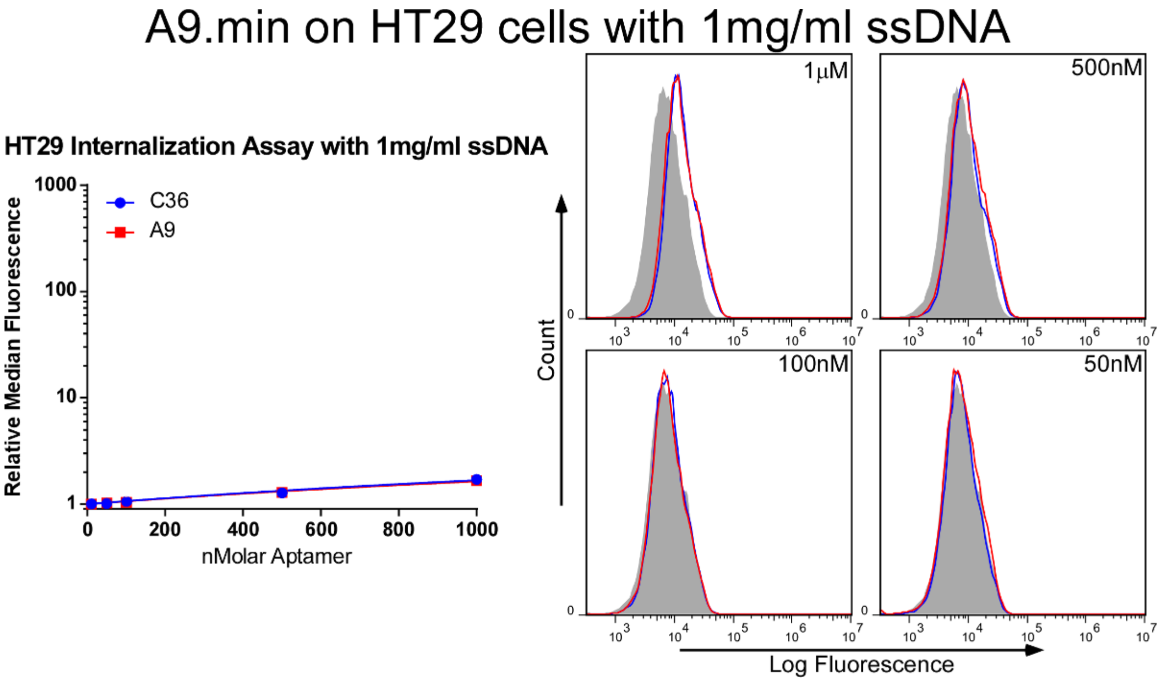

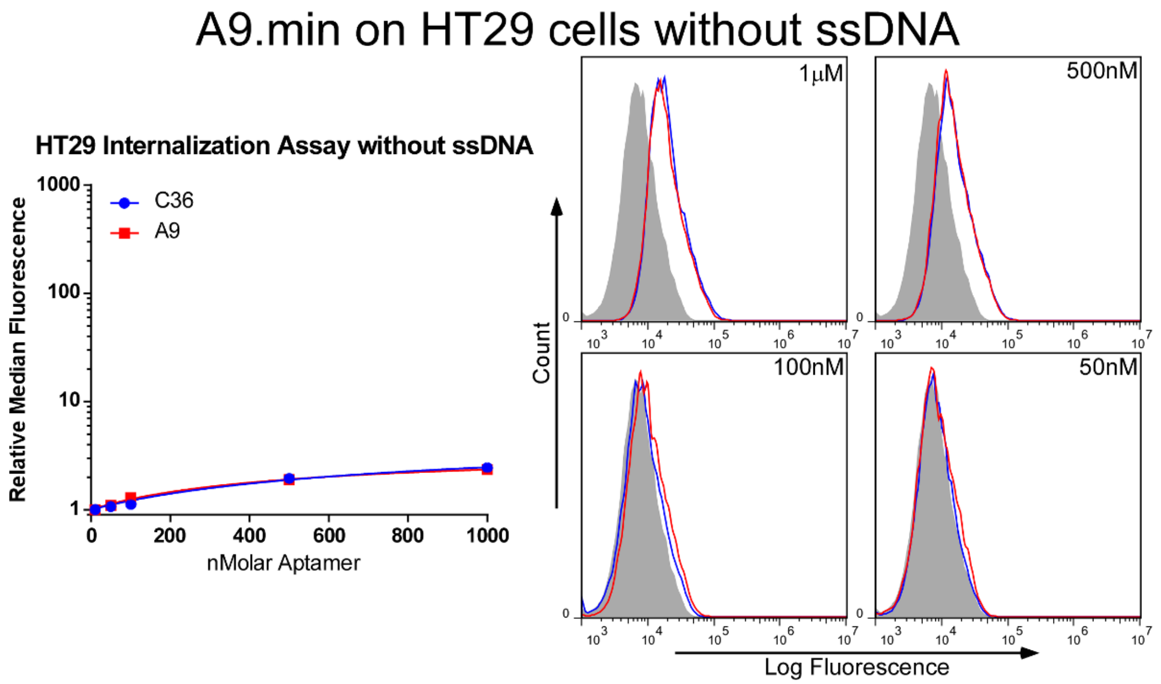

Supplementary Fig. 37. PSMA binding aptamer A9.min internalization and binding assays on HT29 cells. Graphs represent the median fluorescence of the aptamer (Red) and C36 (Blue) relative to unstained cells (Gray).

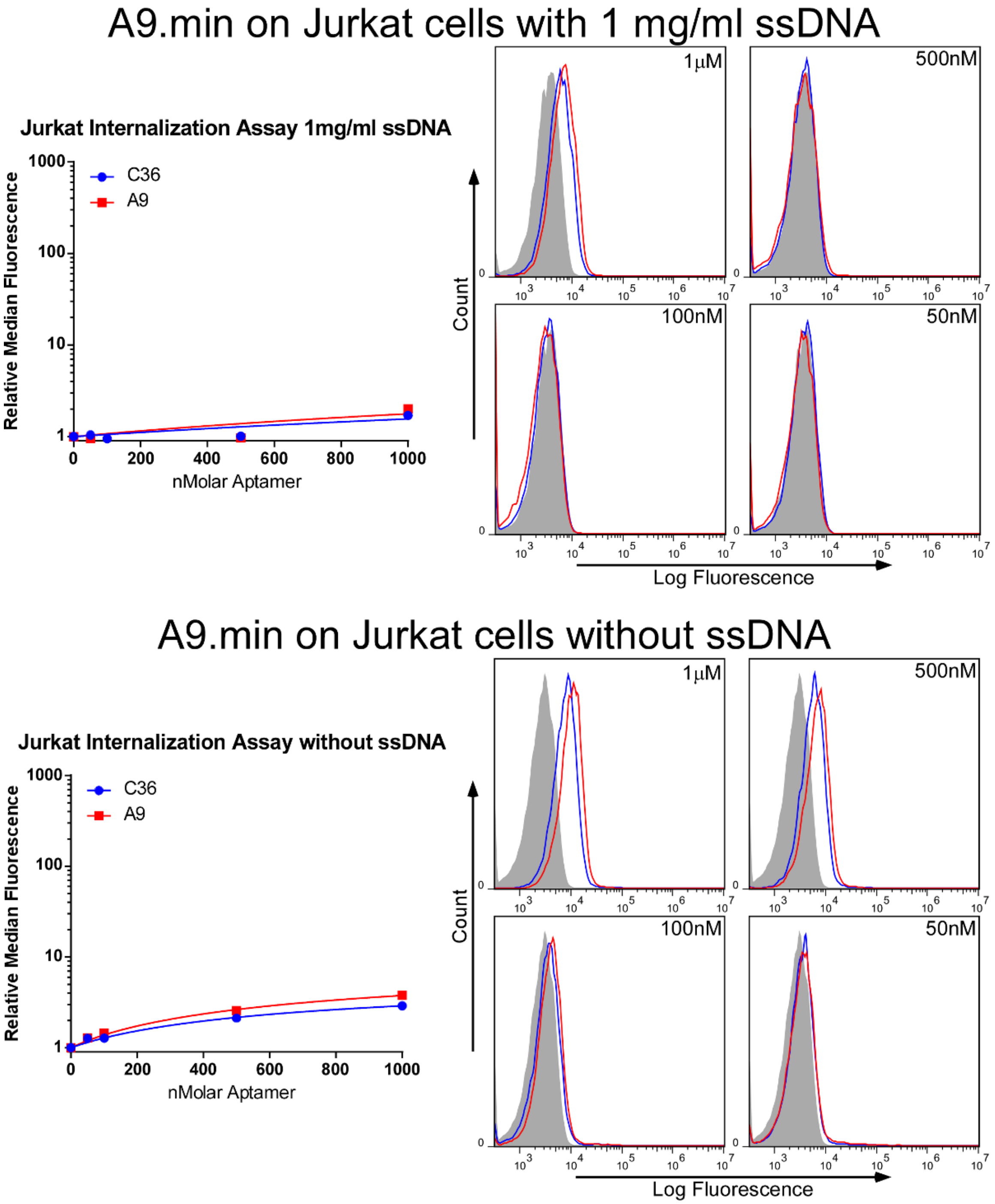

Supplementary Fig. 38. PSMA binding aptamer A9.min internalization and binding assays on Jurkat cells. Graphs represent the median fluorescence of the aptamer (Red) and C36 (Blue) relative to unstained cells (Gray).

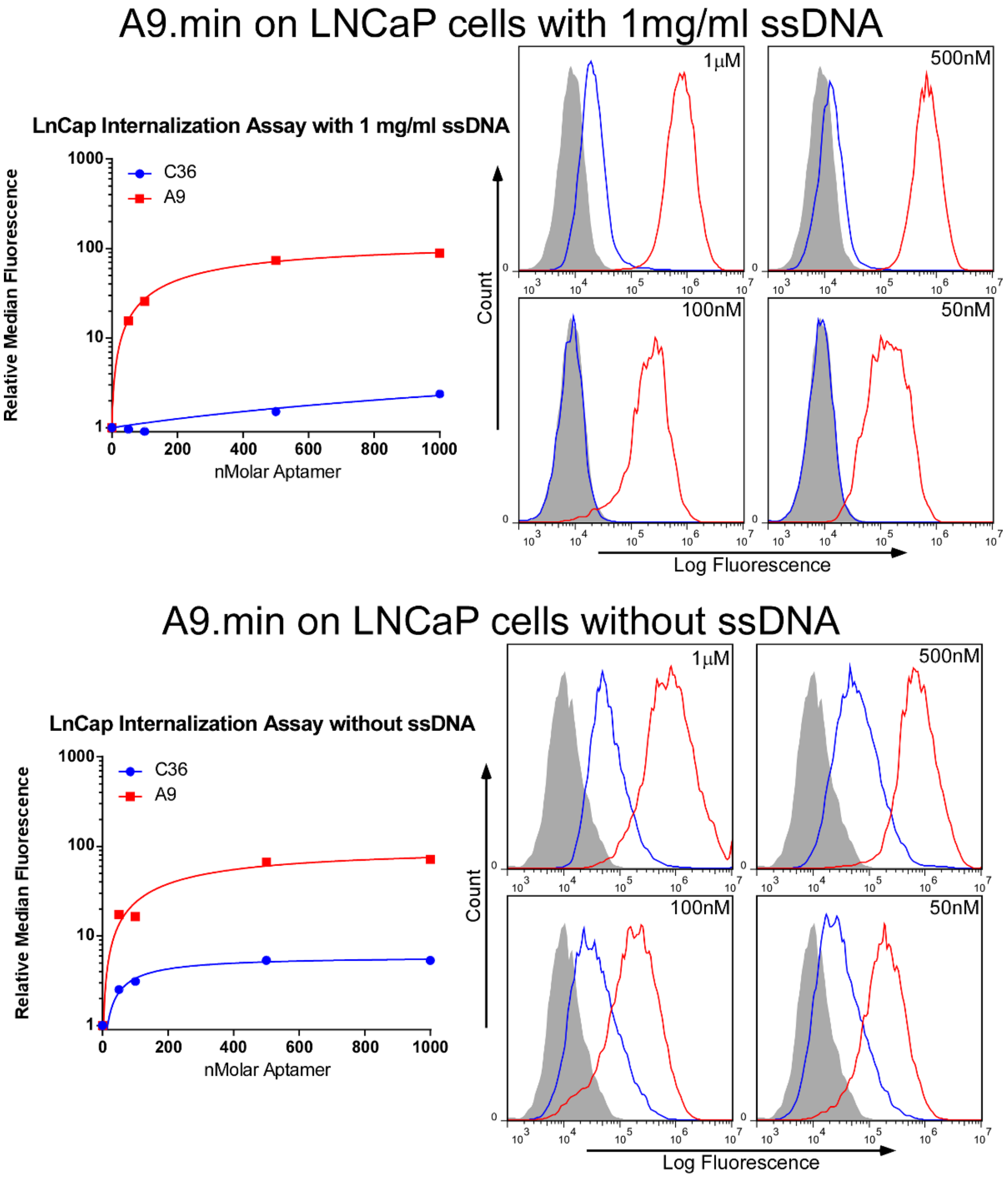

Supplementary Fig. 39. PSMA binding aptamer A9.min internalization and binding assays on LNCaP cells. Graphs represent the median fluorescence of the aptamer (Red) and C36 (Blue) relative to unstained cells (Gray).

A9.min on MCF7 cells with 1mg/ml ssDNA

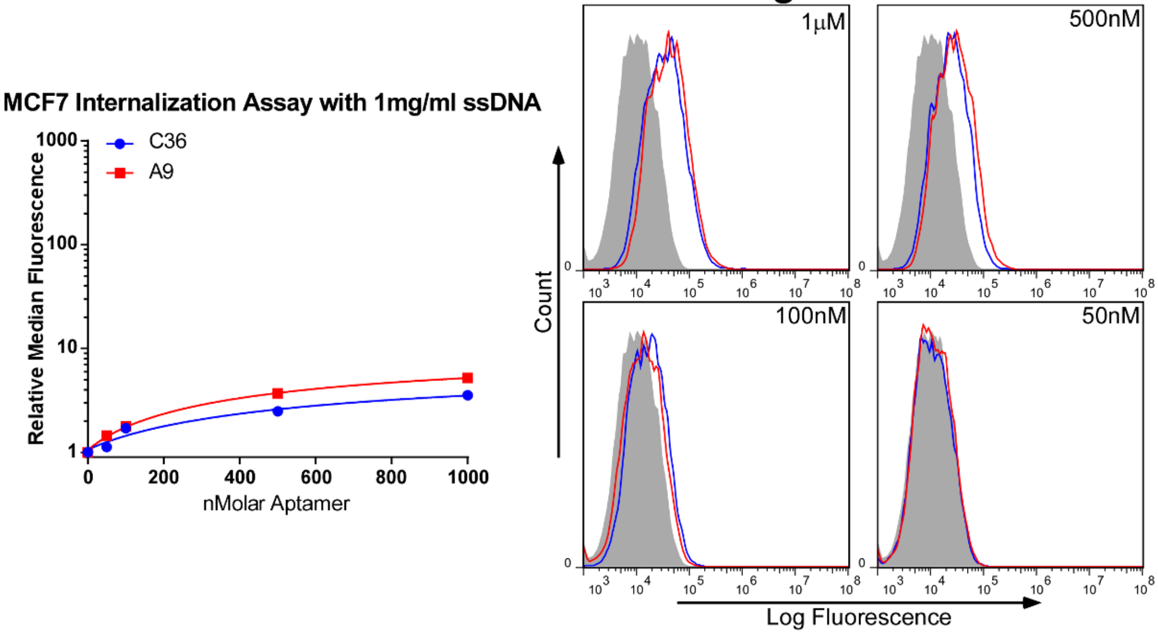

A9.min on MCF7 cells without ssDNA

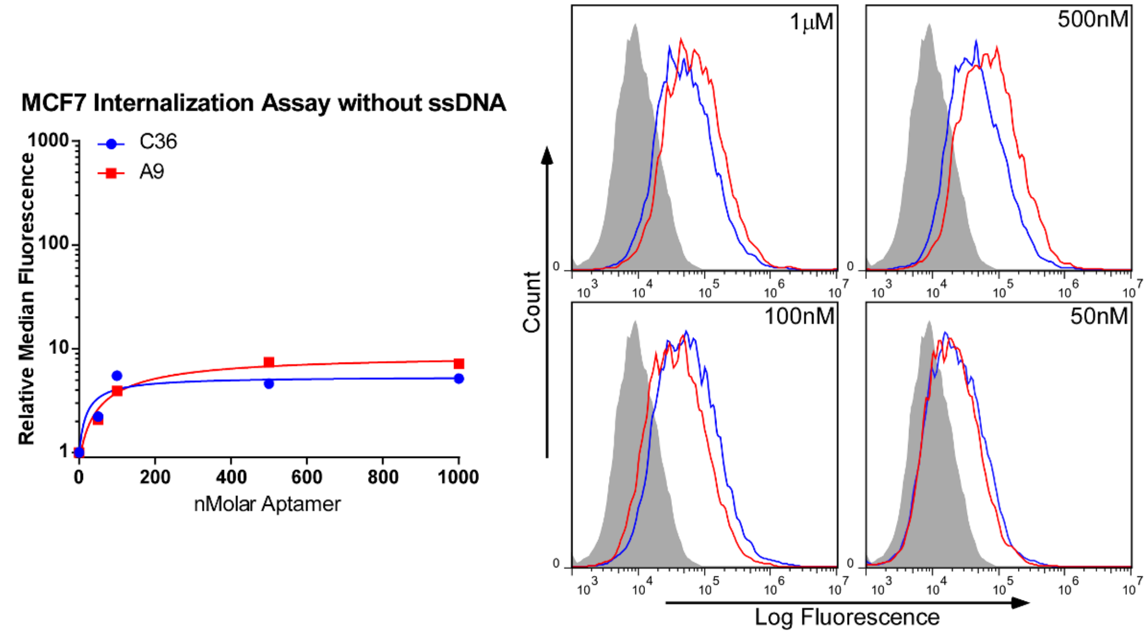

Supplementary Fig. 40. PSMA binding aptamer A9.min internalization and binding assays on MCF7 cells. Graphs represent the median fluorescence of the aptamer (Red) and C36 (Blue) relative to unstained cells (Gray).

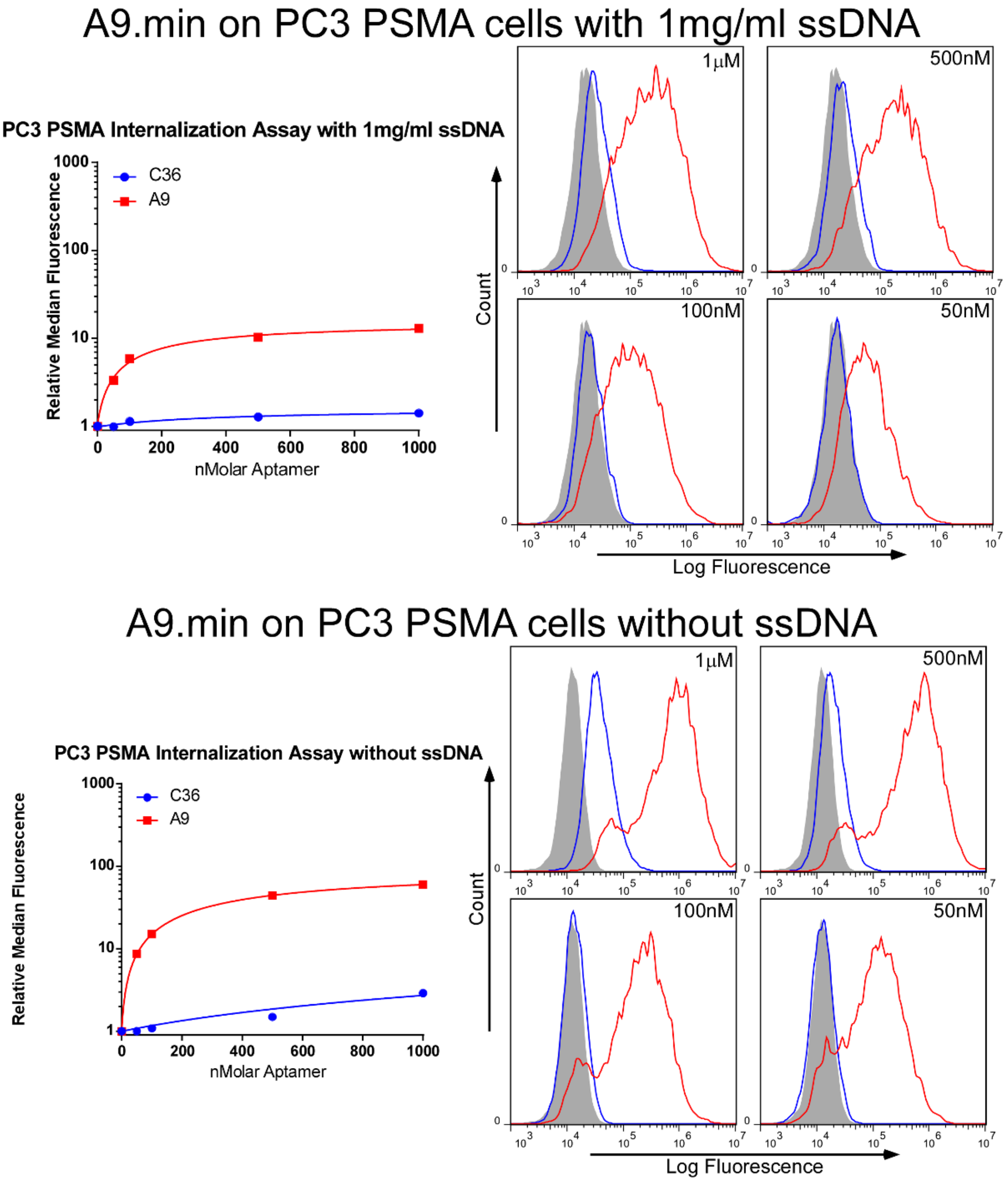

Supplementary Fig. 41. PSMA binding aptamer A9.min internalization and binding assays on PC3 PSMA cells. Graphs represent the median fluorescence of the aptamer (Red) and C36 (Blue) relative to unstained cells (Gray).

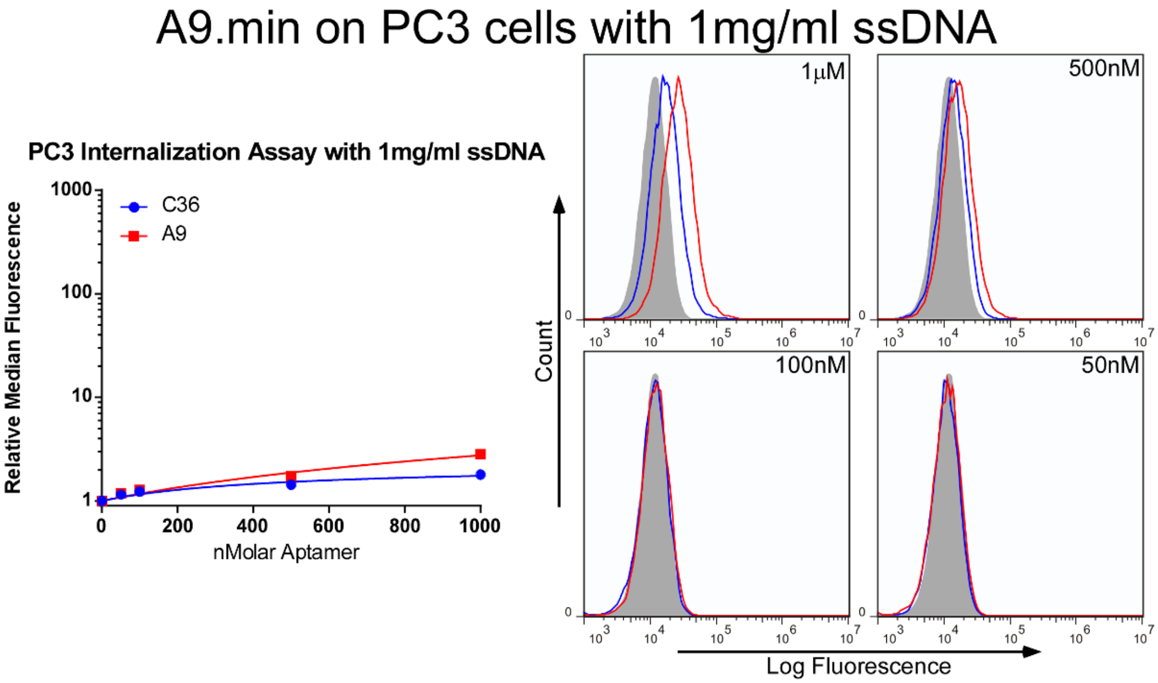

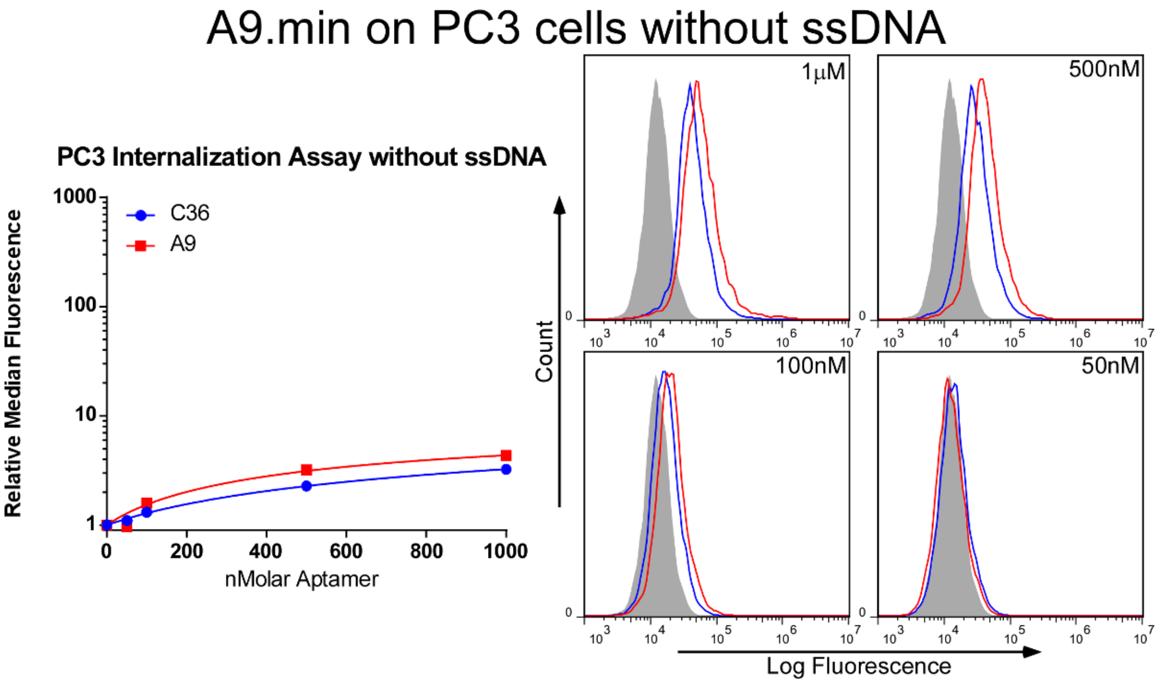

Supplementary Fig. 42. PSMA binding aptamer A9.min internalization and binding assays on PC3 cells. Graphs represent the median fluorescence of the aptamer (Red) and C36 (Blue) relative to unstained cells (Gray).

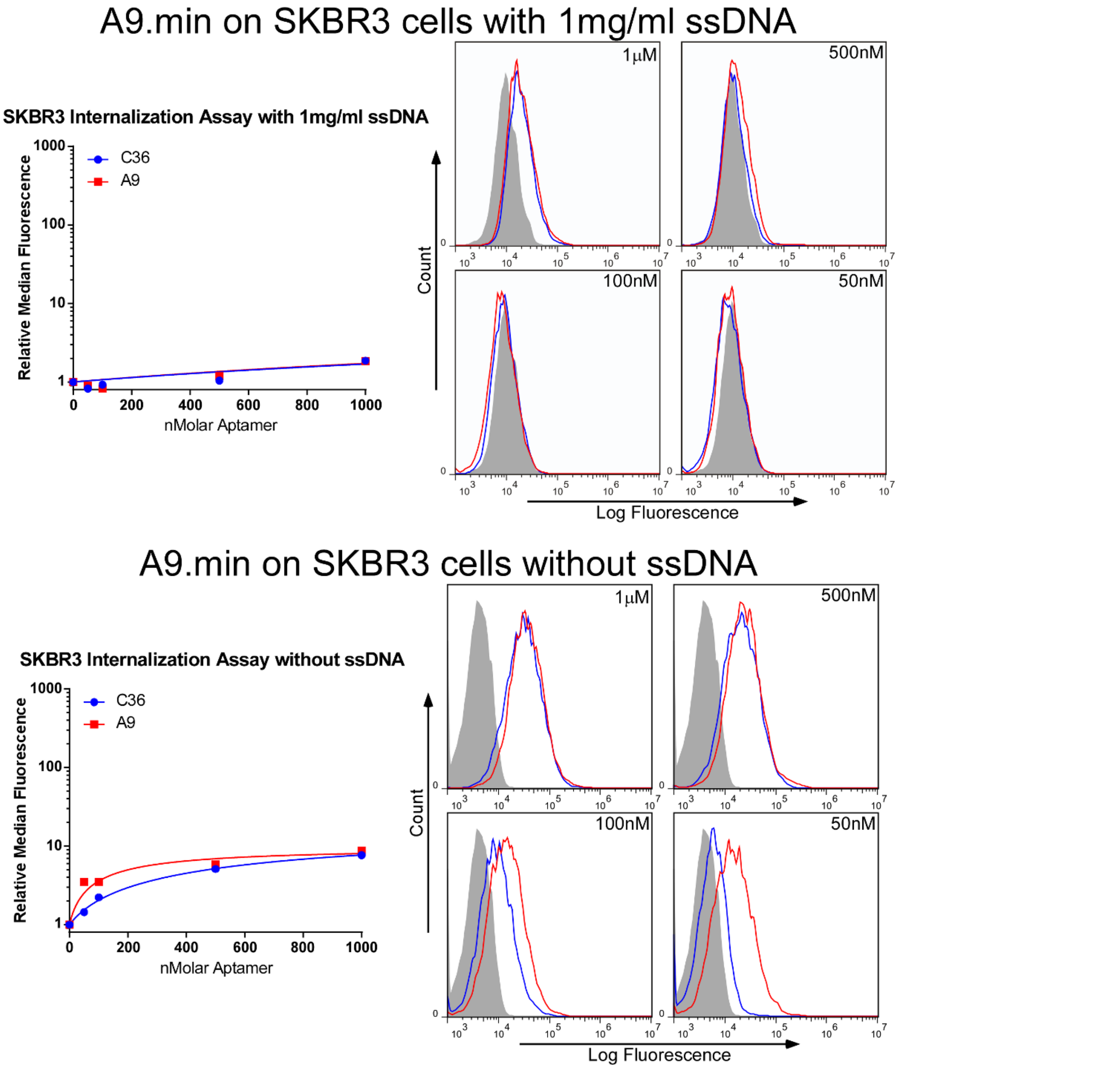

Supplementary Fig. 43. PSMA binding aptamer A9.min internalization and binding assays on SKBR3 cells. Graphs represent the median fluorescence of the aptamer (Red) and C36 (Blue) relative to unstained cells (Gray).

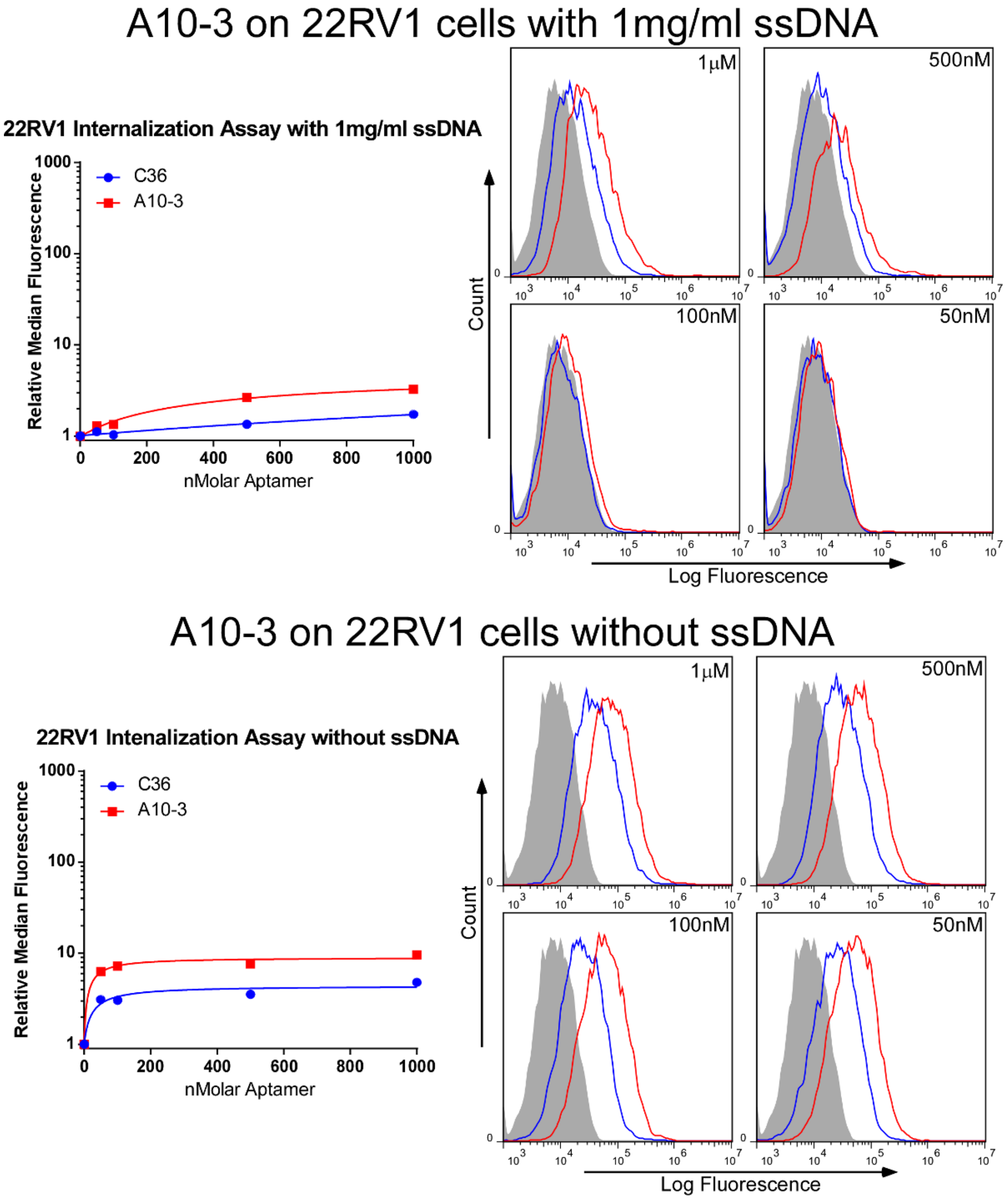

Supplementary Fig. 44. PSMA targeted aptamer A10-3 internalization and binding assays on 22RV1 cells. Graphs represent the median fluorescence of the aptamer (Red) and C36 (Blue) relative to unstained cells (Gray).

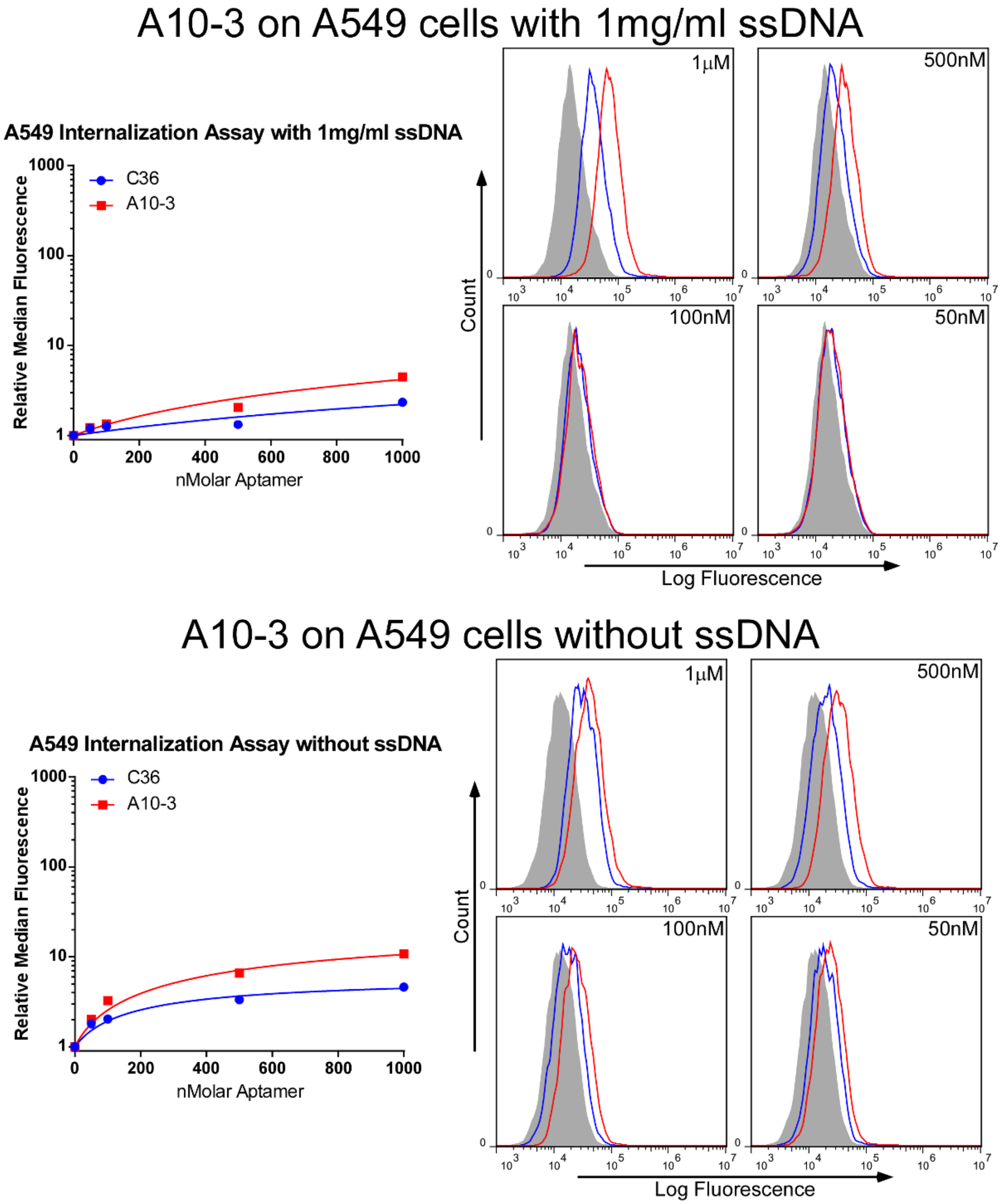

Supplementary Fig. 45. PSMA targeted aptamer A10-3 internalization and binding assays on A549 cells. Graphs represent the median fluorescence of the aptamer (Red) and C36 (Blue) relative to unstained cells (Gray).

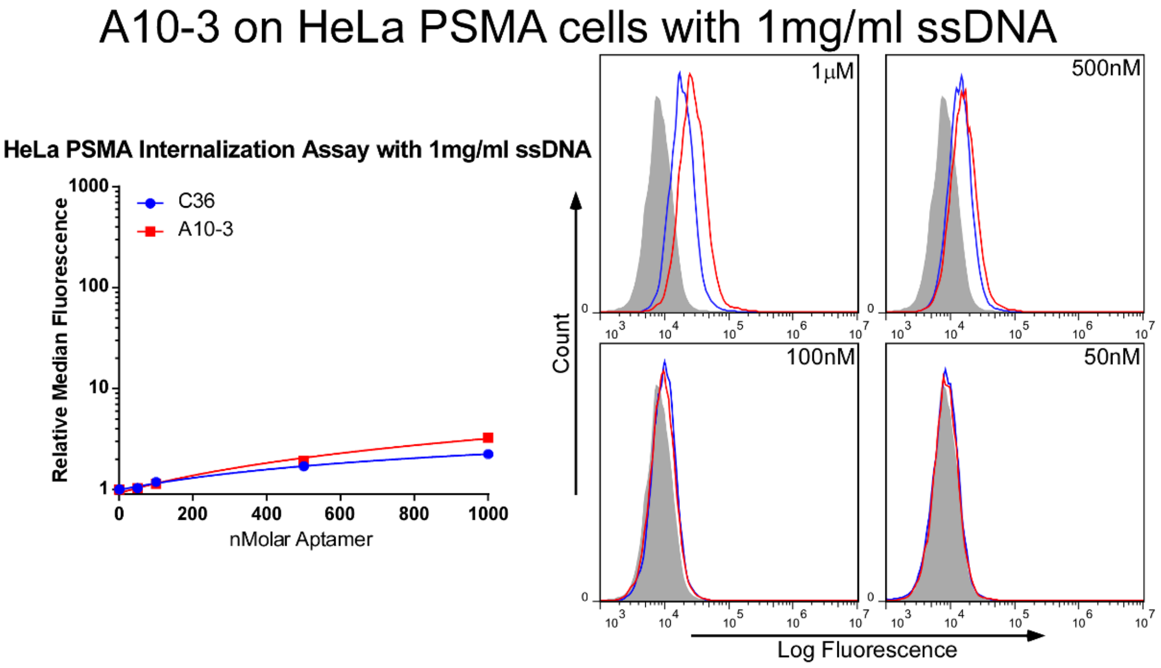

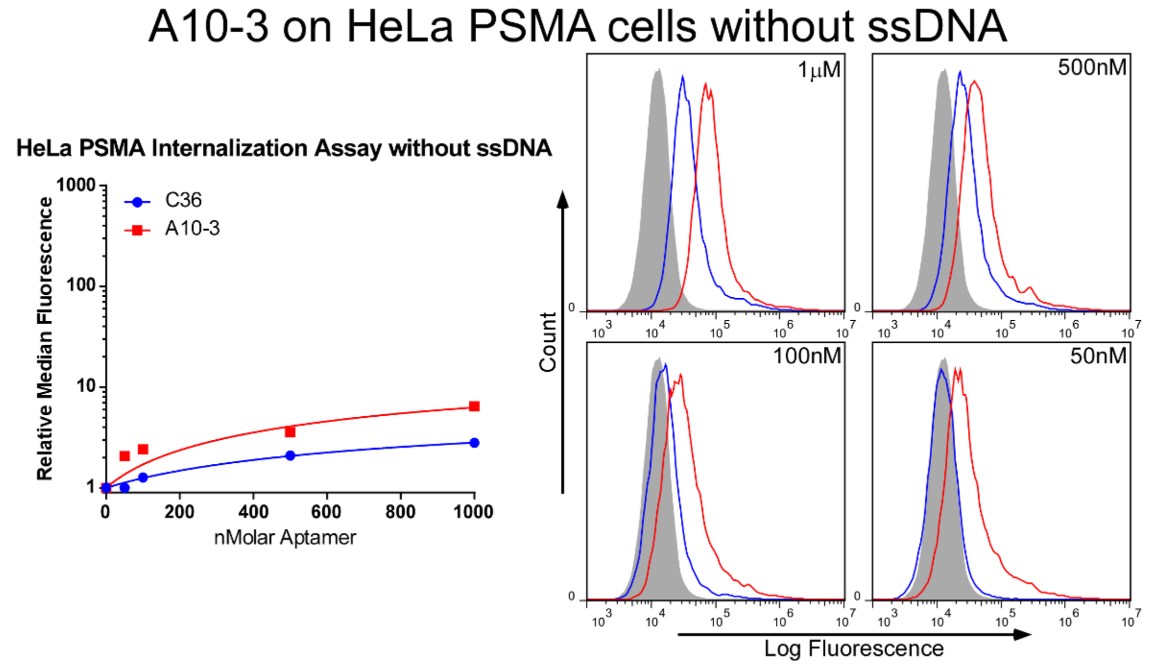

Supplementary Fig. 46. PSMA targeted aptamer A10-3 internalization and binding assays on HeLa PSMA cells. Graphs represent the median fluorescence of the aptamer (Red) and C36 (Blue) relative to unstained cells (Gray).

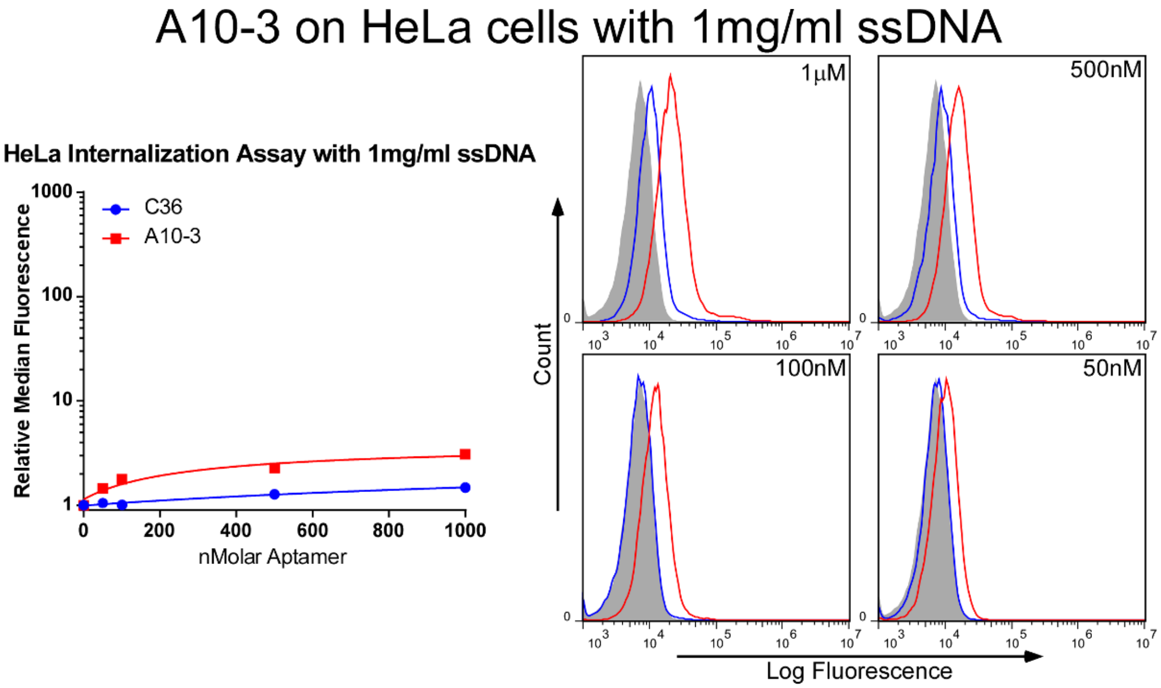

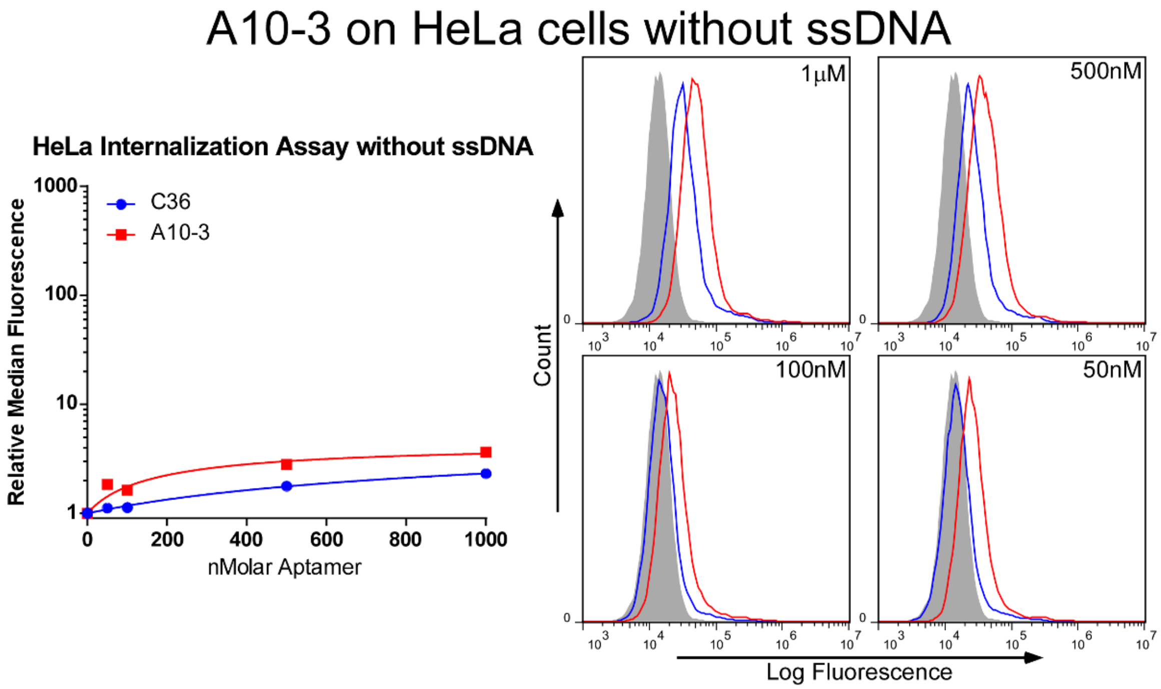

Supplementary Fig. 47. PSMA targeted aptamer A10-3 internalization and binding assays on HeLa cells. Graphs represent the median fluorescence of the aptamer (Red) and C36 (Blue) relative to unstained cells (Gray).

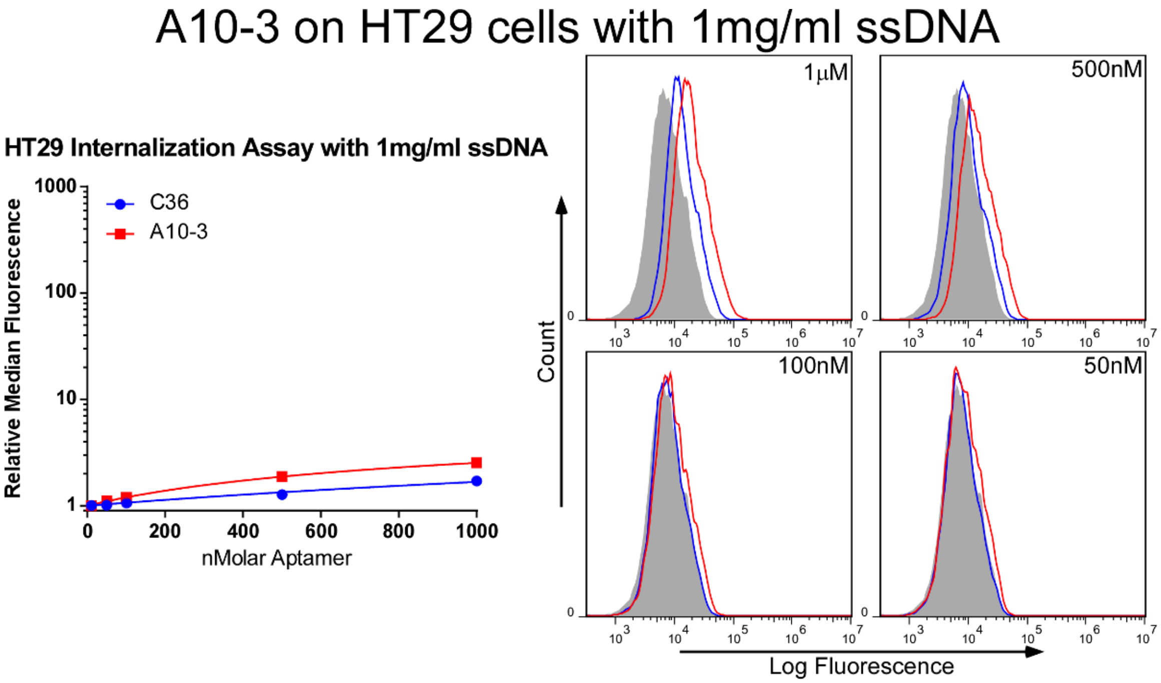

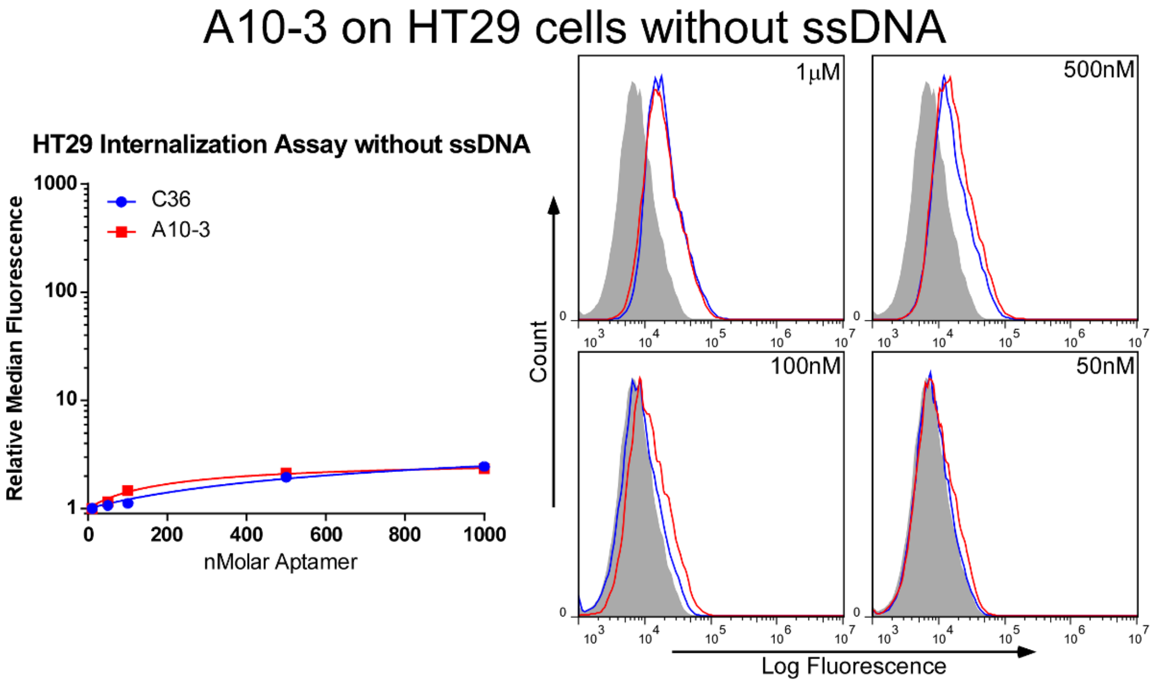

Supplementary Fig. 48. PSMA targeted aptamer A10-3 internalization and binding assays on HT29 cells. Graphs represent the median fluorescence of the aptamer (Red) and C36 (Blue) relative to unstained cells (Gray).

A10-3 on Jurkat cells with 1 mg/ml ssDNA

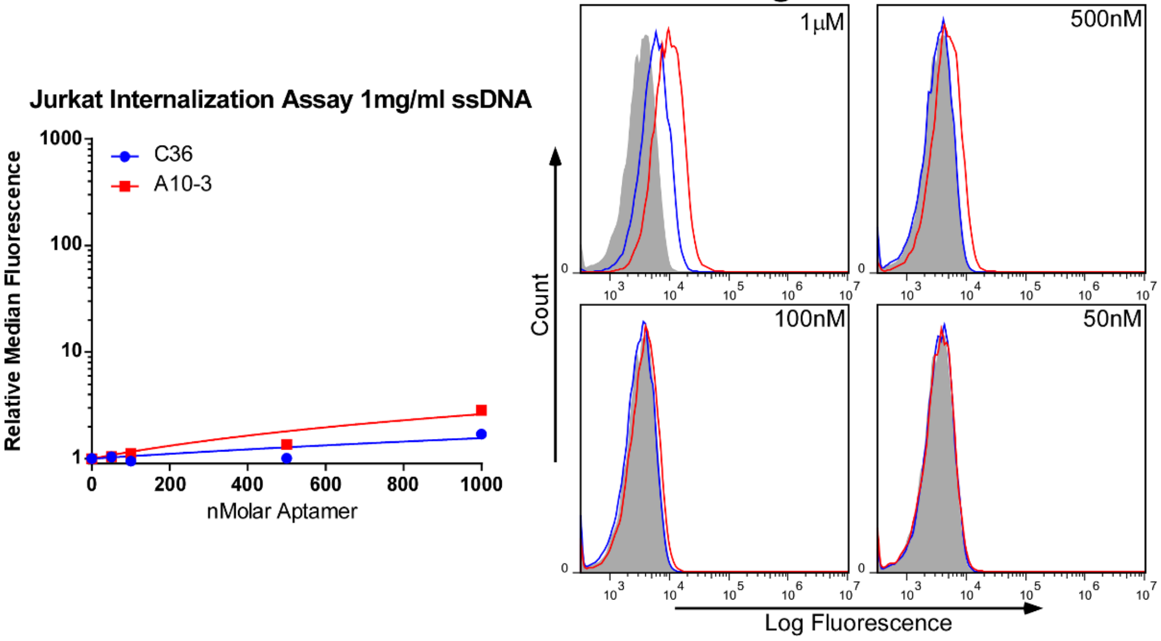

A10-3 on Jurkat cells without ssDNA

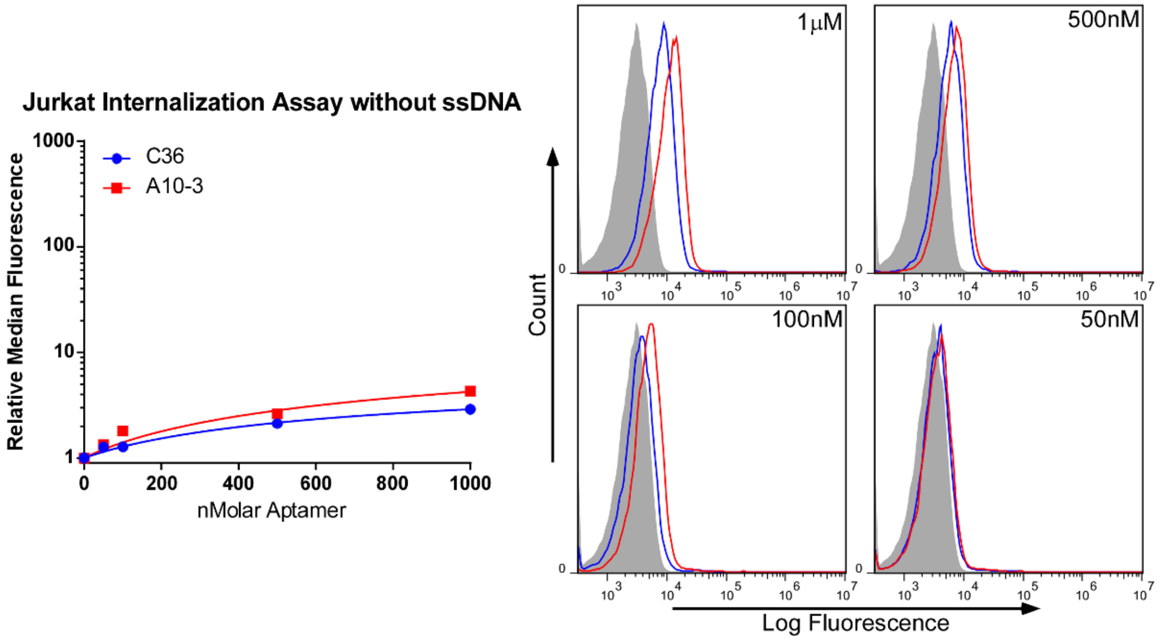

Supplementary Fig. 49. PSMA targeted aptamer A10-3 internalization and binding assays on Jurkat cells. Graphs represent the median fluorescence of the aptamer (Red) and C36 (Blue) relative to unstained cells (Gray).

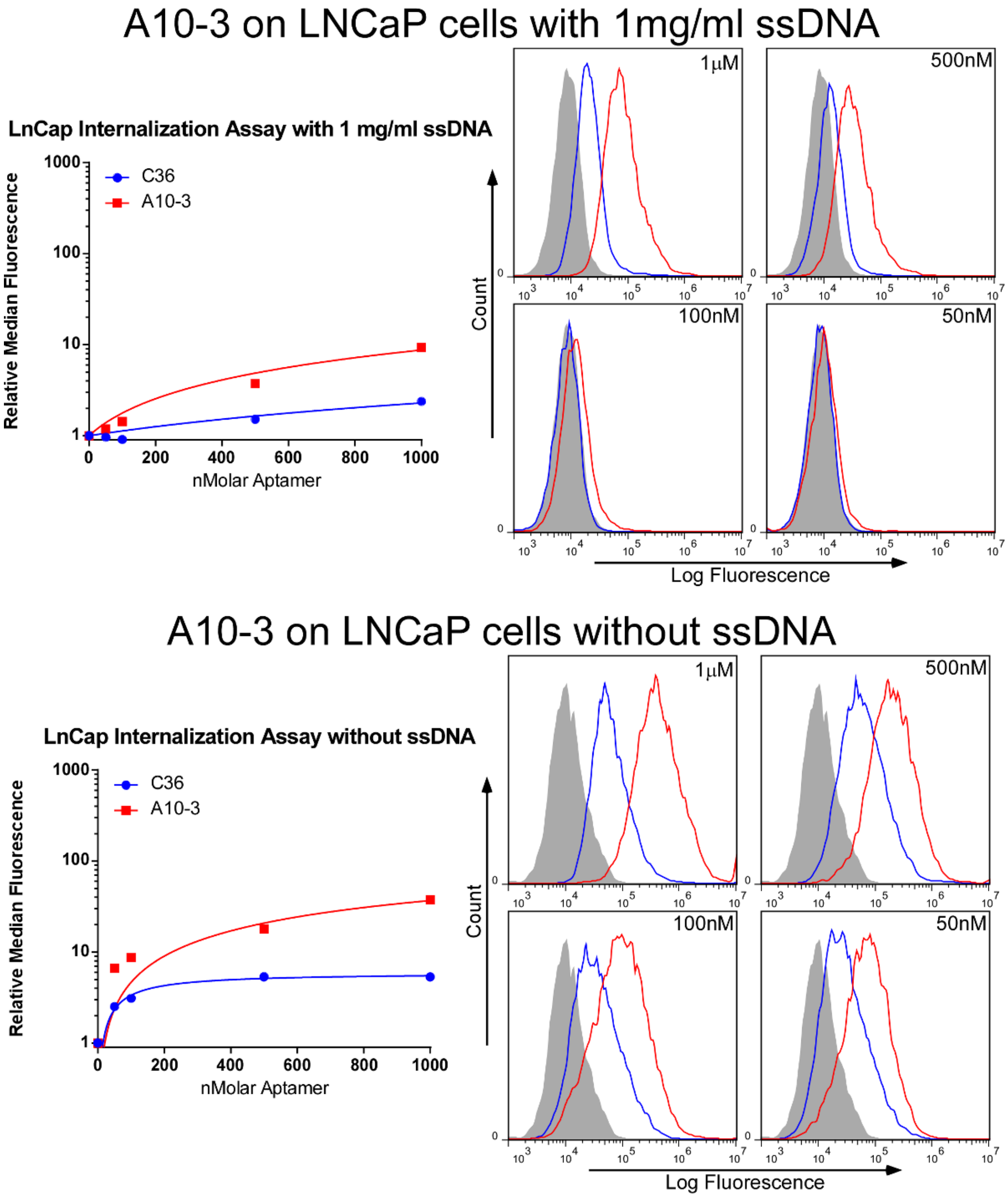

Supplementary Fig. 50. PSMA targeted aptamer A10-3 internalization and binding assays on LNCaP cells. Graphs represent the median fluorescence of the aptamer (Red) and C36 (Blue) relative to unstained cells (Gray).

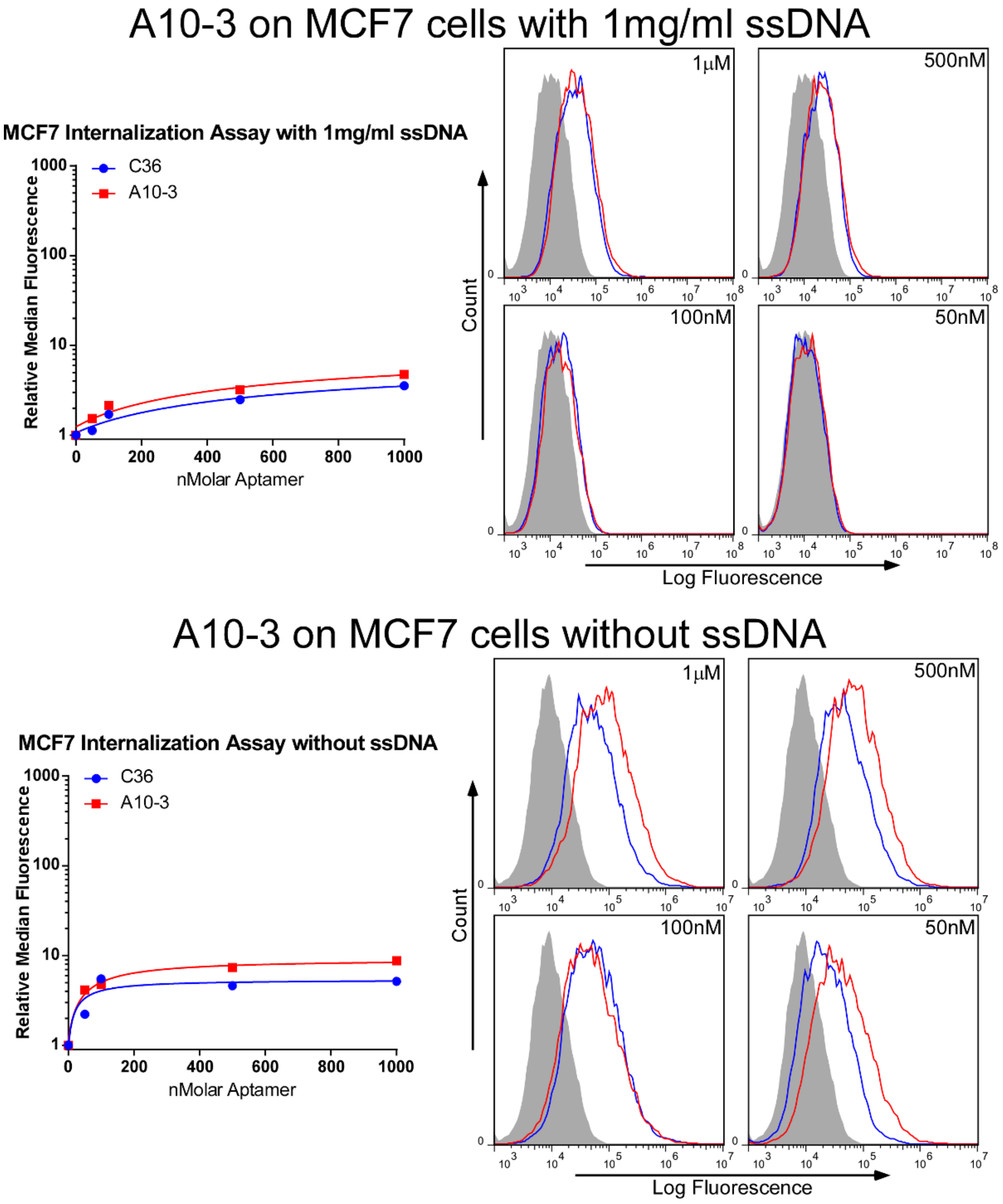

Supplementary Fig. 51. PSMA targeted aptamer A10-3 internalization and binding assays on MCF7 cells. Graphs represent the median fluorescence of the aptamer (Red) and C36 (Blue) relative to unstained cells (Gray).

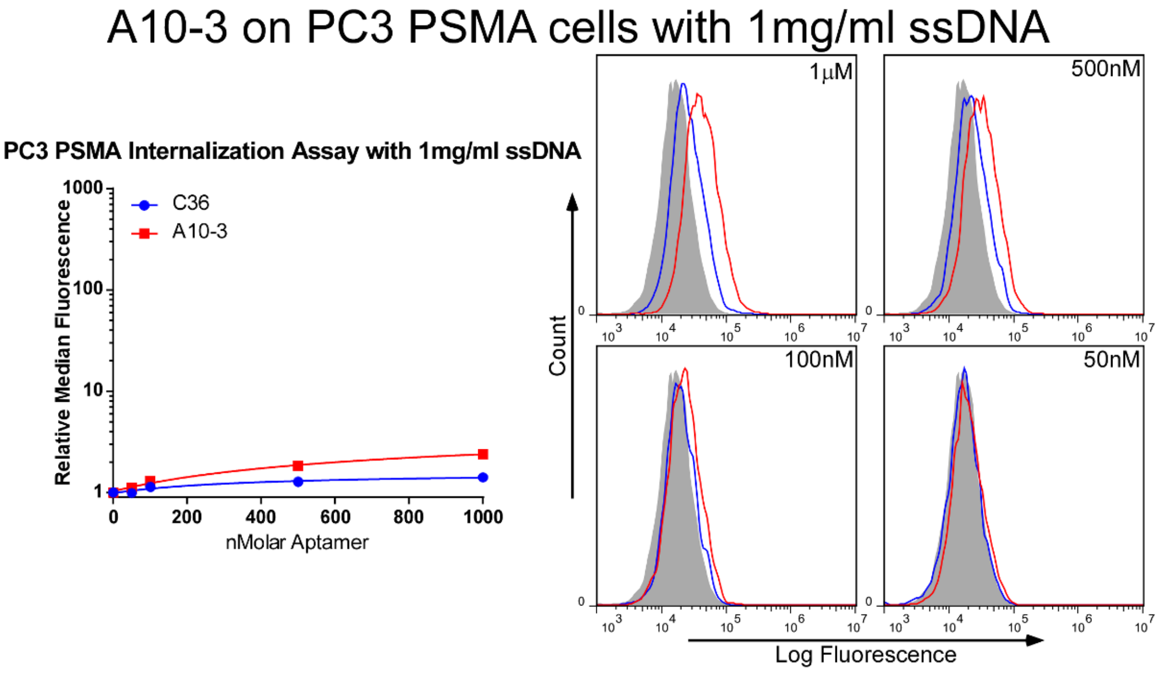

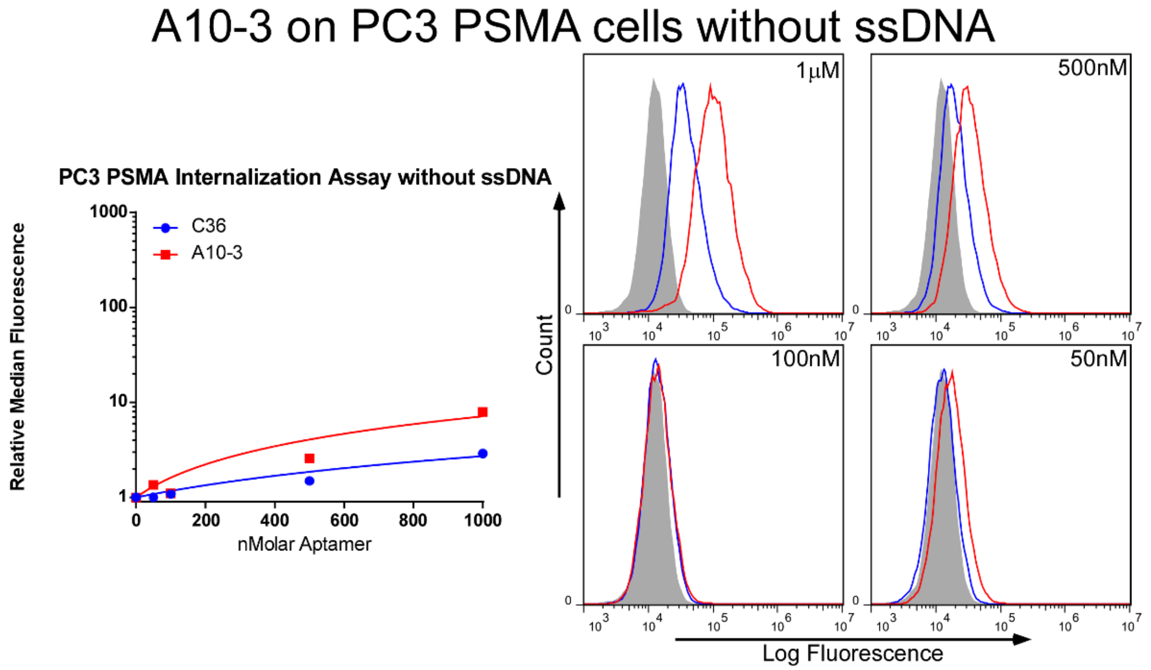

Supplementary Fig. 52. PSMA targeted aptamer A10-3 internalization and binding assays on PC3 PSMA cells. Graphs represent the median fluorescence of the aptamer (Red) and C36 (Blue) relative to unstained cells (Gray).

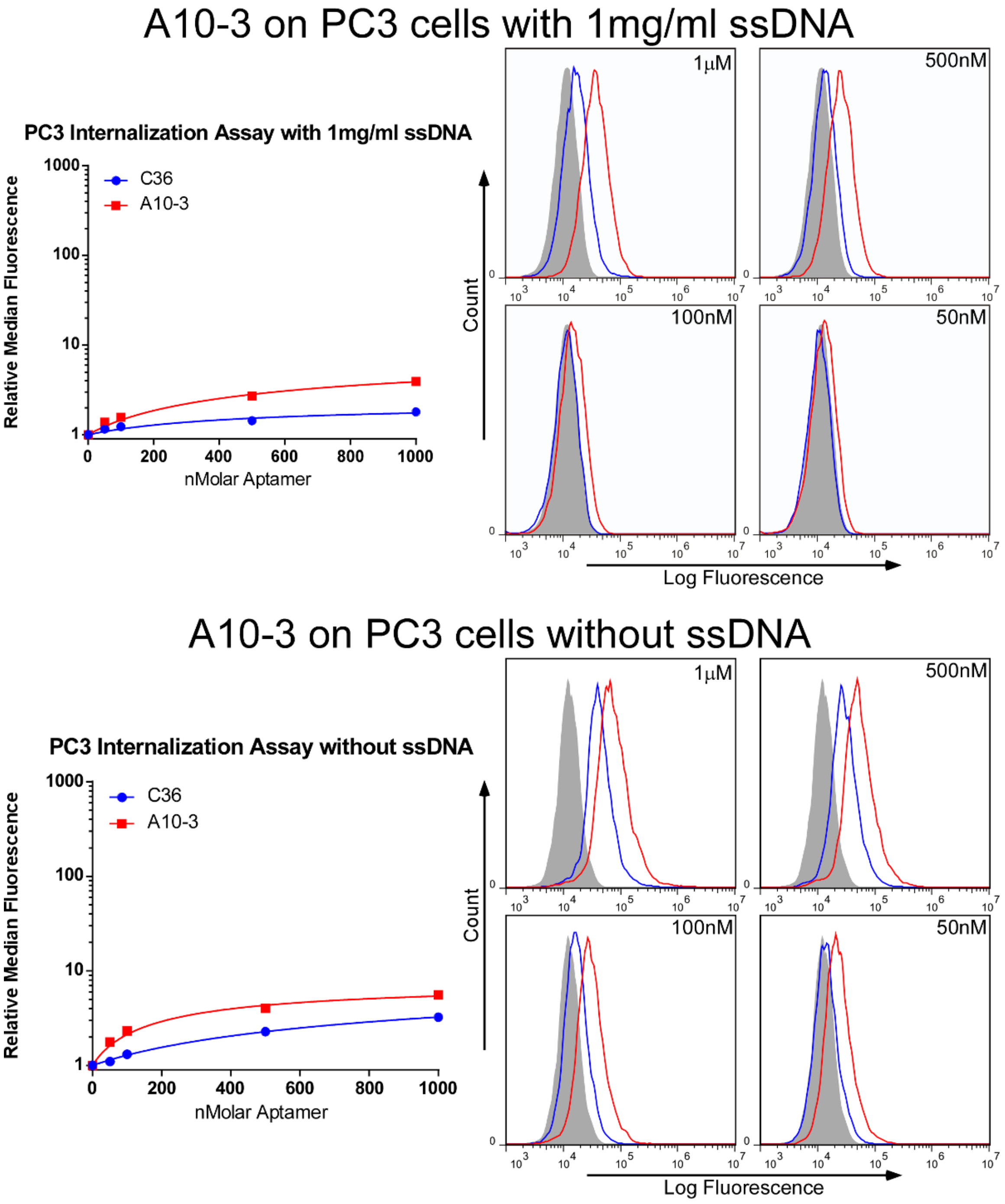

Supplementary Fig. 53. PSMA targeted aptamer A10-3 internalization and binding assays on PC3 cells. Graphs represent the median fluorescence of the aptamer (Red) and C36 (Blue) relative to unstained cells (Gray).

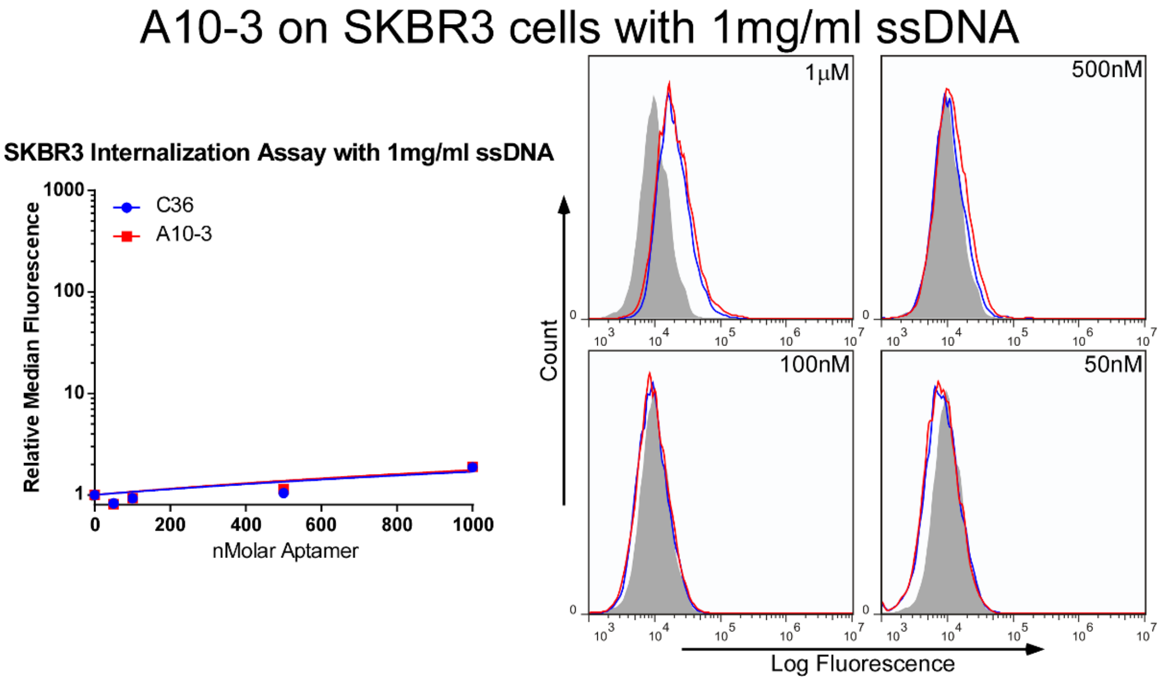

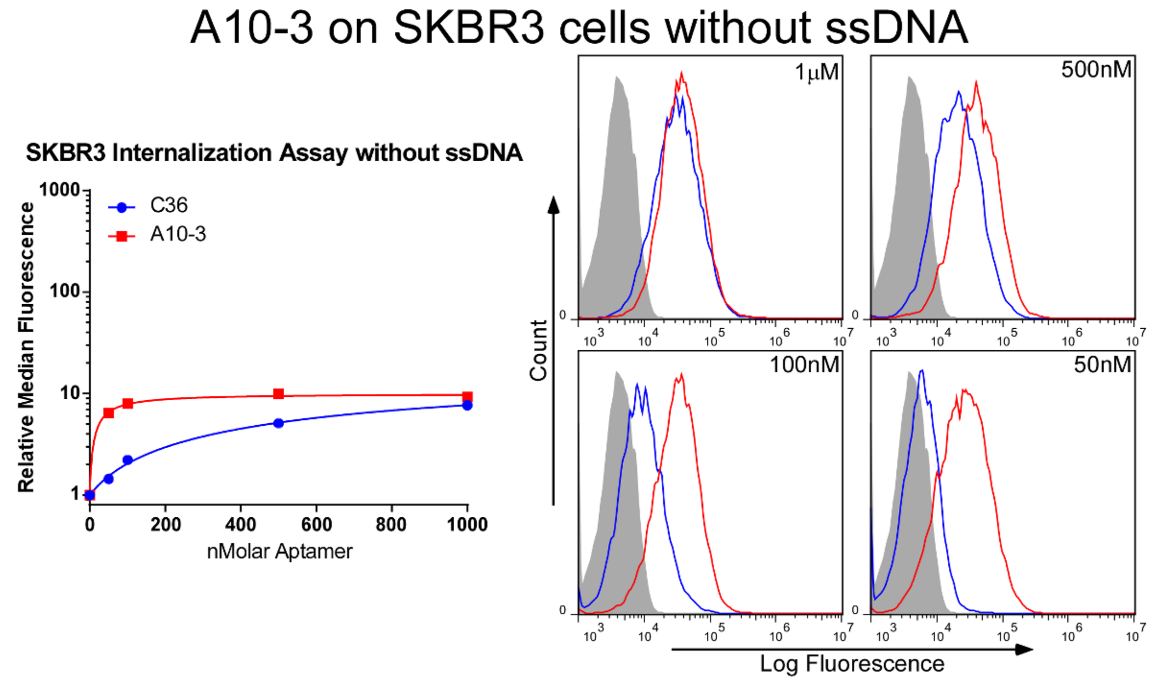

Supplementary Fig. 54. PSMA targeted aptamer A10-3 internalization and binding assays on SKBR3 cells. Graphs represent the median fluorescence of the aptamer (Red) and C36 (Blue) relative to unstained cells (Gray).

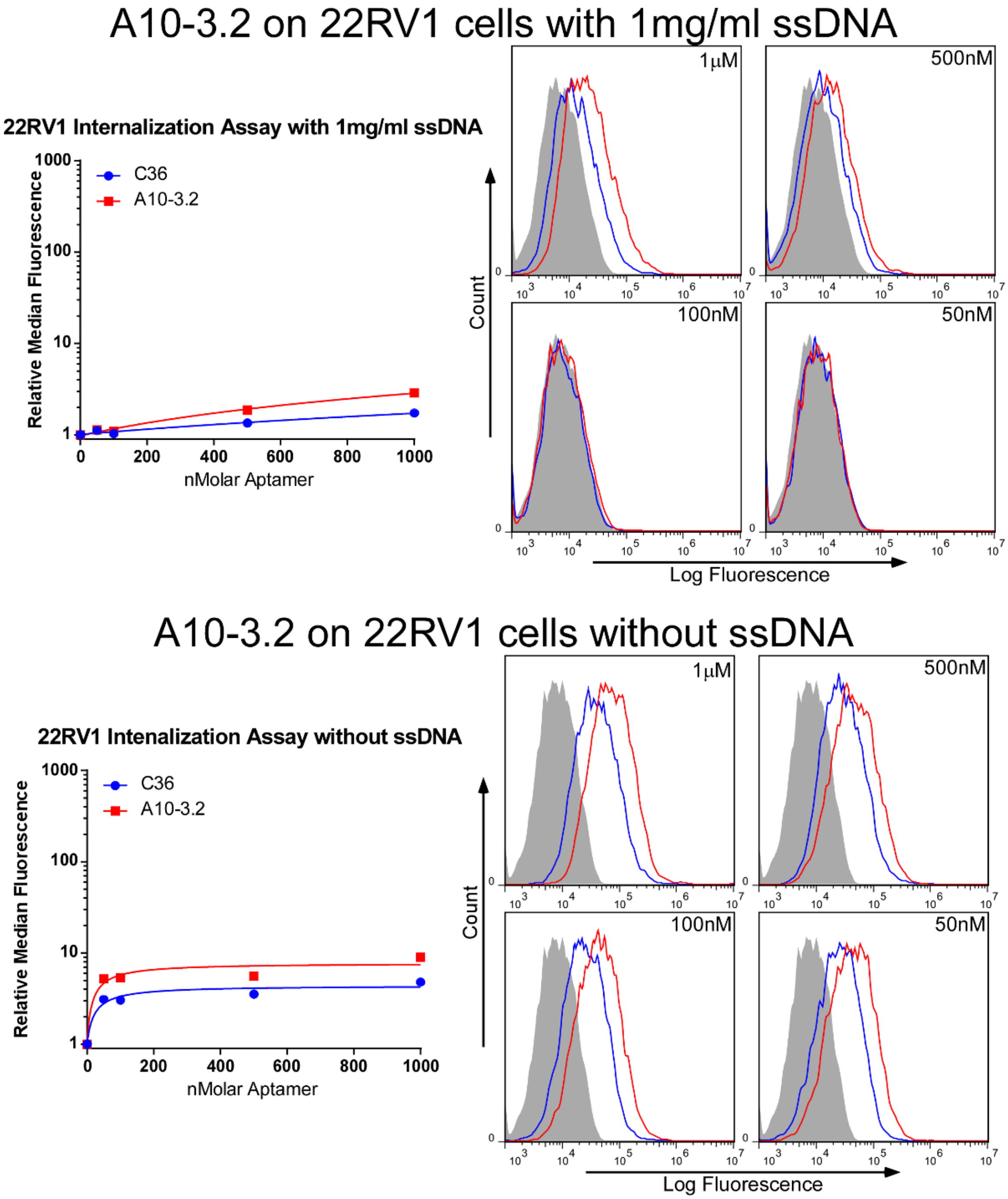

Supplementary Fig. 55. PSMA targeted aptamer A10-3.2 internalization and binding assays on 22RV1 cells. Graphs represent the median fluorescence of the aptamer (Red) and C36 (Blue) relative to unstained cells (Gray).

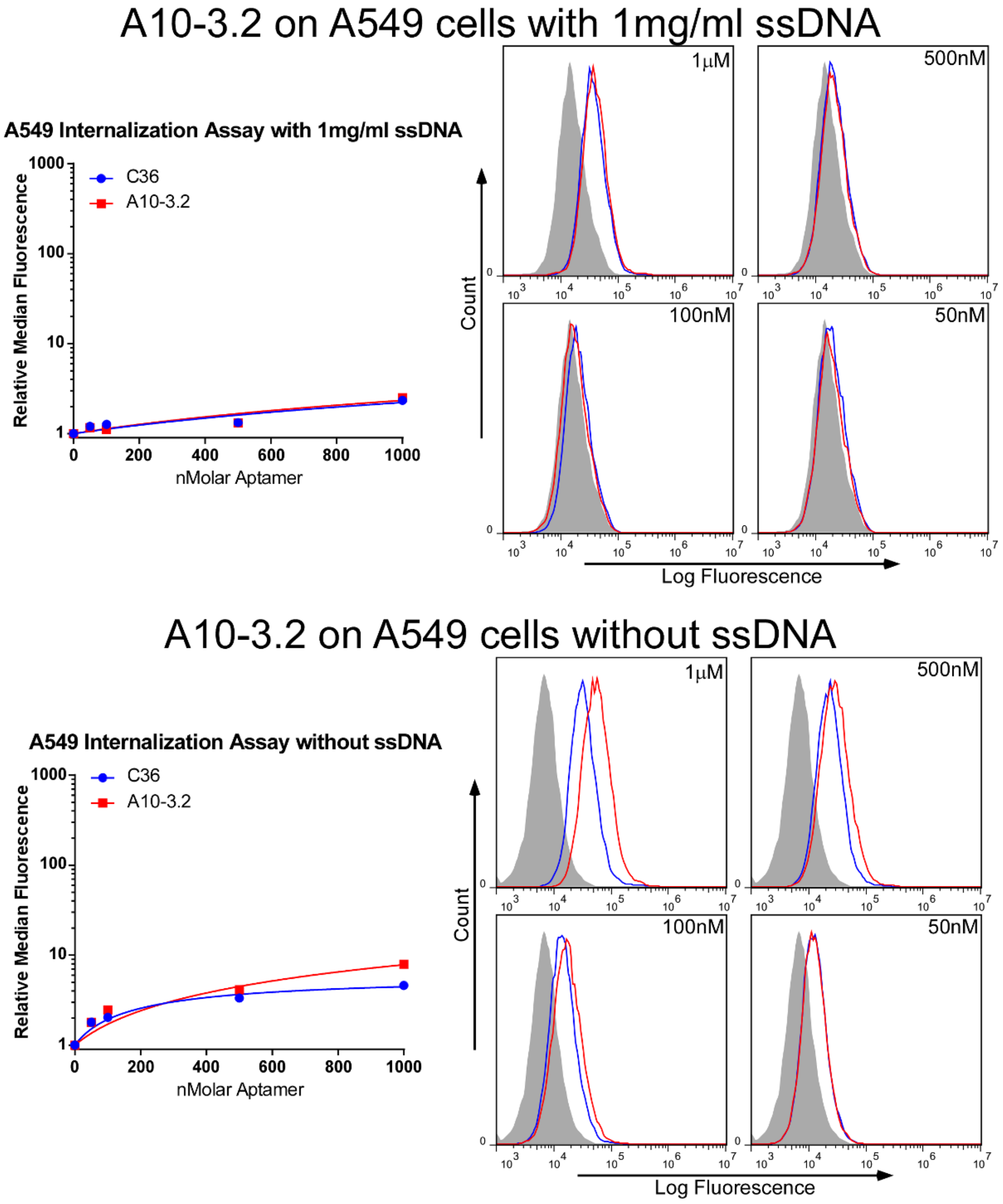

Supplementary Fig. 56. PSMA targeted aptamer A10-3.2 internalization and binding assays on A549 cells. Graphs represent the median fluorescence of the aptamer (Red) and C36 (Blue) relative to unstained cells (Gray).

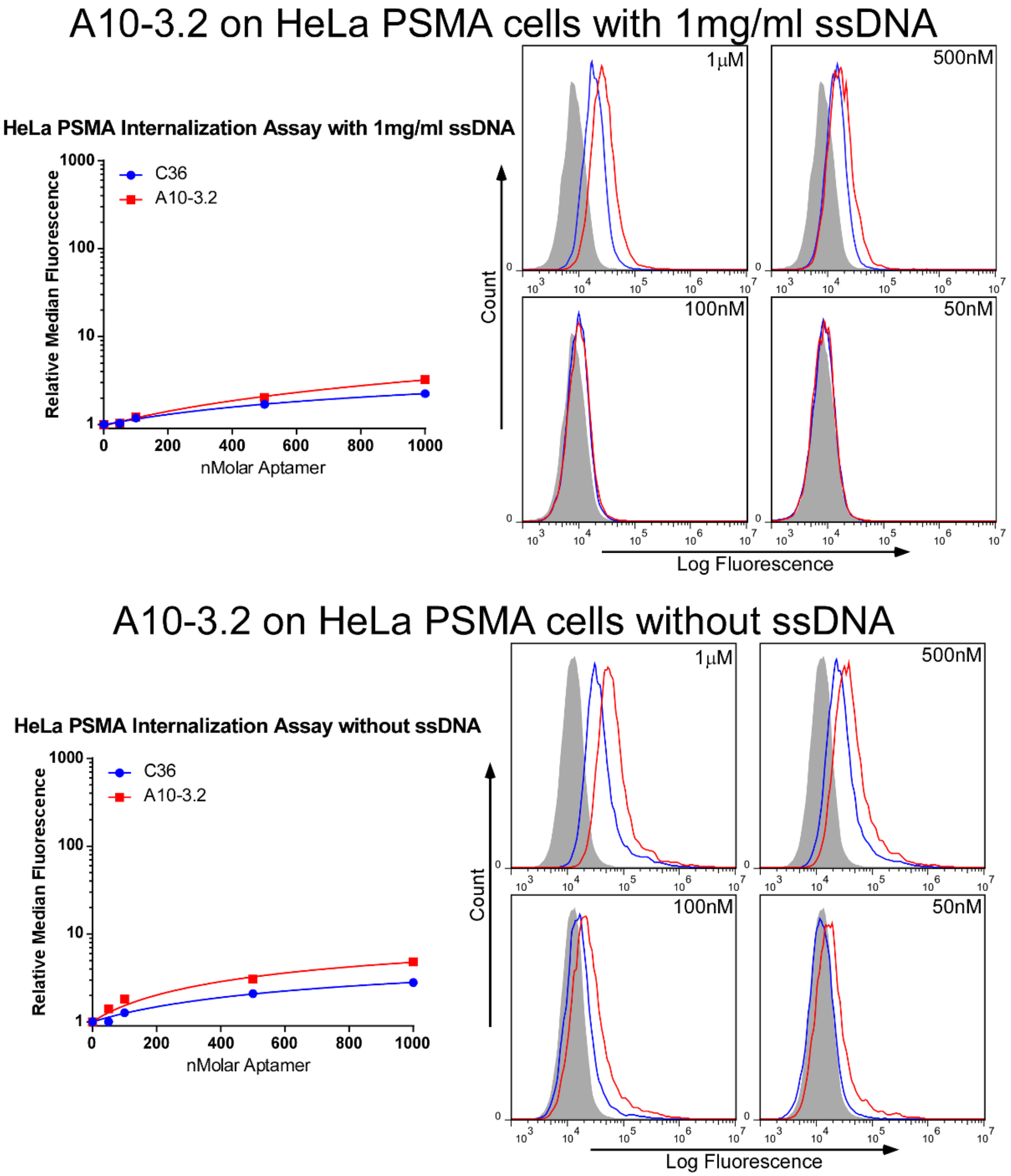

Supplementary Fig. 57. PSMA targeted aptamer A10-3.2 internalization and binding assays on HeLa PSMA cells. Graphs represent the median fluorescence of the aptamer (Red) and C36 (Blue) relative to unstained cells (Gray).

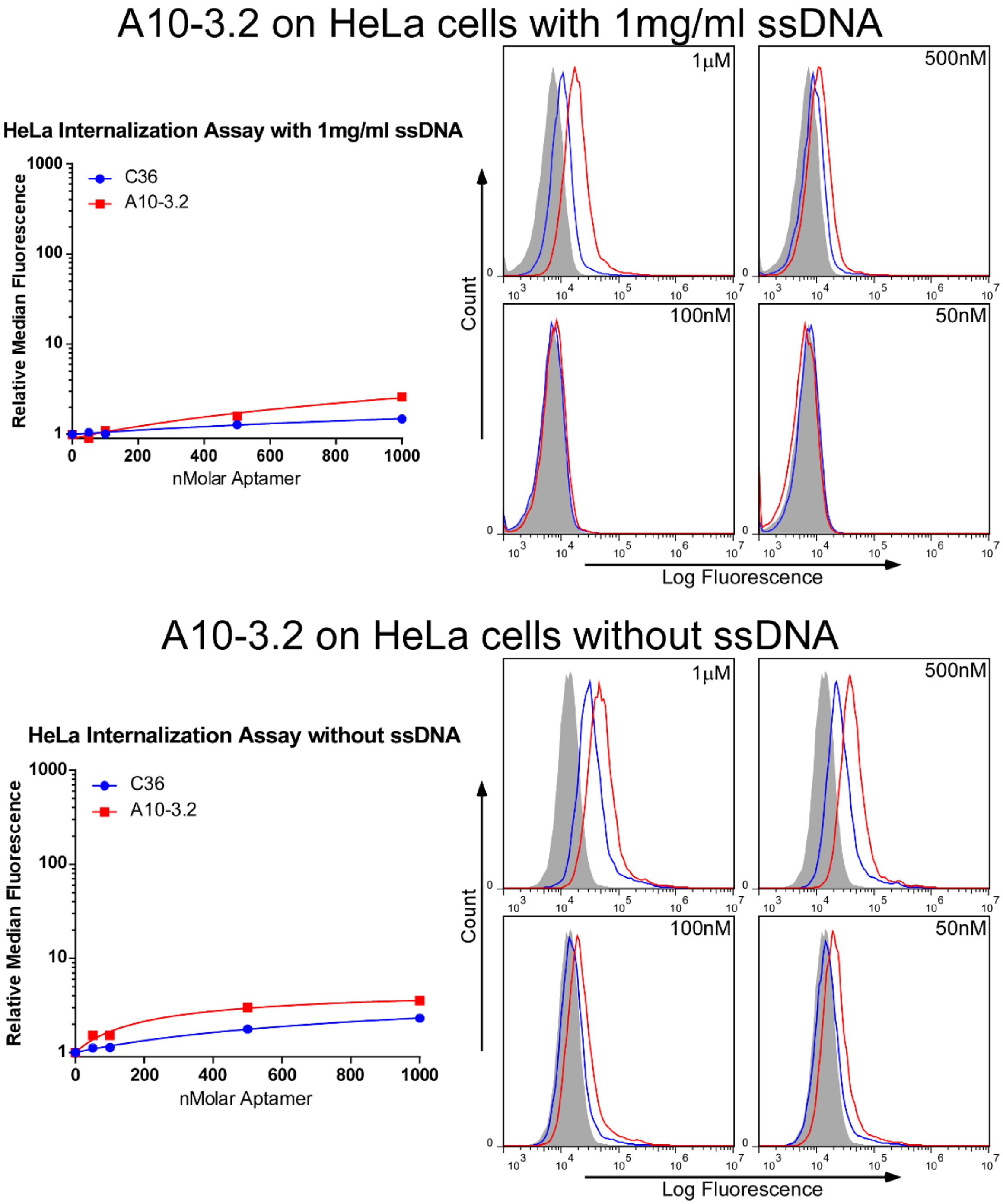

Supplementary Fig. 58. PSMA targeted aptamer A10-3.2 internalization and binding assays on HeLa cells. Graphs represent the median fluorescence of the aptamer (Red) and C36 (Blue) relative to unstained cells (Gray).

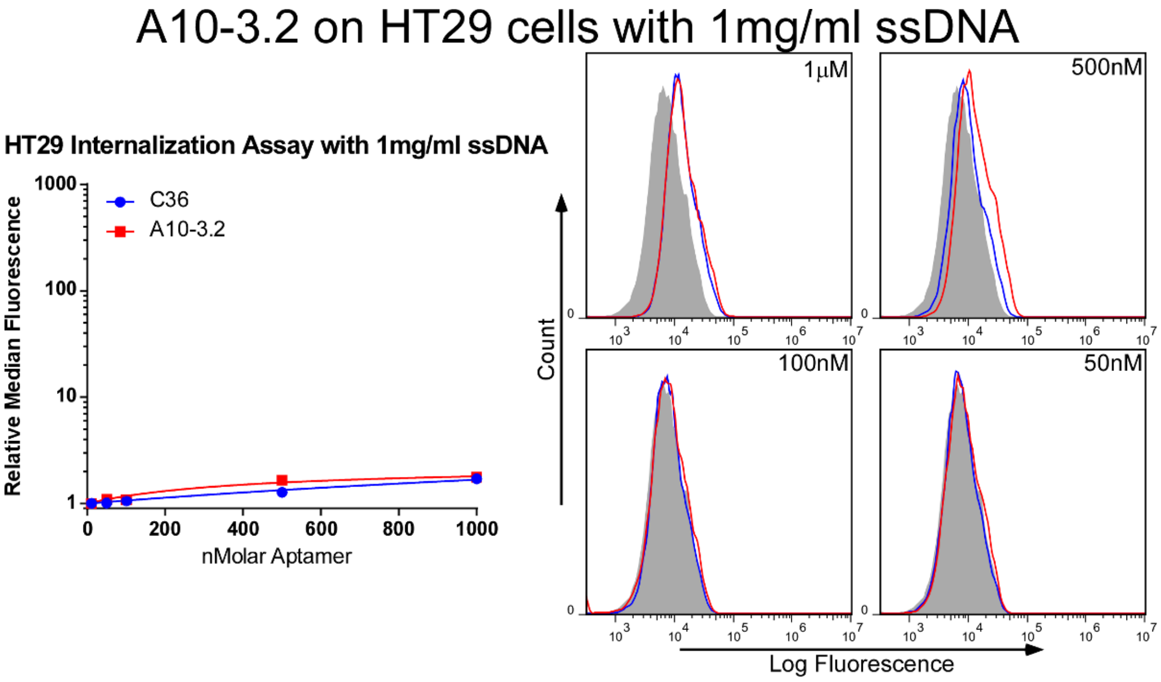

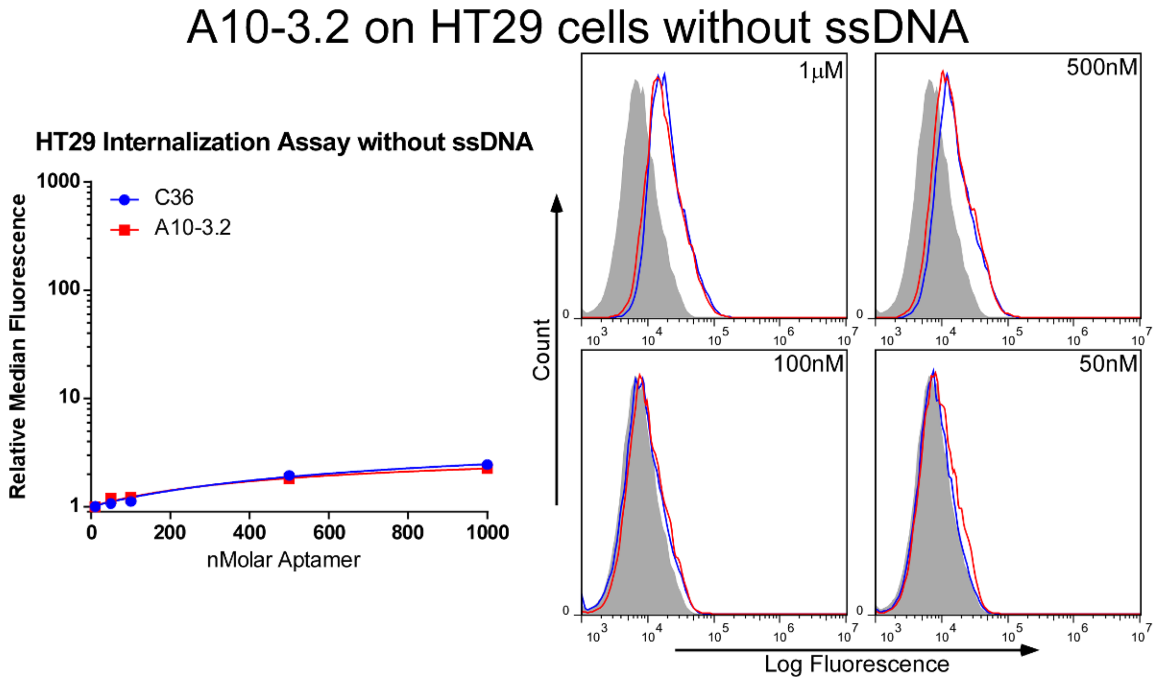

Supplementary Fig. 59. PSMA targeted aptamer A10-3.2 internalization and binding assays on HT29 cells. Graphs represent the median fluorescence of the aptamer (Red) and C36 (Blue) relative to unstained cells (Gray).

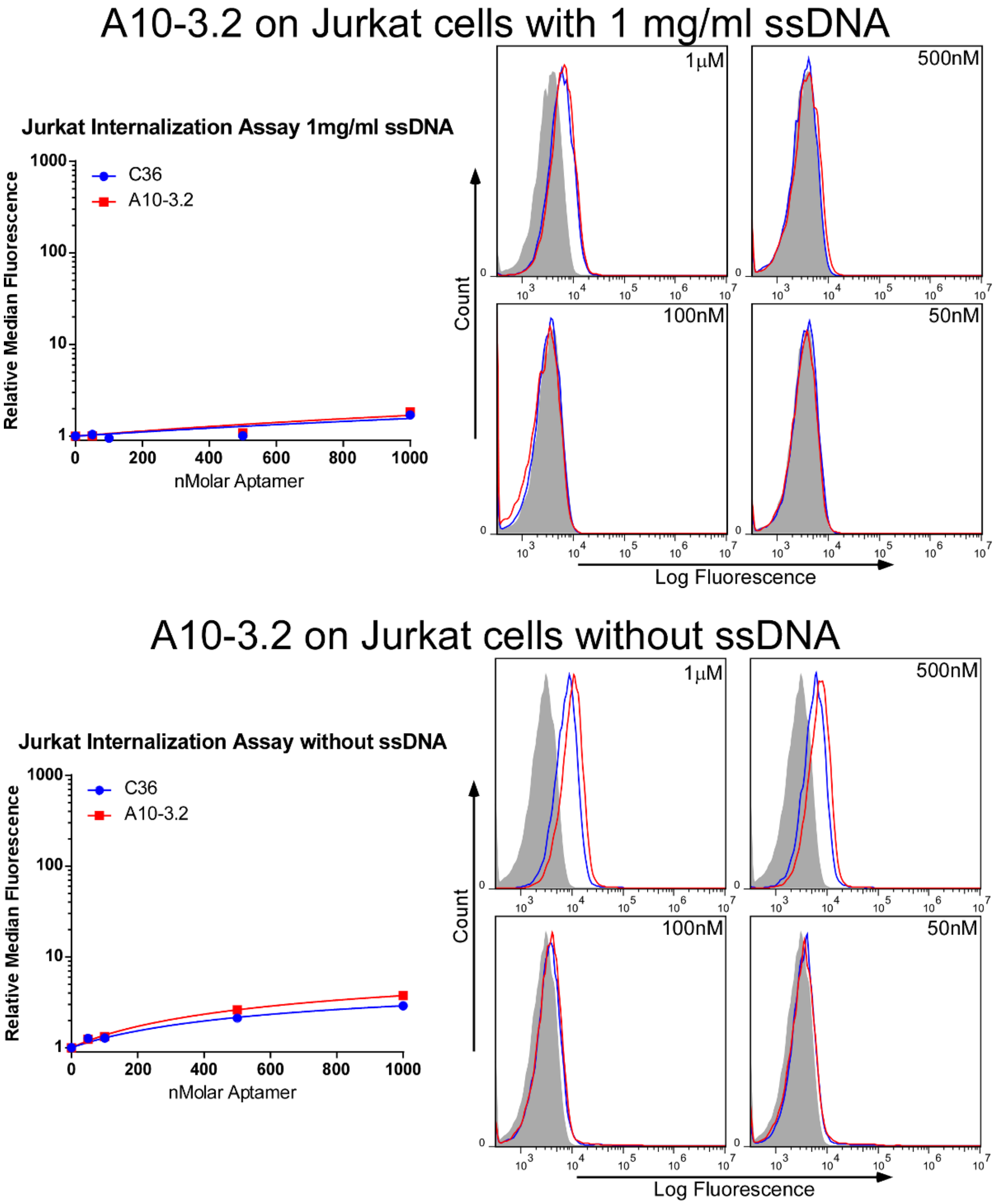

Supplementary Fig. 60. PSMA targeted aptamer A10-3.2 internalization and binding assays on Jurkat cells. Graphs represent the median fluorescence of the aptamer (Red) and C36 (Blue) relative to unstained cells (Gray).

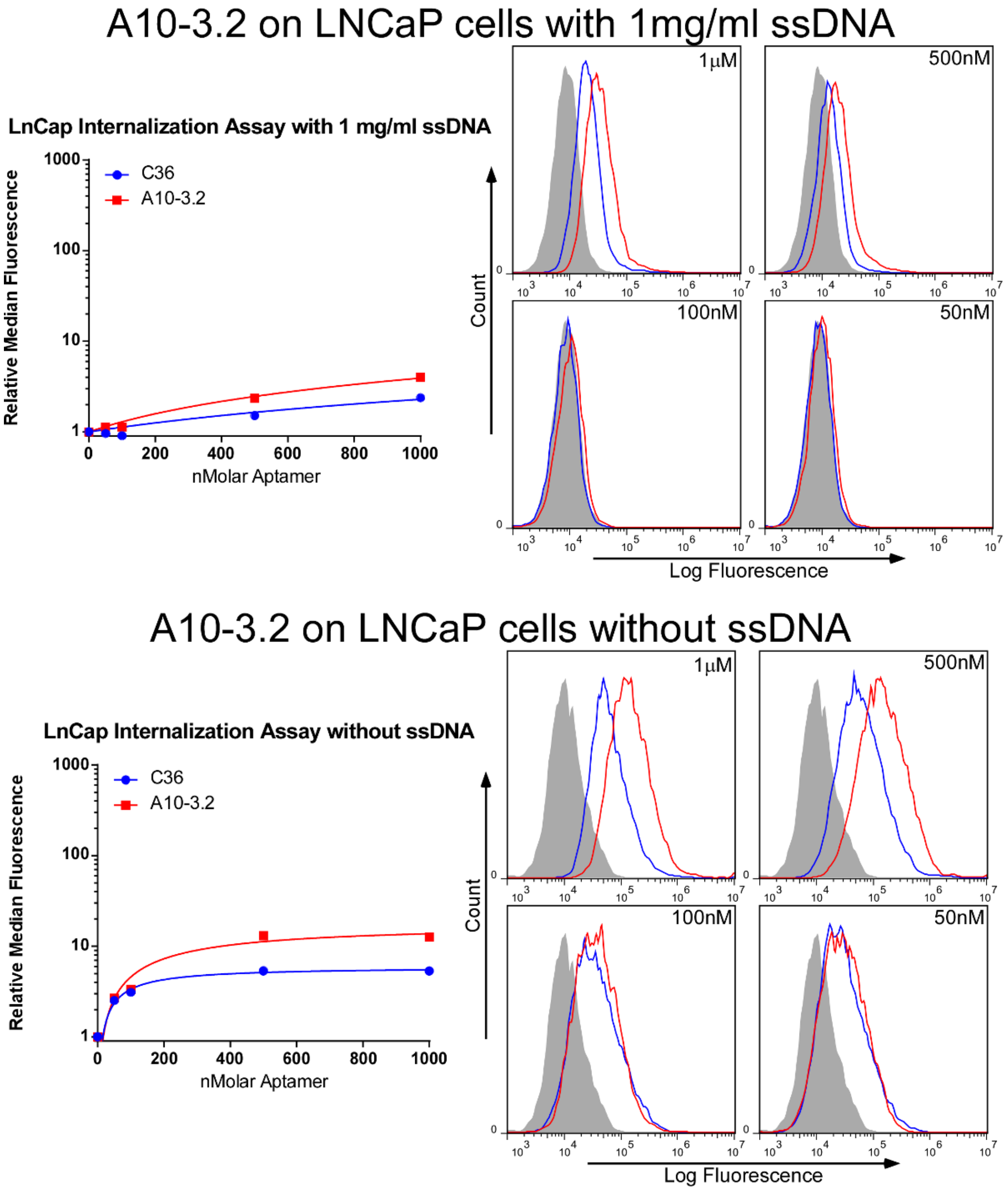

Supplementary Fig. 61. PSMA targeted aptamer A10-3.2 internalization and binding assays on LNCaP cells. Graphs represent the median fluorescence of the aptamer (Red) and C36 (Blue) relative to unstained cells (Gray).

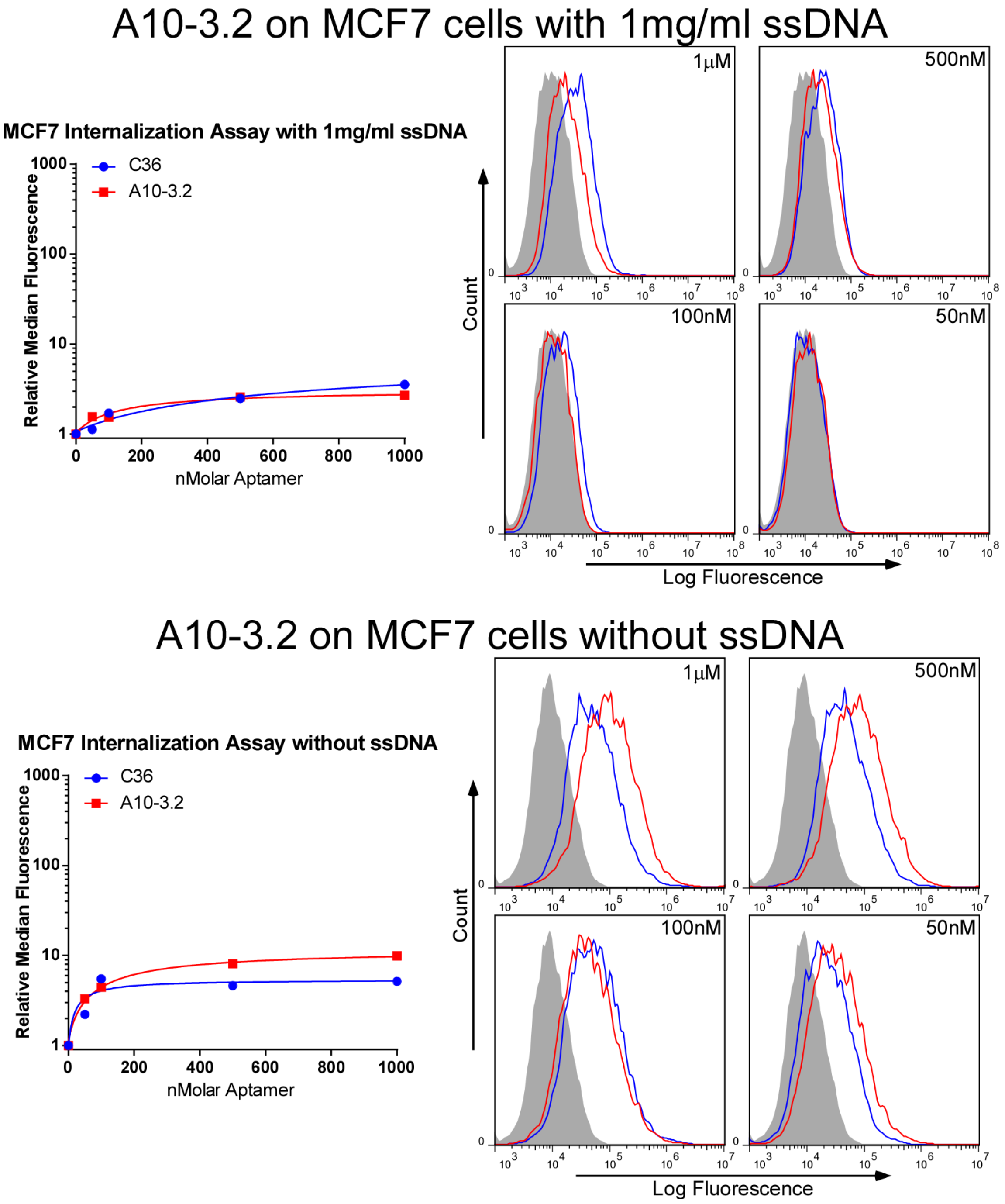

Supplementary Fig. 62. PSMA targeted aptamer A10-3.2 internalization and binding assays on MCF7 cells. Graphs represent the median fluorescence of the aptamer (Red) and C36 (Blue) relative to unstained cells (Gray).

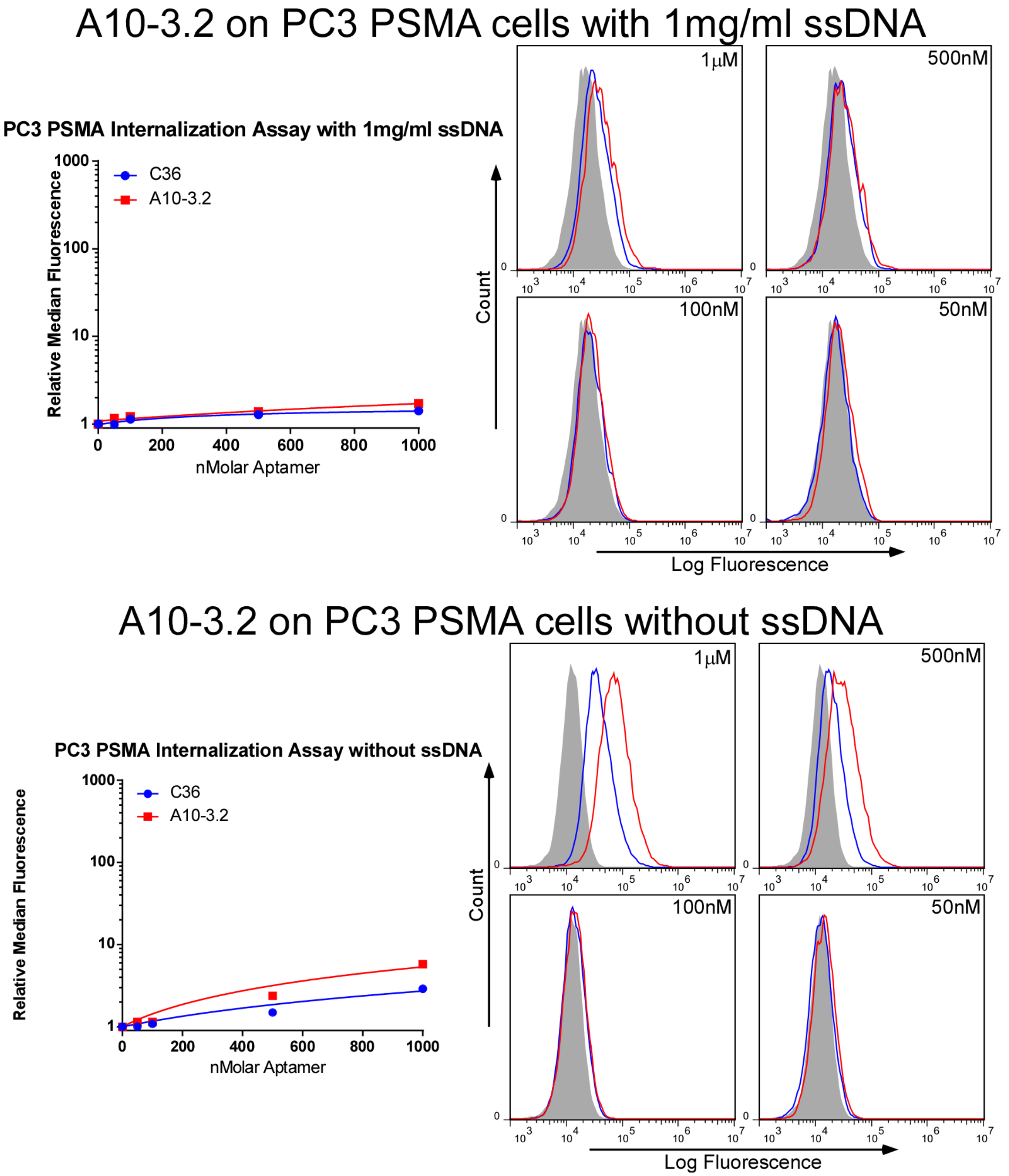

Supplementary Fig. 63. PSMA targeted aptamer A10-3.2 internalization and binding assays on PC3 PSMA cells. Graphs represent the median fluorescence of the aptamer (Red) and C36 (Blue) relative to unstained cells (Gray).

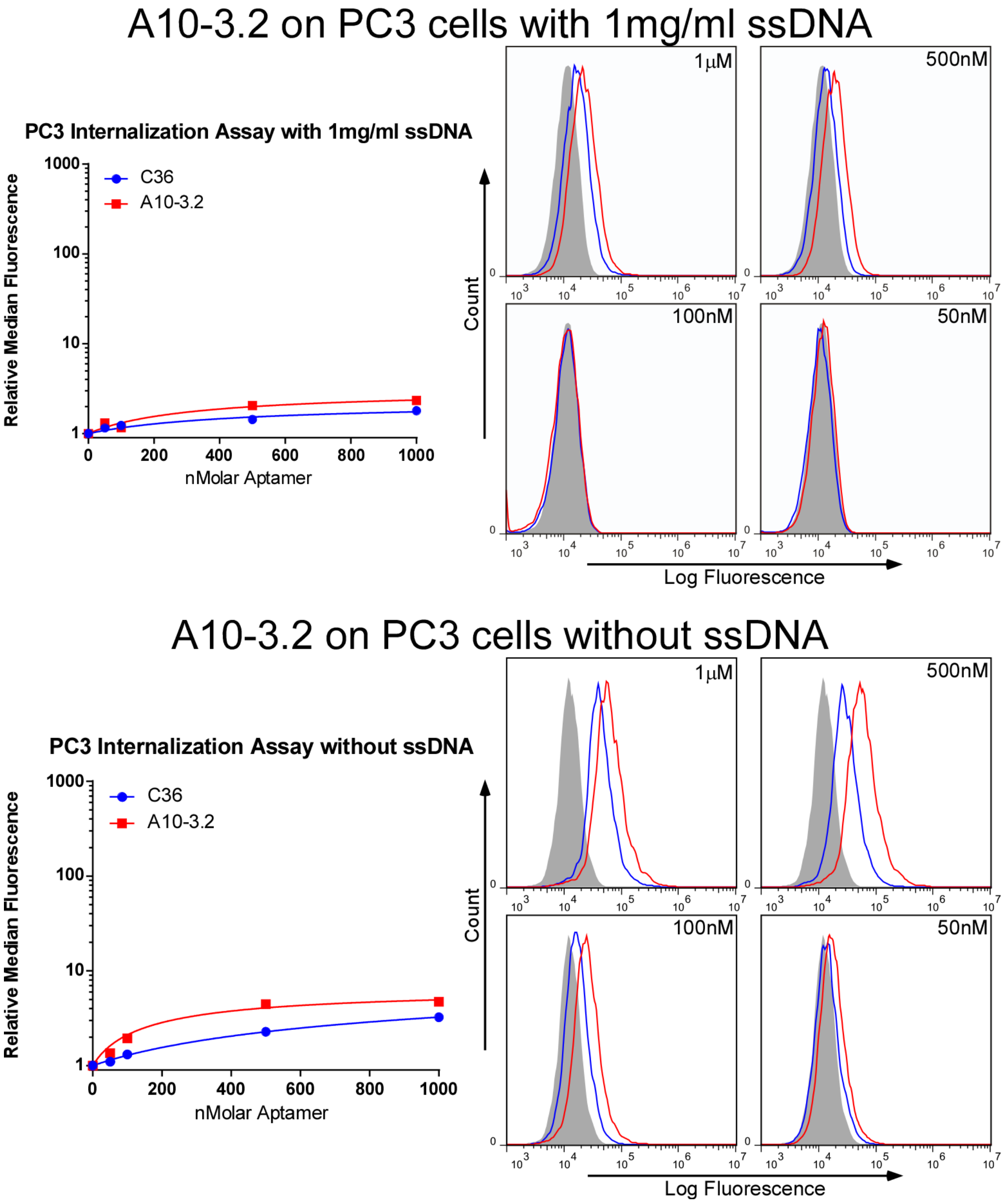

Supplementary Fig. 64. PSMA targeted aptamer A10-3.2 internalization and binding assays on PC3 cells. Graphs represent the median fluorescence of the aptamer (Red) and C36 (Blue) relative to unstained cells (Gray).

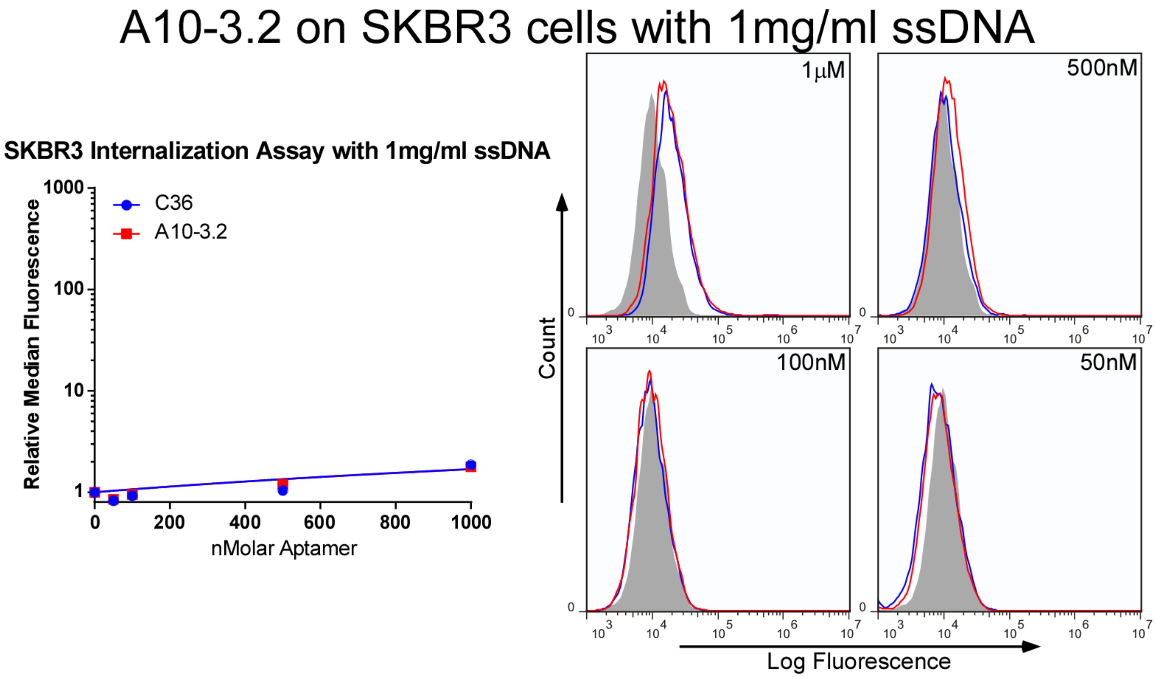

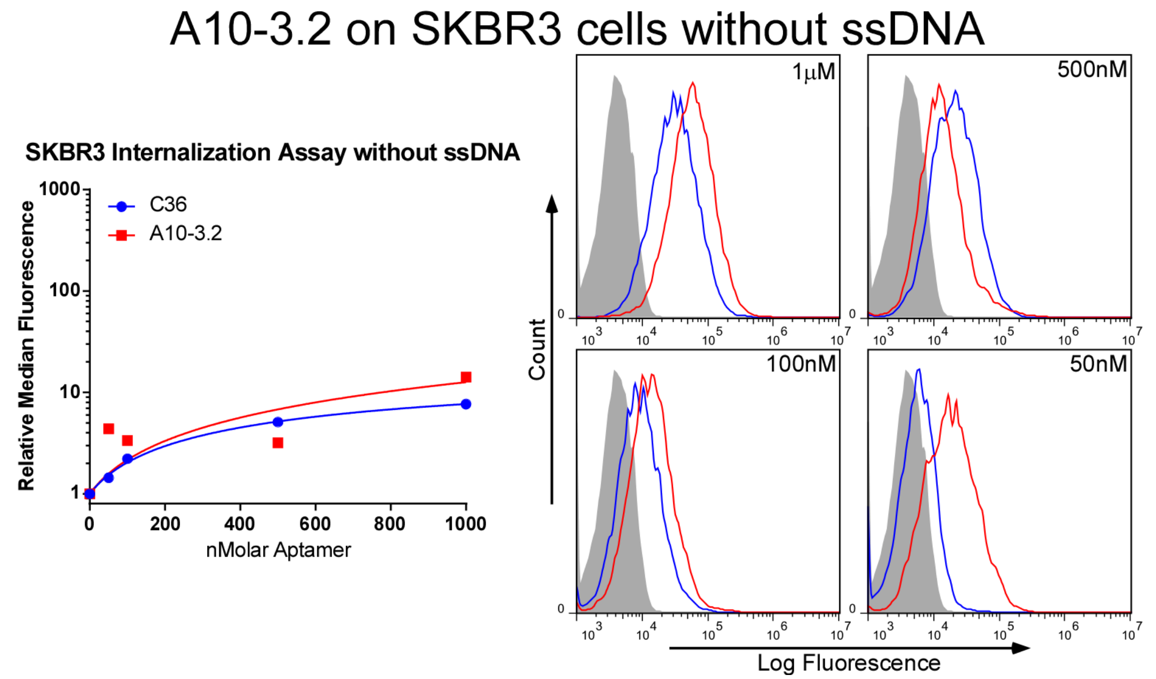

Supplementary Fig. 65. PSMA targeted aptamer A10-3.2 internalization and binding assays on SKBR3 cells. Graphs represent the median fluorescence of the aptamer (Red) and C36 (Blue) relative to unstained cells (Gray).

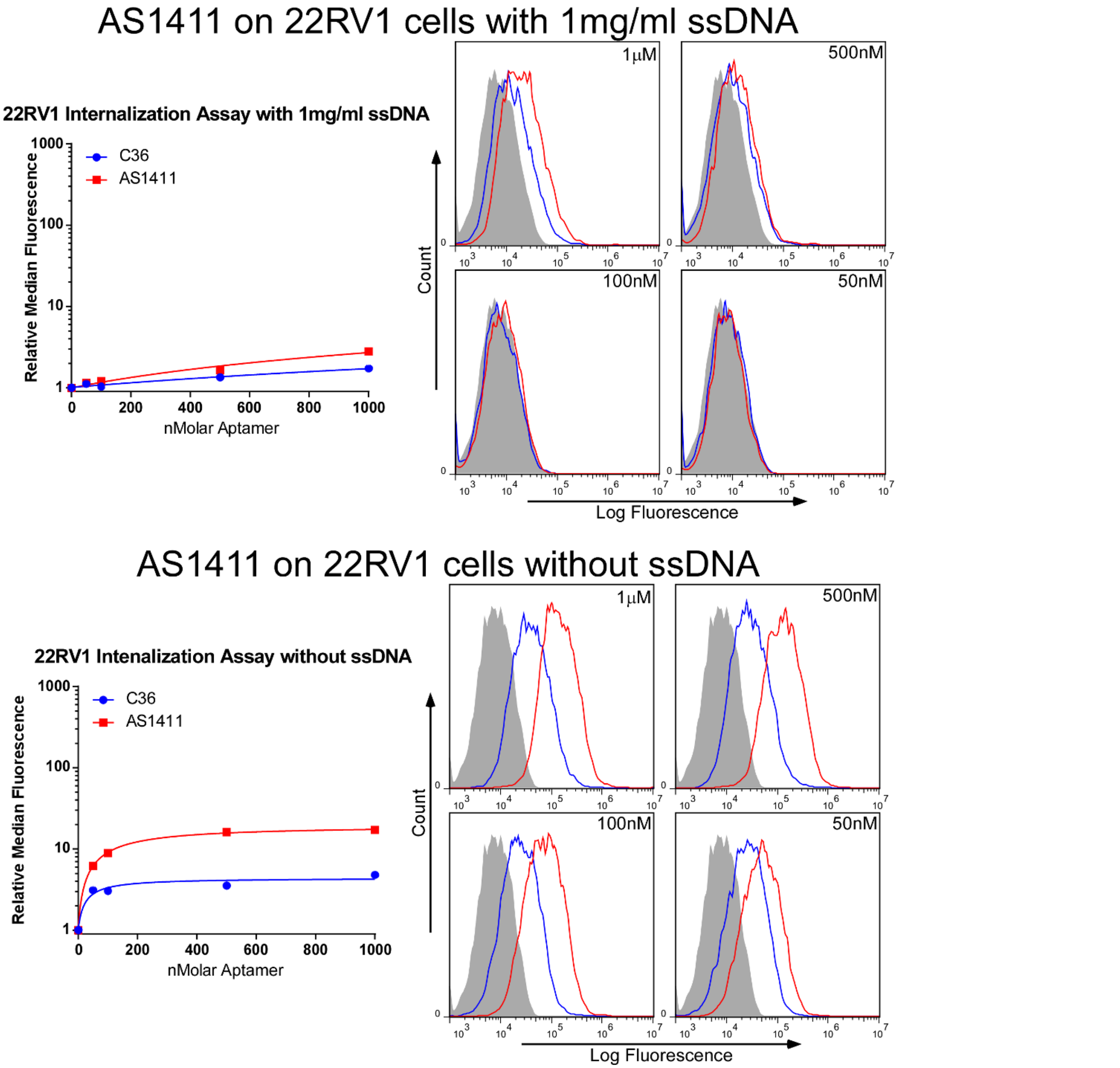

Supplementary Fig. 66. Nucleolin targeted aptamer AS1411 internalization and binding assays on 22RV1 cells. Graphs represent the median fluorescence of the aptamer (Red) and C36 (Blue) relative to unstained cells (Gray).

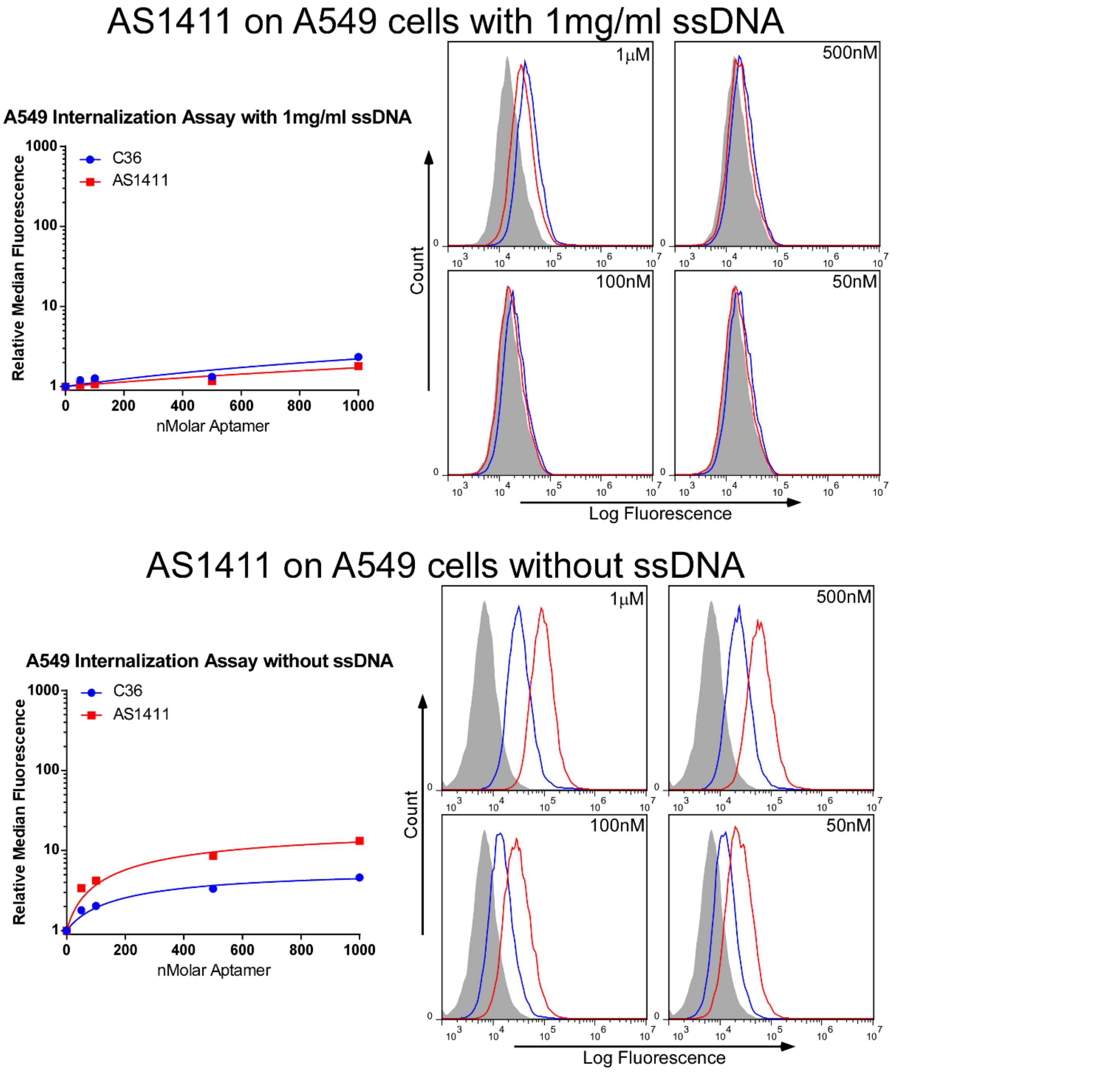

Supplementary Fig. 67. Nucleolin targeted aptamer AS1411 internalization and binding assays on A549 cells. Graphs represent the median fluorescence of the aptamer (Red) and C36 (Blue) relative to unstained cells (Gray).

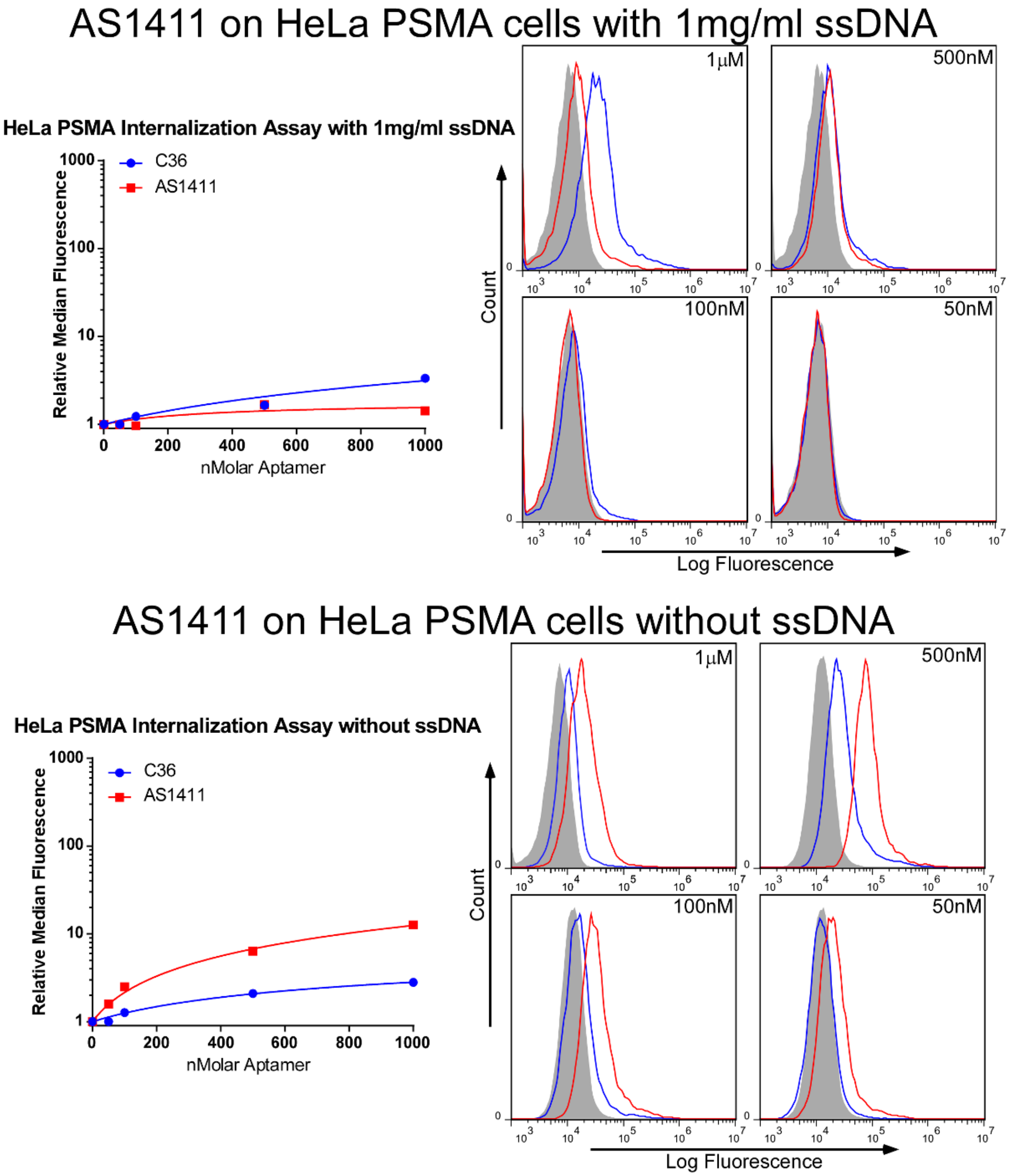

Supplementary Fig. 68. Nucleolin targeted aptamer AS1411 internalization and binding assays on HeLa PSMA cells. Graphs represent the median fluorescence of the aptamer (Red) and C36 (Blue) relative to unstained cells (Gray).

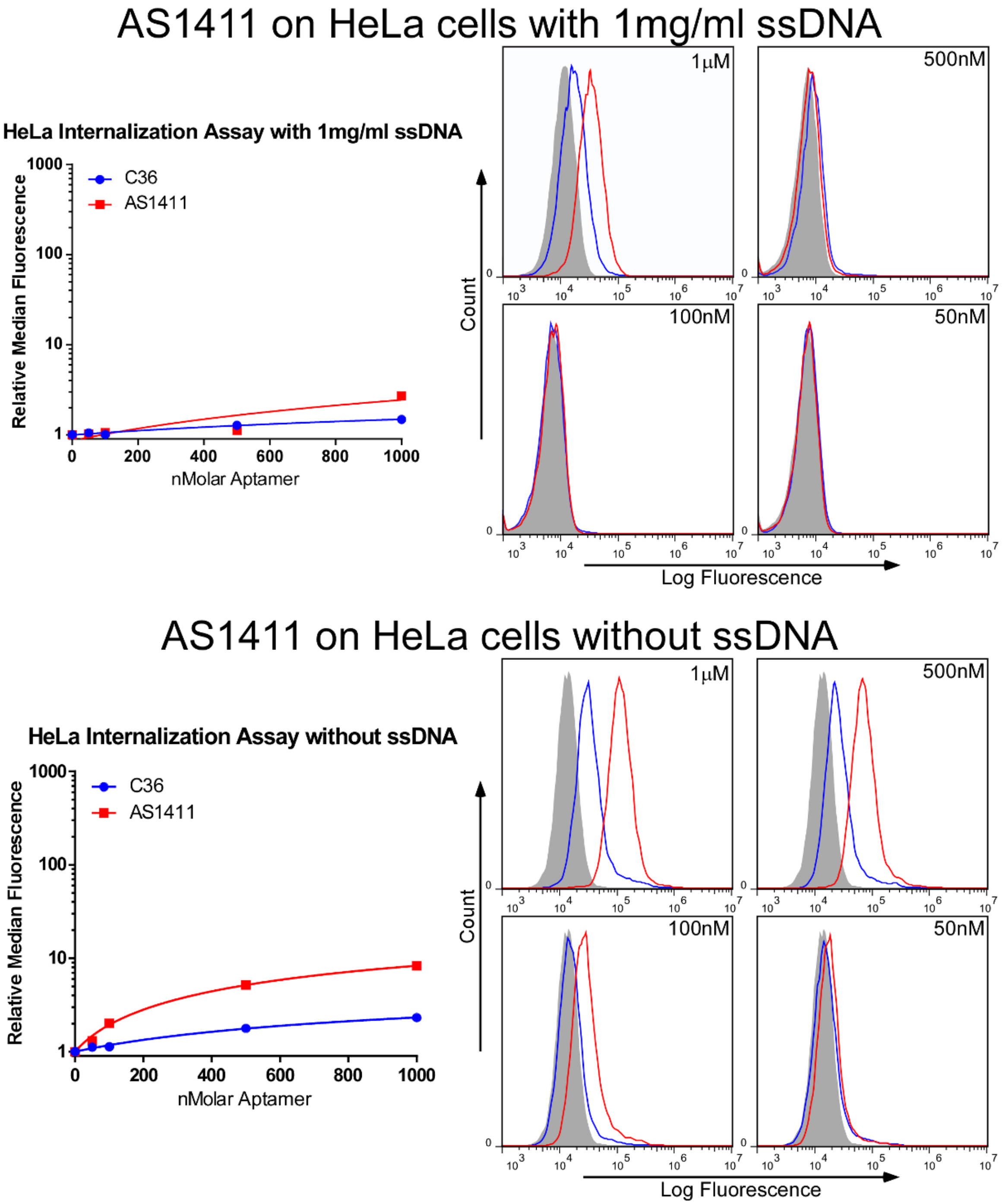

Supplementary Fig. 69. Nucleolin targeted aptamer AS1411 internalization and binding assays on HeLa cells. Graphs represent the median fluorescence of the aptamer (Red) and C36 (Blue) relative to unstained cells (Gray).

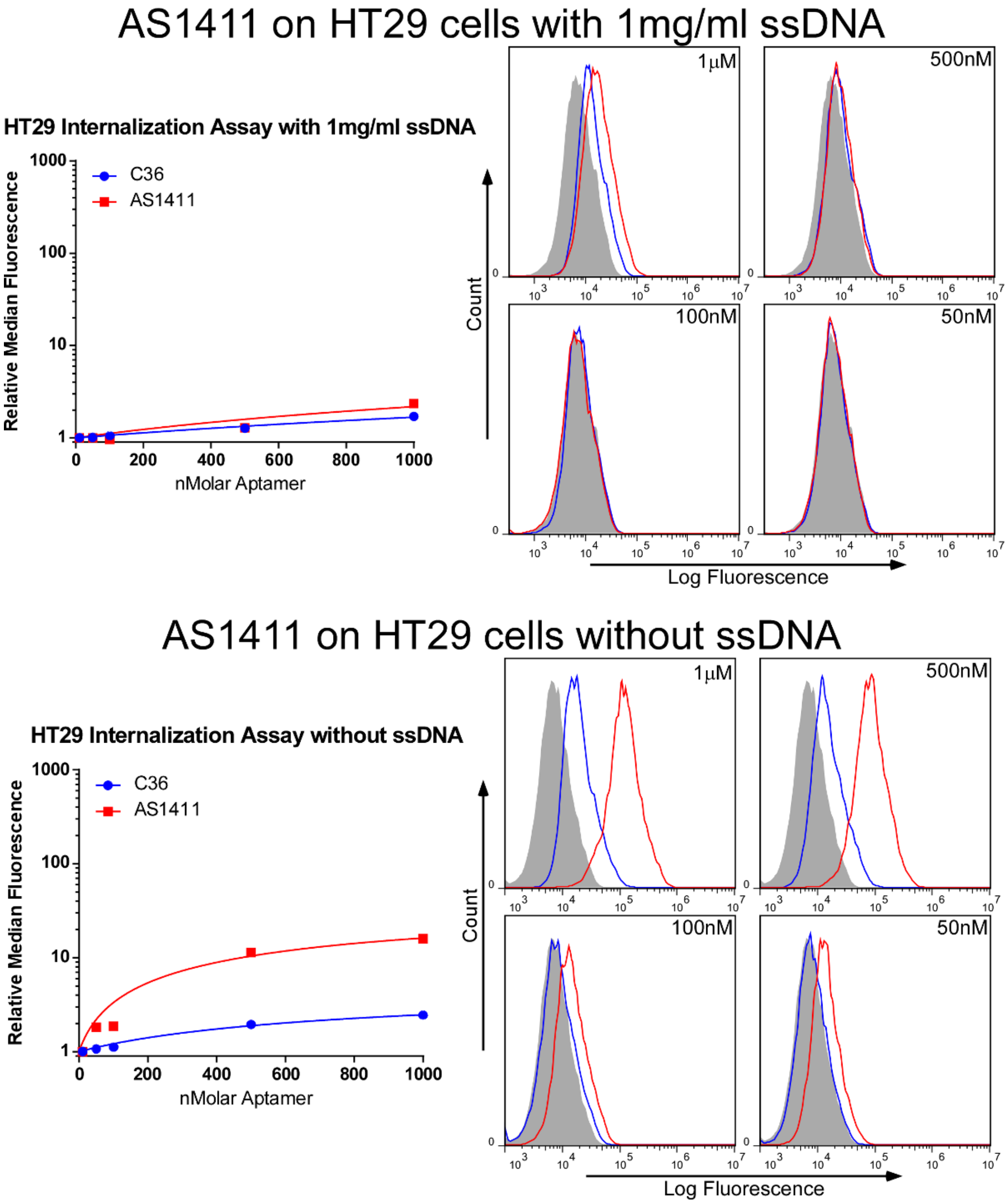

Supplementary Fig. 70. Nucleolin targeted aptamer AS1411 internalization and binding assays on HT29 cells. Graphs represent the median fluorescence of the aptamer (Red) and C36 (Blue) relative to unstained cells (Gray).

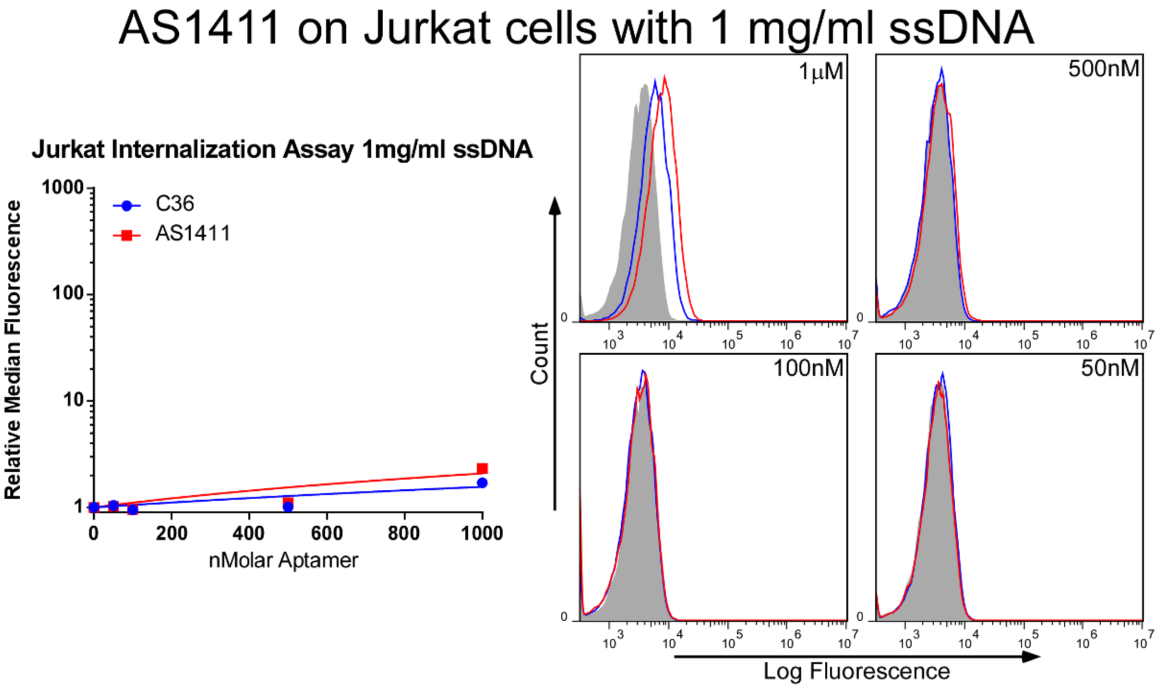

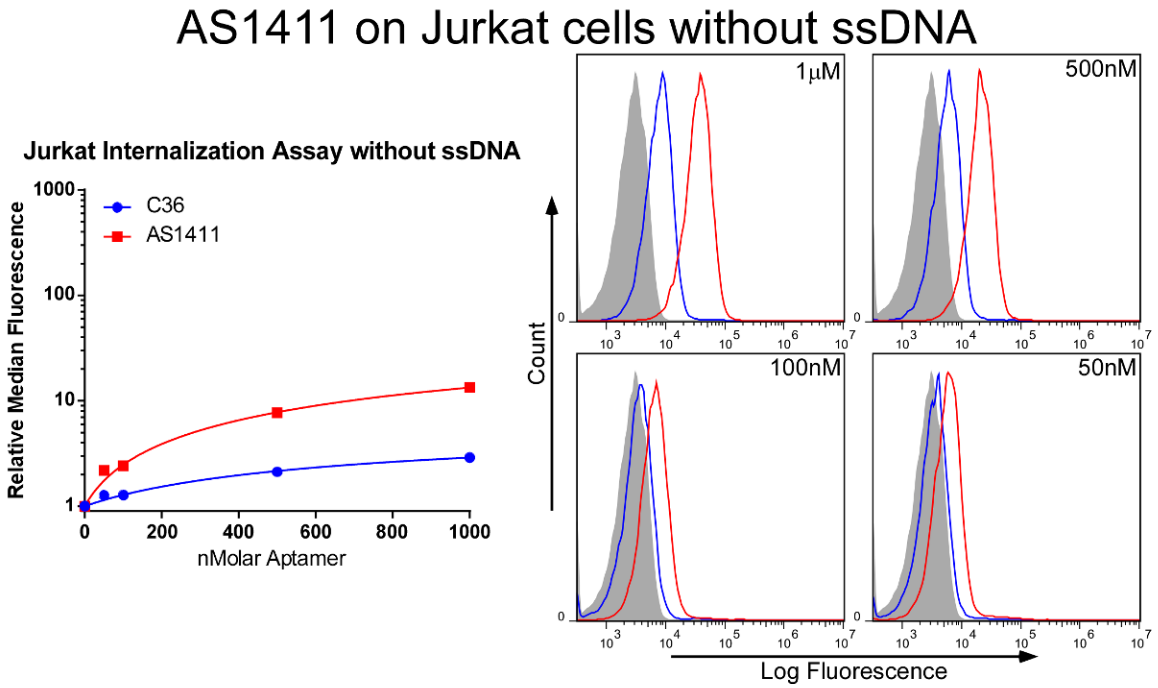

Supplementary Fig. 71. Nucleolin targeted aptamer AS1411 internalization and binding assays on Jurkat cells. Graphs represent the median fluorescence of the aptamer (Red) and C36 (Blue) relative to unstained cells (Gray).

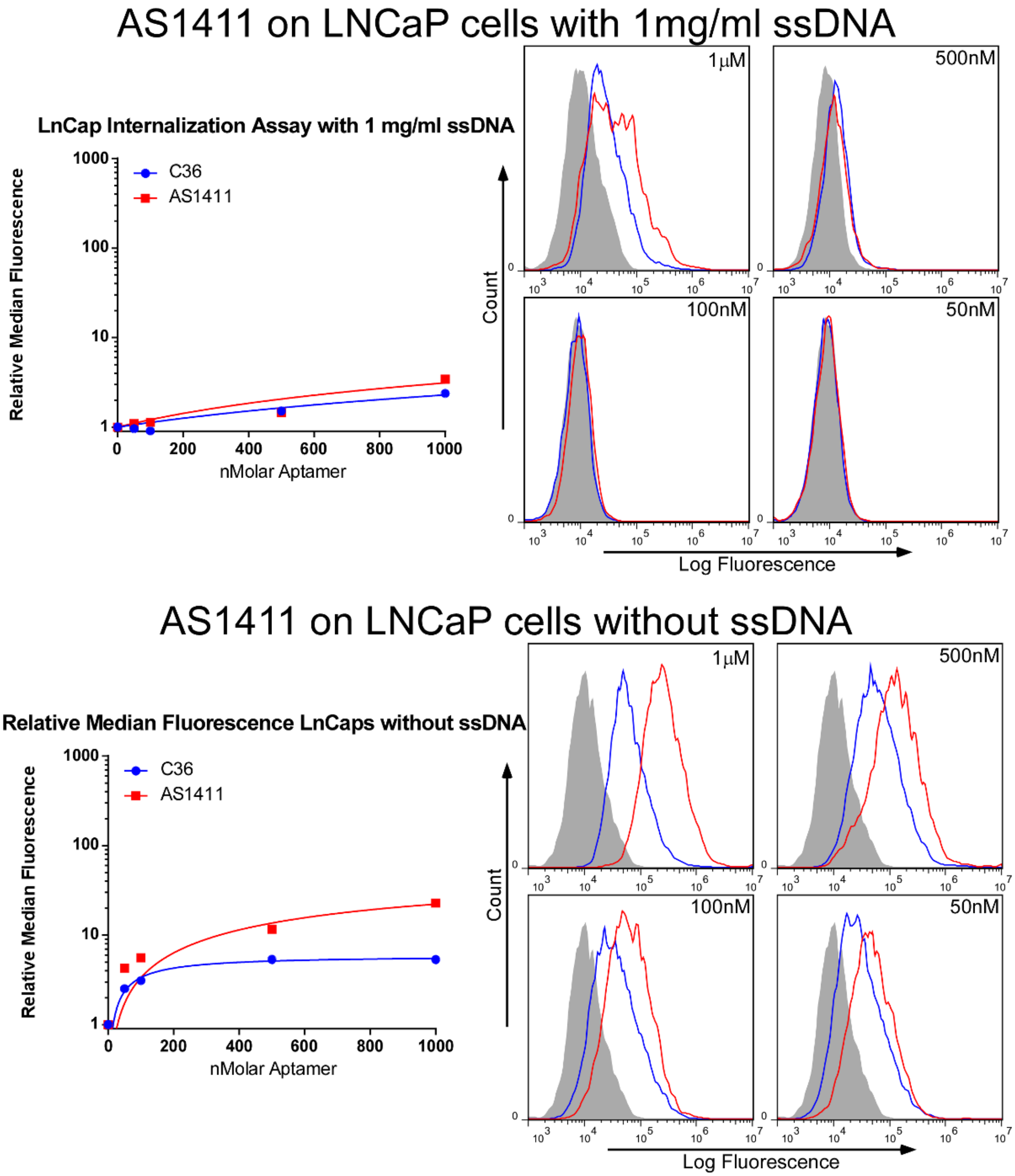

Supplementary Fig. 72. Nucleolin targeted aptamer AS1411 internalization and binding assays on LNCaP cells. Graphs represent the median fluorescence of the aptamer (Red) and C36 (Blue) relative to unstained cells (Gray).

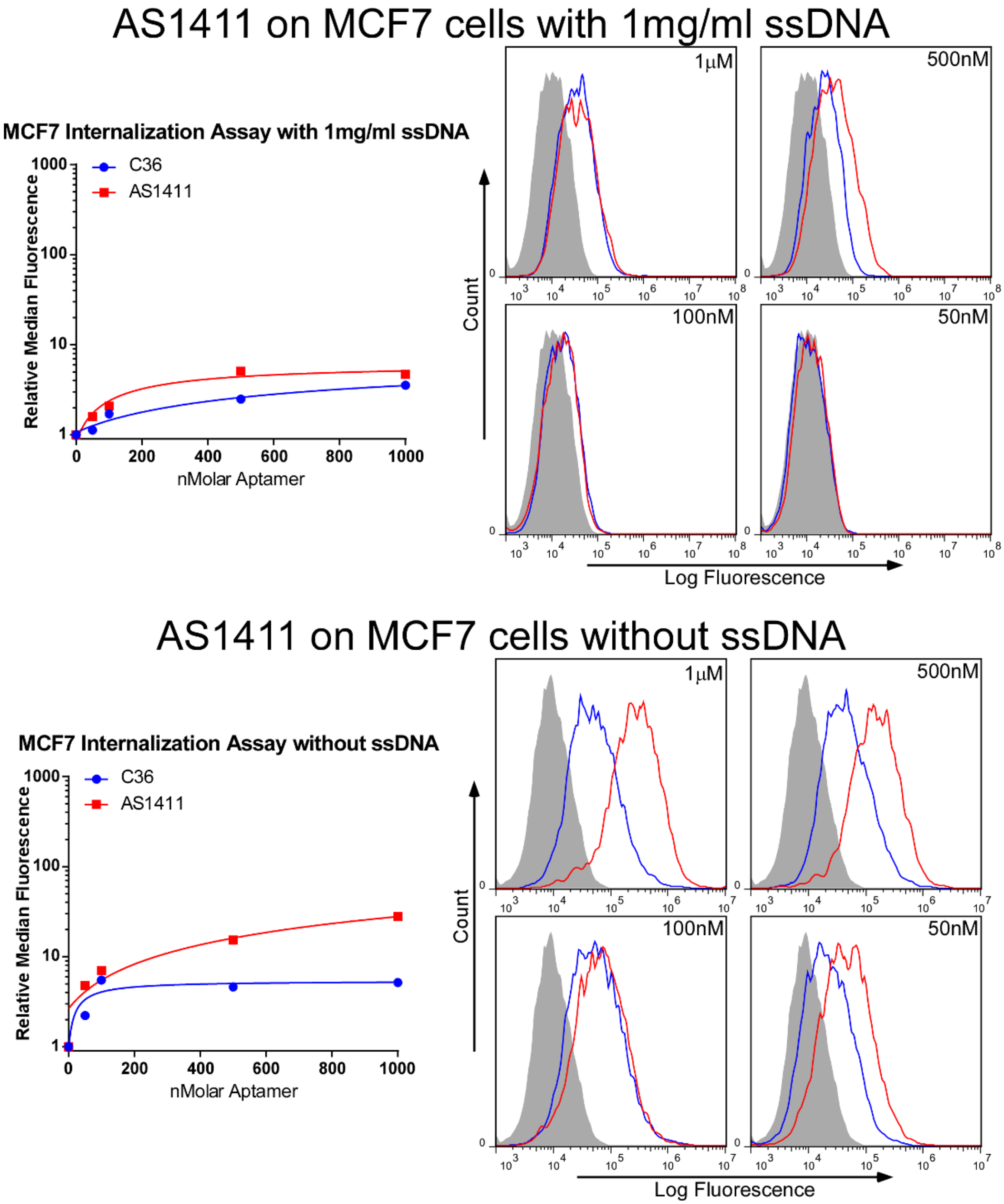

Supplementary Fig. 73. Nucleolin targeted aptamer AS1411 internalization and binding assays on MCF7 cells. Graphs represent the median fluorescence of the aptamer (Red) and C36 (Blue) relative to unstained cells (Gray).

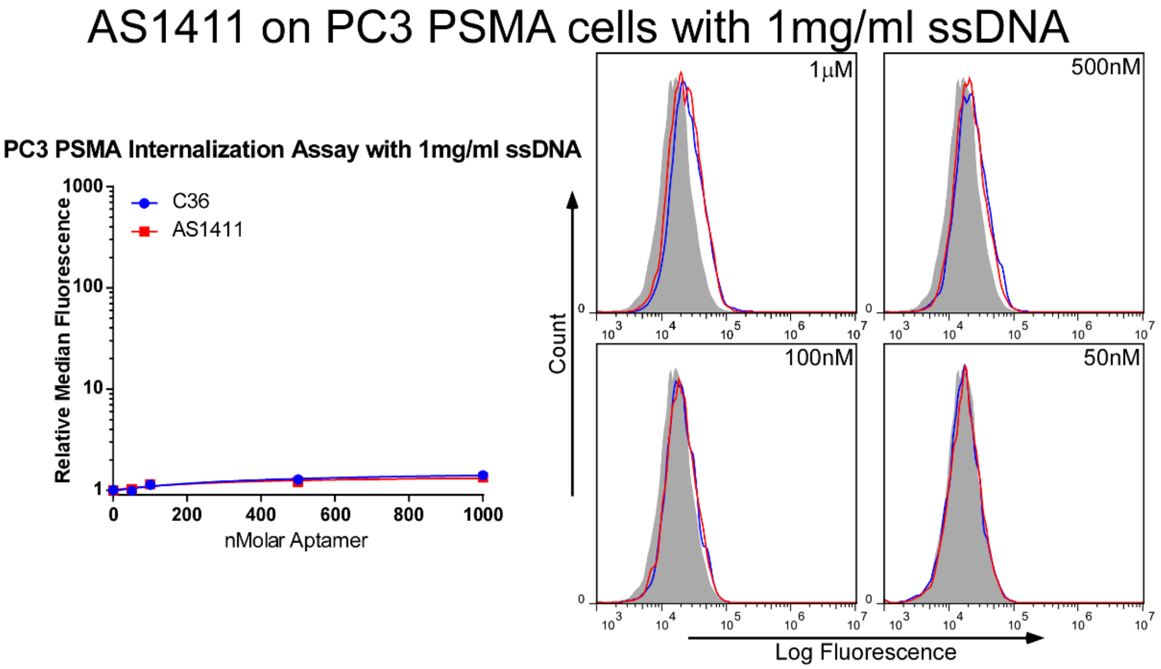

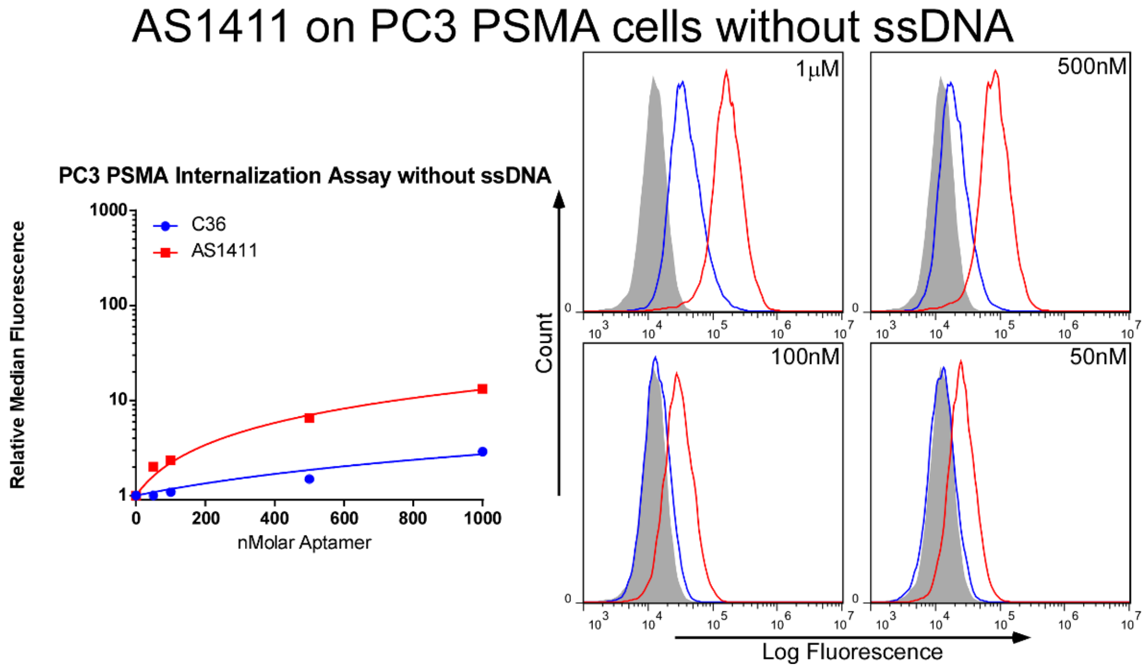

Supplementary Fig. 74. Nucleolin targeted aptamer AS1411 internalization and binding assays on PC3 PSMA cells. Graphs represent the median fluorescence of the aptamer (Red) and C36 (Blue) relative to unstained cells (Gray).

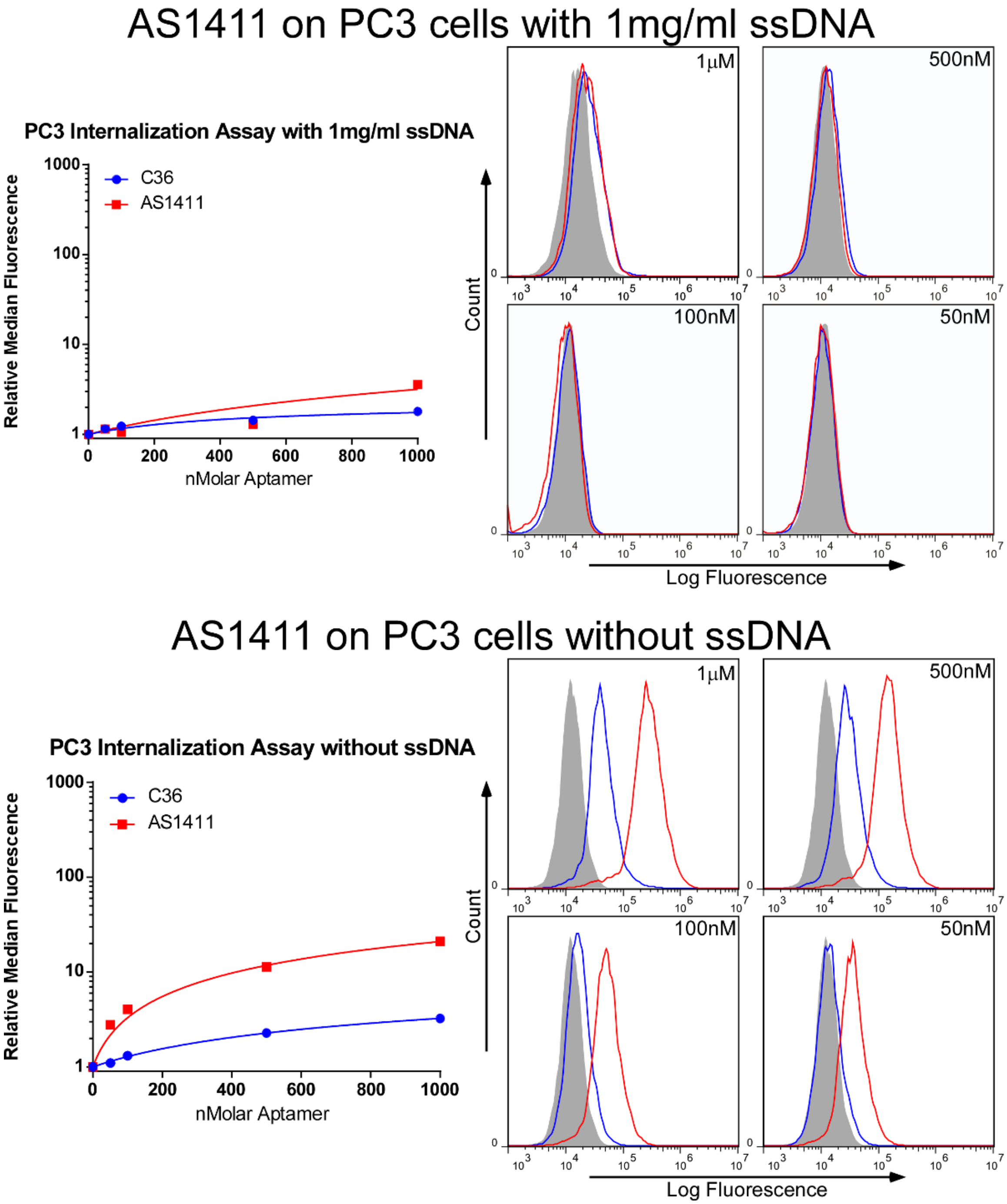

Supplementary Fig. 75. Nucleolin targeted aptamer AS1411 internalization and binding assays on PC3 cells. Graphs represent the median fluorescence of the aptamer (Red) and C36 (Blue) relative to unstained cells (Gray).

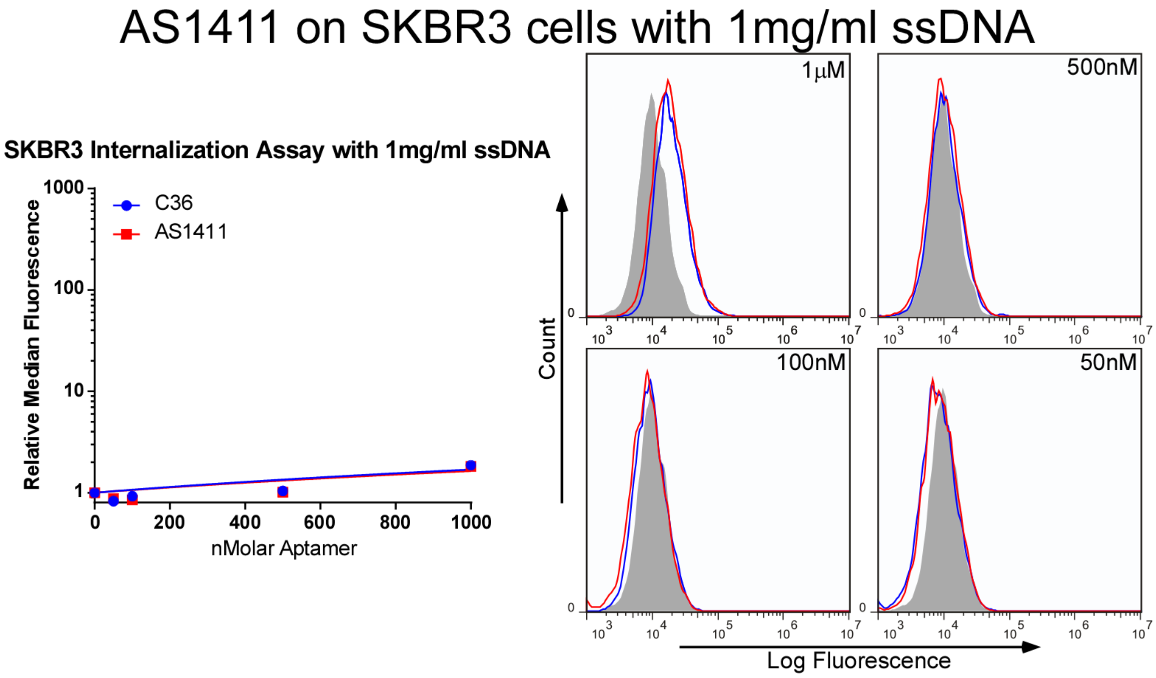

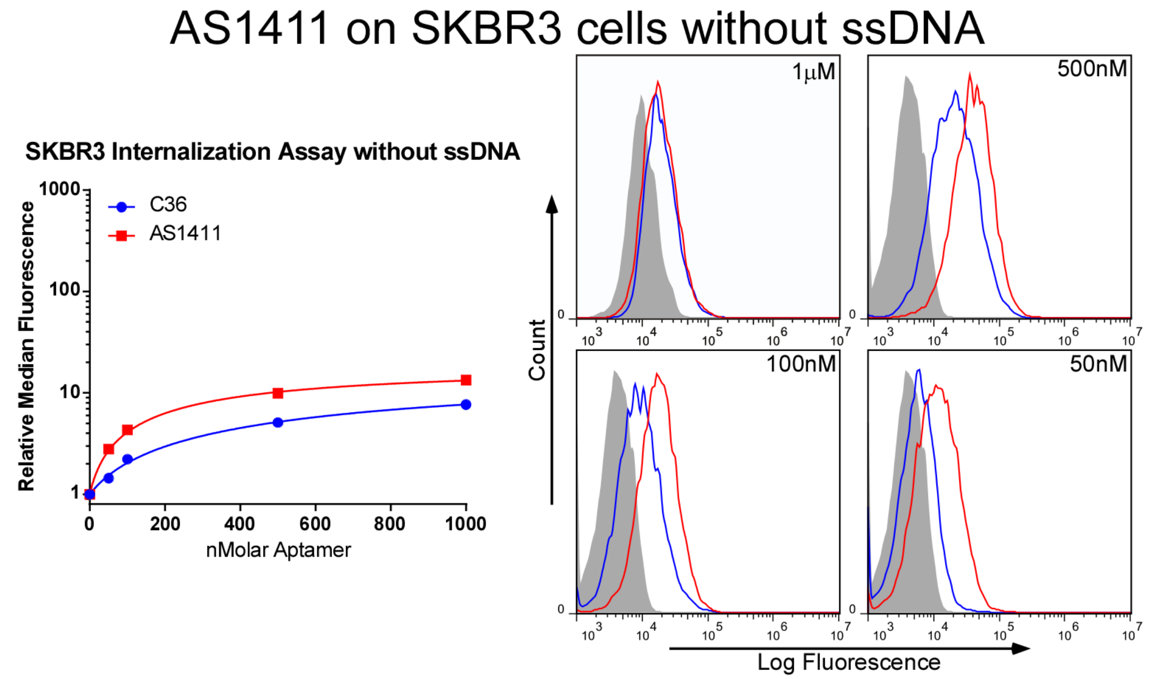

Supplementary Fig. 76. Nucleolin targeted aptamer AS1411 internalization and binding assays on SKBR3 cells. Graphs represent the median fluorescence of the aptamer (Red) and C36 (Blue) relative to unstained cells (Gray).

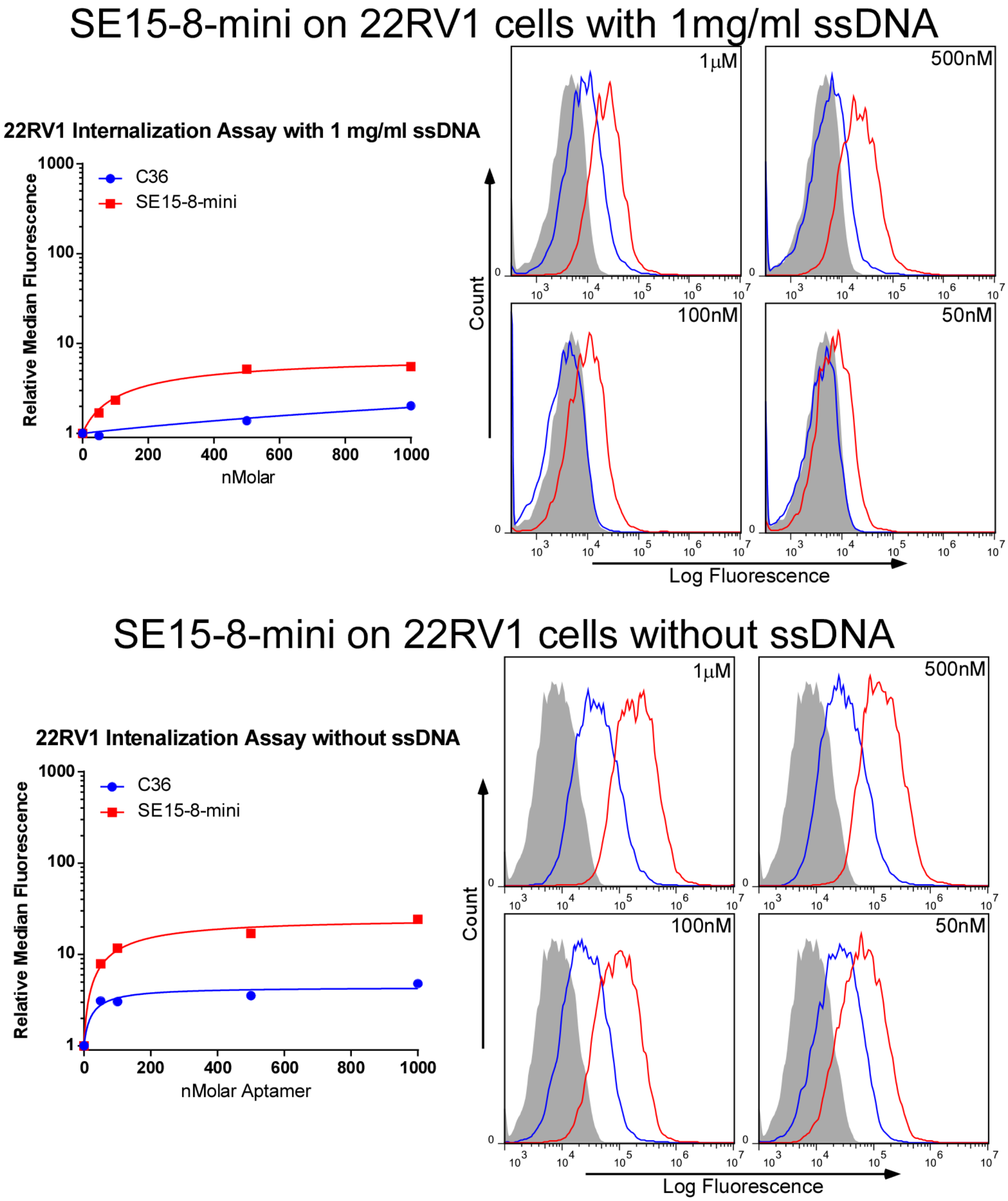

Supplementary Fig. 77. HER2 targeted aptamer SE15-8-mini internalization and binding assays on 22RV1 cells. Graphs represent the median fluorescence of the aptamer (Red) and C36 (Blue) relative to unstained cells (Gray).

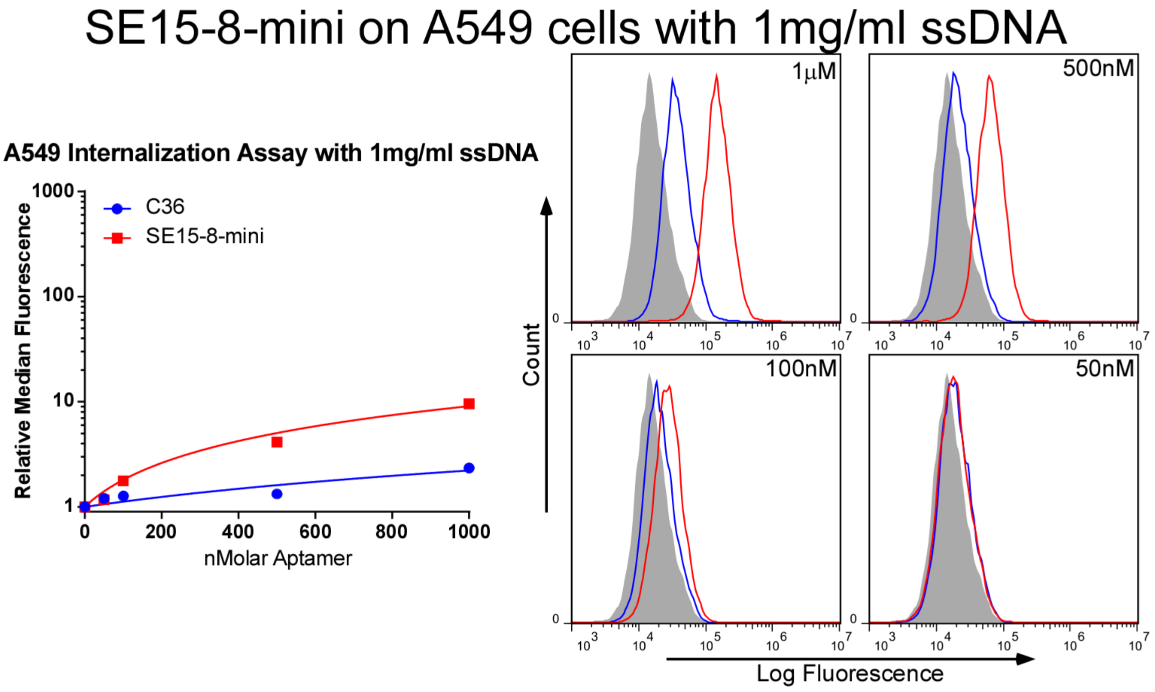

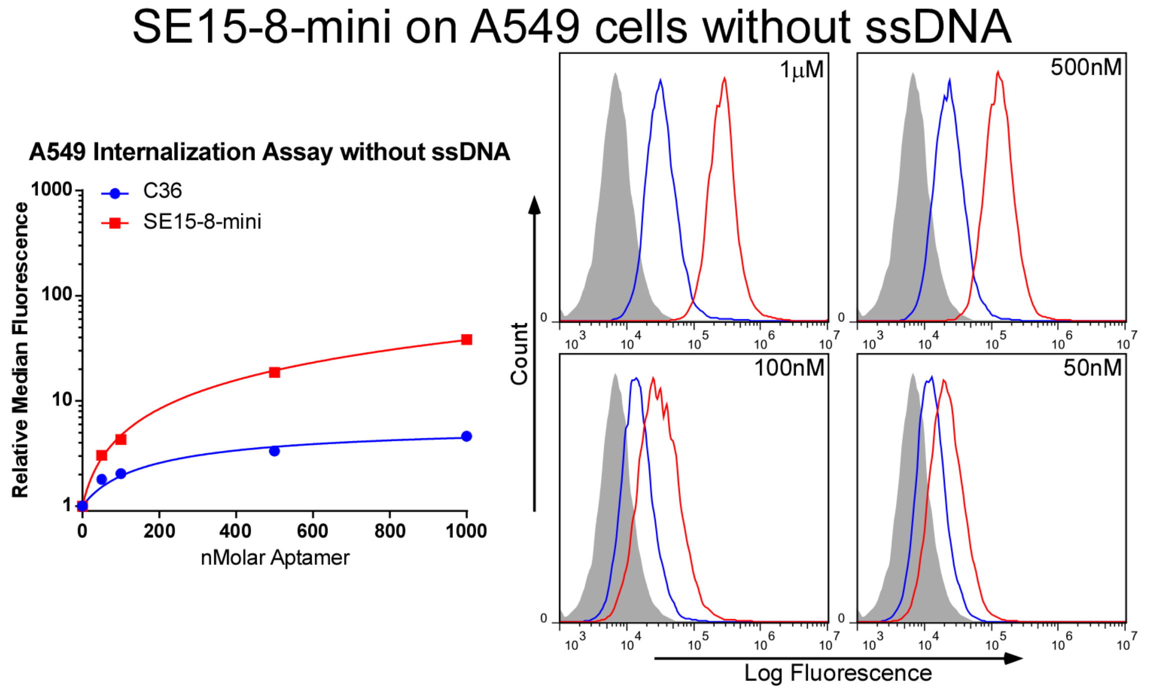

Supplementary Fig. 78. HER2 targeted aptamer SE15-8-mini internalization and binding assays on A549 cells. Graphs represent the median fluorescence of the aptamer (Red) and C36 (Blue) relative to unstained cells (Gray).

SE15-8-mini on HeLa PSMA cells with 1mg/ml ssDNA

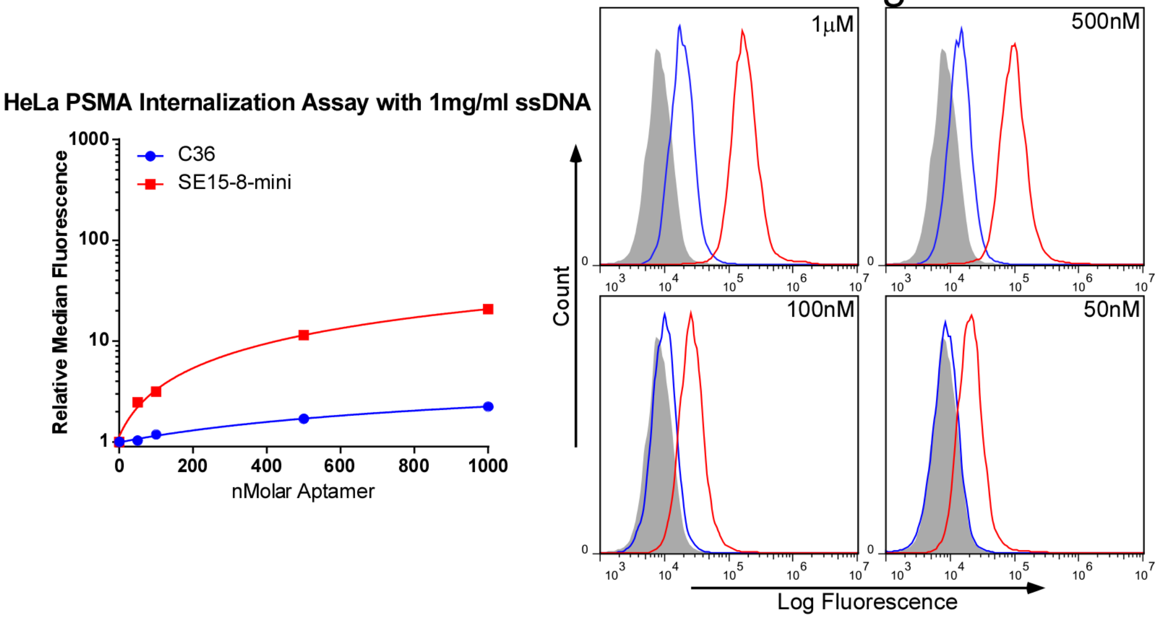

SE15-8-mini on HeLa PSMA cells without ssDNA

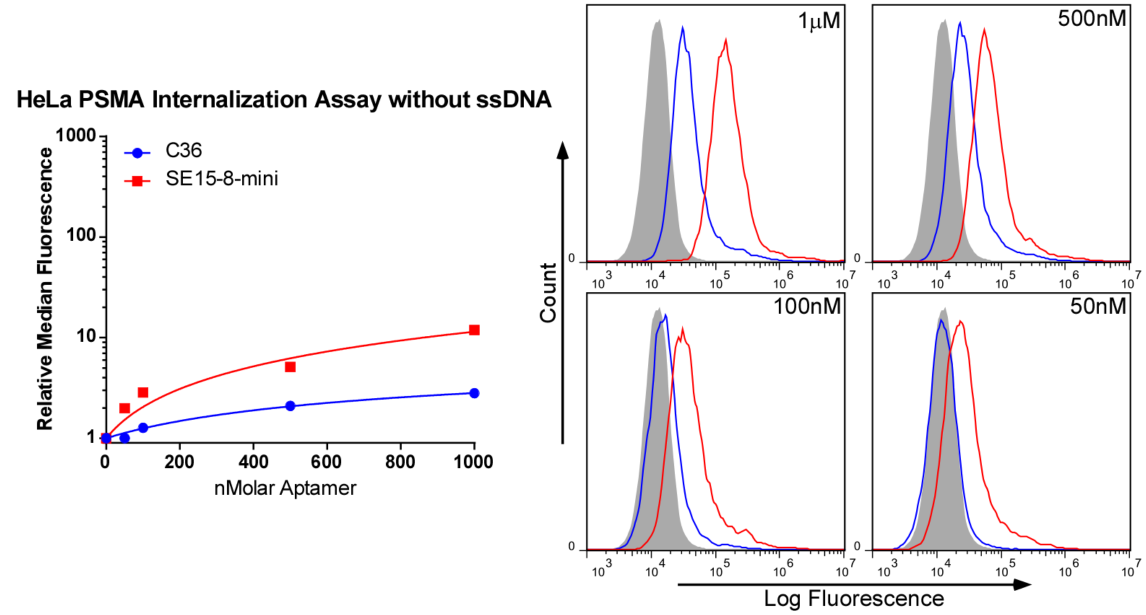

Supplementary Fig. 79. HER2 targeted aptamer SE15-8-mini internalization and binding assays on HeLa PSMA cells. Graphs represent the median fluorescence of the aptamer (Red) and C36 (Blue) relative to unstained cells (Gray).

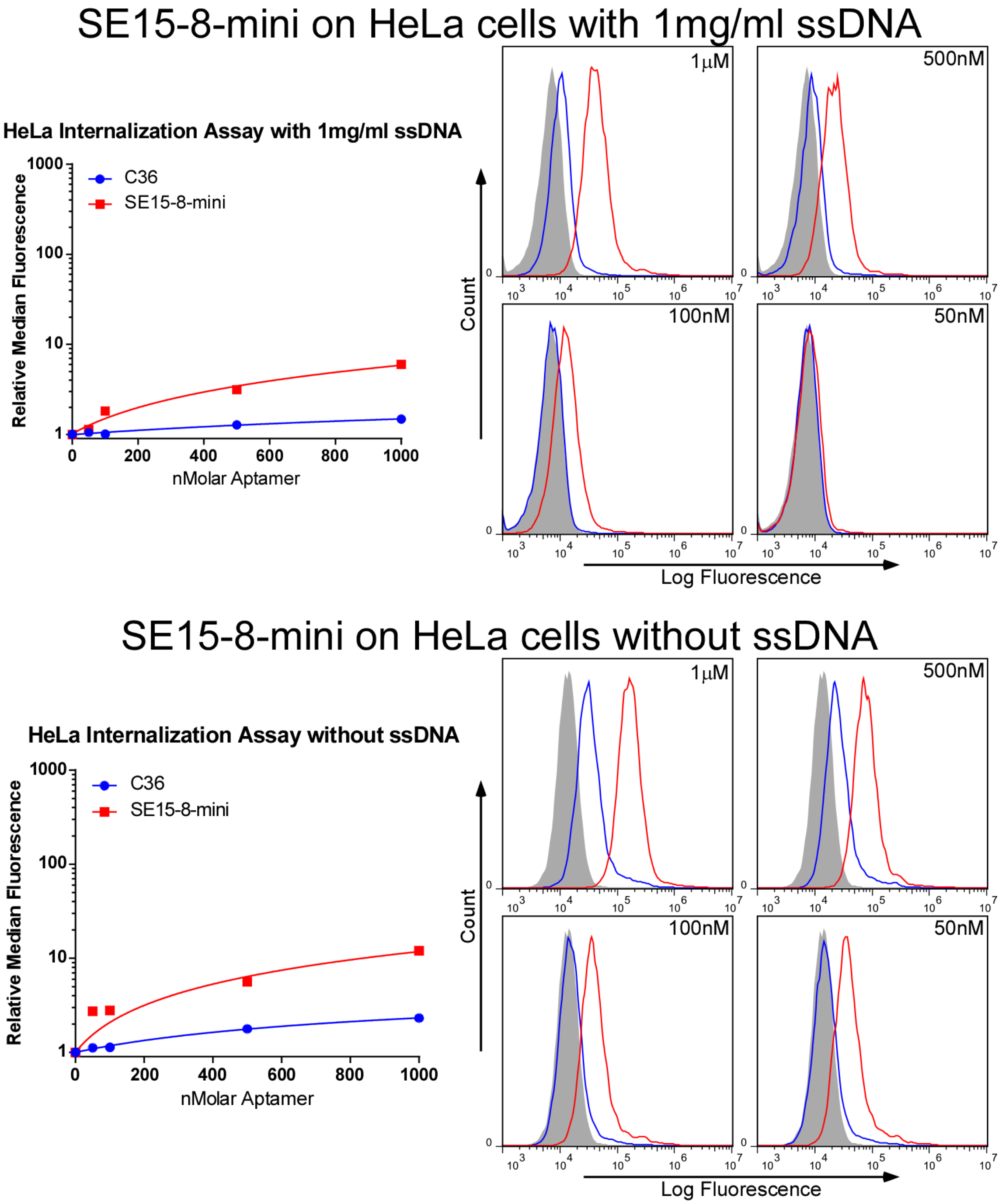

Supplementary Fig. 80. HER2 targeted aptamer SE15-8-mini internalization and binding assays on HeLa cells. Graphs represent the median fluorescence of the aptamer (Red) and C36 (Blue) relative to unstained cells (Gray).

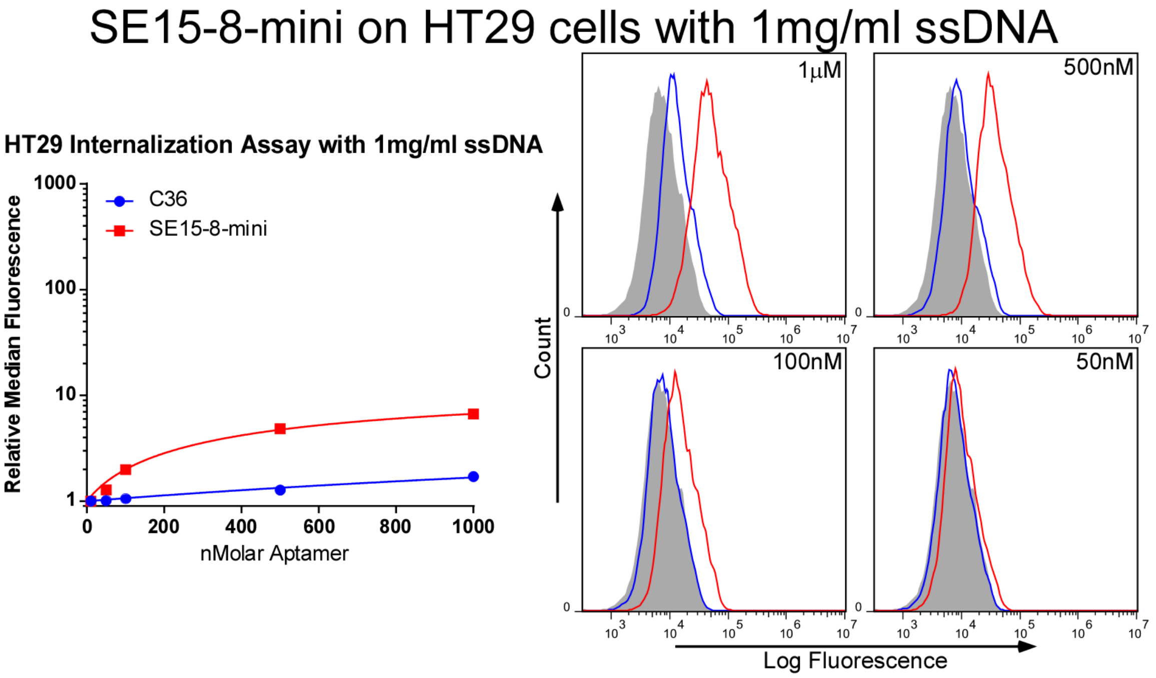

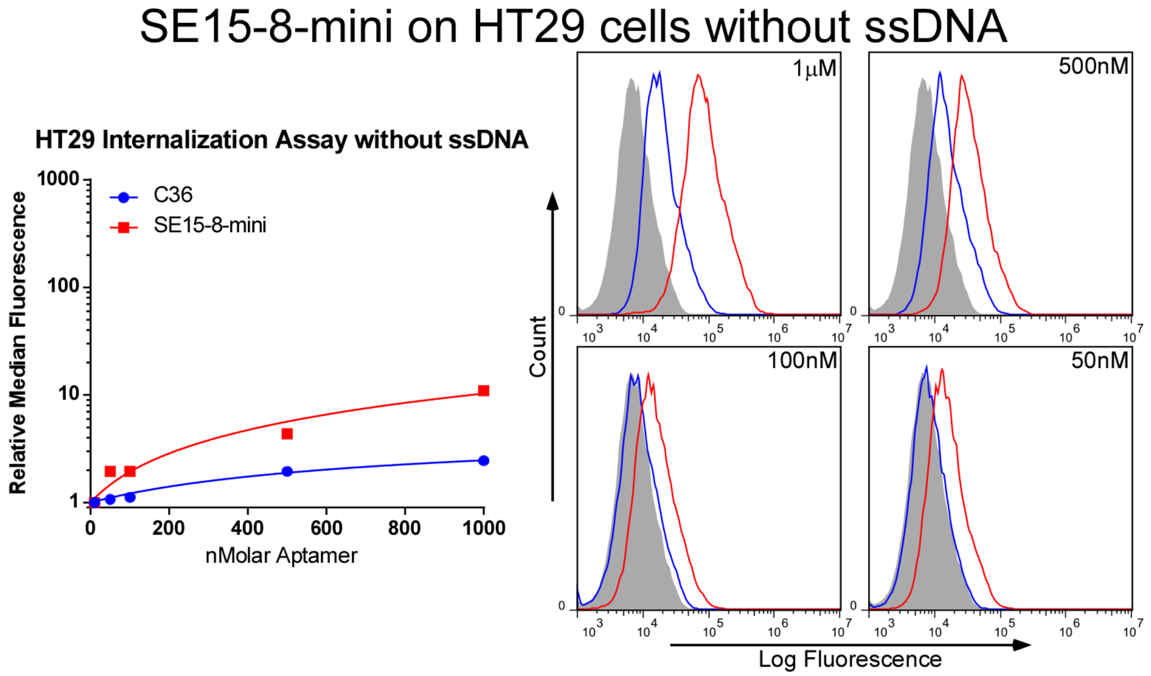

Supplementary Fig. 81. HER2 targeted aptamer SE15-8-mini internalization and binding assays on HT29 cells. Graphs represent the median fluorescence of the aptamer (Red) and C36 (Blue) relative to unstained cells (Gray).

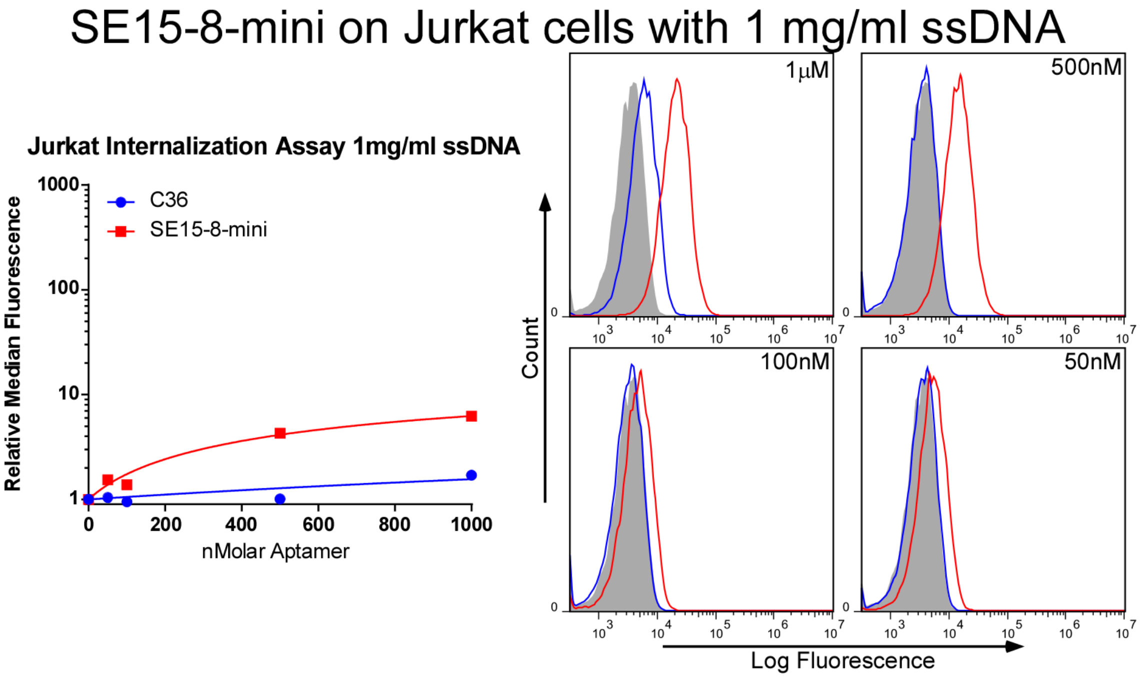

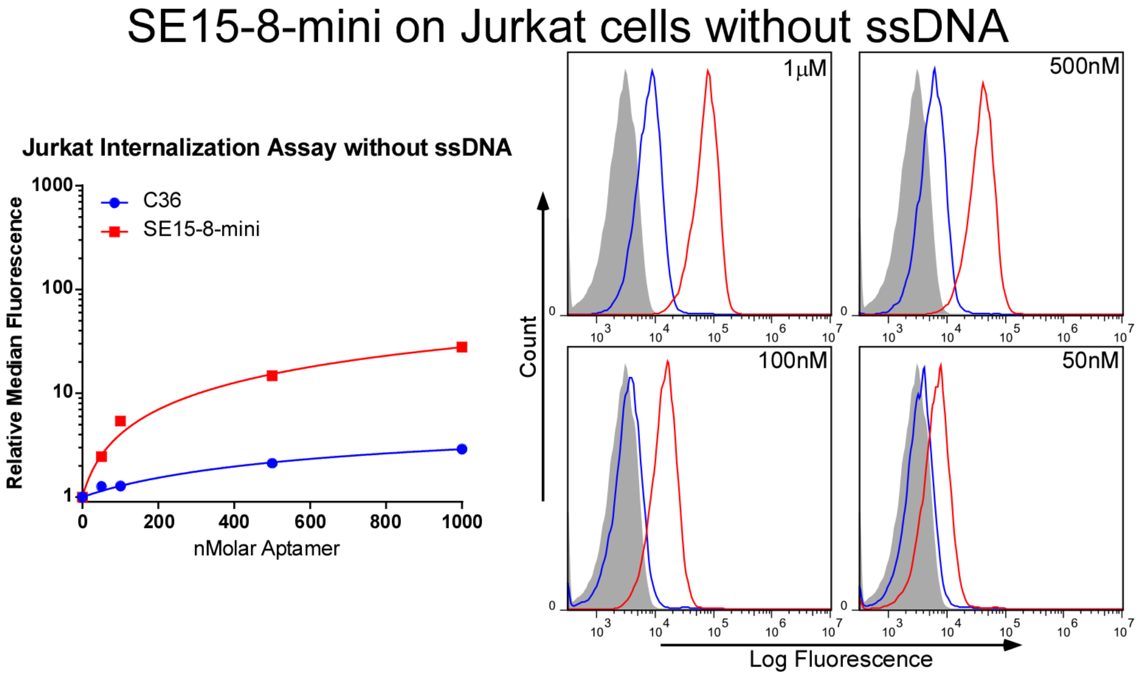

Supplementary Fig. 82. HER2 targeted aptamer SE15-8-mini internalization and binding assays on Jurkat cells. Graphs represent the median fluorescence of the aptamer (Red) and C36 (Blue) relative to unstained cells (Gray).

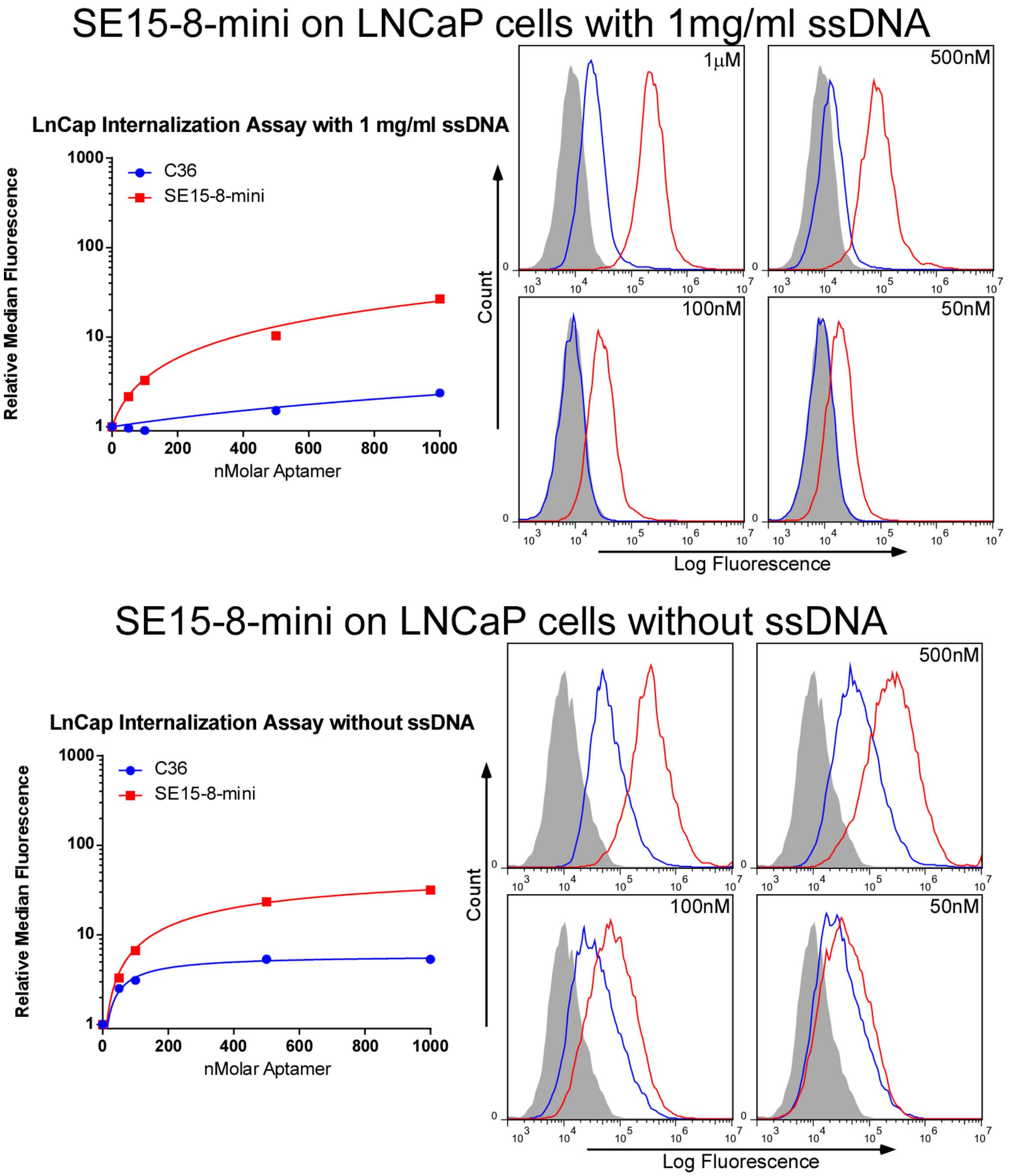

Supplementary Fig. 83. HER2 targeted aptamer SE15-8-mini internalization and binding assays on LNCaP cells. Graphs represent the median fluorescence of the aptamer (Red) and C36 (Blue) relative to unstained cells (Gray).

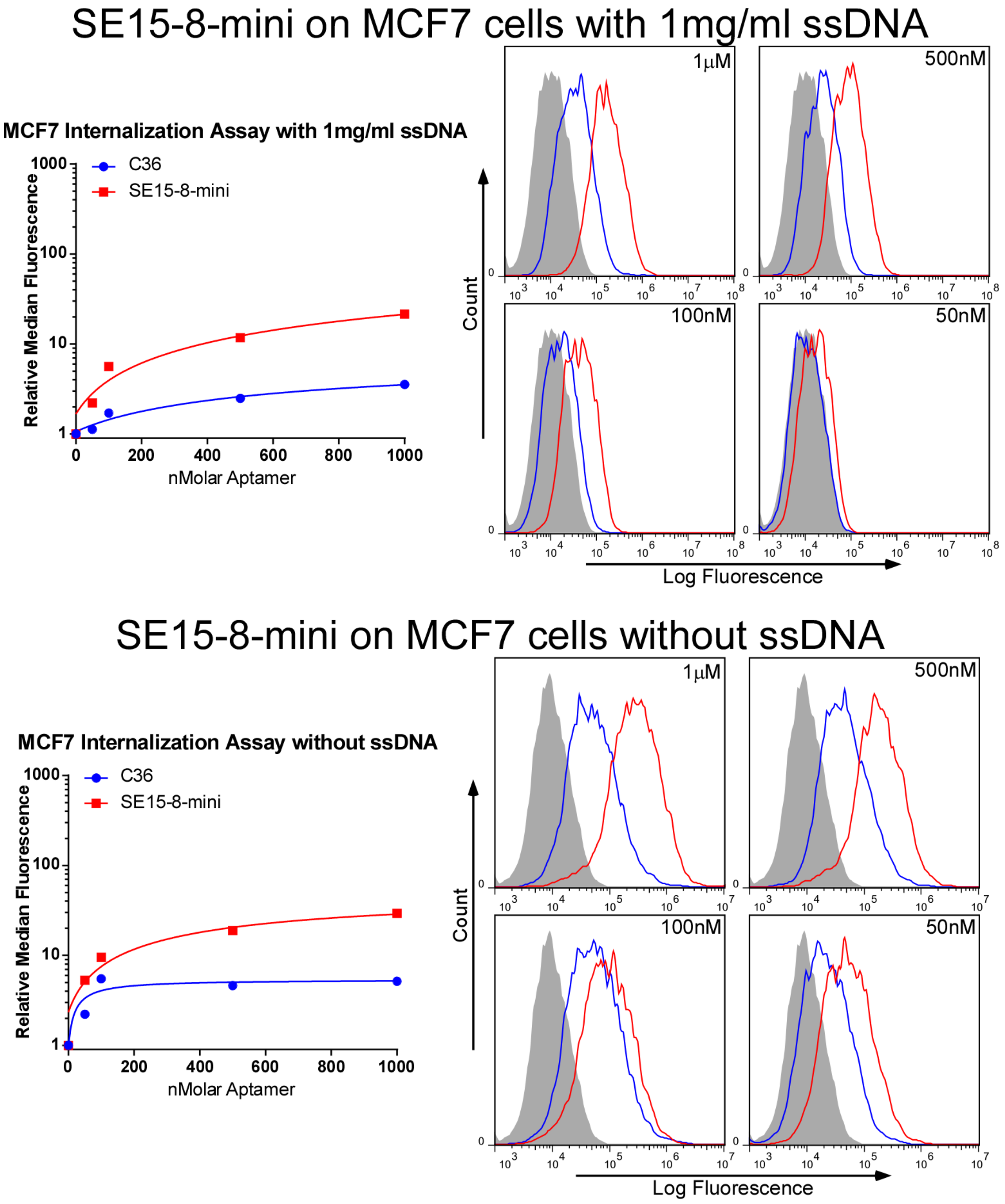

Supplementary Fig. 84. HER2 targeted aptamer SE15-8-mini internalization and binding assays on MCF7 cells. Graphs represent the median fluorescence of the aptamer (Red) and C36 (Blue) relative to unstained cells (Gray).

SE15-8-mini on PC3 PSMA cells with 1mg/ml ssDNA

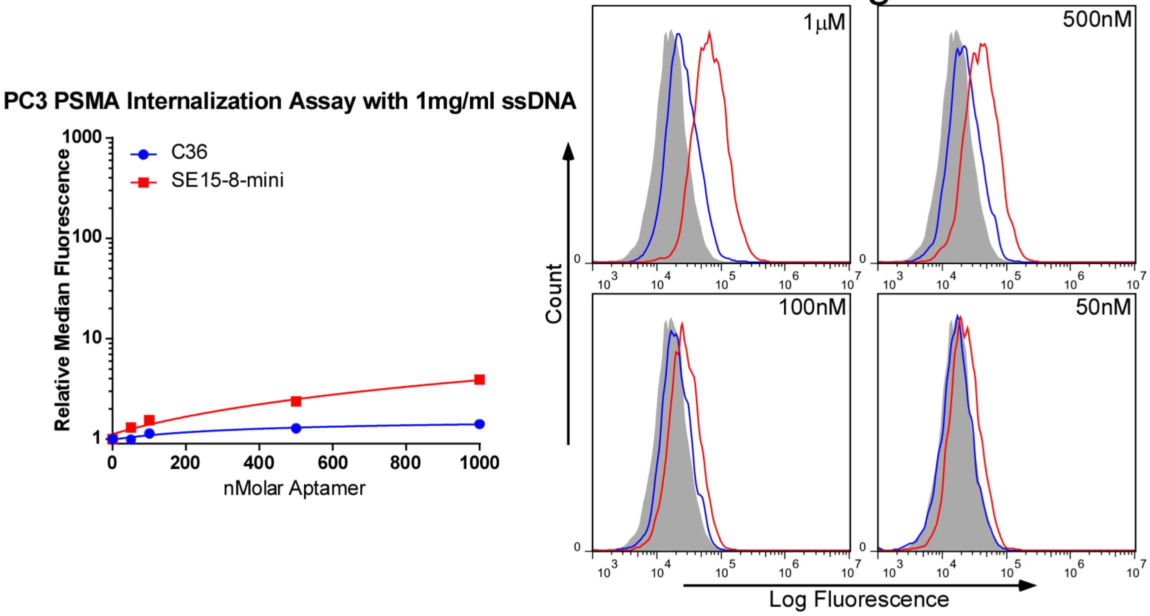

SE15-8-mini on PC3 PSMA cells without ssDNA

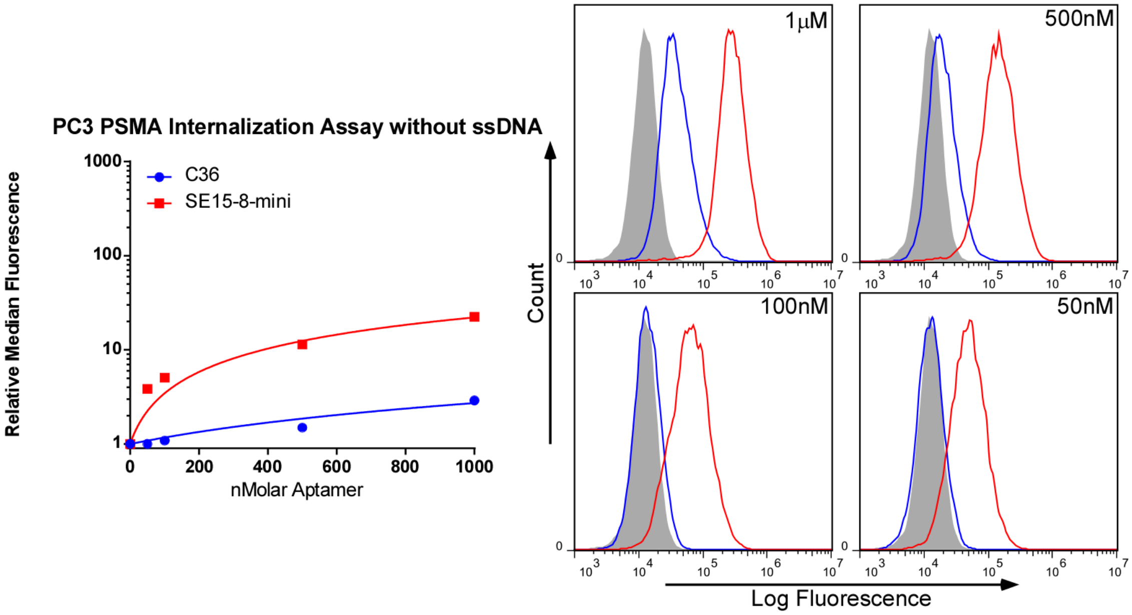

Supplementary Fig. 85. HER2 targeted aptamer SE15-8-mini internalization and binding assays on PC3 PSMA cells. Graphs represent the median fluorescence of the aptamer (Red) and C36 (Blue) relative to unstained cells (Gray).

SE15-8-mini on PC3 cells with 1mg/ml ssDNA

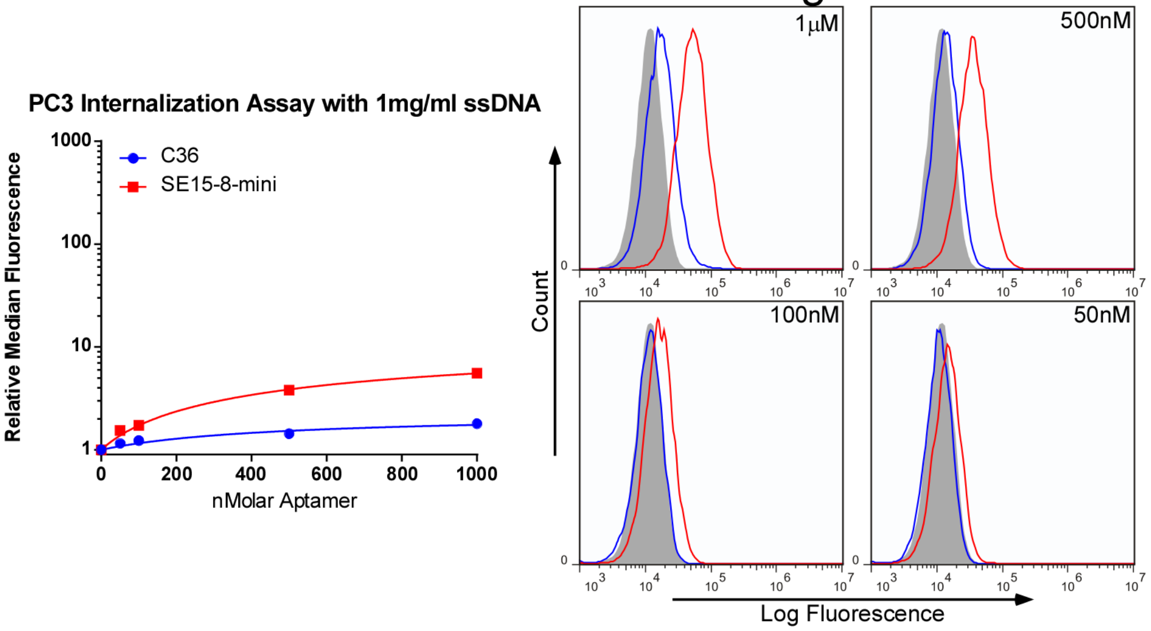

SE15-8-mini on PC3 cells without ssDNA

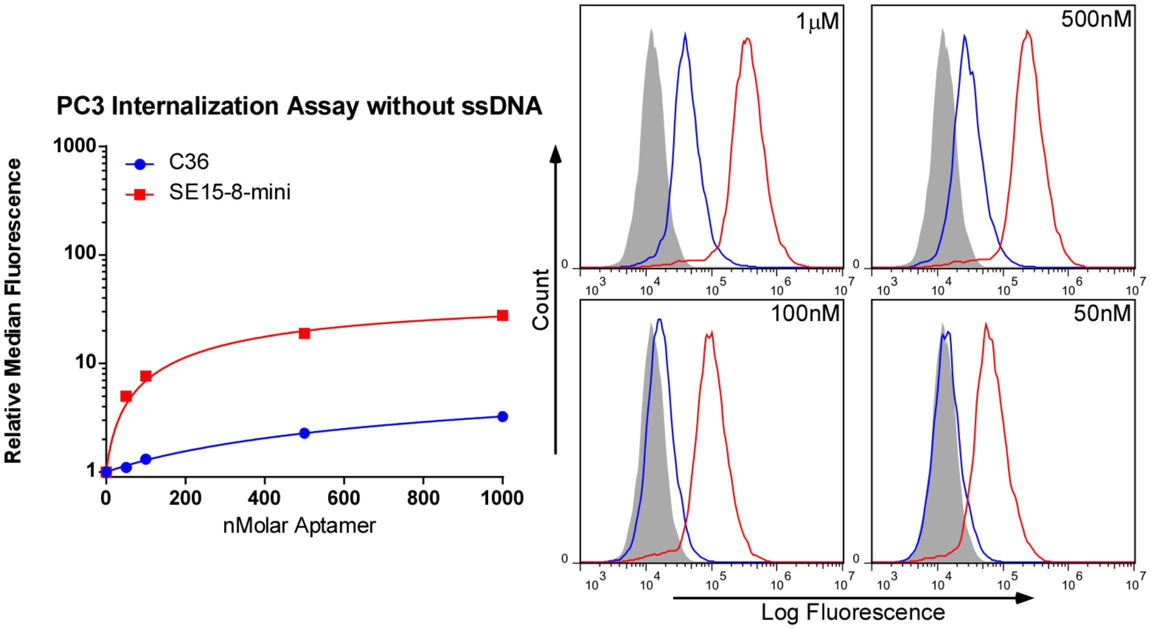

Supplementary Fig. 86. HER2 targeted aptamer SE15-8-mini internalization and binding assays on PC3 cells. Graphs represent the median fluorescence of the aptamer (Red) and C36 (Blue) relative to unstained cells (Gray).

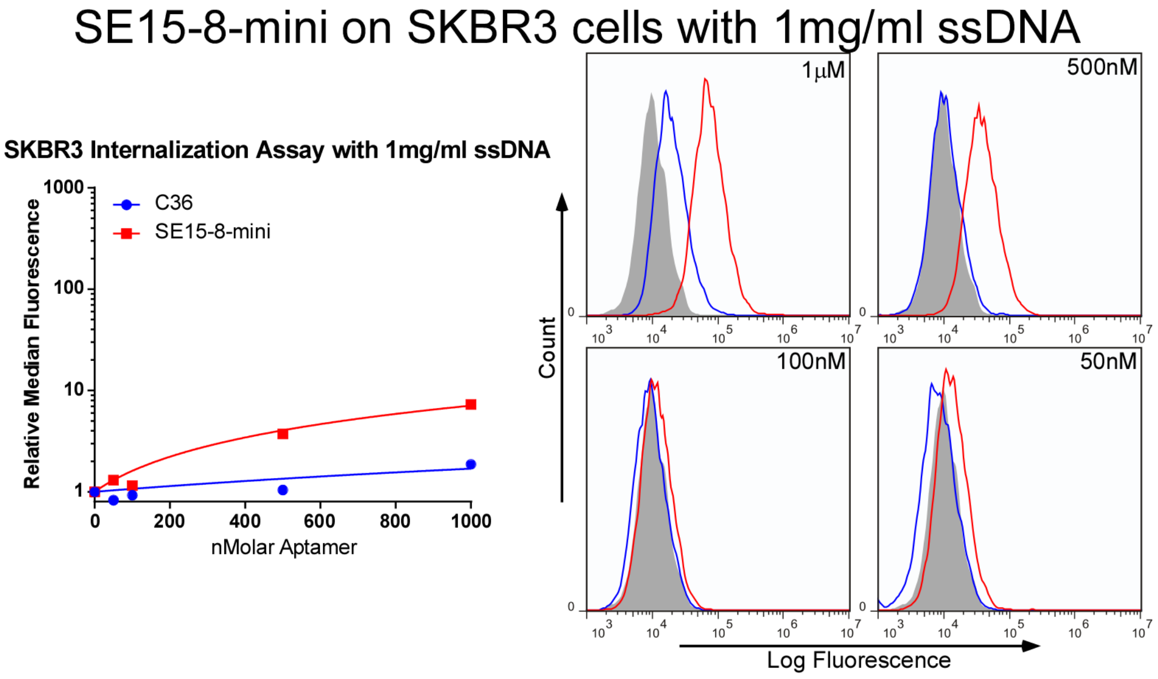

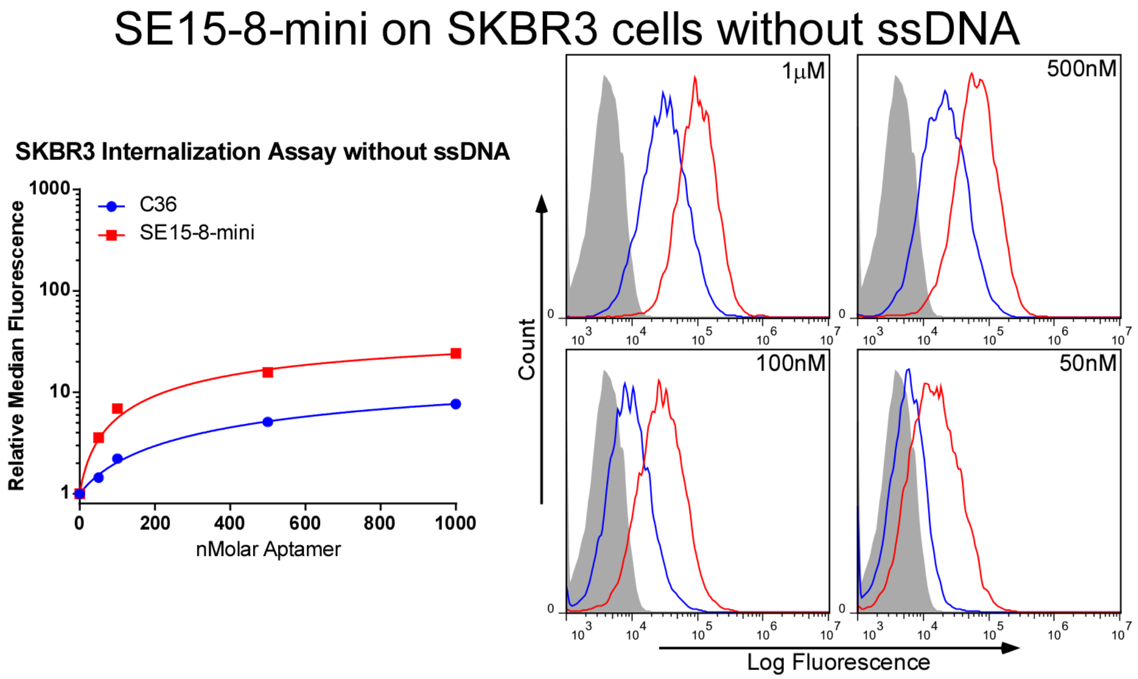

Supplementary Fig. 87. HER2 targeted aptamer SE15-8-mini internalization and binding assays on SKBR3 cells. Graphs represent the median fluorescence of the aptamer (Red) and C36 (Blue) relative to unstained cells (Gray).

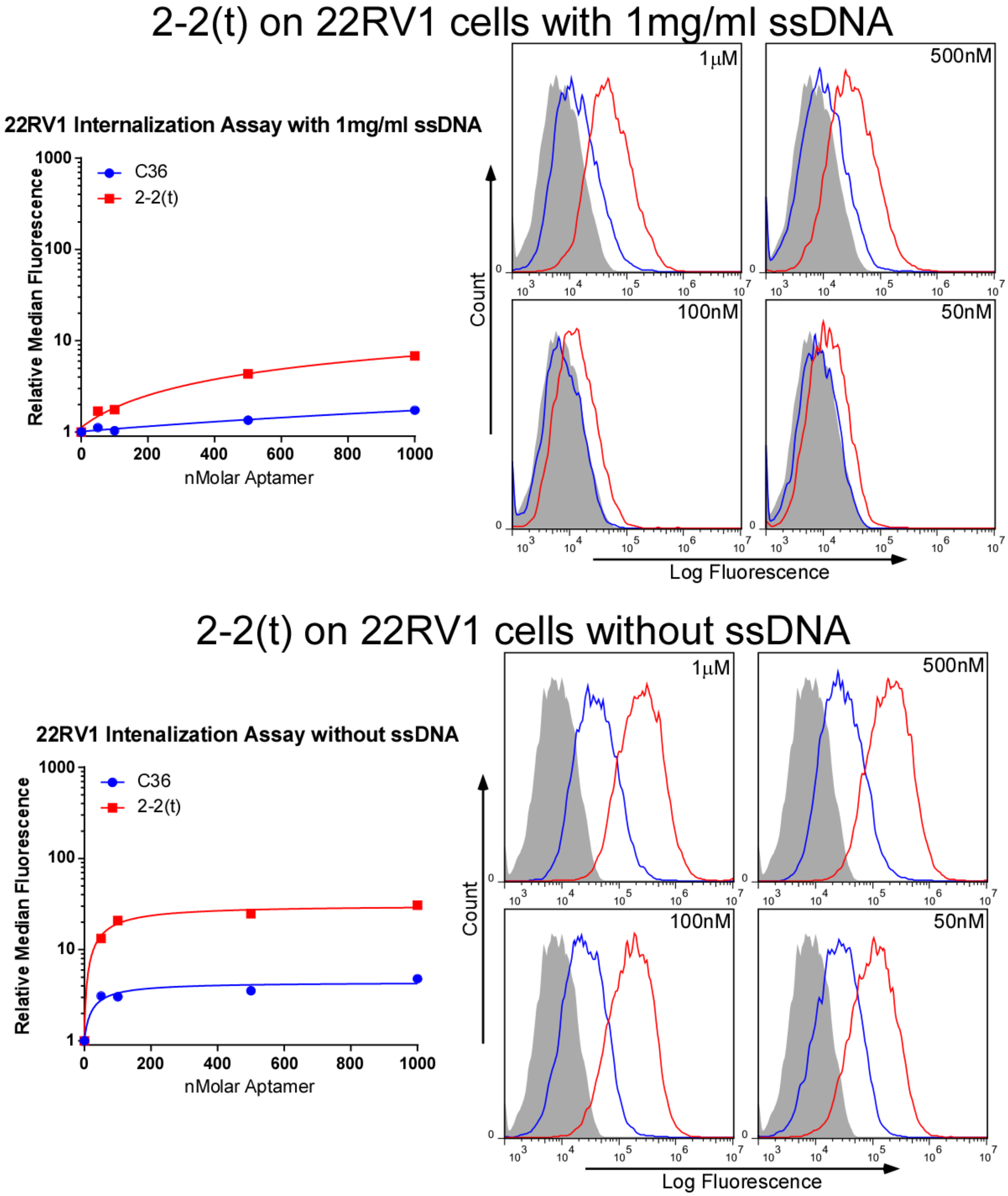

Supplementary Fig. 88. HER2 targeted aptamer 2-2(t) internalization and binding assays on 22RV1 cells. Graphs represent the median fluorescence of the aptamer (Red) and C36 (Blue) relative to unstained cells (Gray).

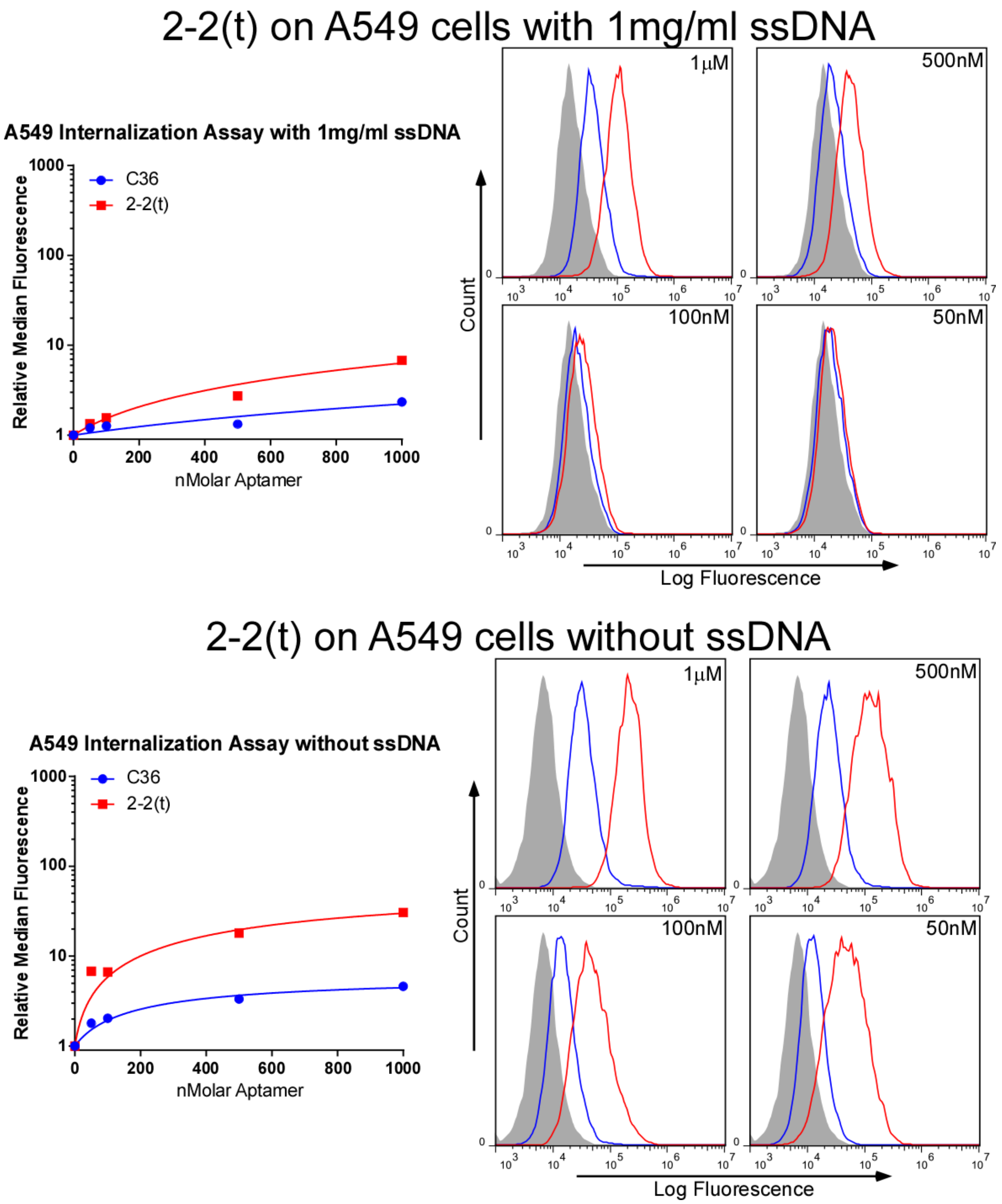

Supplementary Fig. 89. HER2 targeted aptamer 2-2(t) internalization and binding assays on A549 cells. Graphs represent the median fluorescence of the aptamer (Red) and C36 (Blue) relative to unstained cells (Gray).

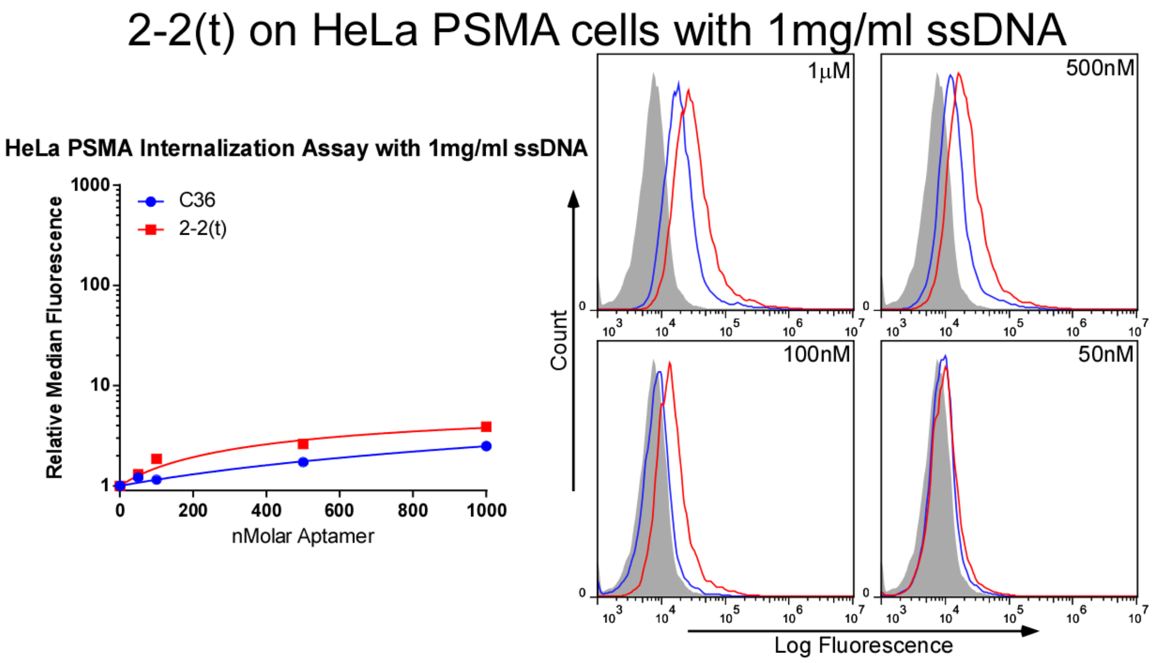

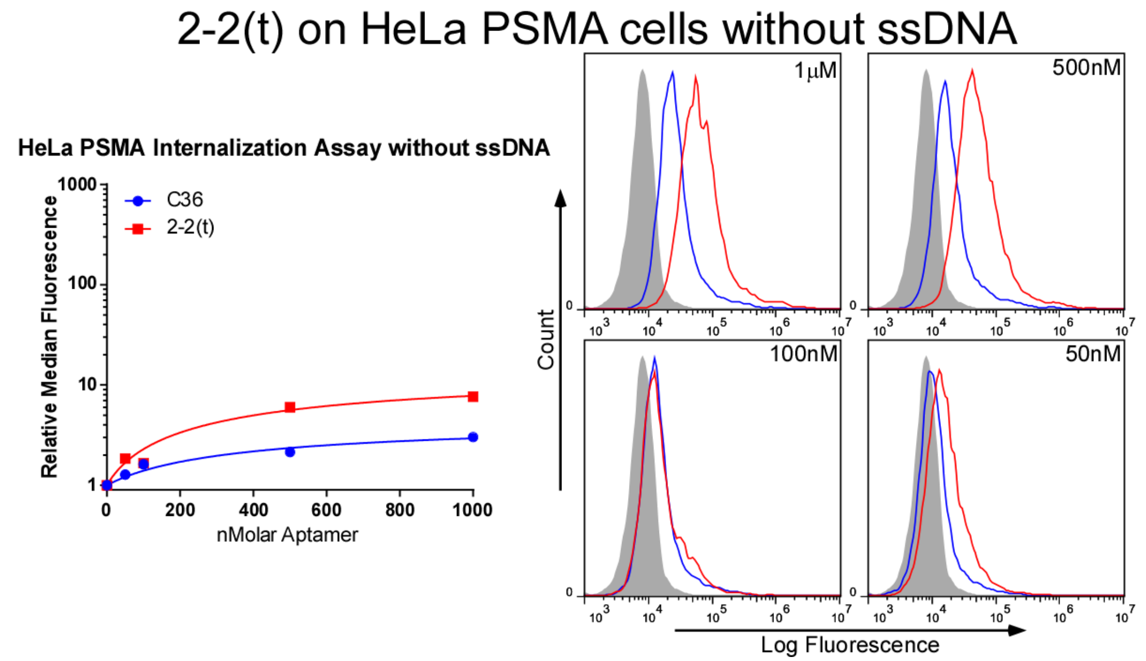

Supplementary Fig. 90. HER2 targeted aptamer 2-2(t) internalization and binding assays on HeLa PSMA cells. Graphs represent the median fluorescence of the aptamer (Red) and C36 (Blue) relative to unstained cells (Gray).

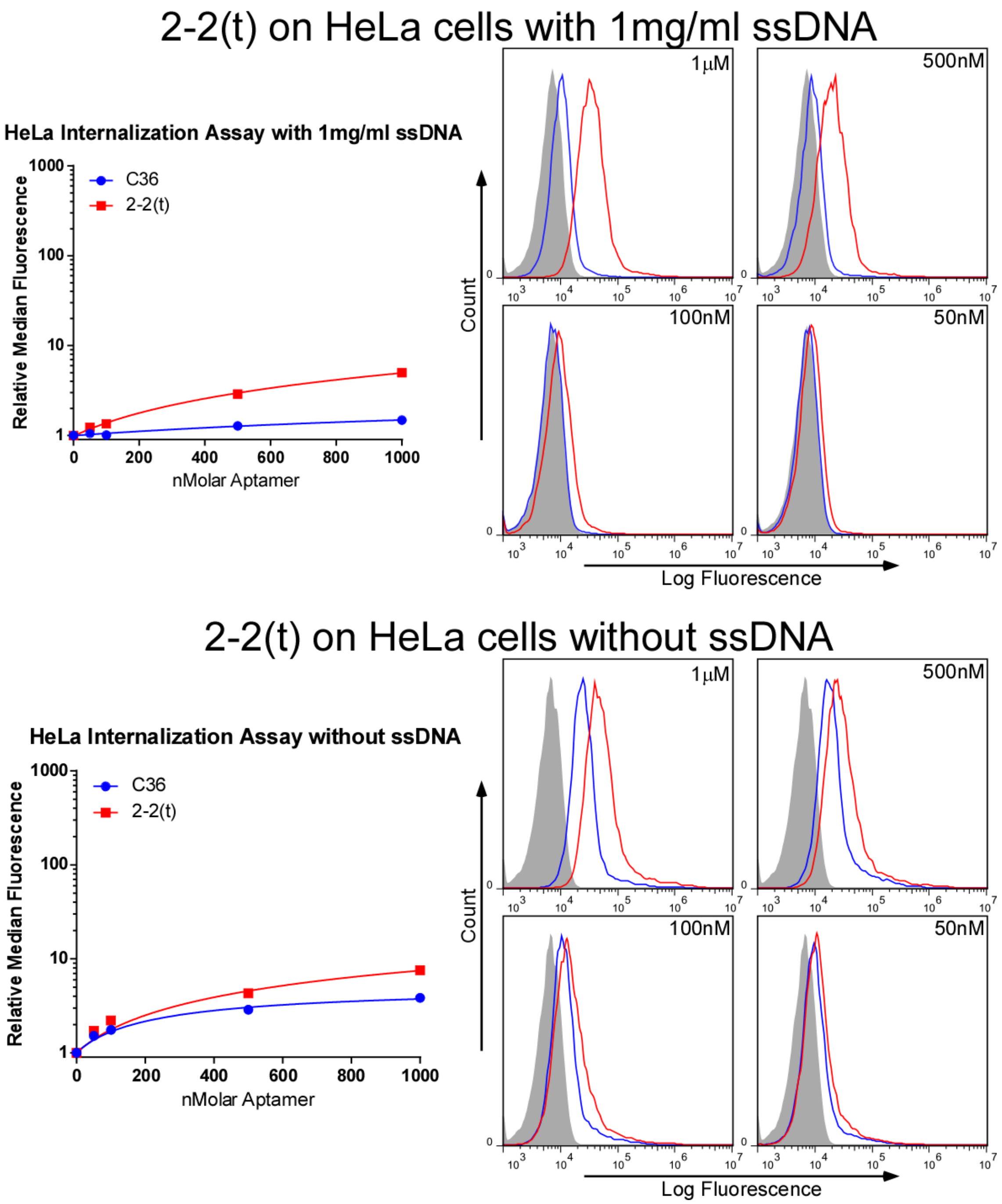

Supplementary Fig. 91. HER2 targeted aptamer 2-2(t) internalization and binding assays on HeLa cells. Graphs represent the median fluorescence of the aptamer (Red) and C36 (Blue) relative to unstained cells (Gray).

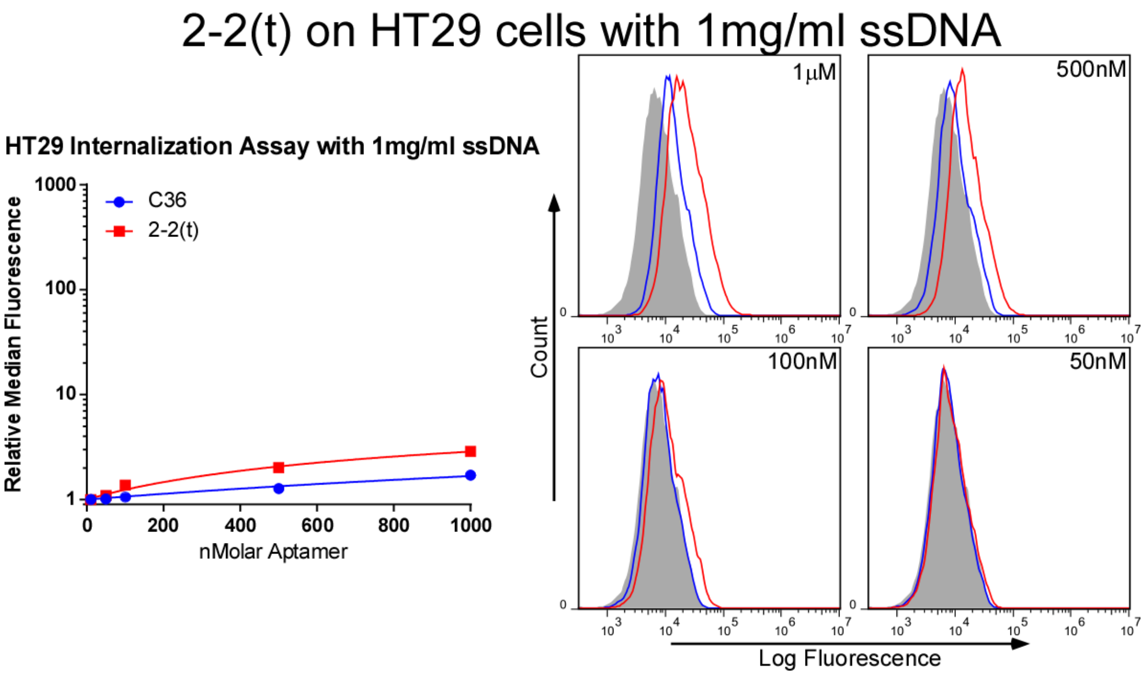

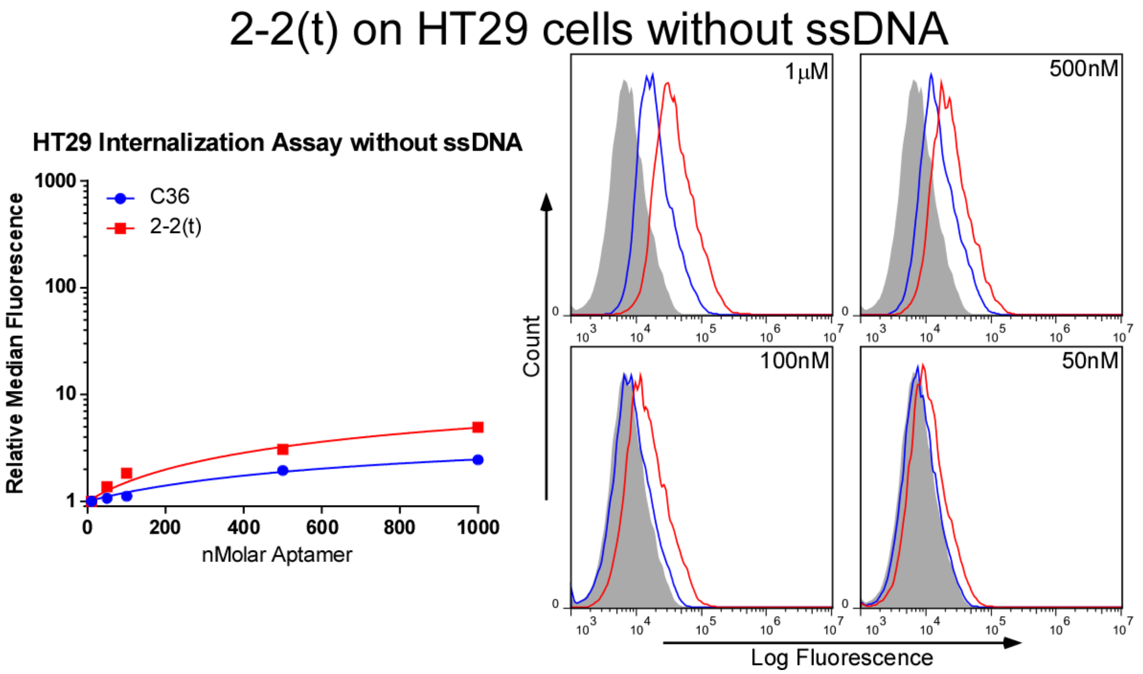

Supplementary Fig. 92. HER2 targeted aptamer 2-2(t) internalization and binding assays on HT29 cells. Graphs represent the median fluorescence of the aptamer (Red) and C36 (Blue) relative to unstained cells (Gray).

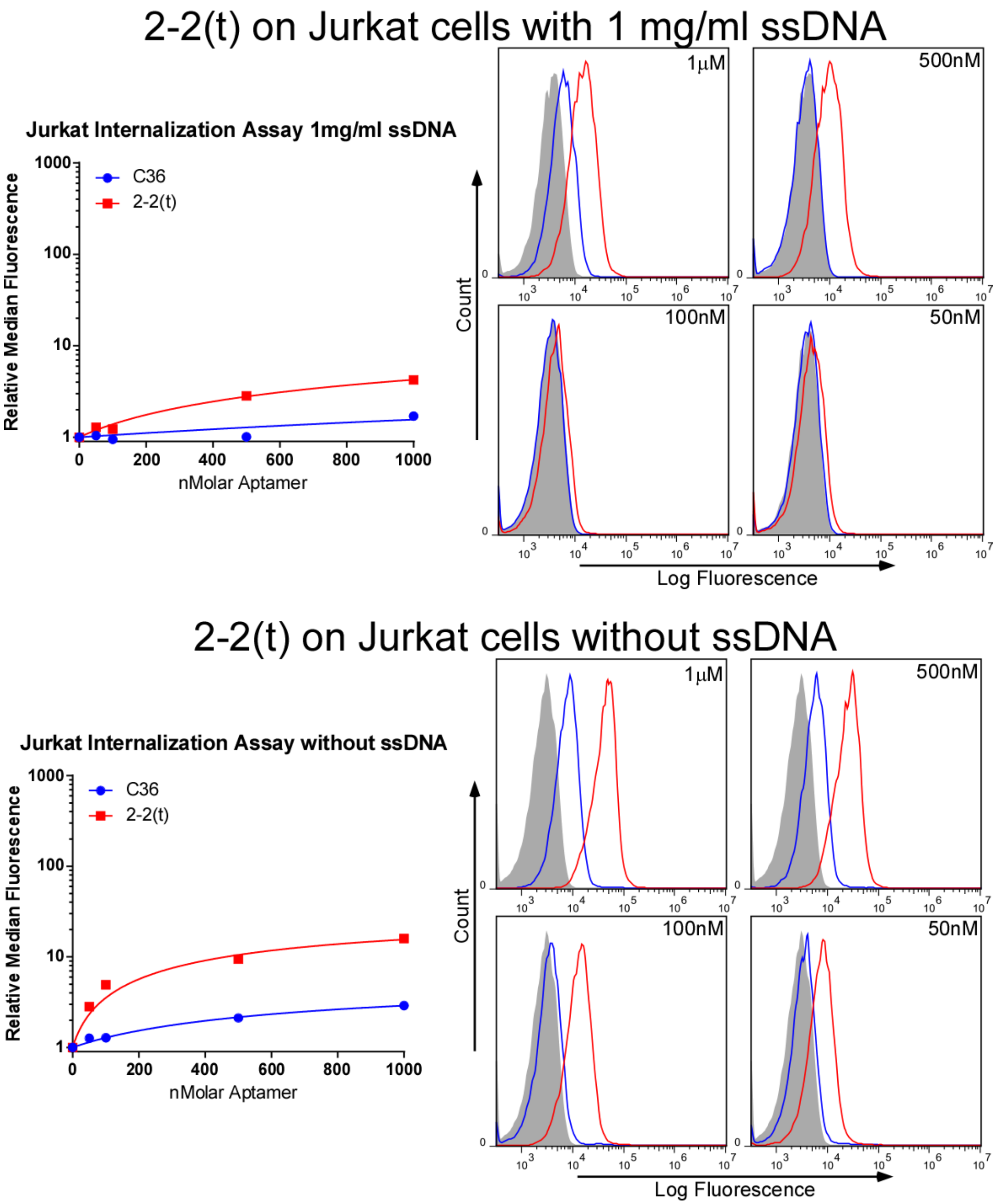

Supplementary Fig. 93. HER2 targeted aptamer 2-2(t) internalization and binding assays on Jurkat cells. Graphs represent the median fluorescence of the aptamer (Red) and C36 (Blue) relative to unstained cells (Gray).

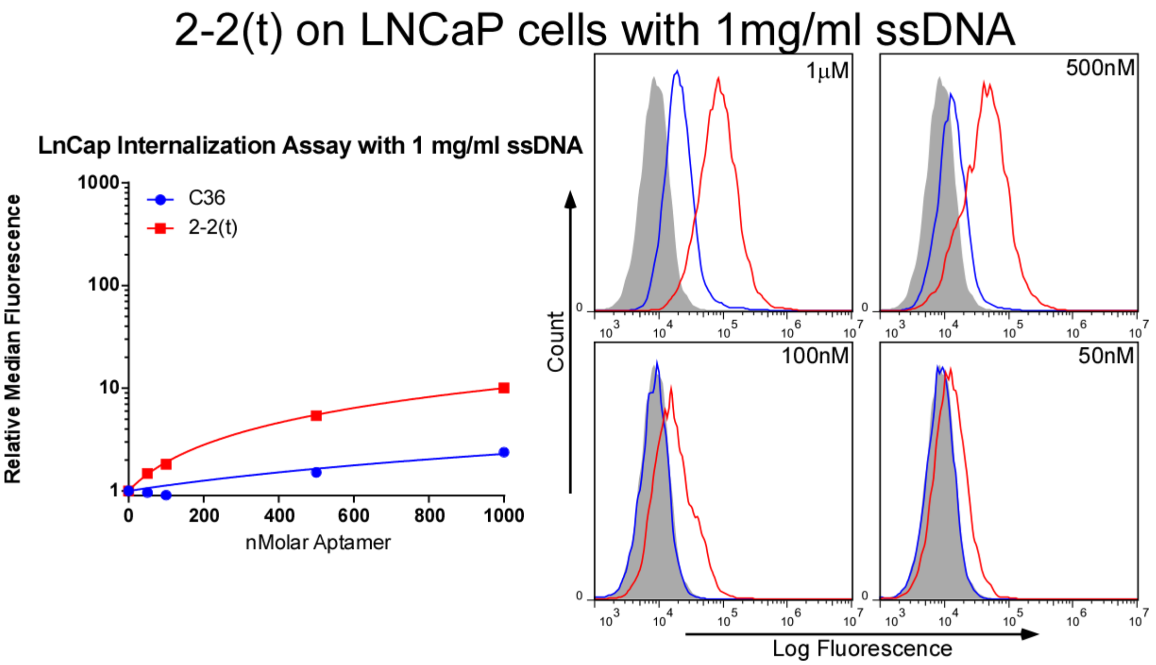

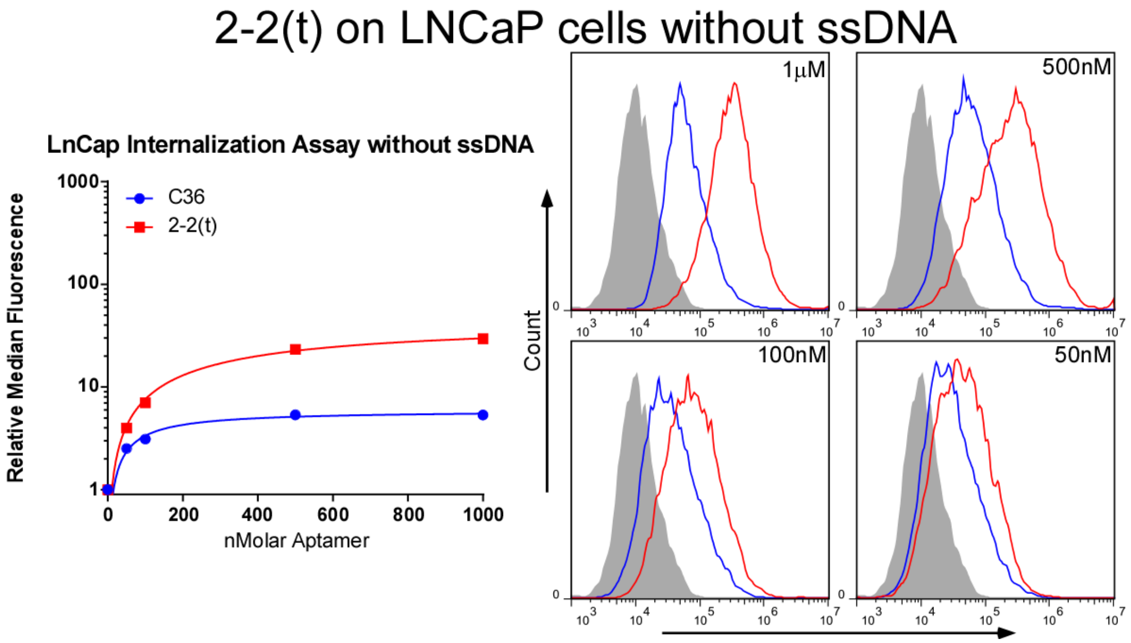

Supplementary Fig. 94. HER2 targeted aptamer 2-2(t) internalization and binding assays on LNCaP cells. Graphs represent the median fluorescence of the aptamer (Red) and C36 (Blue) relative to unstained cells (Gray).

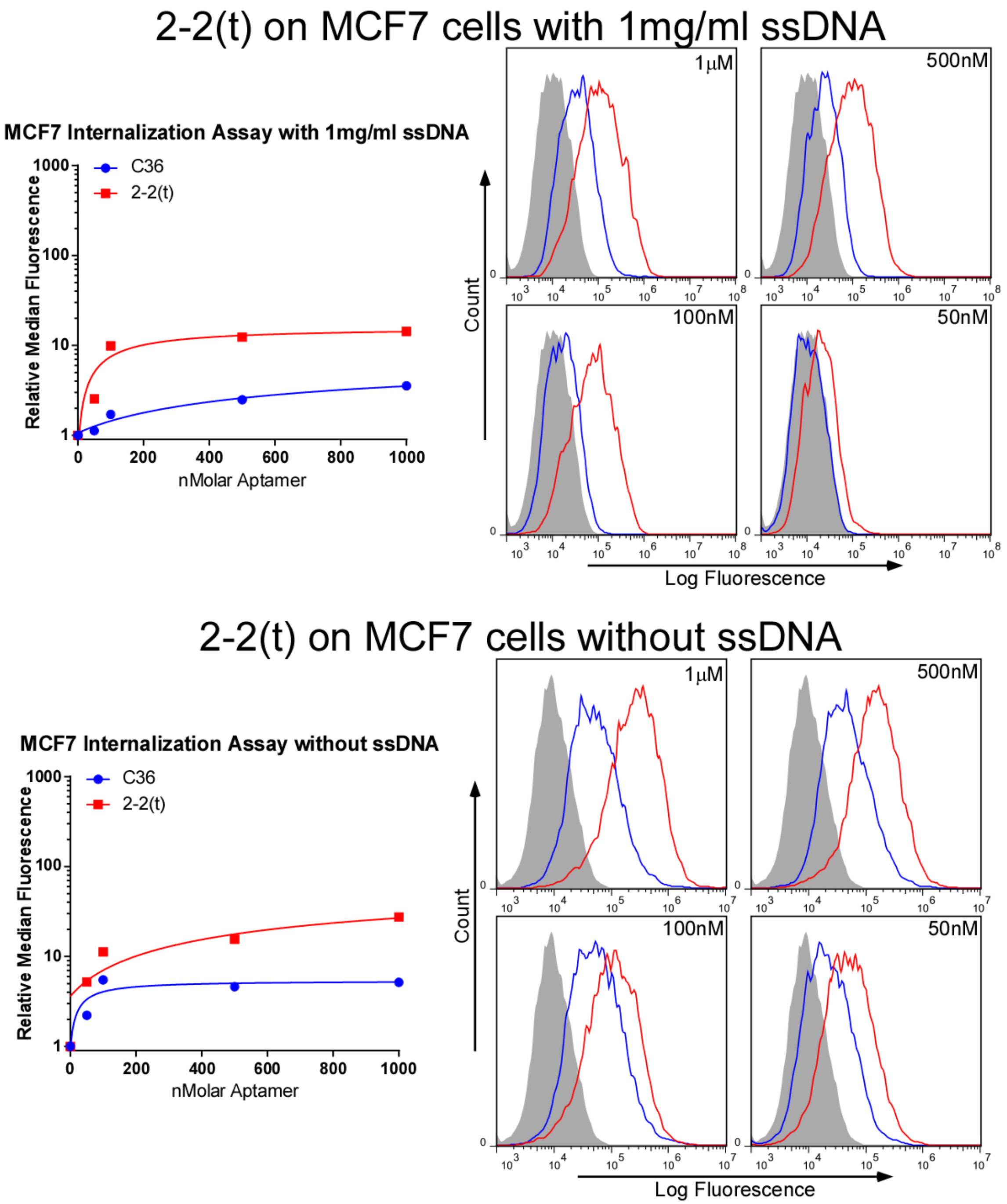

Supplementary Fig. 95. HER2 targeted aptamer 2-2(t) internalization and binding assays on MCF7 cells. Graphs represent the median fluorescence of the aptamer (Red) and C36 (Blue) relative to unstained cells (Gray).

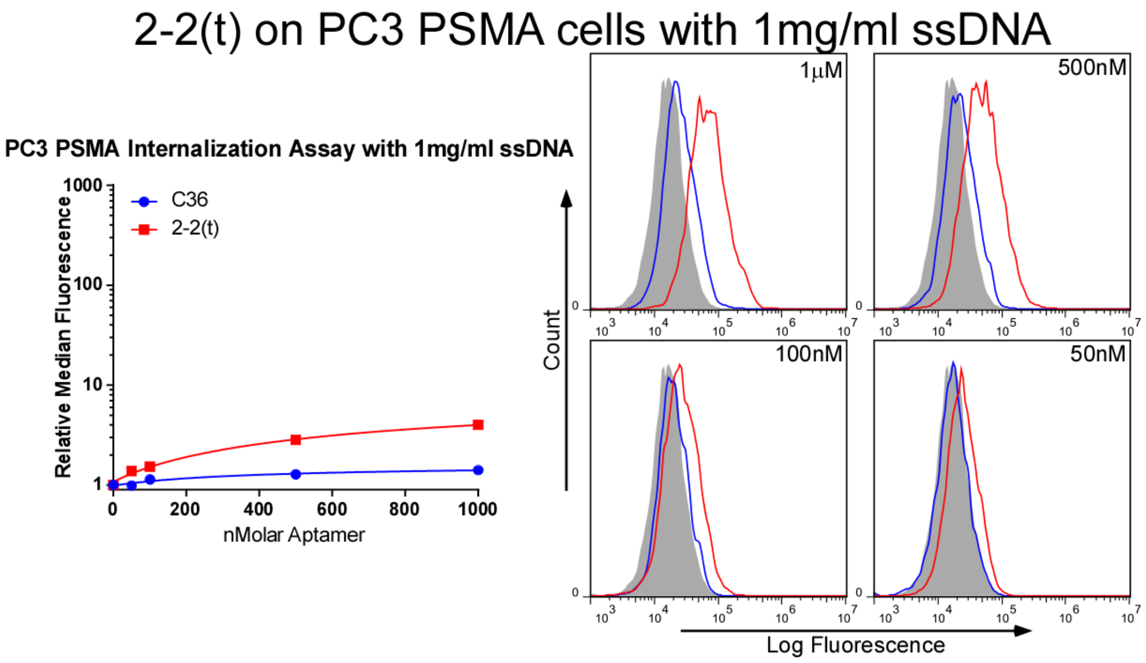

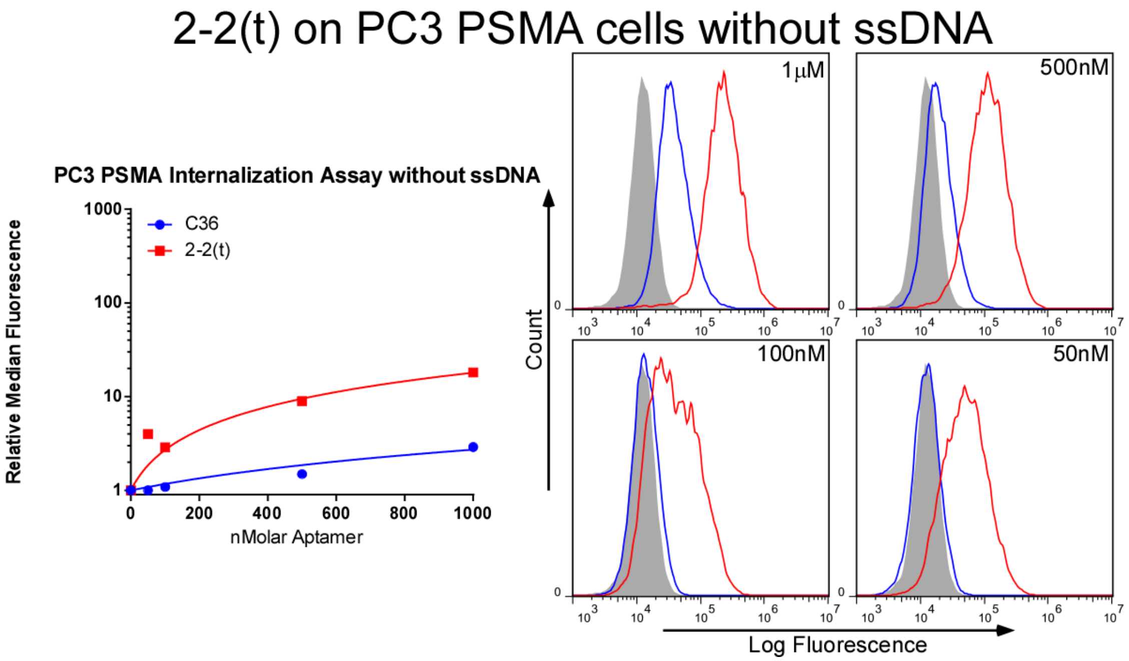

Supplementary Fig. 96. HER2 targeted aptamer 2-2(t) internalization and binding assays on PC3 PSMA cells. Graphs represent the median fluorescence of the aptamer (Red) and C36 (Blue) relative to unstained cells (Gray).

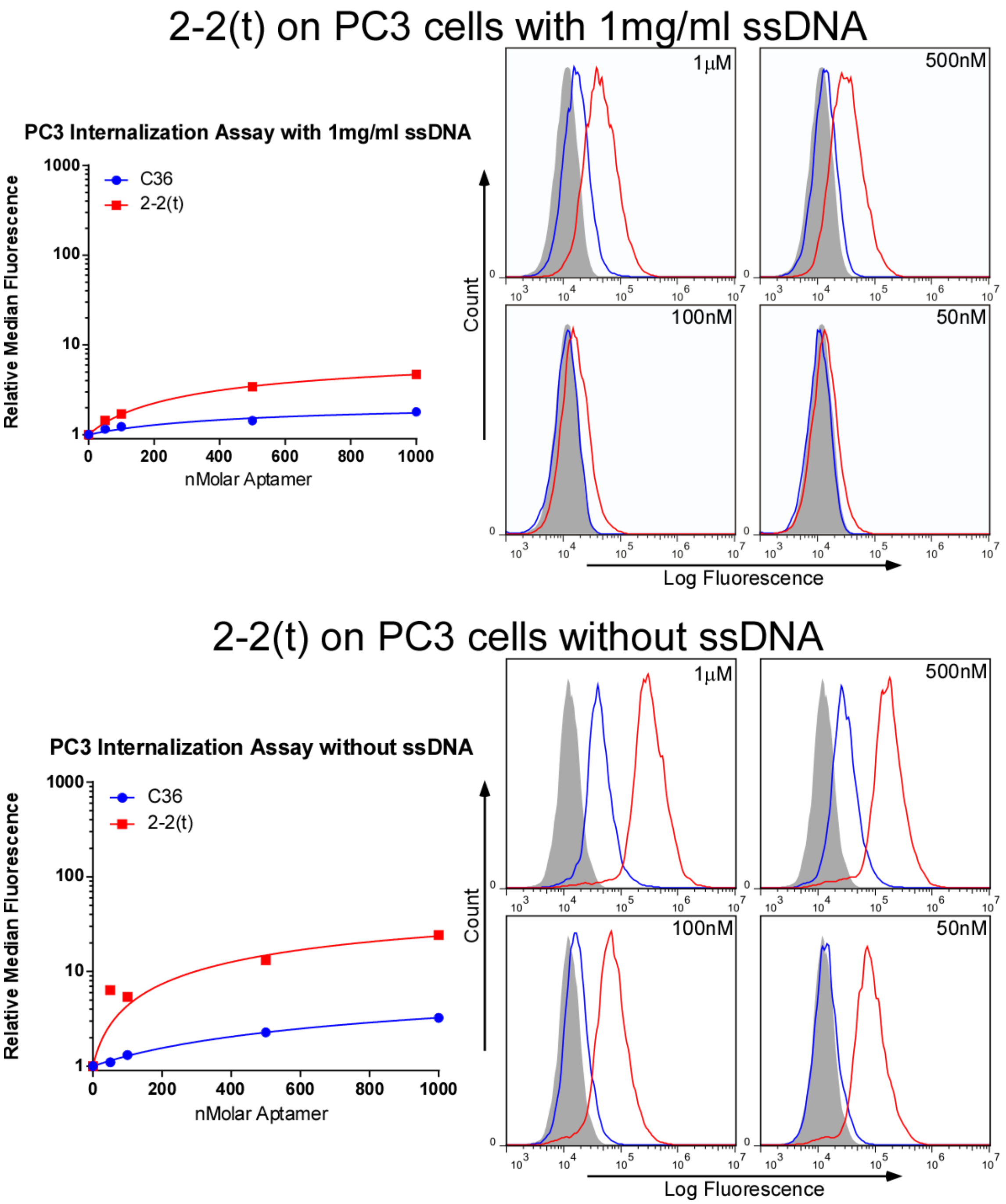

Supplementary Fig. 97. HER2 targeted aptamer 2-2(t) internalization and binding assays on PC3 cells. Graphs represent the median fluorescence of the aptamer (Red) and C36 (Blue) relative to unstained cells (Gray).

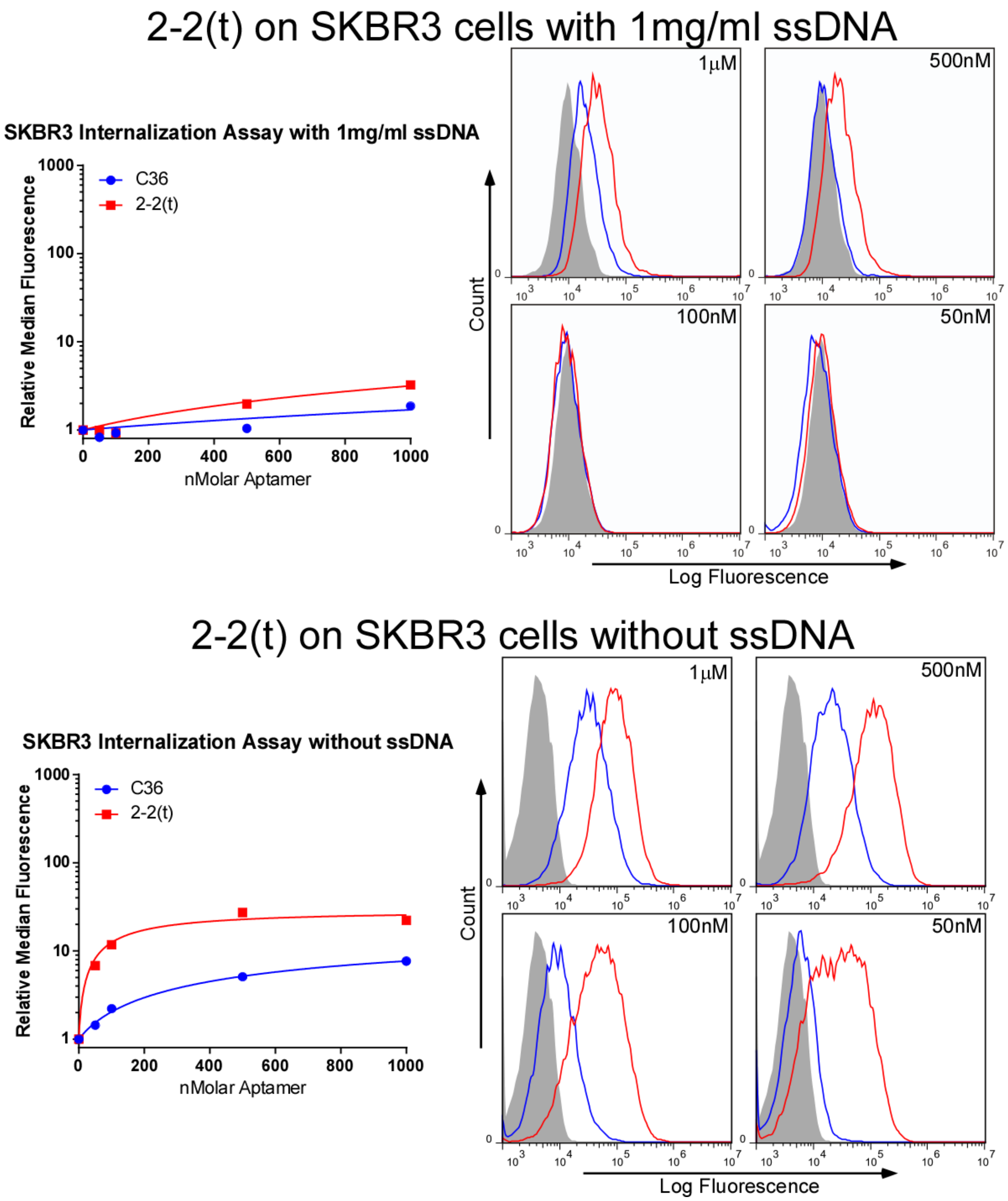

Supplementary Fig. 98. HER2 targeted aptamer 2-2(t) internalization and binding assays on SKBR3 cells. Graphs represent the median fluorescence of the aptamer (Red) and C36 (Blue) relative to unstained cells (Gray).

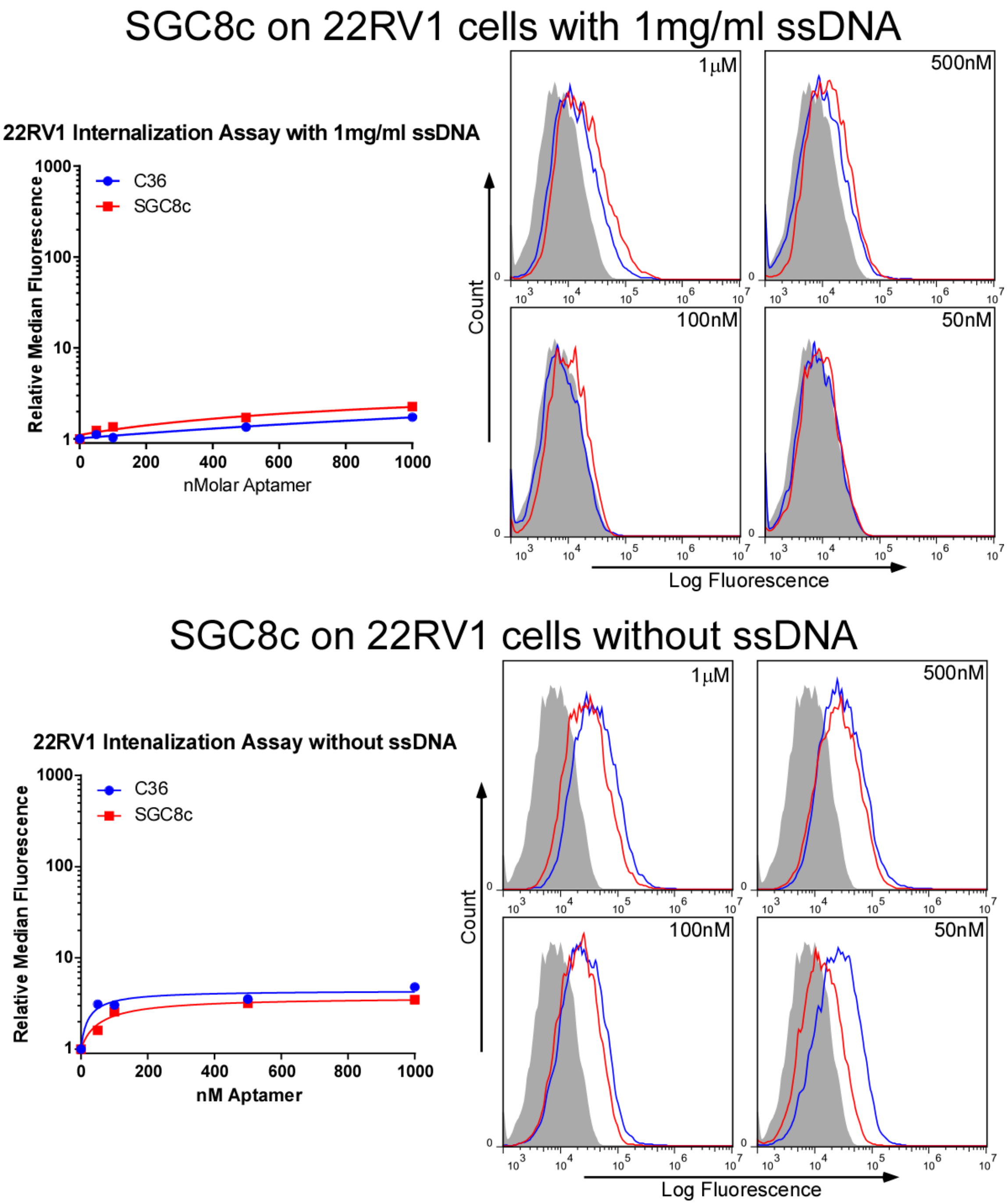

Supplementary Fig. 99. PTK7 targeted aptamer SGC8c internalization and binding assays on 22RV1 cells. Graphs represent the median fluorescence of the aptamer (Red) and C36 (Blue) relative to unstained cells (Gray).

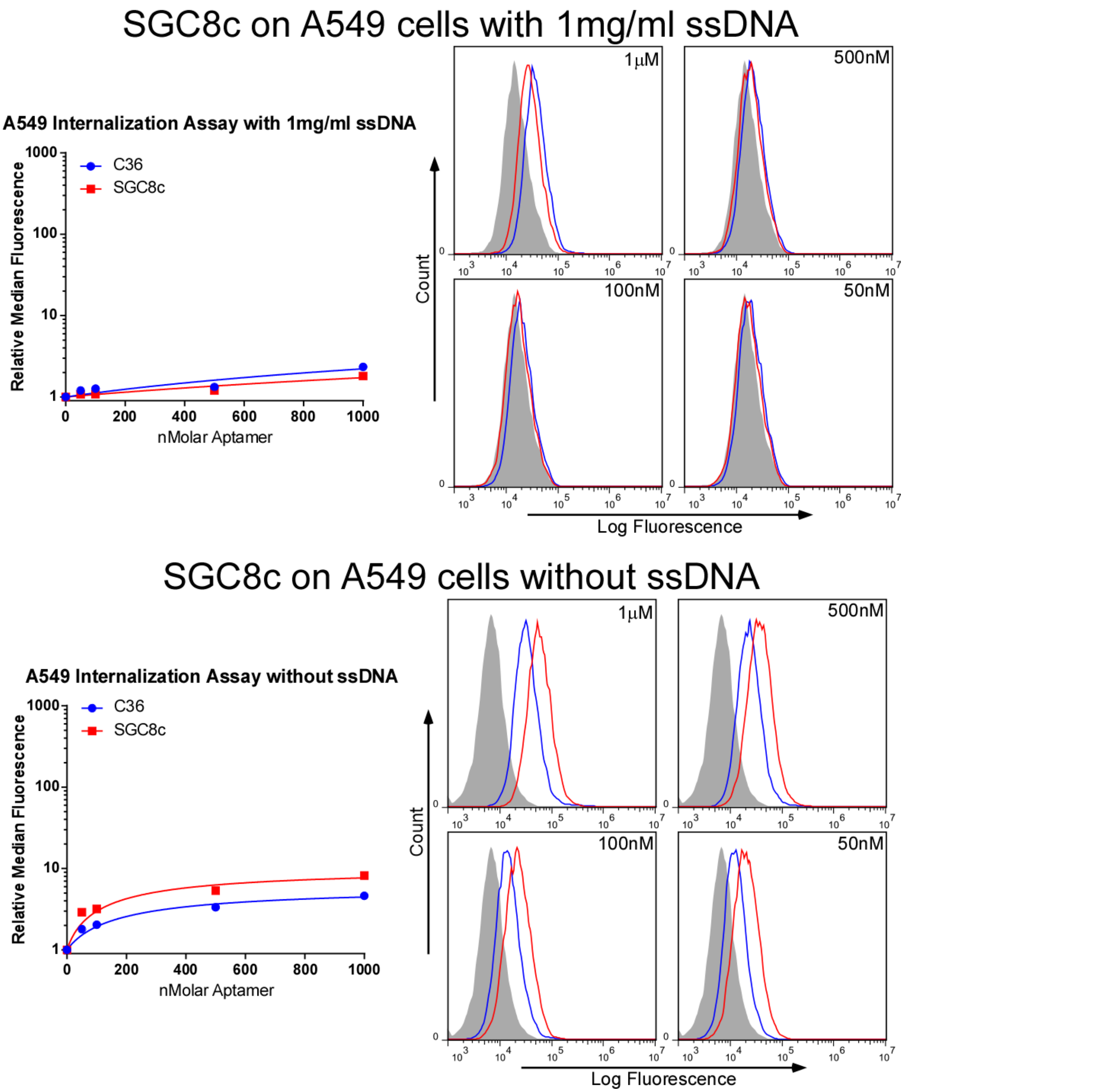

Supplementary Fig. 100. PTK7 binding aptamer SGC8c internalization and internalization assays on A549 cells. Graphs represent the median fluorescence of the aptamer (Red) and C36 (Blue) relative to unstained cells (Gray).

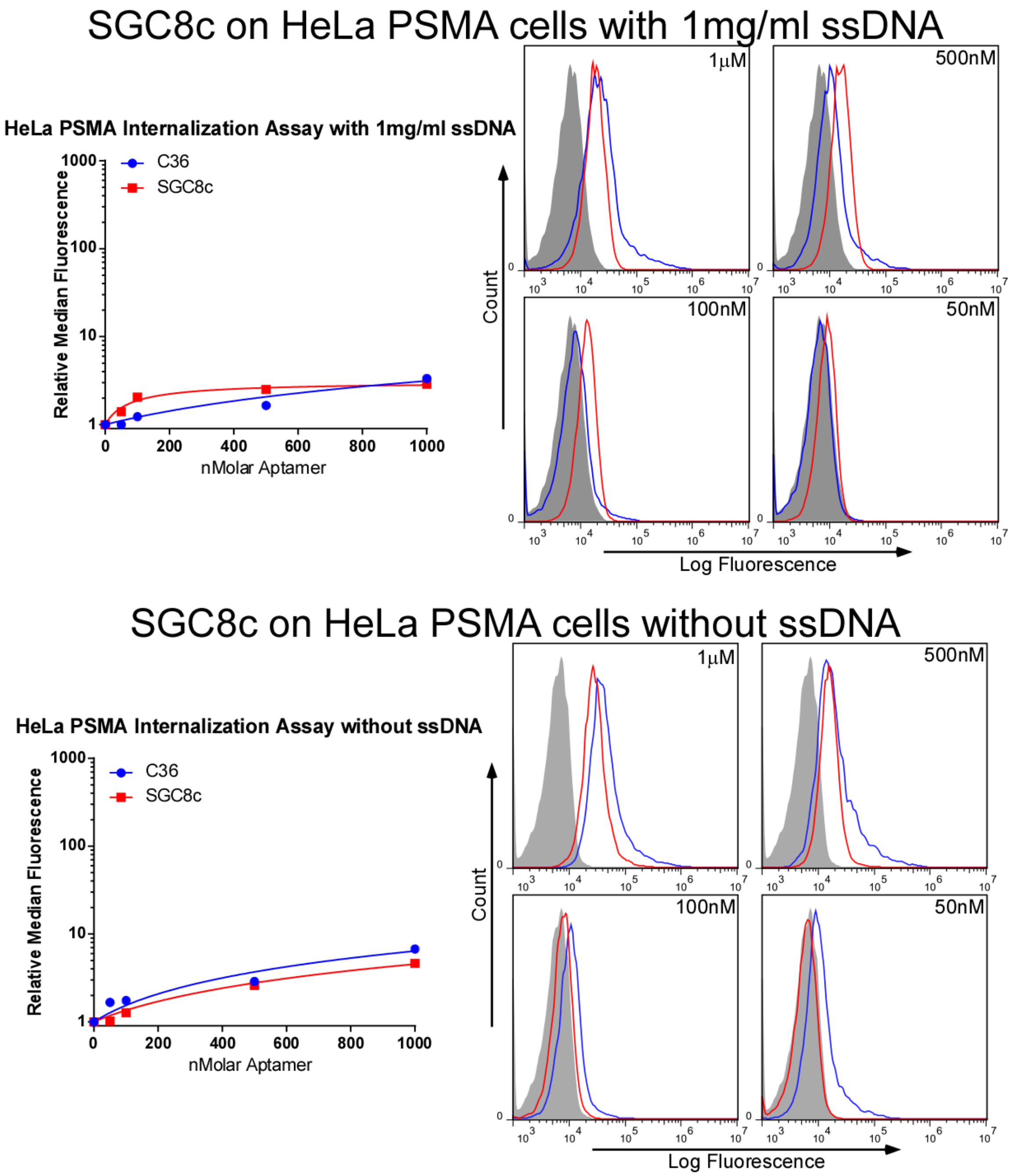

Supplementary Fig. 101. PTK7 binding aptamer SGC8c internalization and binding assays on HeLa PSMA cells. Graphs represent the median fluorescence of the aptamer (Red) and C36 (Blue) relative to unstained cells (Gray).

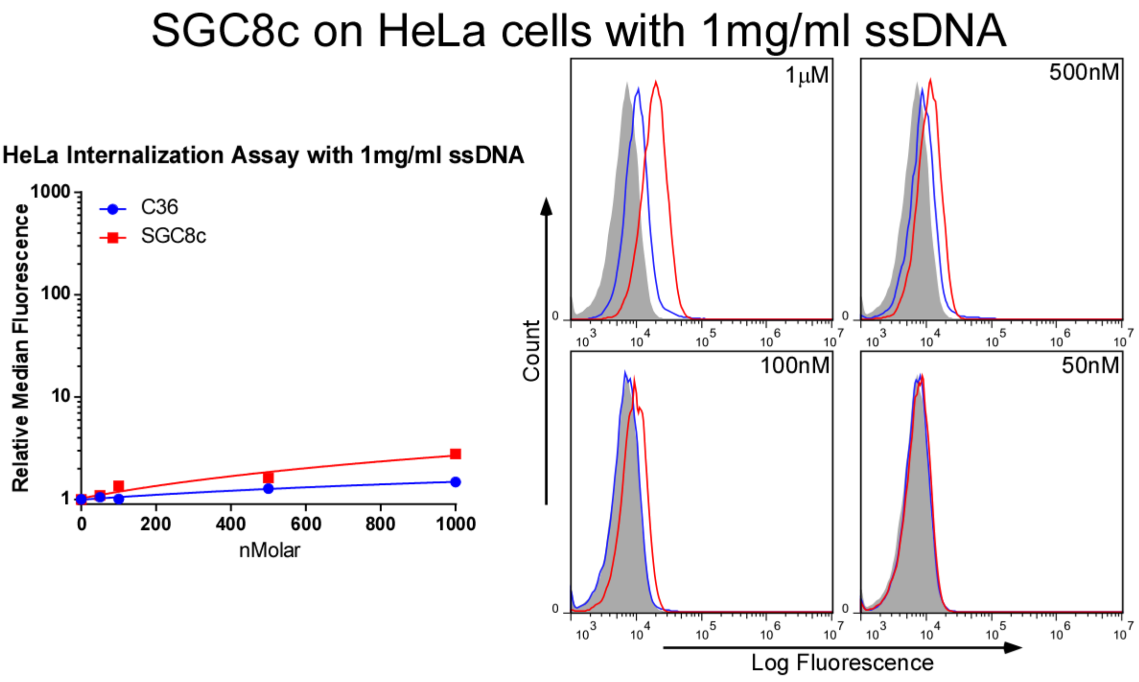

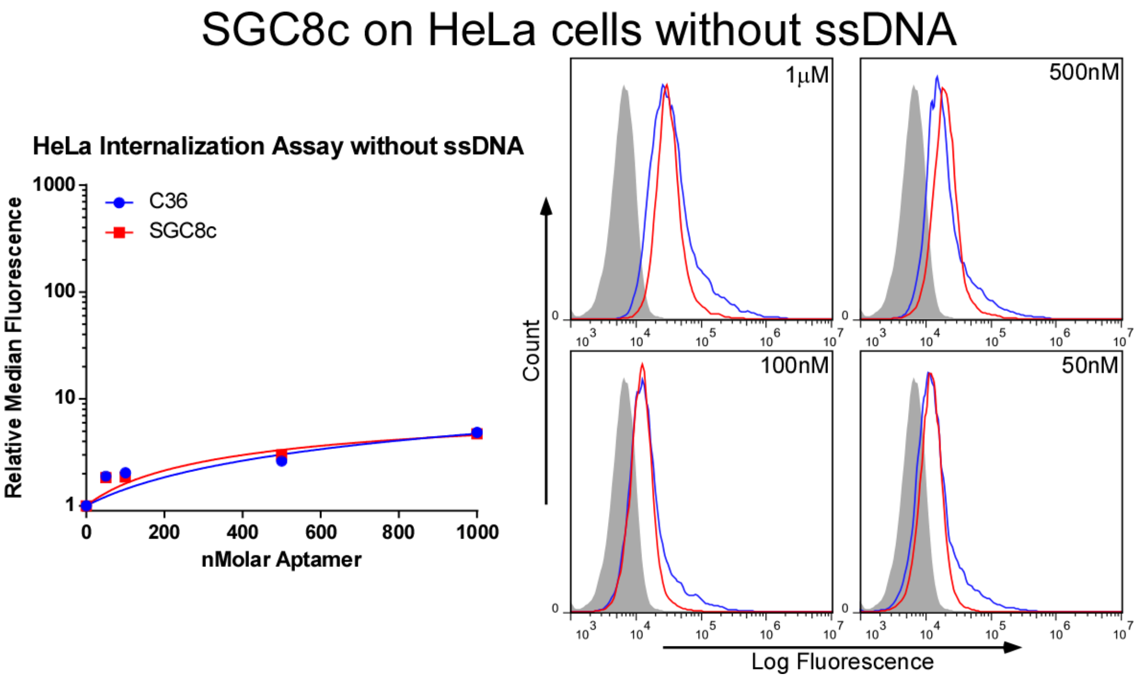

Supplementary Fig. 102. PTK7 binding aptamer SGC8c internalization and binding assays on HeLa cells. Graphs represent the median fluorescence of the aptamer (Red) and C36 (Blue) relative to unstained cells (Gray).

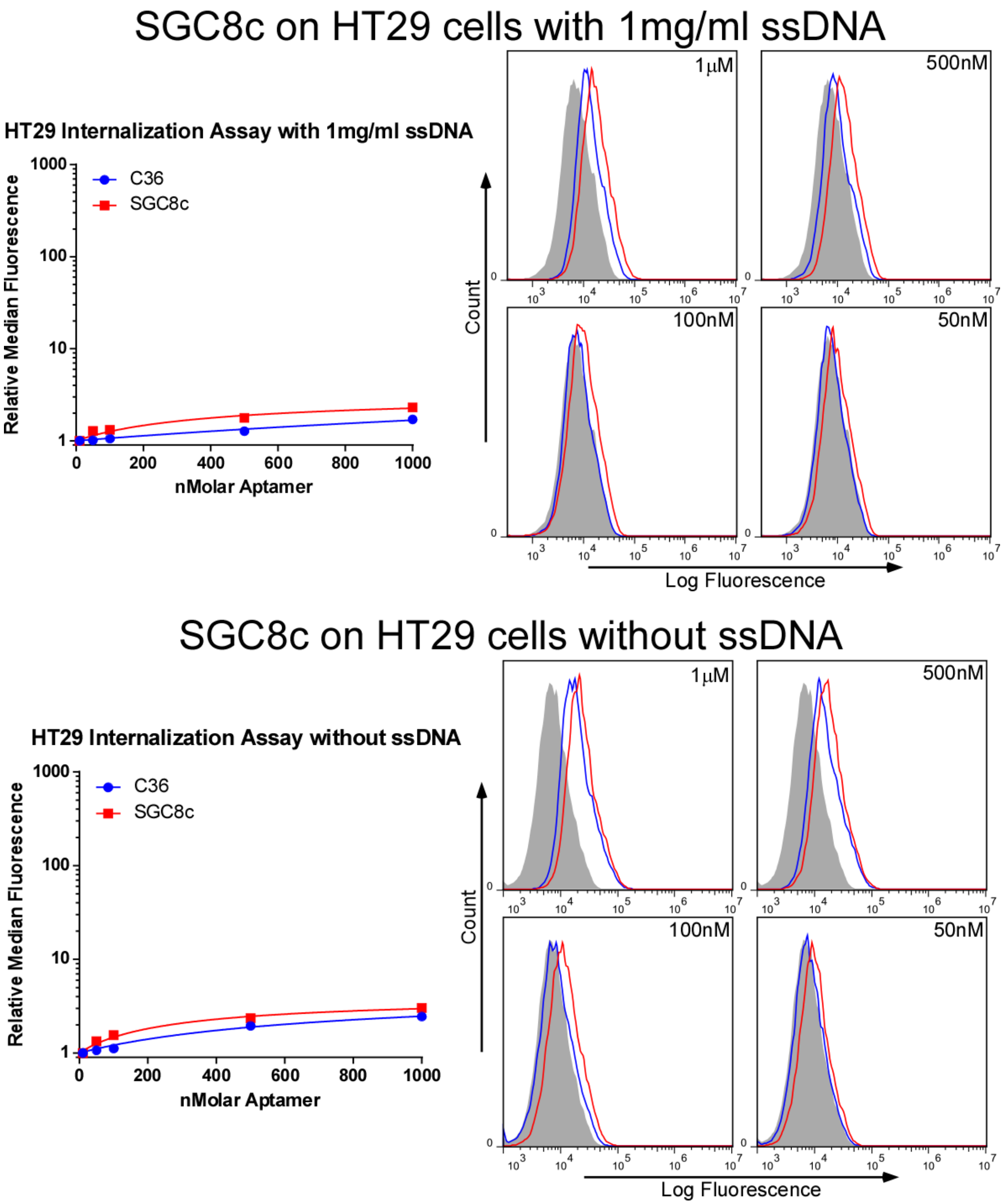

Supplementary Fig. 103. PTK7 binding aptamer SGC8c internalization and binding assays on HT29 cells. Graphs represent the median fluorescence of the aptamer (Red) and C36 (Blue) relative to unstained cells (Gray).

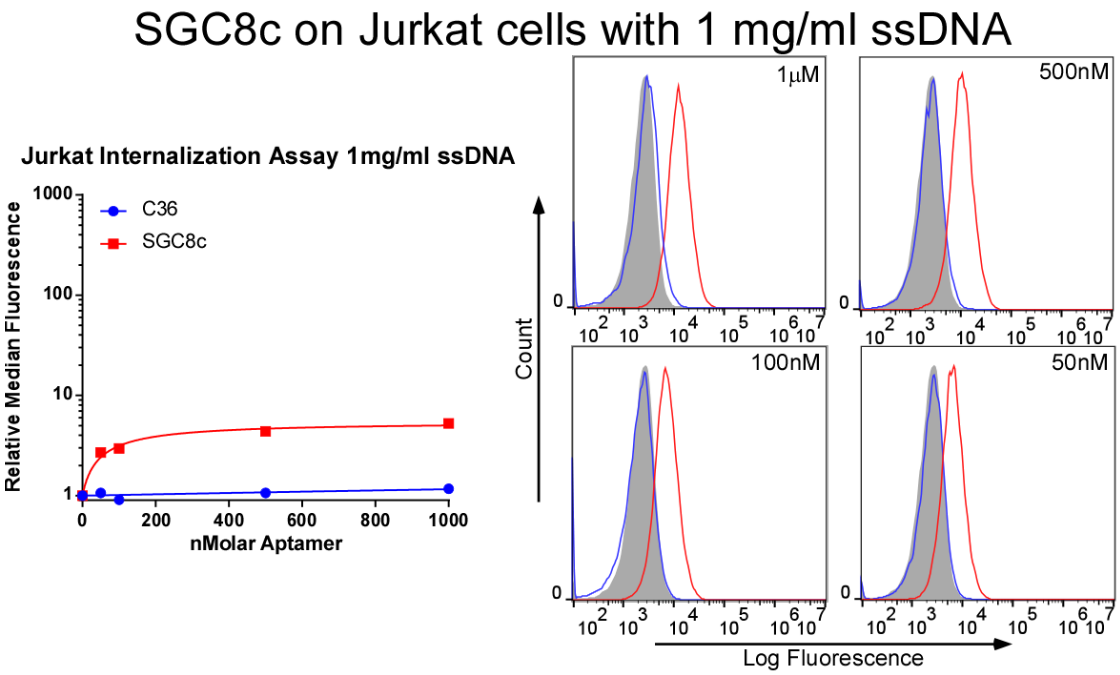

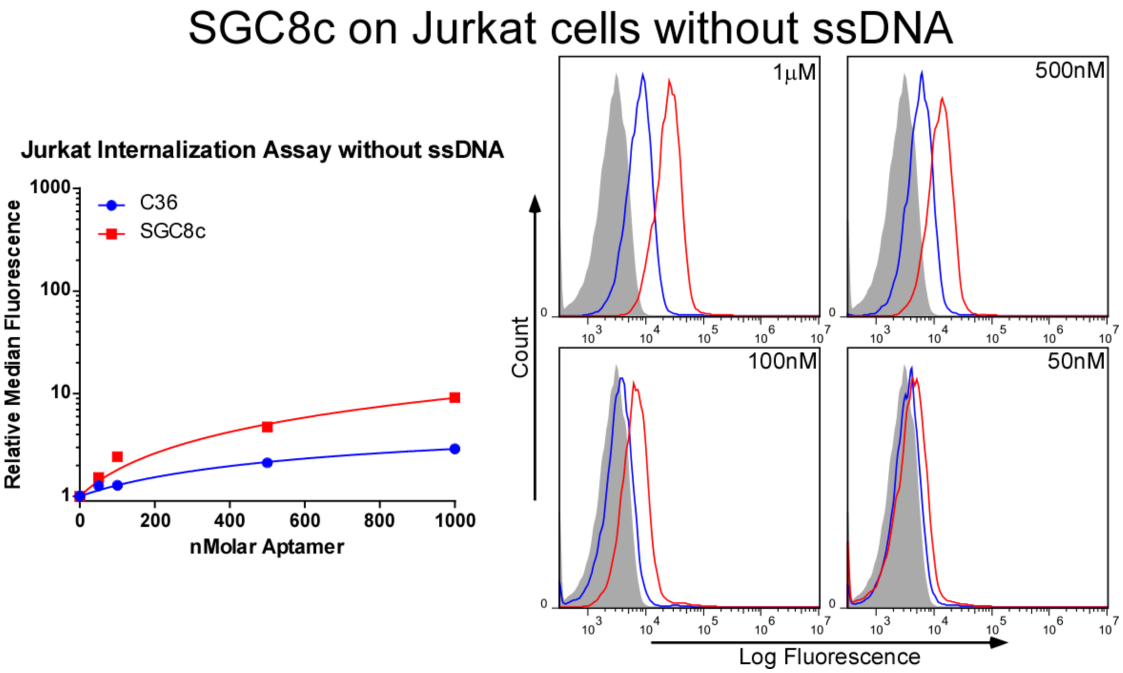

Supplementary Fig. 104. PTK7 binding aptamer SGC8c internalization and binding assays on Jurkat cells. Graphs represent the median fluorescence of the aptamer (Red) and C36 (Blue) relative to unstained cells (Gray).

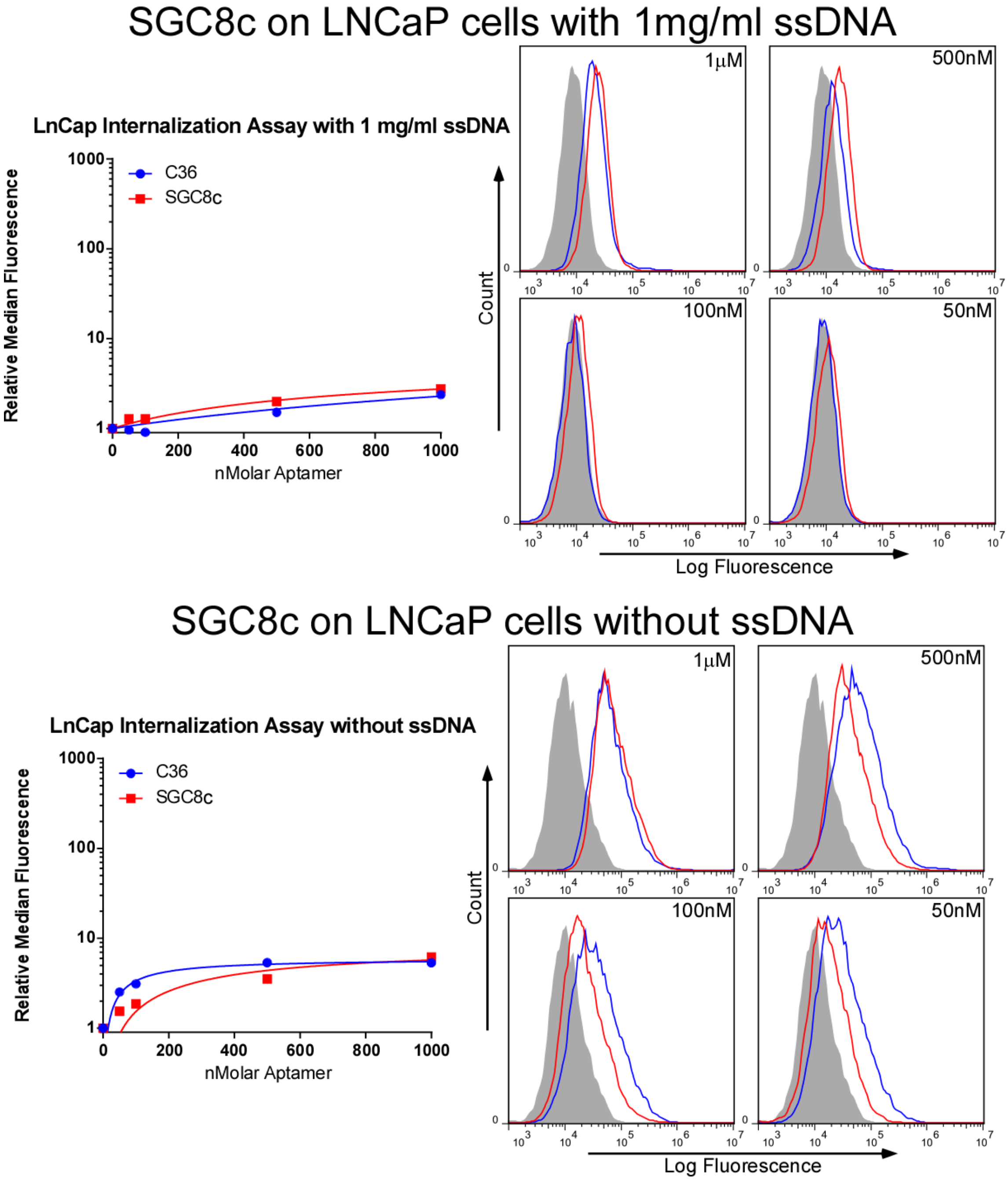

Supplementary Fig. 105. PTK7 binding aptamer SGC8c internalization and binding assays on LNCaP cells. Graphs represent the median fluorescence of the aptamer (Red) and C36 (Blue) relative to unstained cells (Gray).

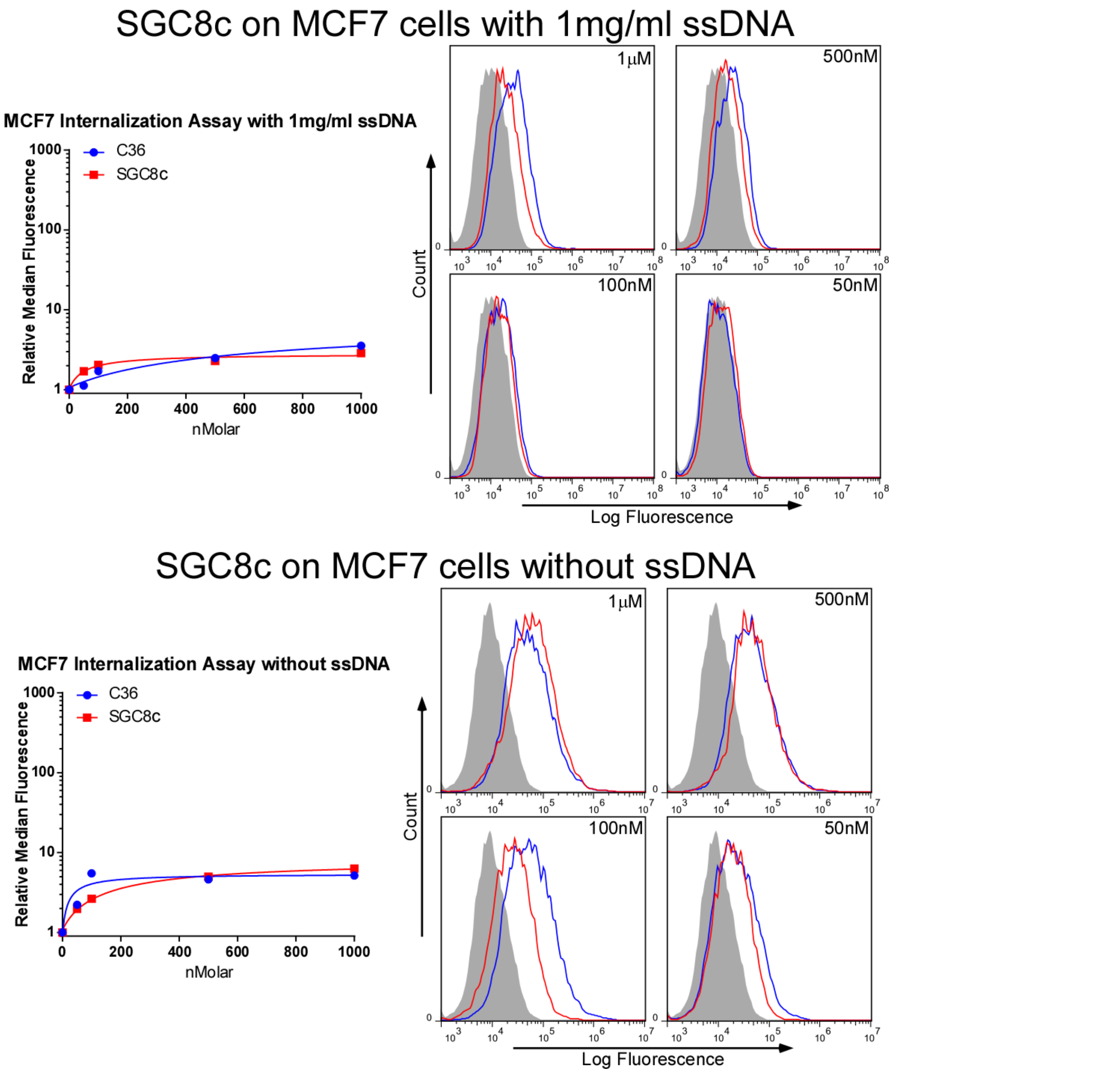

Supplementary Fig. 106. PTK7 binding aptamer SGC8c internalization and binding assays on MCF7 cells. Graphs represent the median fluorescence of the aptamer (Red) and C36 (Blue) relative to unstained cells (Gray).

SGC8c on PC3 PSMA cells with 1mg/ml ssDNA

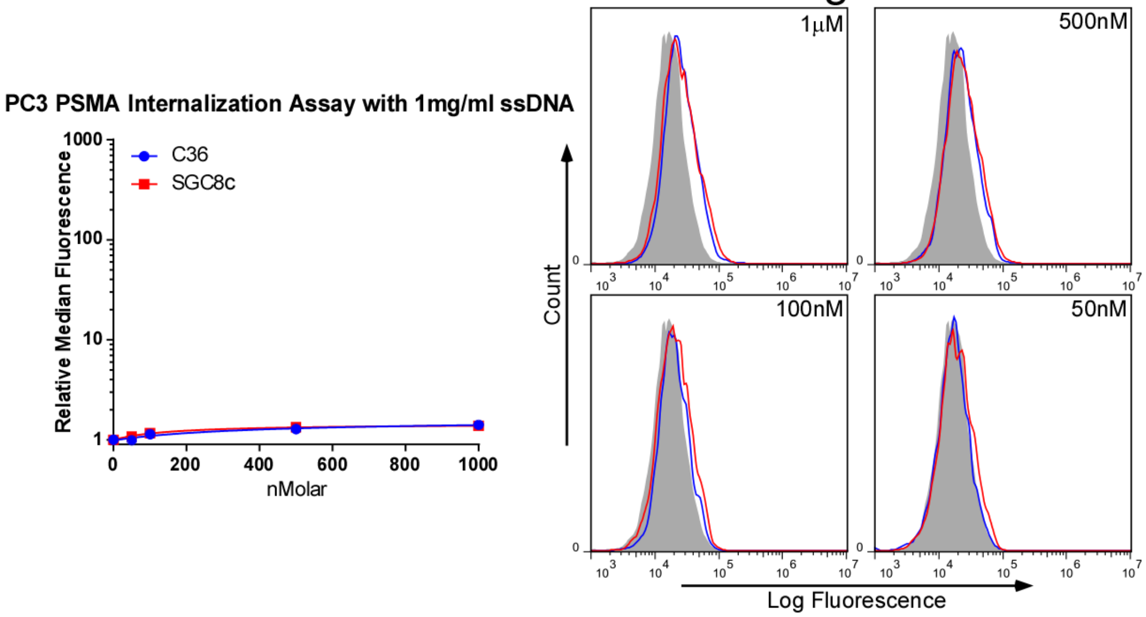

SGC8c on PC3 PSMA cells without ssDNA

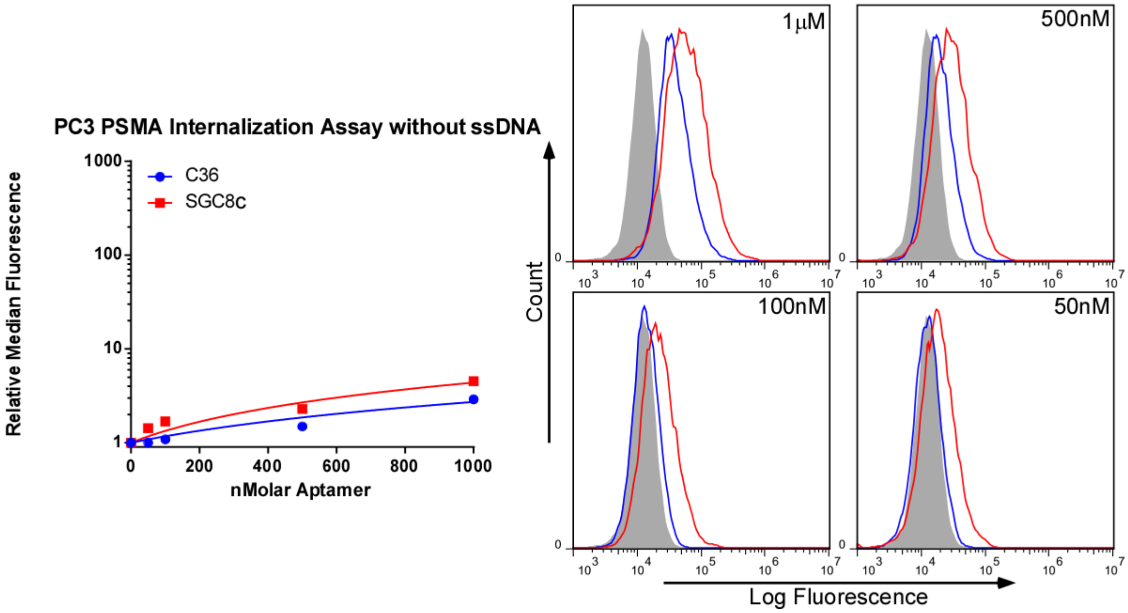

Supplementary Fig. 107. PTK7 binding aptamer SGC8c internalization and binding assays on PC3 PSMA cells. Graphs represent the median fluorescence of the aptamer (Red) and C36 (Blue) relative to unstained cells (Gray).

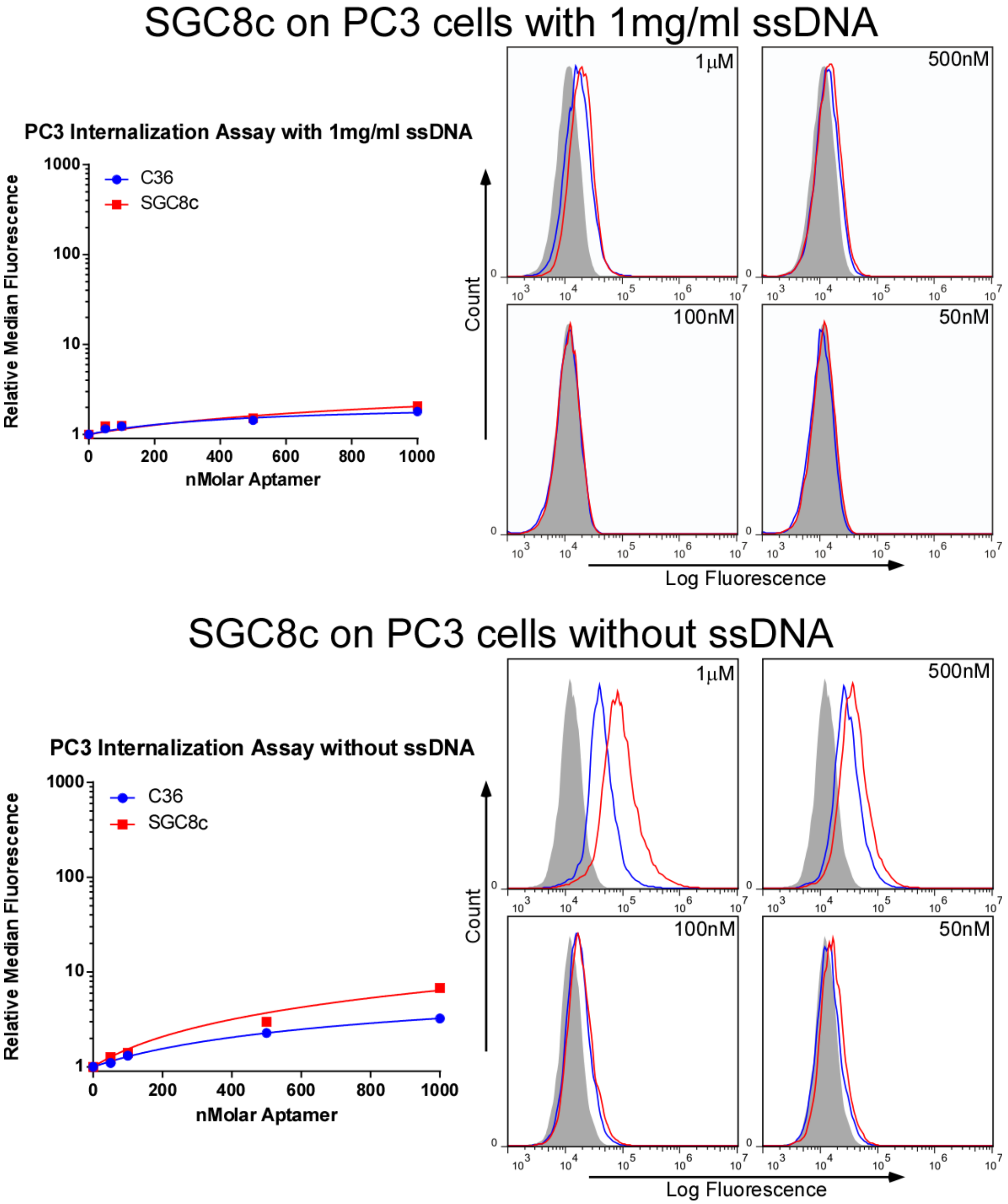

Supplementary Fig. 108. PTK7 binding aptamer SGC8c internalization and binding assays on PC3 cells. Graphs represent the median fluorescence of the aptamer (Red) and C36 (Blue) relative to unstained cells (Gray).

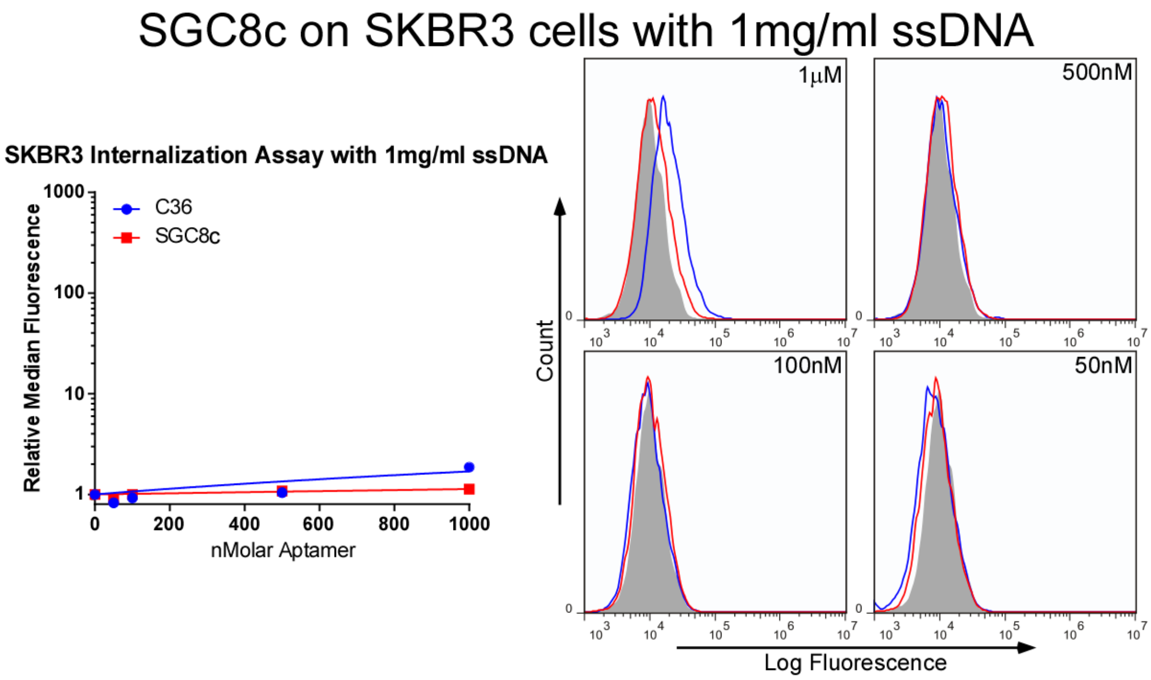

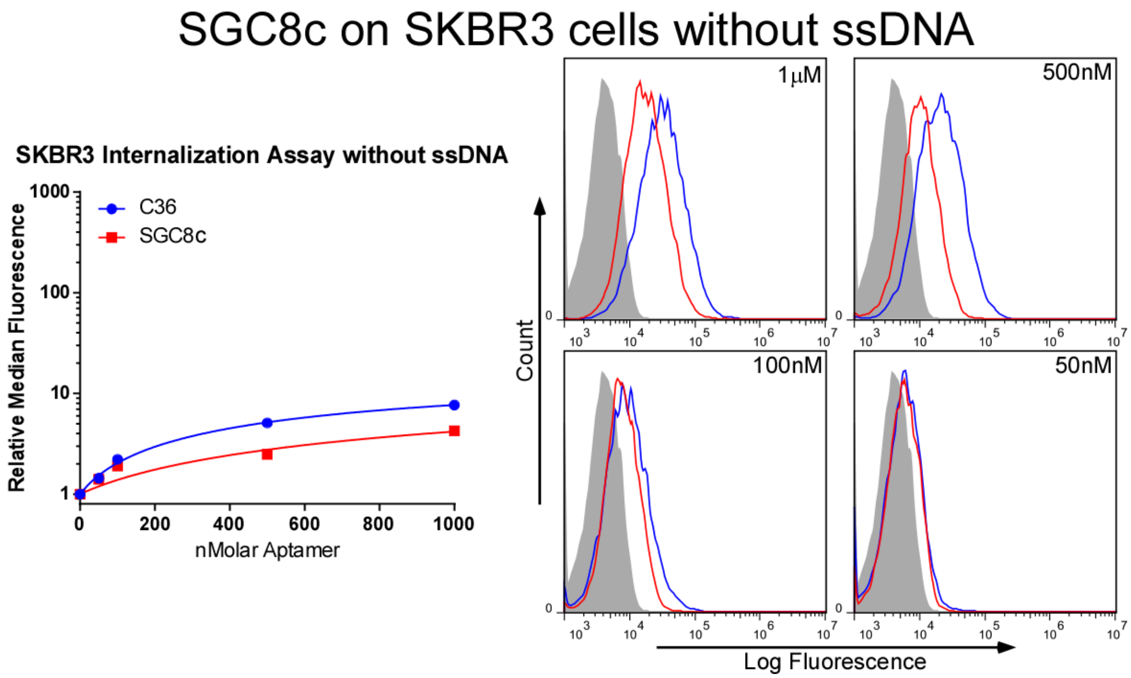

Supplementary Fig. 109. PTK7 binding aptamer SGC8c internalization and binding assays on SKBR3 cells. Graphs represent the median fluorescence of the aptamer (Red) and C36 (Blue) relative to unstained cells (Gray).

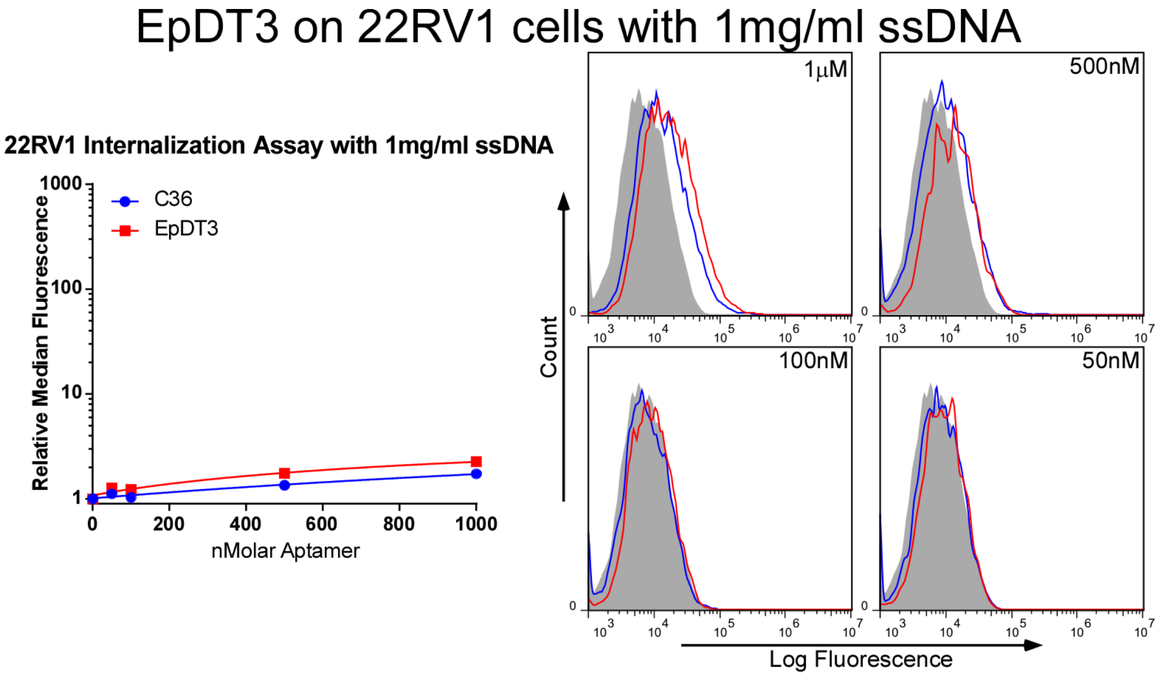

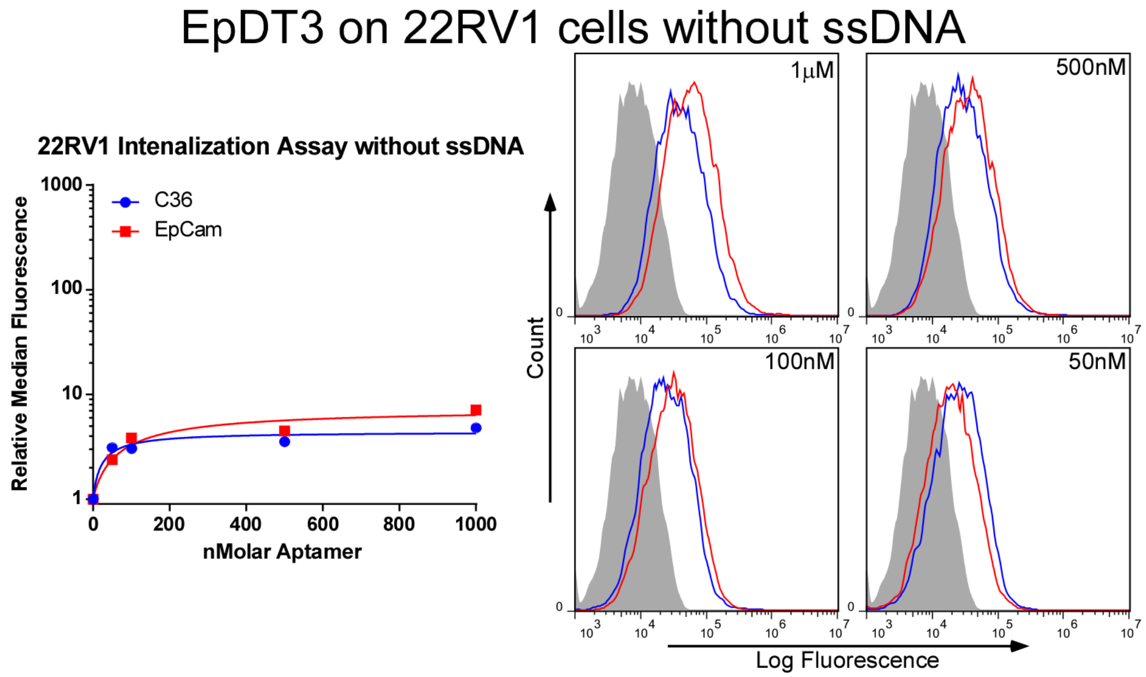

Supplementary Fig. 110. EpCAM targeted aptamer EpDT3 internalization and binding assays on 22RV1 cells. Graphs represent the median fluorescence of the aptamer (Red) and C36 (Blue) relative to unstained cells (Gray).

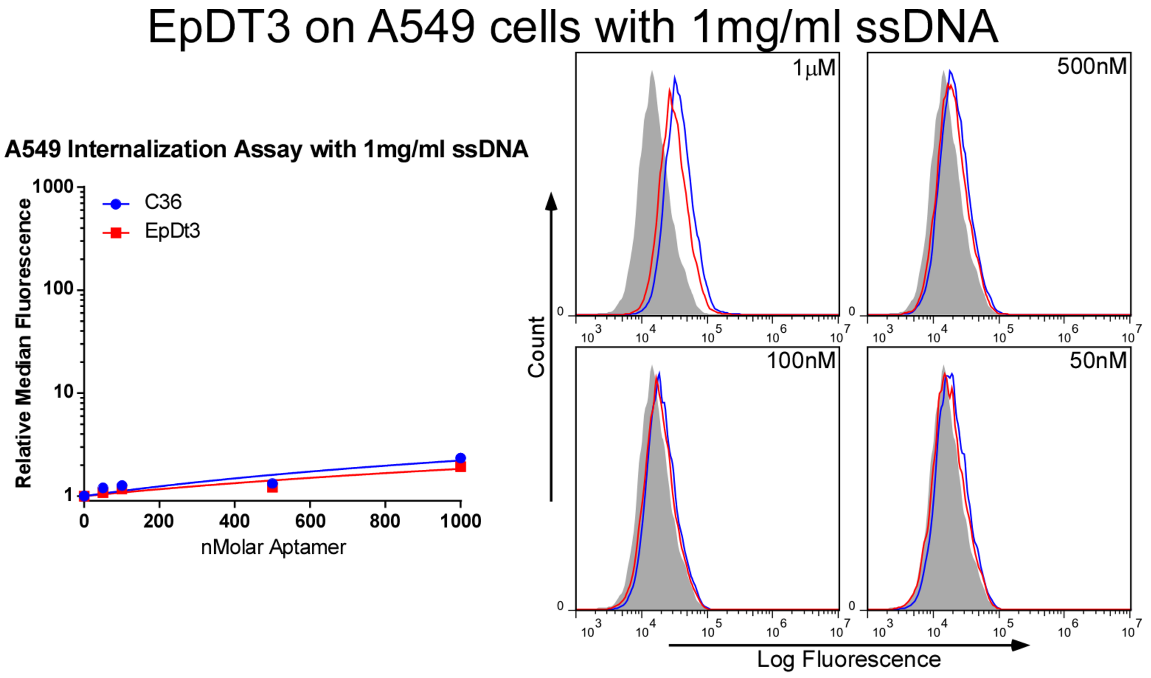

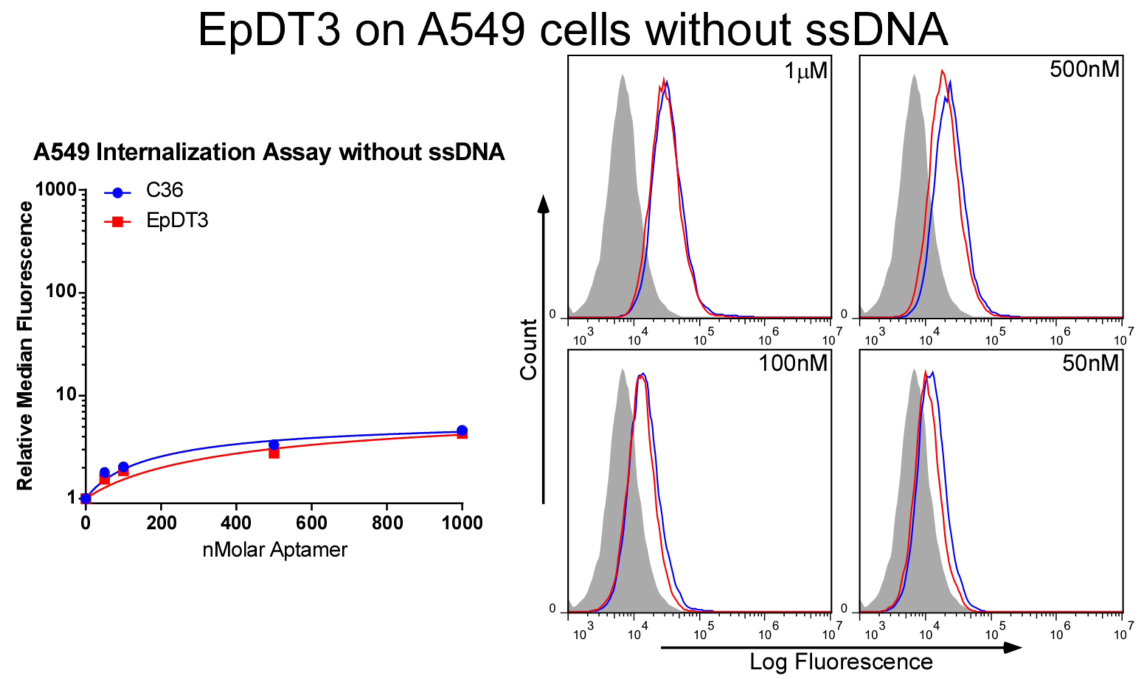

Supplementary Fig. 111. EpCAM targeted aptamer EpDT3 internalization and binding assays on A549 cells. Graphs represent the median fluorescence of the aptamer (Red) and C36 (Blue) relative to unstained cells (Gray).

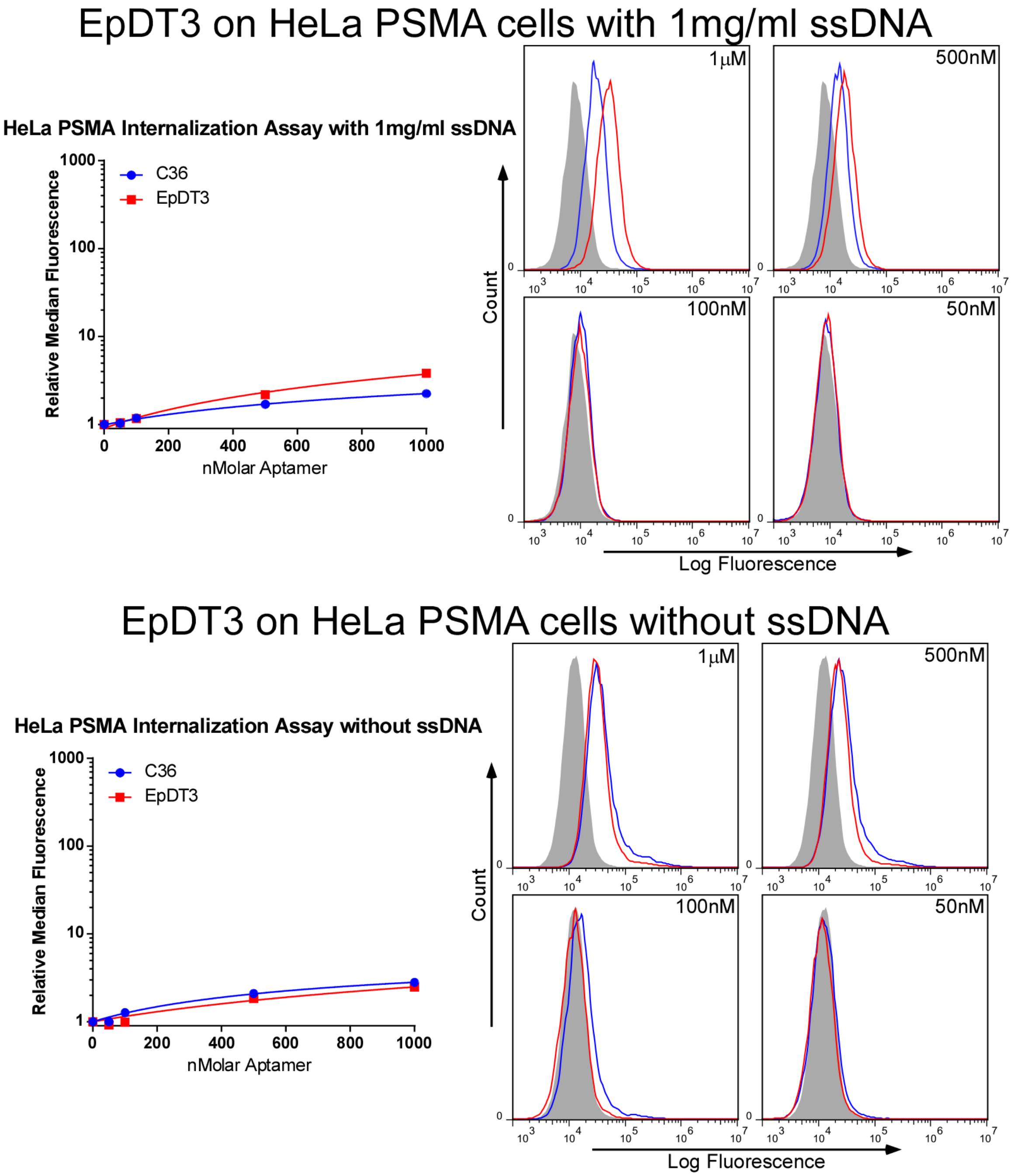

Supplementary Fig. 112. EpCAM targeted aptamer EpDT3 internalization and binding assays on HeLa PSMA cells. Graphs represent the median fluorescence of the aptamer (Red) and C36 (Blue) relative to unstained cells (Gray).

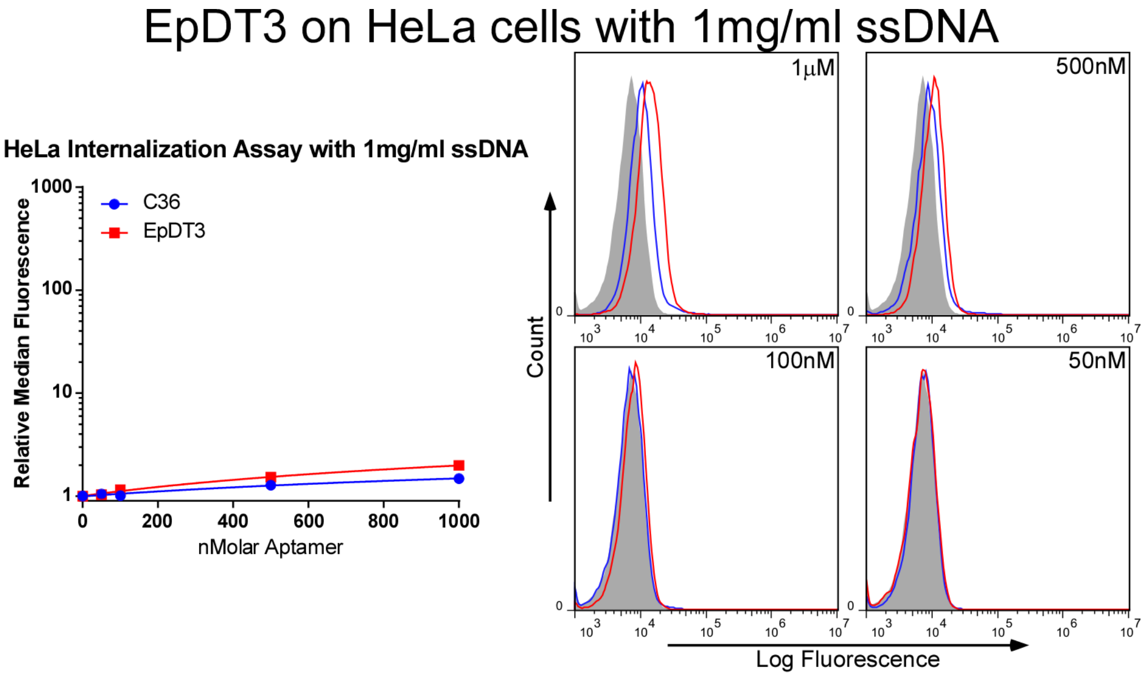

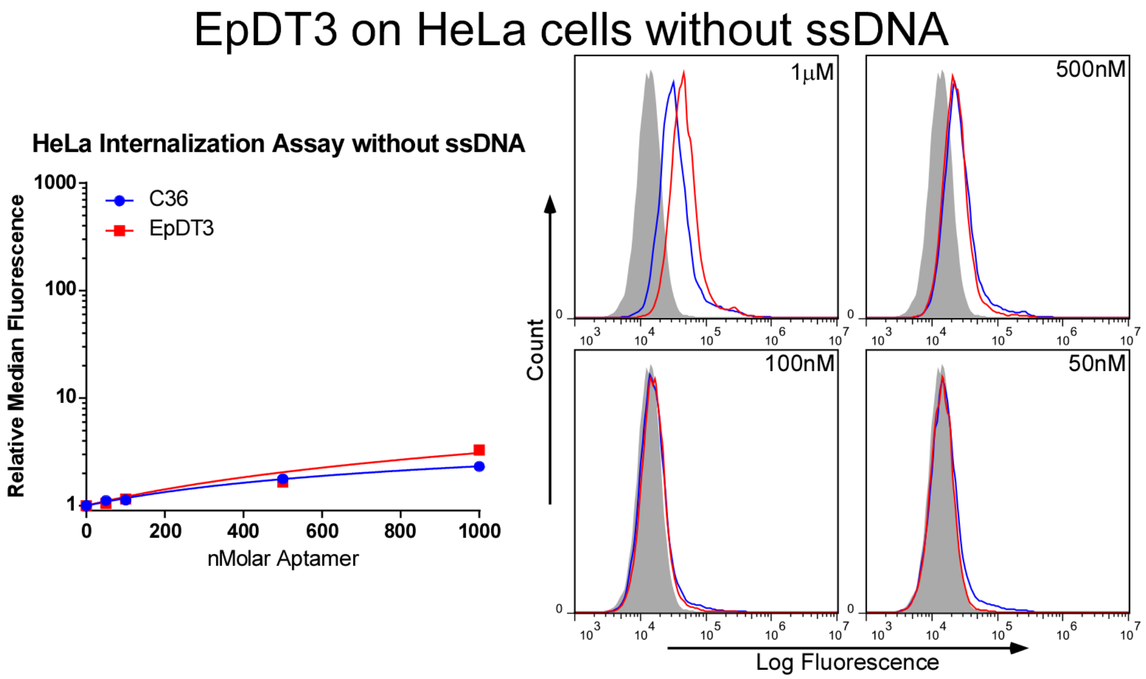

Supplementary Fig. 113. EpCAM targeted aptamer EpDT3 internalization and binding assays on HeLa cells. Graphs represent the median fluorescence of the aptamer (Red) and C36 (Blue) relative to unstained cells (Gray).

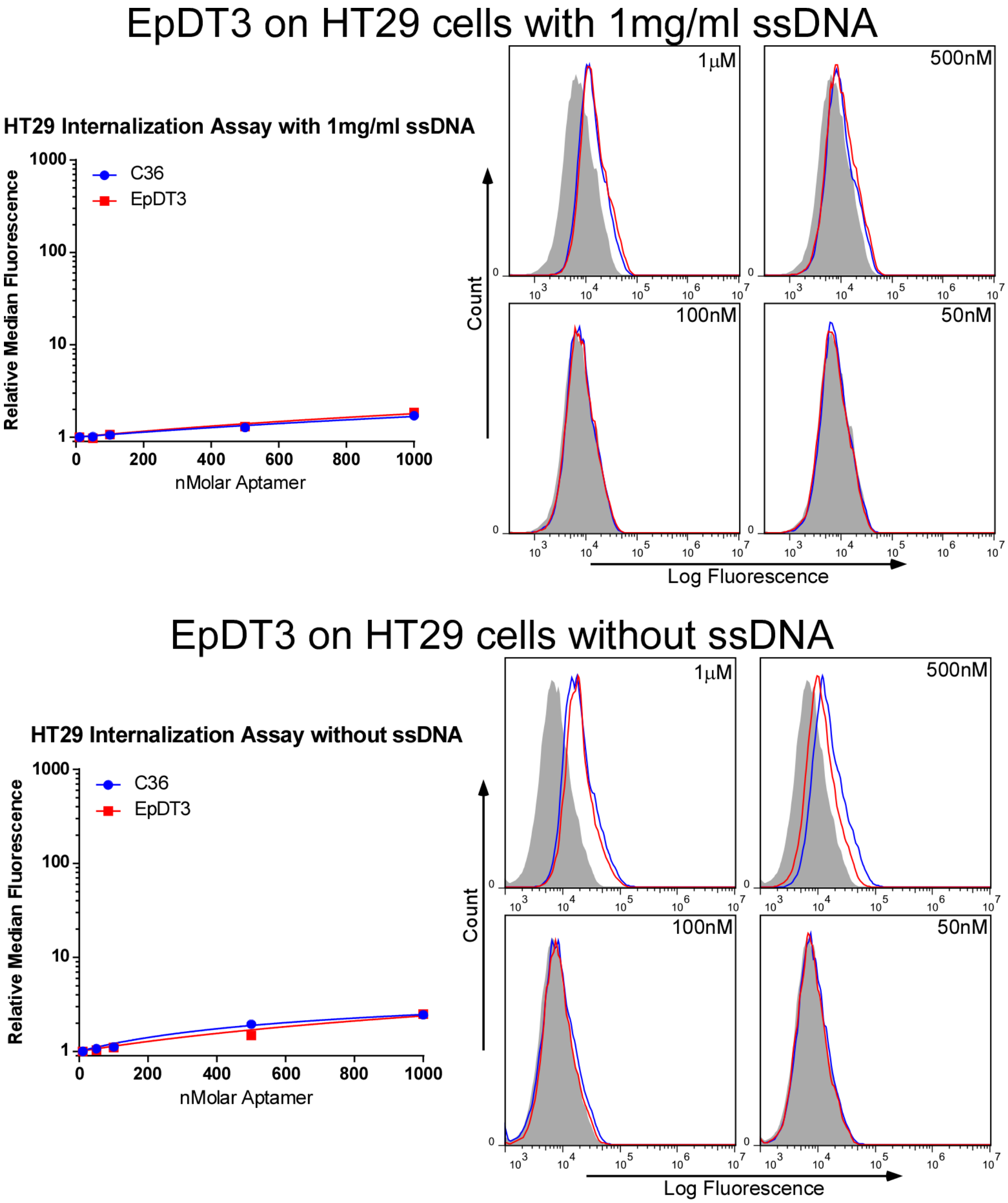

Supplementary Fig. 114. EpCAM targeted aptamer EpDT3 internalization and binding assays on HT29 cells. Graphs represent the median fluorescence of the aptamer (Red) and C36 (Blue) relative to unstained cells (Gray).

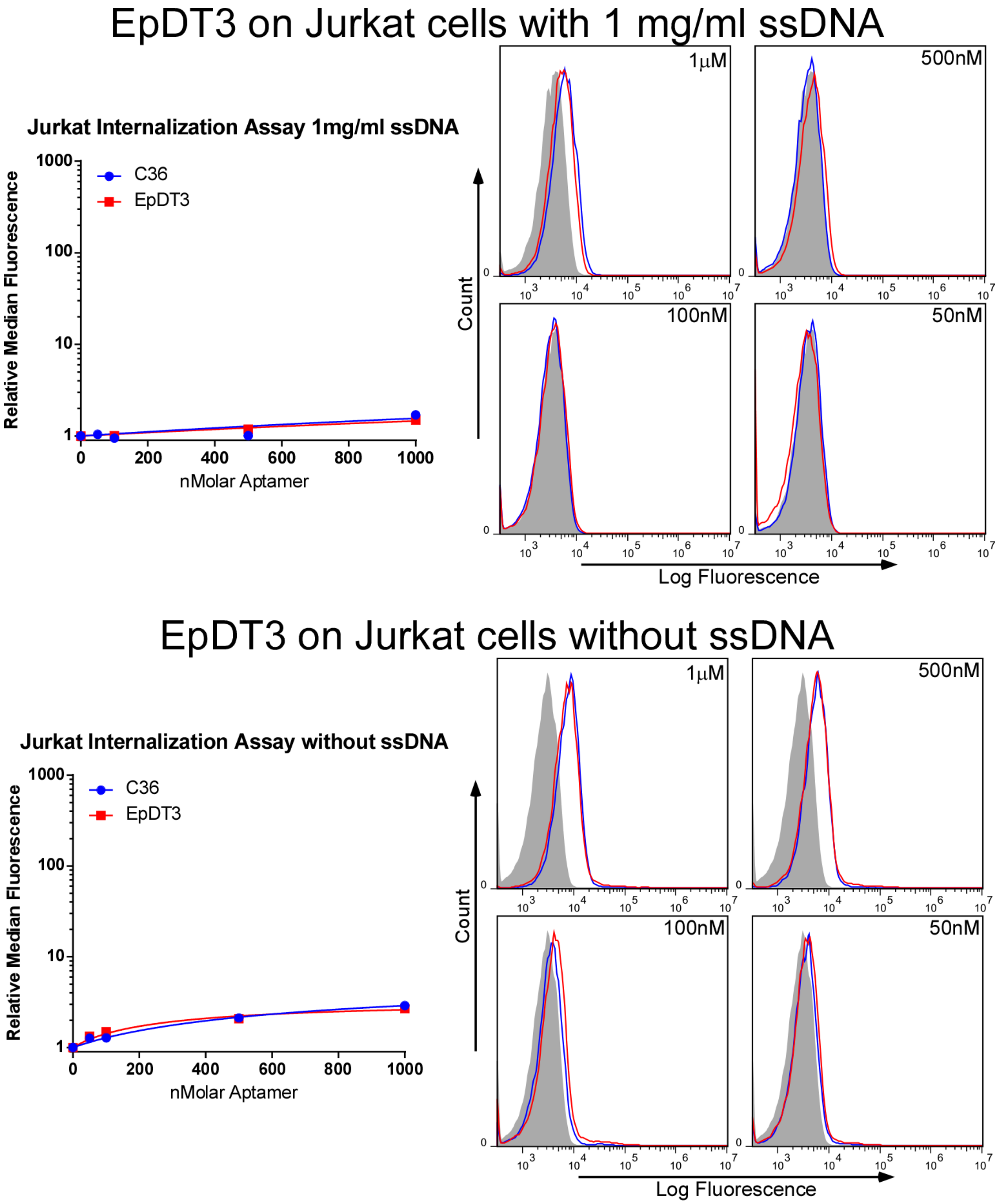

Supplementary Fig. 115. EpCAM targeted aptamer EpDT3 internalization and binding assays on Jurkat cells. Graphs represent the median fluorescence of the aptamer (Red) and C36 (Blue) relative to unstained cells (Gray).

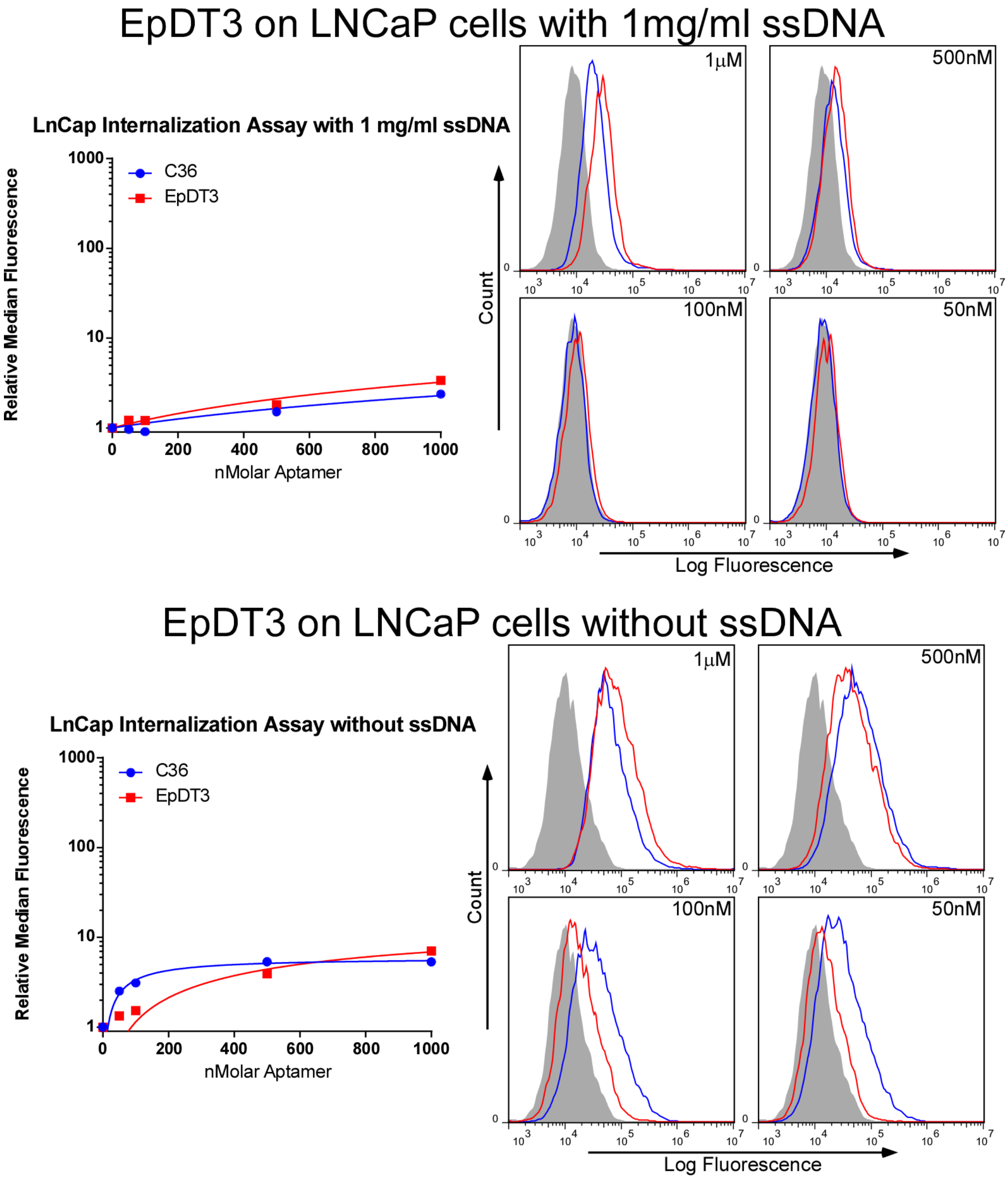

Supplementary Fig. 116. EpCAM targeted aptamer EpDT3 internalization and binding assays on LNCaP cells. Graphs represent the median fluorescence of the aptamer (Red) and C36 (Blue) relative to unstained cells (Gray).

EpDT3 on MCF7 cells with 1mg/ml ssDNA

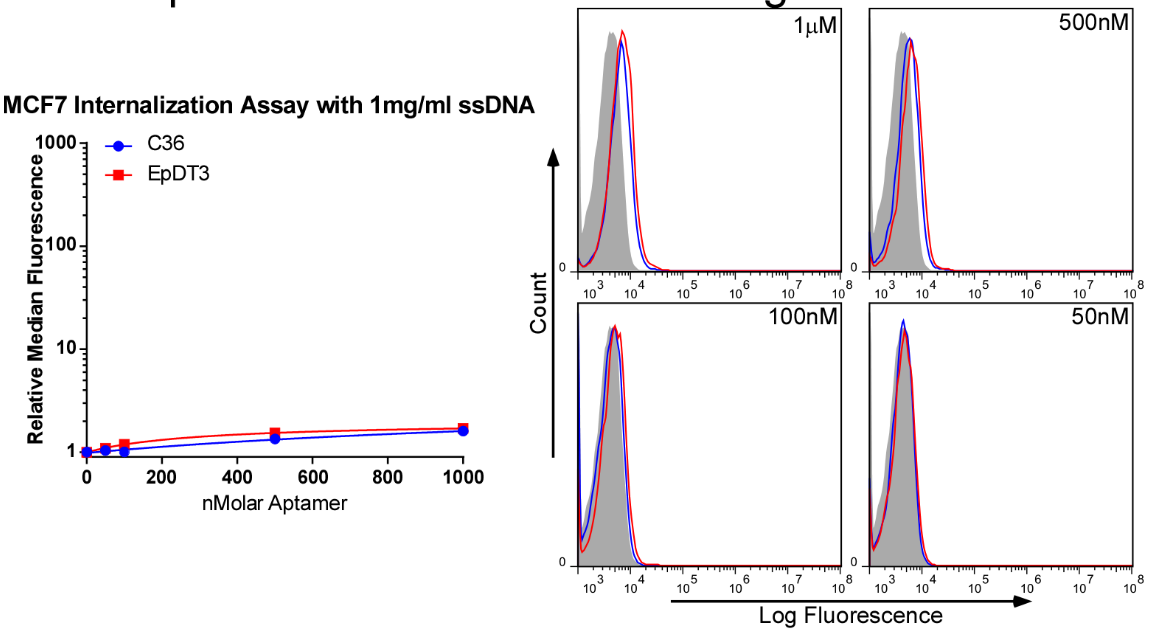

EpDT3 on MCF7 cells without ssDNA

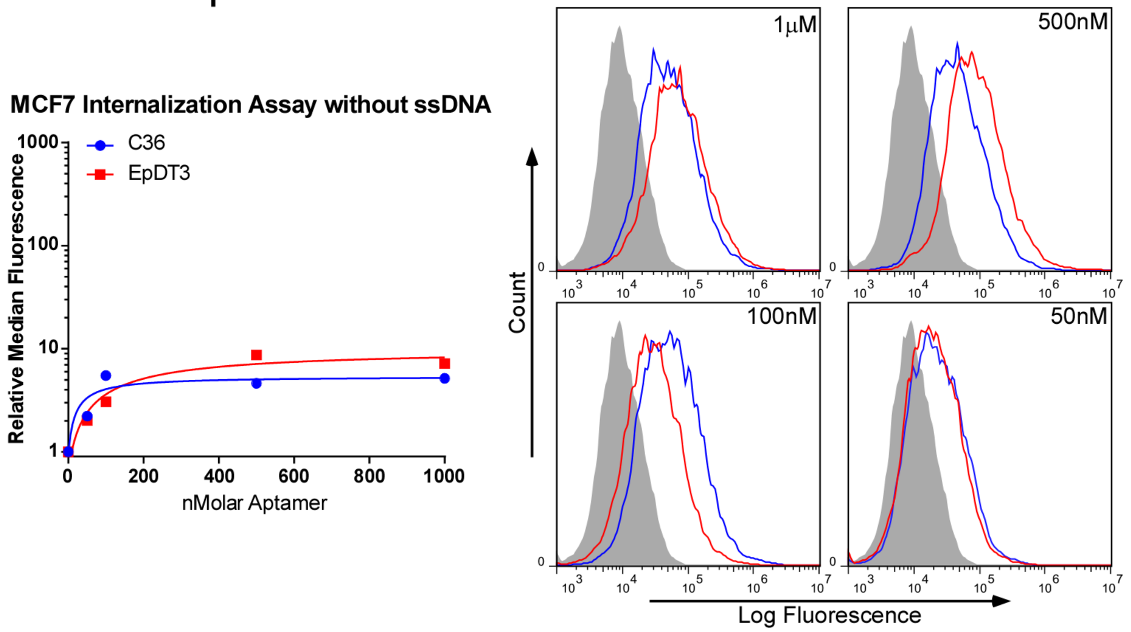

Supplementary Fig. 117. EpCAM targeted aptamer EpDT3 internalization and binding assays on MCF7 cells. Graphs represent the median fluorescence of the aptamer (Red) and C36 (Blue) relative to unstained cells (Gray).

EpDT3 on PC3 PSMA cells with 1mg/ml ssDNA

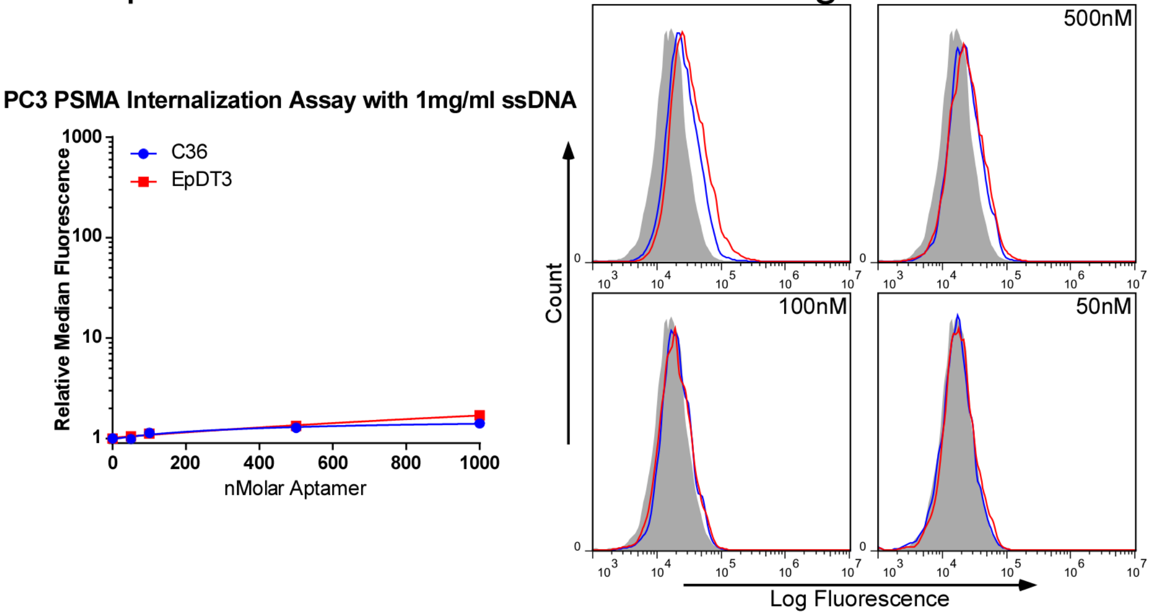

EpDT3 on PC3 PSMA cells without ssDNA

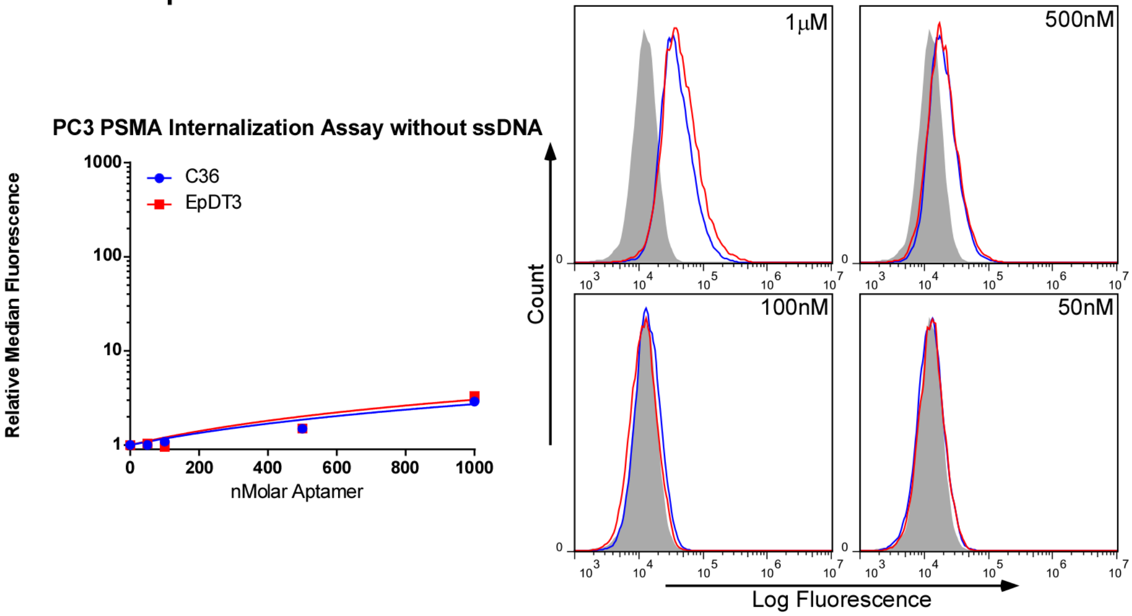

Supplementary Fig. 118. EpCAM targeted aptamer EpDT3 internalization and binding assays on PC3 PSMA cells. Graphs represent the median fluorescence of the aptamer (Red) and C36 (Blue) relative to unstained cells (Gray).

EpDT3 on PC3 cells with 1mg/ml ssDNA

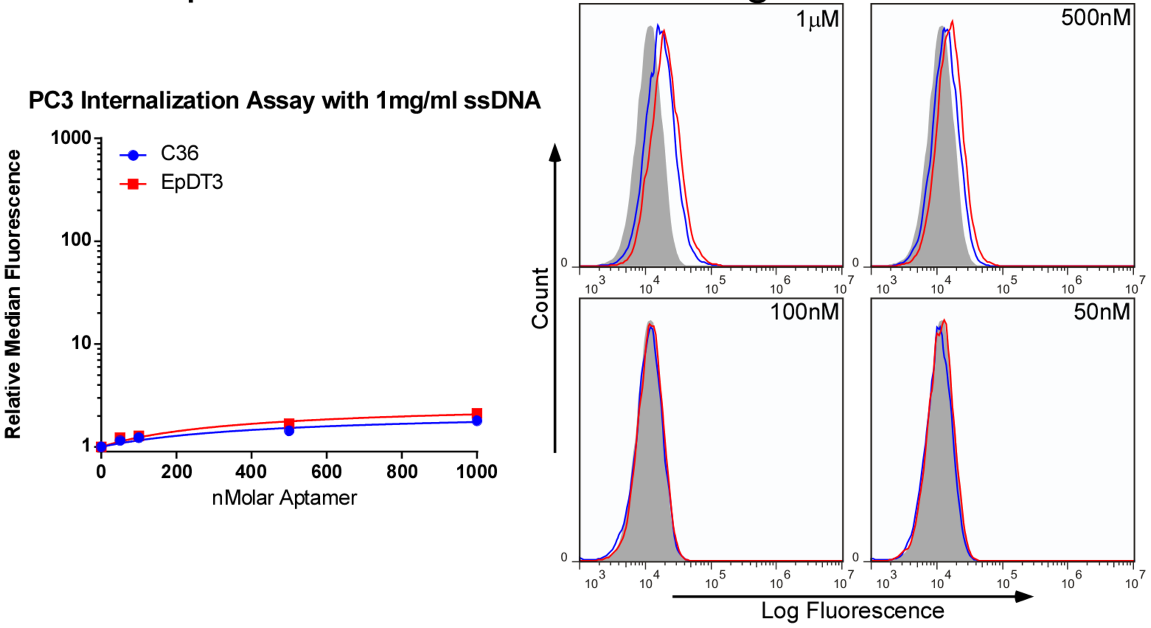

EpDT3 on PC3 cells without ssDNA

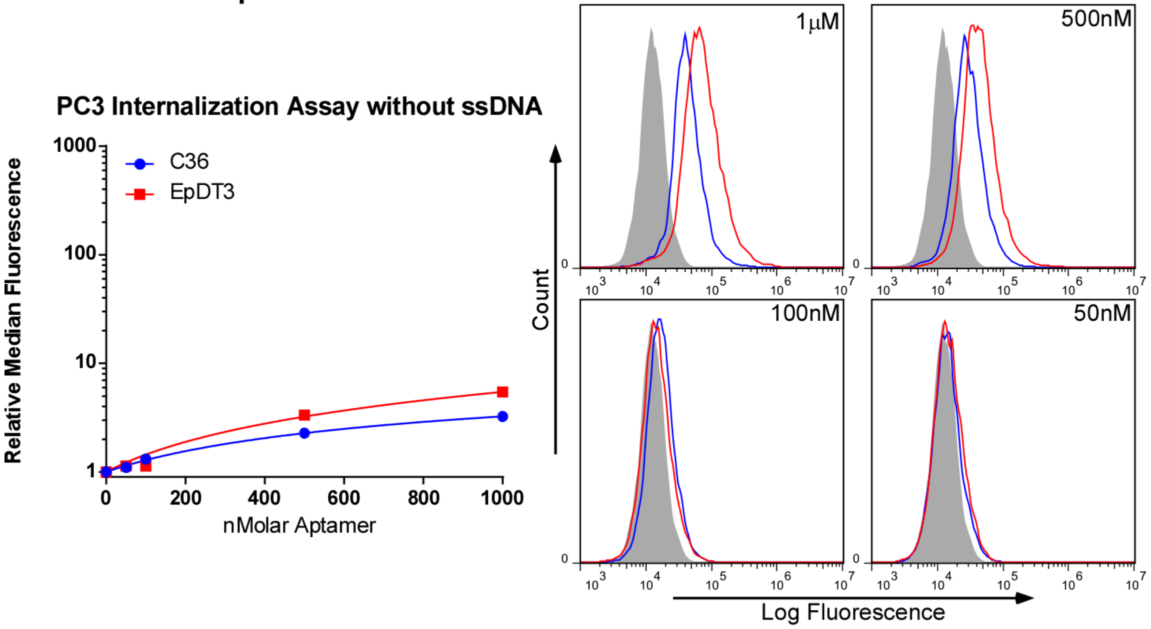

Supplementary Fig. 119. EpCAM targeted aptamer EpDT3 internalization and binding assays on PC3 cells. Graphs represent the median fluorescence of the aptamer (Red) and C36 (Blue) relative to unstained cells (Gray).

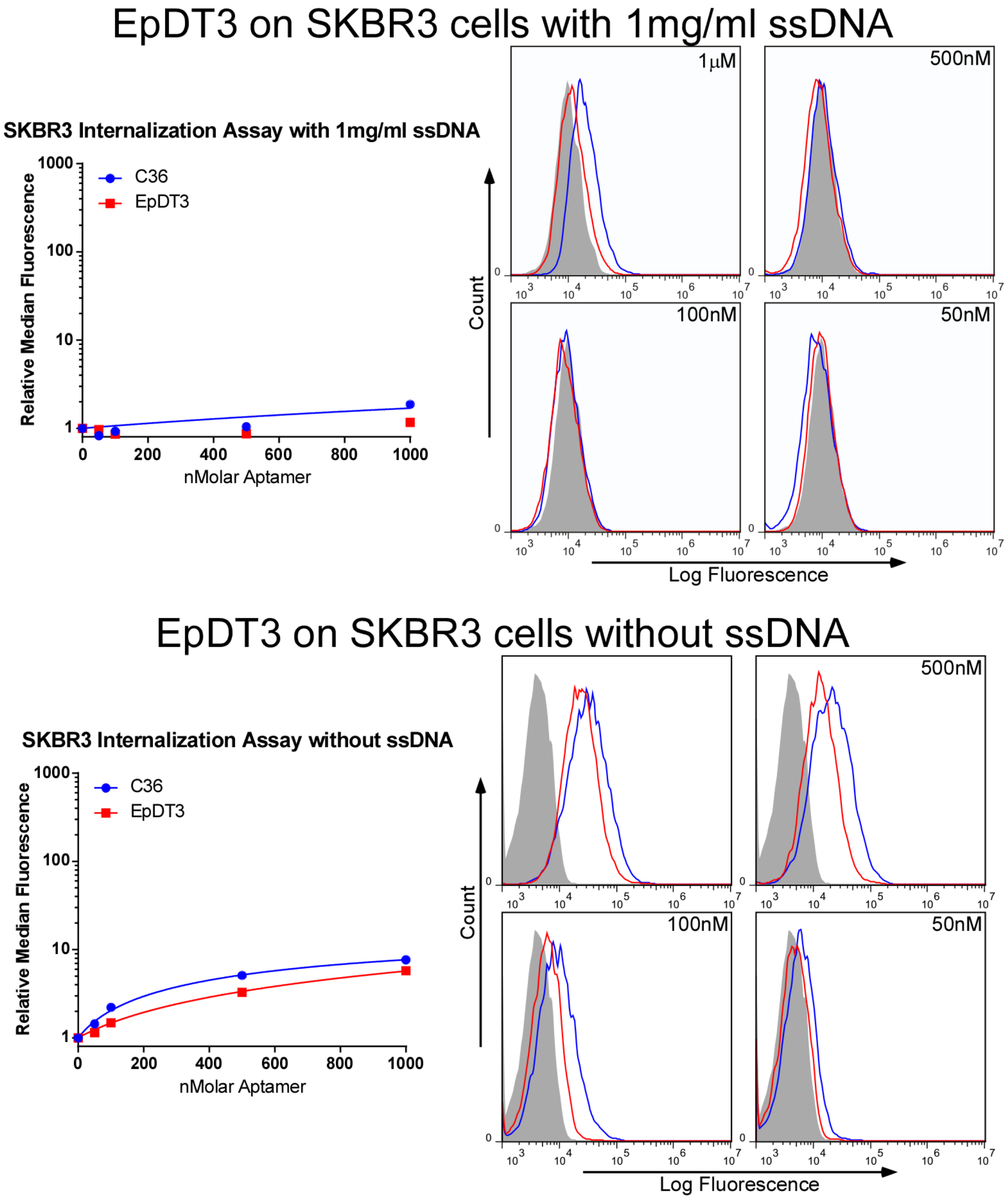

Supplementary Fig. 120. EpCAM targeted aptamer EpDT3 internalization and binding assays on SKBR3 cells. Graphs represent the median fluorescence of the aptamer (Red) and C36 (Blue) relative to unstained cells (Gray).

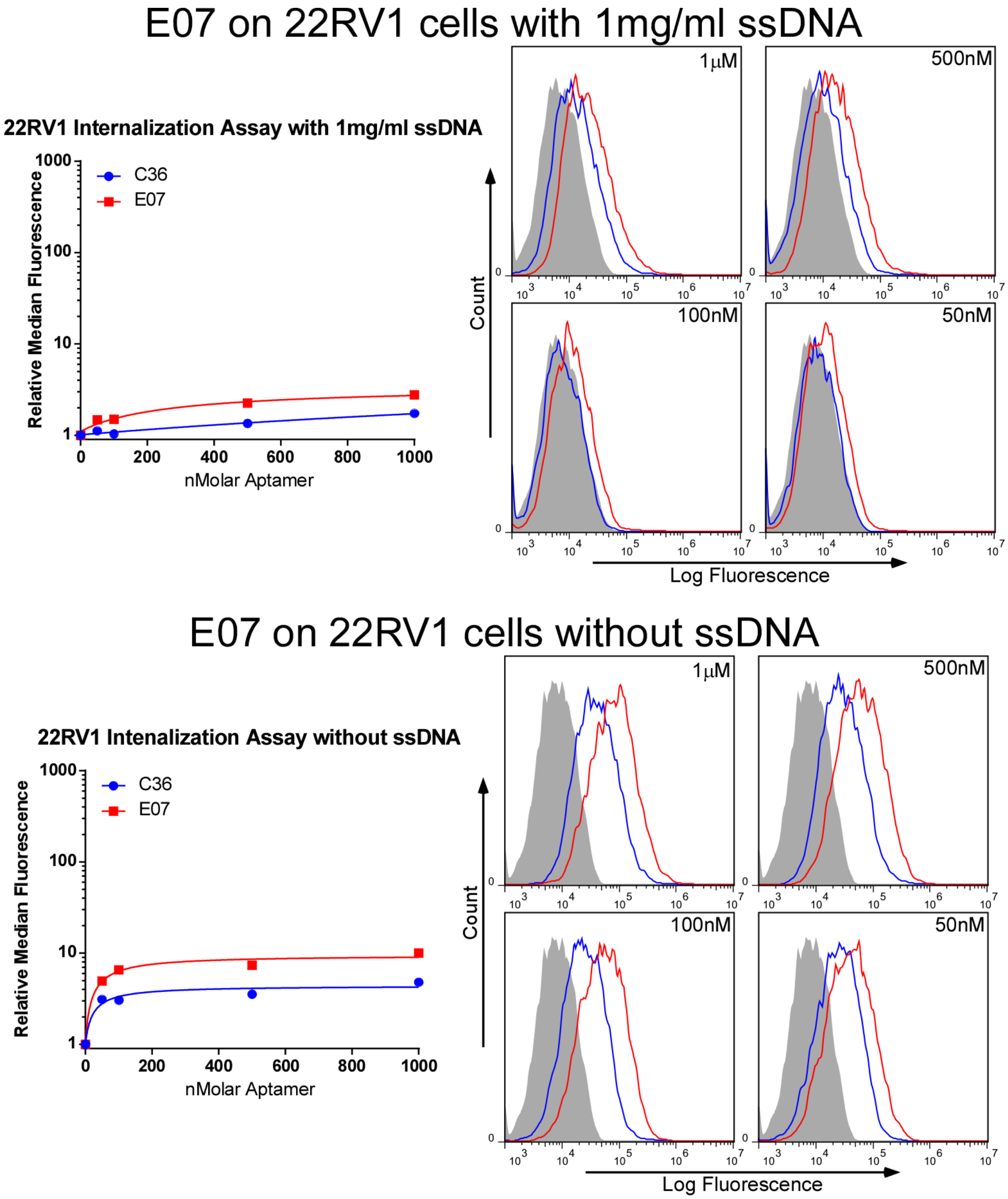

Supplementary Fig. 121. EGFR binding aptamer E07 internalization and binding assays on 22RV1 cells. Graphs represent the median fluorescence of the aptamer (Red) and C36 (Blue) relative to unstained cells (Gray).

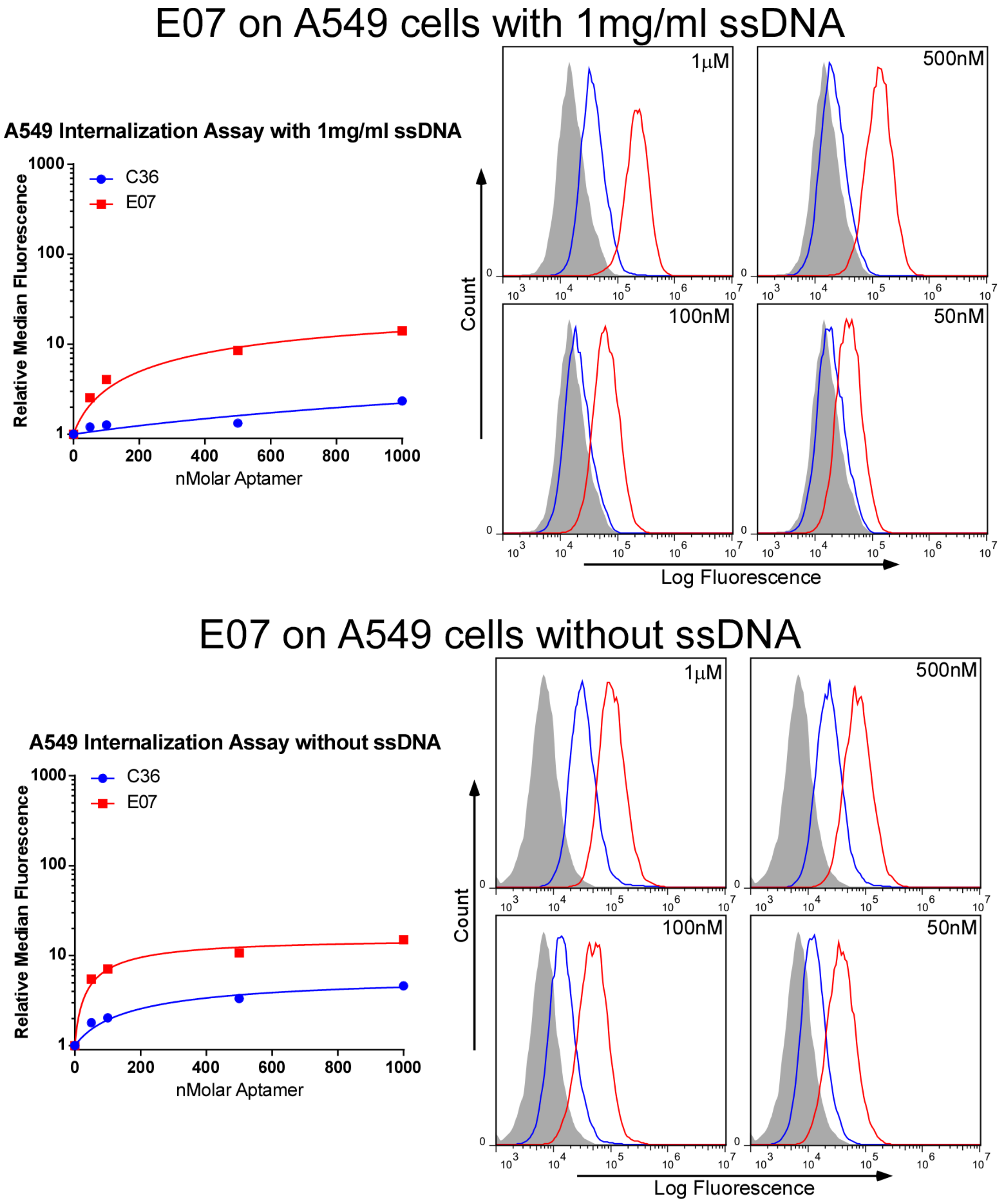

Supplementary Fig. 122. EGFR binding aptamer E07 internalization and binding assays on A549 cells. Graphs represent the median fluorescence of the aptamer (Red) and C36 (Blue) relative to unstained cells (Gray).

E07 on HeLa PSMA cells with 1mg/ml ssDNA

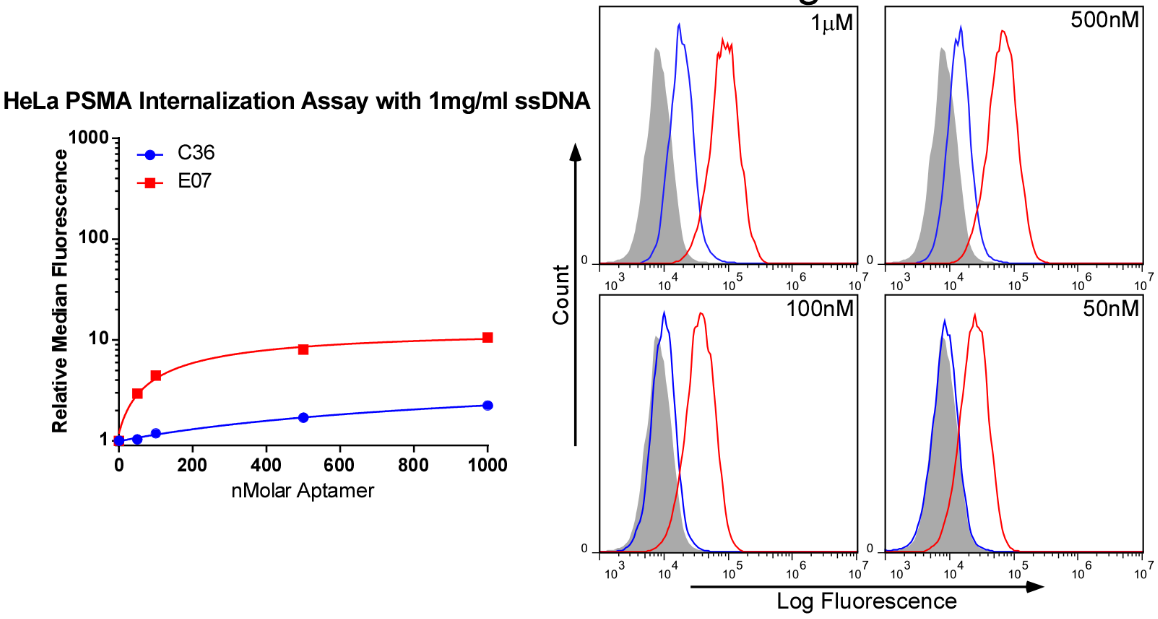

E07 on HeLa PSMA cells without ssDNA

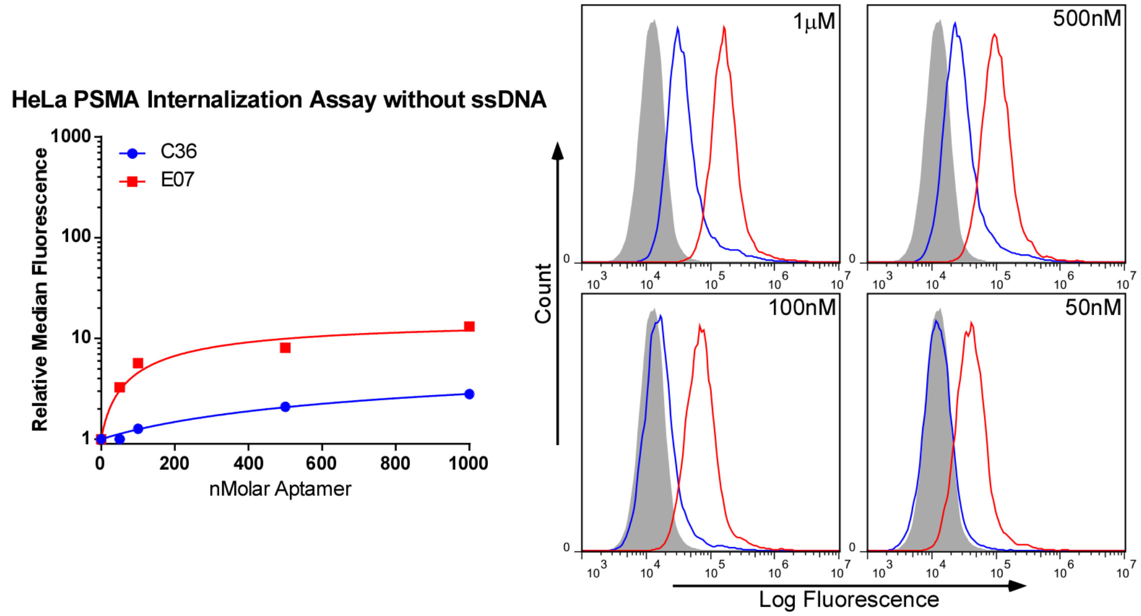

Supplementary Fig. 123. EGFR binding aptamer E07 internalization and binding assays on HeLa PSMA cells. Graphs represent the median fluorescence of the aptamer (Red) and C36 (Blue) relative to unstained cells (Gray).

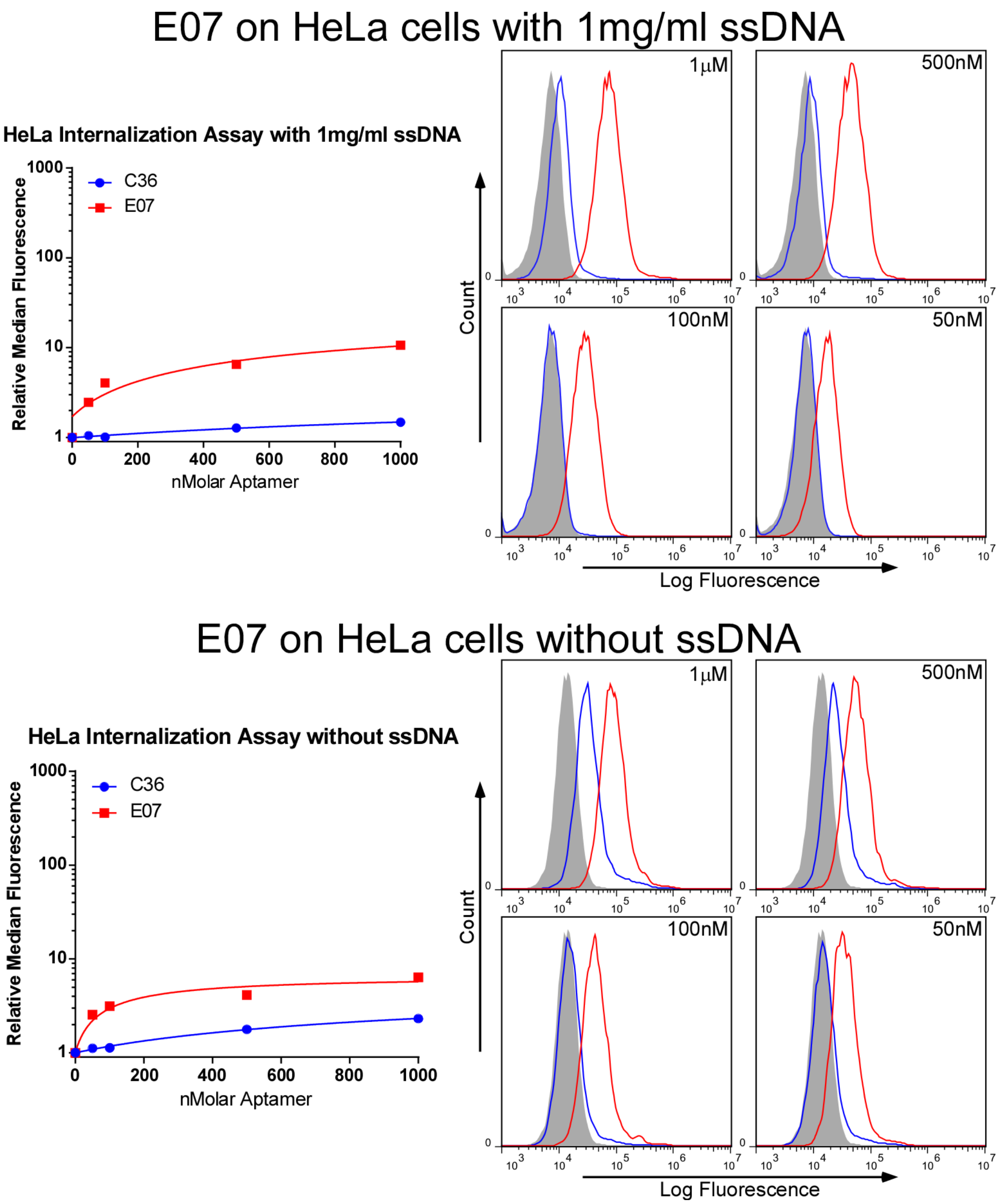

Supplementary Fig. 124. EGFR binding aptamer E07 internalization and binding assays on HeLa cells. Graphs represent the median fluorescence of the aptamer (Red) and C36 (Blue) relative to unstained cells (Gray).

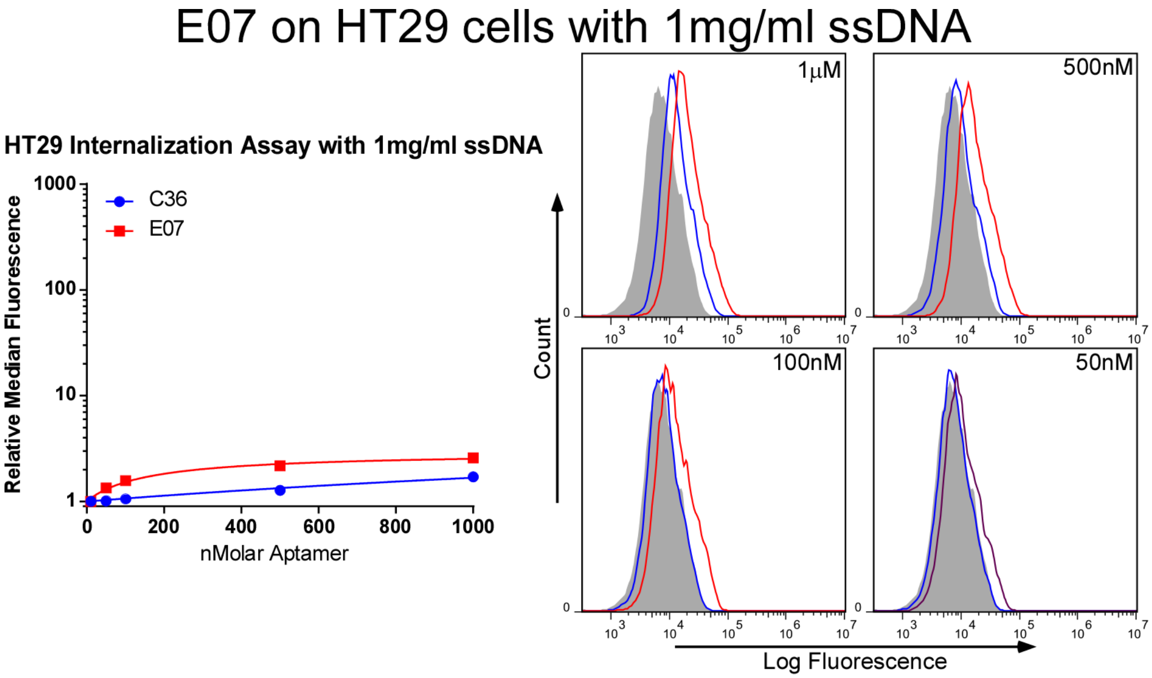

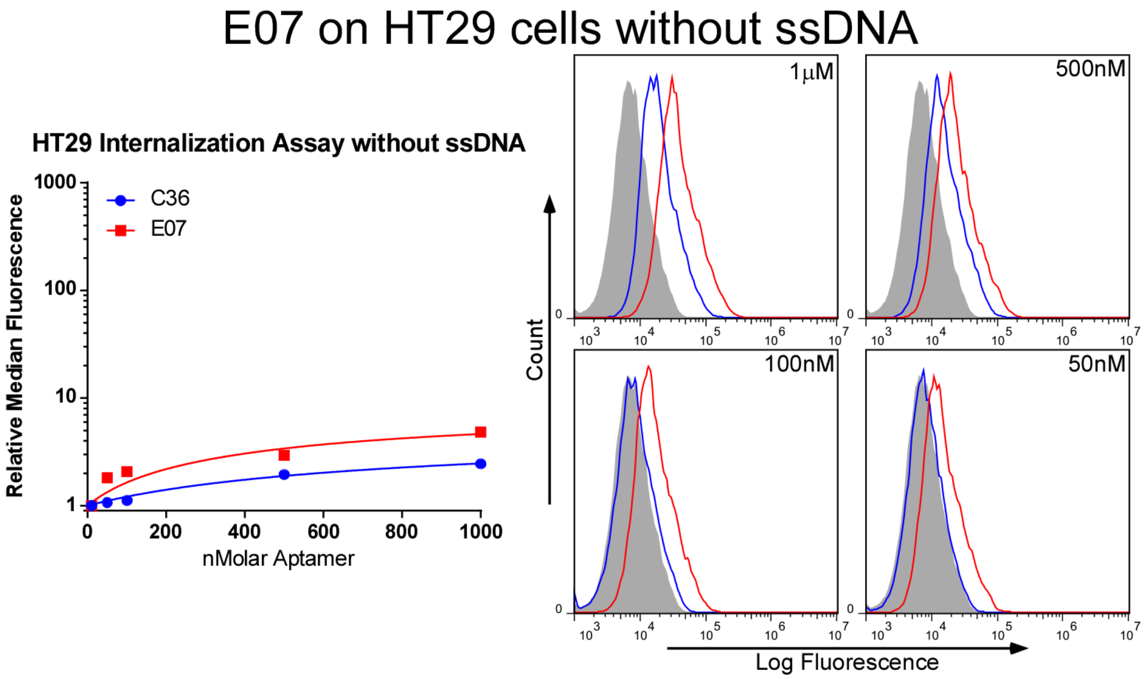

Supplementary Fig. 125. EGFR binding aptamer E07 internalization and binding assays on HT29 cells. Graphs represent the median fluorescence of the aptamer (Red) and C36 (Blue) relative to unstained cells (Gray).

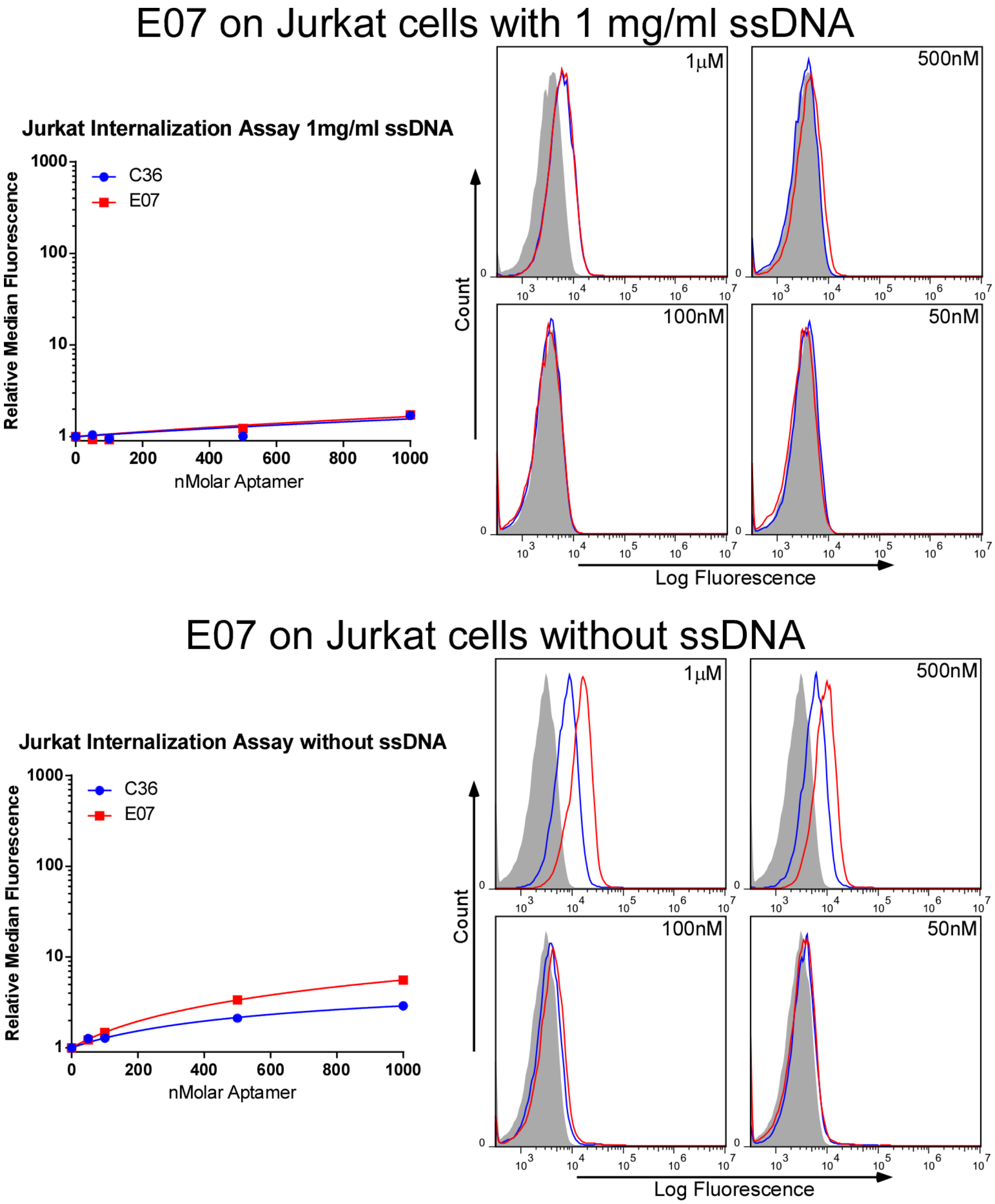

Supplementary Fig. 126. EGFR binding aptamer E07 internalization and binding assays on Jurkat cells. Graphs represent the median fluorescence of the aptamer (Red) and C36 (Blue) relative to unstained cells (Gray).

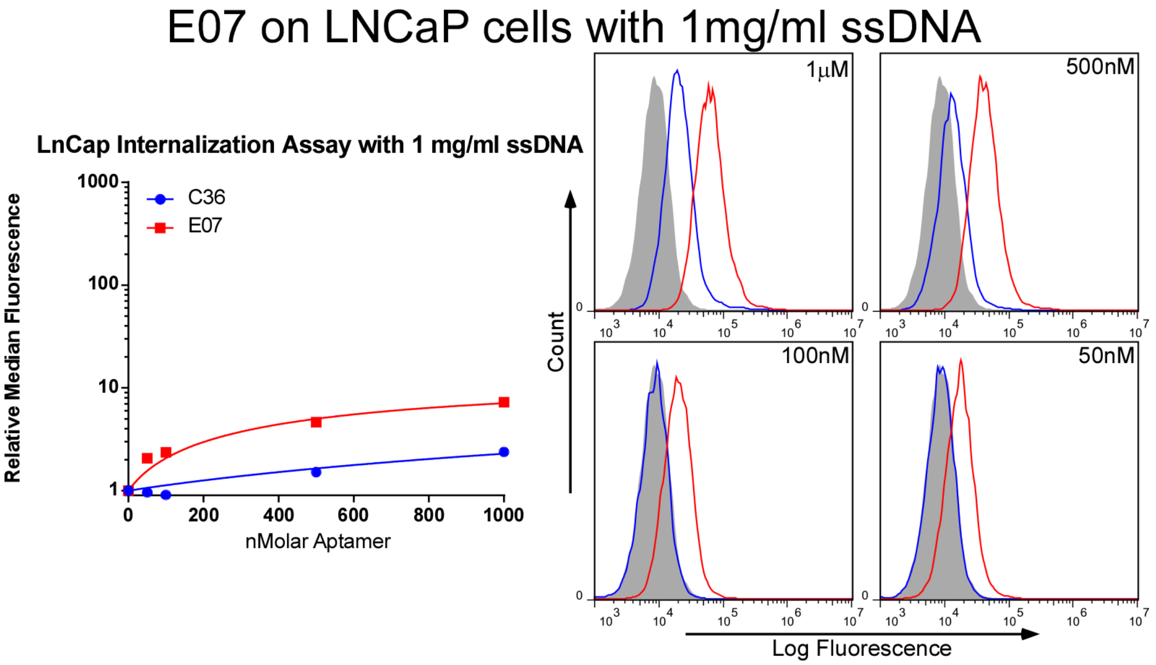

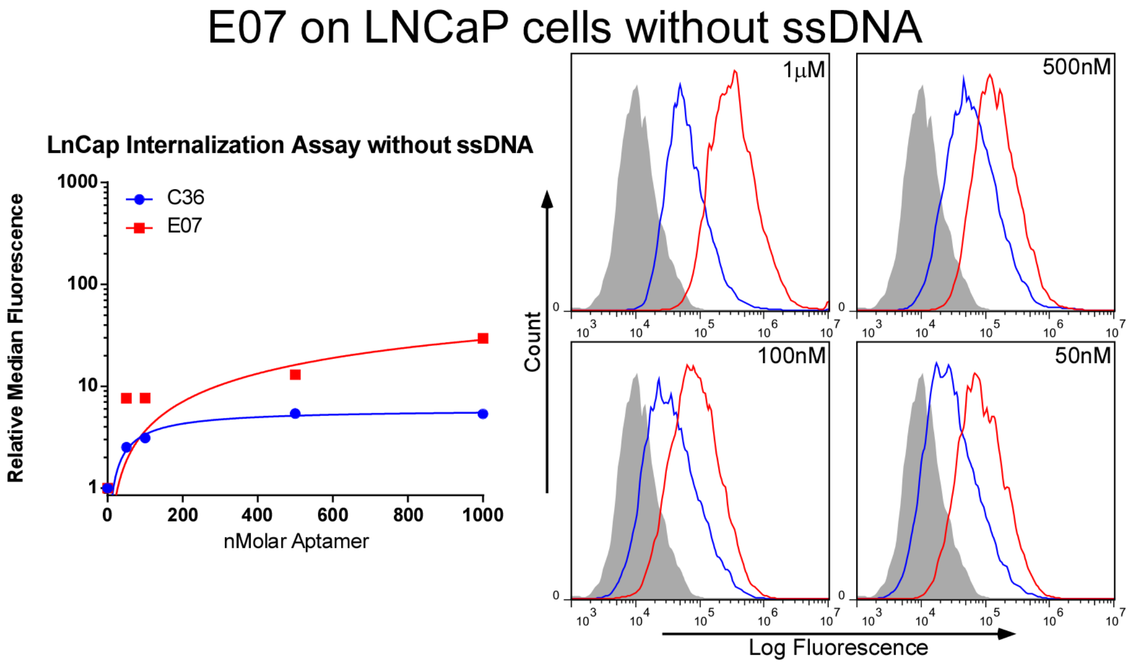

Supplementary Fig. 127. EGFR binding aptamer E07 internalization and binding assays on LNCaP cells. Graphs represent the median fluorescence of the aptamer (Red) and C36 (Blue) relative to unstained cells (Gray).

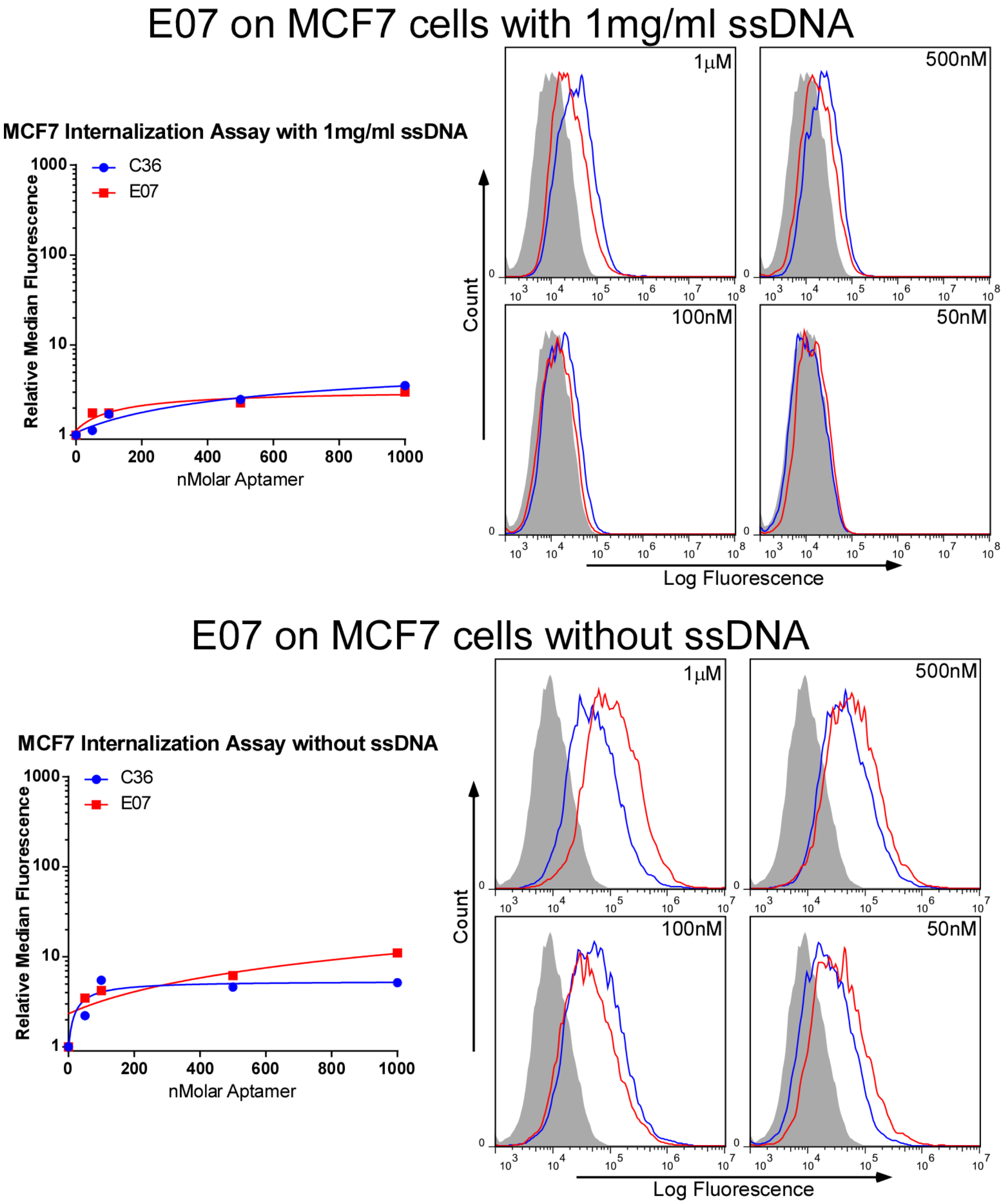

Supplementary Fig. 128. EGFR binding aptamer E07 internalization and binding assays on MCF7 cells. Graphs represent the median fluorescence of the aptamer (Red) and C36 (Blue) relative to unstained cells (Gray).

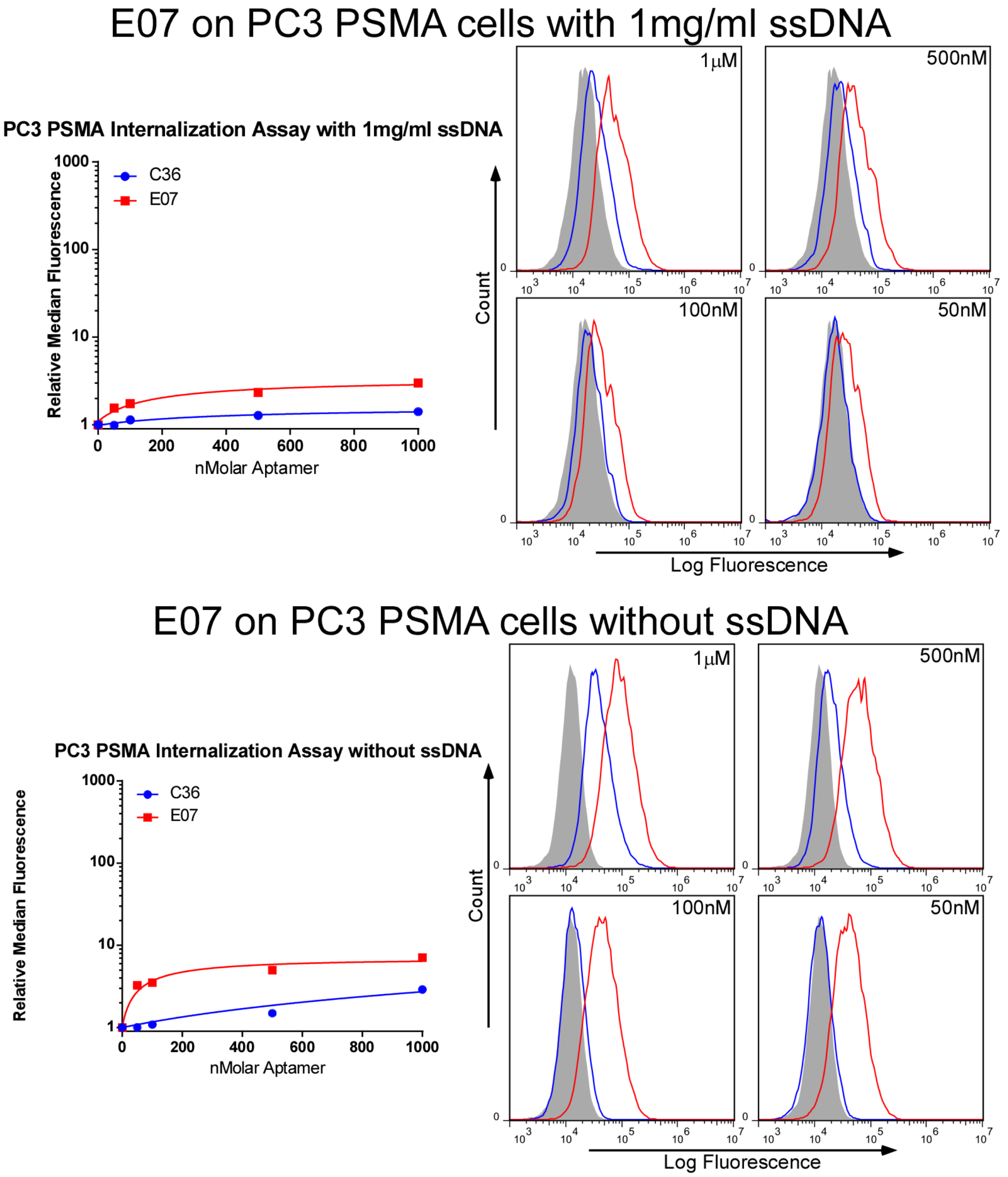

Supplementary Fig. 129. EGFR binding aptamer E07 internalization and binding assays on PC3 PSMA cells. Graphs represent the median fluorescence of the aptamer (Red) and C36 (Blue) relative to unstained cells (Gray).

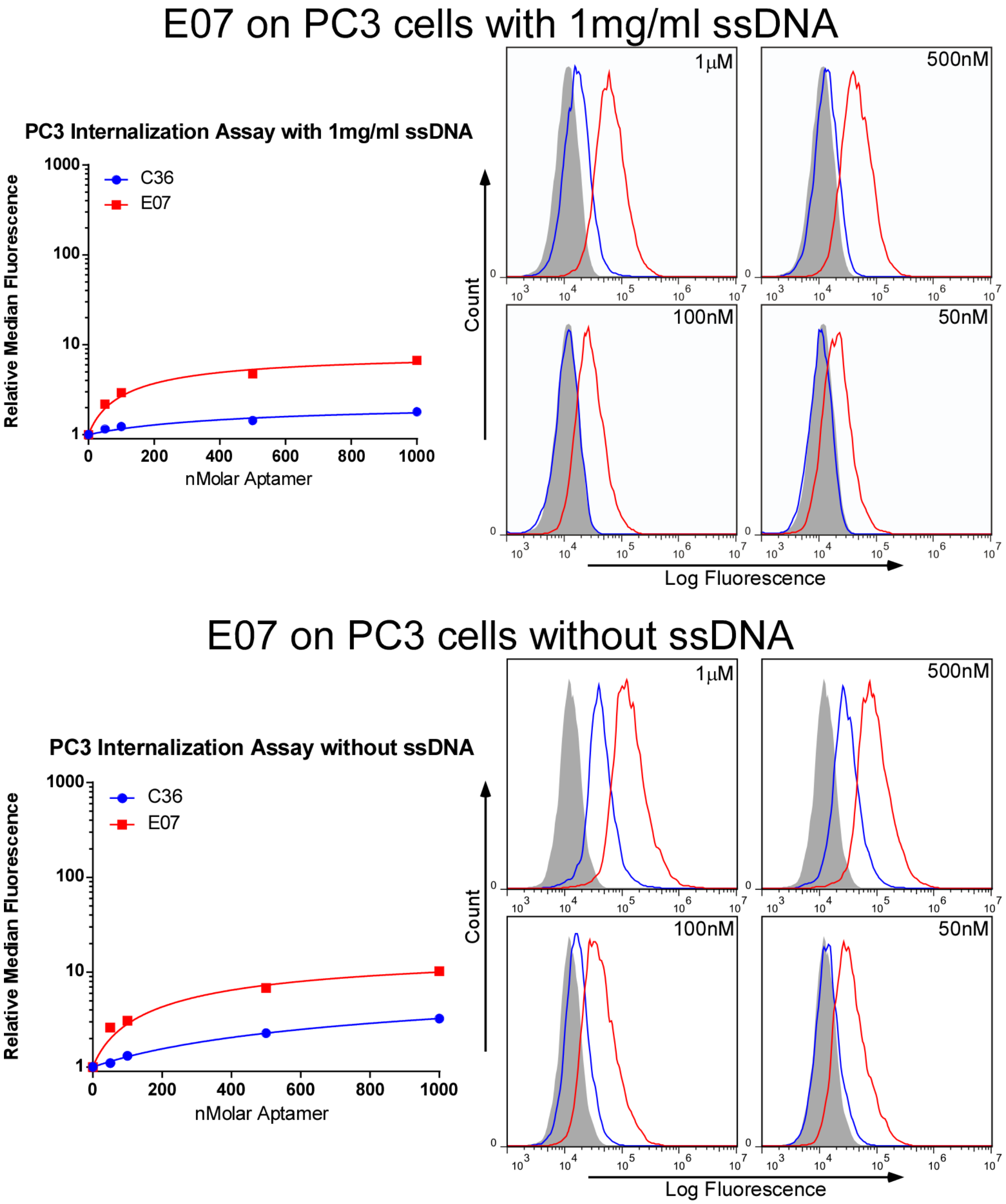

Supplementary Fig. 130. EGFR binding aptamer E07 internalization and binding assays on PC3 cells. Graphs represent the median fluorescence of the aptamer (Red) and C36 (Blue) relative to unstained cells (Gray).

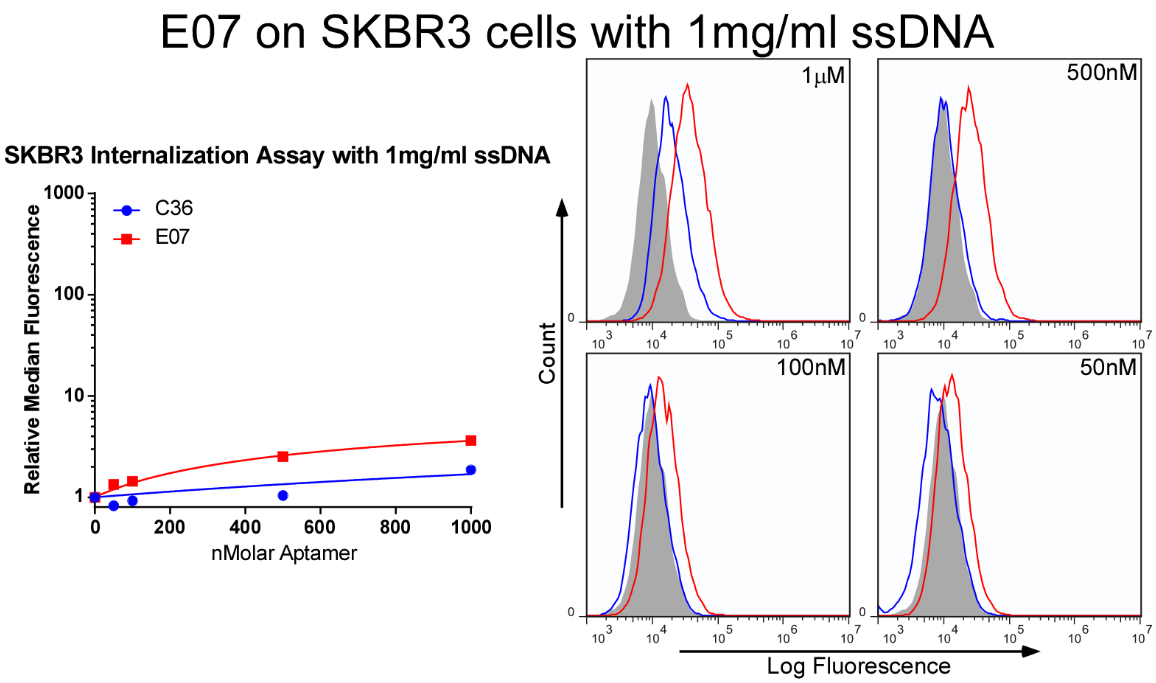

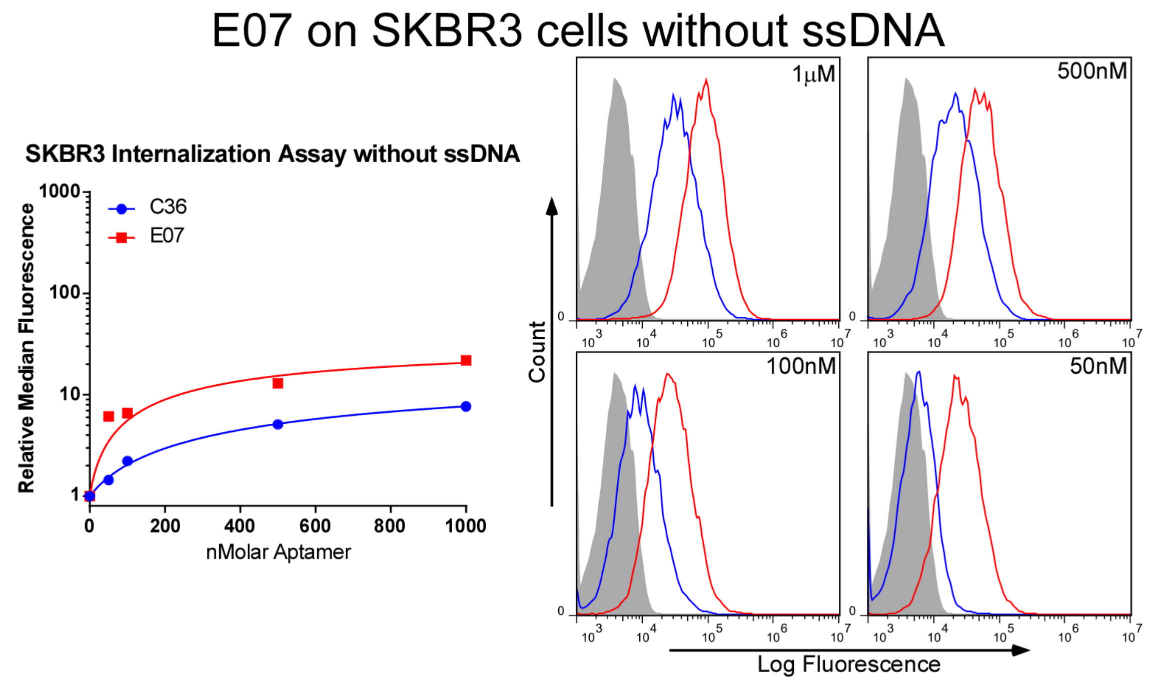

Supplementary Fig. 131. EGFR binding aptamer E07 internalization and binding assays on SKBR3 cells. Graphs represent the median fluorescence of the aptamer (Red) and C36 (Blue) relative to unstained cells (Gray).

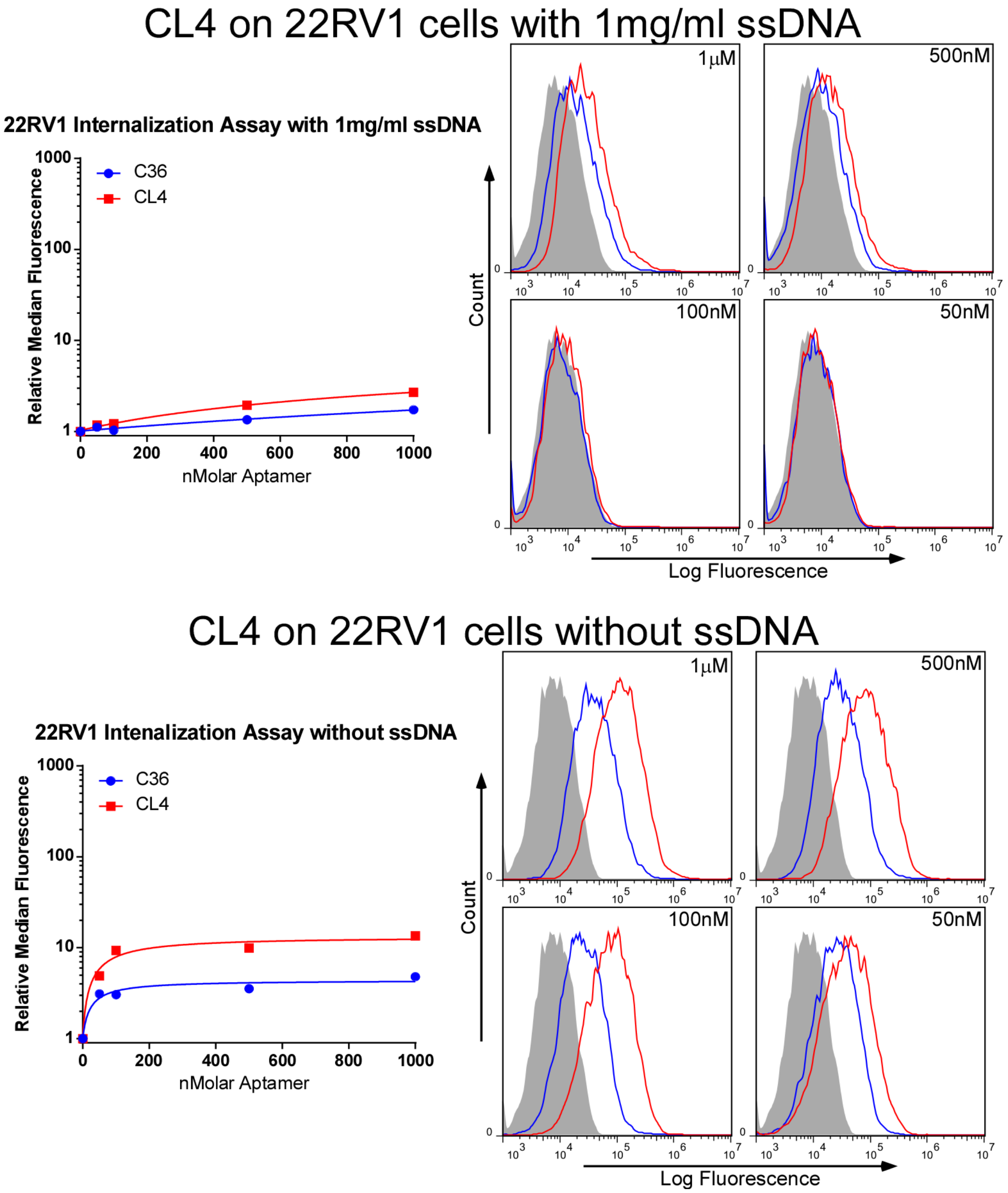

Supplementary Fig. 132. EGFR targeted aptamer CL4 internalization and binding assays on 22RV1 cells. Graphs represent the median fluorescence of the aptamer (Red) and C36 (Blue) relative to unstained cells (Gray).

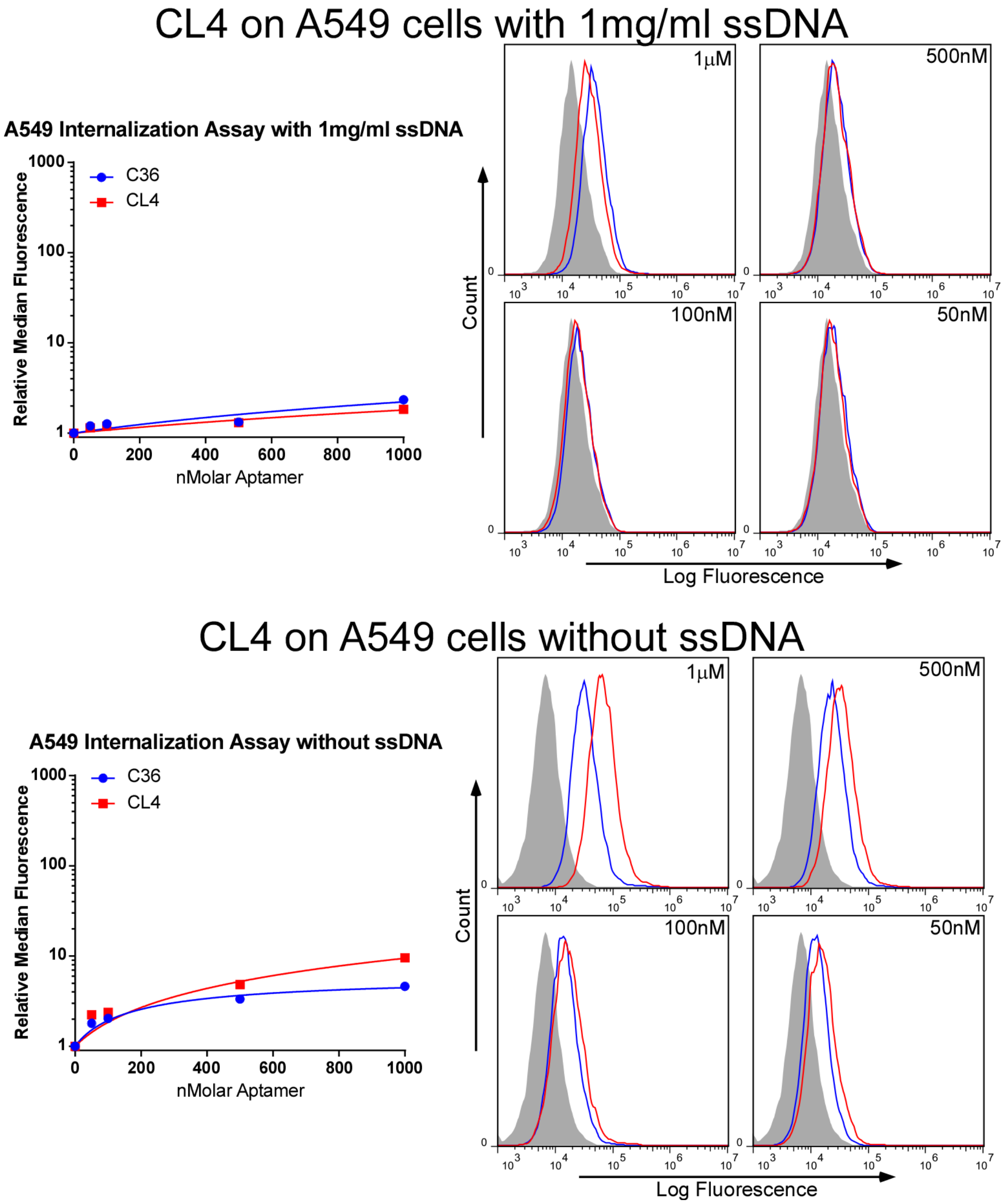

Supplementary Fig. 133. EGFR targeted aptamer CL4 internalization and binding assays on A549 cells. Graphs represent the median fluorescence of the aptamer (Red) and C36 (Blue) relative to unstained cells (Gray).

CL4 on HeLa PSMA cells with 1mg/ml ssDNA

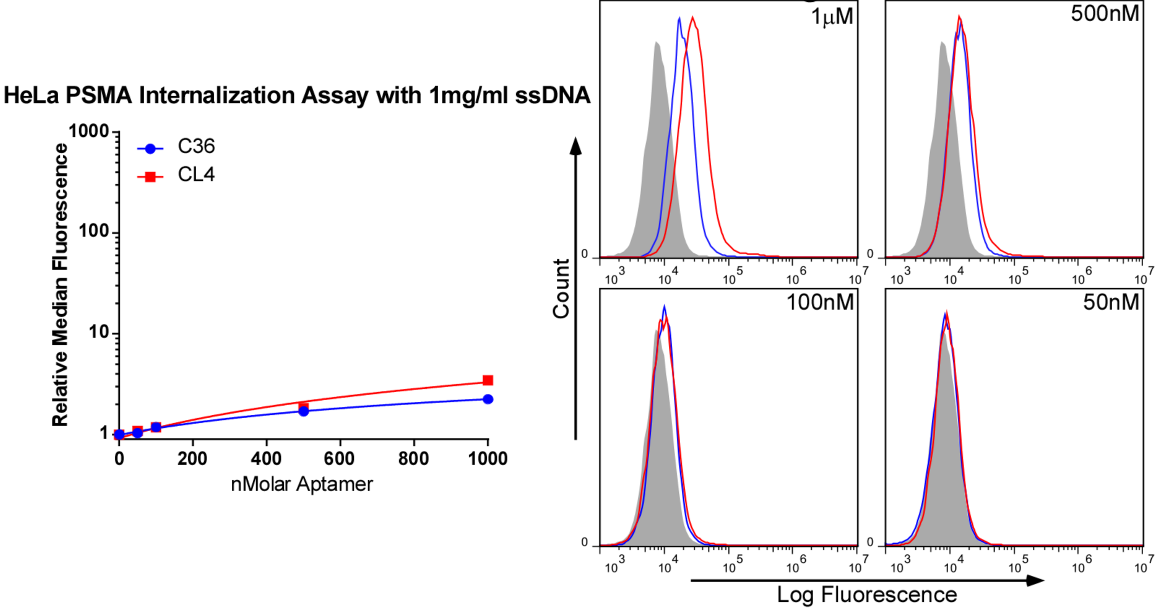

CL4 on HeLa PSMA cells without ssDNA

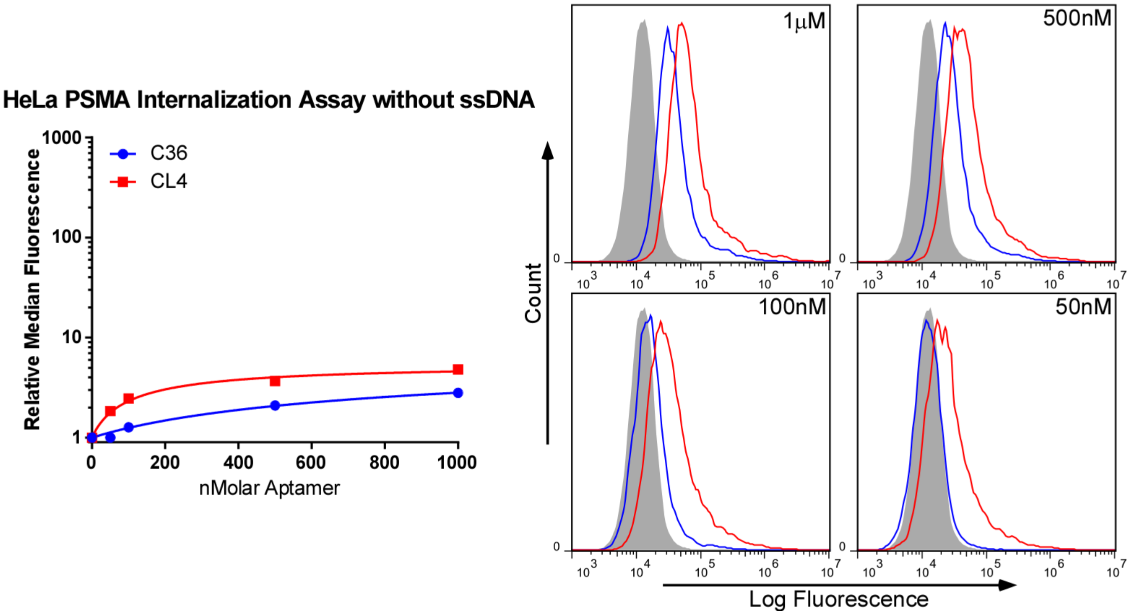

Supplementary Fig. 134. EGFR targeted aptamer CL4 internalization and binding assays on HeLa PSMA cells. Graphs represent the median fluorescence of the aptamer (Red) and C36 (Blue) relative to unstained cells (Gray).

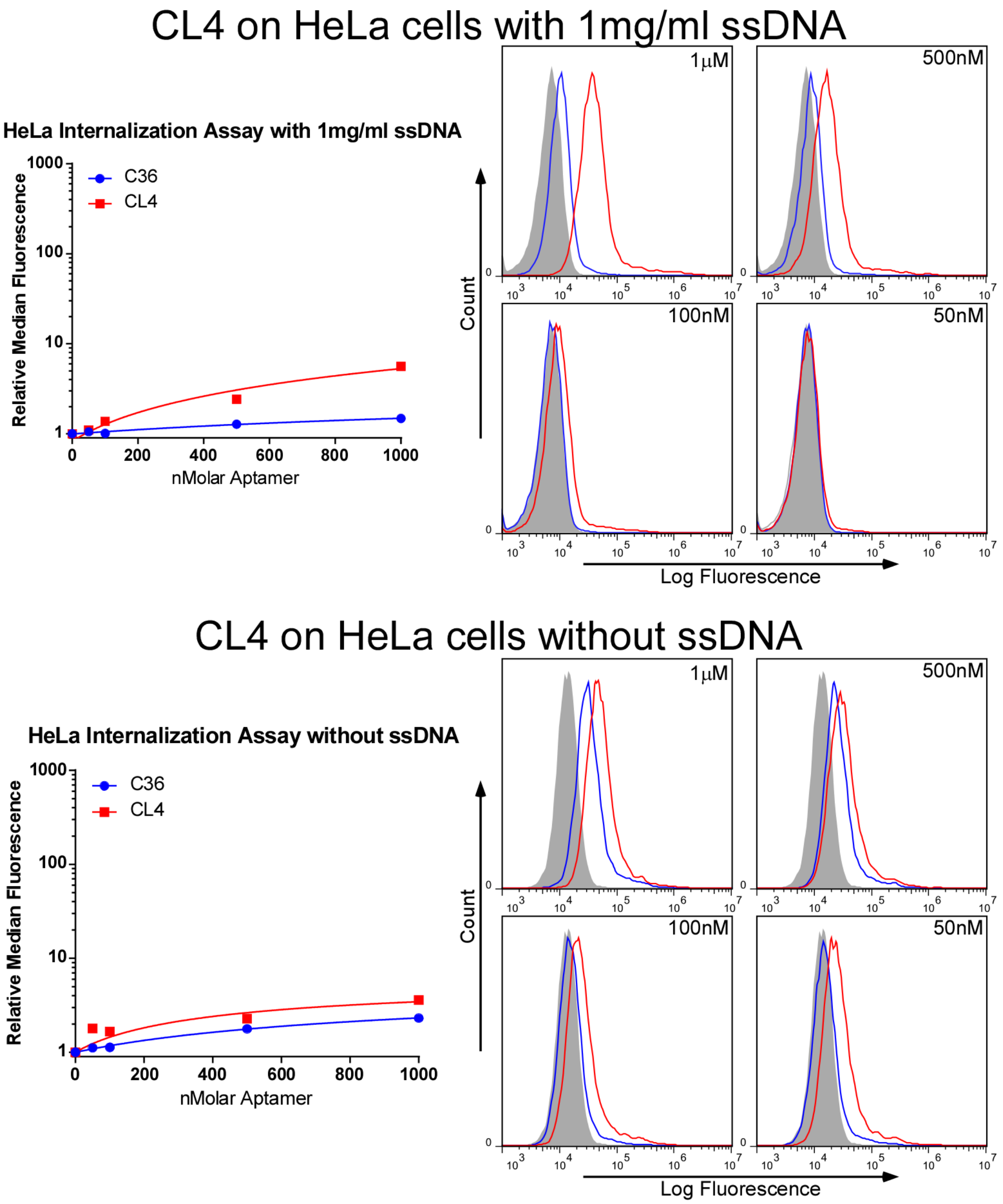

Supplementary Fig. 135. EGFR targeted aptamer CL4 internalization and binding assays on HeLa cells. Graphs represent the median fluorescence of the aptamer (Red) and C36 (Blue) relative to unstained cells (Gray).

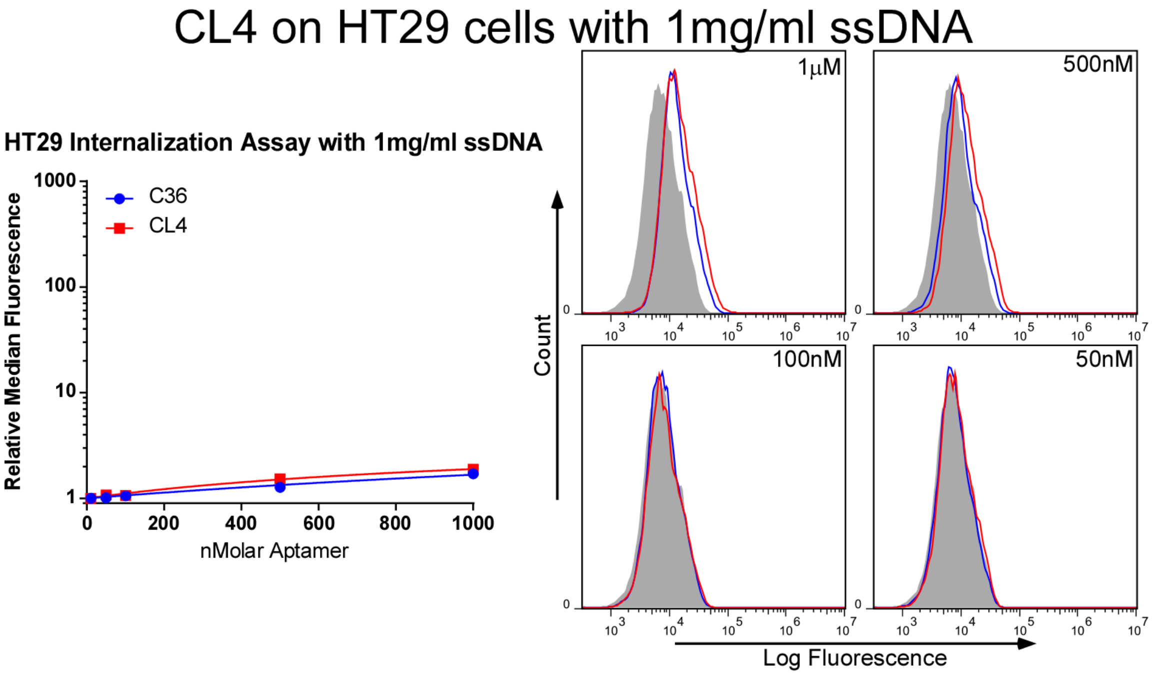

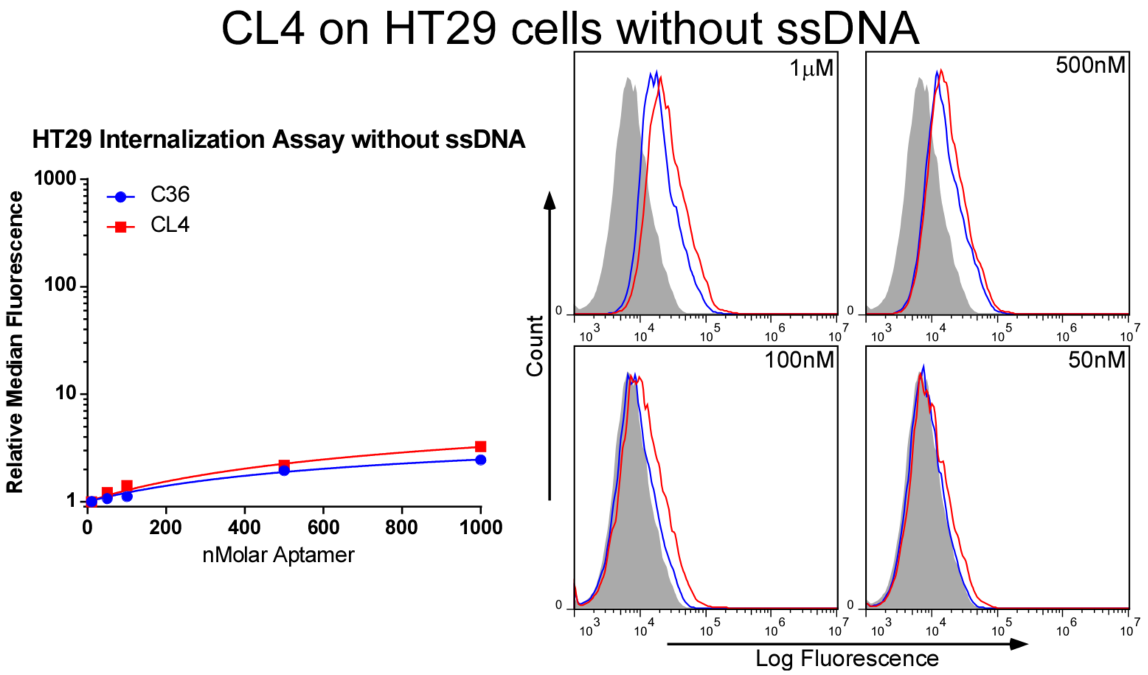

Supplementary Fig. 136. EGFR targeted aptamer CL4 internalization and binding assays on HT29 cells. Graphs represent the median fluorescence of the aptamer (Red) and C36 (Blue) relative to unstained cells (Gray).

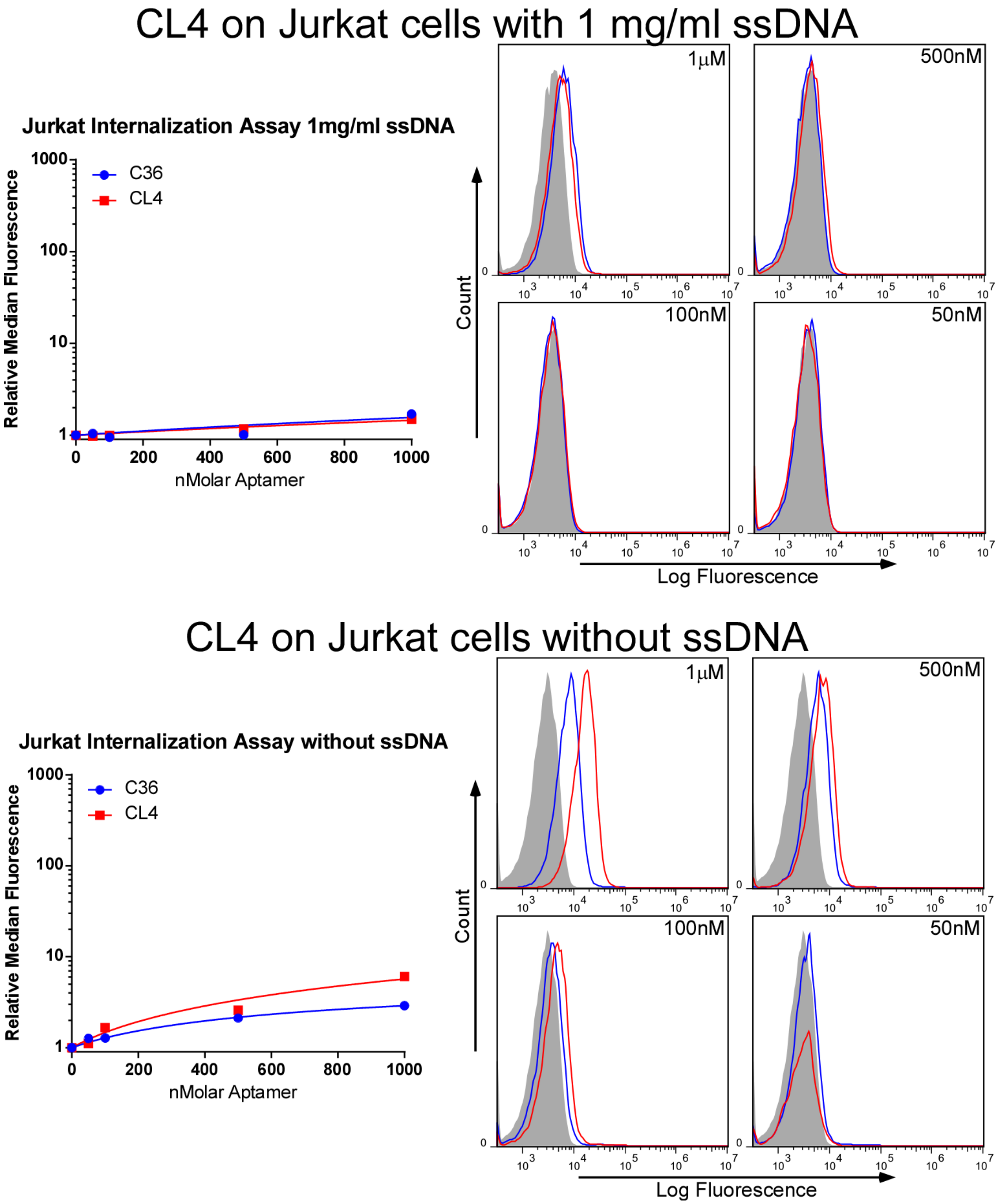

Supplementary Fig. 137. EGFR targeted aptamer CL4 internalization and binding assays on Jurkat cells. Graphs represent the median fluorescence of the aptamer (Red) and C36 (Blue) relative to unstained cells (Gray).

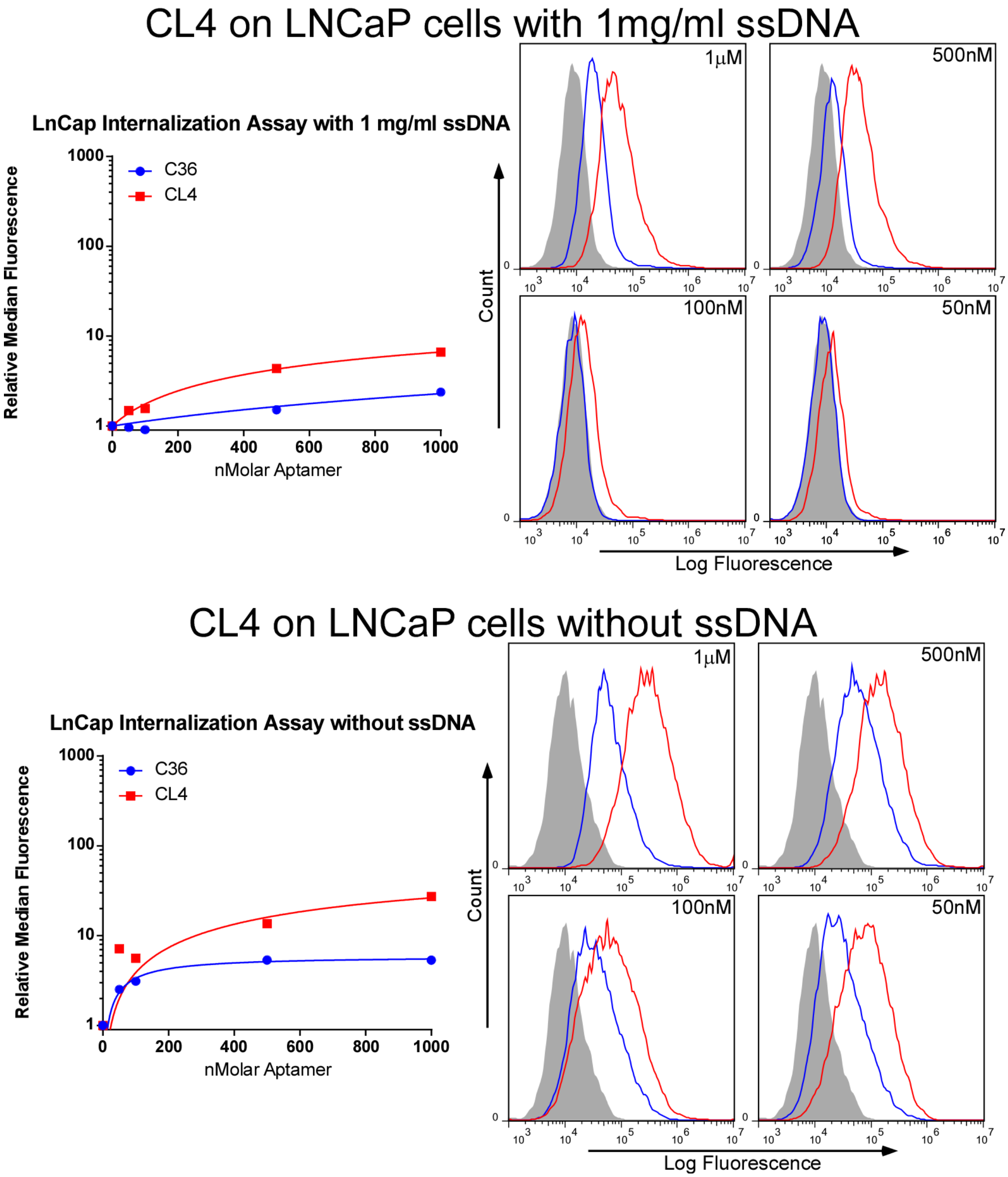

Supplementary Fig. 138. EGFR targeted aptamer CL4 internalization and binding assays on LNCaP cells. Graphs represent the median fluorescence of the aptamer (Red) and C36 (Blue) relative to unstained cells (Gray).

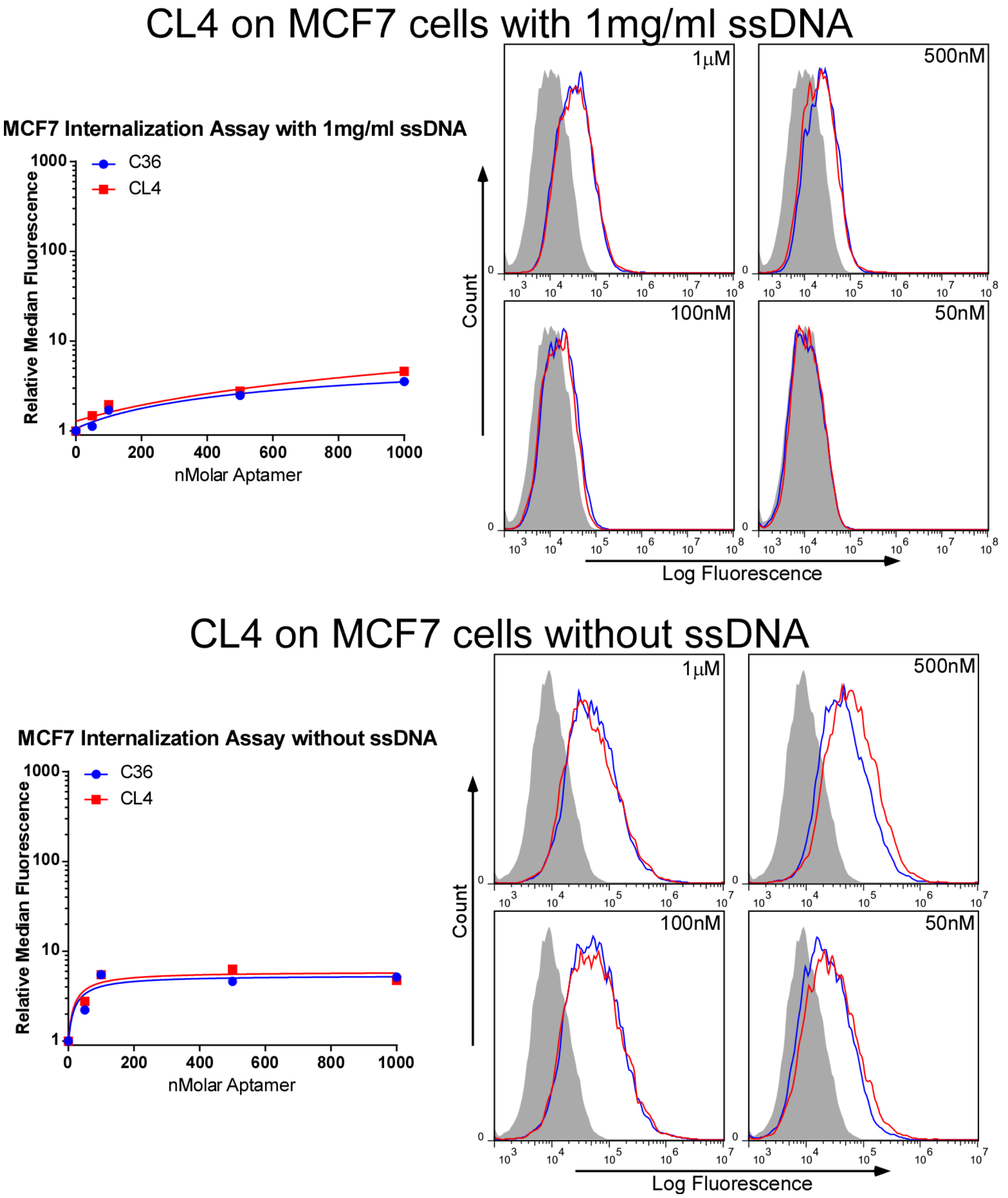

Supplementary Fig. 139. EGFR targeted aptamer CL4 internalization and binding assays on MCF7cells. Graphs represent the median fluorescence of the aptamer (Red) and C36 (Blue) relative to unstained cells (Gray).

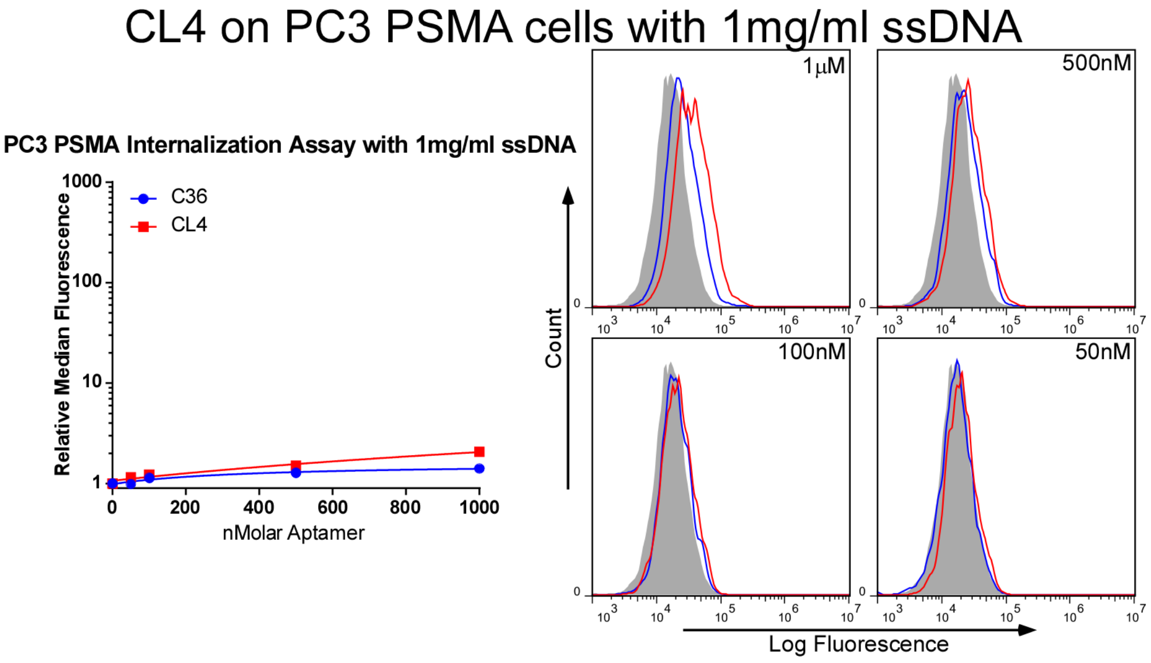

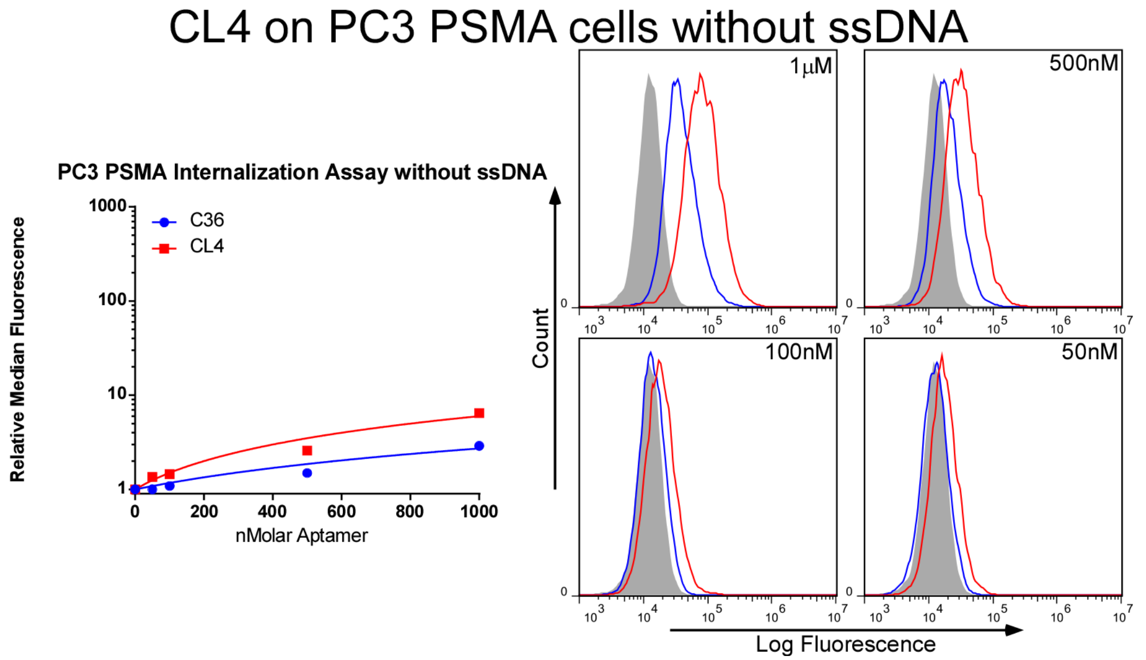

Supplementary Fig. 140. EGFR targeted aptamer CL4 internalization and binding assays on PC3 PSMA cells. Graphs represent the median fluorescence of the aptamer (Red) and C36 (Blue) relative to unstained cells (Gray).

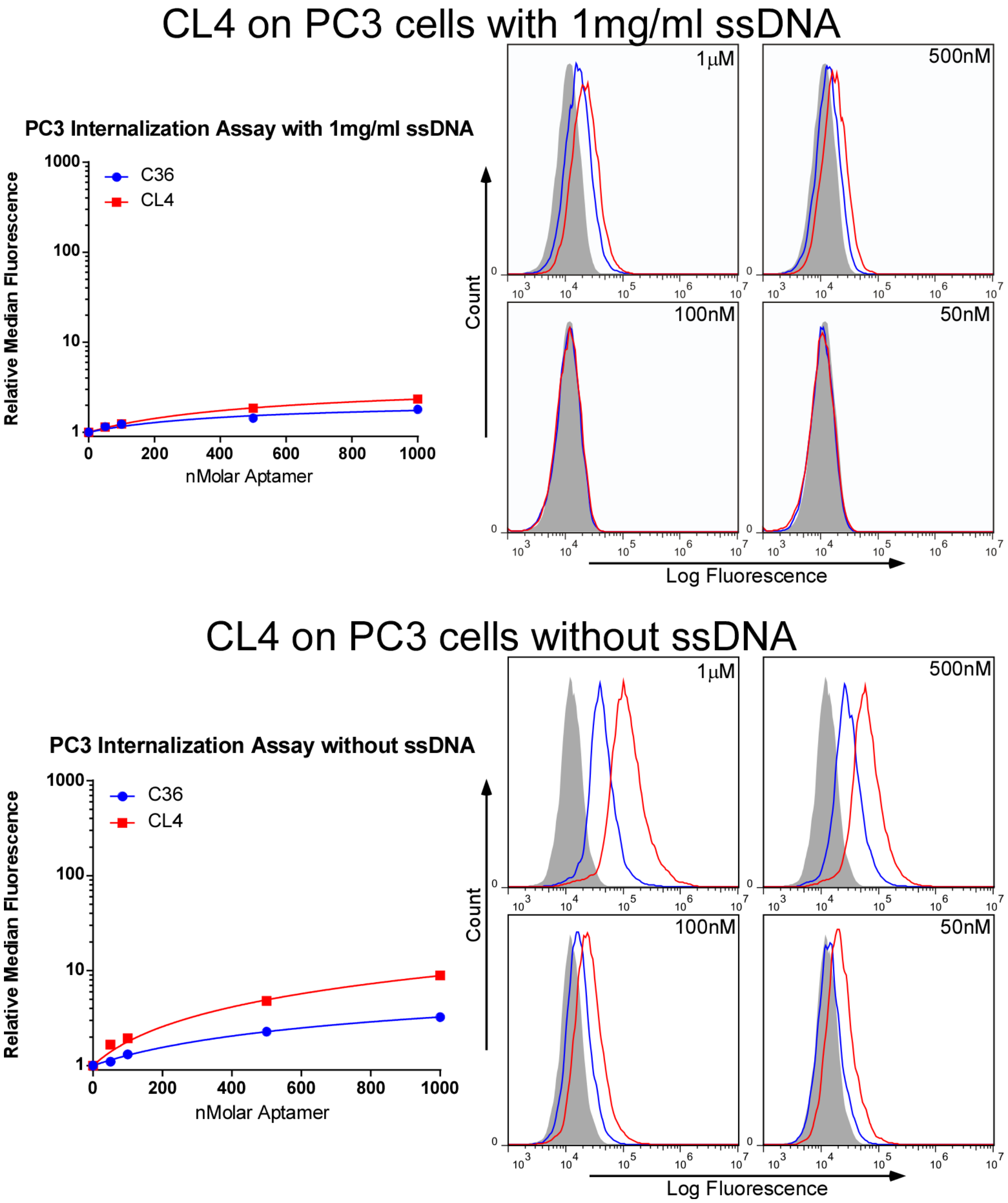

Supplementary Fig. 141. EGFR targeted aptamer CL4 internalization and binding assays on PC3 cells. Graphs represent the median fluorescence of the aptamer (Red) and C36 (Blue) relative to unstained cells (Gray).

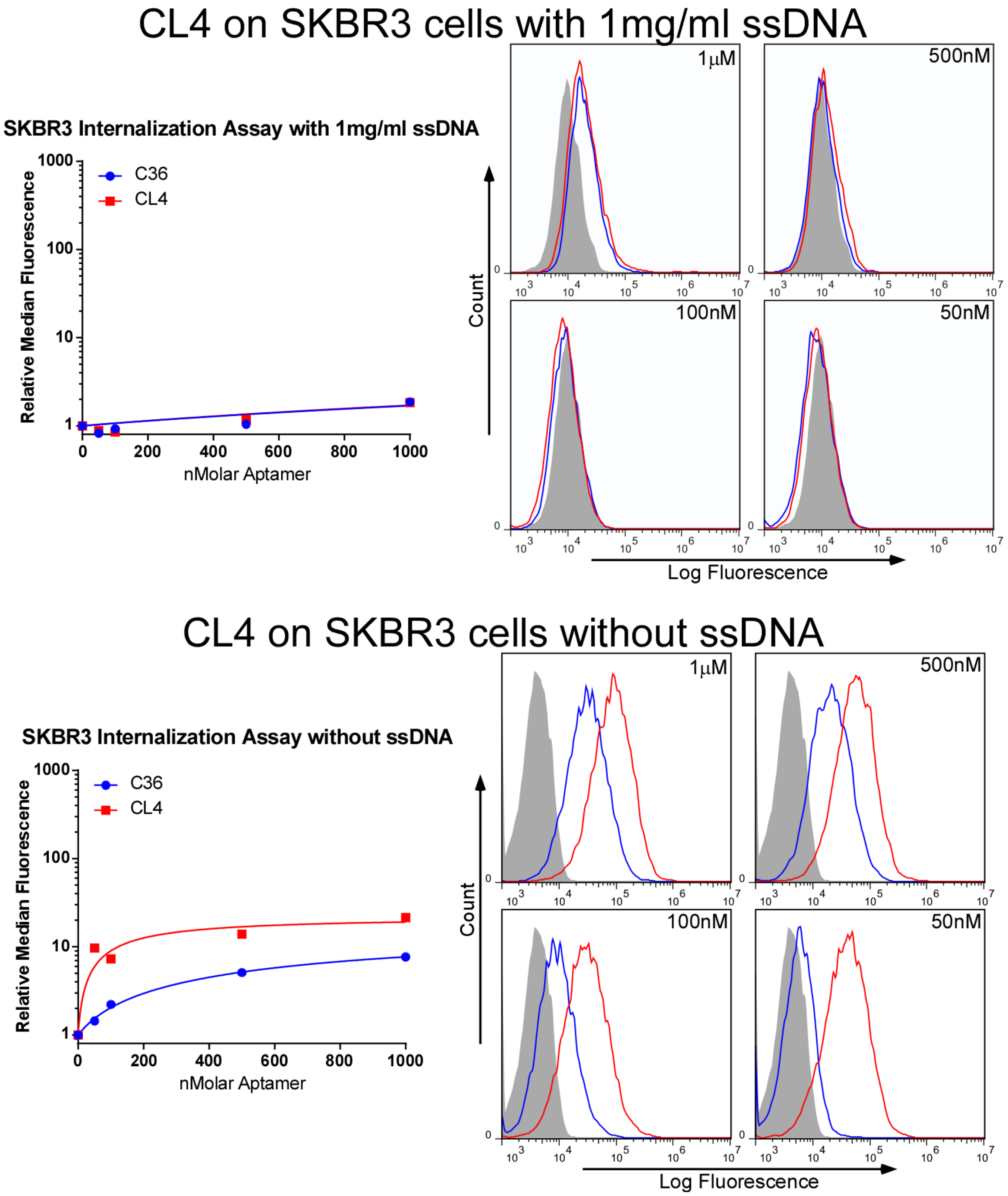

Supplementary Fig. 142. EGFR targeted aptamer CL4 internalization and binding assays on SKBR3 cells. Graphs represent the median fluorescence of the aptamer (Red) and C36 (Blue) relative to unstained cells (Gray).

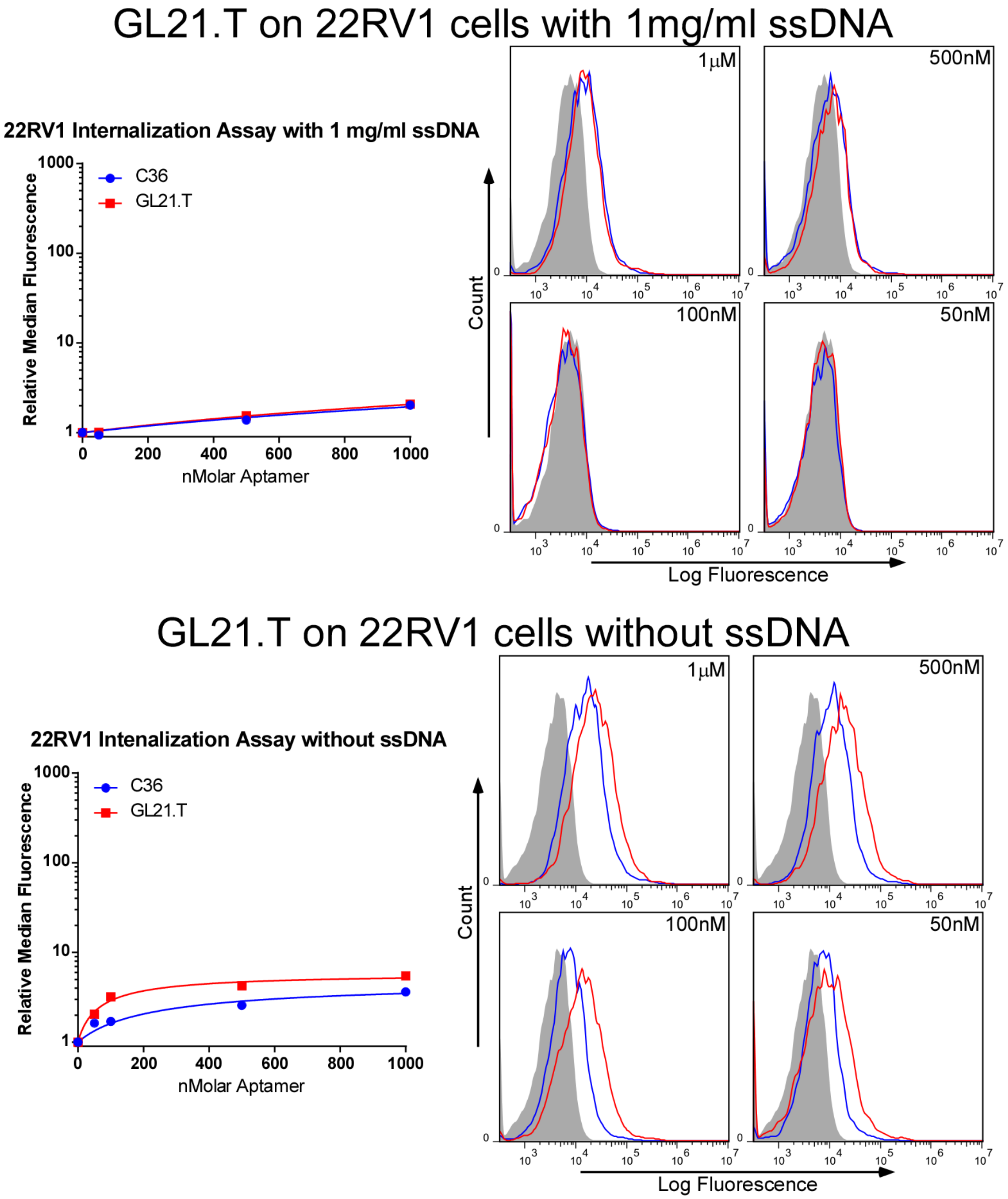

Supplementary Fig. 143. AXL targeted aptamer GL21.T internalization and binding assays on 22RV1 cells. Graphs represent the median fluorescence of the aptamer (Red) and C36 (Blue) relative to unstained cells (Gray).

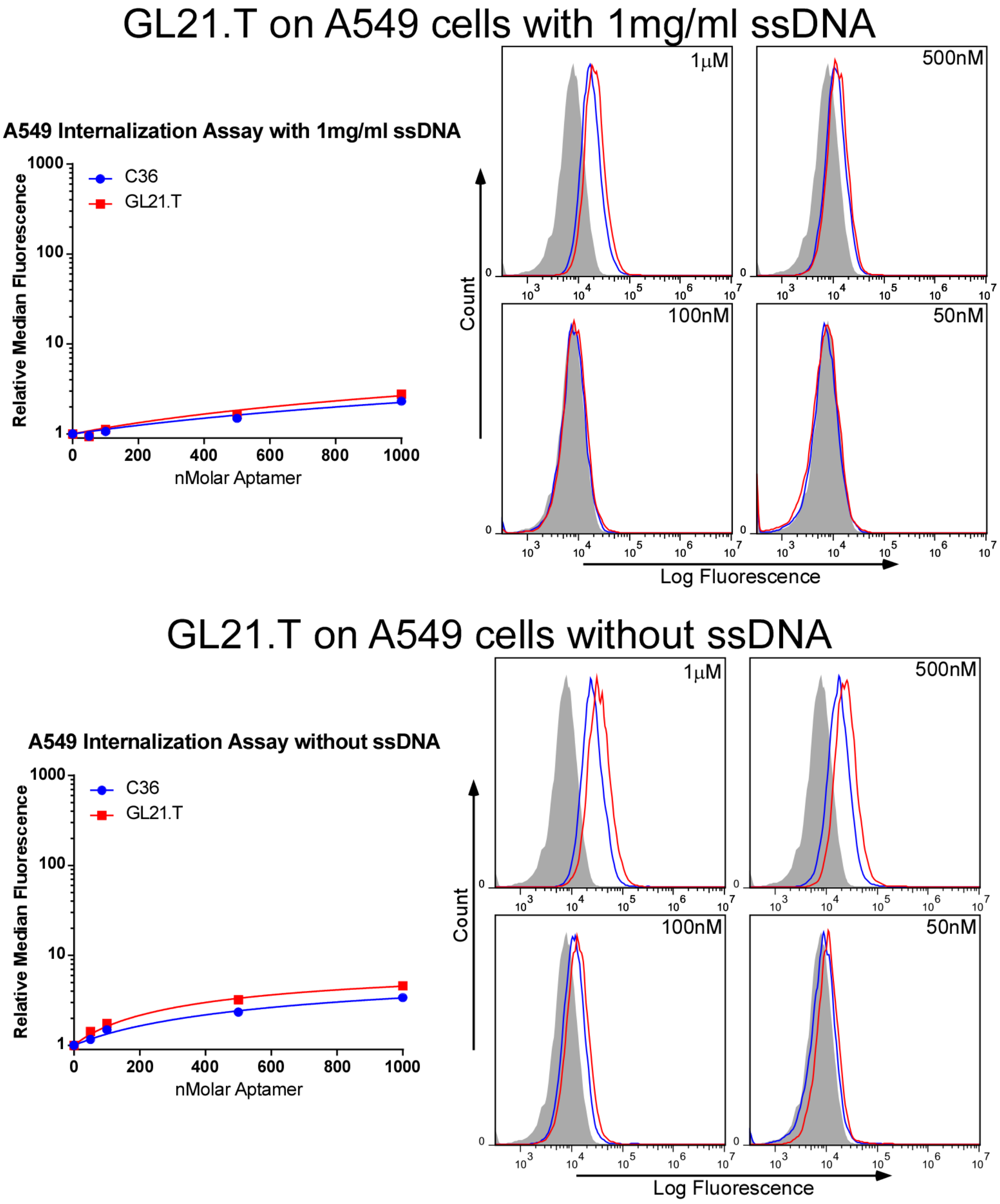

Supplementary Fig. 144. AXL targeted aptamer GL21.T internalization and binding assays on A549 cells. Graphs represent the median fluorescence of the aptamer (Red) and C36 (Blue) relative to unstained cells (Gray).

GL21.T on HeLa PSMA cells with 1mg/ml ssDNA

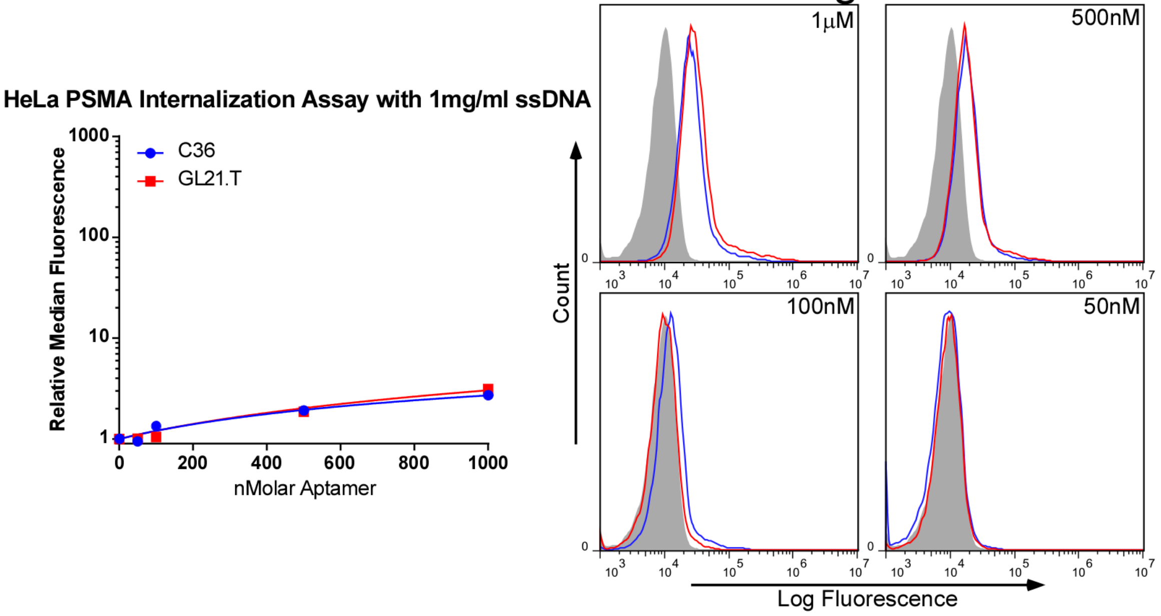

GL21.T on HeLa PSMA cells without ssDNA

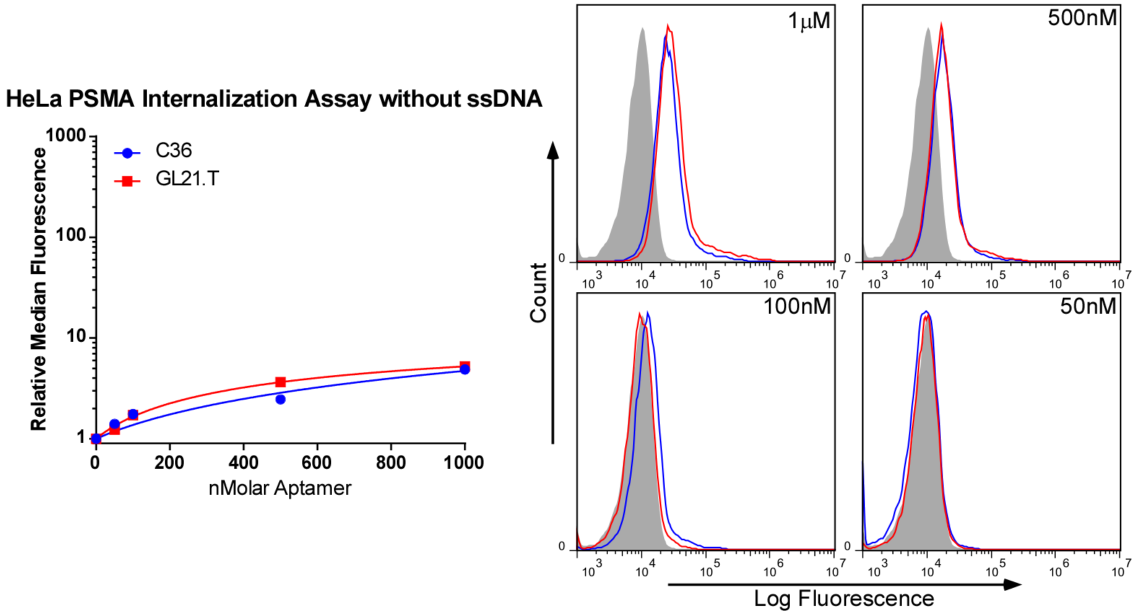

Supplementary Fig. 145. AXL targeted aptamer GL21.T internalization and binding assays on HeLa PSMA cells. Graphs represent the median fluorescence of the aptamer (Red) and C36 (Blue) relative to unstained cells (Gray).

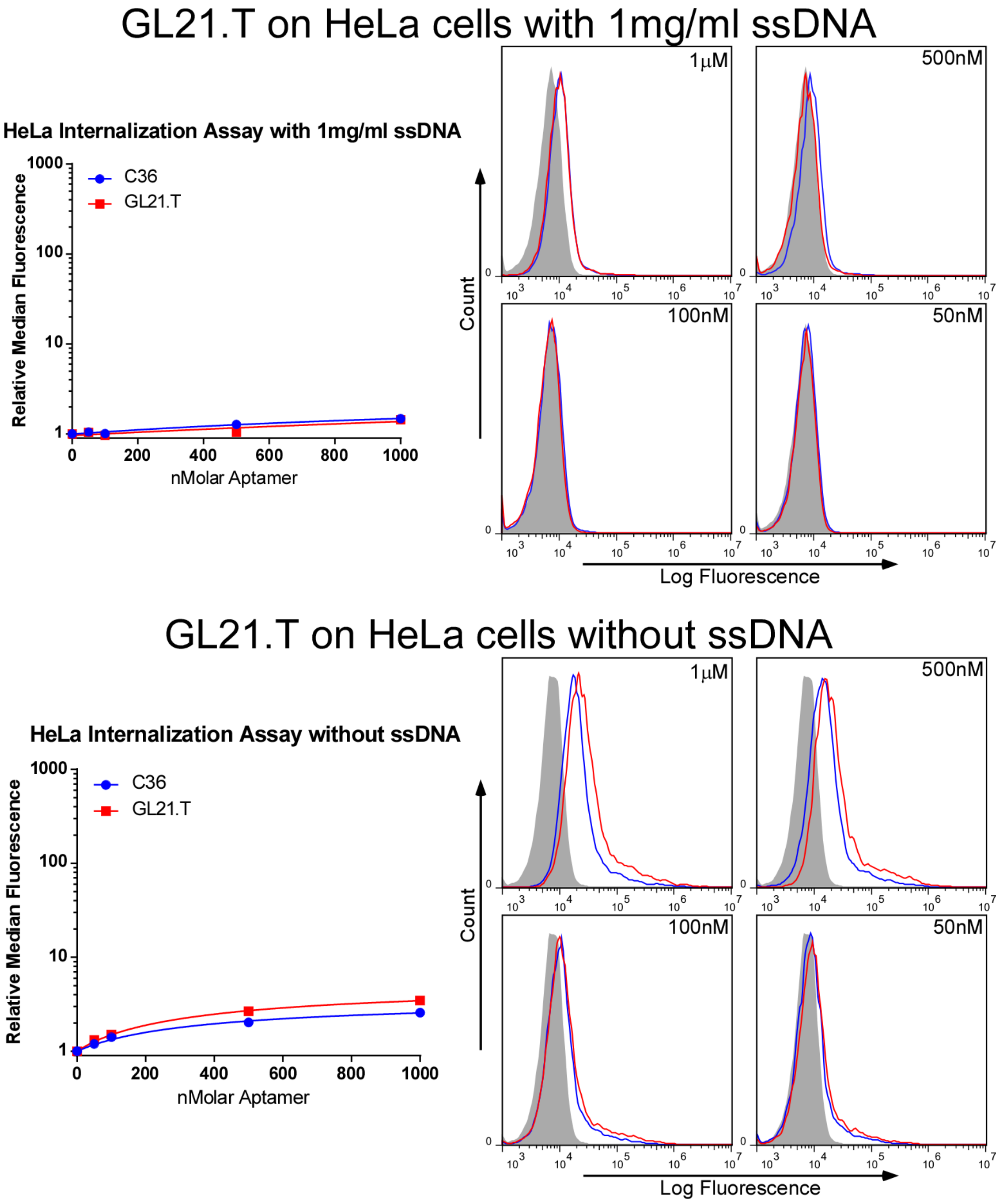

Supplementary Fig. 146. AXL targeted aptamer GL21.T internalization and binding assays on HeLa cells. Graphs represent the median fluorescence of the aptamer (Red) and C36 (Blue) relative to unstained cells (Gray).

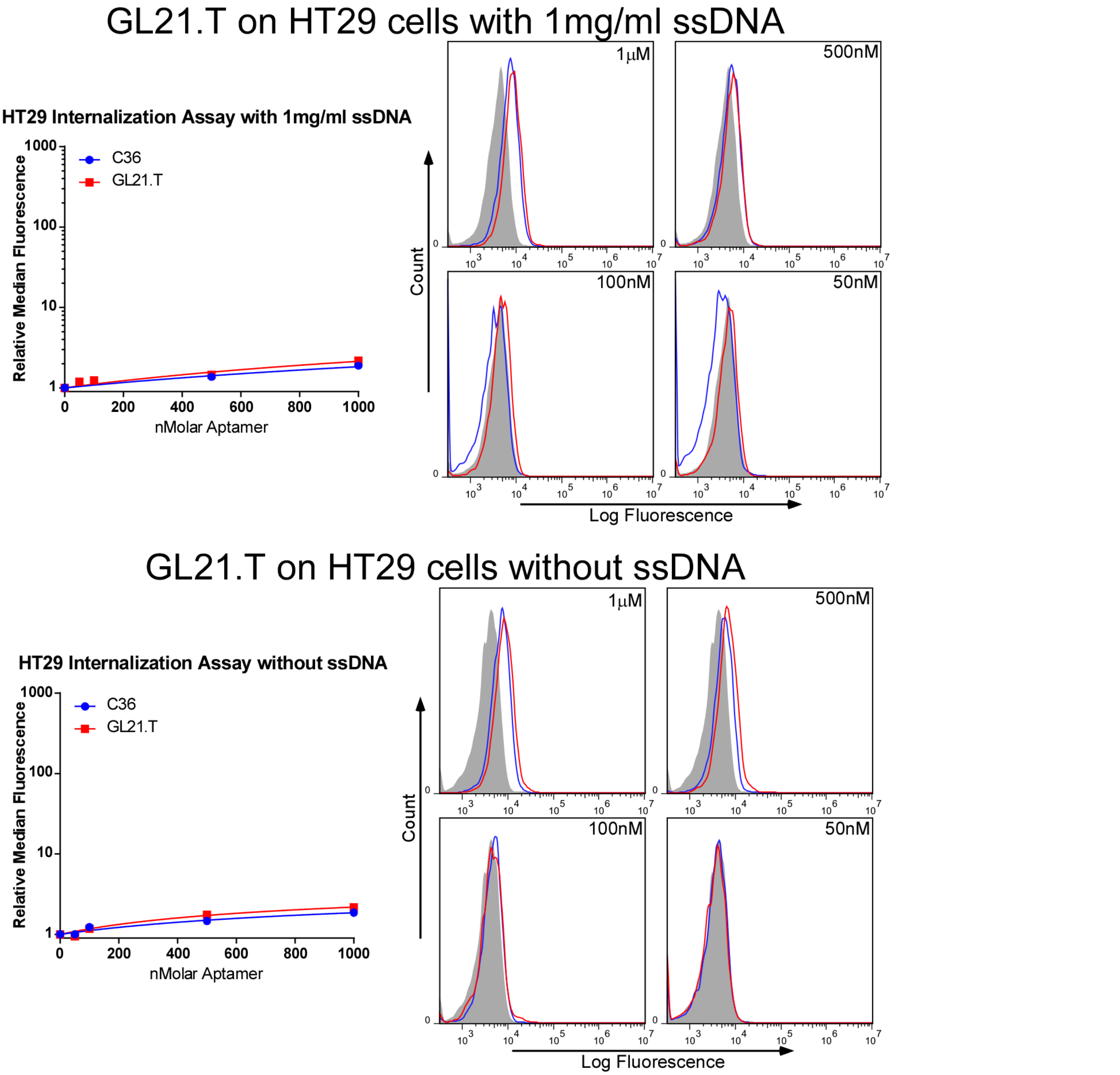

Supplementary Fig. 147. AXL targeted aptamer GL21.T internalization and binding assays on HT29 cells. Graphs represent the median fluorescence of the aptamer (Red) and C36 (Blue) relative to unstained cells (Gray).

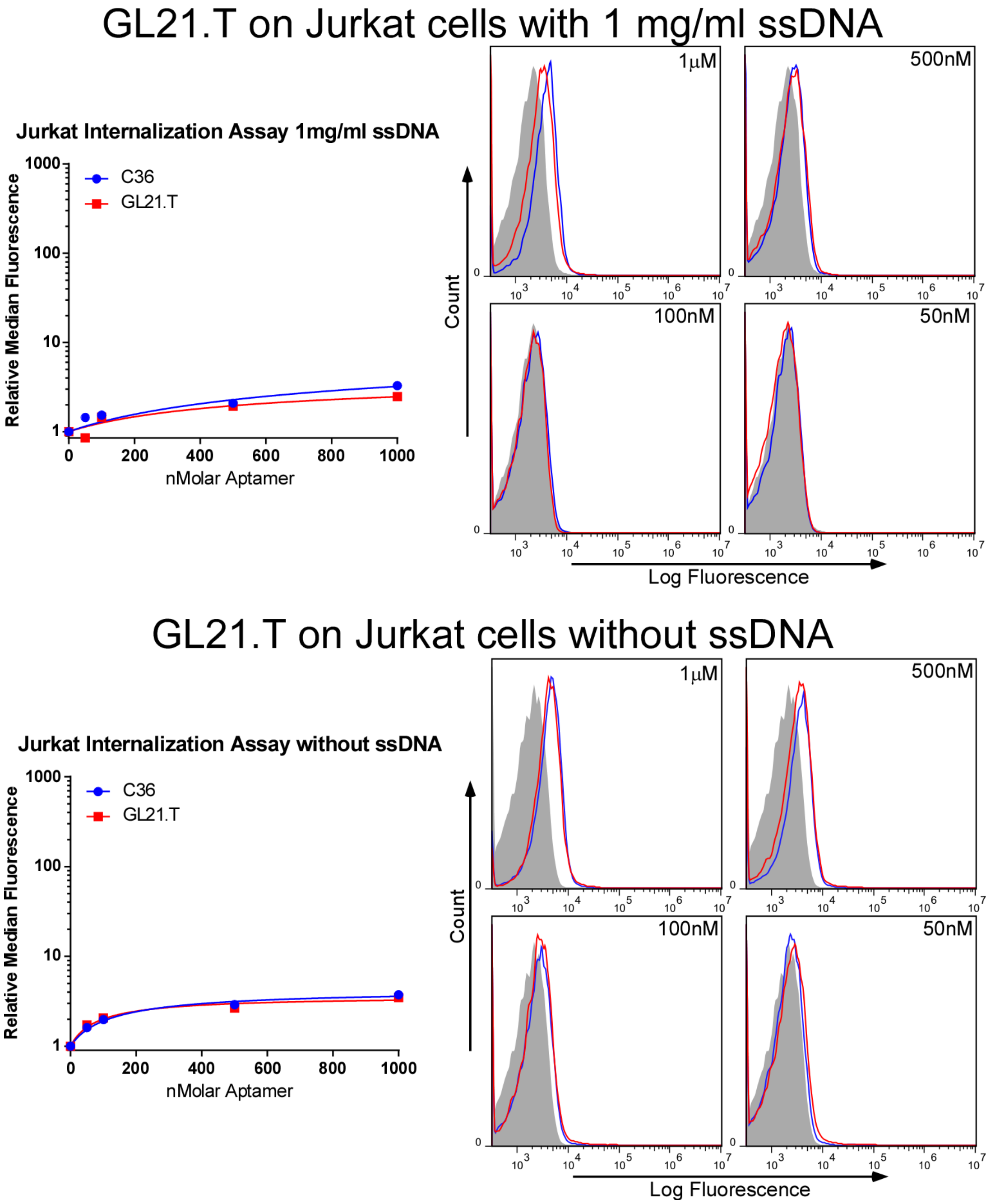

Supplementary Fig. 148. AXL targeted aptamer GL21.T internalization and binding assays on Jurkat cells. Graphs represent the median fluorescence of the aptamer (Red) and C36 (Blue) relative to unstained cells (Gray).

GL21.T on LNCaP cells with 1mg/ml ssDNA

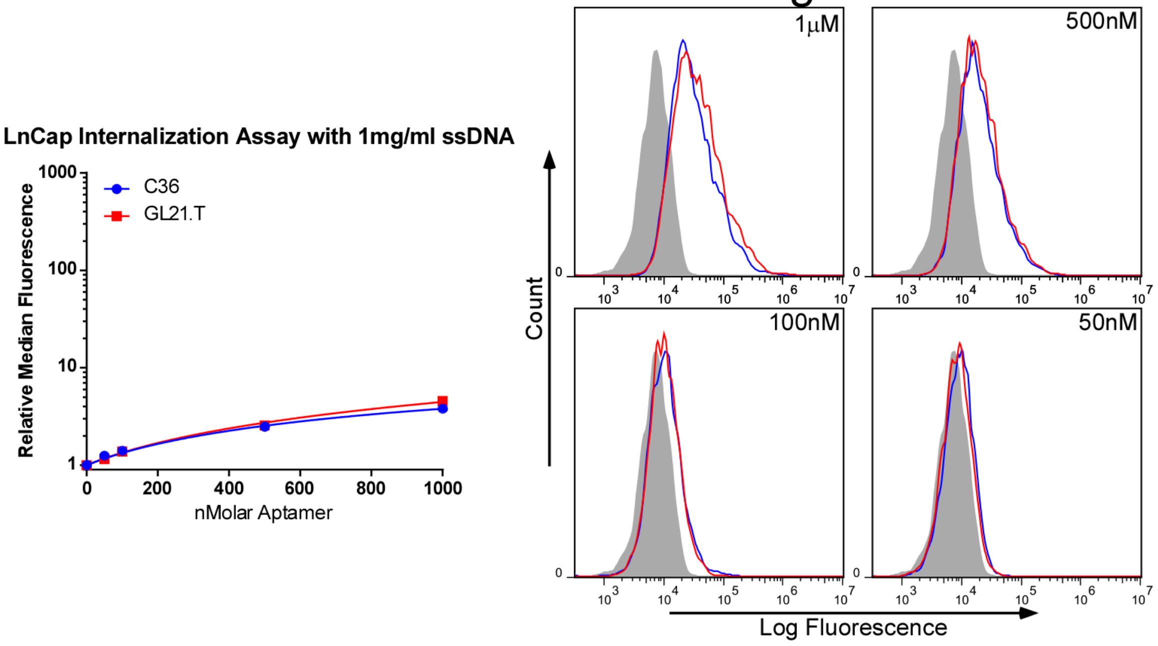

GL21.T on LNCaP cells without ssDNA

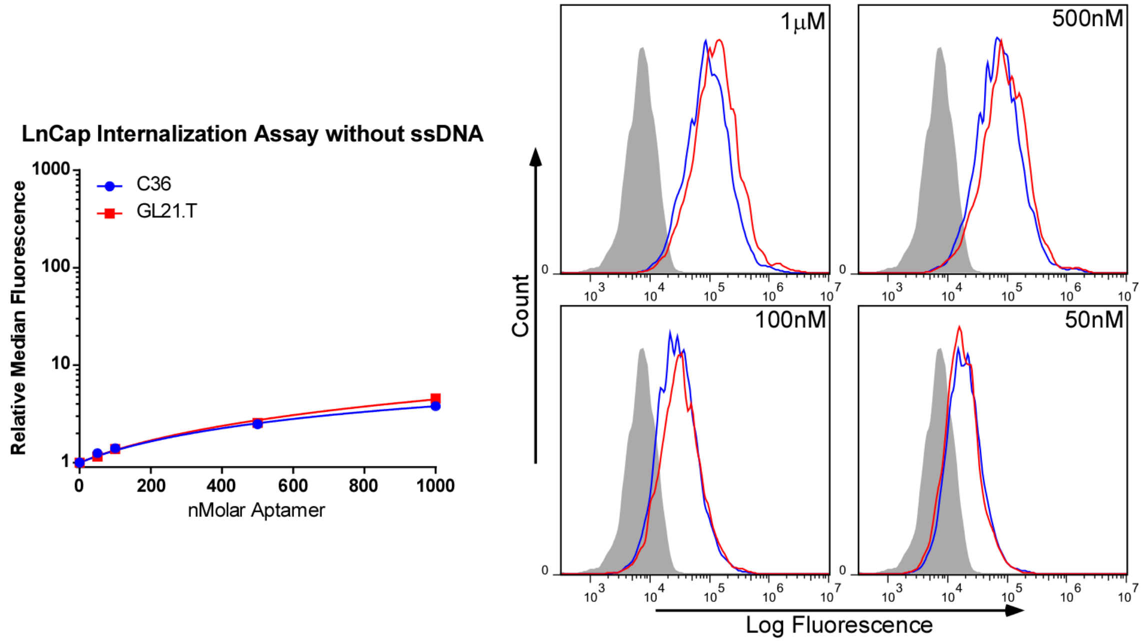

Supplementary Fig. 149. AXL targeted aptamer GL21.T internalization and binding assays on LNCaP cells. Graphs represent the median fluorescence of the aptamer (Red) and C36 (Blue) relative to unstained cells (Gray).

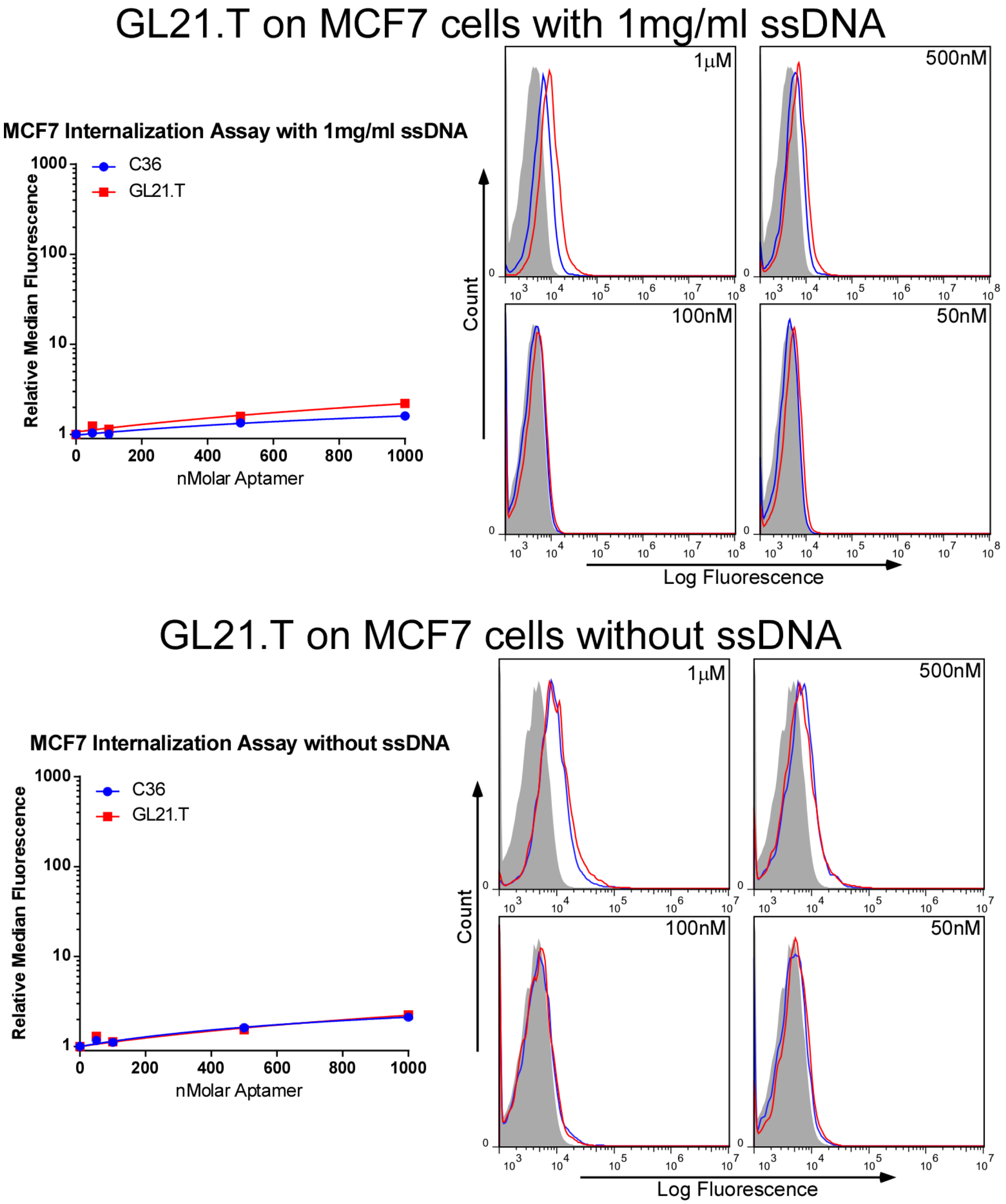

Fig. 150. AXL targeted aptamer GL21.T internalization and binding assays on MCF7 cells. Graphs represent the median fluorescence of the aptamer (Red) and C36 (Blue) relative to unstained cells (Gray).

GL21.T on PC3 PSMA cells with 1mg/ml ssDNA

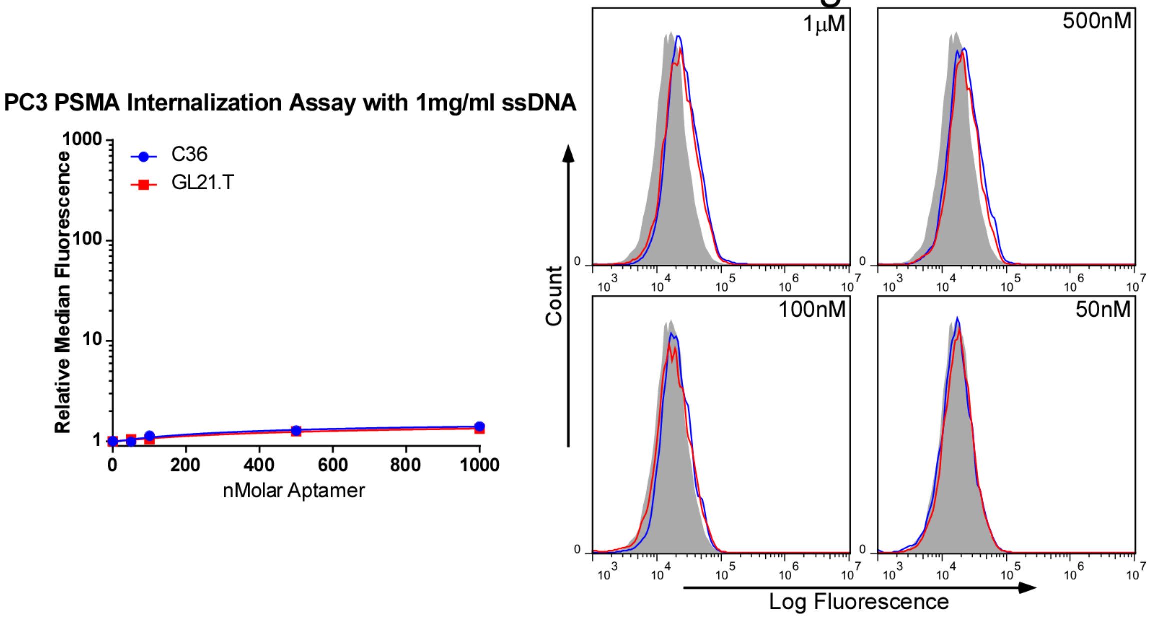

GL21.T on PC3 PSMA cells without ssDNA

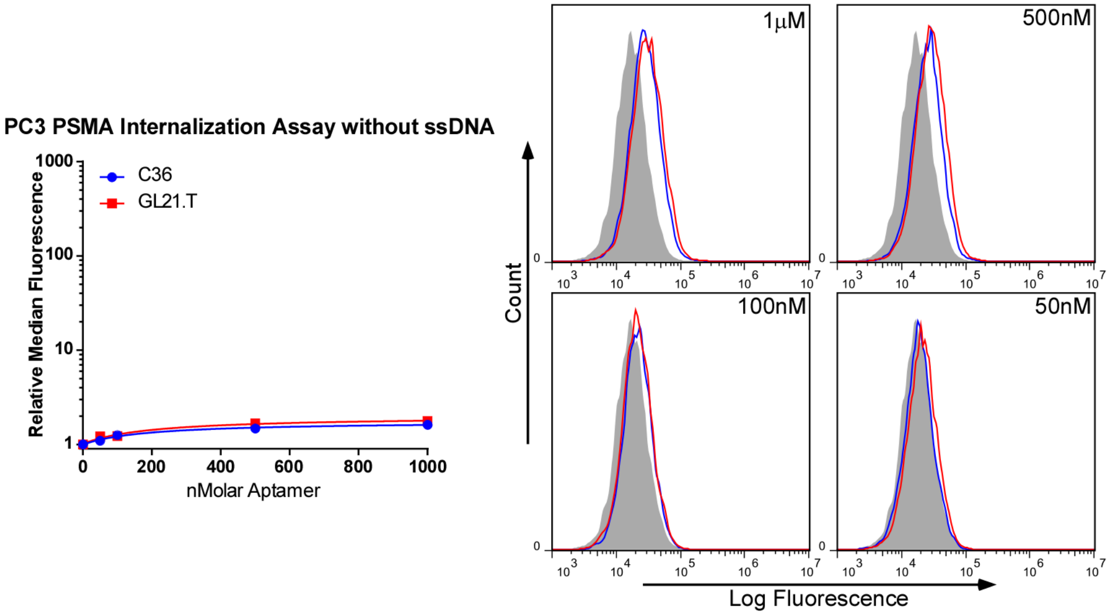

Supplementary Fig. 151. AXL targeted aptamer GL21.T internalization and binding assays on PC3 PSMA cells. Graphs represent the median fluorescence of the aptamer (Red) and C36 (Blue) relative to unstained cells (Gray).

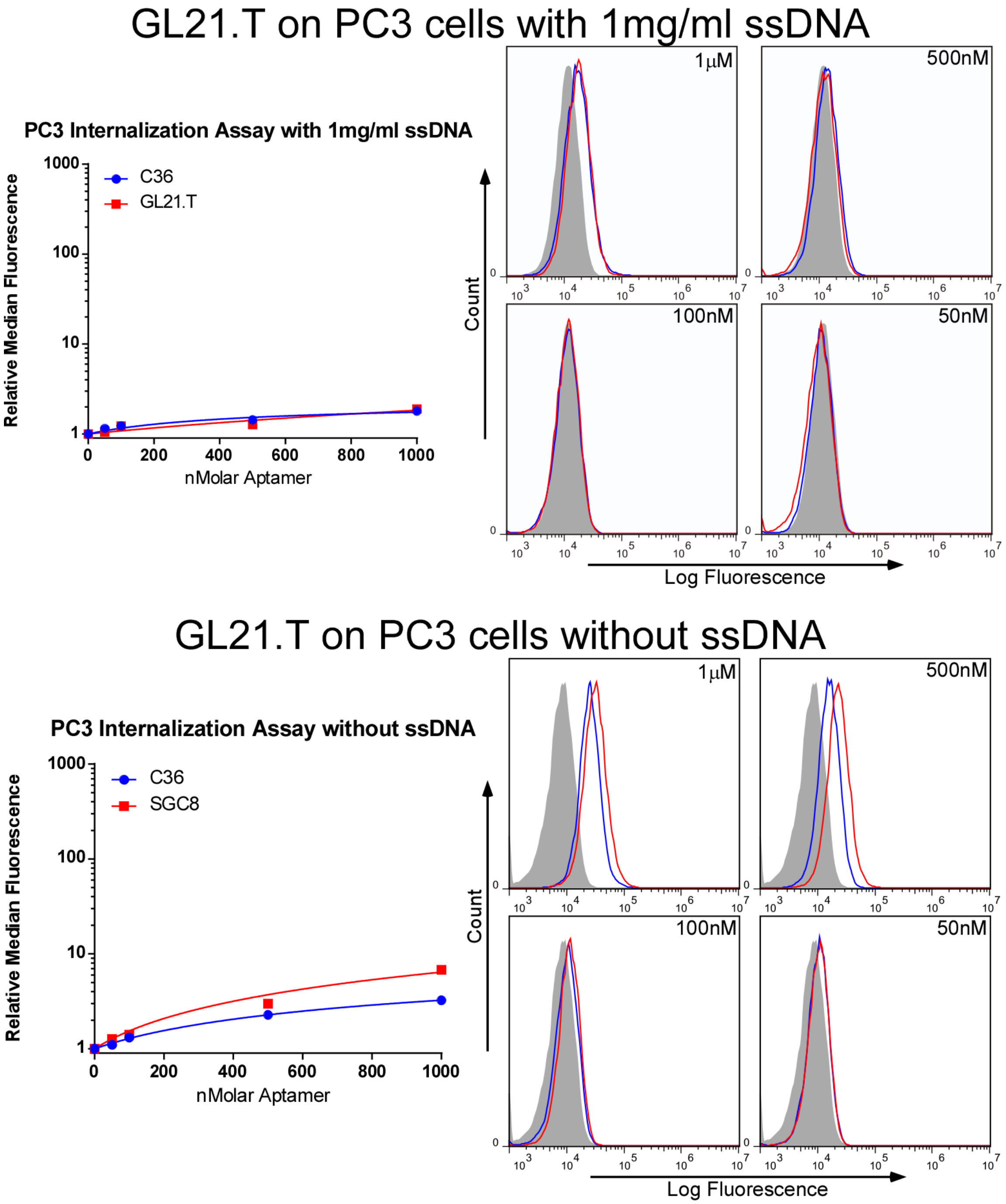

Supplementary Fig. 152. AXL targeted aptamer GL21.T internalization and binding assays on PC3 cells. Graphs represent the median fluorescence of the aptamer (Red) and C36 (Blue) relative to unstained cells (Gray).

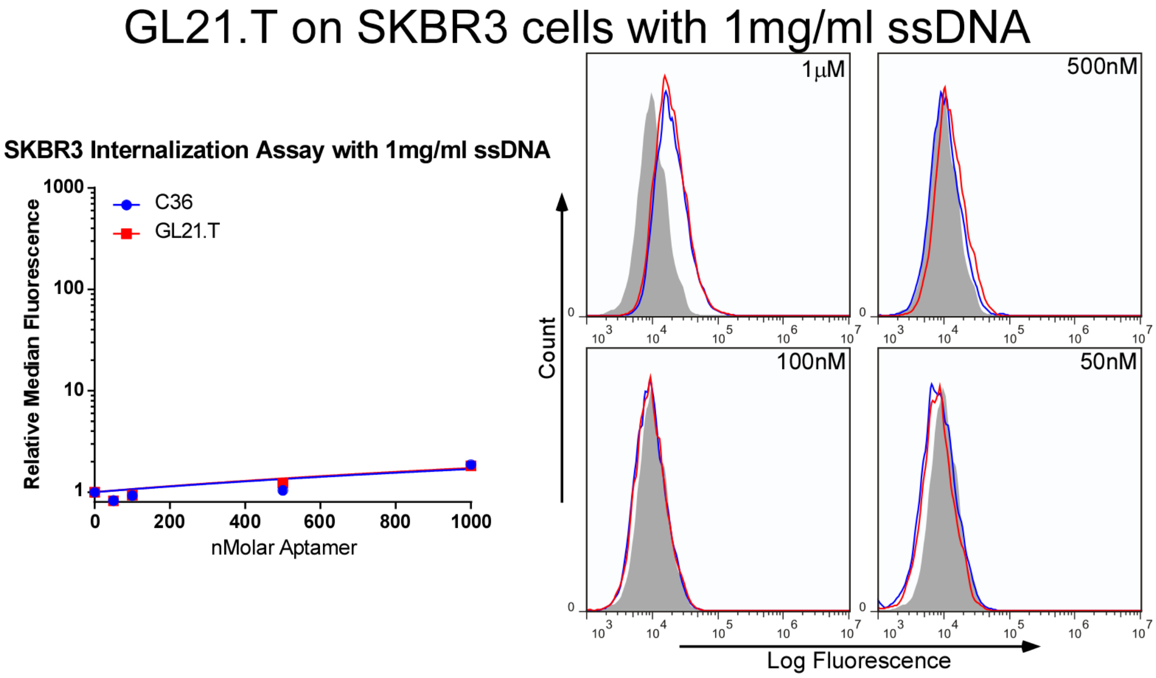

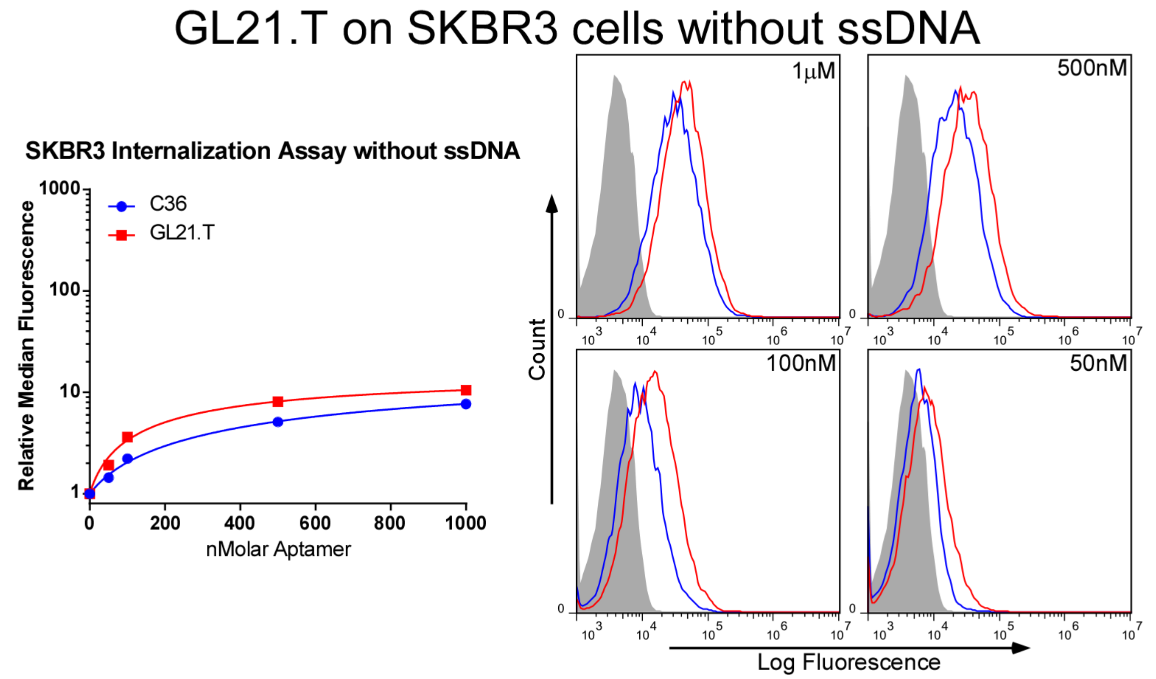

Supplementary Fig. 153. AXL targeted aptamer GL21.T internalization and binding assays on SKBR3 cells. Graphs represent the median fluorescence of the aptamer (Red) and C36 (Blue) relative to unstained cells (Gray).

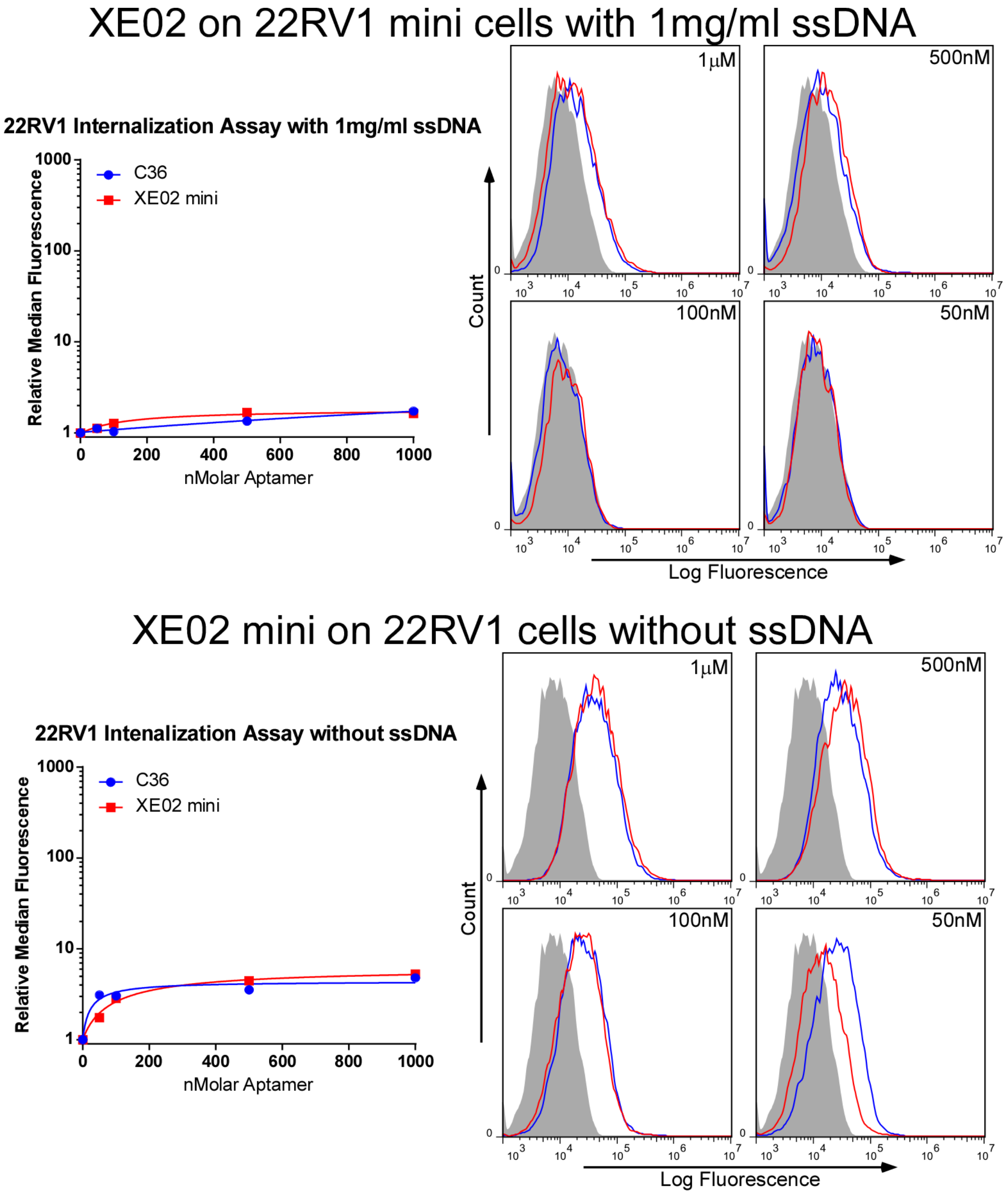

Supplementary Fig. 154. Aptamer XE02.mini internalization and binding assays on 22RV1 cells. Graphs represent the median fluorescence of the aptamer (Red) and C36 (Blue) relative to unstained cells (Gray).

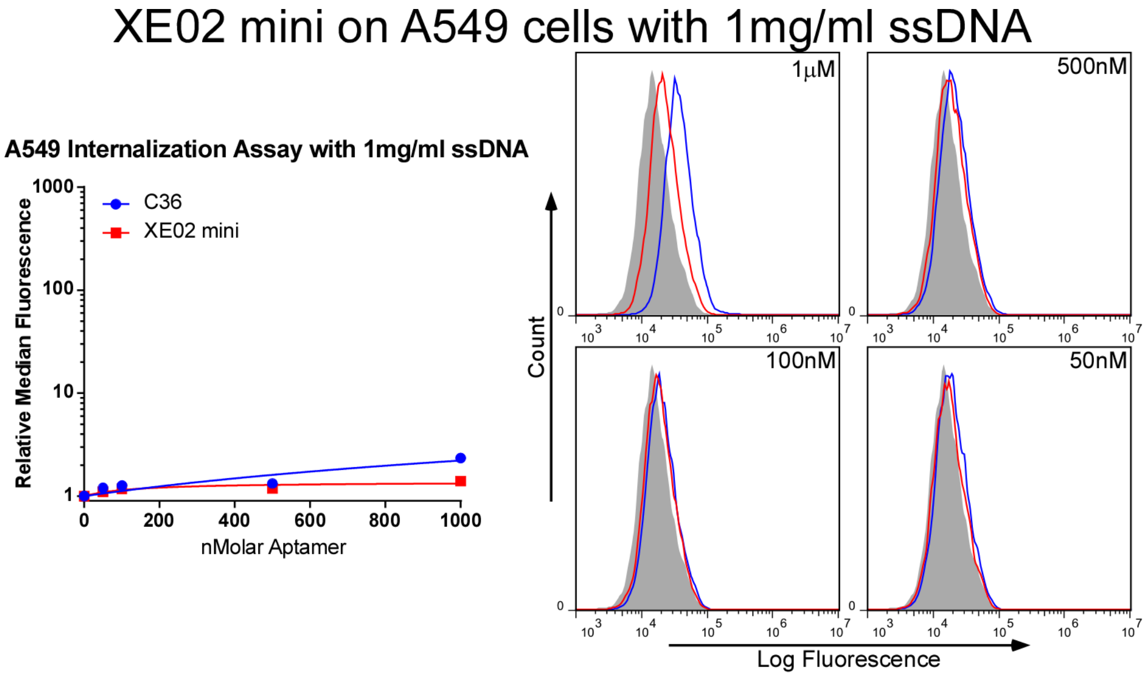

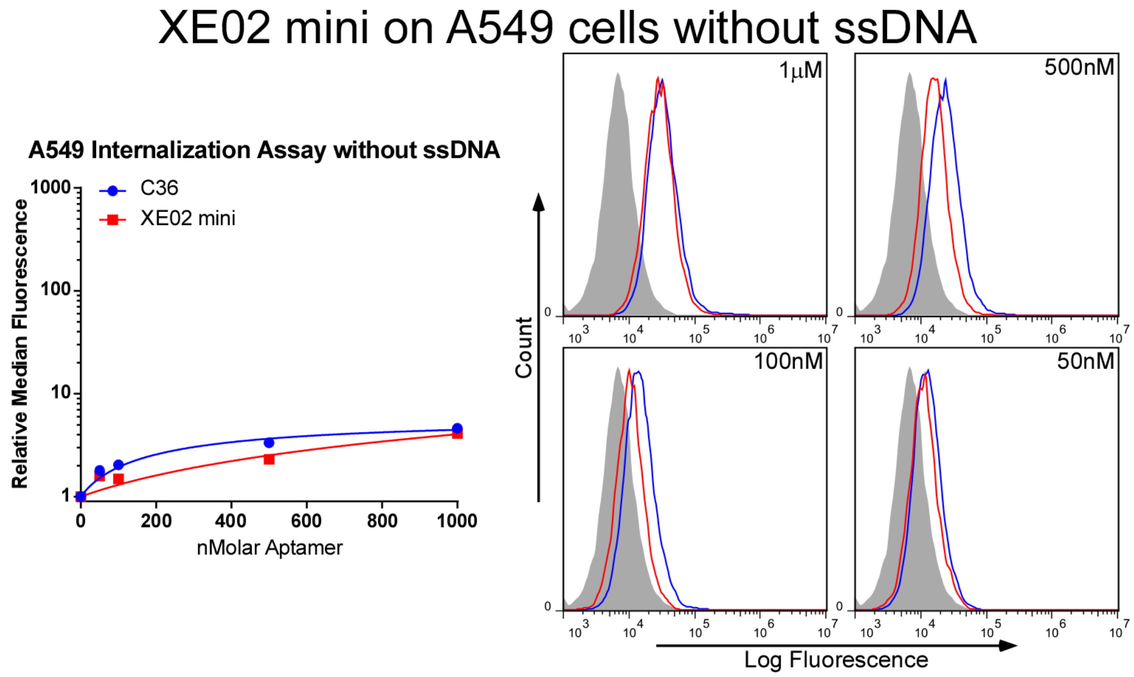

Supplementary Fig. 155. Aptamer XE02.mini internalization and binding assays on A549 cells. Graphs represent the median fluorescence of the aptamer (Red) and C36 (Blue) relative to unstained cells (Gray).

XE02 mini on HeLa PSMA cells with 1mg/ml ssDNA

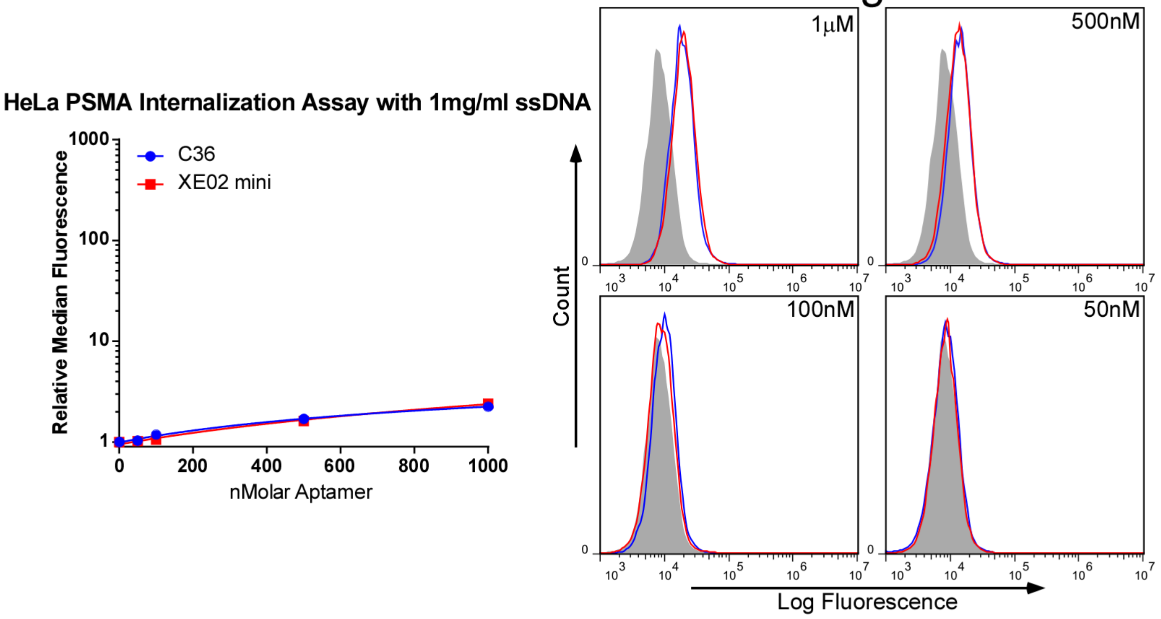

XE02 mini on HeLa PSMA cells without ssDNA

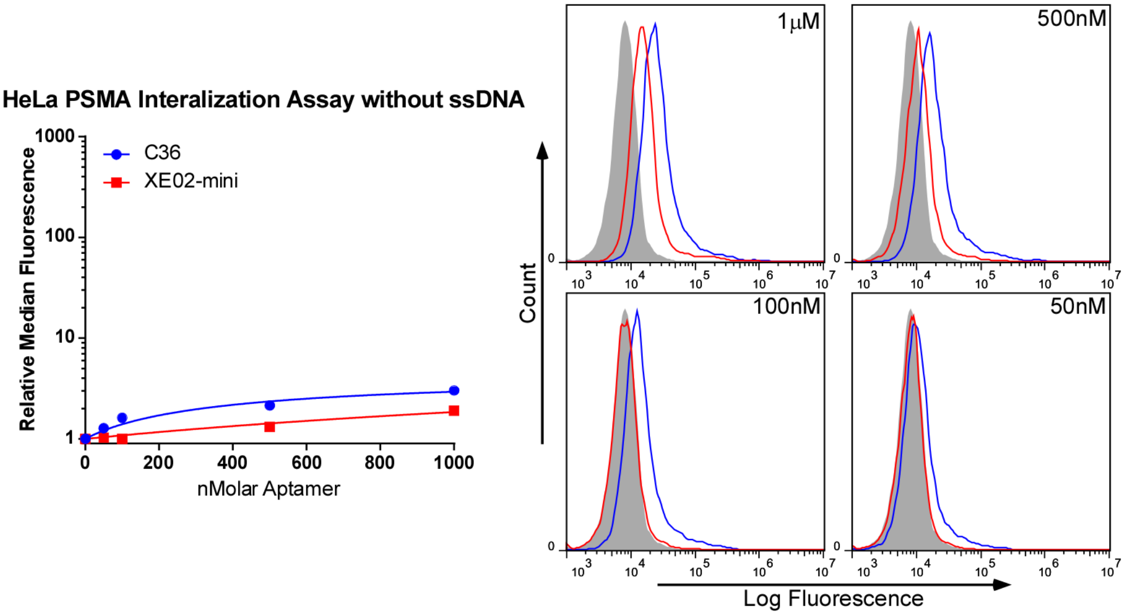

Supplementary Fig. 156. Aptamer XE02.mini internalization and binding assays on HeLa PSMA cells. Graphs represent the median fluorescence of the aptamer (Red) and C36 (Blue) relative to unstained cells (Gray).

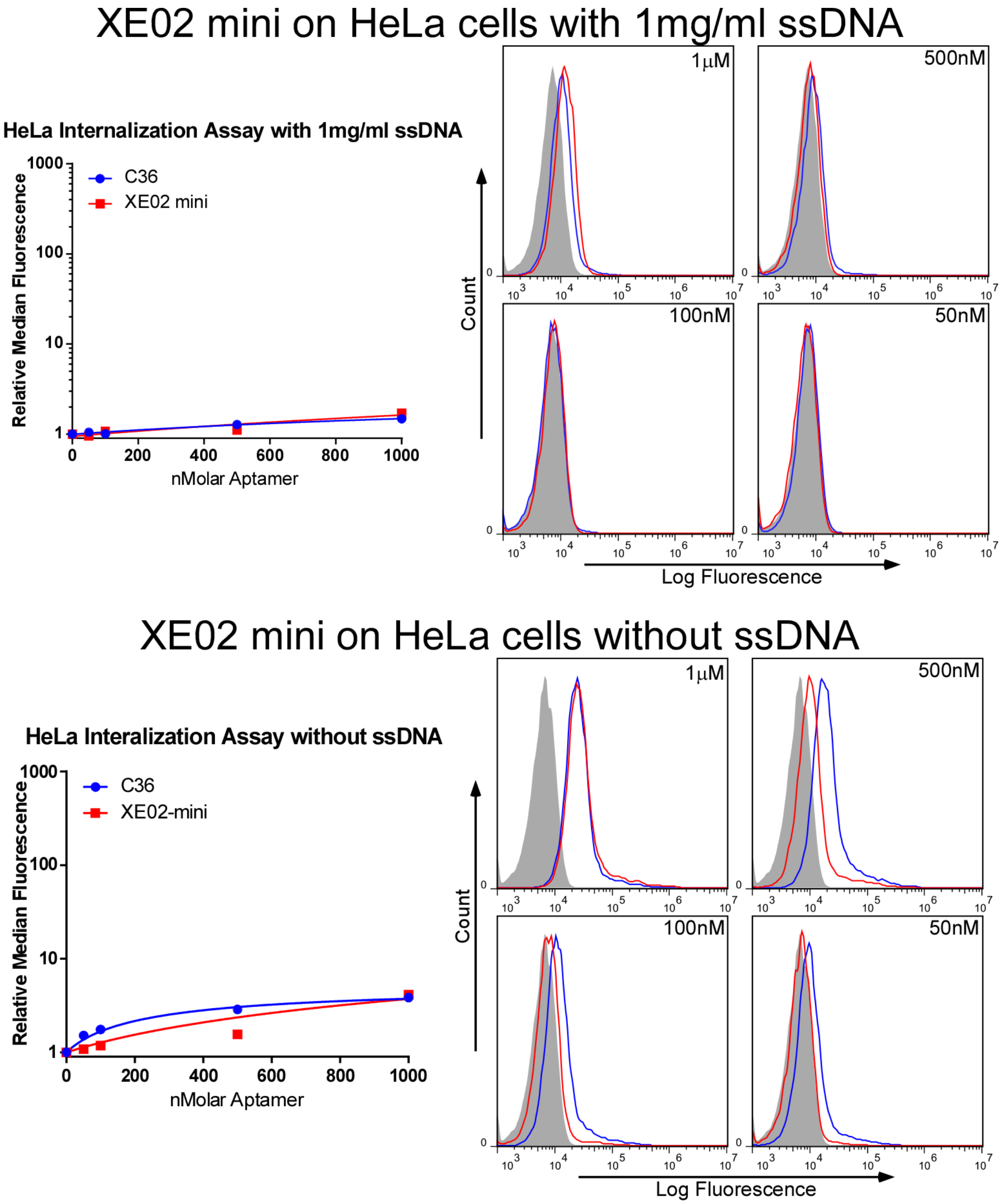

Supplementary Fig. 157. Aptamer XE02.mini internalization and binding assays on HeLa cells. Graphs represent the median fluorescence of the aptamer (Red) and C36 (Blue) relative to unstained cells (Gray).

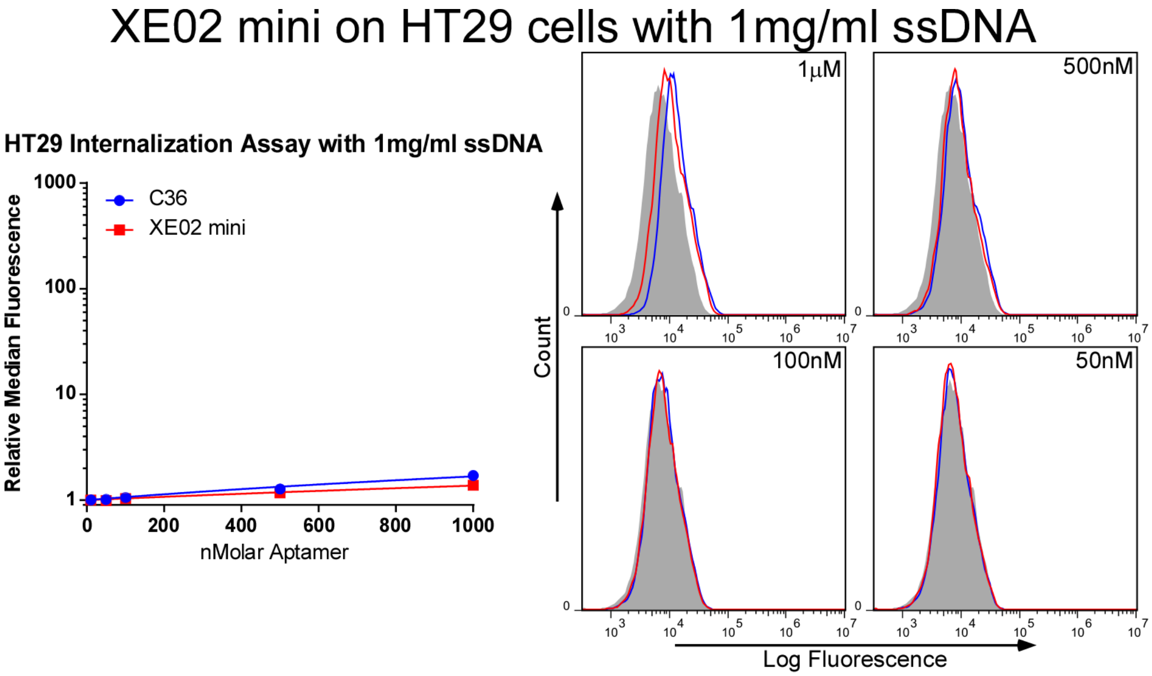

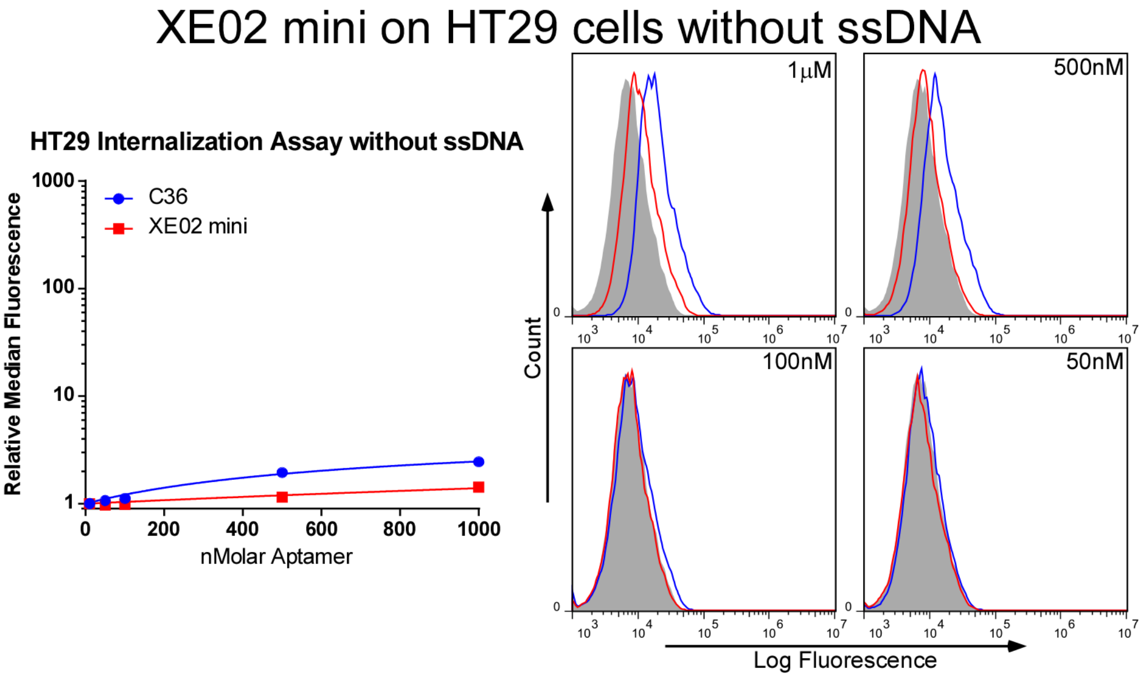

Supplementary Fig. 158. Aptamer XE02.mini internalization and binding assays on HT29 cells. Graphs represent the median fluorescence of the aptamer (Red) and C36 (Blue) relative to unstained cells (Gray).

XE02 mini on Jurkat cells with 1 mg/ml ssDNA

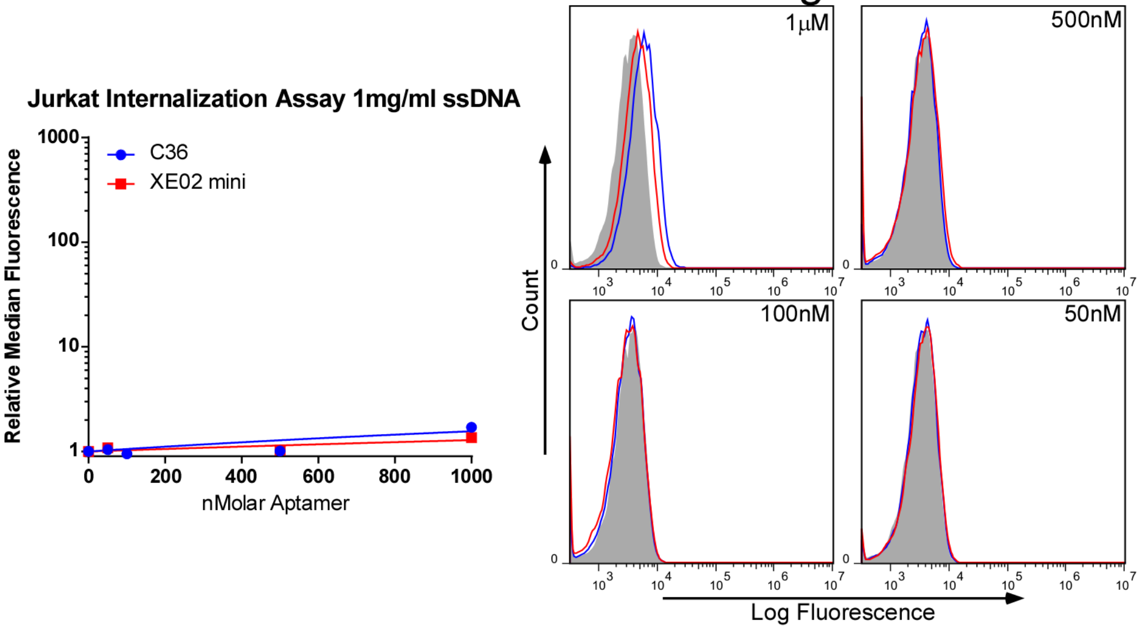

XE02 mini on Jurkat cells without ssDNA

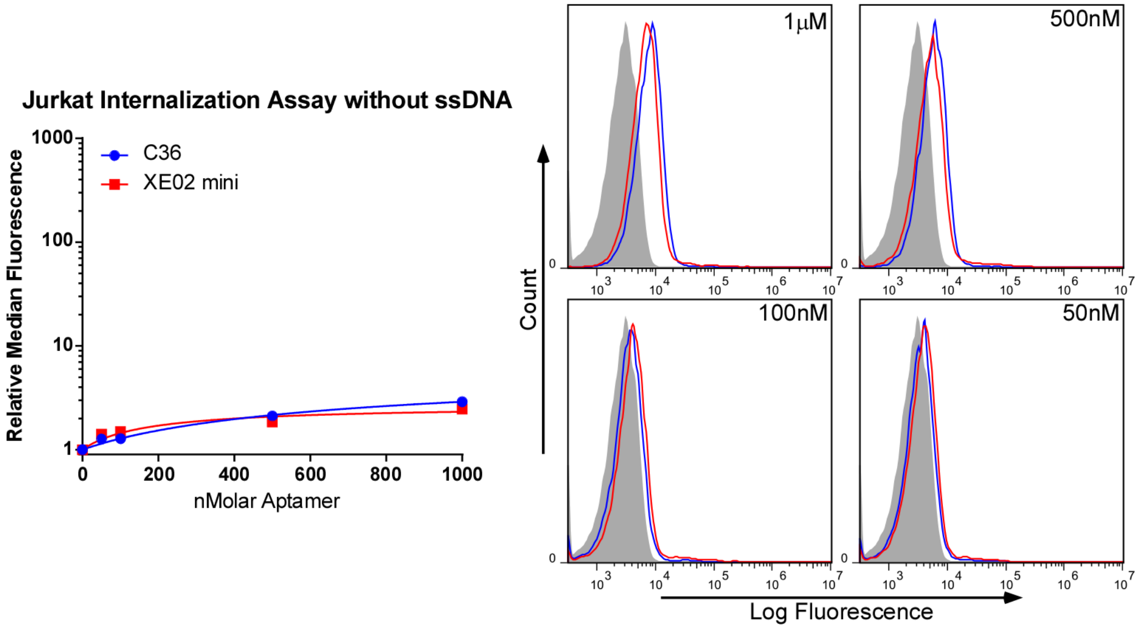

Supplementary Fig. 159. Aptamer XE02.mini internalization and binding assays on Jurkat cells. Graphs represent the median fluorescence of the aptamer (Red) and C36 (Blue) relative to unstained cells (Gray).

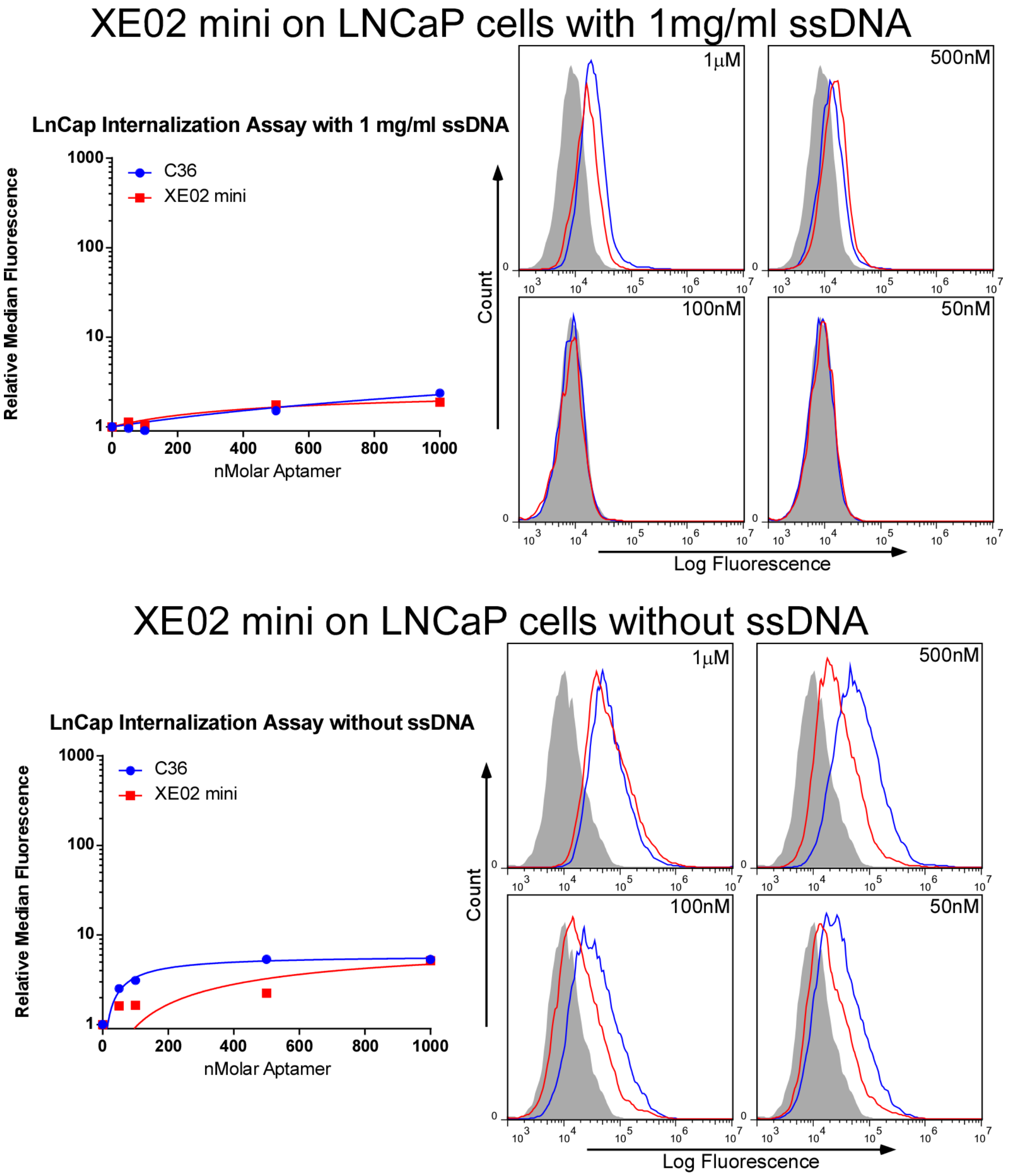

Supplementary Fig. 160. Aptamer XE02.mini internalization and binding assays on LNCaP cells. Graphs represent the median fluorescence of the aptamer (Red) and C36 (Blue) relative to unstained cells (Gray).

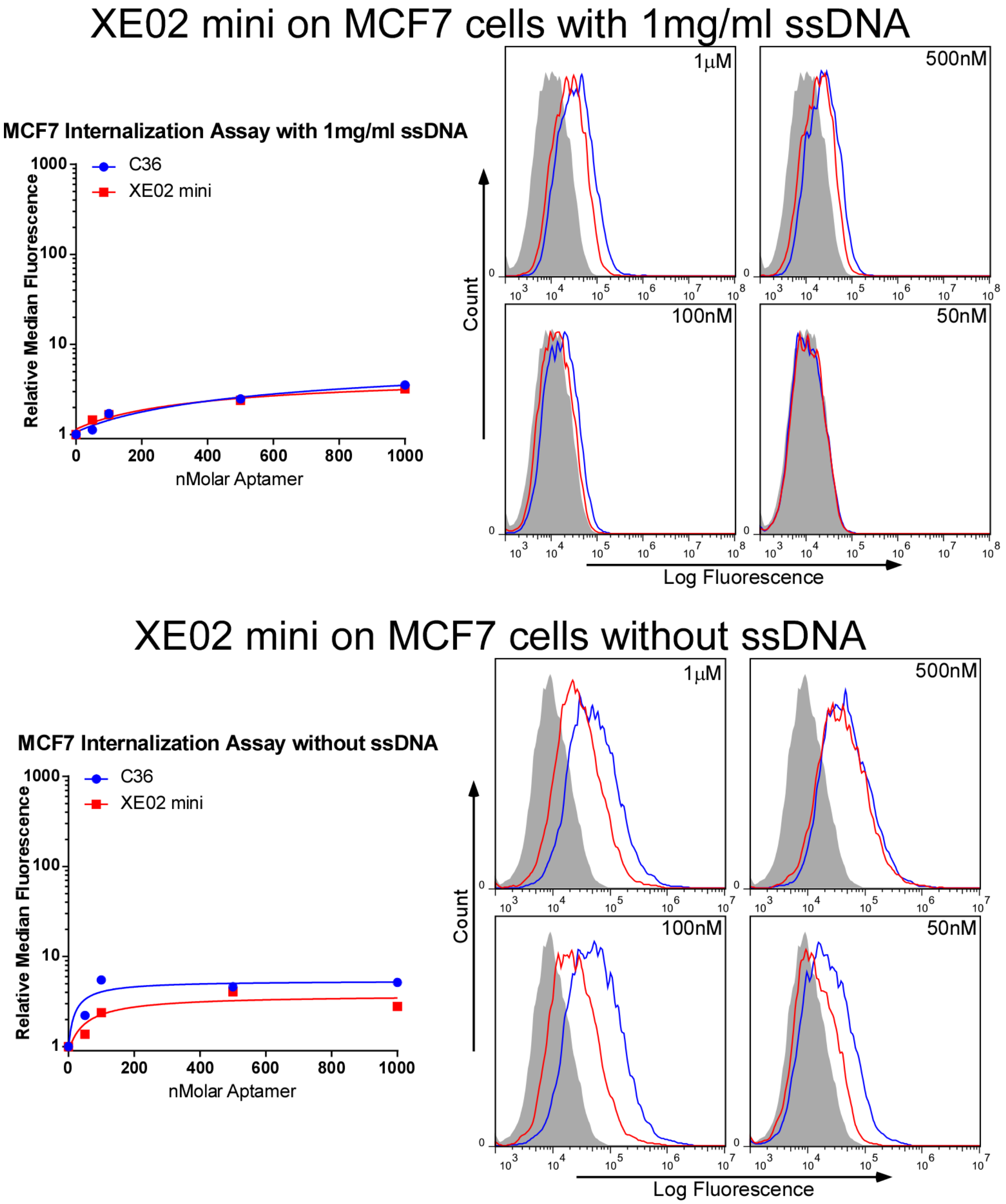

Supplementary Fig. 161. Aptamer XE02.mini internalization and binding assays on MCF7 cells. Graphs represent the median fluorescence of the aptamer (Red) and C36 (Blue) relative to unstained cells (Gray).

XE02 mini on PC3 PSMA cells with 1mg/ml ssDNA

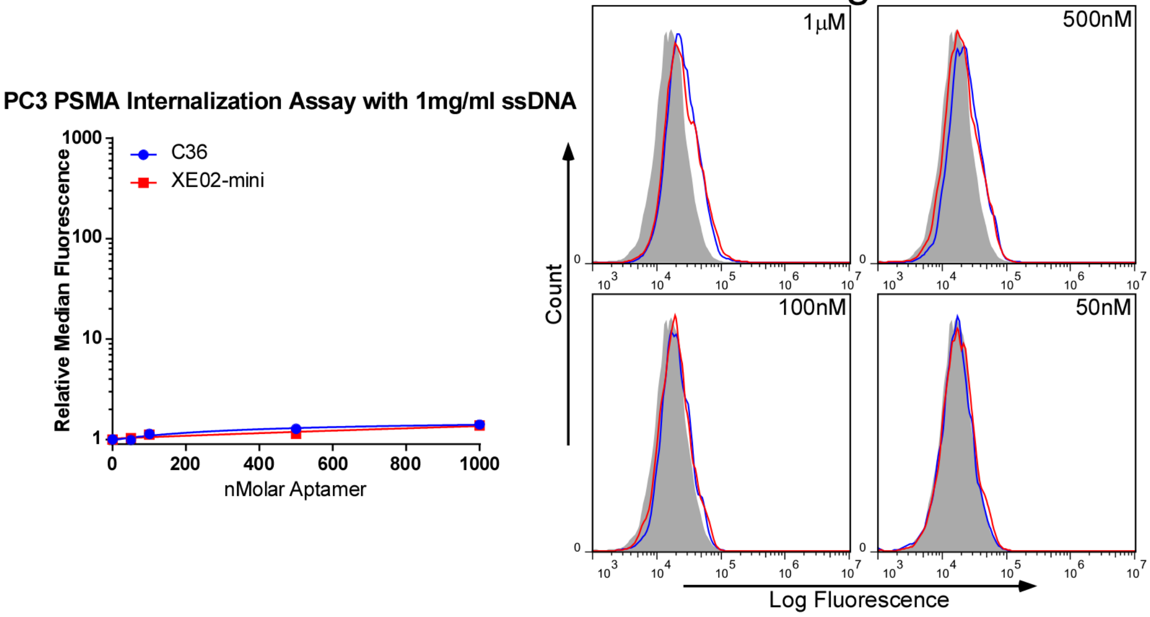

XE02 mini on PC3 PSMA cells without ssDNA

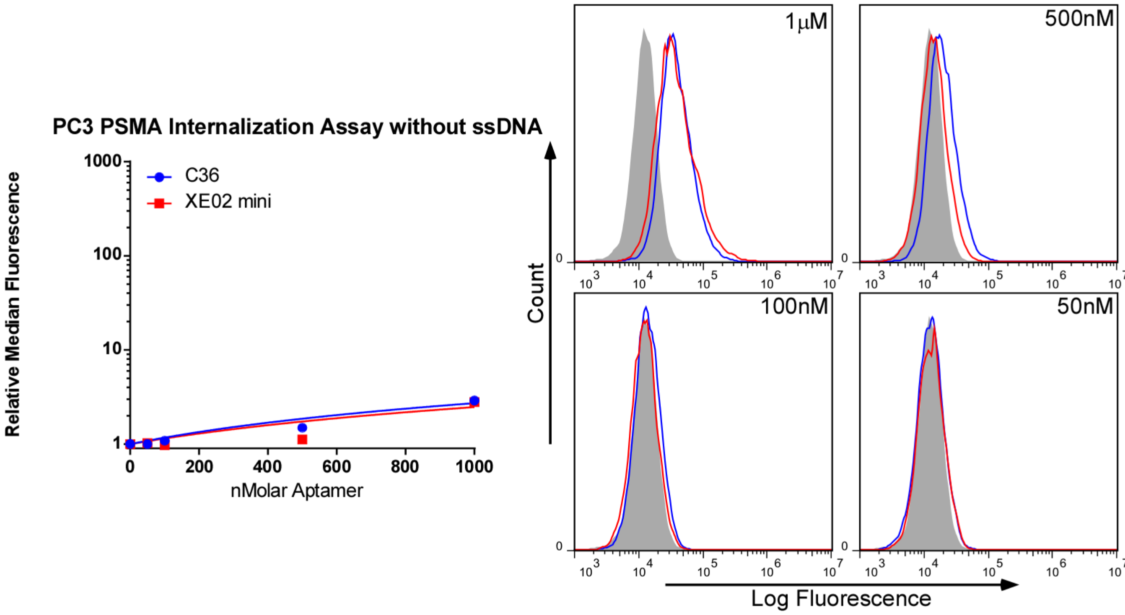

Supplementary Fig. 162. Aptamer XE02.mini internalization and binding assays on PC3 PSMA cells. Graphs represent the median fluorescence of the aptamer (Red) and C36 (Blue) relative to unstained cells (Gray).

XE02 mini on PC3 cells with 1mg/ml ssDNA

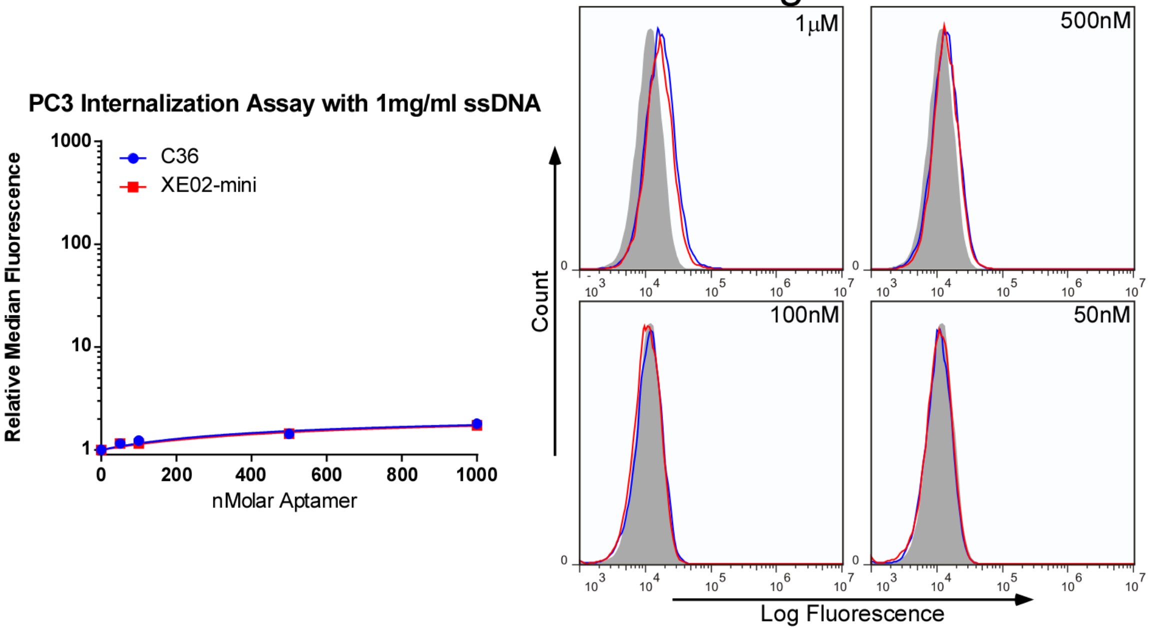

XE02 mini on PC3 cells without ssDNA

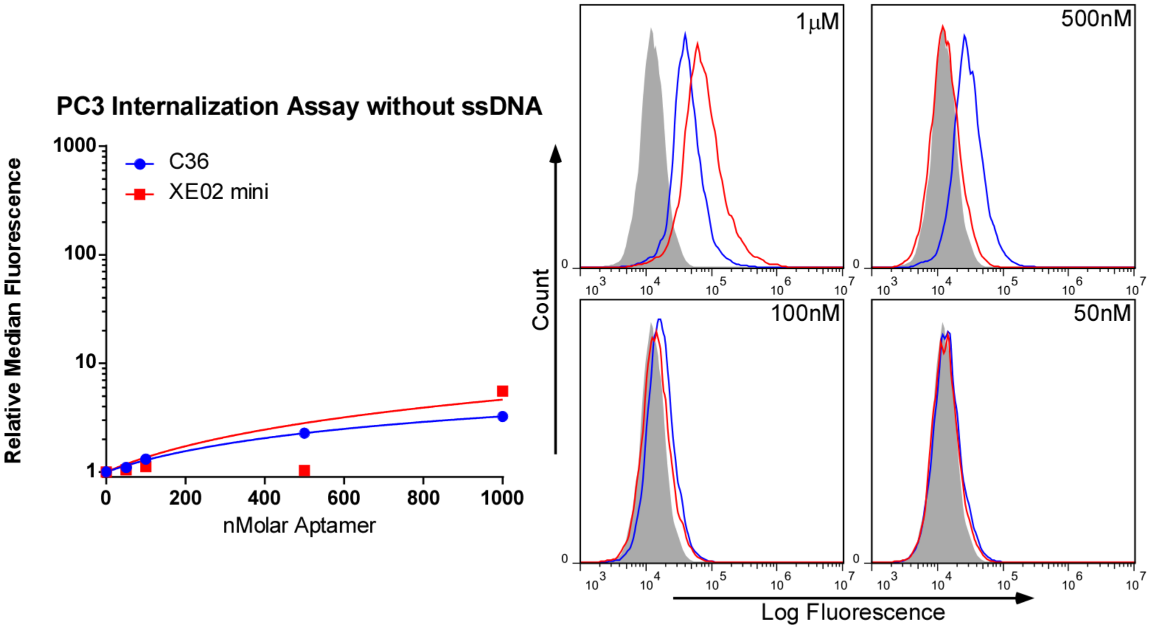

Supplementary Fig. 163. Aptamer XE02.mini internalization and binding assays on PC3 cells. Graphs represent the median fluorescence of the aptamer (Red) and C36 (Blue) relative to unstained cells (Gray).

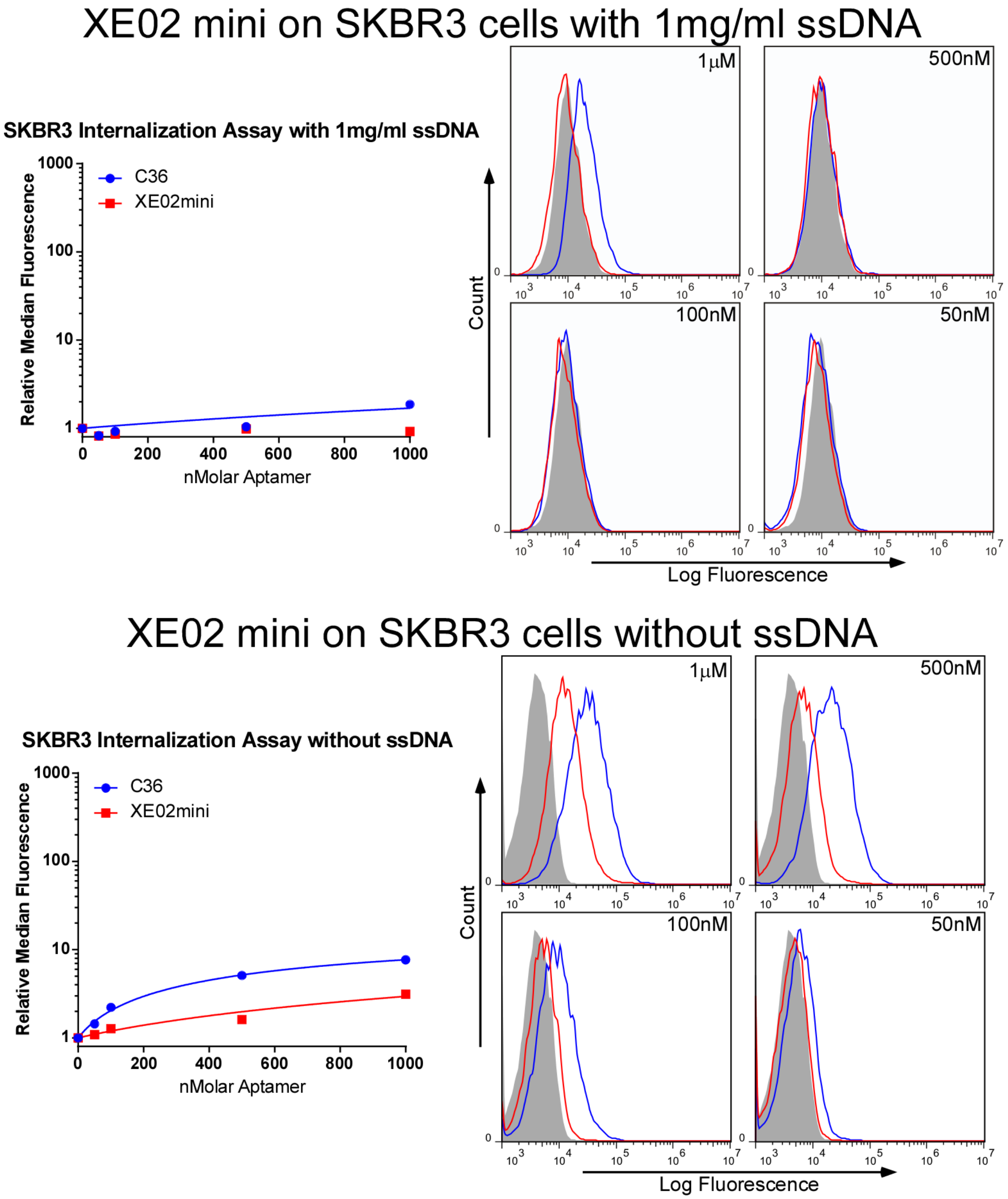

Supplementary Fig. 164. Aptamer XE02.mini internalization and binding assays on SKBR3 cells. Graphs represent the median fluorescence of the aptamer (Red) and C36 (Blue) relative to unstained cells (Gray).

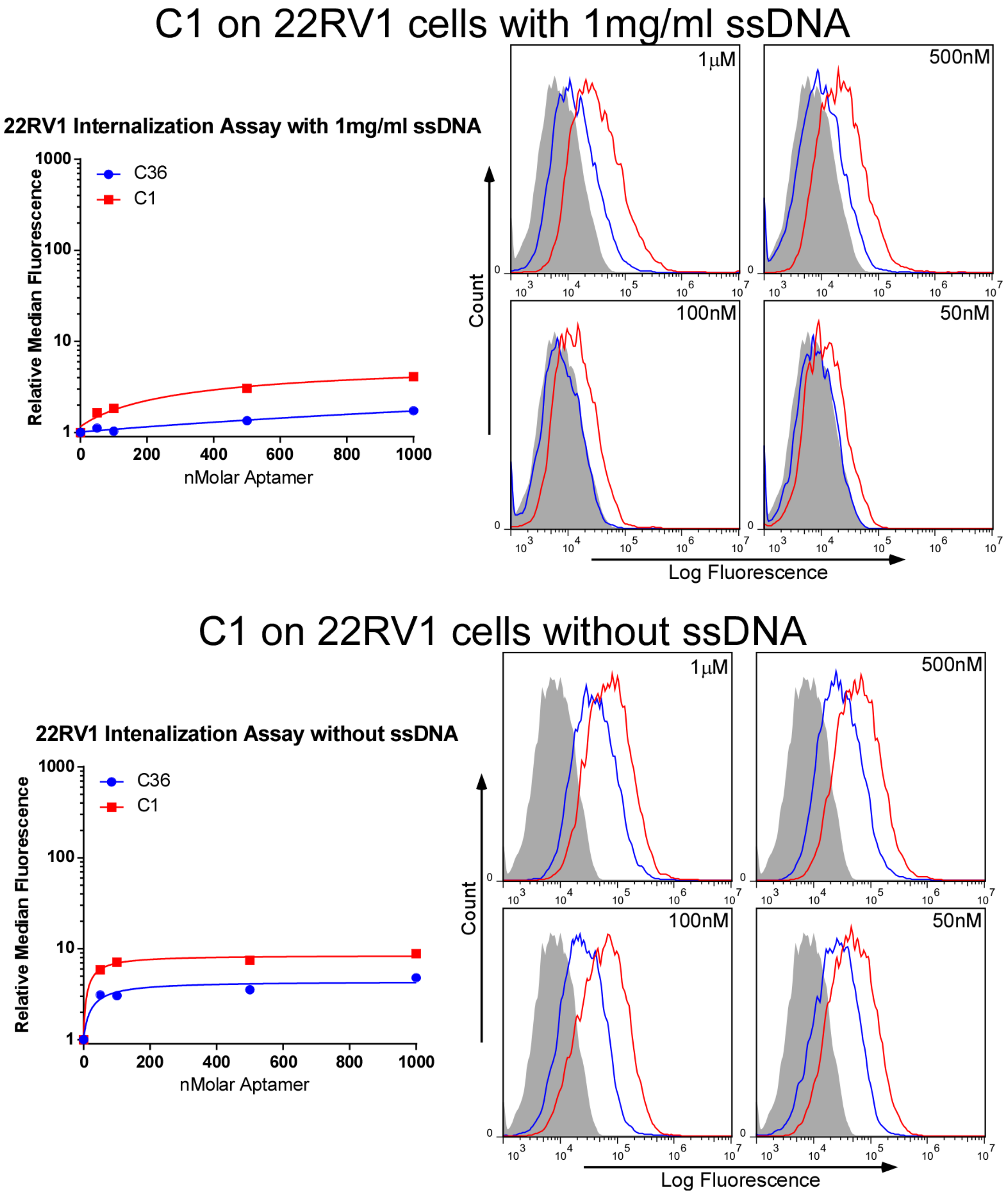

Supplementary Fig. 165. Aptamer C1 internalization and binding assays on 22RV1 cells. Graphs represent the median fluorescence of the aptamer (Red) and C36 (Blue) relative to unstained cells (Gray).

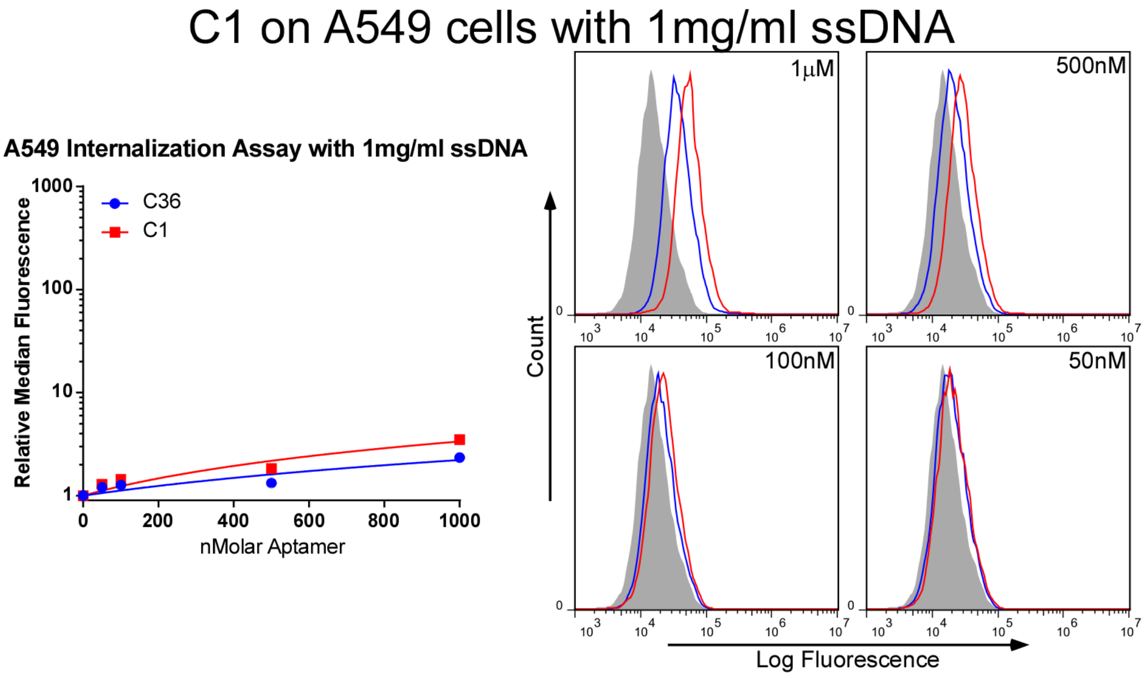

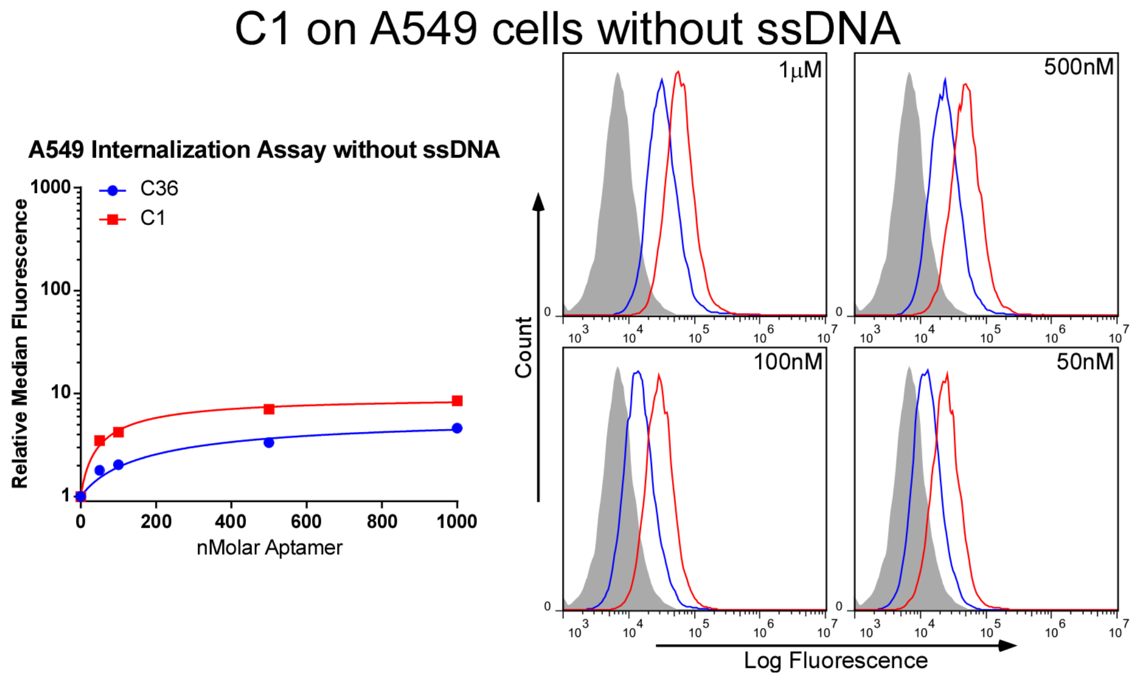

Supplementary Fig. 166. Aptamer C1 internalization and binding assays on A549 cells. Graphs represent the median fluorescence of the aptamer (Red) and C36 (Blue) relative to unstained cells (Gray).

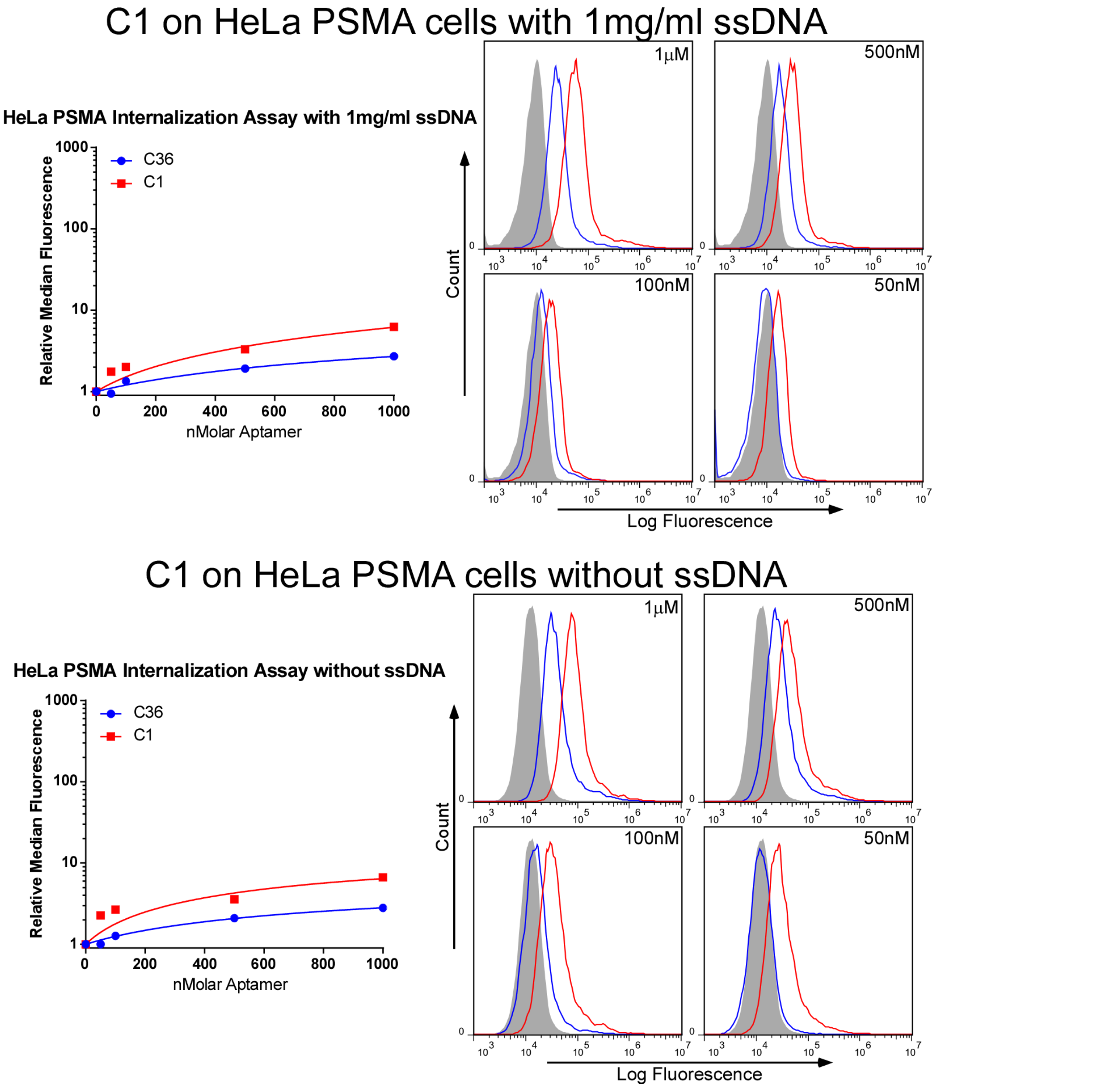

Supplementary Fig. 167. Aptamer C1 internalization and binding assays on HeLa PSMA cells. Graphs represent the median fluorescence of the aptamer (Red) and C36 (Blue) relative to unstained cells (Gray).

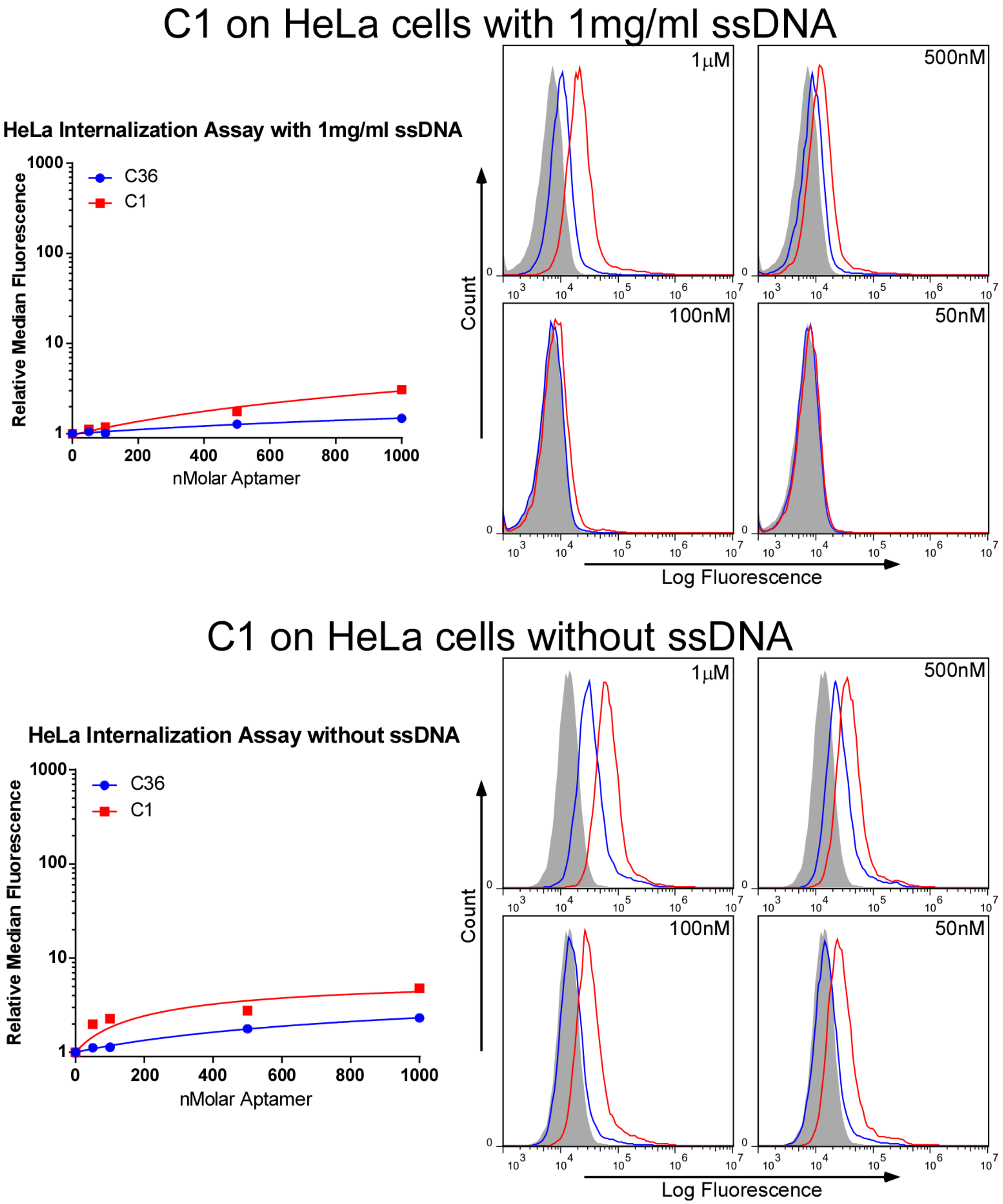

Supplementary Fig. 168. Aptamer C1 internalization and binding assays on HeLa cells. Graphs represent the median fluorescence of the aptamer (Red) and C36 (Blue) relative to unstained cells (Gray).

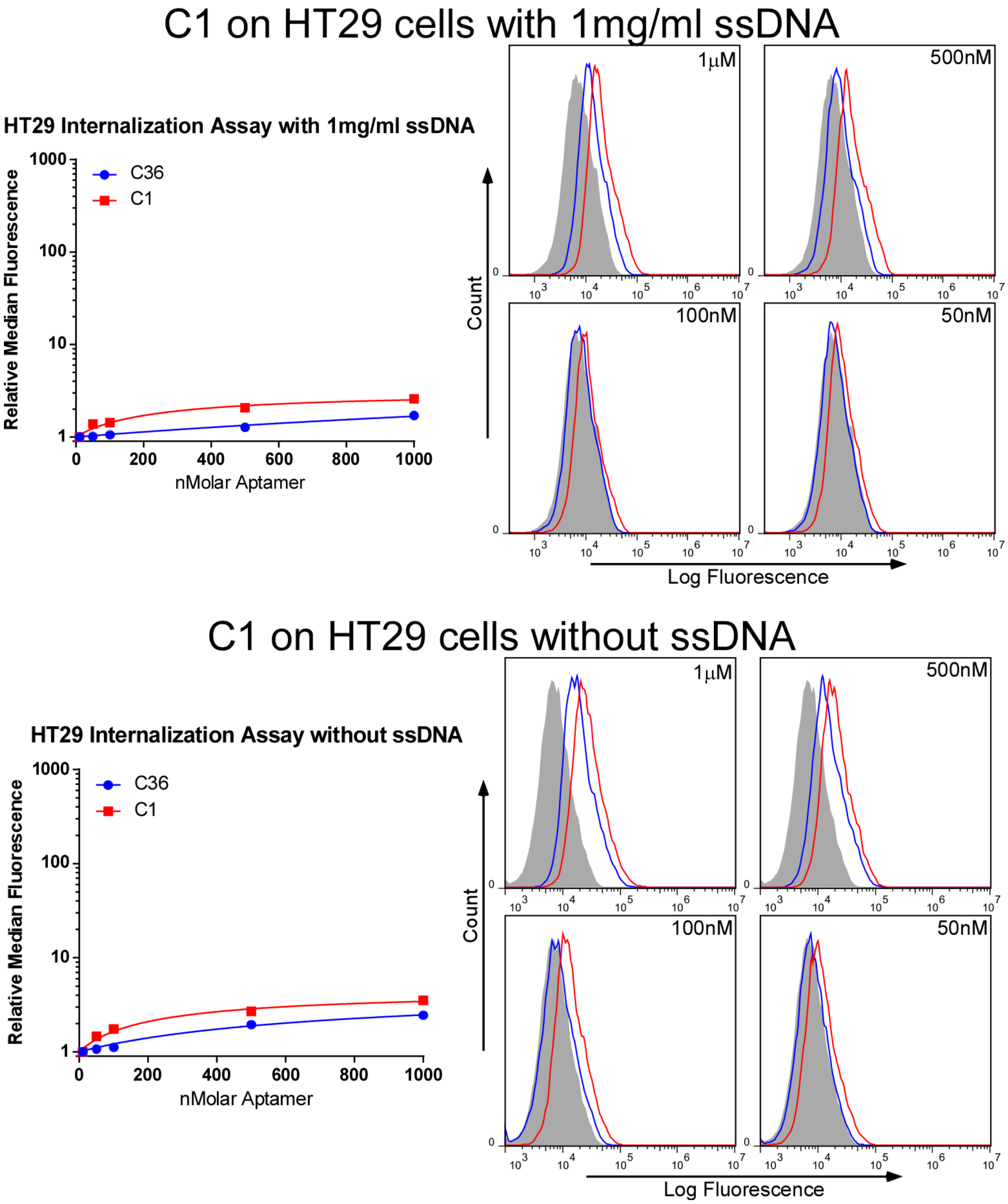

Supplementary Fig. 169. Aptamer C1 internalization and binding assays on HT29 cells. Graphs represent the median fluorescence of the aptamer (Red) and C36 (Blue) relative to unstained cells (Gray).

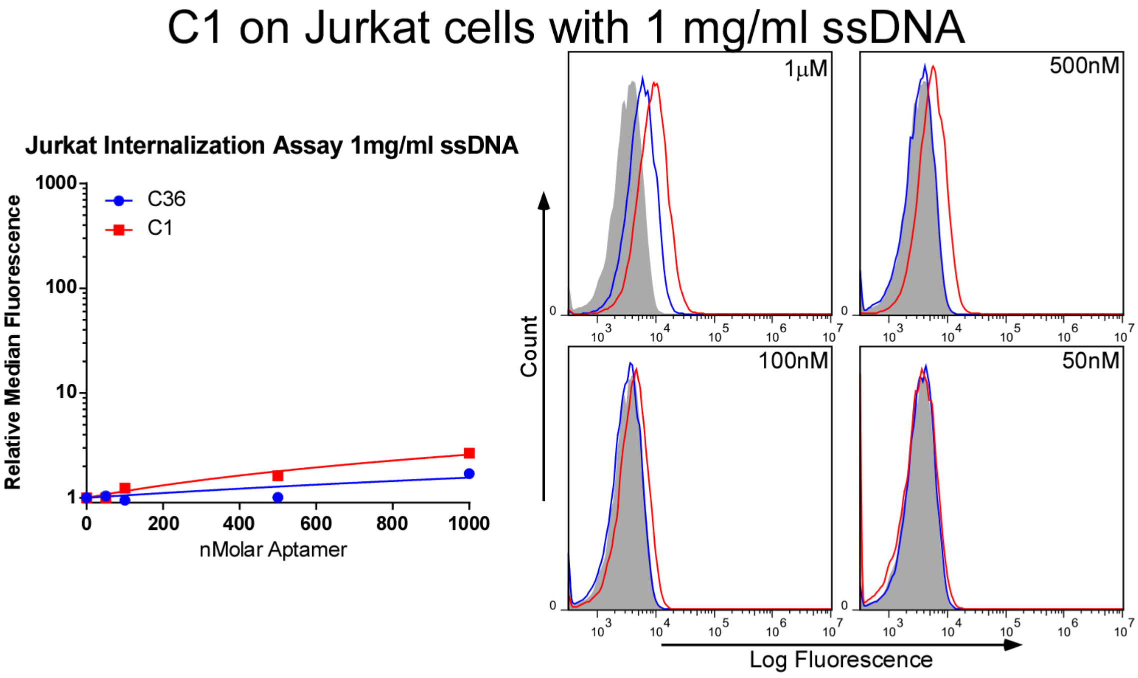

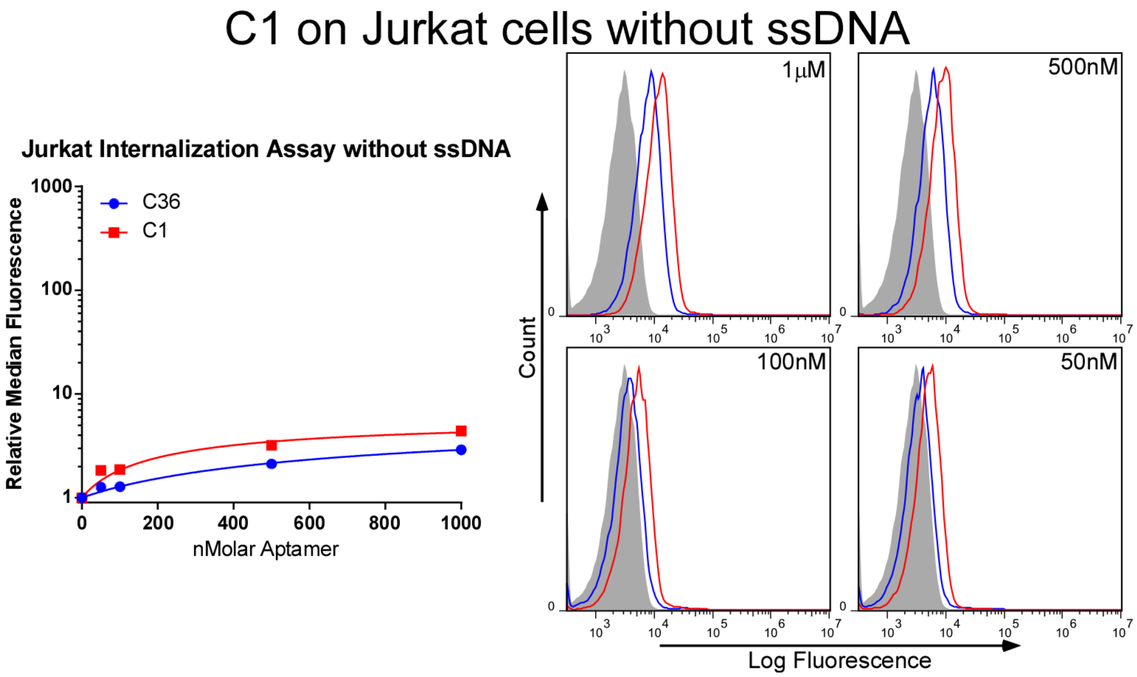

Supplementary Fig. 170. Aptamer C1 internalization and binding assays on Jurkat cells. Graphs represent the median fluorescence of the aptamer (Red) and C36 (Blue) relative to unstained cells (Gray).

C1 on LNCaP cells with 1mg/ml ssDNA

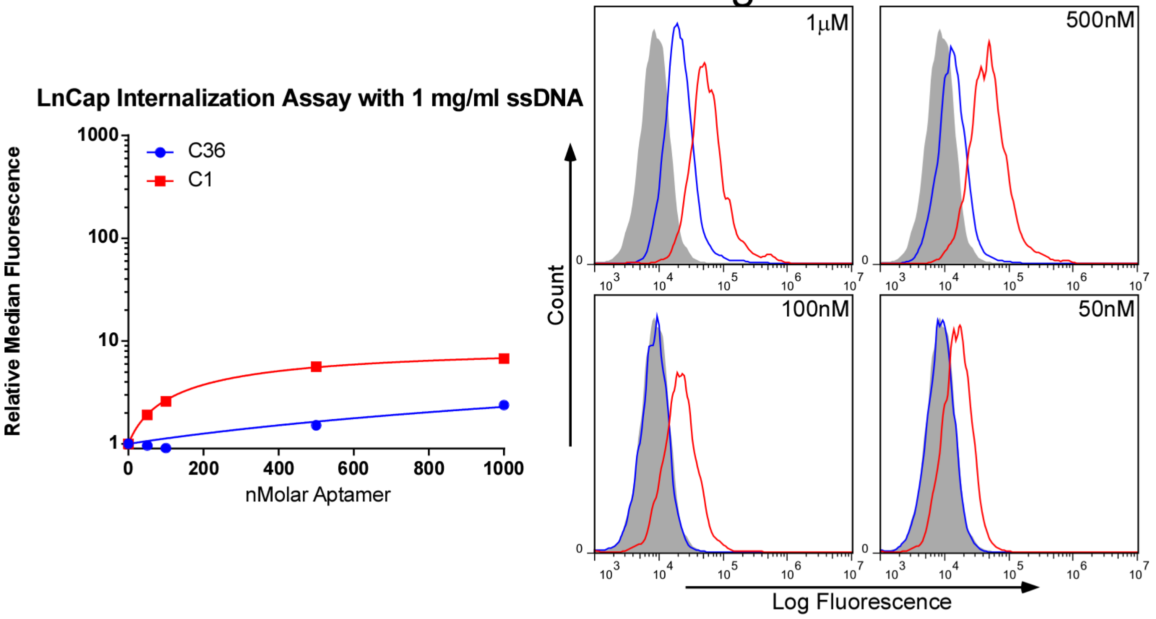

C1 on LNCaP cells without ssDNA

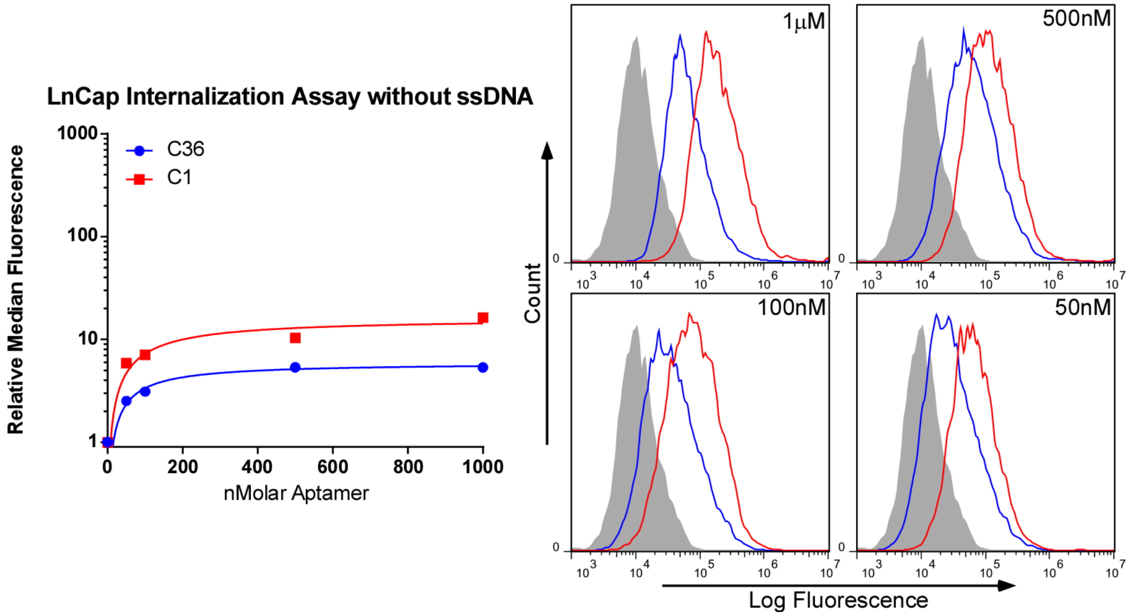

Supplementary Fig. 171. Aptamer C1 internalization and binding assays on LNCaP cells. Graphs represent the median fluorescence of the aptamer (Red) and C36 (Blue) relative to unstained cells (Gray).

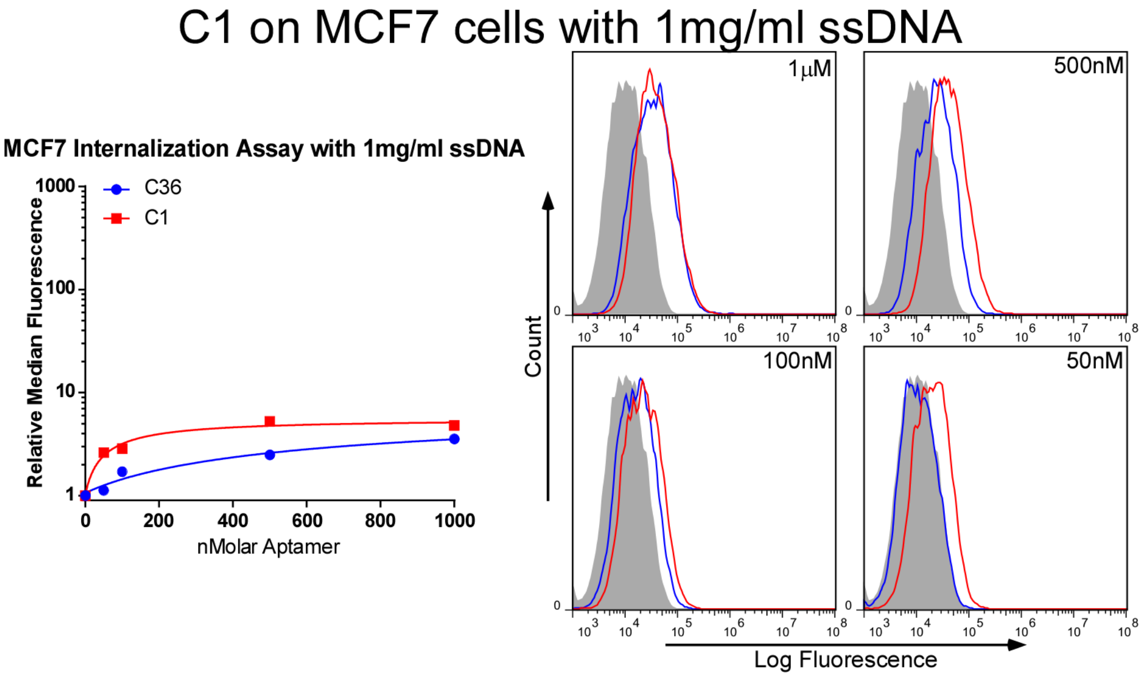

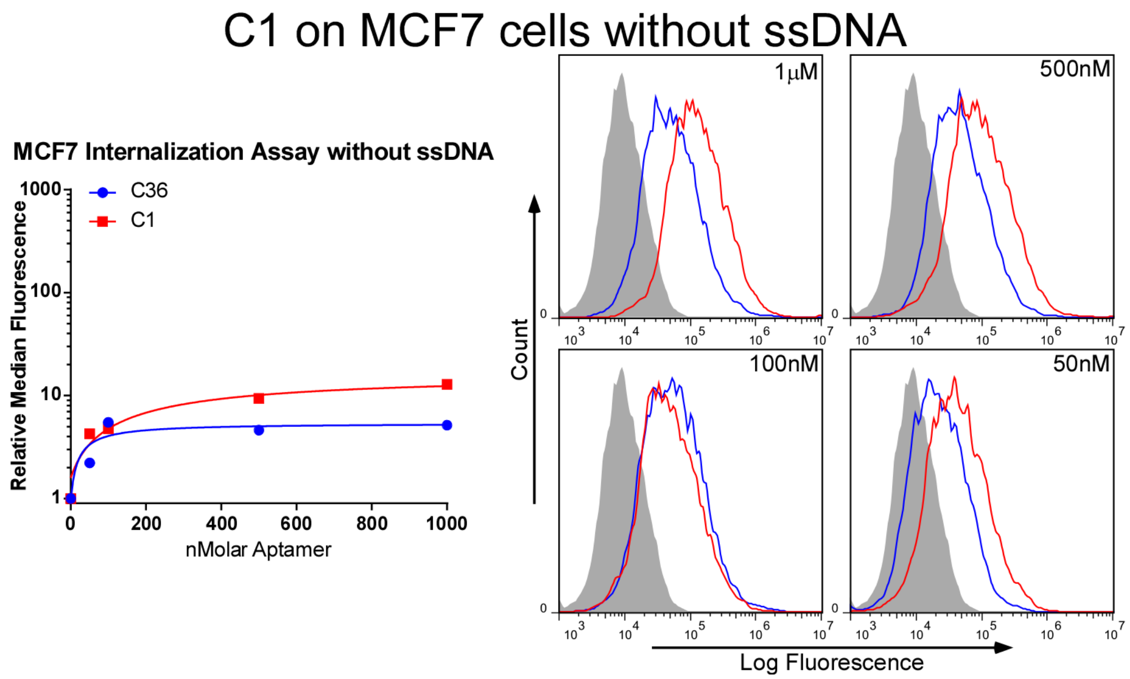

Supplementary Fig. 172. Aptamer C1 internalization and binding assays on MCF7 cells. Graphs represent the median fluorescence of the aptamer (Red) and C36 (Blue) relative to unstained cells (Gray).

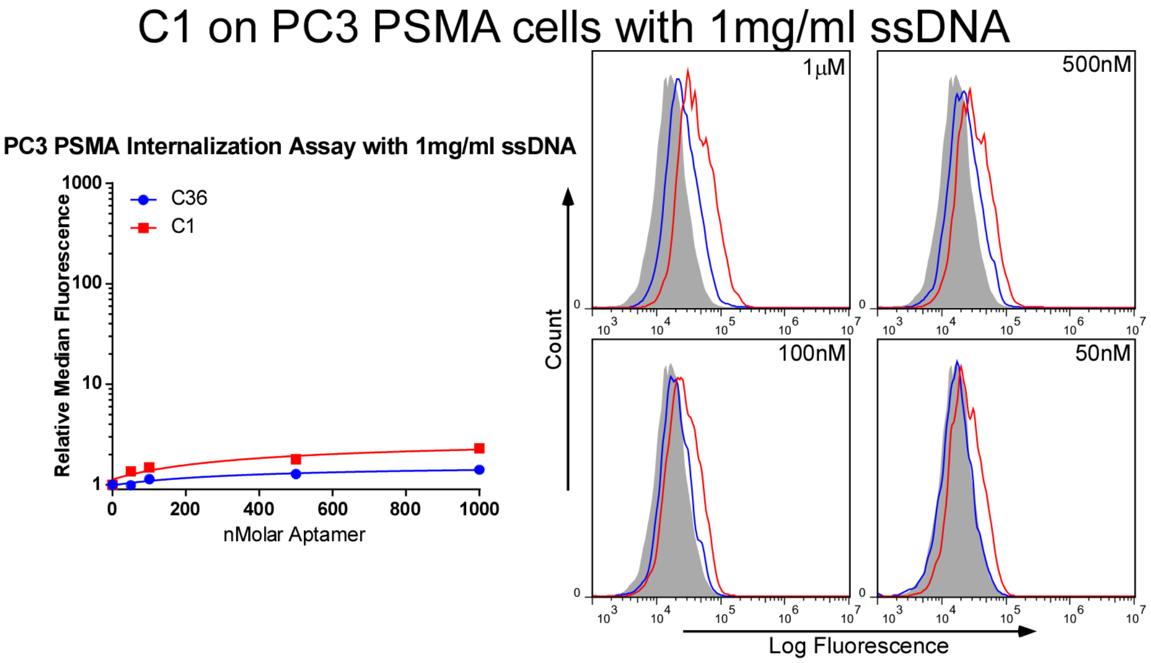

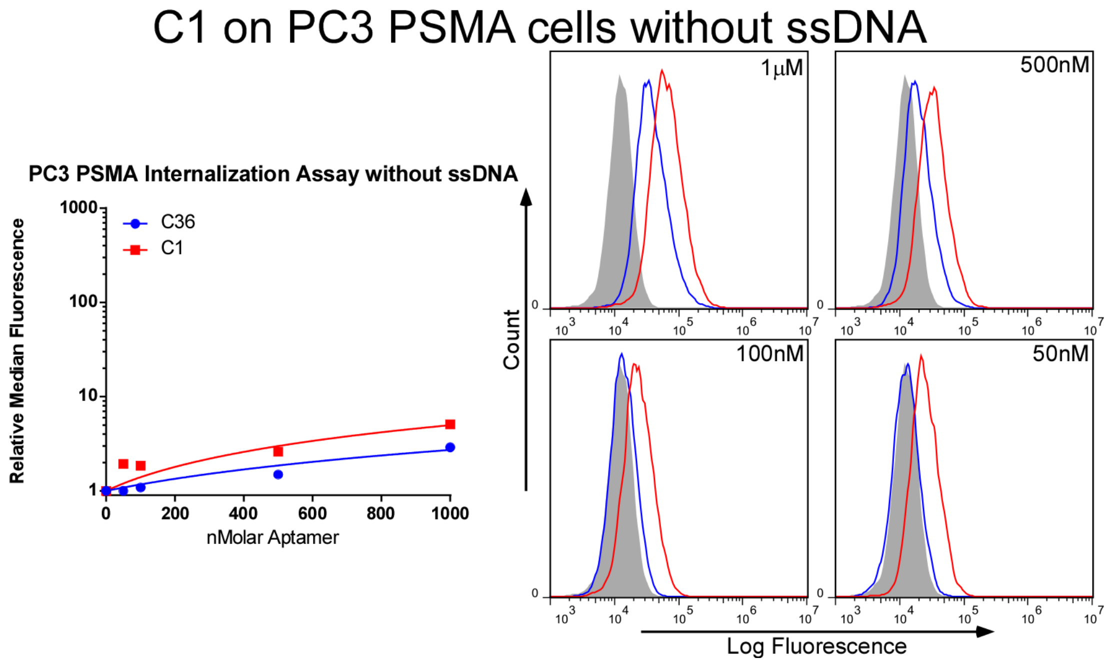

Supplementary Fig. 173. Aptamer C1 internalization and binding assays on PC3 PSMA cells. Graphs represent the median fluorescence of the aptamer (Red) and C36 (Blue) relative to unstained cells (Gray).

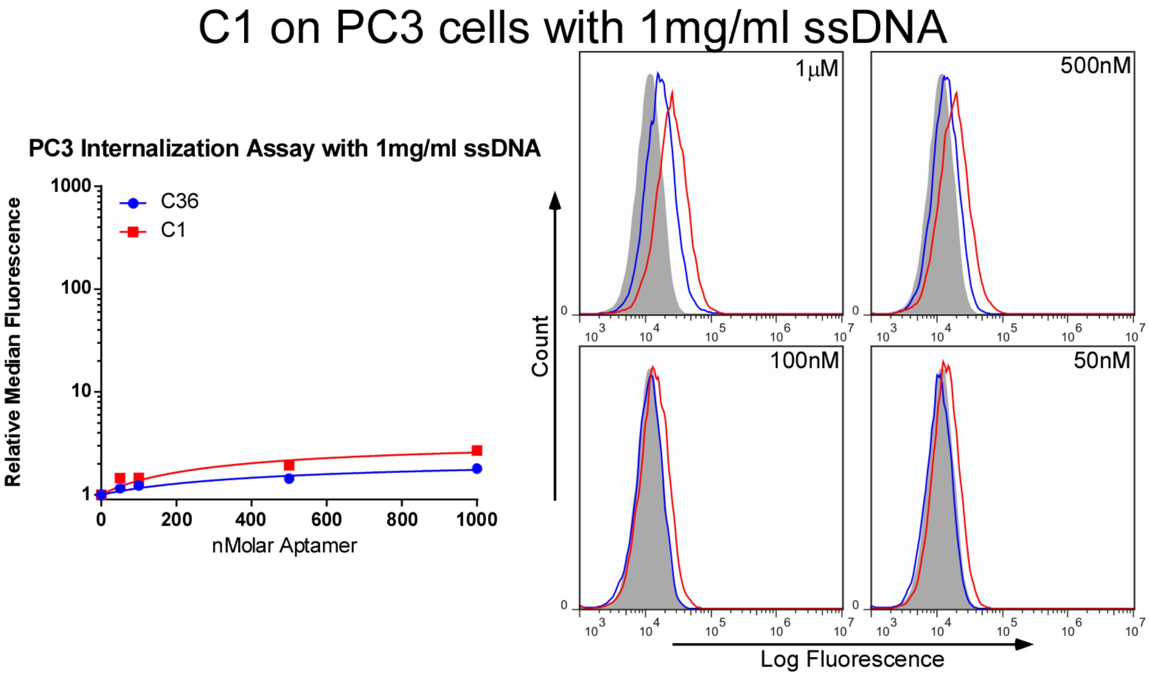

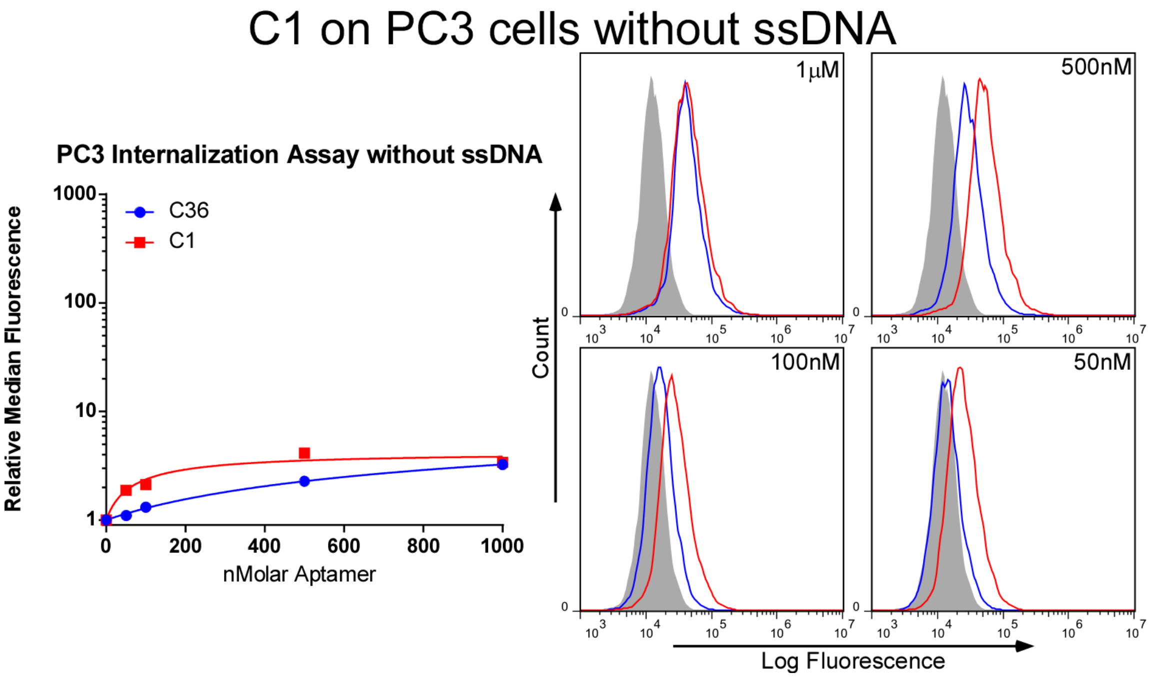

Supplementary Fig. 174. Aptamer C1 internalization and binding assays on PC3 cells. Graphs represent the median fluorescence of the aptamer (Red) and C36 (Blue) relative to unstained cells (Gray).

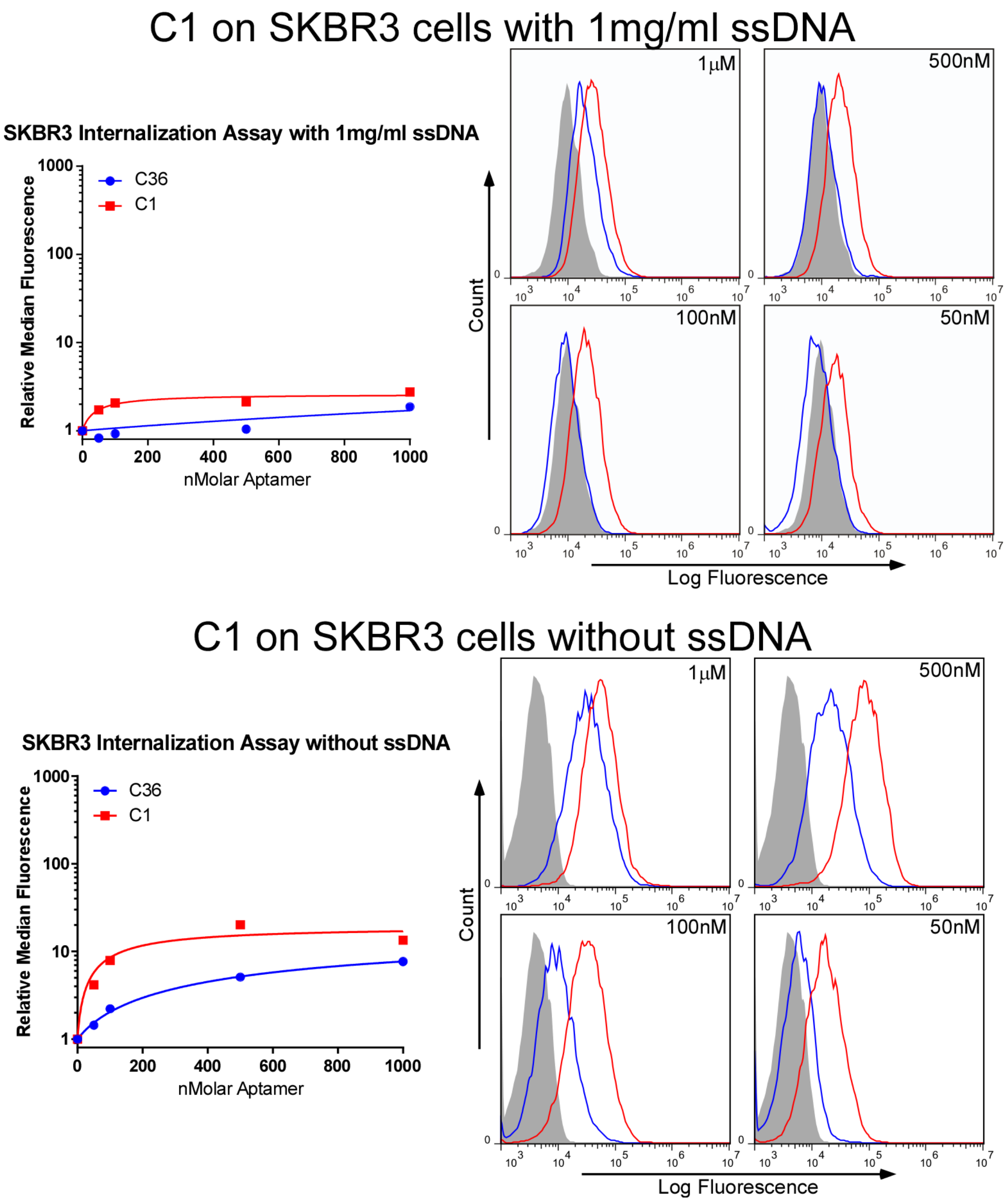

Supplementary Fig. 175. Aptamer C1 internalization and binding assays on PC3 cells. Graphs represent the median fluorescence of the aptamer (Red) and C36 (Blue) relative to unstained cells (Gray).

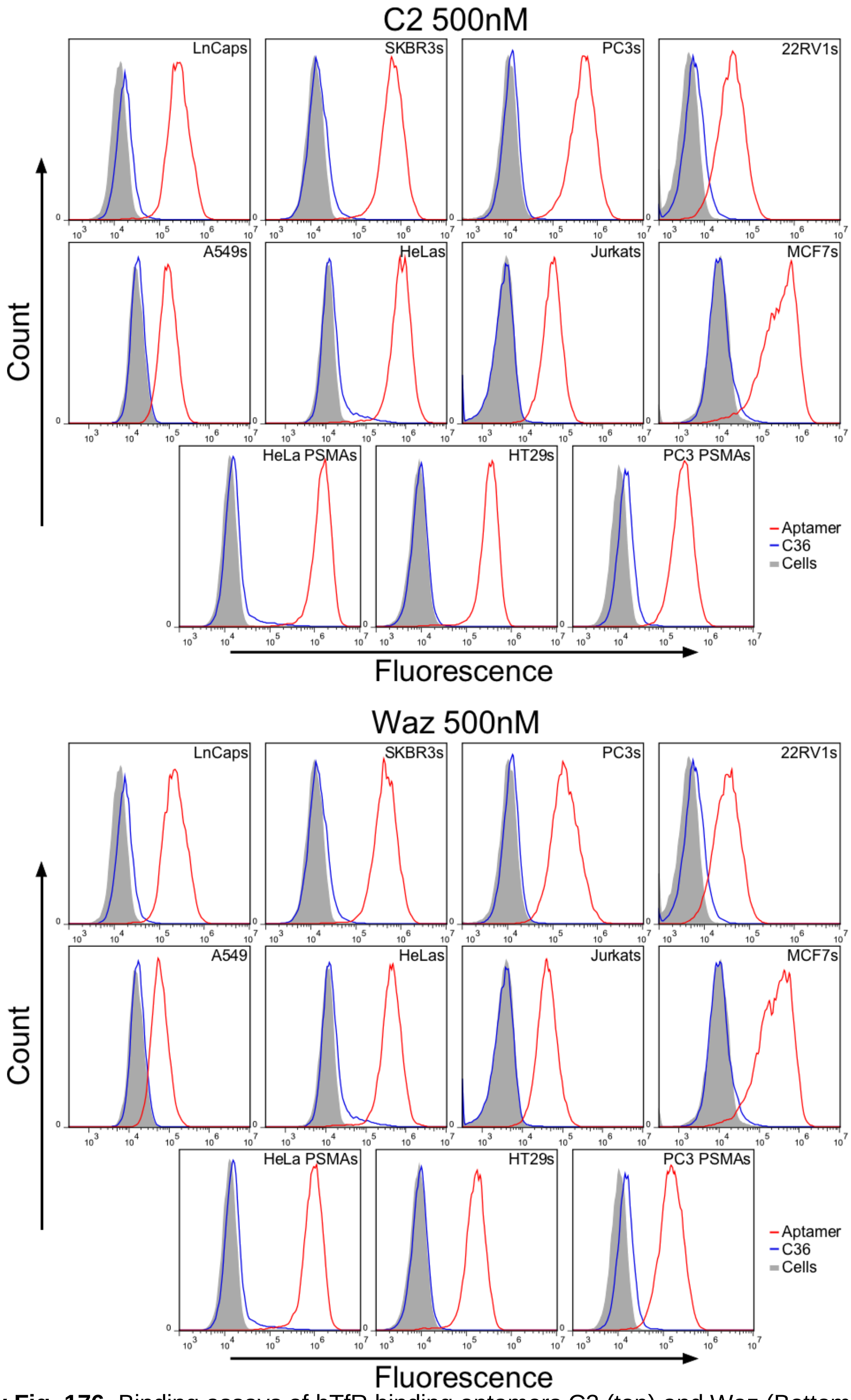

Supplementary Fig. 176. Binding assays of hTfR binding aptamers C2 (top) and Waz (Bottom). Aptamers (red) at 500nM compared to C36 (blue) staining and unstained cells (gray).

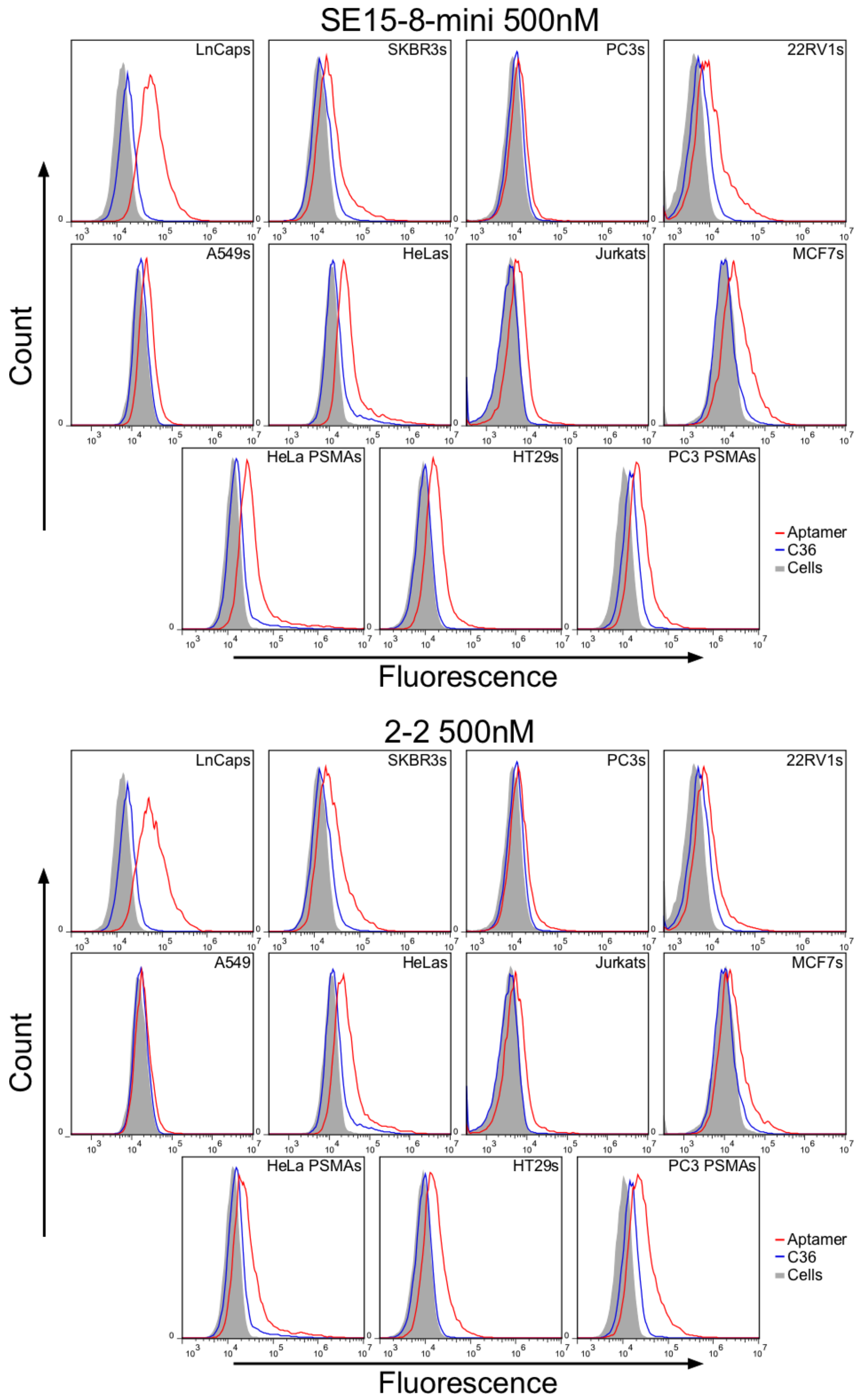

Supplementary Fig. 177. Binding assays of HER2 targeted aptamers SE15-8-mini (top) and 2-2(t) (Bottom). Aptamers (red) at 500nM compared to C36 (blue) staining and unstained cells (gray).

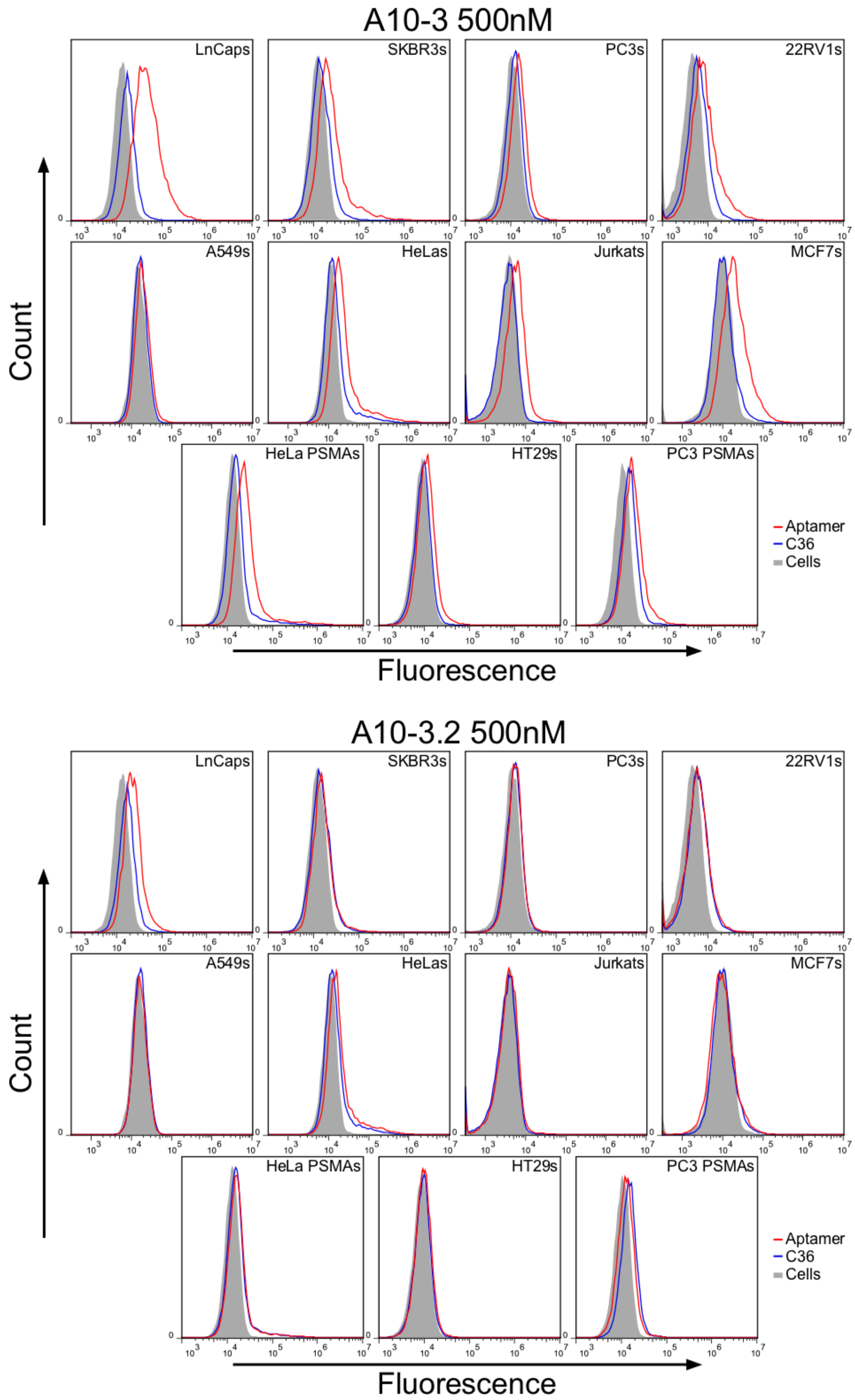

Supplementary Fig. 178. Binding assays of PSMA targeted aptamers A10-3 (top) and A10-3.2 (Bottom). Aptamers (red) at 500nM compared to C36 (blue) staining and unstained cells (gray).

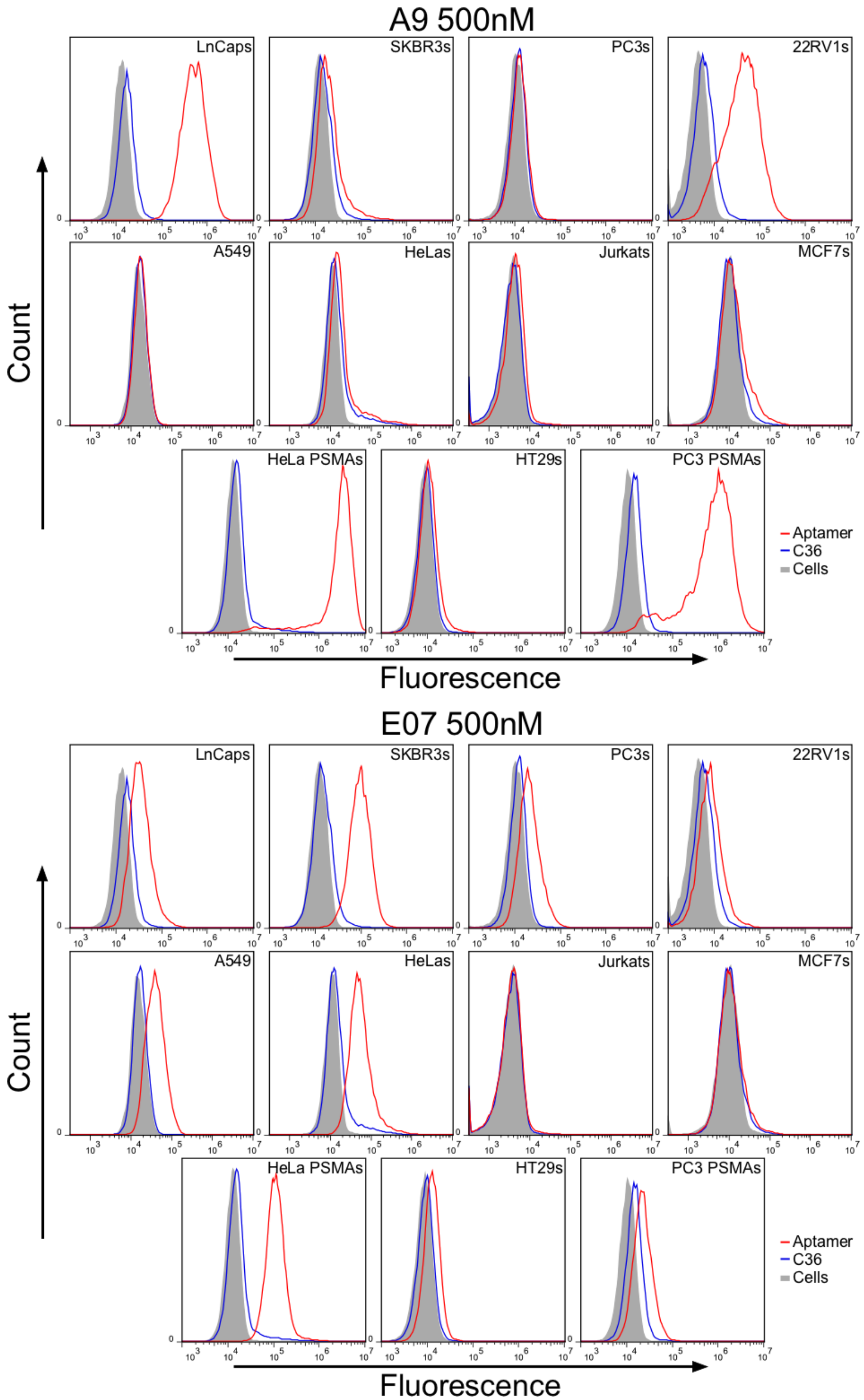

Supplementary Fig. 179. Binding assays of PSMA binding aptamer A9.min (top) and EGFR binding aptamer E07 (Bottom). Aptamers (red) at 500nM compared to C36 (blue) staining and unstained cells (gray).

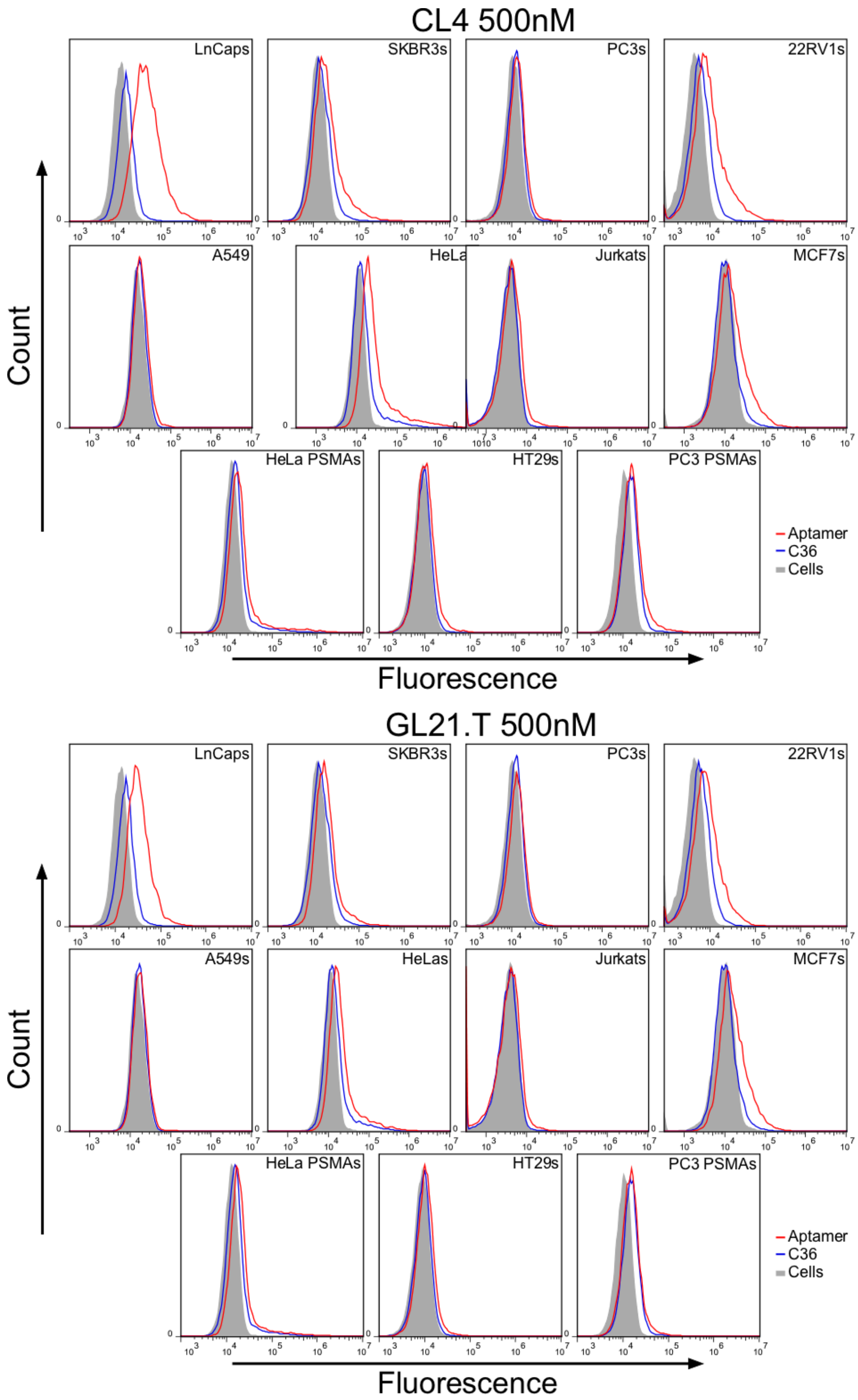

Supplementary Fig. 180. Binding assays of EGFR targeted aptamer CL4 (top) and AXL targeted aptamer GL21.T (Bottom). Aptamers (red) at 500nM compared to C36 (blue) staining and unstained cells (gray).

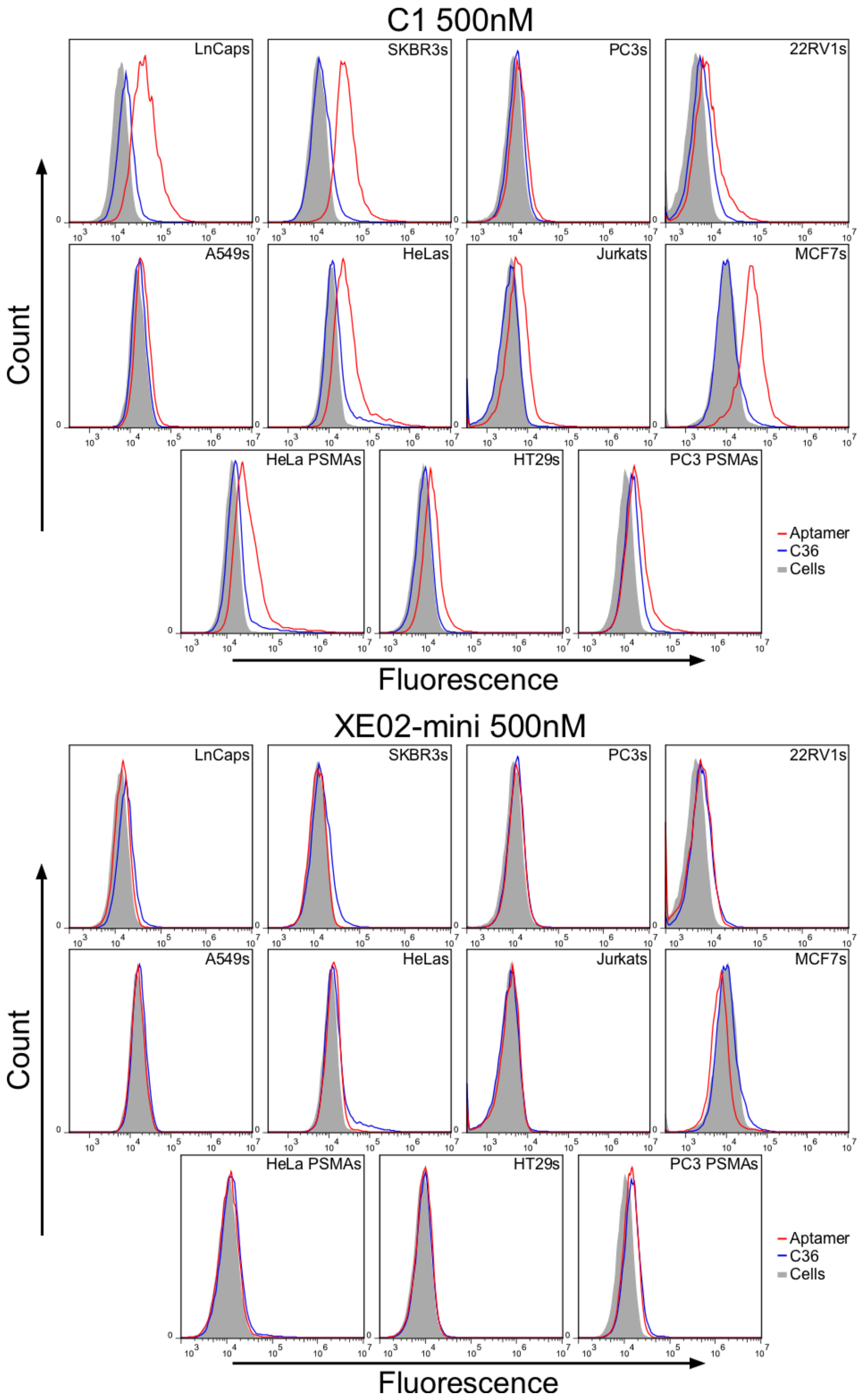

Supplementary Fig. 181. Binding assays of aptamers C1 (top) and XE02-mini (Bottom). Aptamers (red) at 500nM compared to C36 (blue) staining and unstained cells (gray).

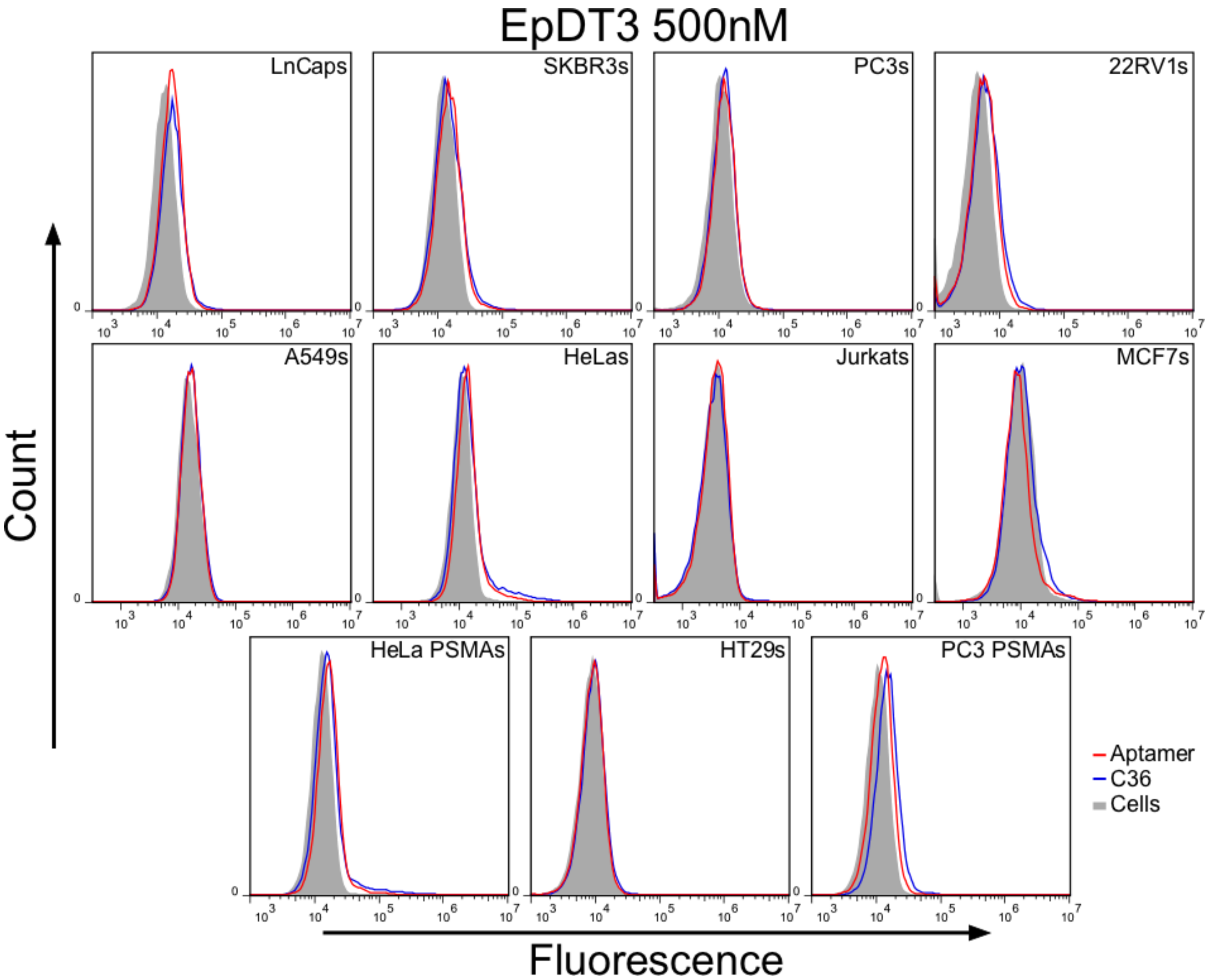

Supplementary Fig. 182. Binding assays of EpCAM targeted aptamer EpDt3. Aptamer (red) at 500nM compared to C36 (blue) staining and unstained cells (gray).

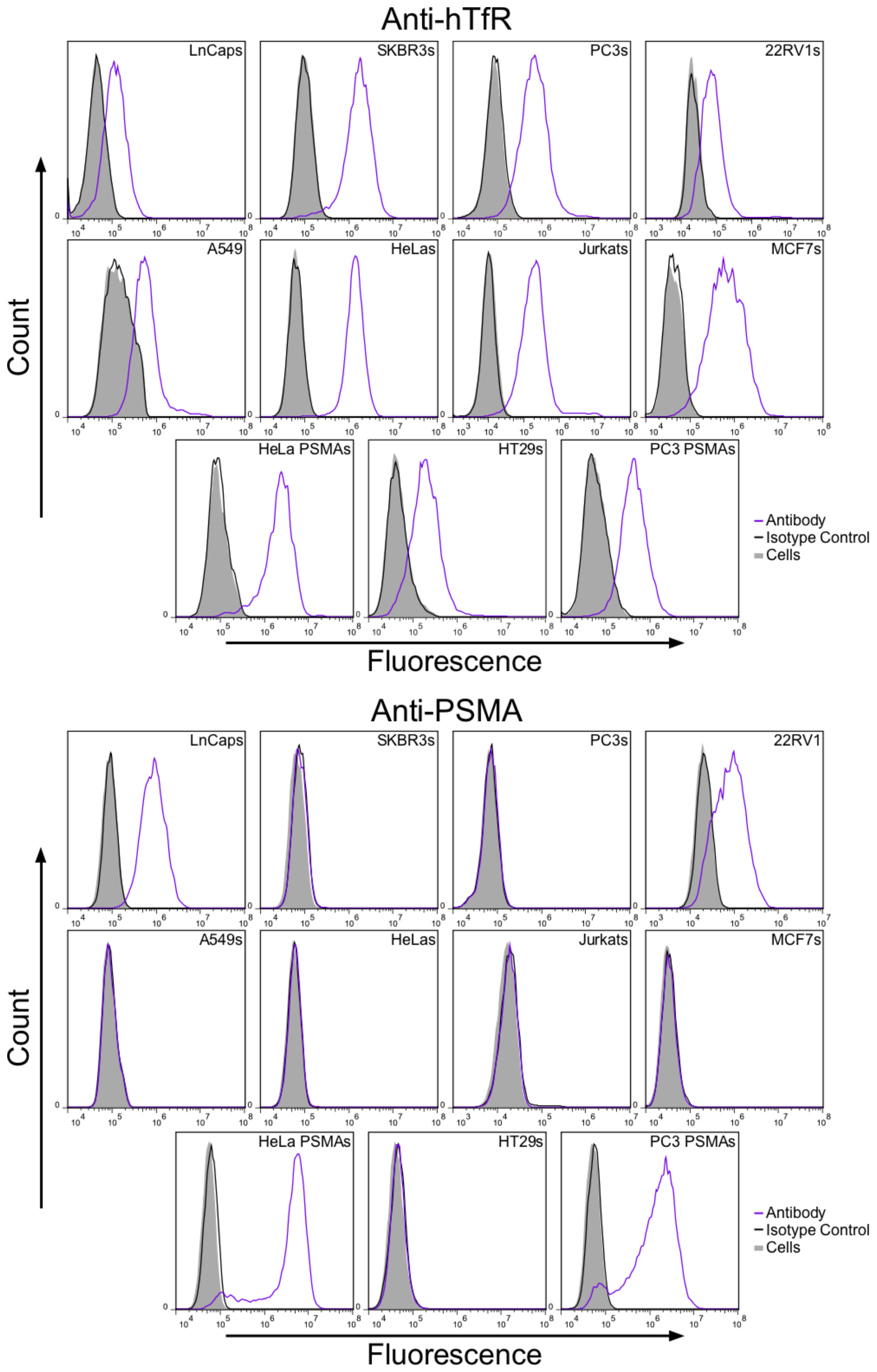

Supplementary Fig. 183. Antibody control assays for hTfR expression (top) and PSMA expression (bottom). Target specific antibodies (purple) are overlaid with isotype controls (black) and unstained cells (gray).

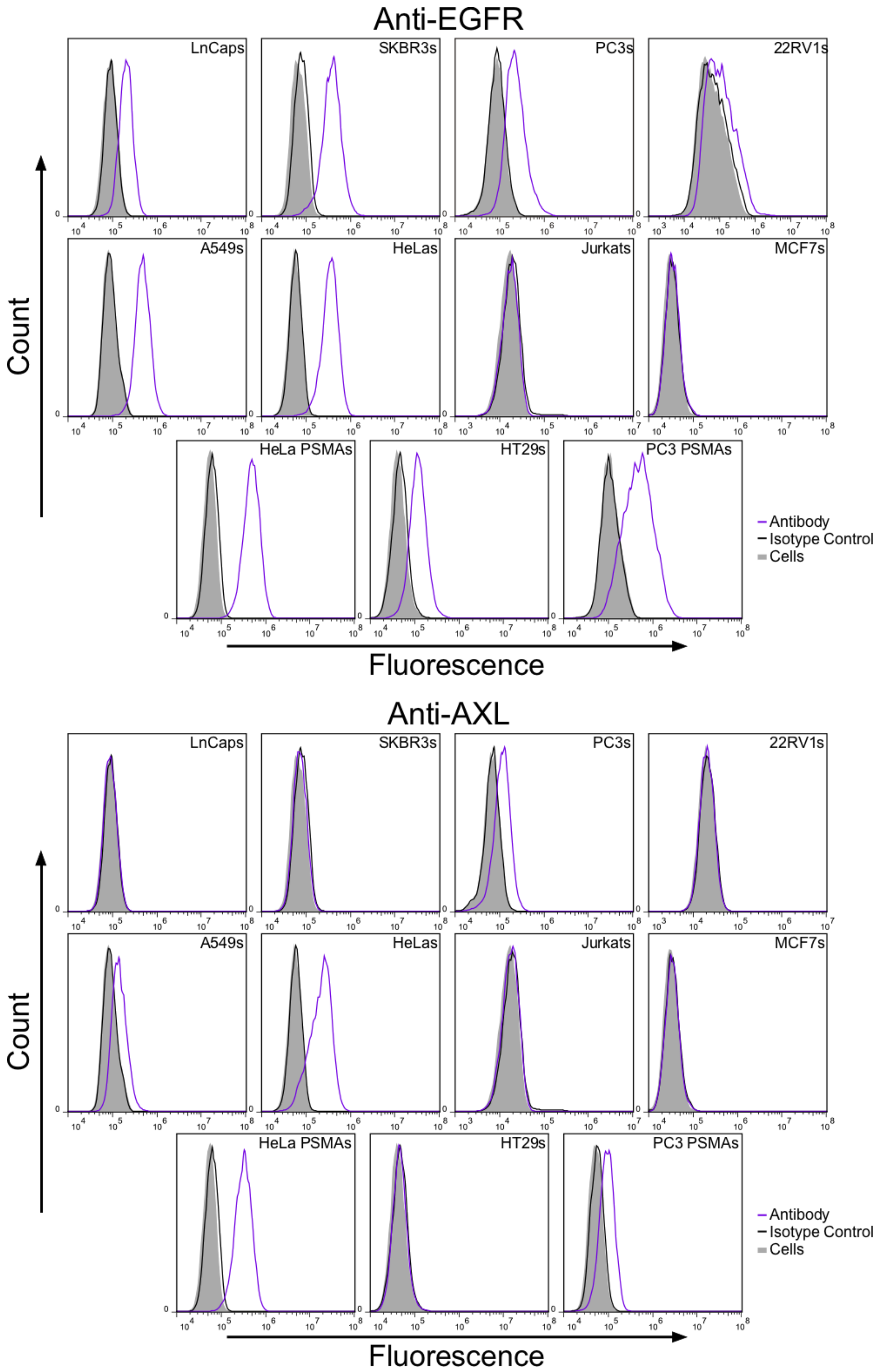

Supplementary Fig. 184. Antibody control assays for EGFR expression (top) and AXL expression (bottom). Target specific antibodies (purple) are overlaid with isotype controls (black) and unstained cells (gray).

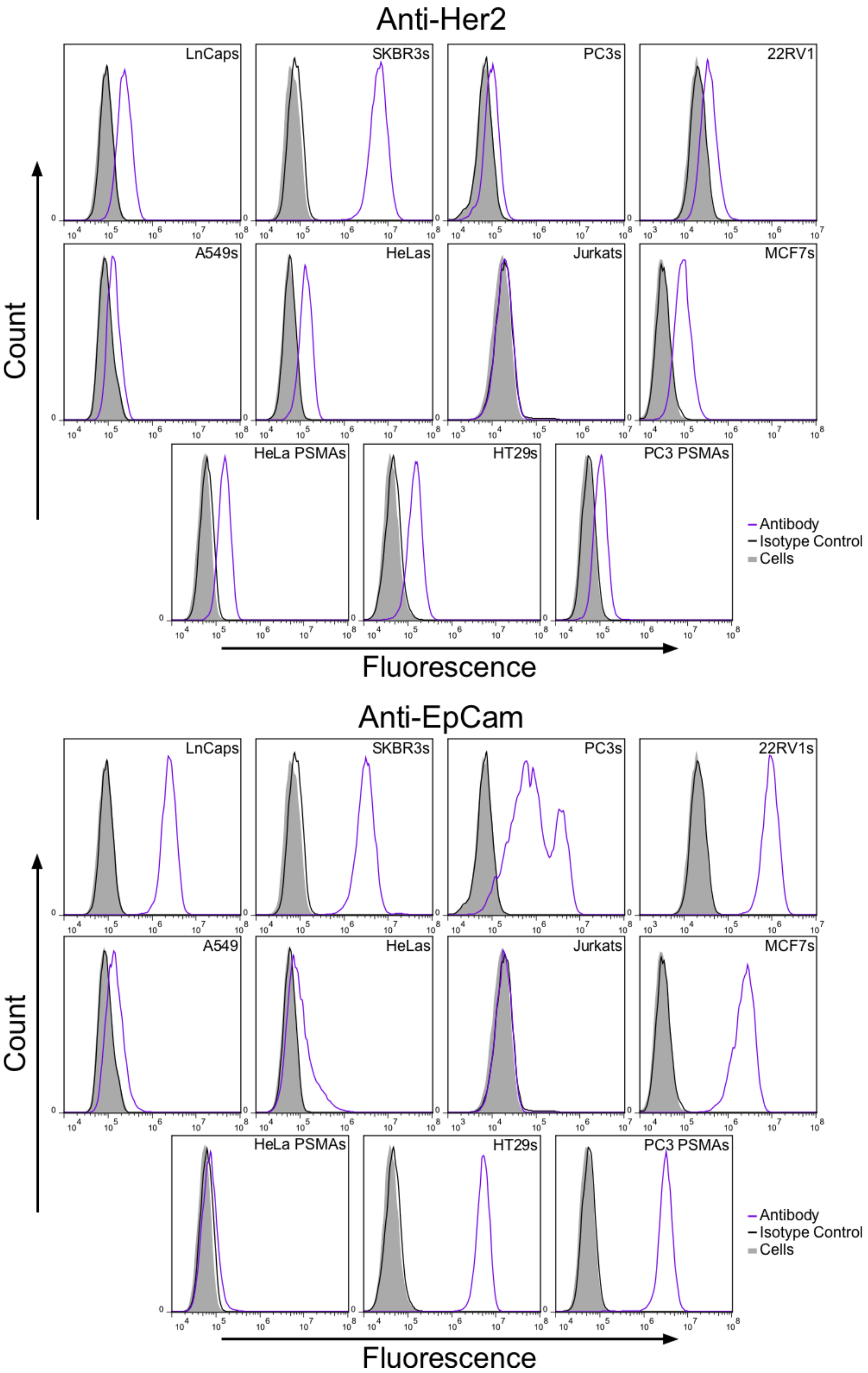

Supplementary Fig. 185. Antibody control assays for HER2 expression (top) and EpCAM expression (bottom). Target specific antibodies (purple) are overlaid with isotype controls (black) and unstained cells (gray).

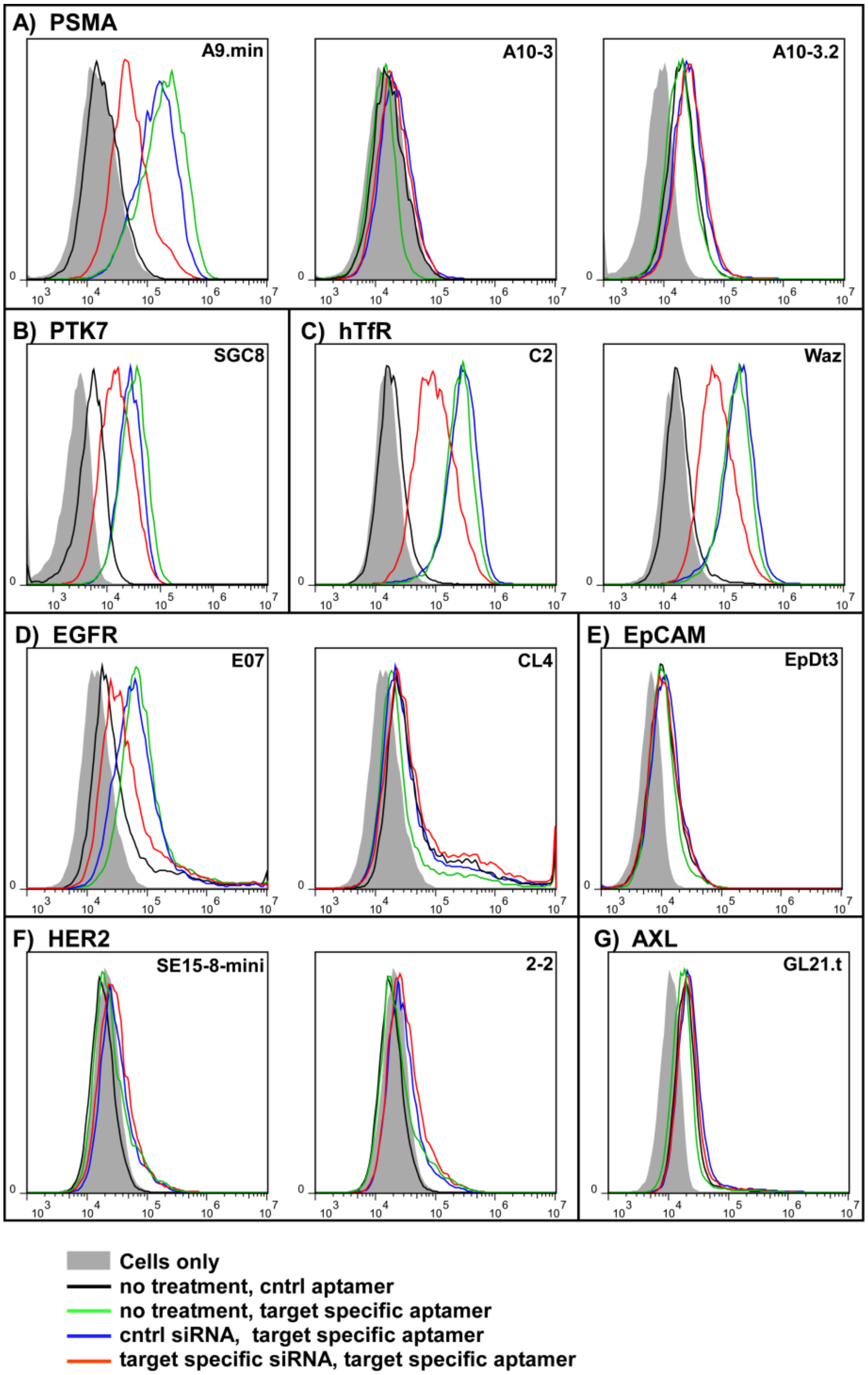

Supplementary Fig. 186. siRNA knockdown of target as assessed by flow cytometry using our cell "binding" assay format. Aptamers indicated were incubated with cells treated as indicated in HBSS containing 1% BSA and 0.1% NaN_3 and incubated for 30 minutes at room temperature. The cells were then washed then analyzed by flow cytometry. Antibody controls to validate target knockdown can be found in **Figure 4**. The cell type and aptamer concentration used for each experiment were: (A) LNCaP cells, A9.min (100nM), A10-3 (100nM) and A10-3.2 (500nM); (B) A431 cells, SGC8 (100nM); (C) HeLa cells, Waz (100nM) and C2.min (100nM); (D) HeLa cells, E07 (500nM) and CL4 (1000nM); (E) SKBR3 cells, SE15-8-mini (1000nM) and 2-2(t) (1000nM). (F) MCF7 cells, EpDt3 (1000nM). (G) HeLa cells, GL21.T (1000nM).

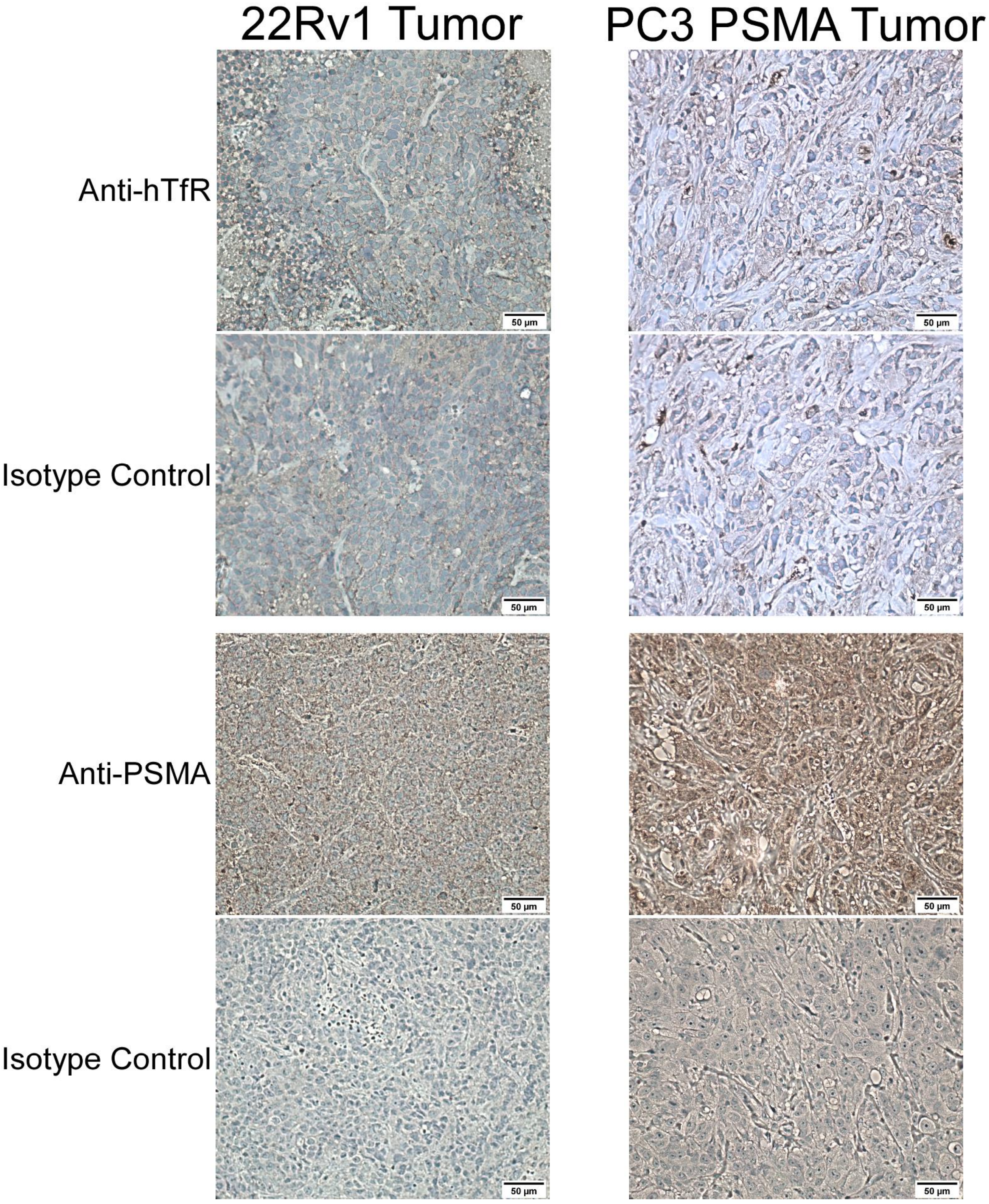

Supplementary Fig. 187. Tumor sections were stained with a primary antibody or an isotype control and a secondary antibody labeled with HRP, then stained with 3-3' diaminobenzidine (DAB), which appears brown when positive. The sections were stained anti-hTfR antibody and its isotype IgG1 control or with anti-PSMA antibody and its isotype control IgG2-k control. Nuclei were stained with DAPI and appear as blue in the images. Scale bar represents 50µm. Images are representative of 3 experimental replicates.

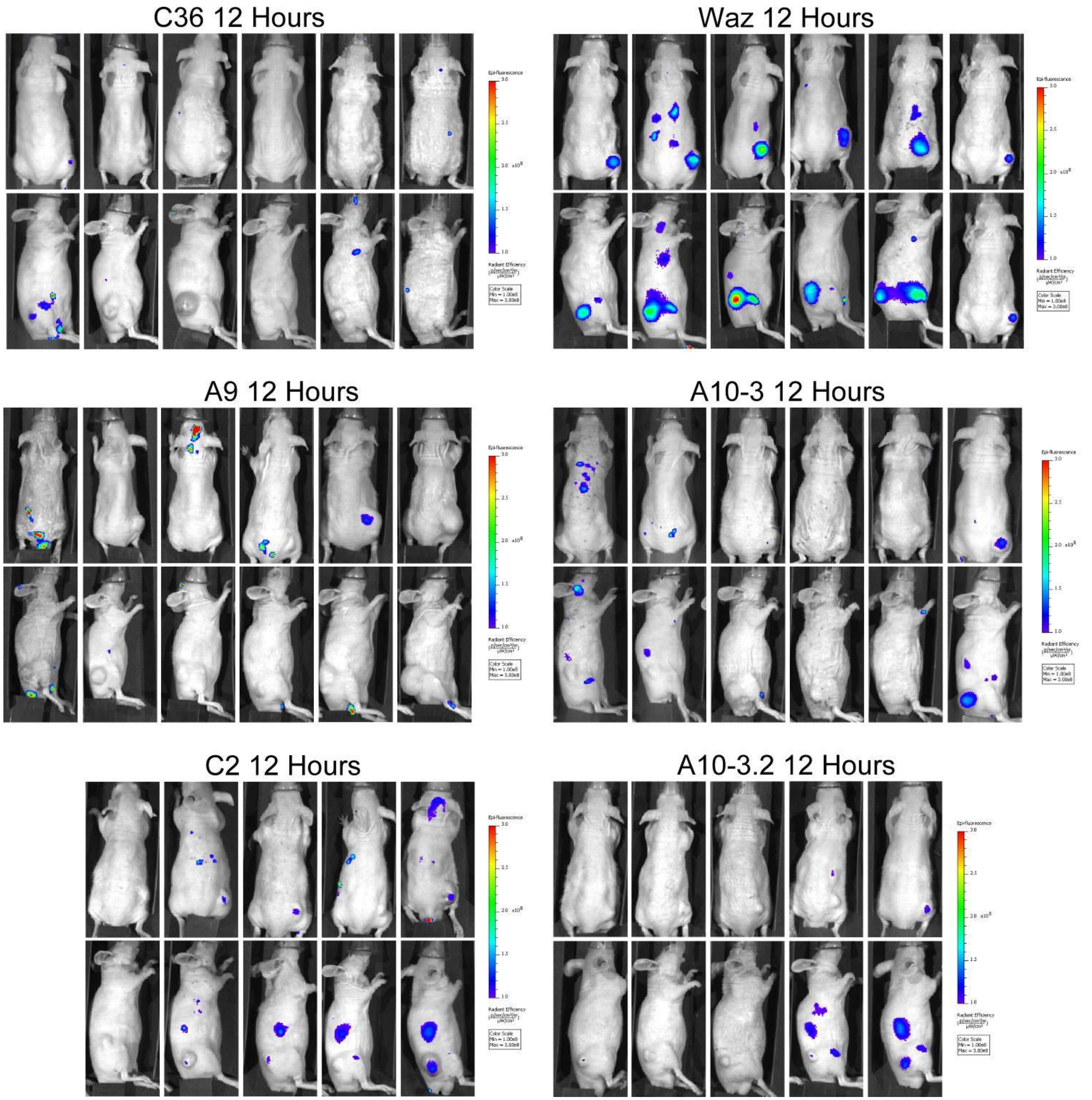

Supplementary Fig. 188. In vivo imaging of AF750 labeled aptamers in mice bearing 22Rv1 tumors, t = 12hrs. The first 3 mice in each group are identical to those shown in **Figure 5**. The data shown here includes the additional animals in each cohort, n = 5 or 6. Mice were injected with two nanomoles of 750-labeled aptamer i.v. by tail vein.

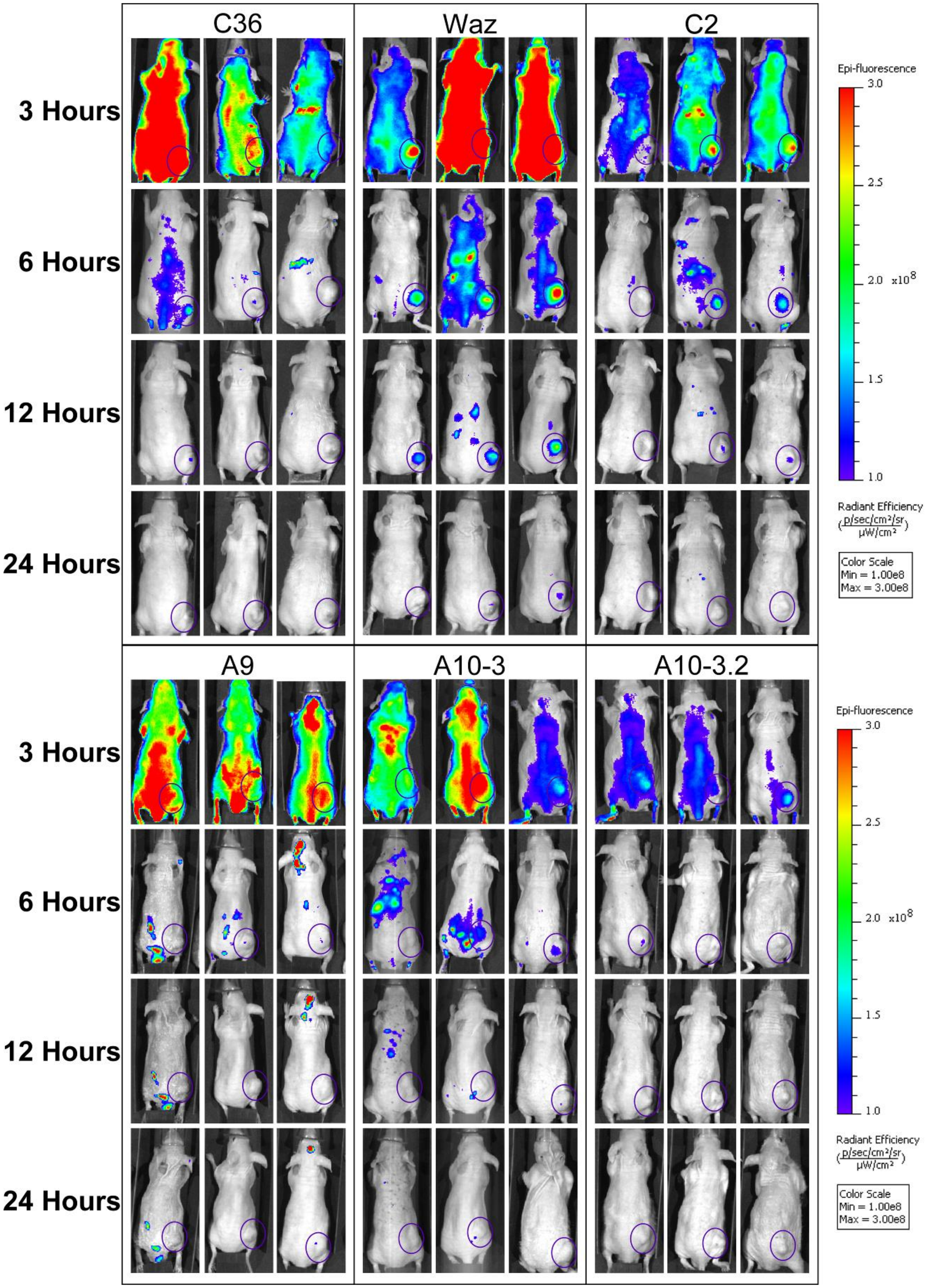

Supplementary Fig. 189. In vivo imaging of AF750 labeled aptamers in mice bearing 22Rv1 tumors, time course. Mice (n = 3) were imaged at 3, 6, 12 and 24 hours post injection of aptamers. Animals imaged at 12 hr are included in the figure shown in **S188** as well as **Figure 5**.

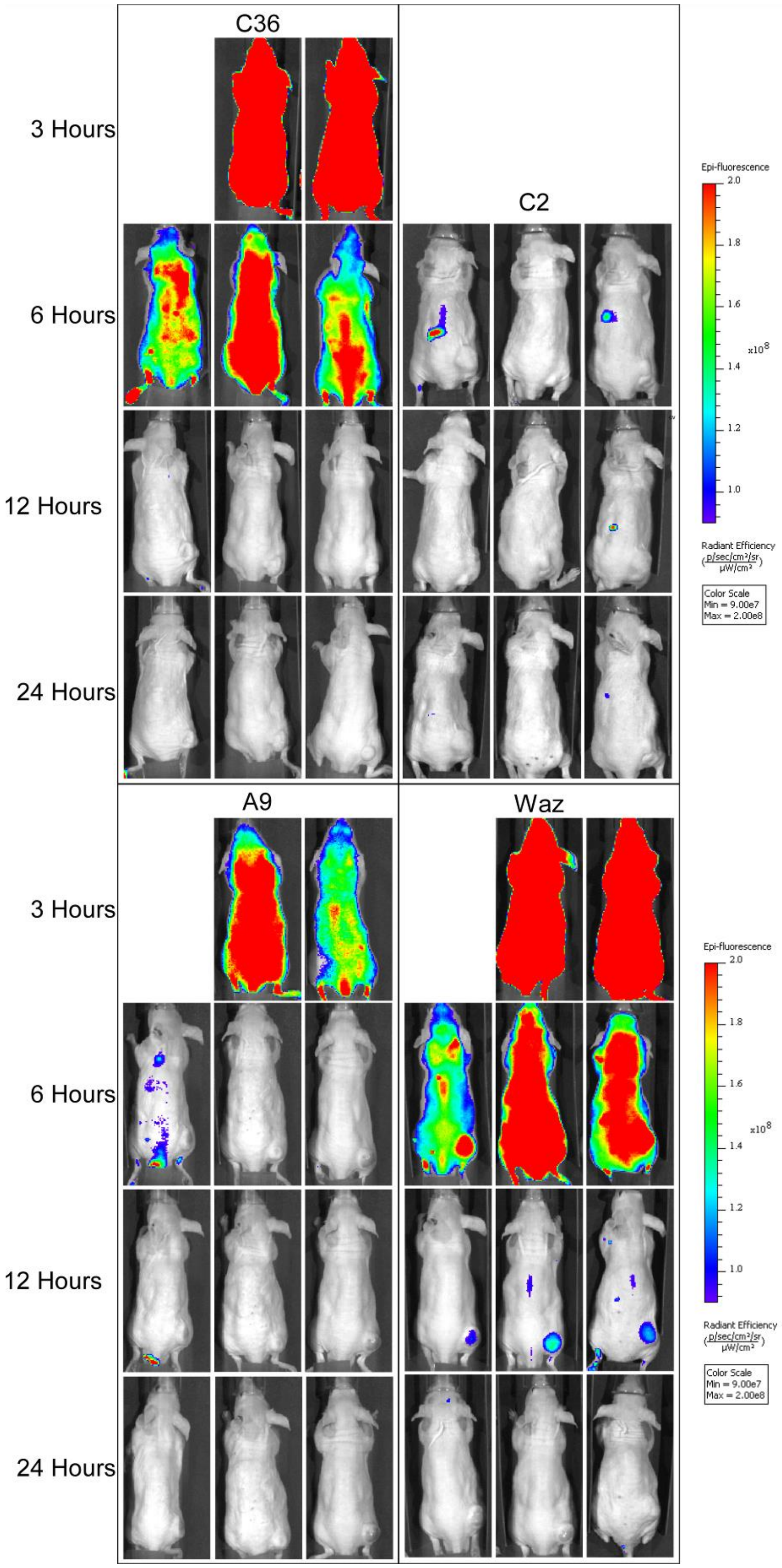

Supplementary Fig. 190. In vivo imaging of AF750 labeled aptamers in mice bearing PC3-PSMA tumors, time course. Mice (n = 3) were imaged at 3, 6, 12 and 24 hours post injection of aptamers. Animals imaged at 12 hr are included in the figure shown in **S186** as well as **Figure 5**.

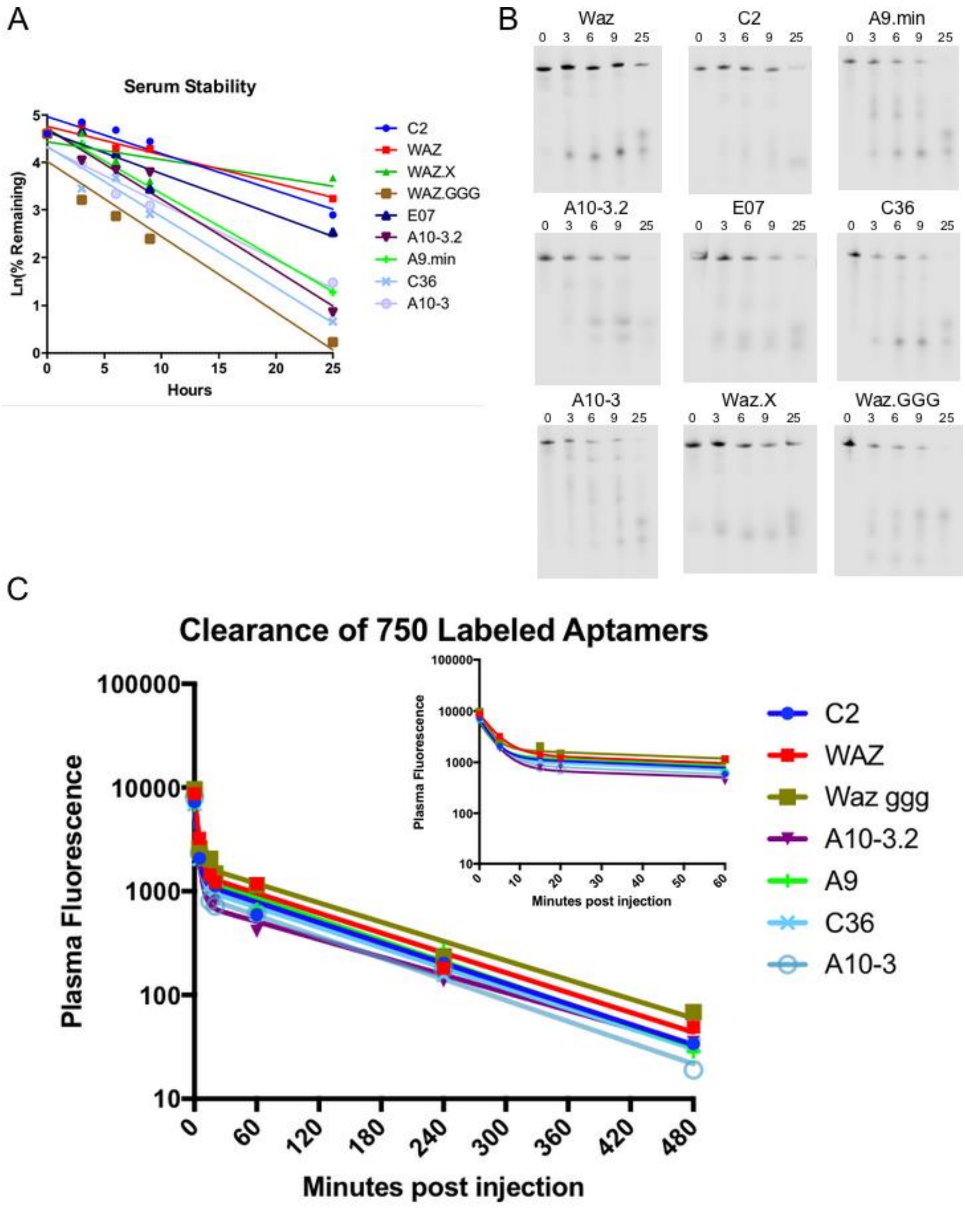

Supplementary Fig. 191. Aptamer serum stability and plasma clearance rates. (**A** and **B**) Aptamers labeled with AF750 were incubated in whole mouse serum for 3, 6, 9, and 25 hours to assess serum stability. The recovered aptamers were run on a denaturing (7M urea) 12% polyacrylamide gel and read on an Odyssey LI-COR imager. The % full length aptamer was quantified using the LI-COR Lite Imaging Suite and the rate of degradation was determined. Time 0 served as size marker for full length aptamer.**(C)** Plasma clearance was determined as described in the **SupplementaryMethods**. Primary and secondary pharmacokinetic parameters were determined from analysis of the concentration vs. time data in the context of a biphasic, two compartment model and are given in **Table S3**. Data representative of 3 experimental replicates.

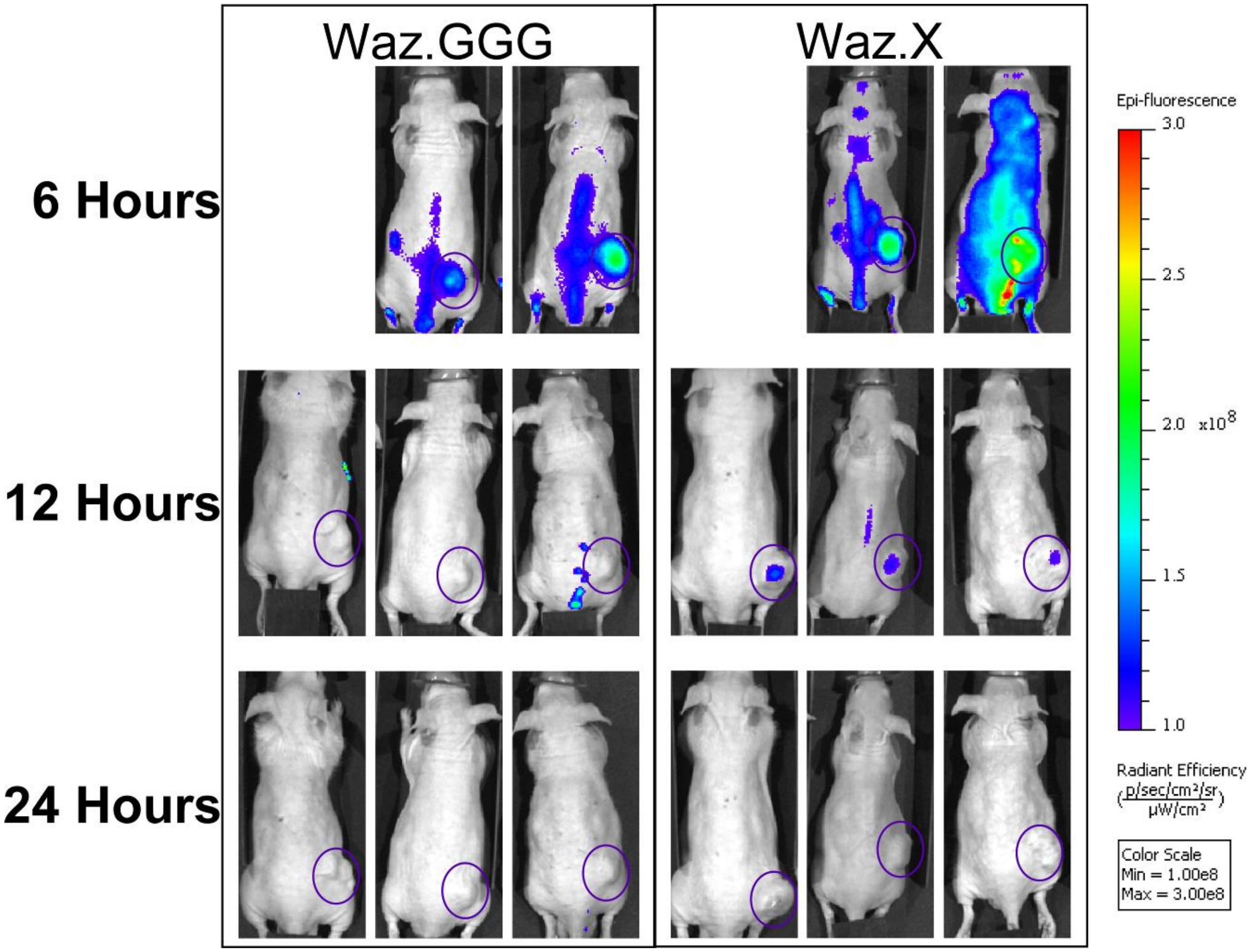

Supplementary Fig. 192. In vivo imaging of AF750 labeled Waz mutants in mice bearing 22Rv1 tumors, time course. To further confirm specificity of anti-hTfR aptamer Waz, mice bearing 22Rv1 tumors were injected with the mutant forms of Waz: Waz.X and Waz.GGG. Mice were imaged at 6, 12, and 24 hours (n = 3).

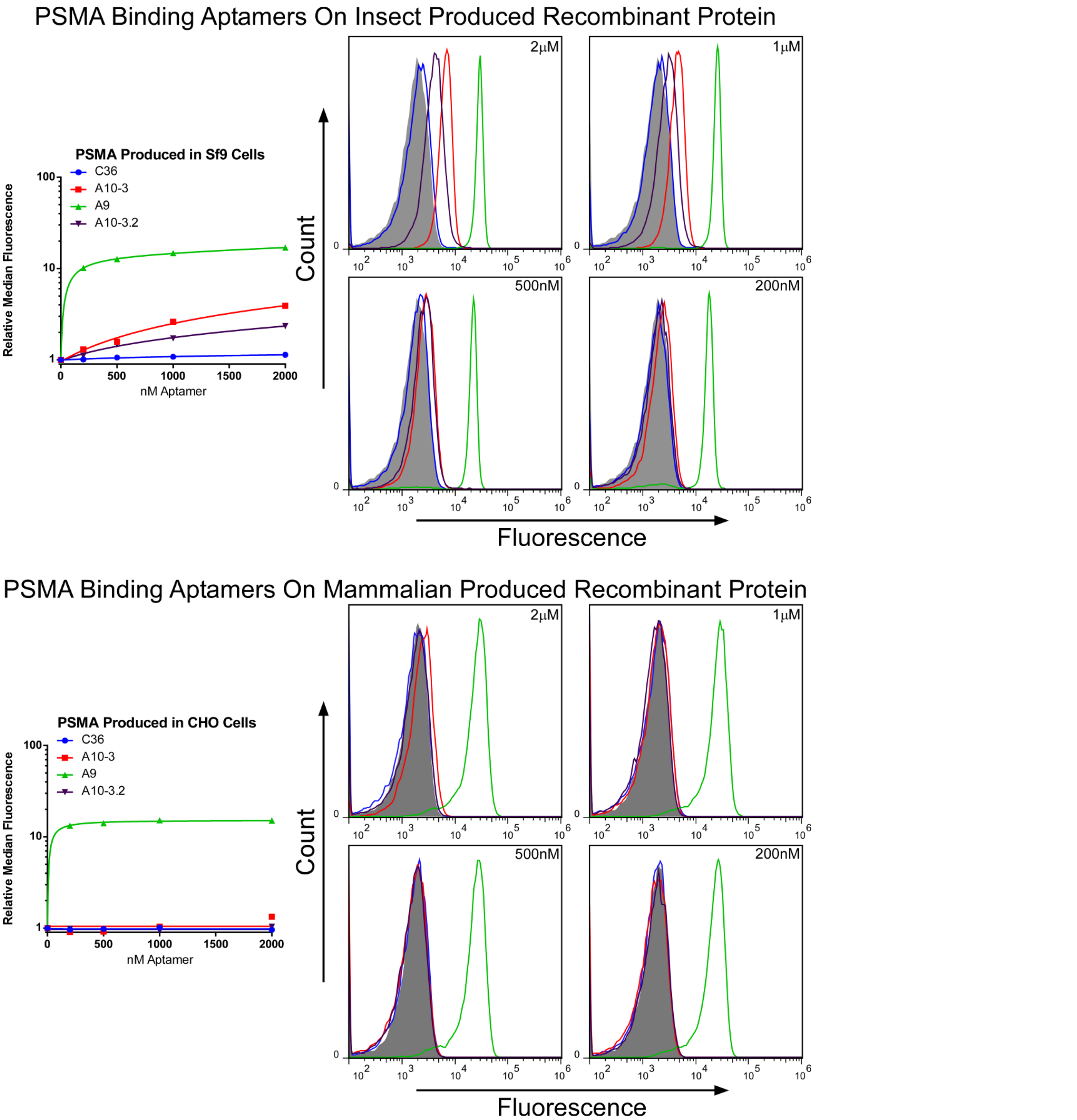

Supplementary Fig. 193. Recombinant PSMA, produced in insect cells (top) or mammalian CHO cells (bottom), was immobilized on Dynal, 1 um His-Tag Isolation magnetic beads at ~1ug protein per 2.5uL of beads. After washing the beads were subsequently stained with increasing concentrations of the reported PSMA specific aptamers, A9.min, A10-3, A10-3.2 or a non-targeted control, C36, washed and analyzed by flow cytometry.

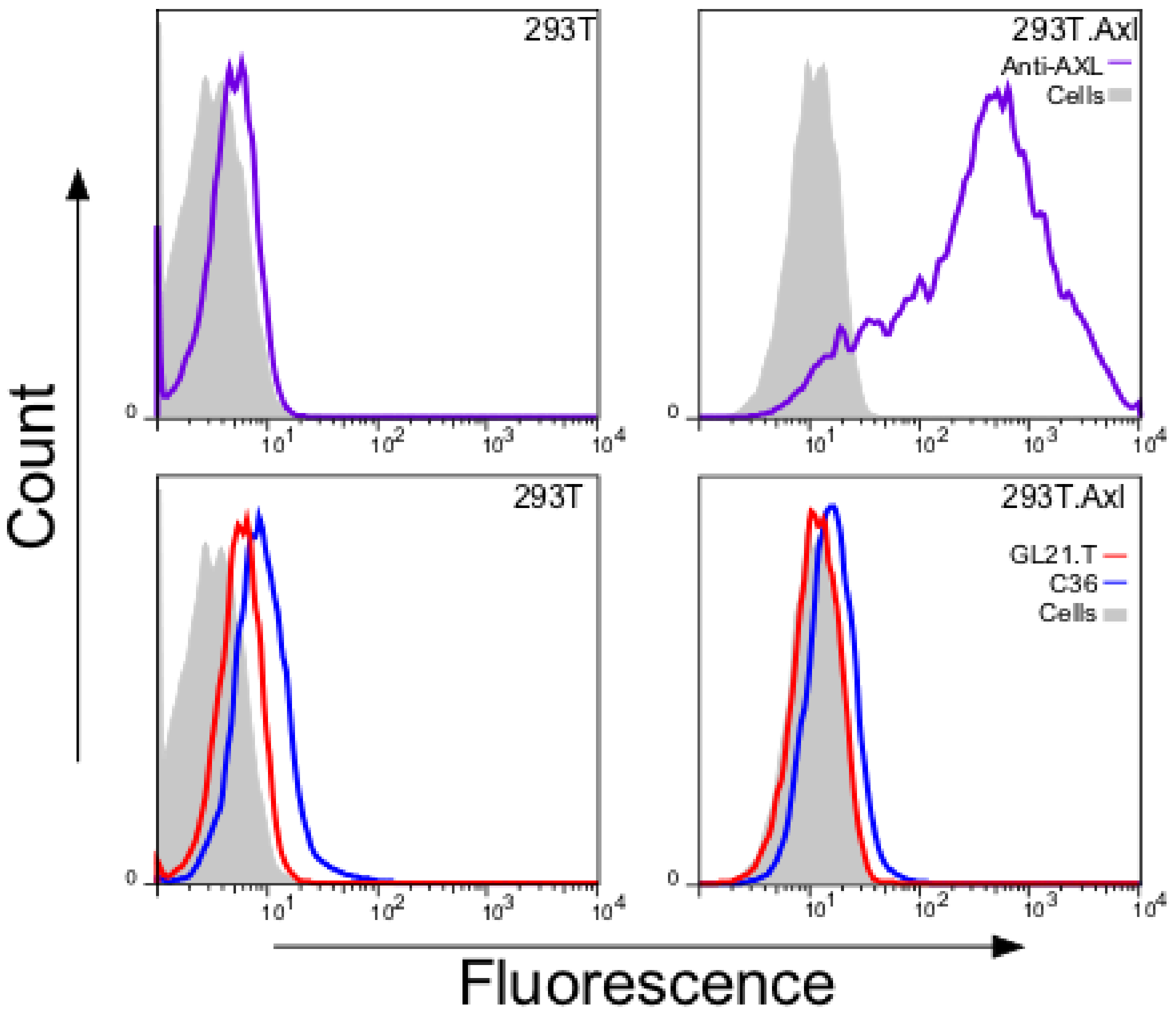

Supplementary Fig. 194. Additional analysis of the AXL aptamer, GL21.T. 293T cells were stably transfected to express AXL. The cells showed considerable staining when stained with an AXL specific antibody labeled with APC (R&D Systems, Minneapolis MN). However, 293T.AXL cells stained using our binding protocol with the anti-AXL aptamer GL21.T at 500 nM did not show any increase in fluorescence above a non-targeted control (C36). Similar results were observed when cell staining experiments were performed using our internalization and binding protocol.

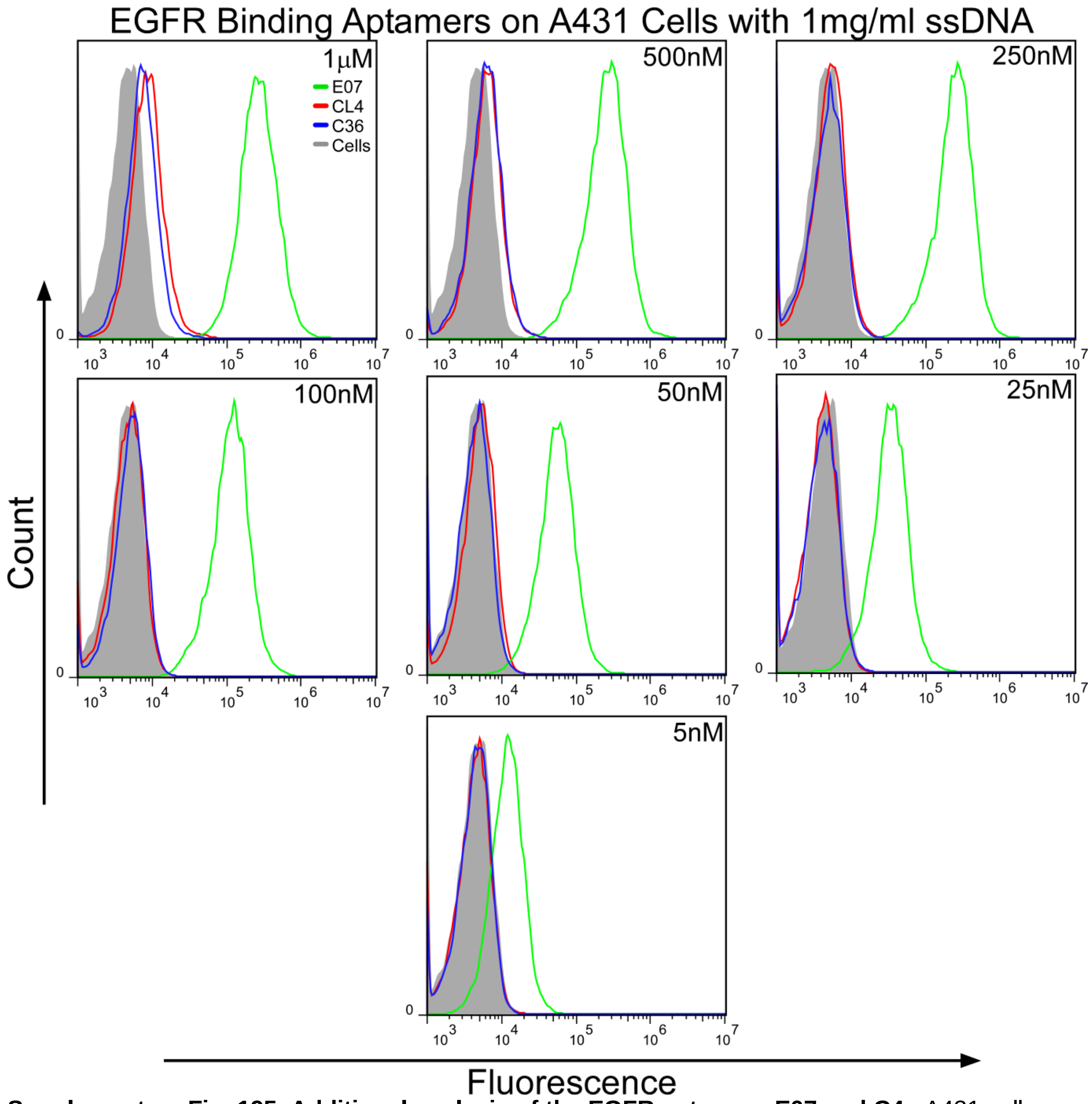

Supplementary Fig. 195. Additional analysis of the EGFR aptamers E07 and C4. A431 cells are an EGFR high expressing cell line. When stained by our internalization and binding assay, E07 demonstrates a strong fluorescence signal at concentrations as low as 5nM. The CL4 aptamer however stains the cells similar to a nonspecific control.

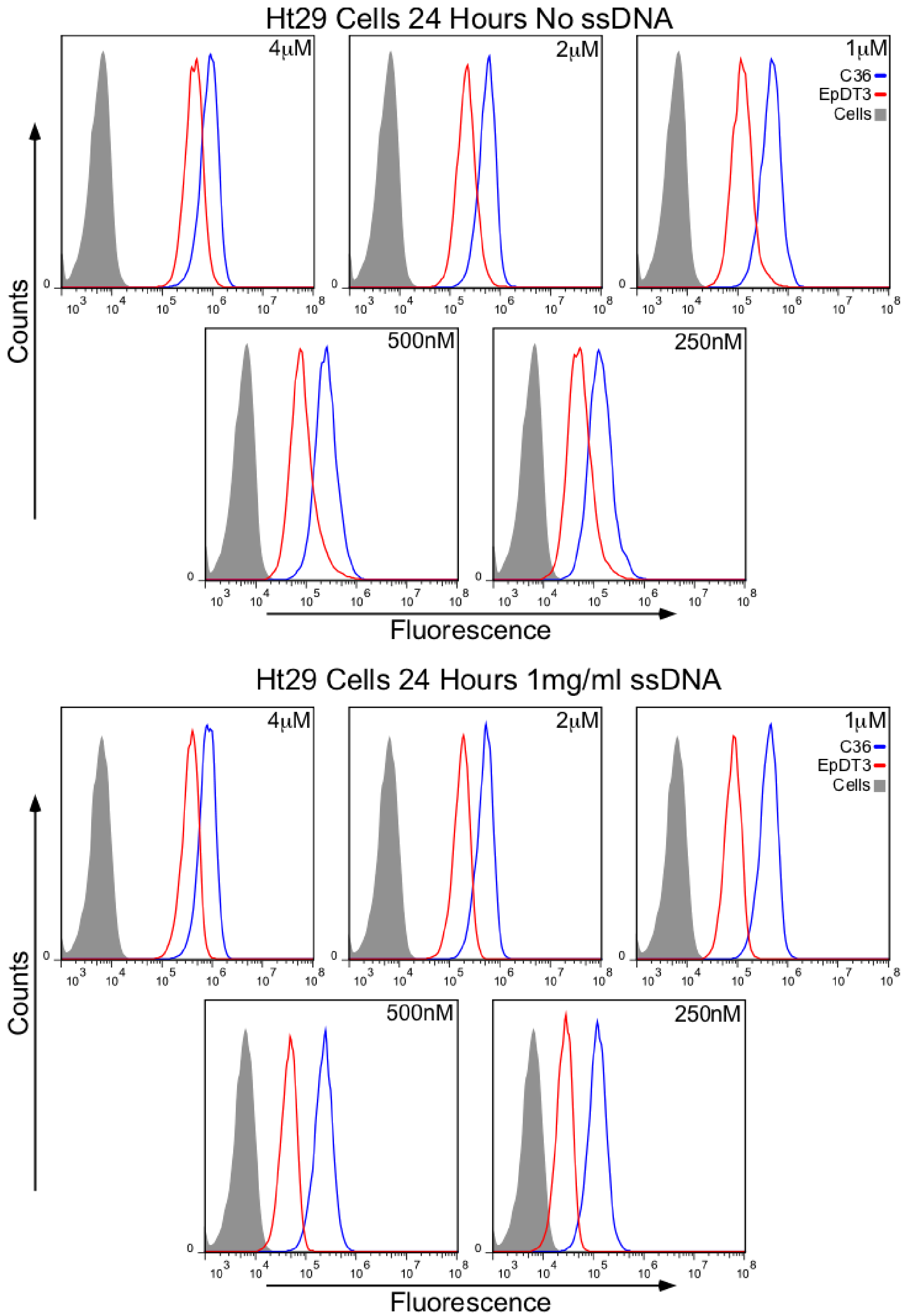

Supplementary Fig. 196. Additional analysis of the EpCAM aptamer EpDT3. HT29 cells which are EPCAM high expressing cells (see **Figure 3K**) were incubated in full growth media with labeled EpDT3 aptamer or a nonspecific control for 24 hours at 37°C. The EpDT3 stained cells showed similar levels of staining to the nonspecific control both in the absence (top) and presence (bottom) of blocking agents.

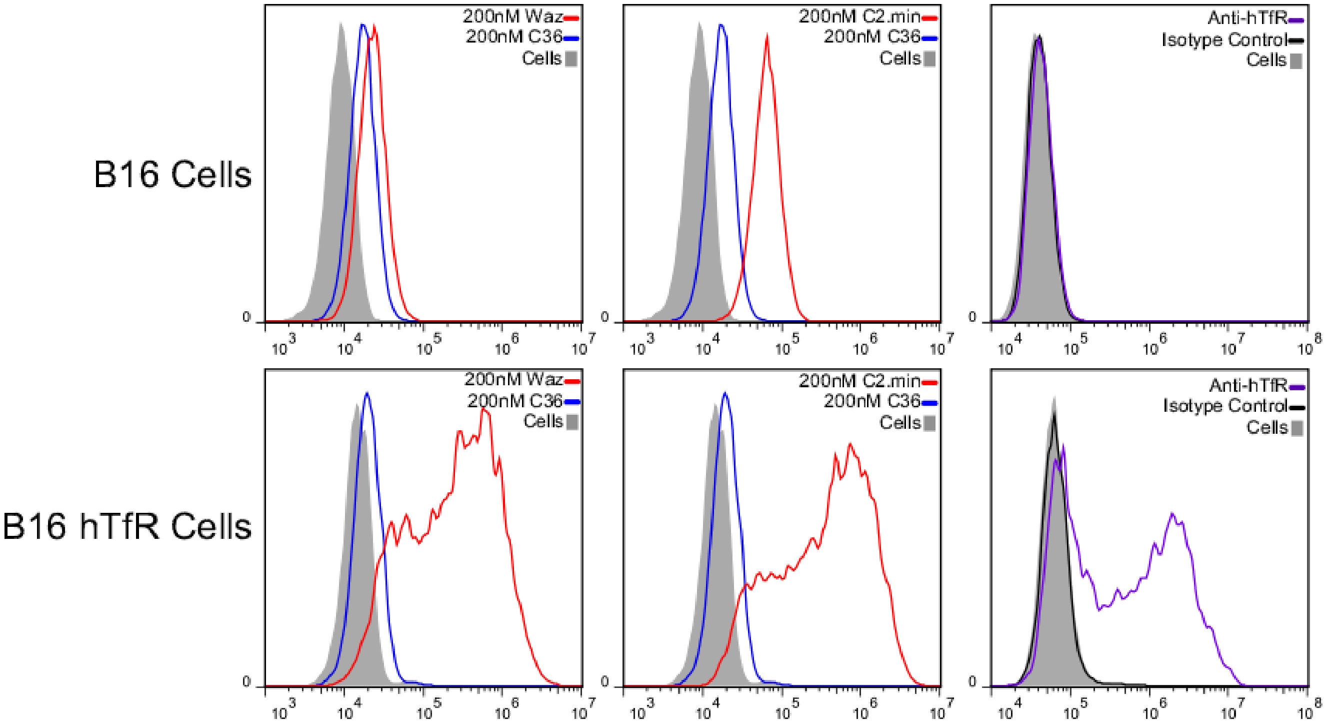

Supplementary Fig. 197. Additional analysis of anti-hTfR aptamers Waz and C2.min. Waz and C2.min were assayed by our internalization and binding assay in the presence of 1 mg/ml ssDNA. Both aptamers were selected against the human protein and show strong staining on B16 cells engineered to express hTfR (B16-hTfR cells). Waz demonstrates only background binding levels on parental B16 cells which express mTfR, but no hTfR. However, C2 does demonstrate some binding above background on the mouse cell line B16 suggesting it cross reacts to some degree with the murine protein.

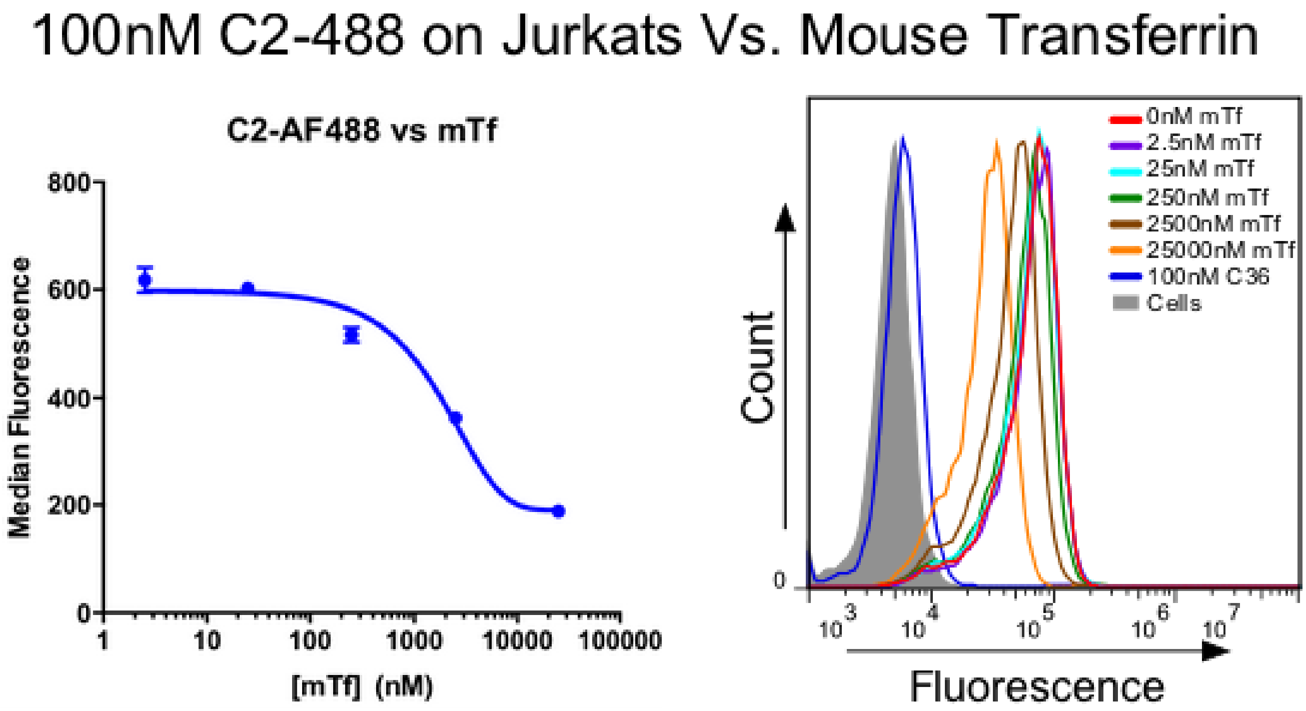

Supplementary Fig. 198. Supraphysiological levels of mouse transferrin is able to compete with C2 for binding to the human receptor. Increasing concentrations of the mTfR protein added to media result in a decrease in fluorescence staining on Jurkat cells stained with 100 nM C2-AF488. However, at 25 µM mTfR, significant cell staining by C2-AF488 is still observed, whereas similar experiments performed against hTfR reduced C2.min staining on this cell line to near background levels (1).

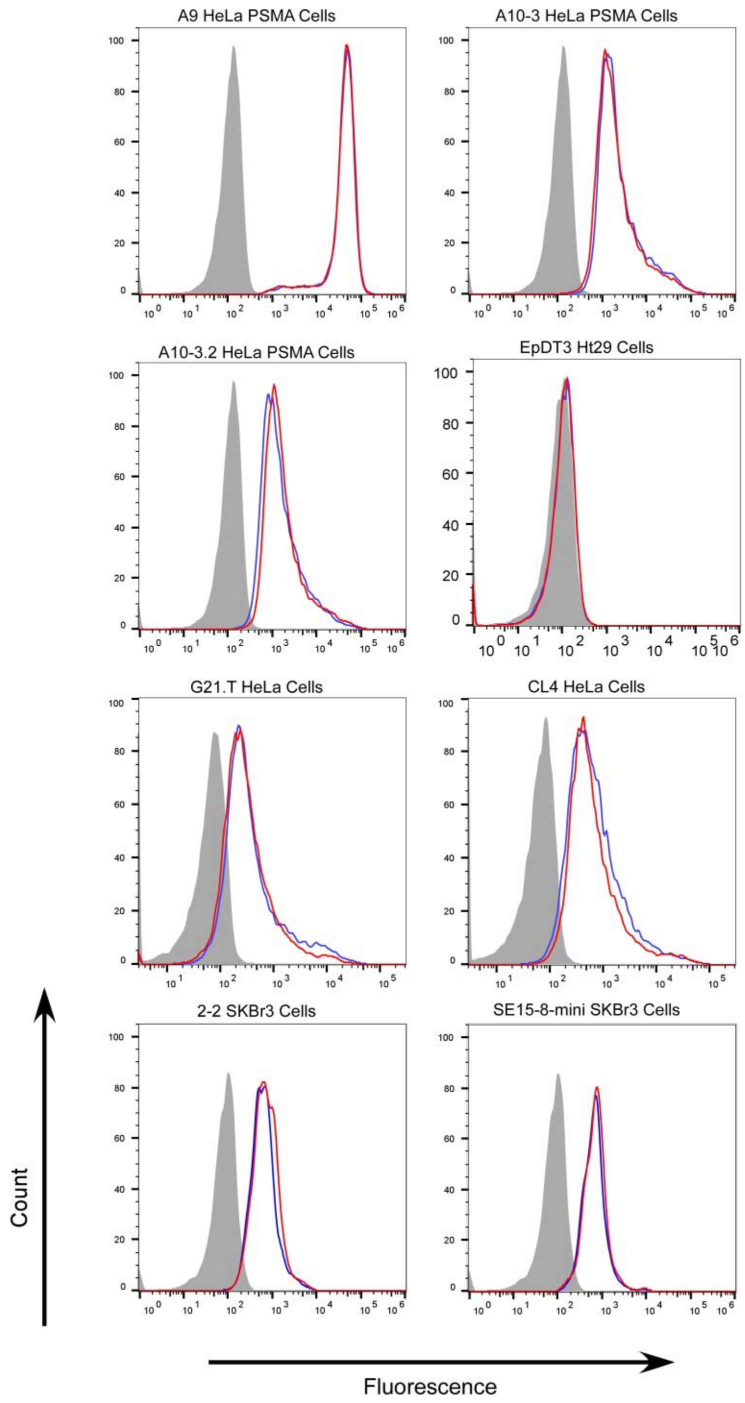

Supplementary Fig. 199. Aptamers were refolded according to conditions and buffers of initial selection paper. All assays were performed at 500 nM aptamer. Grey solid curves represent unstained cells. Red lines indicate aptamers refolded under our standard conditions (DPBS). Blue lines represent aptamers refolded using the published literature buffer and procedure. Folding conditions are listed in **Table S4**.

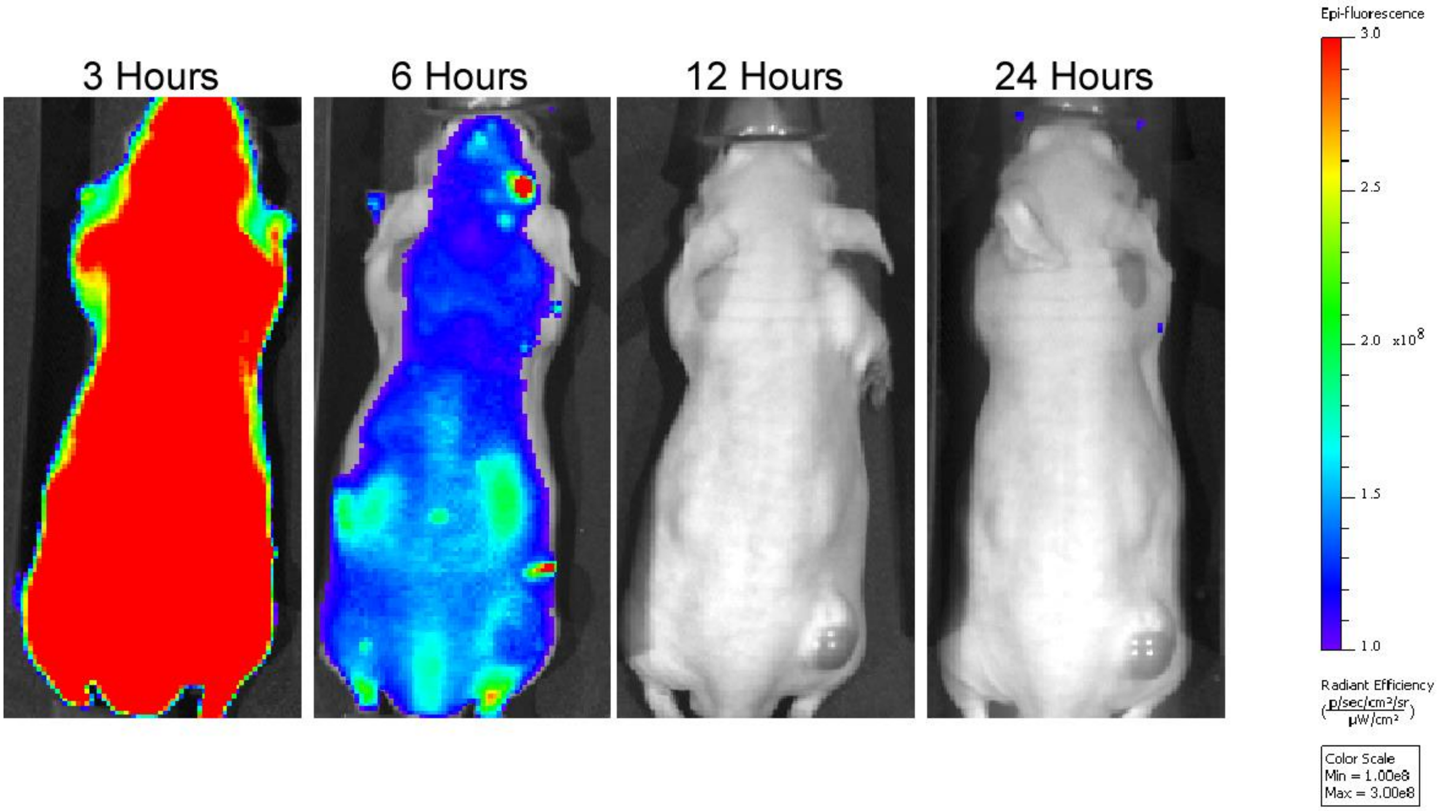

Supplementary Fig. 200. In vivo imaging of AF750 labeled A9 using an amide linked fluorophore in mice bearing 22RV1 tumors. Mice were imaged at 3, 6, 12 and 24 hours post injection of aptamers.

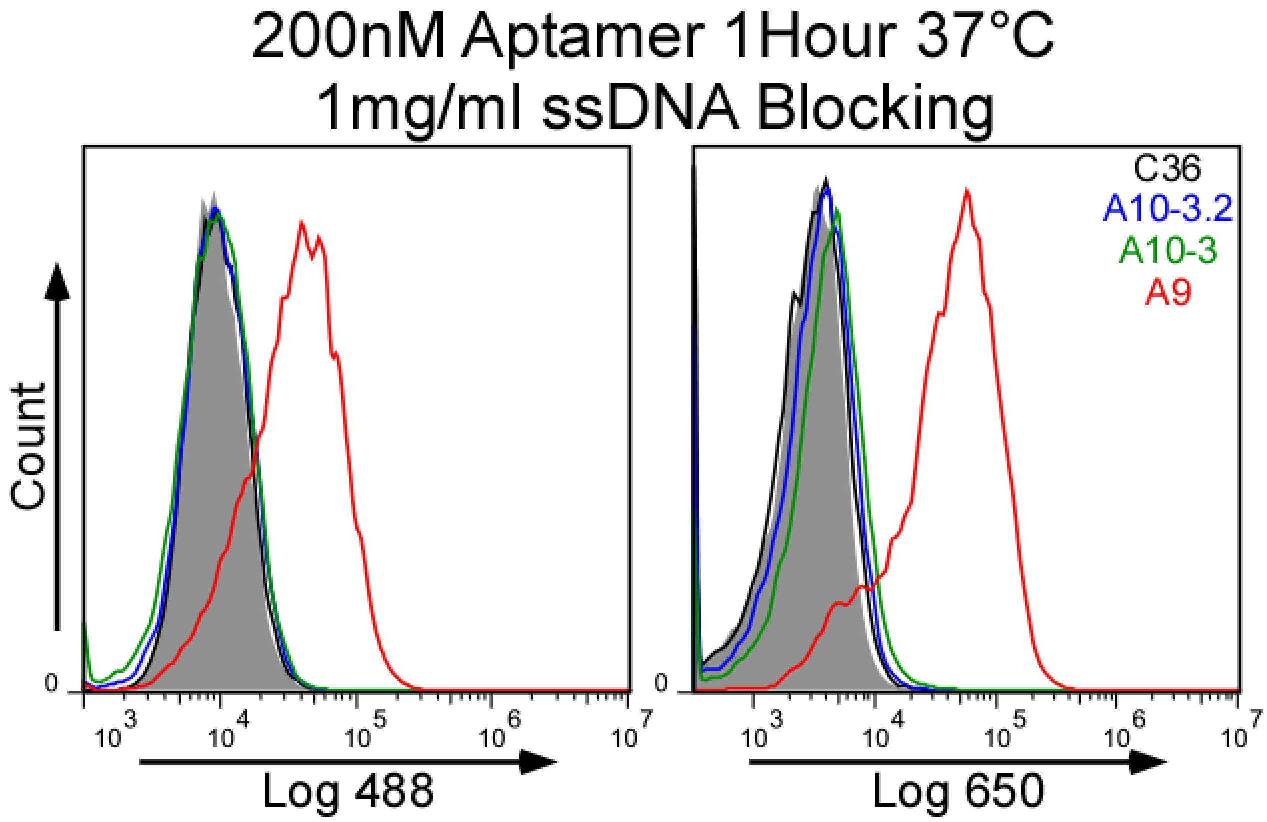

Supplementary Fig. 201. Reported PSMA binding aptamers were labeled with AF488 via thiol maleimide reaction. LnCAP cells were assayed by internalization and binding assay conditions. AF488 labeled aptamers (left panel) displayed a similar binding pattern to Dy 650 labeled aptamers (right panel).

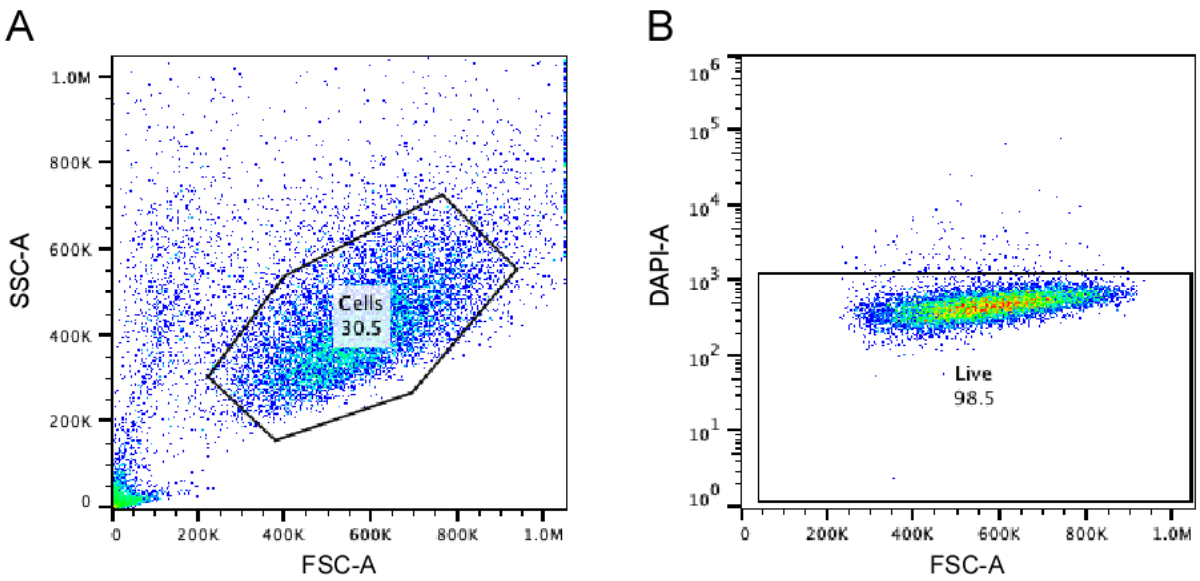

Supplementary Fig. 202. For flow-based assays all cells were gated first on whole cell population of unstained cells (Panel A) to remove debris. The whole cells gate was then gated based on live/dead dye, bisbenzimide (Panel B) to remove any dead cells from analysis. Histograms and median fluorescence values reported are based off this second gate. This figure shows gating on HeLa cells.

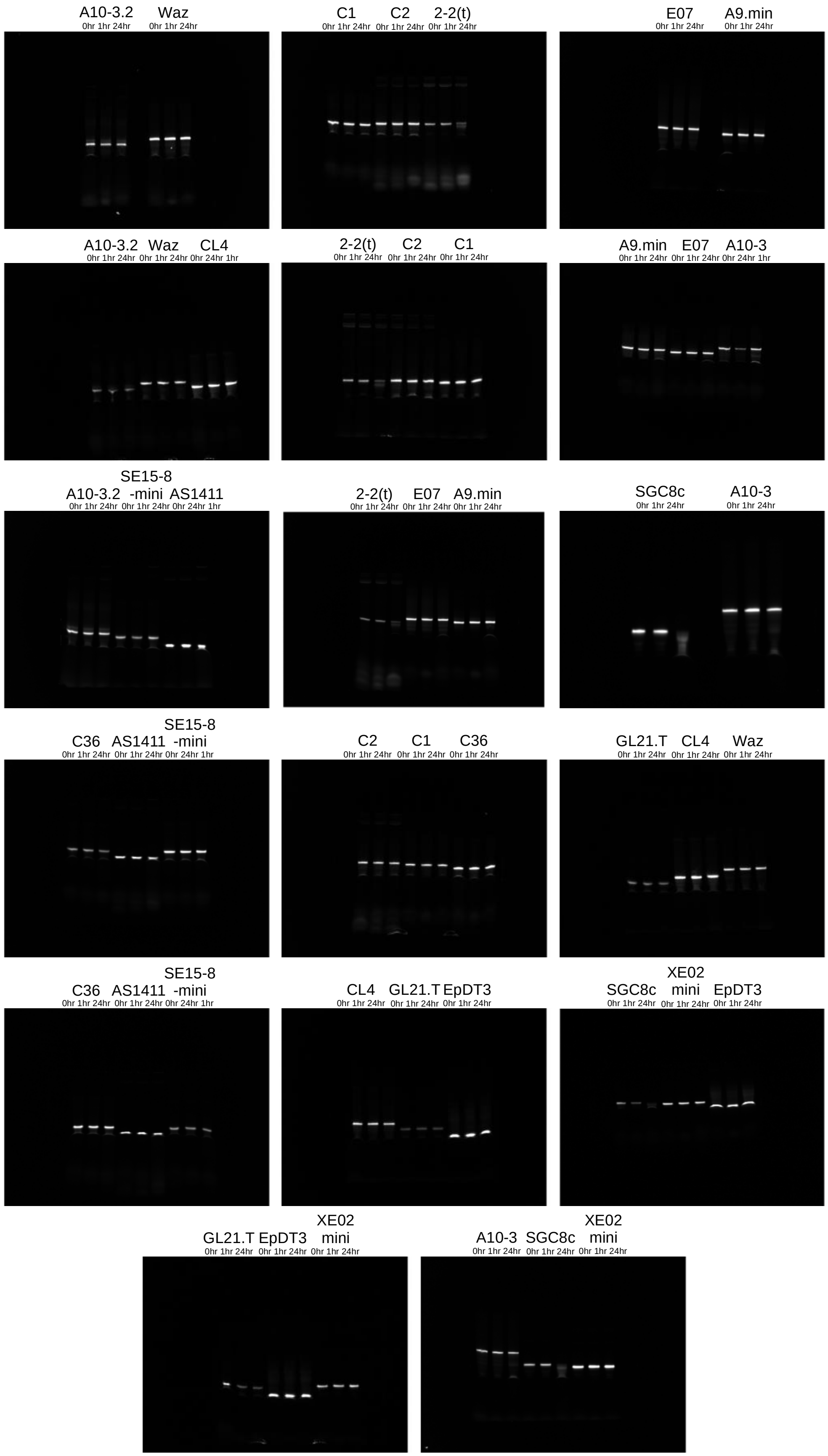

Fig S203. Uncropped gels used to generate supplementary figure 7.

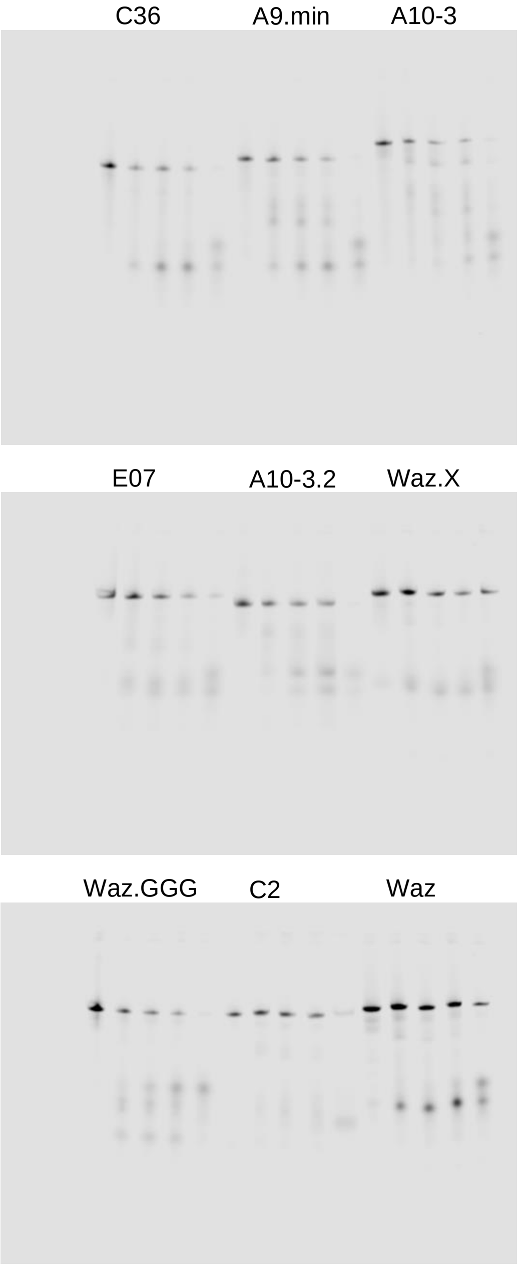

Fig S204. Uncropped gels from supplementaryfigure 191.

References:

1. Wilner SE, *et al.* (2012) An RNA alternative to human transferrin: a new tool for targeting human cells. *Mol Ther Nucleic Acids* 1:e21.
2. Semple SC, *et al.* (2010) Rational design of cationic lipids for siRNA delivery. *Nat Biotechnol* 28(2):172-176.
