# Supplementary material for: A comparative analysis of cell surface targeting aptamers
Source: Nat Commun. 2021 Nov 1;12:6275. doi: 10.1038/s41467-021-26463-w (PMC8560833; doi:10.1038/s41467-021-26463-w)
Supplement: Supplementary file 2 — Reporting Summary [file 41467_2021_26463_MOESM2_ESM.pdf]

nature research

Corresponding author(s): Matthew Levy

Last updated by author(s): 2021-30-09

Reporting Summary

Nature Research wishes to improve the reproducibility of the work that we publish. This form provides structure for consistency and transparency in reporting. For further information on Nature Research policies, see our Editorial Policies and the Editorial Policy Checklist.

Statistics

For all statistical analyses, confirm that the following items are present in the figure legend, table legend, main text, or Methods section.

n/a	Confirmed	
☐	☒	The exact sample size (n) for each experimental group/condition, given as a discrete number and unit of measurement
☐	☒	A statement on whether measurements were taken from distinct samples or whether the same sample was measured repeatedly
☐	☒	The statistical test(s) used AND whether they are one- or two-sided<br>*Only common tests should be described solely by name; describe more complex techniques in the Methods section.*
☒	☐	A description of all covariates tested
☒	☐	A description of any assumptions or corrections, such as tests of normality and adjustment for multiple comparisons
☐	☒	A full description of the statistical parameters including central tendency (e.g. means) or other basic estimates (e.g. regression coefficient) AND variation (e.g. standard deviation) or associated estimates of uncertainty (e.g. confidence intervals)
☐	☒	For null hypothesis testing, the test statistic (e.g. F, t, r) with confidence intervals, effect sizes, degrees of freedom and P value noted<br>*Give P values as exact values whenever suitable.*
☒	☐	For Bayesian analysis, information on the choice of priors and Markov chain Monte Carlo settings
☒	☐	For hierarchical and complex designs, identification of the appropriate level for tests and full reporting of outcomes
☐	☒	Estimates of effect sizes (e.g. Cohen's d, Pearson's r), indicating how they were calculated

Our web collection on statistics for biologists contains articles on many of the points above.

Software and code

Policy information about availability of computer code

Data collection: Icyt Eclipse. Sony SA3800. IVIS Spectrum Imaging System. Odyssey DLx Imager

Data analysis: FlowJo v 7.6.3, GraphPad Prism version 6,7&9, Living Image Version 4.0, Excel, Image Studio Lite v 5.1

For manuscripts utilizing custom algorithms or software that are central to the research but not yet described in published literature, software must be made available to editors and reviewers. We strongly encourage code deposition in a community repository (e.g. GitHub). See the Nature Research guidelines for submitting code & software for further information.

Data

Policy information about availability of data

All manuscripts must include a data availability statement. This statement should provide the following information, where applicable:

- Accession codes, unique identifiers, or web links for publicly available datasets
- A list of figures that have associated raw data
- A description of any restrictions on data availability

All data generated or analyzed during this study are included in this published article (and its supplementary information files). Raw data (cytometry files) generated during and/or analyzed during this study are also available from the corresponding author on reasonable request.

Field-specific reporting

Please select the one below that is the best fit for your research. If you are not sure, read the appropriate sections before making your selection.

- [x] Life sciences
- [ ] Behavioural & social sciences
- [ ] Ecological, evolutionary & environmental sciences

For a reference copy of the document with all sections, see nature.com/documents/nr-reporting-summary-flat.pdf

Life sciences study design

All studies must disclose on these points even when the disclosure is negative.

Sample size	Required experimental sample sizes were chosen according to common practices (three independent experiments, as indicated in the figure legends). Statistical analysis was limited to determining mean +/- SD and is indicated by error bars reported for each experiment. For animal imaging, 6 animals was used per group to provide a data set sufficient to demonstrate targeting specificity. Data collected from all animals in each group is shown. (n=3 within the manuscript and n= 3 in the supplement) Statistical power was not considered prior to choosing sample size.
Data exclusions	No data was excluded from the analyses in this study.
Replication	The number of replicates for all experiments are stated in the figure legends.
Randomization	Randomization was not employed for animals studies. Tumors were size matched as described in the methods section In vivo NIR imaging. Tumor bearing mice were distributed among groups based on size to minimize biases due to tumor growth and size. Randomization of samples is not relevant or necessary for all other studies performed in this work.
Blinding	Blinding for imaging studies was not necessary. Imaging provides unbiased quantitative data for each animal treated. It was also not required for any of the other quantitate biological analyses performed in this works as all of the data collected relies on direct quantitative measurement an is therefore not susceptible to any bias during collection.

Reporting for specific materials, systems and methods

We require information from authors about some types of materials, experimental systems and methods used in many studies. Here, indicate whether each material, system or method listed is relevant to your study. If you are not sure if a list item applies to your research, read the appropriate section before selecting a response.

Materials & experimental systems

n/a	Involved in the study	
☐	☒	Antibodies
☐	☒	Eukaryotic cell lines
☒	☐	Palaeontology and archaeology
☐	☒	Animals and other organisms
☒	☐	Human research participants
☒	☐	Clinical data
☒	☐	Dual use research of concern

Methods

n/a	Involved in the study	
☒	☐	ChIP-seq
☐	☒	Flow cytometry
☒	☐	MRI-based neuroimaging

Antibodies

Antibodies used	Alexa Fluor 488 labeled antibodies and isotype controls ordered from Sony Biotechnology included, Anti-EGFR antibody (Cat #2364535), Anti-EpCAM antibody (Cat #2221045), Anti-PSMA antibody (Cat #2312525), Anti-HER2 antibody (Cat #2222050), Isotype control (Cat #2600660 and #2601645). Alexa Fluor 488 labeled anti-AXL antibody was ordered from R&D systems (Cat #FAB154G). Unlabeled anti-hTfR antibody was ordered from BD systems (Cat # 555534) and anti-PTK7 was ordered from miltenyi biotec (Cat #130-091-578). Unlabeled antibodies were labeled with amine reactive Alexa Fluor 488 from Life technologies (Cat # A20000). All antibodies were titrated on cell lines previously reported to express their respective targets. All antibodies demonstrated activity at the vendor recommended concentrations. Subsequent studies were therefore performed at the vendor recommended concentrations. A full list of antibodies used is provided in the supplemental methods.
Validation	All antibodies were provided with validation, provided by the vendor. Additionally, in the course of the work performed in generation of our manuscript, we used siRNA to knock down the specific target for each of these antibodies and used flow cytometry to confirm their specificity (see Fig 4.) Data sheets for each antibody used in our studies have been now been provided in a zipped folder (Antibody product sheets.zip).

Eukaryotic cell lines

Policy information about cell lines

Cell line source(s)	The following cell lines were used: Jurkat, LNCaP, 22RV1, SKBR3, HeLa, HeLa-PSMA (HeLa cells stably transfected to express human PSMA), MCF7, A431, and HT29, PC3, PC3-PSMA (PC3 cells stably transfected to express human PSMA), A549, HEK293T and HEK293T-Axl (HEK293T cells stably transfected to express human Axl). All cell lines were obtained from ATCC. Stable cell lines expressing specific receptors were generated in house from parental cell lines obtained from the ATCC Maintenance and stable transfection references are listed in the methods section.
Authentication	All cell lines were obtained from the ATCC and supplied with authentication upon receipt from the vendor.
Mycoplasma contamination	All cell lines were routinely checked for mycoplasma contamination using the MycoAlert mycoplasma detection kit (Lonza, Basel, Switzerland). No contamination was observed during any point of our studies.
Commonly misidentified lines (See ICLAC register)	No commonly misidentified cell lines were used in this study.

Animals and other organisms

Policy information about studies involving animals; ARRIVE guidelines recommended for reporting animal research

Laboratory animals	Male Nu/Nu nude mice were purchased from Charles River (Kingston, NY) at 4 weeks of age for tumor studies. C57BL/6 mice were originally purchased from Taconic (Hudson, NY) and bred in house for use in serum stability studies and plasma clearance studies. Male and female animals were between 8 and 14 weeks of age.
Wild animals	No wild animals were used in this study.
Field-collected samples	No field-collected samples were used in this study.
Ethics oversight	Einstein Institutional Animal Care and Use Committee.

Note that full information on the approval of the study protocol must also be provided in the manuscript.

Flow Cytometry

Plots

Confirm that:

- [x] The axis labels state the marker and fluorochrome used (e.g. CD4-FITC).
- [x] The axis scales are clearly visible. Include numbers along axes only for bottom left plot of group (a 'group' is an analysis of identical markers).
- [x] All plots are contour plots with outliers or pseudocolor plots.
- [x] A numerical value for number of cells or percentage (with statistics) is provided.

Methodology

Sample preparation	Cells grown in plates were lifted by brief treatment with trypsin or EDTA prior to analysis.
Instrument	Analysis was performed using a Eclipse EC800 or Sony SA3800
Software	All flow analysis was performed using FlowJo.
Cell population abundance	All flow analysis was preformed on culture cell lines. Cells tested were grown as monocultures. A minimum of 5,000 cells were counted for each experiment.
Gating strategy	Cell populations were gates for live cells only using bisbenzimide to exclude dead cells.

- [x] Tick this box to confirm that a figure exemplifying the gating strategy is provided in the Supplementary Information.
